# Identifying climate refugia for high‐elevation Alpine birds under current climate warming predictions

**DOI:** 10.1111/gcb.16187

**Published:** 2022-04-20

**Authors:** Mattia Brambilla, Diego Rubolini, Ojan Appukuttan, Gianpiero Calvi, Dirk Nikolaus Karger, Primož Kmecl, Tomaž Mihelič, Thomas Sattler, Benjamin Seaman, Norbert Teufelbauer, Johannes Wahl, Claudio Celada

**Affiliations:** ^1^ Lipu/BirdLife Italia Parma Italy; ^2^ MUSE–Museo delle Scienze, Sezione Zoologia dei Vertebrati Trento Italy; ^3^ Fondazione Lombardia per l’Ambiente, Settore Biodiversità e aree protette Milano Italy; ^4^ 9304 Dipartimento di Scienze e Politiche Ambientali Università degli Studi di Milano Milano Italy; ^5^ Istituto di Ricerca sulle Acque, IRSA‐CNR Brugherio Italy; ^6^ Studio Pteryx Basiano Italy; ^7^ Swiss Federal Institute for Forest, Snow and Landscape Research (WSL) Birmensdorf Switzerland; ^8^ DOPPS‐BirdLife Slovenia Ljubljana Slovenia; ^9^ Schweizerische Vogelwarte Sempach Switzerland; ^10^ BirdLife Austria Vienna Austria; ^11^ Dachverband Deutscher Avifaunisten (DDA) Münster Germany

**Keywords:** Alps, climate change, community science, distribution, ecological realism, protected areas, SDM extrapolation

## Abstract

Identifying climate refugia is key to effective biodiversity conservation under a changing climate, especially for mountain‐specialist species adapted to cold conditions and highly threatened by climate warming. We combined species distribution models (SDMs) with climate forecasts to identify climate refugia for high‐elevation bird species (*Lagopus muta*, *Anthus spinoletta*, *Prunella collaris*, *Montifringilla nivalis*) in the European Alps, where the ecological effects of climate changes are particularly evident and predicted to intensify. We considered future (2041–2070) conditions (SSP585 scenario, four climate models) and identified three types of refugia: (1) in‐situ refugia potentially suitable under both current and future climate conditions, ex‐situ refugia suitable (2) only in the future according to all future conditions, or (3) under at least three out of four future conditions. SDMs were based on a very large, high‐resolution occurrence dataset (2901–12,601 independent records for each species) collected by citizen scientists. SDMs were fitted using different algorithms, balancing statistical accuracy, ecological realism and predictive/extrapolation ability. We selected the most reliable ones based on consistency between training and testing data and extrapolation over distant areas. Future predictions revealed that all species (with the partial exception of *A. spinoletta*) will undergo a range contraction towards higher elevations, losing 17%–59% of their current range (larger losses in *L. muta*). We identified ~15,000 km^2^ of the Alpine region as in‐situ refugia for at least three species, of which 44% are currently designated as protected areas (PAs; 18%–66% among countries). Our findings highlight the usefulness of spatially accurate data collected by citizen scientists, and the importance of model testing by extrapolating over independent areas. Climate refugia, which are only partly included within the current PAs system, should be priority sites for the conservation of Alpine high‐elevation species and habitats, where habitat degradation/alteration by human activities should be prevented to ensure future suitability for alpine species.

## Graphical abstract

By combining distribution models (based on a large, high‐resolution dataset, and balancing statistical accuracy, ecological realism, and predictive/extrapolation ability) with climate forecasts, this study identified climate refugia for high‐elevation bird species (*Lagopus muta*, *Anthus spinoletta*, *Prunella collaris*, *Montifringilla nivalis*) in the European Alps, considering both in‐situ refugia (suitable now and in the future) and ex‐situ refugia (suitable only in the future). Most species will undergo a marked contraction towards higher elevations. In‐situ refugia for at least three species cover ~15,000 km^2^ of the Alpine region (44% included within protected areas) and represent priority sites for the conservation of high‐elevation species and habitats.

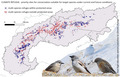

## 1 INTRODUCTION

Anthropogenic climate change is dramatically threatening biodiversity as well as key ecosystem services, both directly and indirectly (e.g., Weiskopf et al., 2020). Mountain regions are particularly sensitive to climatic change because they are home to specialized, cold‐adapted organisms that are tightly linked to the steep environmental gradients occurring in these areas, and temperature warming has been especially pronounced at higher elevations (Pepin et al., 2015). Mountains systems harbor rich biodiversity worldwide (Körner & Ohsawa, 2006), and—although they only cover ~25% of Earth's land surface—they host nearly half the planet's terrestrial biodiversity hotspots (Myers et al., 2000) and are home to a high percentage of endemic species (Essl et al., 2009).

Many alpine species are adapted to cope with the harsh environmental conditions typically found at high elevations (Bettega et al., 2020; Cheviron & Brumfield, 2012; Lu et al., 2009). Because of their tight link with such conditions, these species may be particularly vulnerable to environmental changes, which could dramatically impact their populations (Martin & Wiebe, 2004; Tingley et al., 2009). In particular, the frequent additive or even synergistic effects of climate change, land‐abandonment, and human‐driven habitat alterations (Barras et al., 2020, 2021; Mantyka‐Pringle et al., 2012) are threatening a wide range of species, and especially those linked to mountain grassland and pastures (Brambilla, Gustin, et al., 2020; Chamberlain et al., 2016; García‐Navas et al., 2020). Thus, climate change effects in high elevation regions, where large environmental changes are occurring, which may be both direct and indirect (Brambilla et al., 2016). Birds track climate and environmental changes in general, and are excellent models for investigating the impacts of climate change on mountain biodiversity (e.g., Chamberlain et al., 2013; Lehikoinen et al., 2019). In pyramidal mountain systems such as the European Alps, cold‐adapted species will undergo range contraction even in the case of perfect climate‐tracking, because of the shape of the mountain massif (cf. Elsen & Tingley, 2015), and will also experience increasing isolation of residual suitable patches (Brambilla et al., 2017; Jackson et al., 2015).

The strong pressures acting on alpine species call for conservation strategies that explicitly take the potential consequences of environmental changes into account and integrate them into landscape and conservation planning (Groves et al., 2012; Hannah, 2011; Williams et al., 2005). For some species, the loss of suitable habitat through climate change may be compensated by the colonization of new areas that become suitable in the future (Pearson et al., 2002). The dispersal abilities of avian taxa may allow them to track suitable climates and environments better than many other organisms—at least over relatively fine geographical scales, such as within a mountain system. Nearly all strategies of climate change adaptation include resilience as a key concept (Morecroft et al., 2012); for conservation purposes, we can consider resilient populations as those that are able to recover when favorable conditions are re‐established (Harrison, 1979). Resistant systems are instead able to remain essentially unchanged despite disturbance (Grimm & Wissel, 1997). For conservation planning in a changing climate, we can define resistant populations or units as those that are expected to remain largely unaffected by climate change, at least with respect to their distribution (Brambilla et al., 2017; Ficetola et al., 2016). Similarly, resilient units in this context are those that can allow the re‐establishment of populations under future conditions (Brambilla et al., 2017; Vos et al., 2008).

Resistant and resilient units can be associated with in‐situ and reachable ex‐situ climate refugia, respectively (see, e.g., Hannah, 2011; Yang et al., 2022). The identification and preservation of the ecological integrity of in‐situ refugia, i.e., currently occupied areas expected to remain suitable in the future, and ex‐situ refugia, i.e., areas not currently occupied but expected to become suitable with changing environmental conditions, is indeed a key requirement for effective biodiversity conservation strategies in a changing climate (Keppel et al., 2012). On the one hand, resistance‐only approaches (focusing exclusively on in‐situ refugia) may fail because they may ignore the potential detrimental effects of increasing isolation, and the risks of identifying areas too small and isolated to allow long‐term species’ persistence (Verboom et al., 2001). On the other hand, resilience‐only strategies based on ex‐situ refugia may fail to consider the overwhelming importance of in‐situ refugia for population persistence (Ficetola et al., 2016; Keppel et al., 2012). Integrating both these aspects in conservation planning may maximize the chances of maintaining high levels of biodiversity in a changing climate.

Using citizen‐science data, we aimed to identify future climate refugia across the entire Alpine mountain system for a group of climate‐sensitive avian species dependent on high‐elevation open habitats. We focused on four high Alpine species (rock ptarmigan *Lagopus muta*, water pipit *Anthus spinoletta*, alpine accentor *Prunella collaris*, and white‐winged snowfinch *Montifringilla nivalis*; hereafter, snowfinch) whose distribution encompasses the entire Alpine arc and spans the seven European Alpine countries. We relied on species distribution models (SDMs), based on a citizen‐science bird occurrence dataset for the Alpine region of unprecedented size, to obtain a spatially explicit evaluation of the potential distribution of the target species under a range of current and future climatic conditions. We built SDMs using five alternative algorithms and evaluated them according to multiple aspects of their effectiveness. We focused not only on their statistical accuracy and generalizability across different spatial partitions of the study area (Roberts et al., 2017), but also on their ecological realism (Guevara & León‐Paniagua, 2019) and their ability to predict distribution over distant areas (Fourcade et al., 2018). Thus, we explicitly tested their extrapolation potential and, indirectly, the consistency of species‐environment associations across different mountain systems.

**FIGURE 1:**
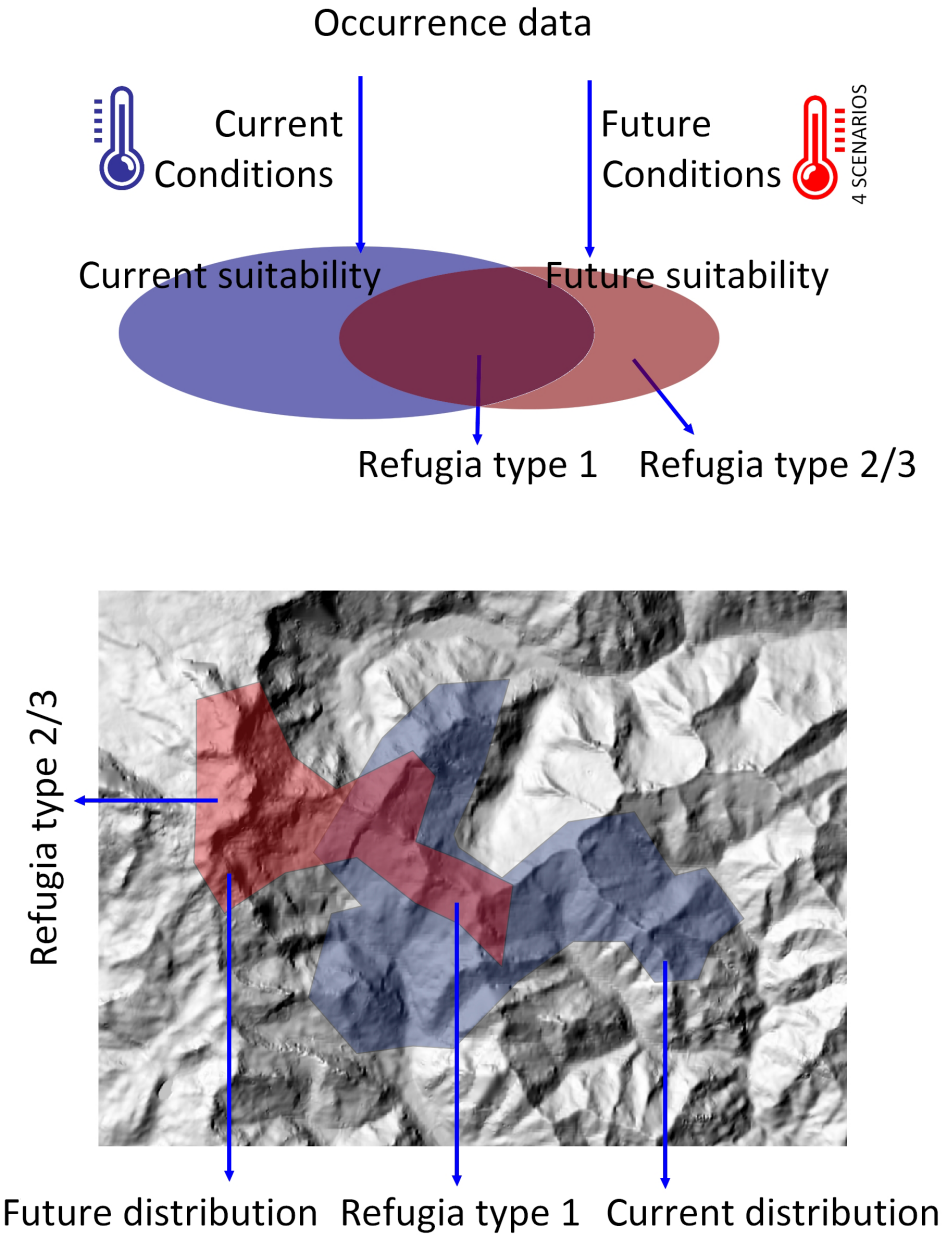
Graphical representation of the approach adopted to identify climate refugia. Type 1 refugia represent in‐situ refugia, suitable now and in the future, whereas type 2/3 are ex‐situ refugia, suitable only under all or most, respectively, possible future climates

We aimed to identify those areas in the Alps that maximize the long‐term persistence of the target species in a changing climate, i.e., those areas overlapping with in‐situ or ex‐situ climate refugia, respectively (Brambilla et al., 2017; Morelli et al., 2020; Figure 1). This would provide crucial, essential knowledge for promoting effective transnational biodiversity conservation under different climate change conditions. We thus overlaid the most relevant refugia (type 1) with the current network of protected areas (PAs) to assess their adequacy in biodiversity conservation under predicted climatic changes. In the Anthropocene, PAs are going to play a crucial role in preserving biodiversity and in averting climate‐related extinctions (Cumming, 2016; Gray et al., 2016; Lehikoinen et al., 2021; Thomas & Gillingham, 2015). However, their static locations and extents could hamper their potential to adequately preserve many species undergoing dramatic and abrupt distributional changes as a response to rapidly changing climate conditions (Hannah et al., 2007; Regos et al., 2016). This assessment and gap analysis was therefore intended to identify the strengths and weaknesses of the Alpine PA system for the conservation of high‐elevation taxa in a changing climate; identifying and promoting effective preservation of climate refugia is indeed a key strategy for conservation under a changing climate (Game et al., 2011). High coverage of climate refugia by the current PA system would suggest that existing PAs constitute a network of sites likely to be efficient for the target species and their associated habitats and communities even in a warmer future. Conversely, low coverage of refugia would reveal the likely inadequacy of current PA network in the face of climate change.

## 2 METHODS

### 2.1 Study area and selection of model species

**FIGURE 2:**
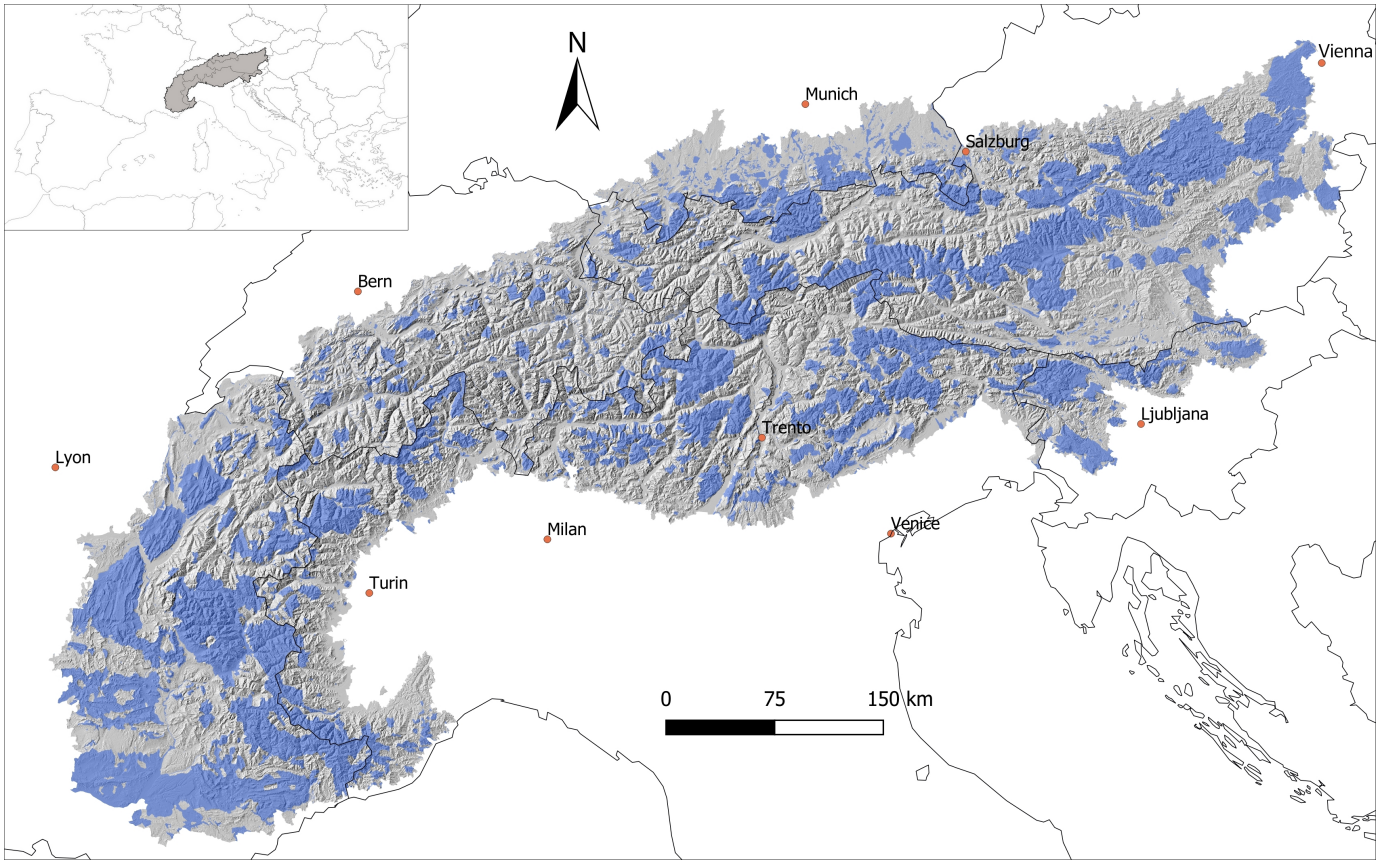
Study area showing the Alpine relief (hillshade based on the digital elevation model used for modeling, see Section 2 for details) and some main cities (boundaries: Alpine Convention area; shapefile produced by the Permanent Secretariat of the Alpine Convention and available on https://www.atlas.alpconv.org/). The top left inset shows the position of the study area within Southern Europe; blue polygons show the distribution of protected areas (all types of protected areas, covering 33.4% of the study area; extracted from World Database on Protected Areas, see Section 2 for details) within the Alps

This study encompassed all of the European Alps, identified as the main area included within the Alpine Convention (Figure 2), across seven countries (~190,000 km^2^). The Alps occupy a central position on the European continent, representing one of its most prominent features, and are among the most densely populated mountain regions in the world. Global warming in the Alps has been particularly evident and is expected to continue at a rapid pace (Gobiet et al., 2014), threatening alpine habitats (Malfasi & Cannone, 2020; Schwager & Berg, 2019). Many high‐elevation species are expected to contract their range toward higher elevations in the Alpine region, and notable shifts or contractions have already been reported (Furrer et al., 2016; Pernollet et al., 2015; Scridel et al., 2017).

Our target species are mountain specialists and/or cold‐adapted species that commonly breed across the Alps (and in at least one other mountain region of Central or Southern Europe) and have been reported to be already or potentially affected by climate change in the Alpine region (Brambilla et al., 2016, 2017, 2018; Chamberlain et al., 2013; Furrer et al., 2016; Imperio et al., 2013; Revermann et al., 2012). They are thus highly suitable candidates for assessing potential impacts of climate modification and identifying climate refugia.

### 2.2 Data collection and preparation

Data were gathered from web portals (from west to east: www.faune‐france.org, www.ornitho.it, www.ornitho.ch, www.ornitho.de, www.ornitho.at, DOPPS database) collecting bird observations based on a citizen science approach (see, e.g., Knaus et al., 2018; see also the details in the Acknowledgements).

We collated 127,309 occurrence records of different spatial accuracy, from different time periods (of the year, and from different years), and associated with different levels of breeding evidence. We retained only data collected from the year 2000 onwards, as older records often proved to be less accurate. We removed all records with non‐breeding or undefined breeding status, as well as those with unknown or low accuracy (>1 km), and those outside the breeding period of each species (1 May–31 July for rock ptarmigan, 15 May–31 July for water pipit and alpine accentor; and 1 June–31 July for snowfinch, given that many records even in the second half of May were related to sites hardly suitable for the species, likely because they involved individuals not yet engaged in reproduction). Further data in April and August for rock ptarmigan, early May and August for water pipit and alpine accentor, and the first 15 days of August for snowfinch were, however, included when associated with probable or certain breeding and located in sites potentially suitable for breeding, based on expert evaluation of aerial orthophotographs (to avoid individuals dispersing or crossing clearly unsuitable landscapes). Then, we proceeded with a final, visual inspection of all records against aerial orthophotographs and removed a further few data likely erroneously georeferenced or with erroneous species names (e.g., occurrence at the bottom of the valleys, in woodlands or urban habitats located at >1 km from potentially suitable open habitats). This resulted in 96,861 (rock ptarmigan: 8324; water pipit: 66,731; alpine accentor: 14,483; snowfinch: 7323) spatially accurate and reliable records used for SDMs.

### 2.3 Environmental variables and conditions used for modeling current and future distribution

We considered three categories of environmental variables representing the main factors potentially affecting species distribution: land‐use/land‐cover, topography, and climate. For land‐use/land‐cover, we used the CORINE land cover inventory (European Environment Agency, 2016), which provides the finest resolution land‐use data available for the entire study region (Table S1). Topographic predictors were derived from a 25‐m resolution digital elevation model (DEM; EU‐DEM v1.0, publicly provided by the European Environment Agency). As climatic predictors, we selected mean annual 2‐m air temperature, annual range in 2‐m air temperature, annual precipitation sum, and precipitation seasonality, which have been previously shown to strongly correlate with species distributions (Thuiller et al., 2019). Using monthly values from CHELSA V2.1 (Karger et al., 2017, 2021), mean annual 2‐m air temperature was calculated separately for each year, and each occurrence record was attributed the value for the year of collection of that specific record. For the background points (see below), we considered the annual mean temperature for the period 2000–2019. The other predictors were derived from long‐term values provided by CHELSA V2.1 for the period 1981–2010 (Karger et al., 2017, 2021).

All variables were computed for 1 × 1 km^2^ cells: land‐use/land‐cover as proportional cover within the cell, topographic, and climatic predictors as average values across the cell. All climatic predictors have an original resolution of 30 arc sec (corresponding to <1 km at the latitude of the Alps); they were resampled to the resolution of this study (1 × 1 km, coordinate reference system EPSG 3035) using bilinear interpolation. We excluded a priori all land‐cover variables with negligible cover, and pooled some other variables into single categories (urban and inland waters). The variables obtained were not correlated among each other (*r* < |.7|; cf. Grimmett et al., 2020). All environmental variables used for SDMs are reported in Table S1.

To represent medium‐term future climate, we used downscaled CMIP6 (Coupled Model Intercomparison Project Phase 6) data for the period 2041–2070, provided by CHELSA database version 2.1 (Karger et al., 2017, 2021). We used four climate models (two ‘warmer’ and two ‘colder’ models) provided by the Intersectoral Impact model Intercomparison Project (ISIMIP; Warszawski et al., 2014), that have been subject to a trend‐preserving bias correction (Lange, 2019; GFDL‐ESM4, UKESM1‐0‐LL, IPSL‐CM6A‐LR and MRI‐ESM2‐0). The ISIMIP data has been especially tailored for applications in climate change impact studies. The selection of climate models in ISIMIP follows that of ISIMIP3b_BA, where models are selected according to process representation, structural independence, climate sensitivity, and performance in the historical period (Lange, 2019). We selected a ‘worst case’ scenario (SSP585; Eyring et al., 2016) in order to evaluate the largest potential changes in species’ distribution: climate refugia working for pessimistic scenarios are likely to be effective also under less extreme conditions, especially for what concerns the overwhelmingly important ‘resistant units’. Hereafter, when we mention future (climate) conditions, we refer to the four alternative possible conditions as described by these climate models under SSP585.

### 2.4 Species distribution modeling

All analyses were carried out in R (R Development Core Team, 2020), using the packages raster (Hijmans, 2020), ENMeval (Muscarella et al., 2014), SDMtune (Vignali et al., 2020). All maps were produced with QGIS software (version 3.18). Data used for modeling species distribution (occurrence, background and environmental variables) are available in Brambilla (2022a).

We modeled the potential distribution of the target species as a function of climate, topography, and land cover, based on the 1 × 1 km grid superimposed over the study area. While the several available SDM methods all combine spatially explicit information on species occurrences with spatially explicit descriptors of climate and environment, and from such a combination infer relationships that allow predicting the distribution of a species over space and/or time, the choice of the specific modeling approach(es) may affect the modeling outcomes. For this reason, selecting a single SDM algorithm leaves a major degree of uncertainty when projecting future species distributions (Thuiller et al., 2019). We therefore used an ensemble of different SDM algorithms: (1) a maximum entropy approach, as implemented in Maxent (Phillips et al., 2006), a method that generally outperforms other approaches when nonstandardized data are used and is particularly suited for presence‐only data (Elith et al., 2011; Grimmett et al., 2020; Merow et al., 2013), as in our case; (2) boosted regression trees (BRT), an ‘ensemble method’ frequently adopted in ecological studies (Elith et al., 2008); (3) Artificial Neural Network (ANN; Lek & Guégan, 1999), a method gaining momentum in distribution modeling (Lin et al., 2021); and (4) Random Forest (RF), another machine learning method (Breiman, 2001) often reported to perform very well with distribution data, even for rare species (Mi et al., 2017), and recently adopted for modeling alpine species distribution (de Gabriel Hernando et al., 2021).

For presence‐background or presence‐pseudoabsence methods, an adequate placement of background/pseudoabsence points is crucial (Fourcade et al., 2014; Hertzog et al., 2014): they need to mirror the environmental conditions sampled with data collection, to avoid the inclusion into the background of conditions where the target species was not found simply because such conditions were not surveyed. In our study, we did not have any information about the sampling effort; therefore, we restricted the background to the areas containing occurrence records (cf. Brambilla, Scridel, et al., 2020) by creating a 2 km‐buffer around the occurrence records, within which we randomly placed 50,000 background points. This way, we could be reasonably certain that background points were only placed in actually sampled areas, or very close to them. To make models as robust as possible, we partitioned the occurrence data of each species into four spatially independent subsets using the checkerboard 2 method (aggregation factors: 4 and 2) implemented in the R package ENMeval (Muscarella et al., 2014). For each species, we thus used the subset containing data from three partitions to train the model (training dataset), and the fourth partition to test it on spatially independent data (testing dataset). This procedure led to the following sample sizes of occupied 1 × 1 km^2^ (training–testing): rock ptarmigan: 2818–1172, water pipit: 9282–3319, alpine accentor: 4348–1635, snowfinch: 2106–795.

ANN, BRT, and RF models were fitted in R using the package SDMtune (Vignali et al., 2020). For each species and for all algorithms, we first fit a full model with default parameters (using the training dataset). Then, we optimized the model according to the respective hyperparameters, with the command ‘optimizeModel’. The parameters involved were: for ANN, size (10–20), decay (0.01, 0.05, 0.1, 0.2, 0.3, 0.4, 0.5), and maximum iteration (50, 100, 300, 500); for BRT, number of trees (steps of 20 between 40 and 1020), interaction depth (1–4), and shrinkage (steps of 0.01 between 0.05 and 0.1); for RF, number of trees (steps of 20 between 420 and 1000, number of variables randomly sampled (mtry; 2–5), and node size (1–10). Optimization was based on TSS (True Skill Statistic; Allouche et al., 2006) values on the test dataset. Successively, we computed permutation importance for each variable and, by removing sequentially all predictors with a permutation importance lower than 1%, obtained a reduced model, based on a jackknife approach and TSS on the test dataset. Finally, we optimized the resulting model, using the same procedure described at the second step.

MaxEnt models were built in R using an ad hoc procedure. To reduce the risk of overfitting, we only used linear and quadratic features. We then built models balancing power and complexity (Warren & Seifert, 2011), according to the following procedure: first, we selected the regularization multiplier on the basis of the Akaike's Information Criterion, corrected for a small sample size (AICc). We then removed all variables with Lambda = 0, suggesting no noticeable effect on species occurrence. Then, with the remaining variables, we performed model tuning, which includes selecting (according to the AICc value) the regularization multiplier, the features to be used (linear and/or quadratic), the number of iterations, and the inclusion/exclusion of environmental variables (starting from the removal of the variable with the lowest value of permutation importance, i.e., the likely least important ones; Phillips et al., 2006). The AICc value was computed using an ad hoc script to calculate it only on presence and background points (Appendix S5), instead of over all raster layers, as is commonly done by all R packages currently available for developing MaxEnt models. This seemed more coherent with the need to take sampling efforts into account, and the environmental conditions actually available to the species. For all species, a regularization multiplier of 0.5 and both linear and quadratic features were eventually selected. The number of iterations varied from 240 (rock ptarmigan), to 260 and 300 (alpine accentor and water pipit, respectively) and 500 (snowfinch).

### 2.5 Model evaluation

We performed a multistep evaluation of model reliability. First, we calculated the TSS (True Skill Statistic) and AUC (area under the curve of the receiver operating characteristics) values for each species over the training and test datasets. Even if the absolute value of such statistics should be considered very carefully, as it is highly sensitive to prevalence and to the considered extent (see, e.g., criticisms for AUC expressed by Lobo et al., 2008), their use across different algorithms using the same datasets allows comparisons of different modeling strategies, whereas their use over independent data partitions allows a first evaluation of model robustness and generalizability. For MaxEnt, we also computed omission rates at selected thresholds (10th percentile on training data, and minimum training presence) for the test data, to further check for overfitting. Omission rates on test datasets were always close to the expected values, i.e., 0.1 for 10th percentile (all values between 0.09 and 0.10) and 0 for minimum training presence (Table S2).

We also performed an evaluation of the ecological meaning of the species‐habitat relationships identified by the models (Figures S1–S16), to check whether they corresponded with the current knowledge on species’ habitat requirements (Guevara et al., 2018). Despite this step being a qualitative evaluation based on expert knowledge, such an assessment of the biological reliability of the modeled species‐habitat relationships is essential to evaluate the *ecological realism* of any models (Elith & Leathwick, 2009; Merow et al., 2014), and this is likely to be crucial when models are to be projected over different areas or time spans (Fourcade et al., 2018; Guevara et al., 2018; Merow et al., 2014). We evaluated the importance of predictors included in each model according to the relative permutation importance, assessed by means of 10 permutations, and compared the relative effects with available knowledge.

As a final validation step, we evaluated the transferability of distribution models over independent, distant areas (see Appendix S2). Assessing models’ capacity when transferred to distant areas is the most robust method to evaluate the reliability of the modeled relationships and their outcomes on predicted distribution (cf. Fourcade et al., 2018). We thus projected the environmental suitability estimated by the models for the target species across Central‐Southern Europe, to include other mountain regions where the target species occur, as well as many absence areas. We then checked whether the main areas predicted to be suitable overlapped with known occurrence areas as depicted by BirdLife International shapefiles of species distribution (BirdLife International & Handbook of the Birds of the World, 2020), the new European breeding bird atlas (Keller et al., 2020), and vice versa. Given that the European atlas has a 50‐km resolution and that BirdLife distribution shapefiles are meant to be used at a coarse resolution, and are therefore sometimes not accurate for mountain species (cf. Brambilla, Resano‐Mayor, et al., 2020), we performed a qualitative assessment of the consistency between observed and predicted distribution. Models that correctly predict the occurrence patterns of a species over distant sites, such as other mountain regions or neighboring areas, could be considered as more reliable and useful for extrapolation over different contexts (including over different time periods) than models that perform poorly when projected outside the calibration area.

After comparing model performances by means of accuracy statistics and, especially, the assessment of ecological realism and projection over different areas, we selected the most reliable algorithm for future projections. Choosing the model able to best predict distribution outside the study area based on ecologically realistic species‐environment relationships likely provides us with the most robust and useful tool for predicting future distribution. We therefore opted to extrapolate predictions using a single, but well parametrized algorithm, without averaging it with other less suitable models (Hao et al., 2020; Kaky et al., 2020). MaxEnt invariably proved to be the most suitable algorithm (see Supporting Information), and hence, it was used for future predictions of species distribution. Notably, MaxEnt had been reported as one of the algorithms less prone to ‘extreme’ predictions over future scenarios (Beaumont et al., 2016), and well‐parametrized MaxEnt models, based on plausible species‐habitat relationships, have been reported to perform very well in identifying range limits (Guevara et al., 2018).

To exclude potential effects due to biases in observation locations and the climatic variables and methods adopted (Warren et al., 2021), we simulated the distribution of a virtual species occupying the same area of our target ones, randomly distributing 10,000 records across the same calibration area. These data were processed according to the same procedure adopted for the real species occurrence records, and a MaxEnt model was then developed according to the same method. Had the modeled result been based on biases in the approach rather than ecological reasons, we would have expected similar outcomes to those predicted for real species (Warren et al., 2021). However, no valid model was obtained for the virtual species: all environmental variables were left out of the model, thus indirectly confirming the non‐randomness of species‐habitat relationships and of the distribution models we obtained.

### 2.6 Assessing the potential changes in distribution caused by climate change

To simulate the future distribution of the four model species, we used the four alternative climatic conditions described above to project the selected SDMs to explore the likely impact of climate change on the target species in the Alps. A crude estimate of the degree of change predicted by different models can be obtained by comparing across all future conditions the value of average annual temperature, which is the single most relevant climatic predictor of environmental suitability for all the model species (see Section 3), as well as a main factor of climate change. Mean annual 2‐m air temperature was 5.34°C for the period 1981–2010 and 6.17°C for the period 2000–2019 over the study area, according to CHELSA V2.1 (Karger et al., 2017). The lowest rate of temperature increase in the Alps is predicted by the MRI‐ESM2‐0model (7.88°C in 2041–2070, 2.54°C more than 2000–2019) and the highest by UKESM1‐0‐LL (10.36 and 4.19°C more than the last two decades), with the other two GCMs predicting an intermediate level of change. For future projections, we adopted the future values of the bioclimatic predictors, while keeping the other variables (land‐cover and topographical ones) constant, considering the very slow habitat changes at high elevations (but see Section 4). Notably, given the predominant effect of temperature on species’ distributions, and the fact that current warm areas are still outside the climatic niche of the target species, there is virtually no risk due to extrapolation to non‐analogous climates (clamping was therefore set to “false”).

We compared the current and future extent, as well as the average elevation, of suitable areas for each species, according to each GCM, by considering as suitable cells all those with an environmental suitability higher than the 10th percentile threshold (working on the cloglog‐transformed model). Such a threshold was preferred over the others commonly adopted for the reclassification into suitable versus unsuitable areas (Liu et al., 2005, 2013, 2016), because it provided results that were the most coherent with current knowledge on the species’ actual distribution.

### 2.7 Identification of climate refugia

We defined in‐situ and ex‐situ refugia based on the outcomes of both models and projections. Climate refugia should include areas that probably will keep (in‐situ, resistant units), or attain (ex‐situ, resilient units) climate characteristics suitable for each target species. For each species, we identified three different types of climate refugia (Brambilla et al., 2017; Morelli et al., 2020): (1) type 1 sites are suitable for a species under current and all future conditions (i.e., the most important sites, suitable for a species irrespectively of the period and of the future condition, where population resistance is most likely); (2) type 2 depicts the most important sites among ex‐situ refugia–sites currently unsuitable for a species, but suitable under all future modeled conditions; and (3) type 3 identifies a broader sample of potential refugia (including refugia of type 2 as a subset)–ex‐situ refugia currently unsuitable for a species, but suitable under at least three out of four future modeled conditions. While refugia of type 1 are crucial to enhance population resistance, refugia of type 2 and 3 are key sites to allow future redistribution, promoting resilience.

Finally, we identified all the areas acting as type 1 refugia for at least three species. Those areas represent multispecies and temporally persistent refugia, and are of particular importance for the conservation of alpine species. We then overlapped those refugia with the current PA network. The latter was obtained by merging Natura 2000 sites with the European inventory of nationally designated PAs (Nationally designated areas; CDDA), updated in 2020 (https://www.eea.europa.eu/data‐and‐maps/data/nationally‐designated‐areas‐national‐cdda‐15; accessed 2 February 2021), and coincided exactly with the PAs included in the IUCN WDPA (World Database on Protected Areas; available on https://www.protectedplanet.net/en; accessed on 2 May 2021). We repeated the analyses after excluding those PAs not managed with biodiversity conservation as a main objective. To do this, we kept only the PAs falling under IUCN categories 1–4 (Dudley et al., 2013), plus some of the non‐categorized areas (Natura 2000 sites–Sites of Community Importance/Special Areas of Conservation, Special Protection Areas; national forest reserve, natural monument, national private nature reserve).

## 3 RESULTS

### 3.1 Selected distribution models

The three‐step evaluation procedure led us to select, for all four species, MaxEnt models as the best compromise between statistical accuracy, ecological realism, and consistency with the observed distribution in the extrapolation areas (Table S3). Permutation importance showed that mean annual 2 m air temperature was the most (or the second most) important predictor of environmental suitability for all species (Table 1).

**TABLE 1:** Permutation importance of environmental variables according to MaxEnt models. Standard deviation of permutation importance was invariably ≤.01 across three replicates. Variables have been grouped according to three main categories (land‐use/land‐cover, topography, climate) and ordered (top to bottom) within each category according to the average permutation importance across species

Variable	Rock ptarmigan	Water pipit	Alpine accentor	Snowfinch
Land‐use/land‐cover				
312 (coniferous forest)	2.3	21.3	33.4	33.3
321 (natural grassland)	13.4	17.2	3.5	0.8
332 (bare rocks)	16.0	3.2	6.9	1.8
333 (sparsely vegetated areas)	11.9	2.6	7.3	5.1
313 (mixed forest)	0.7	2.9	2.0	3.9
231 (pastures)	2.9	0.2	3.7	2.6
311 (broad‐leaved forest)	—	0.8	1.6	4.0
322 (moors and heathland)	3.8	0.1	—	1.4
324 (transitional woodland‐shrub)	—	0.3	0.2	2.6
335 (glaciers and perpetual snow)	—	—	—	0.6
Topography				
Slope (average slope in °)	5.3	8.9	2.4	11.8
solar_med (summer‐spring solar radiation)	0.2	0.4	0.5	0.1
Climate				
bio1 (annual mean temperature)	40.5	41.0	31.3	32.0
bio15 (precipitation seasonality)	1.1	0.6	4.0	4.7
bio7 (temperature annual range)	1.8	—	2.9	0.2
bio12 (annual precipitation)	0.0	0.6	0.3	0.3

### 3.2 Future projections of distribution models

The future projections showed a sensible reduction of suitable area predicted for the period 2041–2070 for rock ptarmigan, alpine accentor, and snowfinch, whereas for water pipit the predicted variation changed according to climatic conditions. The three most affected species may suffer more or less marked contractions of environmentally suitable range, and hence of potential distribution in all of the four GCMs, with less extreme changes according to MRI‐ESM2‐0 and GFDL‐ESM4, and the most dramatic ones according to the most extreme climate model UKESM1‐0‐LL (all species). The predicted contraction of potentially suitable area ranged between 24% and 59% for rock ptarmigan (average: −35.7%), 17%–43% for alpine accentor (average: −26.1%), and 18%–39% for snowfinch (average: −24.5%). Water pipit may undergo minor changes in the extent of suitable area (varying from −9% to +7%; average: +2.1%). Snowfinch was the species with the smallest extent of suitable habitat both now and in the future (with the single exception of a future climatic condition where rock ptarmigan is predicted to have a slightly smaller potential range). Table 2 displays a summary of the forecast changes in extent and elevation of suitable areas for each species, while Appendix S3 shows the spatial variation in the occurrence of suitable habitats for each target species. The change in potential distribution was matched by a concomitant increase in the average elevation of suitable cells for all species (Table 3). Snowfinch consistently showed the highest mean elevation of suitable areas, both currently and in the future, but also the lowest change from 1980–2010 to future conditions. For all species, the highest increase in average elevation was forecast by the most extreme climate model UKESM1_0_LL (between c. 352 and 457 m), and the lowest by MRI_ESM1_0 (between c. 190 and 237 m), consistently with the predicted distribution changes.

**TABLE 2:** Extent of suitable habitats (km^2^) from current to alternative future conditions (for all climatic models: SSP 585; see text), considering as suitable all sites with environmental suitability higher than the 10th percentile threshold, and the extent of different types of refugia (type 1: in‐situ refugia; types 2 and 3: ex‐situ refugia)

Species	1981–2010	GFDL_ESM4	IPSL_CM6A_LR	MRI_ESM2_0	UKESM1_0_LL	Refugia 1	Refugia 2	Refugia3
Rock ptarmigan	38,024	28,721	24,705	28,867	15,455	13,170	1514	1807
Water pipit	42,776	45,565	44,183	46,077	38,795	29,796	5882	6672
Alpine accentor	40,054	33,318	29,636	32,576	22,798	20,874	1093	1362
Snowfinch	29,783	24,410	23,181	24,200	18,157	15,837	1247	1642

**TABLE 3:** Average elevation (m asl) of suitable habitats (all sites with environmental suitability higher than the 10th percentile threshold) from current to alternative future (2041–2070) conditions

Species	Average elevation of suitable areas (m asl)				
	1980–2010	IPSL_CM6A_LR	GFDL_ESM4	MRI_ESM2_0	UKESM1_0_LL
Rock ptarmigan	2187	2493	2433	2424	2644
Water pipit	1924	2220	2180	2161	2335
Alpine accentor	2214	2466	2416	2416	2584
Snowfinch	2301	2538	2507	2491	2653

### 3.3 Climate refugia

**FIGURE 3:**
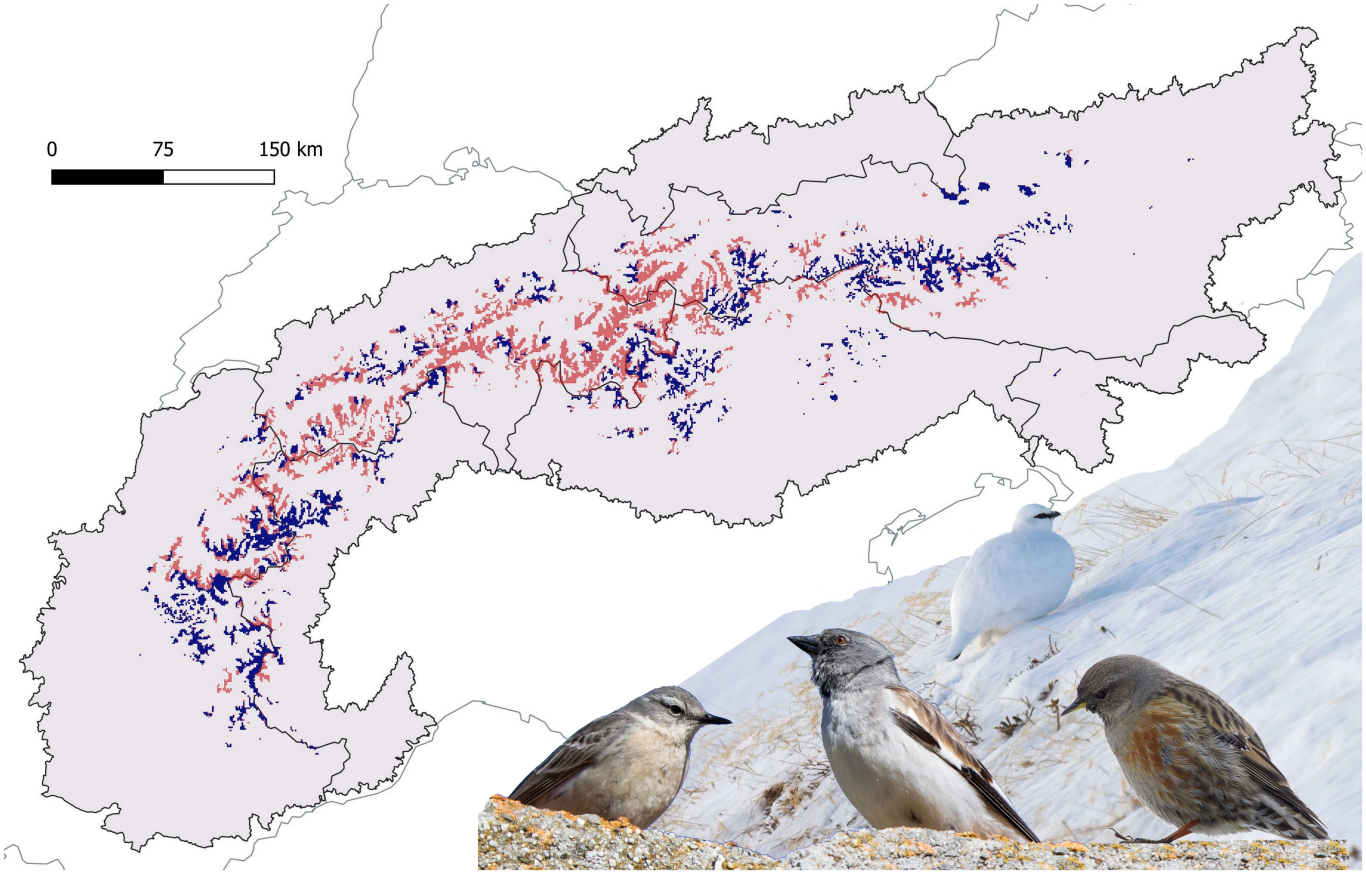
Multispecies type 1 refugia (i.e., areas suitable under current and all future conditions effective for at least three out of four target species, whatever the future climate) within (dark blue) and outside (light red) protected areas

A variable percentage of the currently suitable area for a species was likely to remain suitable in the period 2041–2070, irrespective of future climate (type 1 refugia; Table 2). The proportion of such areas varied from 35% for rock ptarmigan, to 52%–53% for alpine accentor and snowfinch, to 70% for water pipit. A total of 14,865 km^2^ was classified as potential type 1 refugia for at least three species (Figure 3). Nearly two thirds (65%) of those areas may be suitable for water pipit, and >99% for the other three species. These multispecies refugia can be regarded as priority sites for the conservation of alpine avian taxa. The type 2 refugia for at least three species were invariably close to analogous type 1 refugia (Figure S33).

Type 2 and 3 refugia had much smaller extents relative to type 1 refugia (Table 2). Obviously, refugia of type 2 (areas suitable only in the future according to all climatic conditions), which are a subset of refugia of type 3 (areas suitable only in the future according to most climatic conditions), were smaller than the latter. Spatially explicit layers for the different types of refugia for all the target species and for the multispecies refugia are available online (Brambilla, 2022b).

### 3.4 Overlap with PAs

A substantial proportion of type 1 multispecies refugia (44%, 6491 km^2^) overlapped with the current PA system (Figure 3). The per‐country values ranged from 18% for Switzerland, 52% for Austria, 54% for Italy, to 66% for France. In Slovenia and Germany, which have small areas classified as multispecies refugia (6 and 31 km^2^, respectively) and smaller extents of the Alpine chain, the coverage of refugia by the existing PAs was almost complete (100% and 94%, respectively). When considering only selected PAs (see Section 2), the percentages of refugia within PAs (39% at the Alpine scale) were lower in most countries: 13% for Switzerland, 47% for Austria, 54% for France and Italy, 80% for Germany, and 100% for Slovenia. No refugia occurred in Liechtenstein (even if one was located along its southern border).

## 4 DISCUSSION

Climate refugia are key areas allowing the persistence of species and habitats threatened by climate change (Morelli et al., 2020), because such sites are the most likely to preserve suitable ecological conditions for these habitats and species. Therefore, they represent crucial and concrete targets for conservation planning. Here, we have identified, within the framework of a spatially explicit assessment at a relatively fine scale, the location of the most likely climate refugia for four threatened Alpine bird species. These species are key representatives of high‐altitude Alpine avian communities, which are highly sensitive to ongoing climate and habitat changes (Chamberlain et al., 2013).

Our findings suggest that the fate of high‐elevation specialist species may be heavily impacted by climate change and, in particular, by the increase in temperature, which was among the most important drivers of environmental suitability in all target species. The four different GCMs were largely consistent in predicting future distributions (2041–2070), with only minor changes in the extent and elevation of suitable areas associated with the level of temperature changes forecast by each climate models. Nevertheless, a predicted contraction of suitable areas, and hence of potential range, was beyond doubt for most species, and the general patterns appeared consistent across GCMs. Such a contraction, according to the ‘worst case’ (SSP585) scenario here considered, may imply a loss of 17% to 59% of suitable range for rock ptarmigan (most marked contractions), alpine accentor and snowfinch, whereas changes may be less marked for water pipit, in accordance with previous studies on the target species (Brambilla et al., 2016, 2017; Ceresa et al., 2021; Jähnig et al., 2020). The less dramatic range contraction estimated here compared to some of the previous studies may be due to a plurality of factors, including an examination of different time periods and different GCMs, as well as the coverage of larger areas, encompassing all the northern side of the Alps and all the highest peaks. A potential additional explanation for the smaller range contractions predicted here is that we have assessed the effects of environmental predictors in the immediate vicinity of the species’ records (within 2 km), i.e., at sites that were largely suitable for the target species. This likely led to a lower importance being attributed to temperature in models compared to assessments covering broader areas, including climatically unsuitable sites. As an example of the latter process, the permutation importance of annual average temperature for the snowfinch was much higher when modeling involved larger spatial extents, including much warmer, unsuitable areas, despite the similarity in the variable effect, with a peak in suitability occurring at similar values (Brambilla, Resano‐Mayor, et al., 2020). This is consistent with the scale‐dependent relative importance of climate in driving species distribution (Brambilla et al., 2019; Pearson & Dawson, 2003). Nevertheless, a rather alarming perspective on future conditions emerged for all four species, with the partial exception of the water pipit. Such an unfavorable outlook is coherent with previous similar studies (e.g., Brambilla et al., 2016, 2017; de Gabriel Hernando et al., 2021; Furrer et al., 2016; Hotta et al., 2019; Schai‐Braun et al., 2021; Scridel et al., 2021) and with the observed and likely still ongoing range contractions or shifts of many cold‐adapted species in, e.g., Italy (Scridel et al., 2017) and Switzerland (Knaus et al., 2018), as well as throughout most of Europe (Keller et al., 2020). Together with high elevation avian taxa, other species and entire habitats sharing the same ecological space are at high risk due to climate change and its indirect effects. Species and habitats currently occurring above the treeline are particularly at risk (Malfasi & Cannone, 2020). Grasslands are becoming increasingly encroached by shrubs and trees, and often cannot shift upward due to abiotic constraints (Cannone et al., 2007). Their contraction is exacerbated by processes of land abandonment occurring in many mountain systems, implying that extensive grassland and pastures are abandoned and the remaining ones are subject to agricultural intensification (Assandri et al., 2019), synergistically impacting on these habitats with potentially strong negative effects on several bird species (Brambilla, Gustin, et al., 2020; Scridel et al., 2018), including those less likely to be dramatically impacted by direct effects of temperature change per se (e.g., water pipit; Ceresa et al., 2021; Jähnig et al., 2020).

### 4.1 Modeling limitations

A critical issue for the reliability of modeling tasks, especially when projecting distributions outside the calibration context, is the robustness and generalizability of distribution models (e.g., Brun et al., 2020). Here, we coupled the evaluation of statistical accuracy and ‘robustness’ with an explicit, albeit qualitative, assessment of ecological realism and transferability by means of extrapolation over distant areas. Even though advocated several times, a similar approach, which integrates models’ ecological and extrapolation reliability (Guevara & León‐Paniagua, 2019; Guevara et al., 2018) has seldom been adopted to date, and we are not aware of previous applications to mountain birds. Our results indicate that the statistically best performing algorithm (according to evaluation metrics, such as TSS and AUC) did not provide ecologically realistic predictions. A severe overfitting for some models other than MaxEnt was already suggested by the differences in accuracy and discriminatory statistics between training and testing datasets, and was confirmed by the inspection of the species‐habitat relationships (see also Brun et al., 2020). This also led to inadequate predictions outside the study area.

The MaxEnt models we used for forecasting future distributions were robust from a statistical point of view, but also ecologically sound, and able to correctly predict species’ occurrence over distant areas (Table S3; Figures S17–S32). Even if these strengths do not imply that the future predictions will necessarily be met (the discrepancy between future conditions indeed suggests some uncertainty), they confirm that–based on current data–the models obtained are as reliable as possible for this kind of approach. In our study, we deliberately considered future land‐use/land‐cover variables as matching the current conditions. This is clearly a simplification, because some changes in vegetation cover are already occurring because of climate change, especially around and above the tree line (Harsch et al., 2009; Malfasi & Cannone, 2020), and may be further exacerbated by land abandonment, which can lead to further shrub and tree encroachment in open habitats (e.g., Laiolo et al., 2004). Our approach is therefore conservative in estimating potential range contractions (given the climate predictions used): habitat losses caused by an upwards shift of vegetation zones, particularly above the tree line, may indeed result in a further reduction of open habitats. Nevertheless, such changes could be at least partly counteracted by management actions (see below), and large changes are unlikely to occur over many areas identified as climate refugia, because of soil and topographical constraints at several high elevation sites. Further development of this approach could therefore incorporate scenarios of future habitat change, although forecasting such changes is challenging. Similarly, models at finer spatial scales could also incorporate the local effects of topography and the related buffering against warmer temperatures that north‐facing slopes could potentially offer, especially to species with small spatial requirements (Corradini et al., in press; Feldmeier et al., 2020).

We did not consider less pessimistic scenarios than the SSP585. This choice assumed that more optimistic future conditions will simply lead to intermediate predicted distributions, between the current and the pessimistic‐future distribution we predicted here, considering the overwhelming importance of temperature for the distribution of the target species when compared to other bioclimatic predictors. We therefore also assume that the most relevant climate refugia (type 1) identified under the worst scenario adopted here will most likely also provide suitable environments under less dramatic climate changes.

Finally, our models were built using spatially accurate data collected by citizen scientists. The largely available information regarding spatial accuracy allowed us to perform an initial screening and selection of the best records. A careful inspection of data led to the removal of a few hundred records, which, despite being reported as spatially accurate, occurred in clearly unsuitable sites. Many of those data could refer to transient individuals flying over the observation site, but it is very likely that they also included some records attributed by observers to wrong locations or to the wrong species. As well as highlighting the enormous potential of citizen science data with information on spatial accuracy, our work also emphasizes the need to carefully check data accuracy before model building.

### 4.2 Implications for conservation

The availability of high‐elevation areas in the Alps may promote distributional shifts performed by species to accommodate niche tracking: the sensible shift in elevation of suitable areas predicted under future climatic conditions will allow alpine species to partially buffer the loss of suitable environment through climate change. This mountain region is thus particularly important for the conservation of high‐elevation species in Europe, where there are no other comparable extents of high‐elevation habitats. We suggest that identified climate refugia should be considered priority areas for the conservation of high‐elevation species in the Alps. Type 1 multispecies refugia include the entire future distribution of the three more threatened species and two thirds of the water pipit's distribution, considering areas suitable for the target species under all future conditions. These are indeed key areas for the conservation of the target species, but are also likely of broader relevance for high‐elevation biodiversity and habitats. In addition, analogous type 2 multispecies refugia are invariably close to type 1 multispecies refugia (Figure S33): preserving suitable conditions in type 1 multispecies refugia will also further the colonization of the new suitable sites, promoting population resilience to climate change.

Our findings pose the question of what needs be done in the near future to ensure that these crucial climate refugia conserve their role for survival of high‐elevation species. Above all, habitat degradation (Chamberlain et al., 2016), disturbance and other human‐induced alterations should be avoided in such areas, preserving them from direct anthropogenic impacts (Arlettaz et al., 2013; Patthey et al., 2008). One of the main threats in some areas of the Alps is the development of new ski facilities. Ski pistes and associated infrastructures represent a major source of impact on biodiversity and ecosystems in the Alps (Caprio et al., 2011; Negro et al., 2010; Rolando et al., 2007, 2013). Climate change is exacerbating the potential impact of downhill skiing, because the overlap of areas suitable for ski facilities and areas suitable for high‐elevation species is increasing, and several climate refugia for birds will also be attractive ‘refugia’ for ski pistes too (Brambilla et al., 2016). Other types of anthropogenic impacts that should be avoided or limited in climate refugia include new hydroelectric basins and quarries, and direct disturbance of species by recreational activities (Arlettaz et al., 2007; Chamberlain et al., 2016). The latter is particularly harmful to rock ptarmigan, a highly sensitive species, which is still a game species in different Alpine regions, despite ongoing population decline (Furrer et al., 2016) and low breeding success (Canonne et al., 2020). Finally, suitable habitats in terms of vegetation type and structure should be maintained by means of dedicated management, such as carefully planned grazing to limit the height of grassland sward at suitable values for foraging alpine birds (Brambilla et al., 2018), or for the prevention of shrub and tree encroachment (Malfasi & Cannone, 2020).

We suggest that all these conservation actions be adopted in climate refugia to limit or counteract the negative impacts of climate change on sensitive Alpine species. Their concrete implementation and application should occur at different levels, from territorial planning, to management plans of PAs of all categories in the Alpine region. In some cases, the designation of new or the expansion of existing PAs could be proposed to include refugia currently located outside the boundaries of PAs, especially in Switzerland, where the percentage of multi‐species refugia actually included in PAs is the lowest, but also in some areas in Italy, Austria and France (Figure 3). The expansion of PA networks is an even more compelling measure considering that PAs potentially buffer climate change impacts on birds (Lehikoinen et al., 2021), which is crucial to mitigate losses and avert local extinctions (P. Lehikoinen et al., 2019). Nevertheless, even in areas where multispecies refugia are largely included within PAs, it is essential that PAs are adequately and effectively managed. Weak regulations or low enforcement of protection rules can lead to habitat alteration even within PAs, undermining their potential significance in conserving refugia, and biodiversity and ecosystems in general.

For conservation‐oriented research, climate refugia are key sites for investigating the fine‐scale drivers of species occurrence and habitat use, or to begin studies on alpine bird demography. The latter is a neglected topic of potentially great relevance to understanding the ultimate impact of climate change on wild species; recent studies have just started shedding light on this relevant topic, highlighting the role of temperature and/or precipitation (e.g., Chiffard et al., 2019; Strinella et al., 2020). Investigating associations between alpine birds and habitat characteristics in relation to management regimes within climate refugia will help promoting the maintenance of crucial foraging or nesting habitats for such threatened species at the local scale (Brambilla et al., 2018).

## 5 CONFLICT OF INTEREST

The authors declare no competing financial interests.

## Supporting information

Supplementary Material

## Data Availability

Data used for modeling species distribution are available (in CSV and RDS format) as SWD files with occurrence, background points, and environmental variables in UNIMI dataverse, together with codes used for the main models (Brambilla, 2022a). Raster files for refugia (coordinate reference system: EPSG 3035; resolution: 1 km) are available in UNIMI dataverse (Brambilla, 2022b).
