# Supplementary material for: Identifying climate refugia for high‐elevation Alpine birds under current climate warming predictions
Source: Glob Chang Biol. 2022 Apr 20;28(14):4276–91. doi: 10.1111/gcb.16187 (PMC9546033; doi:10.1111/gcb.16187)

Supplementary Material for the paper

Identifying climate refugia for high-elevation Alpine birds under current climate warming predictions

Mattia Brambilla, Diego Rubolini, Ojan Appukuttan, Gianpiero Calvi, Dirk Nikolaus Karger, Primož Kmecl, Tomaž Mihelič, Thomas Sattler, Benjamin Seaman, Norbert Teufelbauer, Johannes Wahl, Claudio Celada

Appendix 1

Table S1. List of environmental predictors of different kind adopted for species distribution modelling. Urban habitats and waterbodies were left out to avoid potential biases related to number of visits by observers. Predictors were derived from CORINE (CLC; land cover), CHELSA V. 2.1 (climate) and EU-DEM v1.0 (see text for details).

**variable**	**description**
**topography**	
solar_med	average summer-spring solar radiation (global solar radiation considering shadowing reliefs, calculated over the summer period) over the cell; calculated for 21st March, 21st June and 22nd September, averaged over time and cell, in GRASS (Neteler et al., 2012)

slope	average slope (°) in the cell
**climate**	
bio1	Annual Mean Temperature – calculated ad hoc for each year and for 2000-2019
bio7	Temperature Annual Range
bio12	Annual Precipitation
bio15	Precipitation Seasonality
**land cover (CLC code and description)**	
141	Green urban areas
231	Pastures
244	Agro-forestry areas
311	Broad-leaved forest
312	Coniferous forest
313	Mixed forest
321	Natural grasslands
322	Moors and heathland
323	Sclerophyllous vegetation
324	Transitional woodland-shrub
331	Beaches, dunes, sands
332	Bare rocks
333	Sparsely vegetated areas

334	Burnt areas
335	Glaciers and perpetual snow

1.1 Models' evaluation and performance

Most of the distribution models we obtained appeared to be statistically reliable, with values of accuracy statistics being nearly equal for training and testing datasets, apart from random forest. The latter showed the highest values of AUC and TSS (being always ~1 on the training dataset), but also the larger differences between training and testing (always > 0.1 for both AUC and TSS), suggesting overfitting (Table S2). AUC and TSS showed rather similar values across the other three methods, with also limited differences between training and testing partitions (especially in MaxEnt). In MaxEnt, all omission rates displaying values extremely close to the expected ones (Table S2).

Table S2. Values of True Skill Statistic (TSS), Area Under the Curve (AUC) of the ROC plot, omission rates (OR) at minimum training presence (MTP) and 10th percentile (10th), for the target species.

	MaxEnt						Artificial Neural Network				Boosted Regression Trees				Random Forest			
**species**	**TSS train**	**TSS test**	**AUC train**	**AUC test**	**OR MTP test**	**OR 10th test**	**TSS train**	**TSS test**	**AUC train**	**AUC test**	**TSS train**	**TSS test**	**AUC train**	**AUC test**	**TSS train**	**TSS test**	**AUC train**	**AUC test**
rock ptarmigan	0.412	0.420	0.766	0.772	0.000	0.096	0.439	0.406	0.789	0.769	0.442	0.436	0.793	0.778	1.000	0.579	1.000	0.854
water pipit	0.344	0.357	0.735	0.739	0.000	0.098	0.366	0.370	0.748	0.746	0.361	0.355	0.745	0.739	1.000	0.548	1.000	0.851
alpine accentor	0.392	0.401	0.751	0.760	0.000	0.090	0.419	0.414	0.776	0.761	0.426	0.418	0.786	0.770	1.000	0.553	1.000	0.835
snowfinch	0.515	0.518	0.831	0.832	0.000	0.094	0.524	0.529	0.840	0.832	0.511	0.505	0.836	0.824	1.000	0.628	1.000	0.880

The species-habitat relationships depicted by models were in general fully consistent with current knowledge on the ecology target species (see Table S3) for MaxEnt (Figs. S1-S4), largely also for ANN and BRT (Figs. S5-S12), even if the latter two showed some signs of overfitting and a few unrealistic patterns. RF species-habitat relationships revealed more critical overfitting and lacked ecological realism in several cases (Fig. S13-16).

For all MaxEnt models, the predicted distribution showed a rather good consistency with the known one, even outside the Alps (Table S3): the distribution of environmentally suitable areas in other European areas matched fairly well the reported distribution of all target species (Figs. S17-S20). The inclusion, among potentially suitable areas, of a few mountain regions that are not currently occupied by a target species (because outside its current range) would suggest they could represent a conservative choice also in terms of extrapolation aimed at quantifying future changes in distribution. ANN and BRT models also led to a decent concordance with observed distributions, even if some occupied areas did not show any suitable patches (Figs. S21-S28). Finally, RF models led to poor matching with observed distributions especially outside the Alps (Figs. S29-S32). For rock ptarmigan, which in central and southern Europe occurs only in the Alps and the Pyrenees, MaxEnt, ANN and BRT provided consistent predictions. For all other species, MaxEnt model provided the most reliable prediction of species' distribution outside the Alps.

Therefore, our three-step evaluation procedure led us to select MaxEnt models for all the species, as the best compromise between statistical accuracy, ecological realism and consistency with the observed distribution in the extrapolation areas (Table S3).

Table S3. Summary of the results of the valuation procedure of models' accuracy, ecological realism and extrapolation ability over different areas. Abbreviations: ANN: Artificial Neural Network; BRT: Boosted Regression Trees; ME: MaxEnt; RF: Random Forest; RP: rock ptarmigan; WP: water pipit; AA: alpine accentor; WS: white-winged snowfinch; mod.: moderate. Category ranking from worst to best: low, partial, moderate, good, high.

**model**	**accuracy**				**ecological realism**				**extrapolation**			
	**RP**	**WP**	**AA**	**WS**	**RP**	**WP**	**AA**	**WS**	**RP**	**WP**	**AA**	**WS**
ANN	mod.	partial	mod.	good	high	mod.	good	partial	high	high	good	mod.
BRT	mod.	partial	mod.	good	mod.	mod.	good	mod.	good	partial	mod.	partial
ME	mod.	partial	mod.	good	high	high	high	high	high	high	high	high
RF	high	high	high	high	low	low	low	low	low	low	low	low

1.2 Species-habitat relationships depicted by different distribution models: explanation and figure captions

Those shown are the marginal response curves, obtained by means of the ‘plotResponse’ command of SDMtune (Vignali et al., 2020), graphically displaying the effect of a variable when all the other predictors are kept at the respective average value (with the option “only presence” set to false, so that the mean value of the predictors is computed over presence and background points).

Explanation and abbreviation of variable names used in the figures:

**abbreviation**	**variable name (text, Table 1)**	**short description (see text for full details)**
solar_med	solar_med	average summer-spring solar radiation
slope	slope	average slope (°) in the cell
bio1	bio1	Annual Mean Temperature
bio7	bio7	Temperature Annual Range
bio12	bio12	Annual Precipitation
bio15	bio15	Precipitation Seasonality
X141	141	Green urban areas
X231	231	Pastures
X244	244	Agro-forestry areas
X311	311	Broad-leaved forest
X312	312	Coniferous forest

X313	313	Mixed forest
X321	321	Natural grasslands
X322	322	Moors and heathland
X323	323	Sclerophyllous vegetation
X324	324	Transitional woodland-shrub
X331	331	Beaches, dunes, sands
X332	332	Bare rocks
X333	333	Sparsely vegetated areas
X334	334	Burnt areas
urban	urban	overall cover of urban areas
X335	335	Glaciers and perpetual snow

Cited reference: Vignali, S., Barras, A.G., Arlettaz, R., Braunisch, V., 2020. SDMtune: An R package to tune and evaluate species distribution models. Ecol. Evol. 10, 11488–11506. https://doi.org/10.1002/ece3.6786

Figure captions

Figure S1. Species-habitat relationships according to the MaxEnt model for rock ptarmigan.

Figure S2. Species-habitat relationships according to the MaxEnt model for water pipit.

Figure S3. Species-habitat relationships according to the MaxEnt model for alpine accentor.

Figure S4. Species-habitat relationships according to the MaxEnt model for white-winged snowfinch.

Figure S5. Species-habitat relationships according to the Artificial Neural Network model for rock ptarmigan.

Figure S6. Species-habitat relationships according to the Artificial Neural Network model for water pipit.

Figure S7. Species-habitat relationships according to the Artificial Neural Network model for alpine accentor.

Figure S8. Species-habitat relationships according to the Artificial Neural Network model for white-winged snowfinch.

Figure S9. Species-habitat relationships according to the Boosted Regression Trees model for rock ptarmigan.

Figure S10. Species-habitat relationships according to the Boosted Regression Trees model for water pipit.

Figure S11. Species-habitat relationships according to the Boosted Regression Trees model for alpine accentor.

Figure S12. Species-habitat relationships according to the Boosted Regression Trees model for white-winged snowfinch.

Figure S13. Species-habitat relationships according to the Random Forest model for rock ptarmigan.

Figure S14. Species-habitat relationships according to the Random Forest model for water pipit.

Figure S15. Species-habitat relationships according to the Random Forest model for alpine accentor.

Figure S16. Species-habitat relationships according to the Random Forest model for white-winged snowfinch.

Figure S1. Species-habitat relationships according to the MaxEnt model for rock ptarmigan.

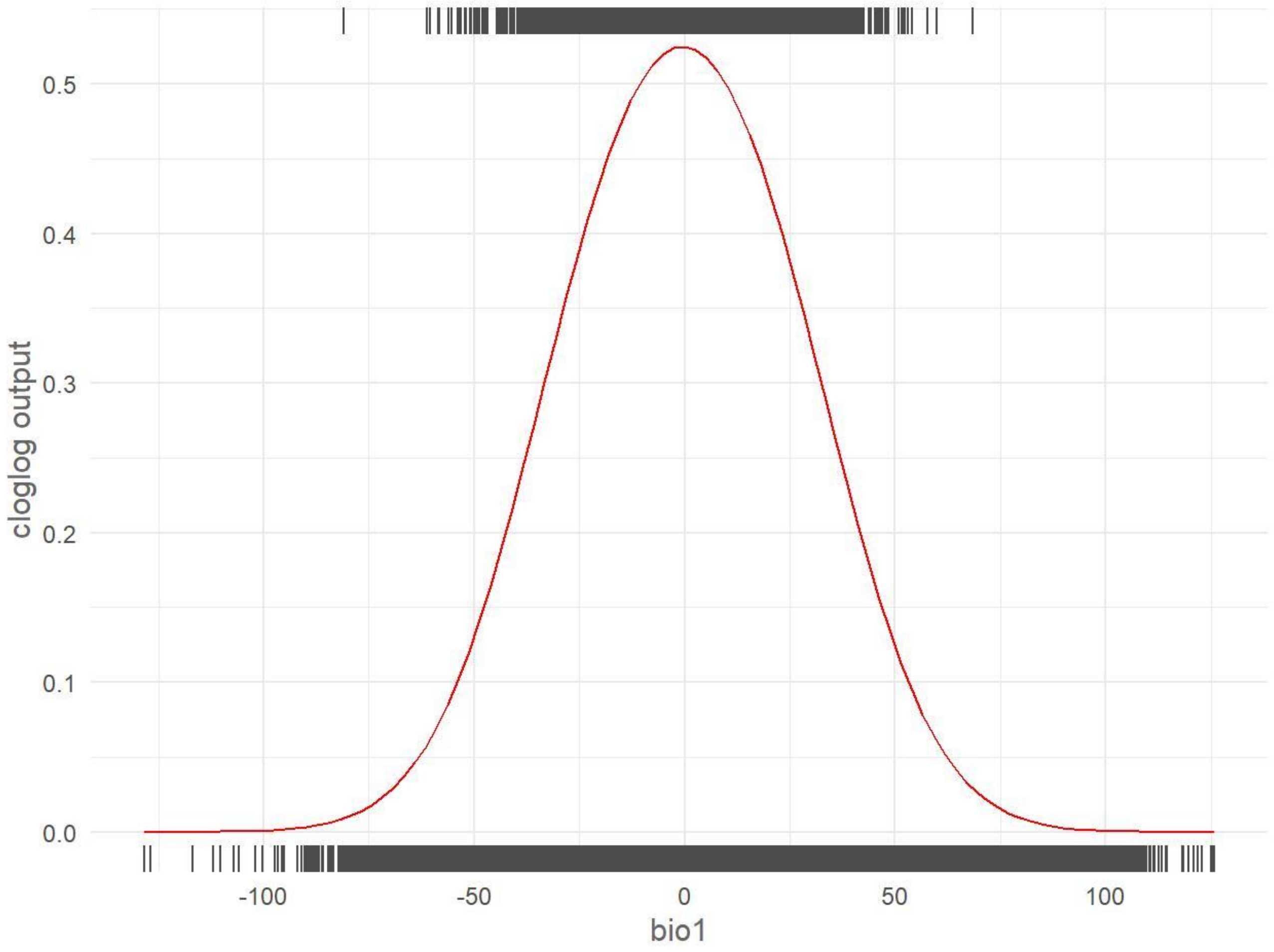

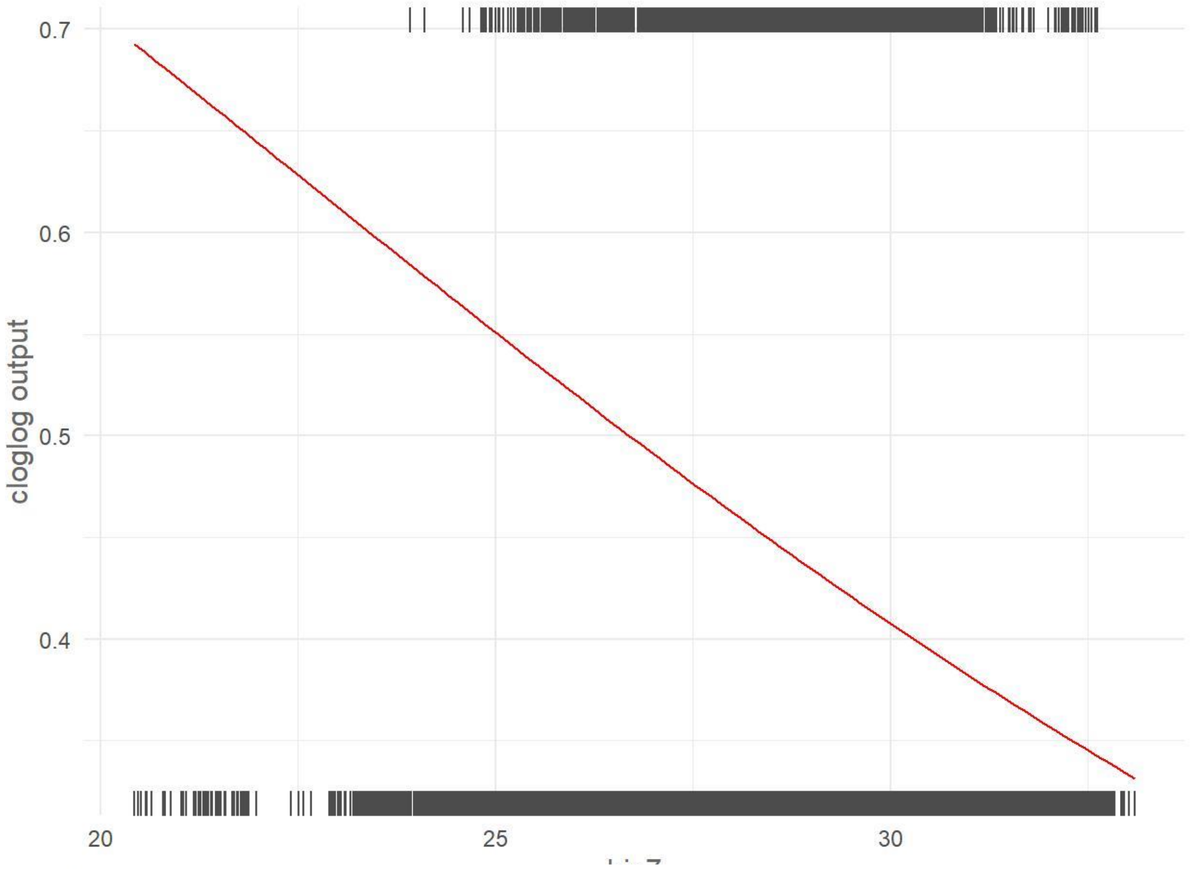
cloglog output
0.7
0.6
0.5
0.4
20
25
30

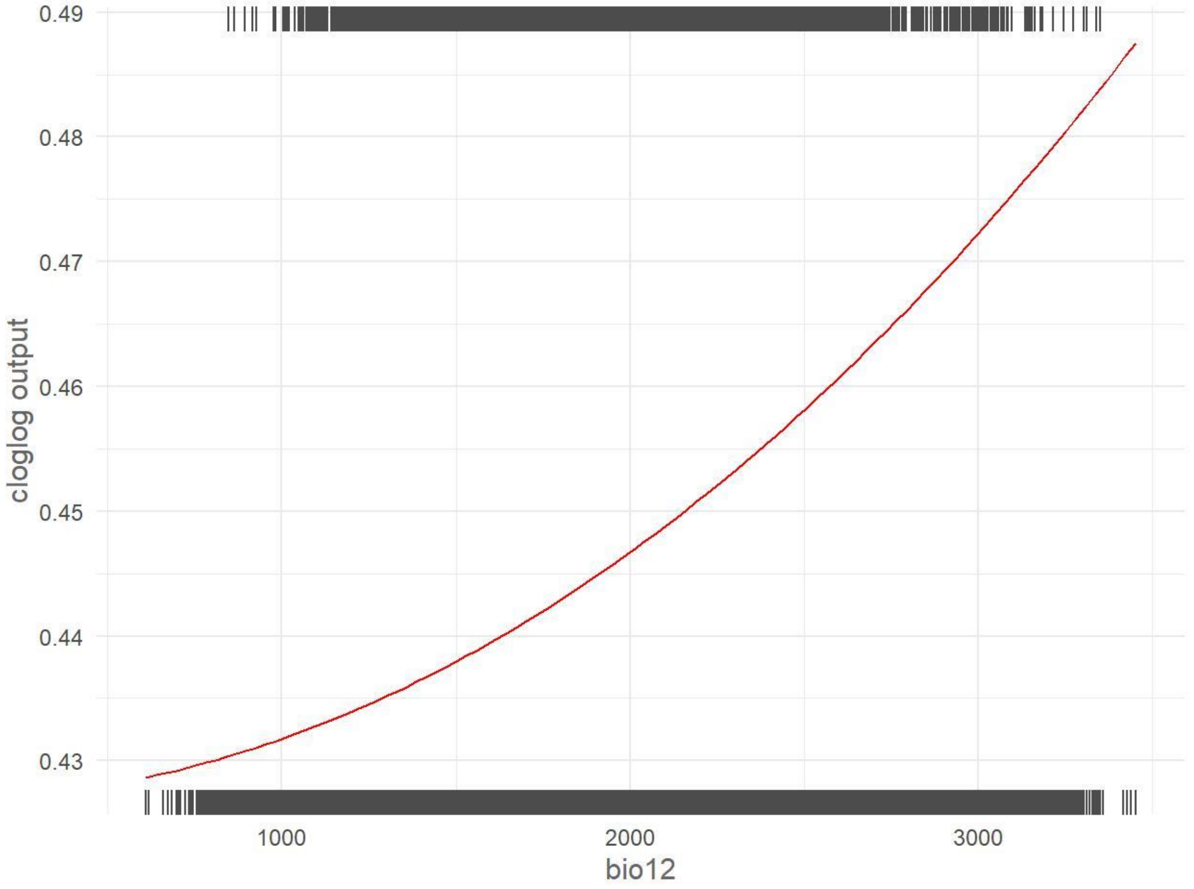
cloglog output
0.49
0.48
0.47
0.46
0.45
0.44
0.43
1000
2000
3000
bio12

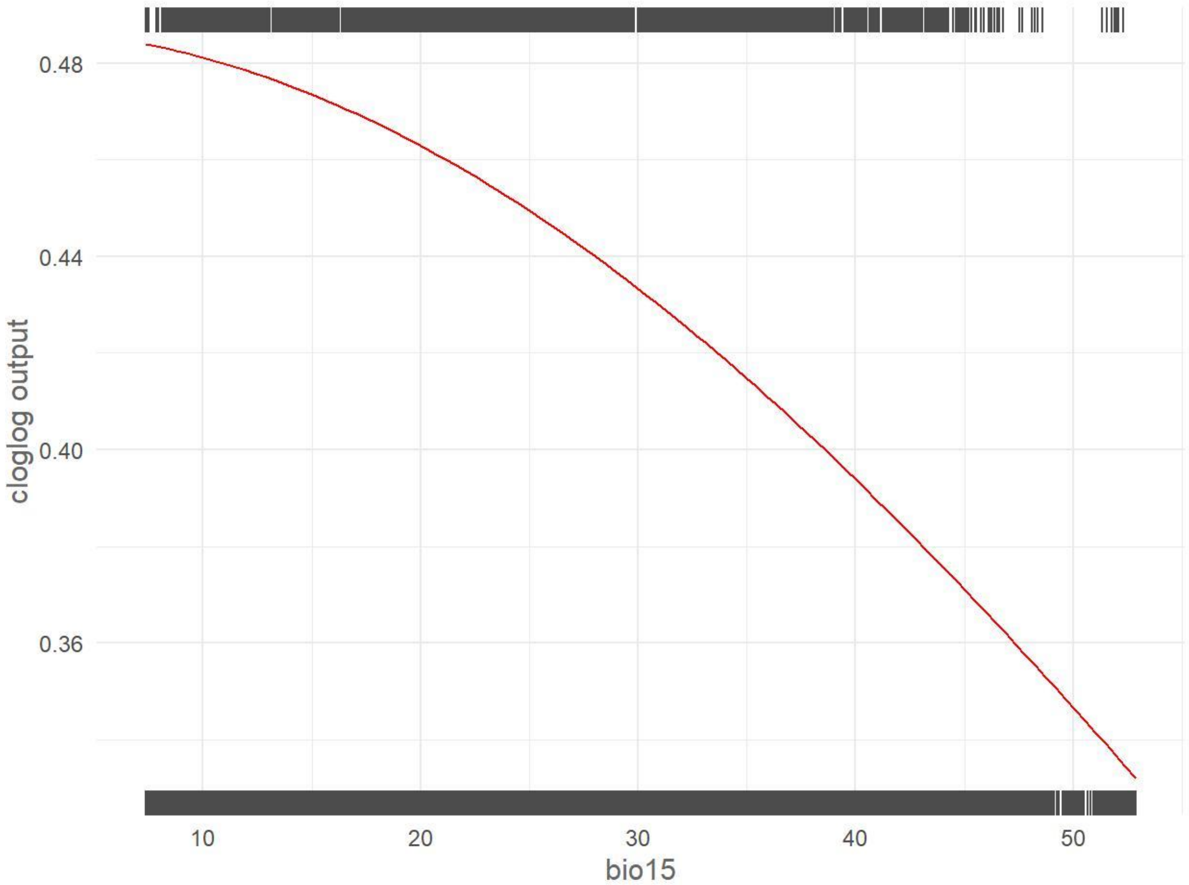

0.48
0.44
0.40
0.36
cloglog output
10
20
30
40
50
bio15

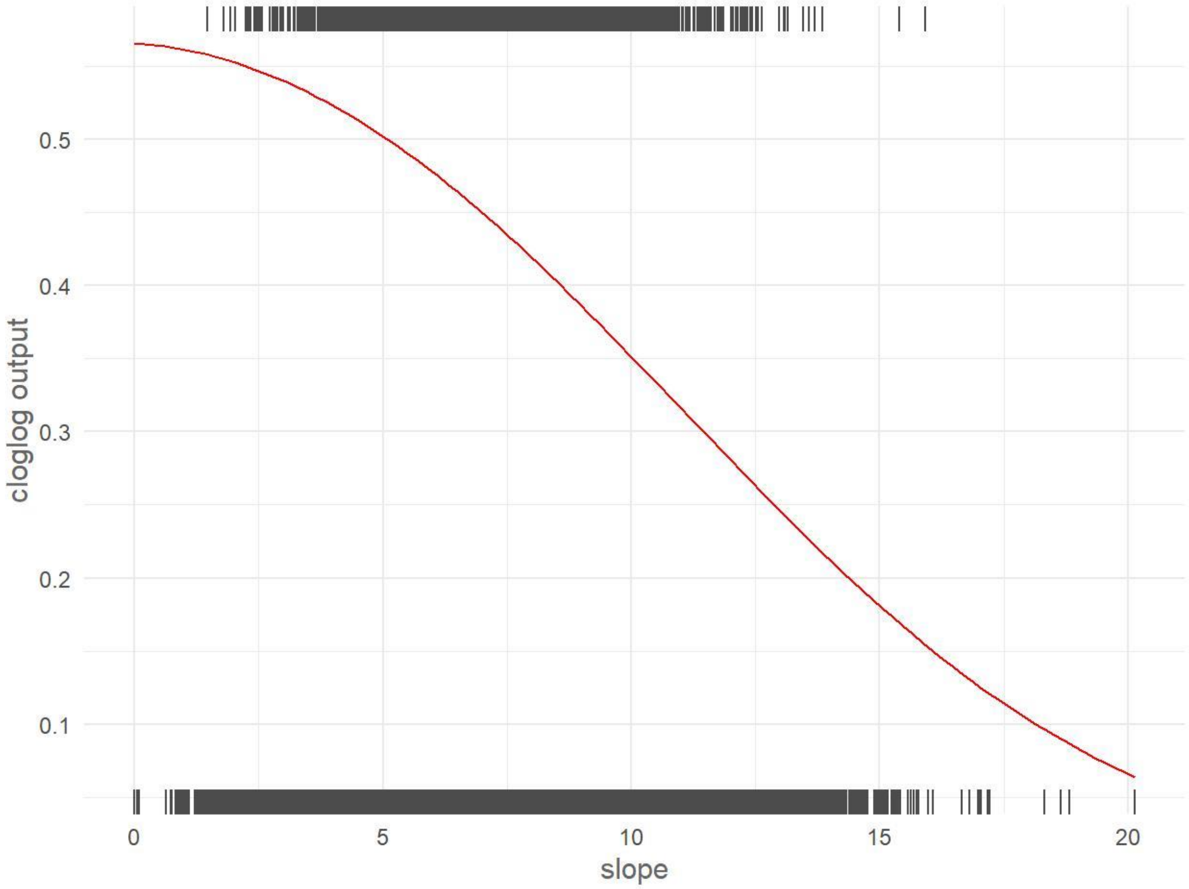

0.5
0.4
0.3
0.2
0.1
cloglog output
0
5
10
15
20
slope

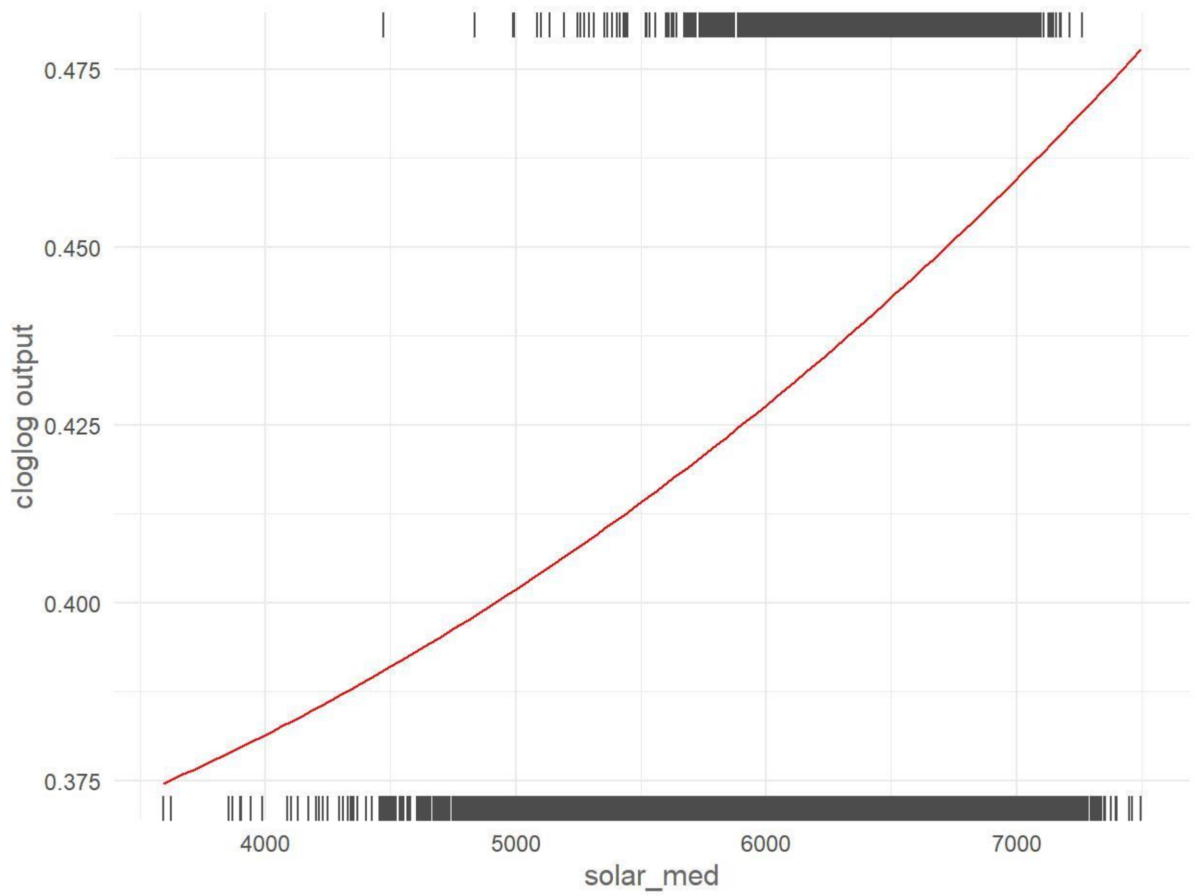

0.475
0.450
0.425
0.400
0.375
cloglog output
4000
5000
6000
7000
solar_med

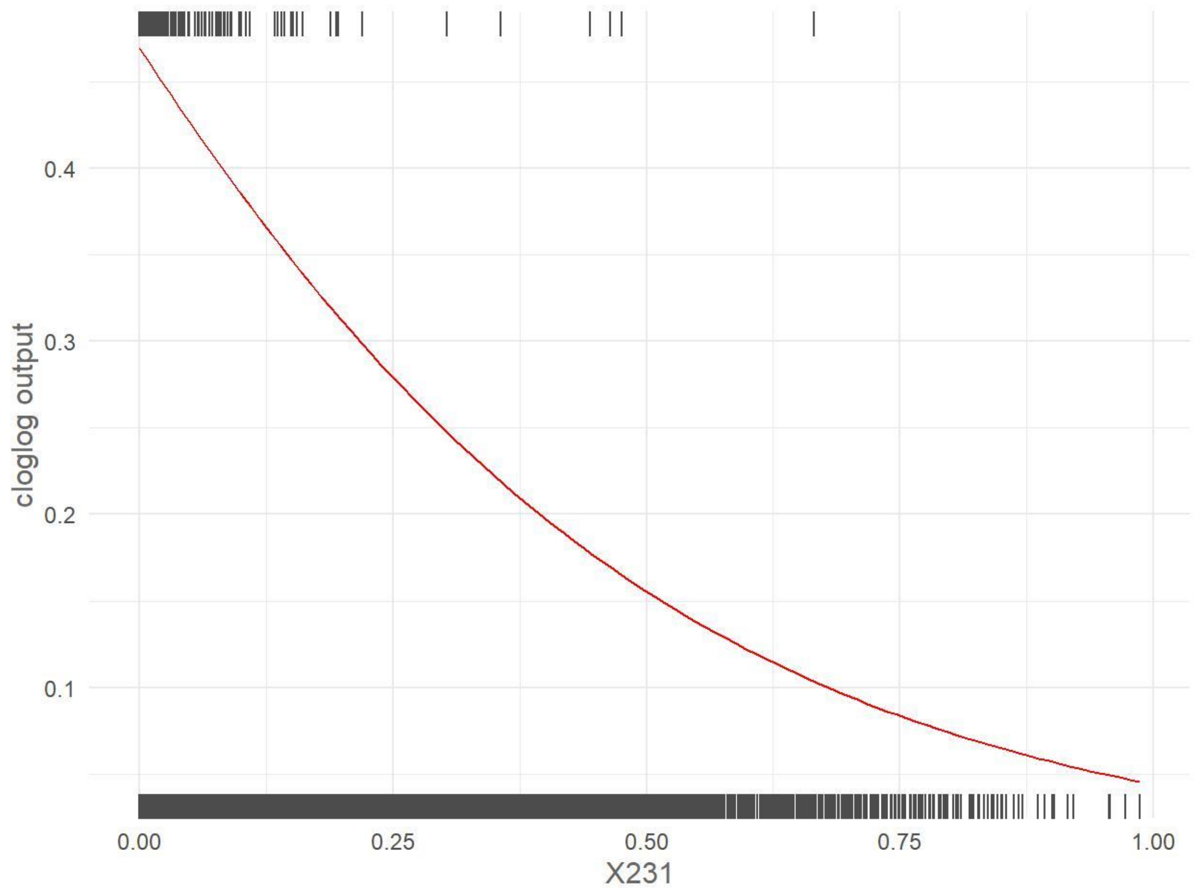

0.4
0.3
0.2
0.1
cloglog output
0.00
0.25
0.50
0.75
1.00
X231

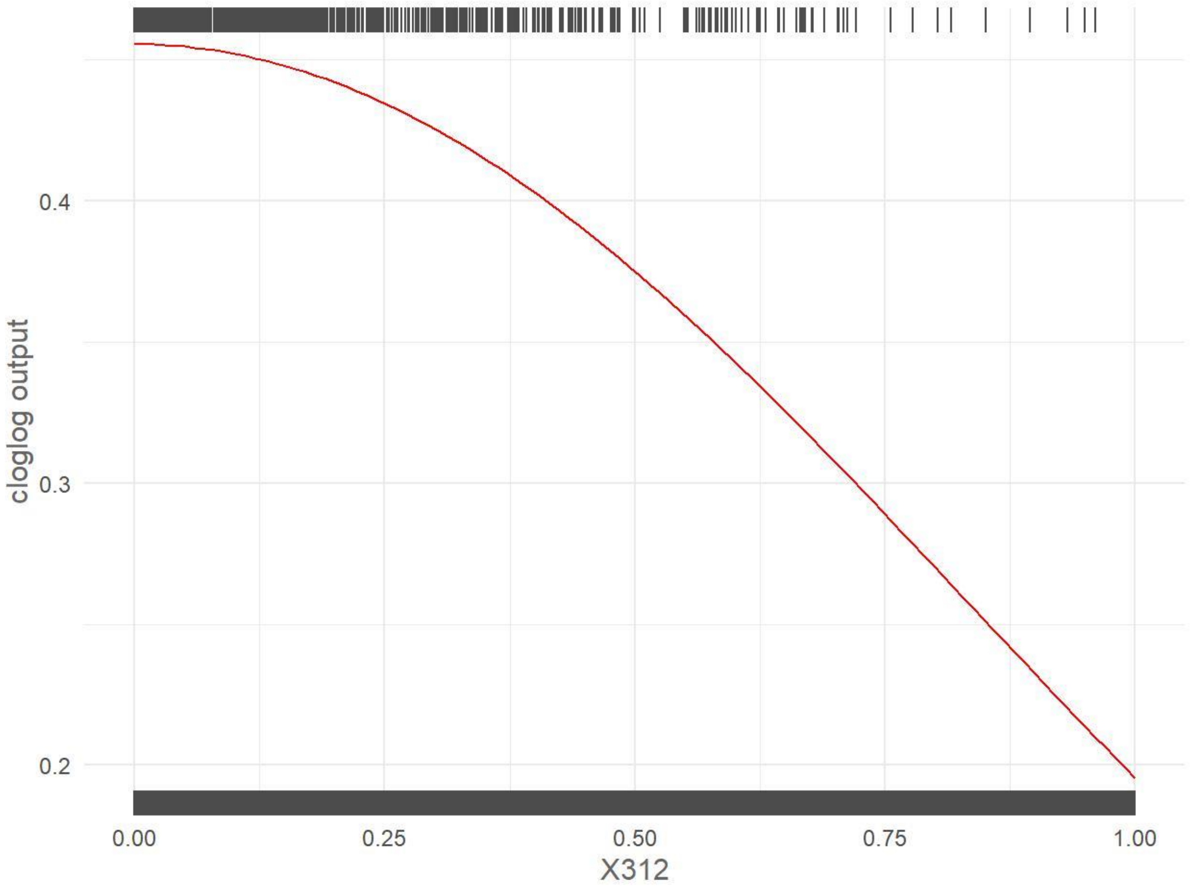

0.4
0.3
0.2
cloglog output
0.00
0.25
0.50
0.75
1.00
X312

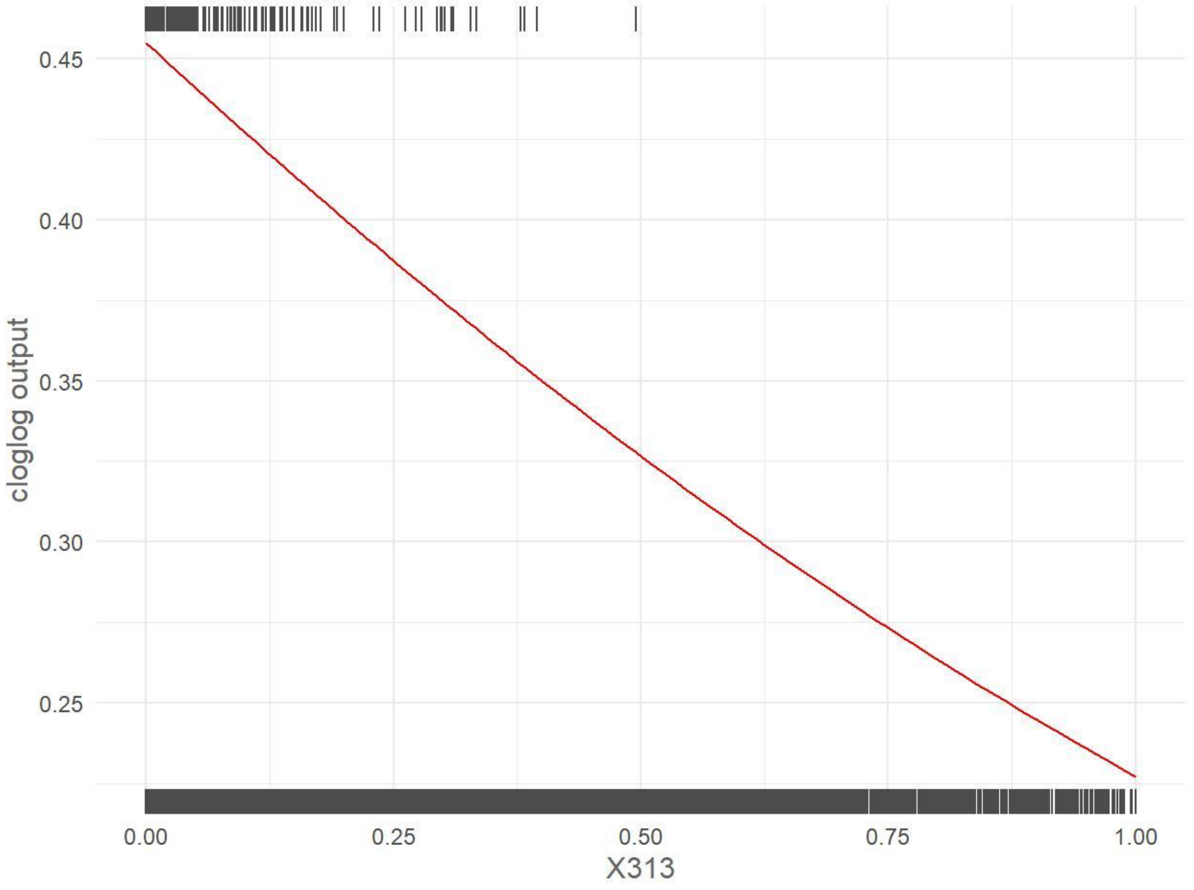

0.45
0.40
0.35
0.30
0.25
cloglog output
0.00
0.25
0.50
0.75
1.00
X313

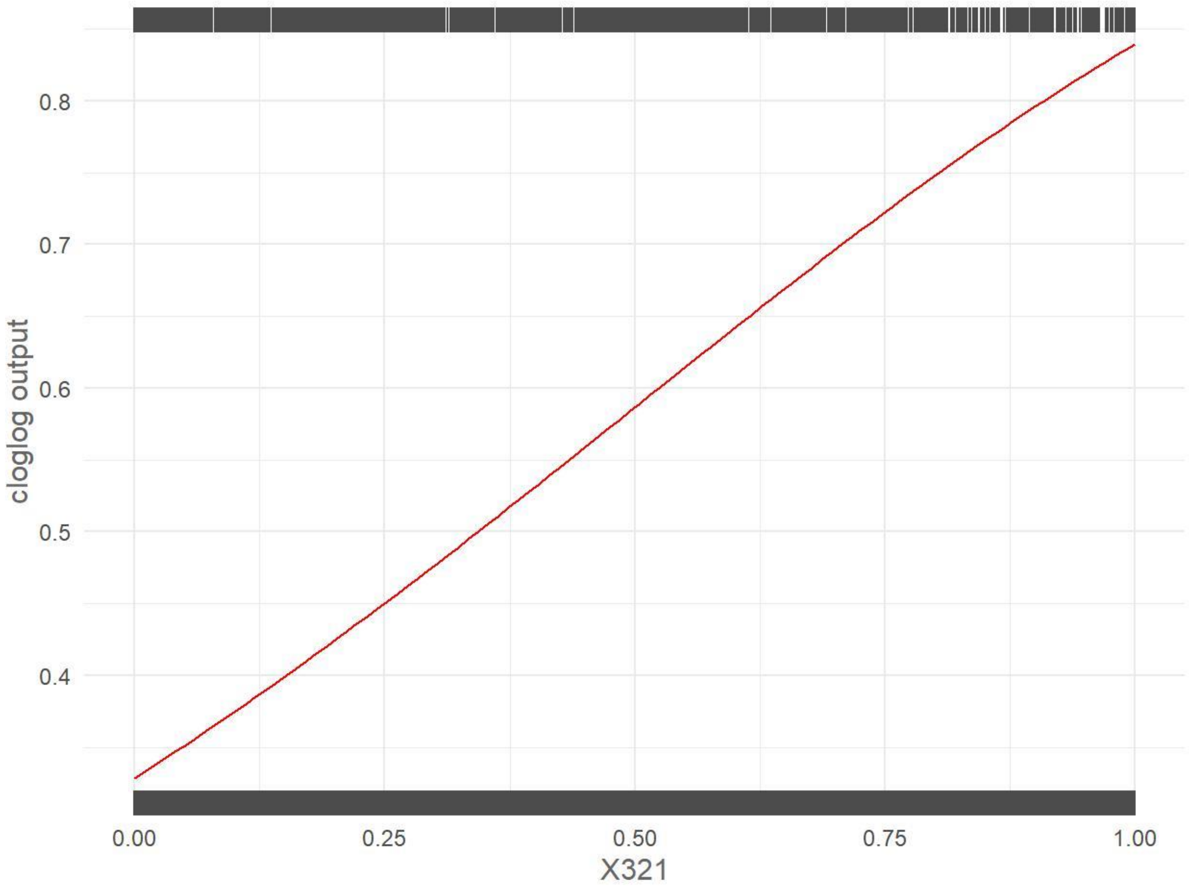

cloglog output
0.4
0.5
0.6
0.7
0.8
0.00
0.25
0.50
0.75
1.00
X321

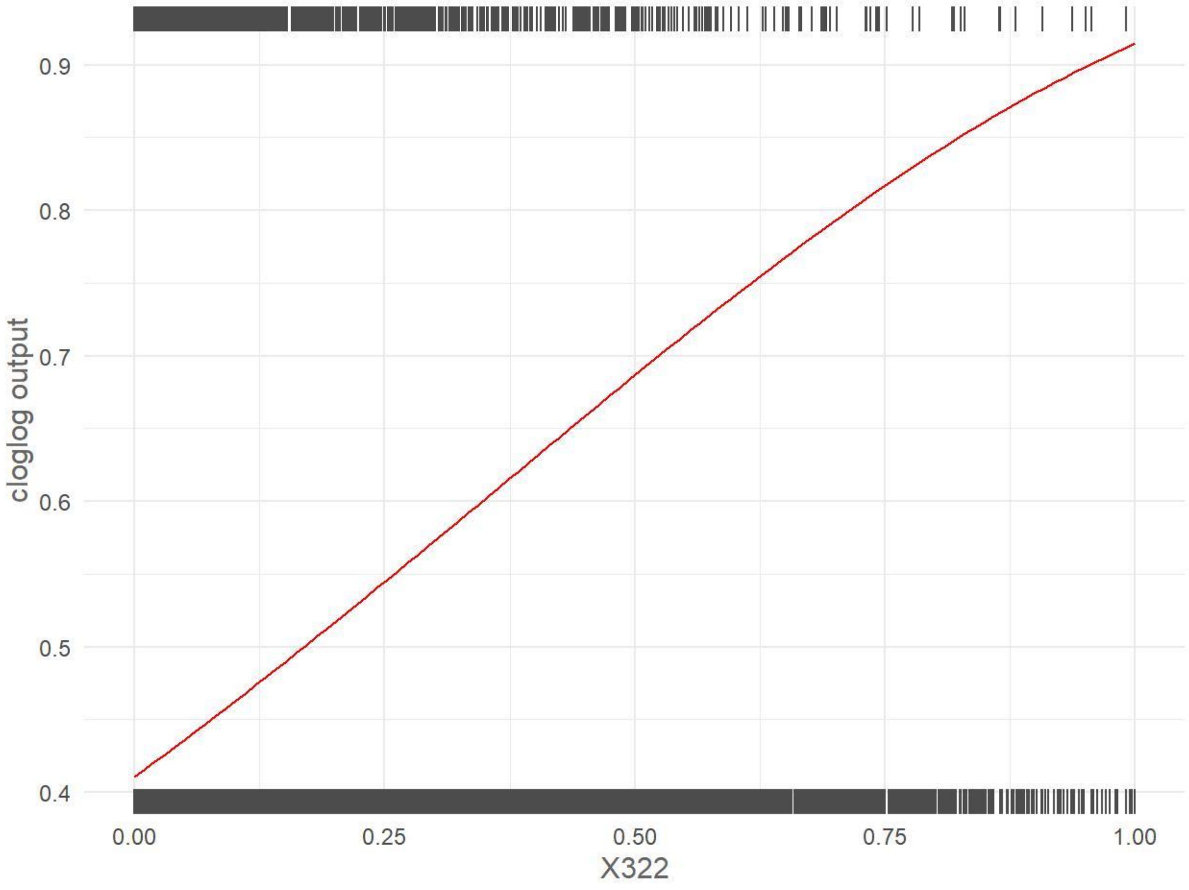

cloglog output
0.4
0.5
0.6
0.7
0.8
0.9
0.00
0.25
0.50
0.75
1.00
X322

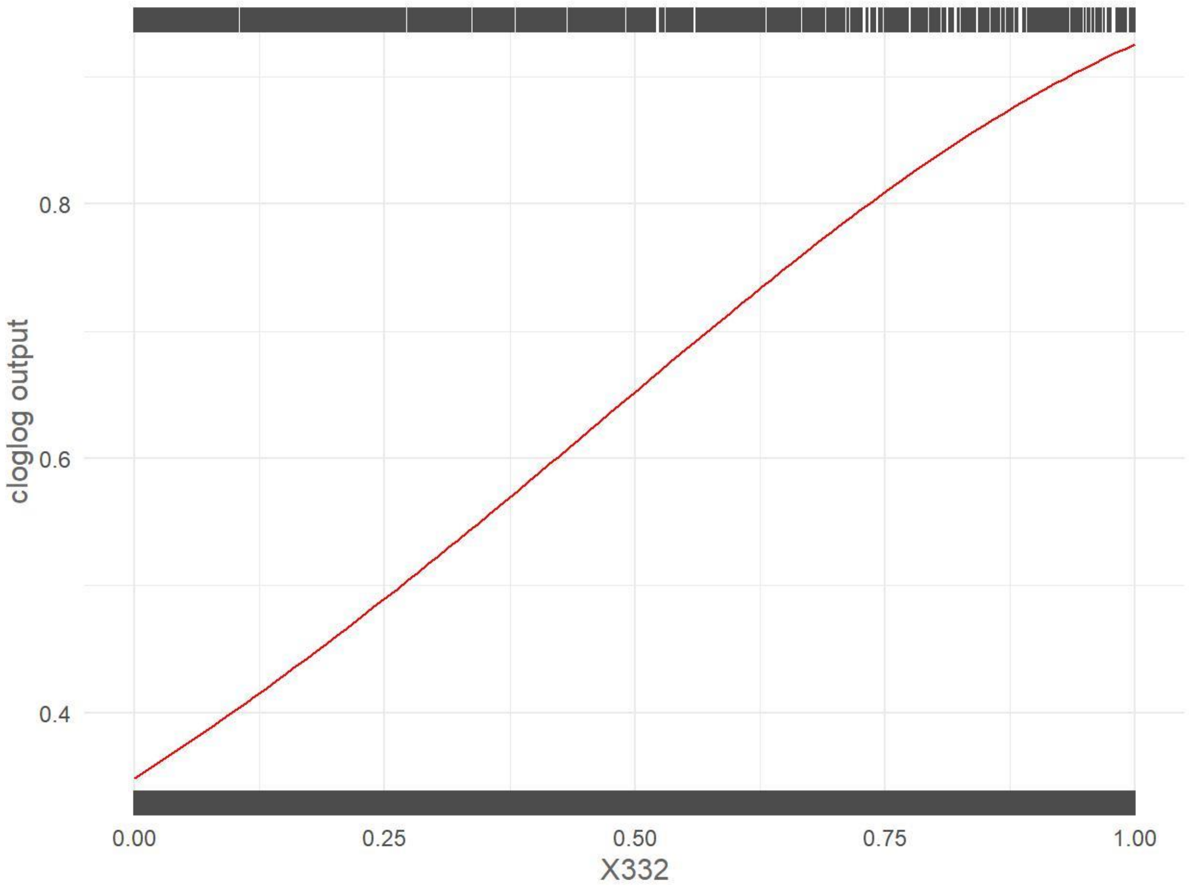
0.8
0.6
0.4
cloglog output
0.00
0.25
0.50
0.75
1.00
X332

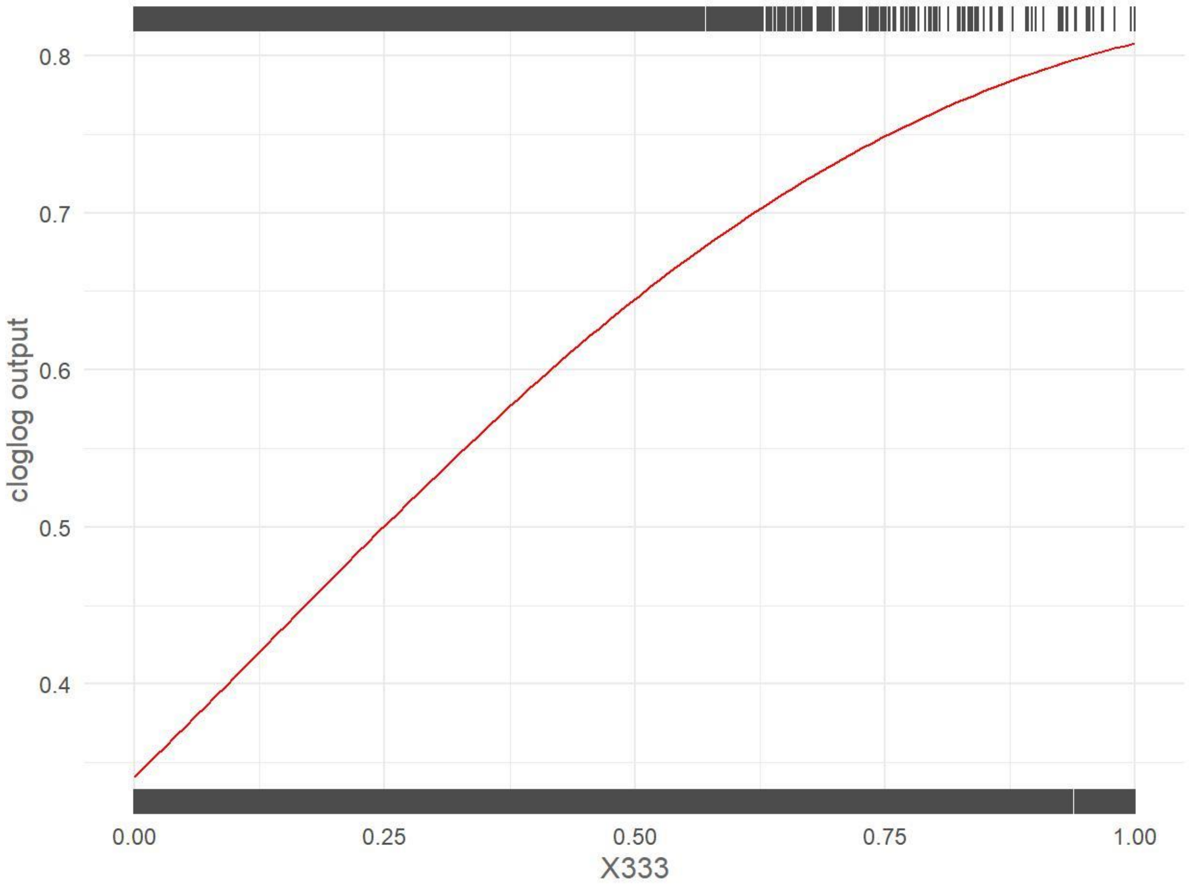
0.8
0.7
0.6
0.5
0.4
cloglog output
0.00
0.25
0.50
0.75
1.00
X333

Figure S2. Species-habitat relationships according to the MaxEnt model for water pipit.

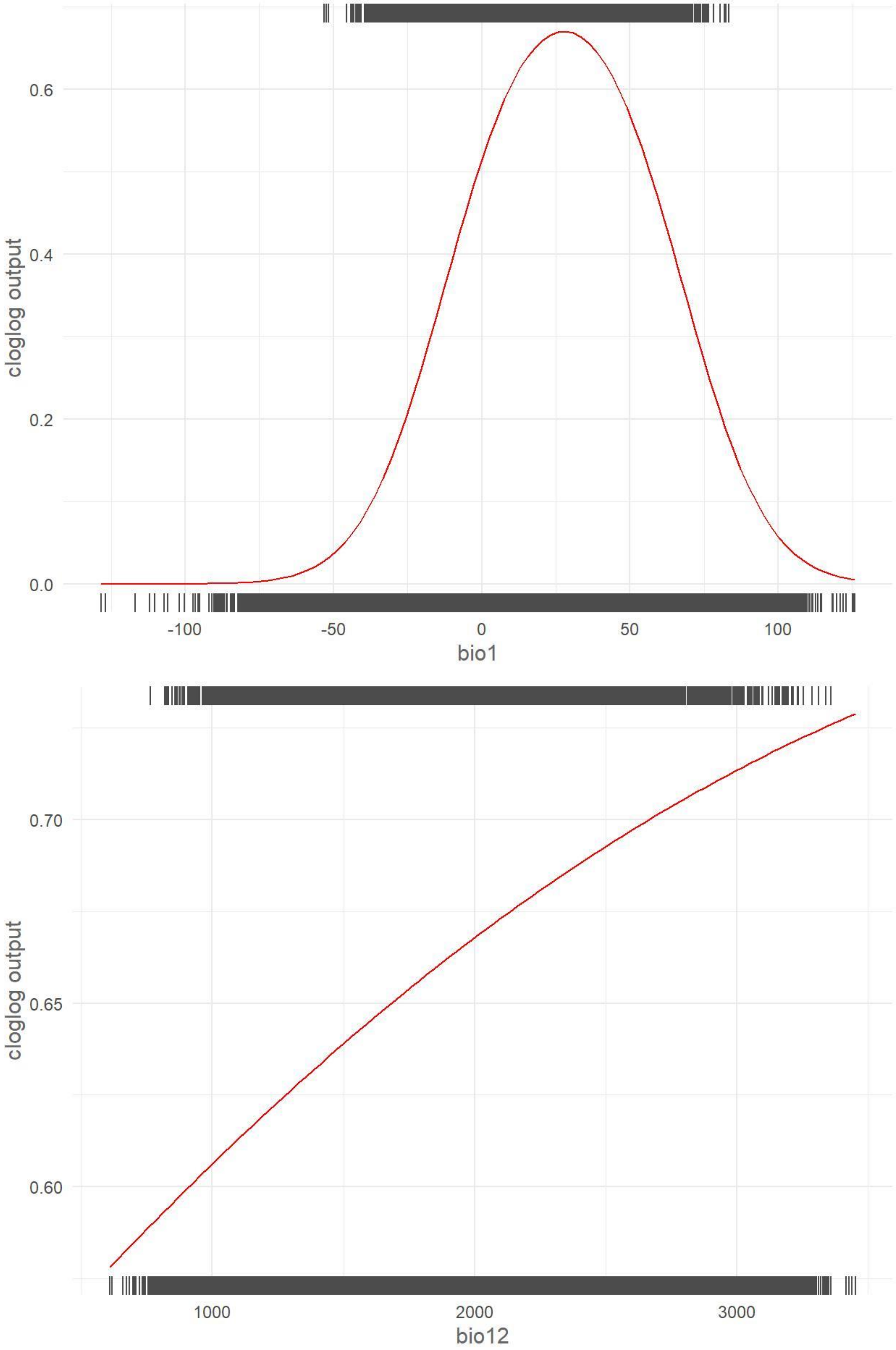

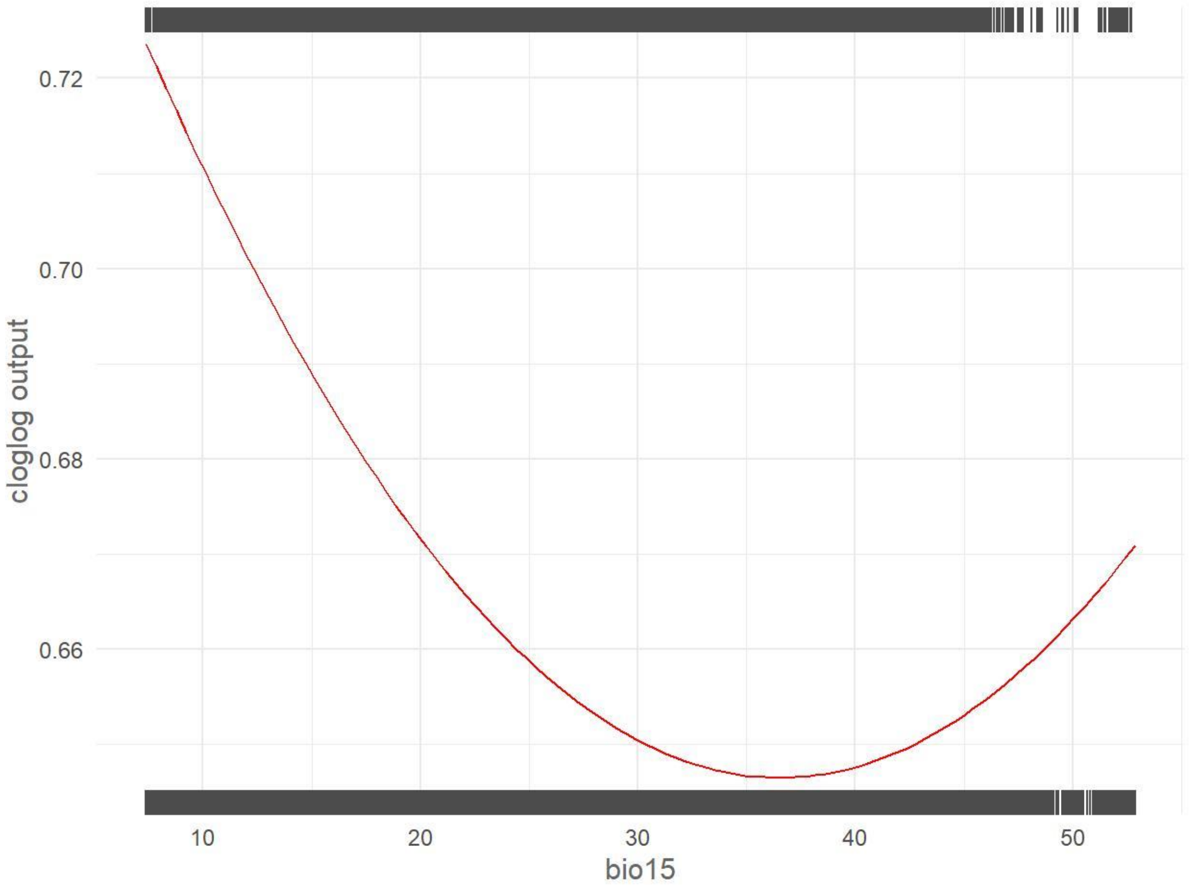
0.72
0.70
0.68
0.66
cloglog output
10
20
30
40
50
bio15

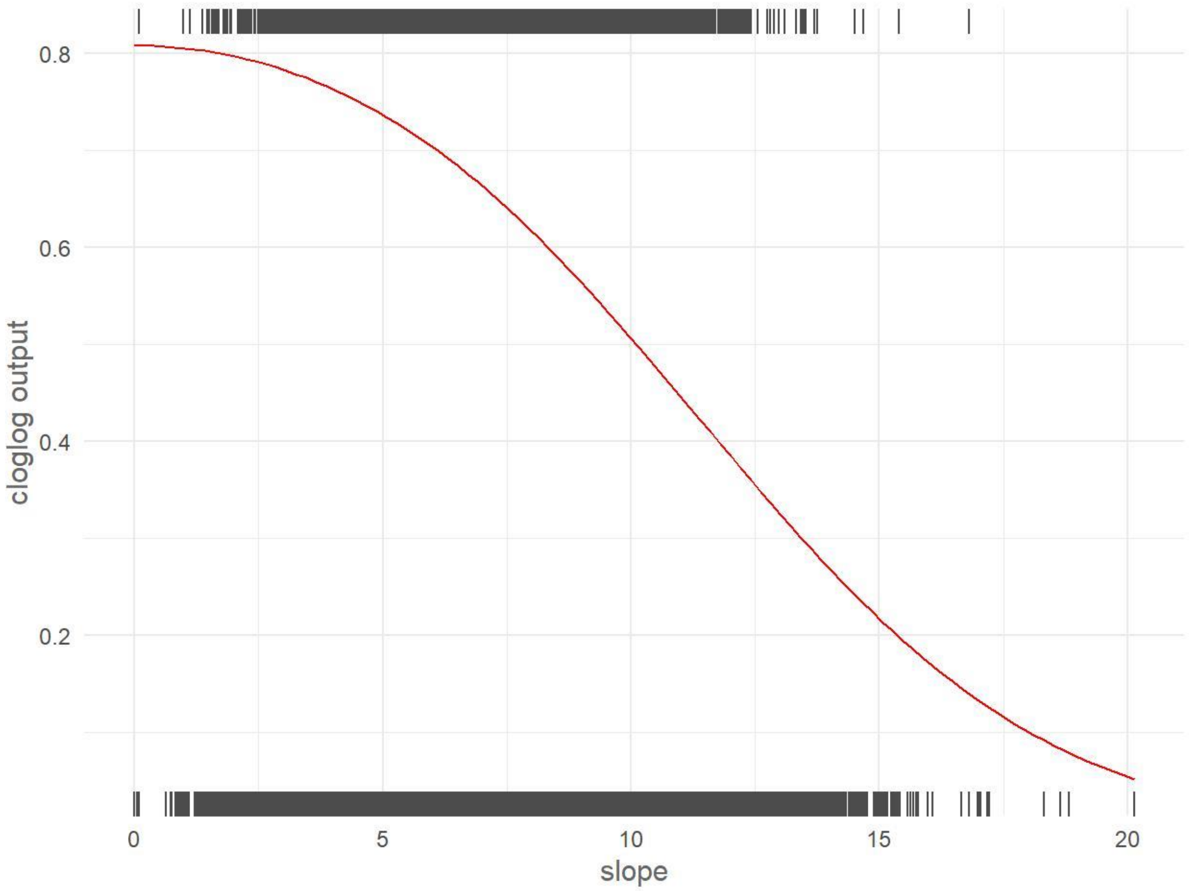
0.8
0.6
0.4
0.2
cloglog output
0
5
10
15
20
slope

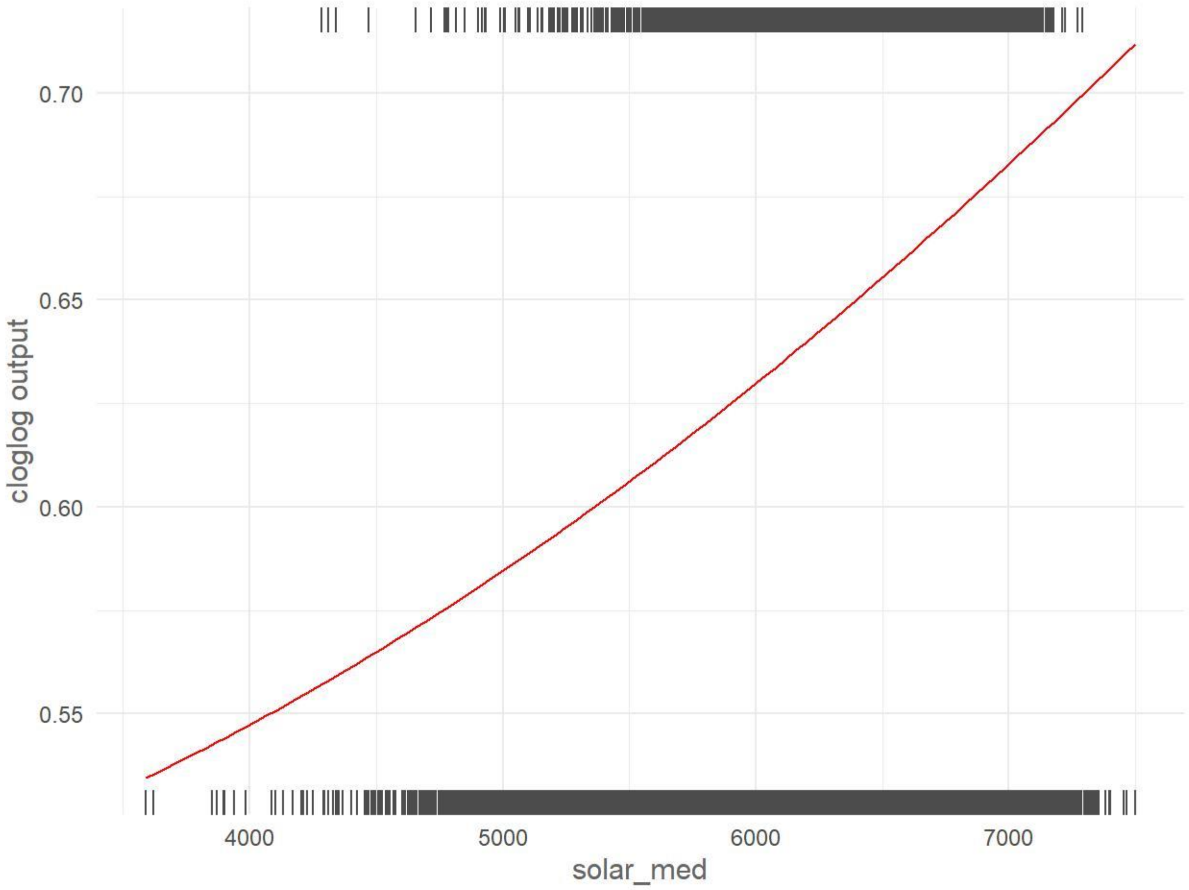

0.70
0.65
0.60
0.55
cloglog output
4000
5000
6000
7000
solar_med

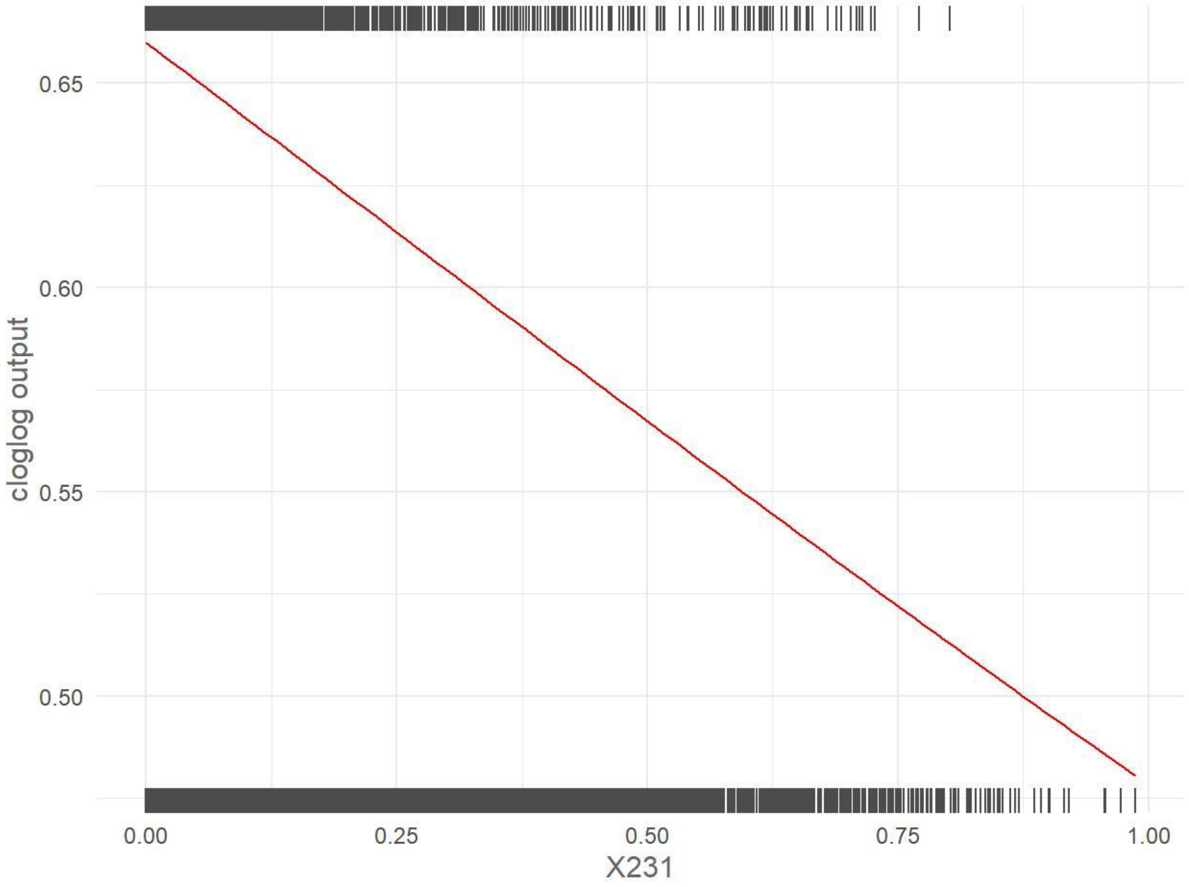

0.65
0.60
0.55
0.50
cloglog output
0.00
0.25
0.50
0.75
1.00
X231

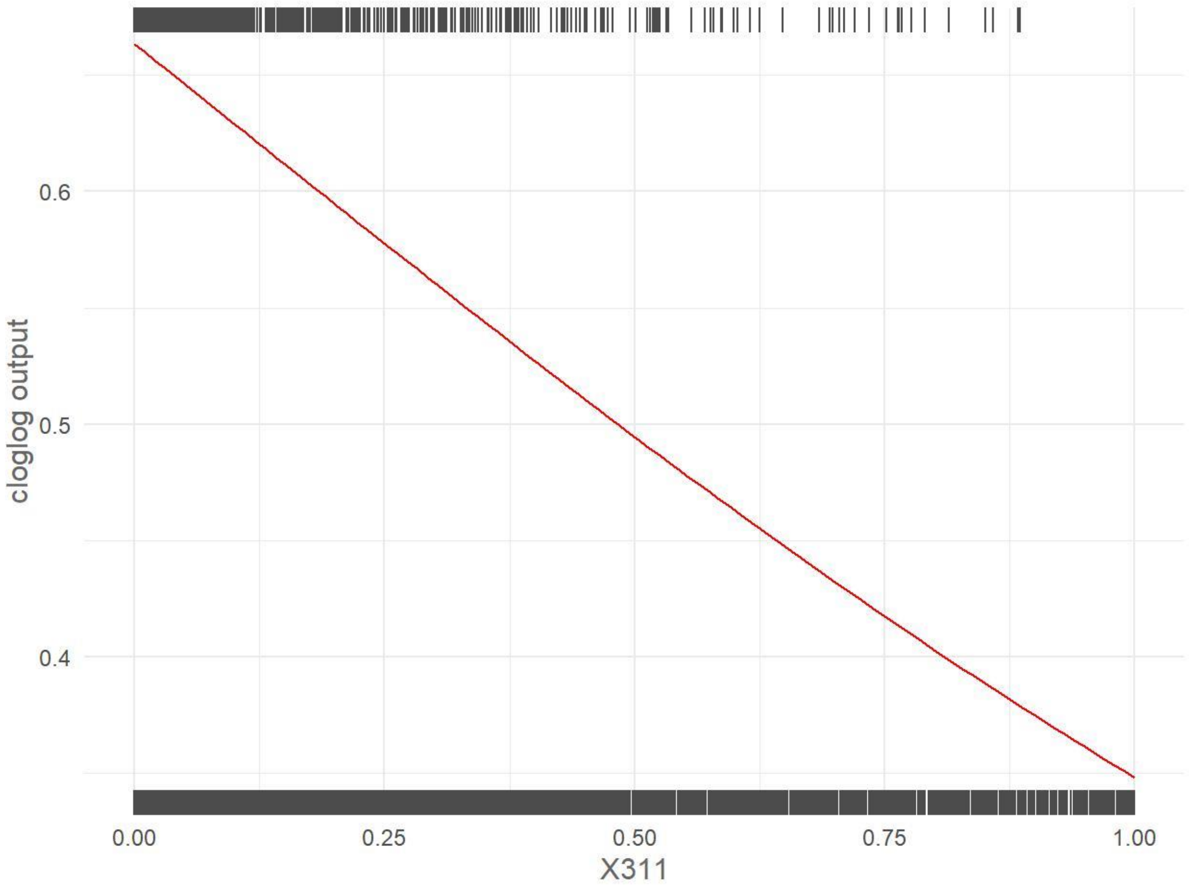

0.6
0.5
0.4
cloglog output
0.00
0.25
0.50
0.75
1.00
X311

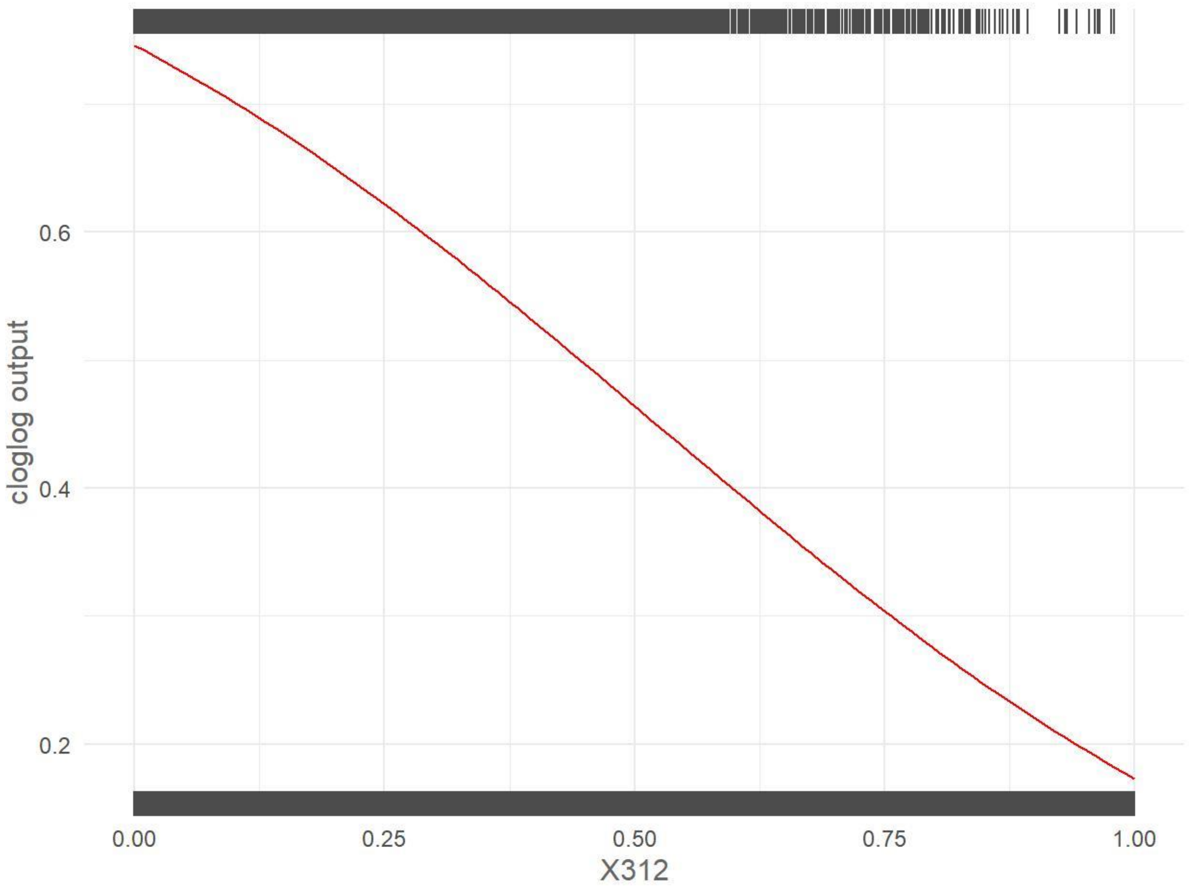

0.6
0.4
0.2
cloglog output
0.00
0.25
0.50
0.75
1.00
X312

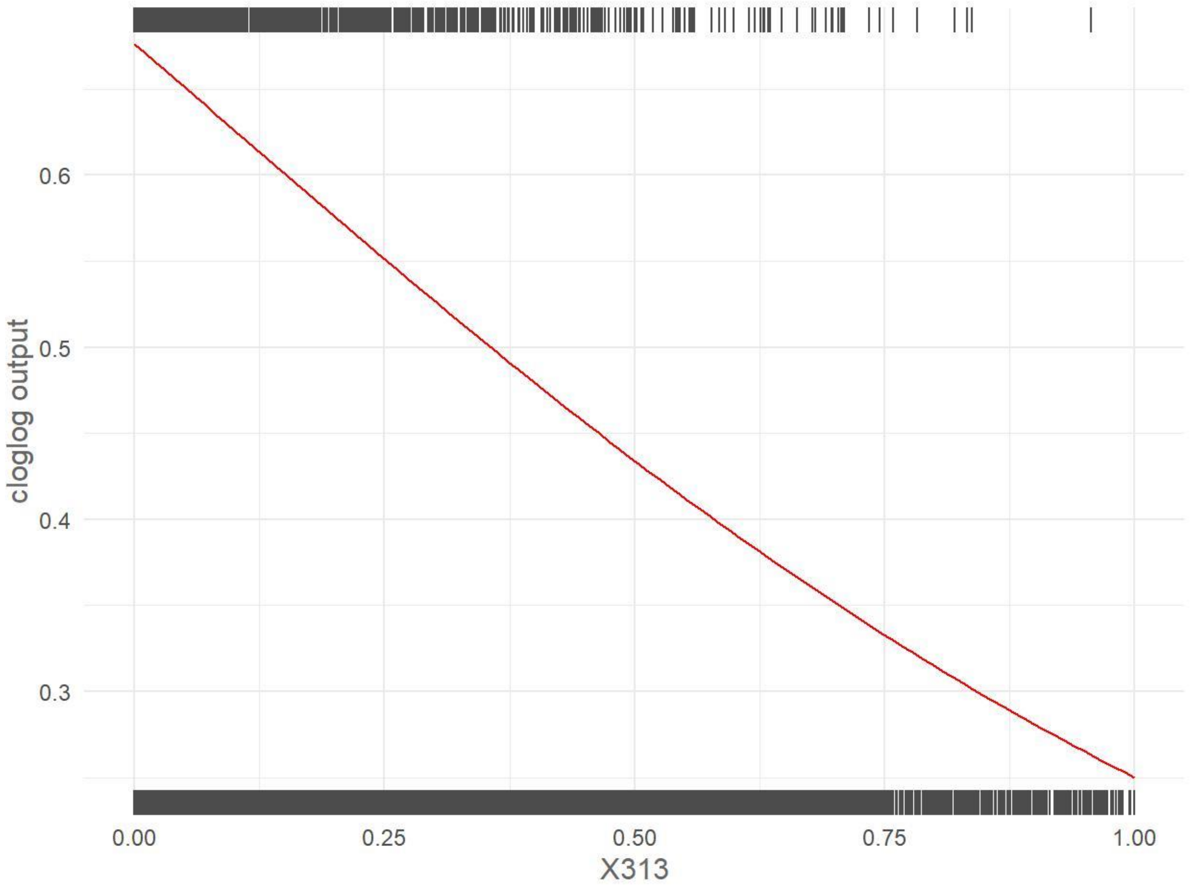
cloglog output
0.6
0.5
0.4
0.3
0.00
0.25
0.50
0.75
1.00
X313

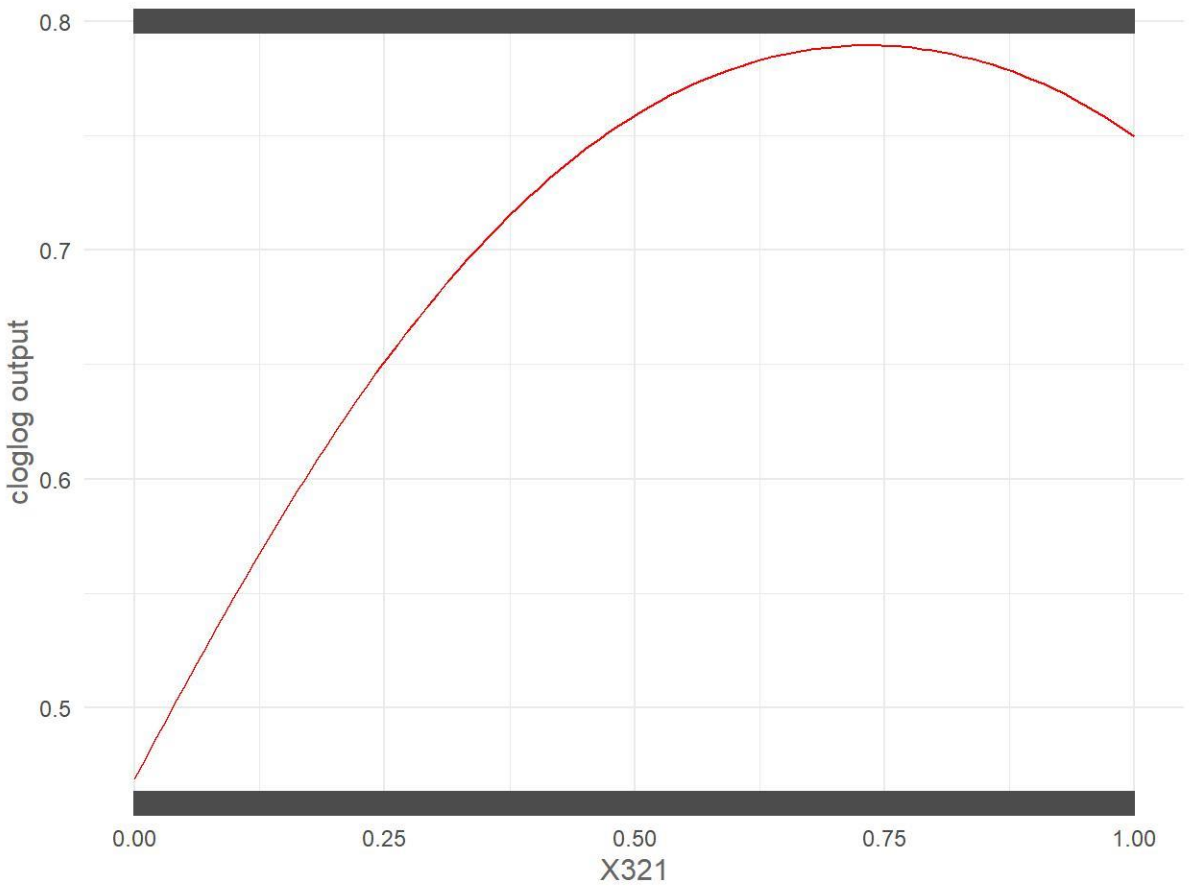
cloglog output
0.8
0.7
0.6
0.5
0.00
0.25
0.50
0.75
1.00
X321

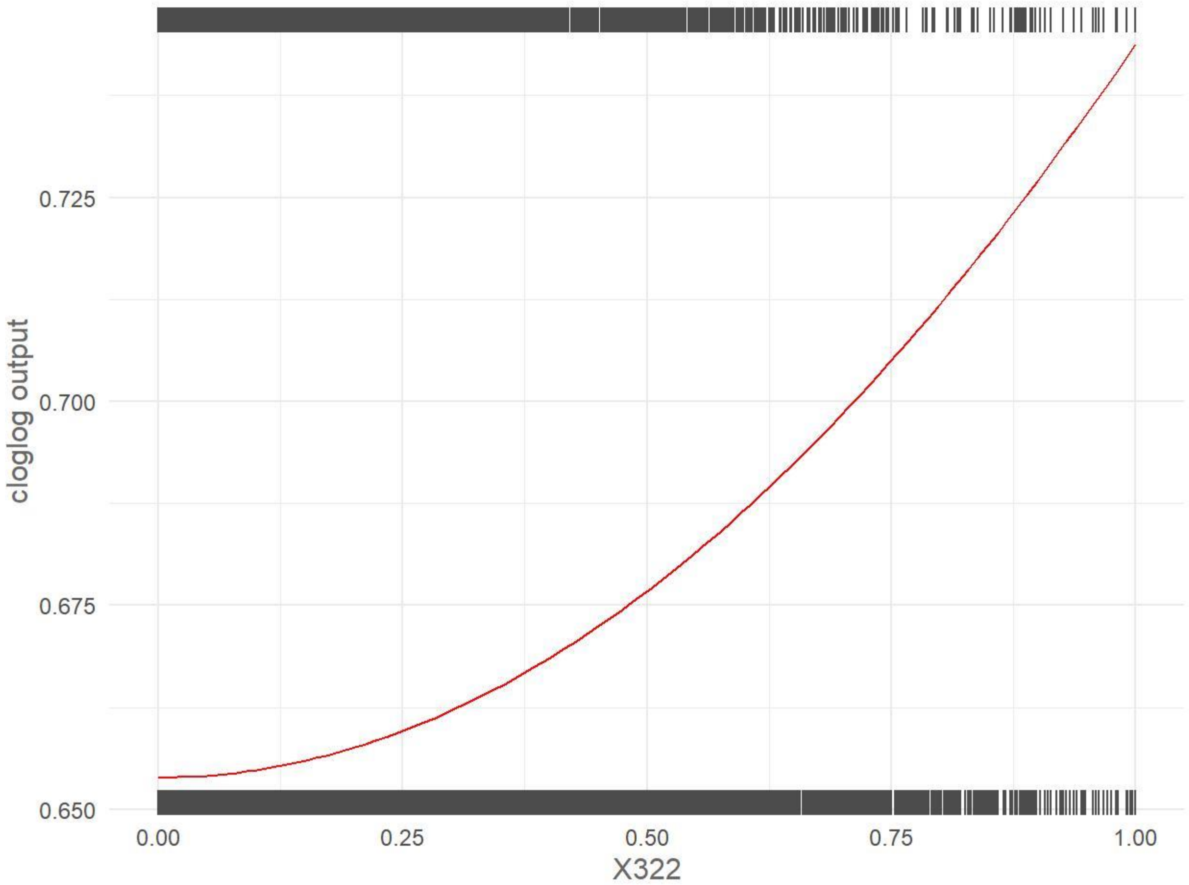

cloglog output
0.650
0.675
0.700
0.725
0.00
0.25
0.50
0.75
1.00
X322

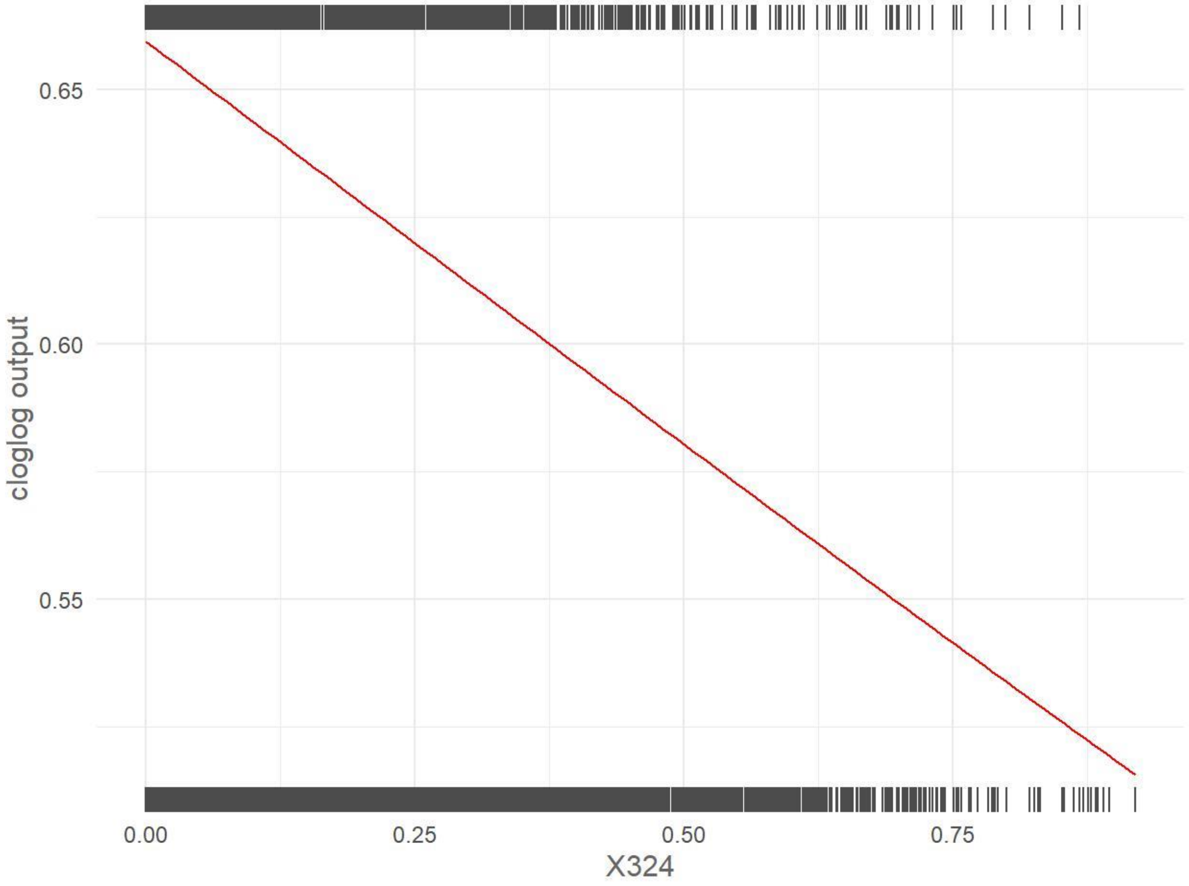

cloglog output
0.55
0.60
0.65
0.00
0.25
0.50
0.75
X324

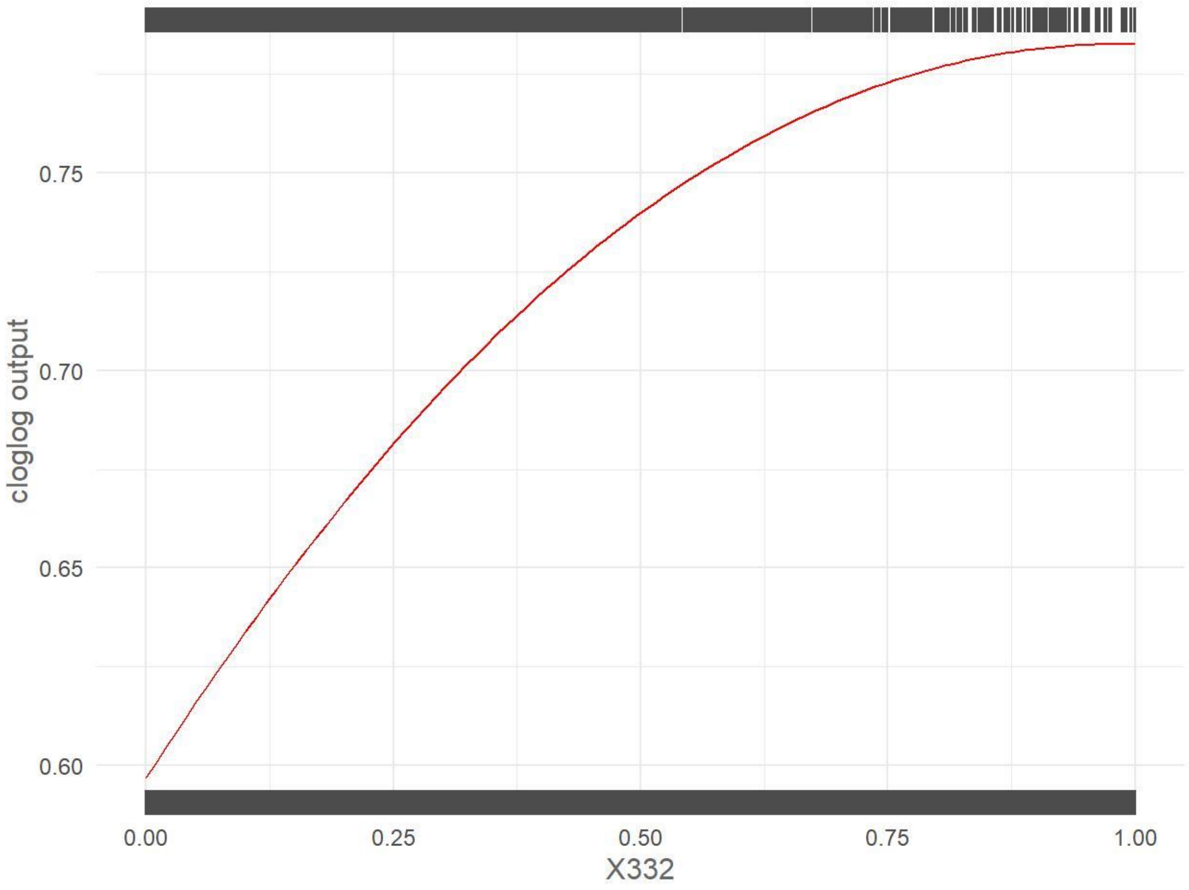

cloglog output
0.75
0.70
0.65
0.60
0.00
0.25
0.50
0.75
1.00
X332

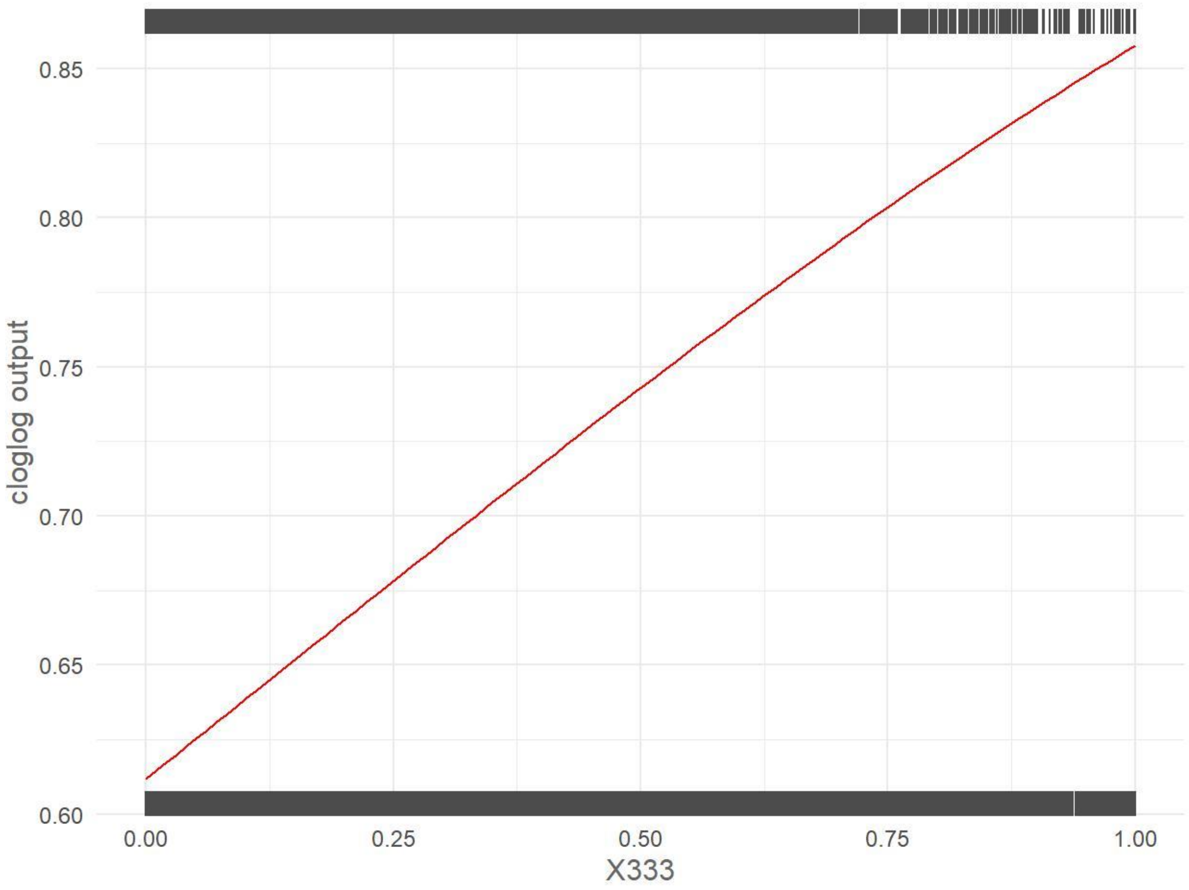

cloglog output
0.85
0.80
0.75
0.70
0.65
0.60
0.00
0.25
0.50
0.75
1.00
X333

Figure S3. Species-habitat relationships according to the MaxEnt model for alpine accentor.

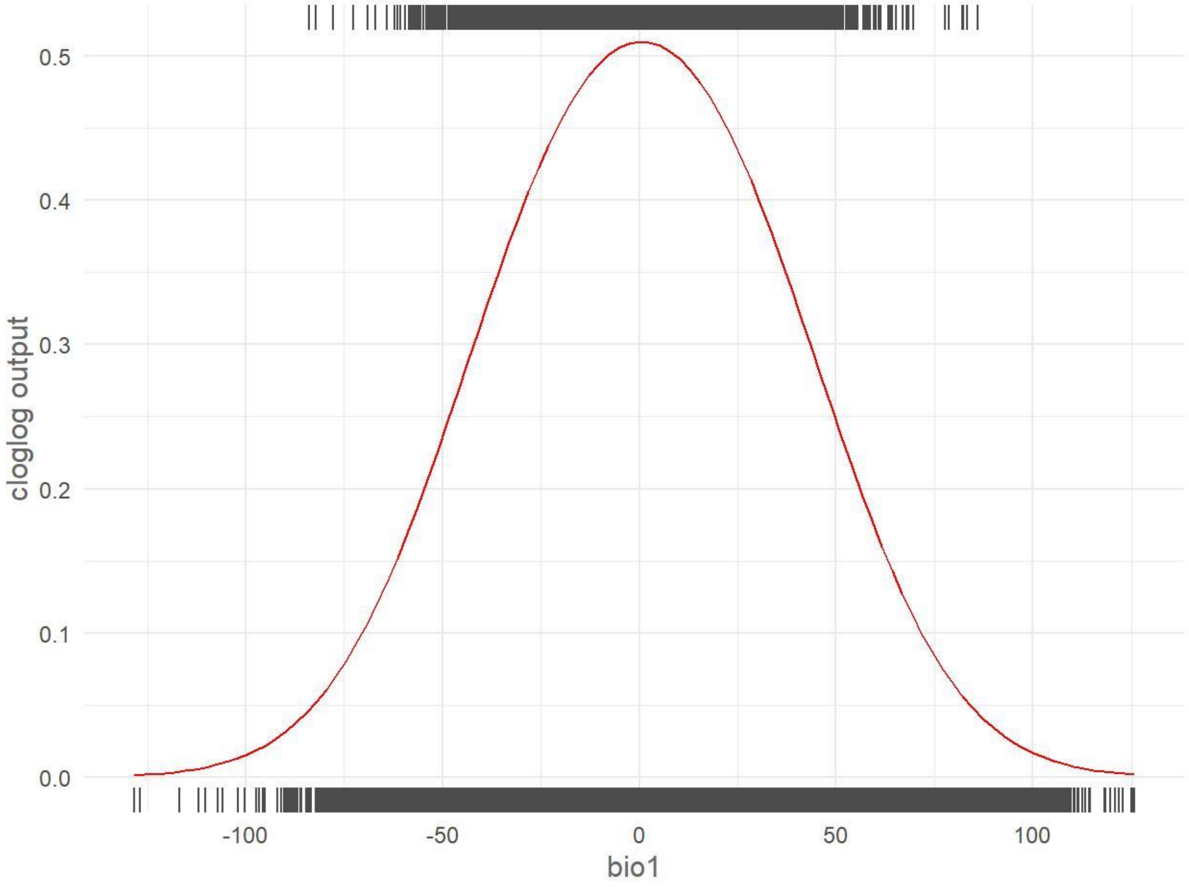

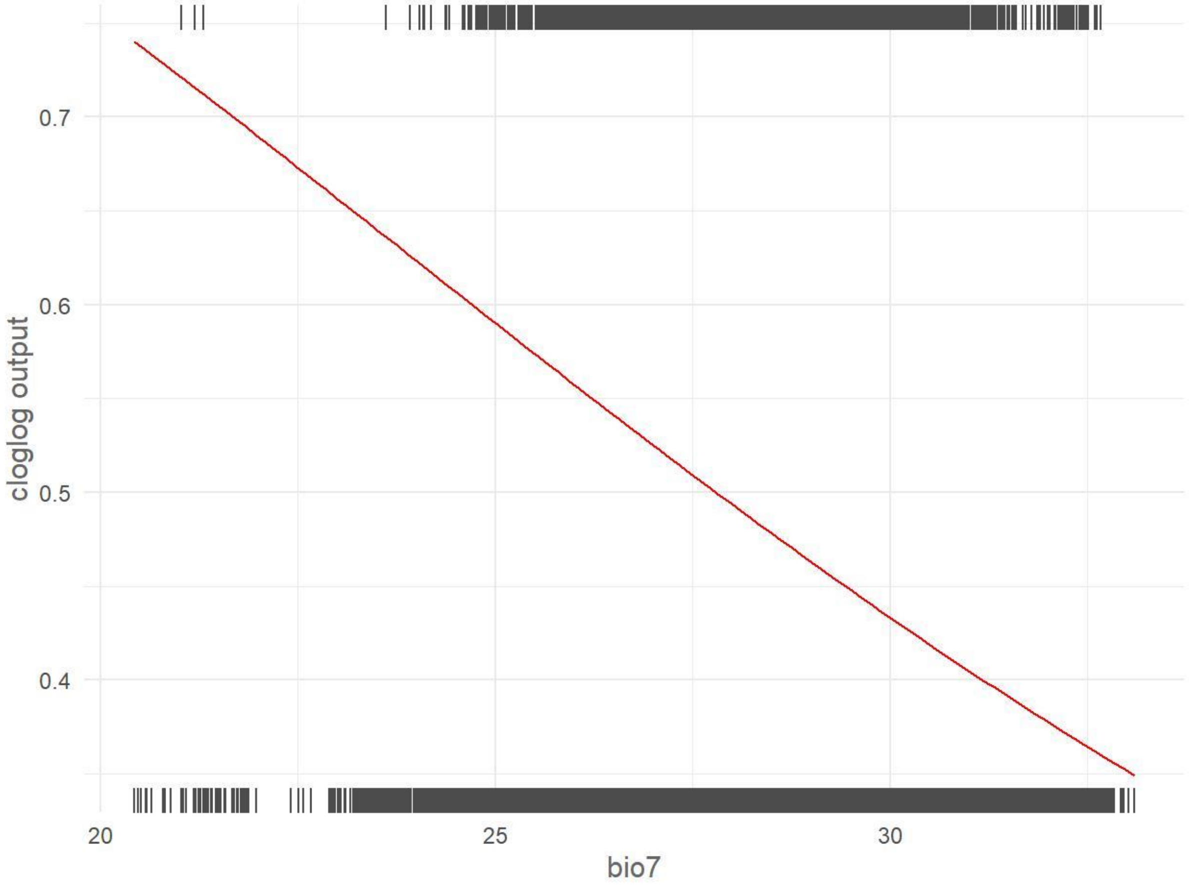

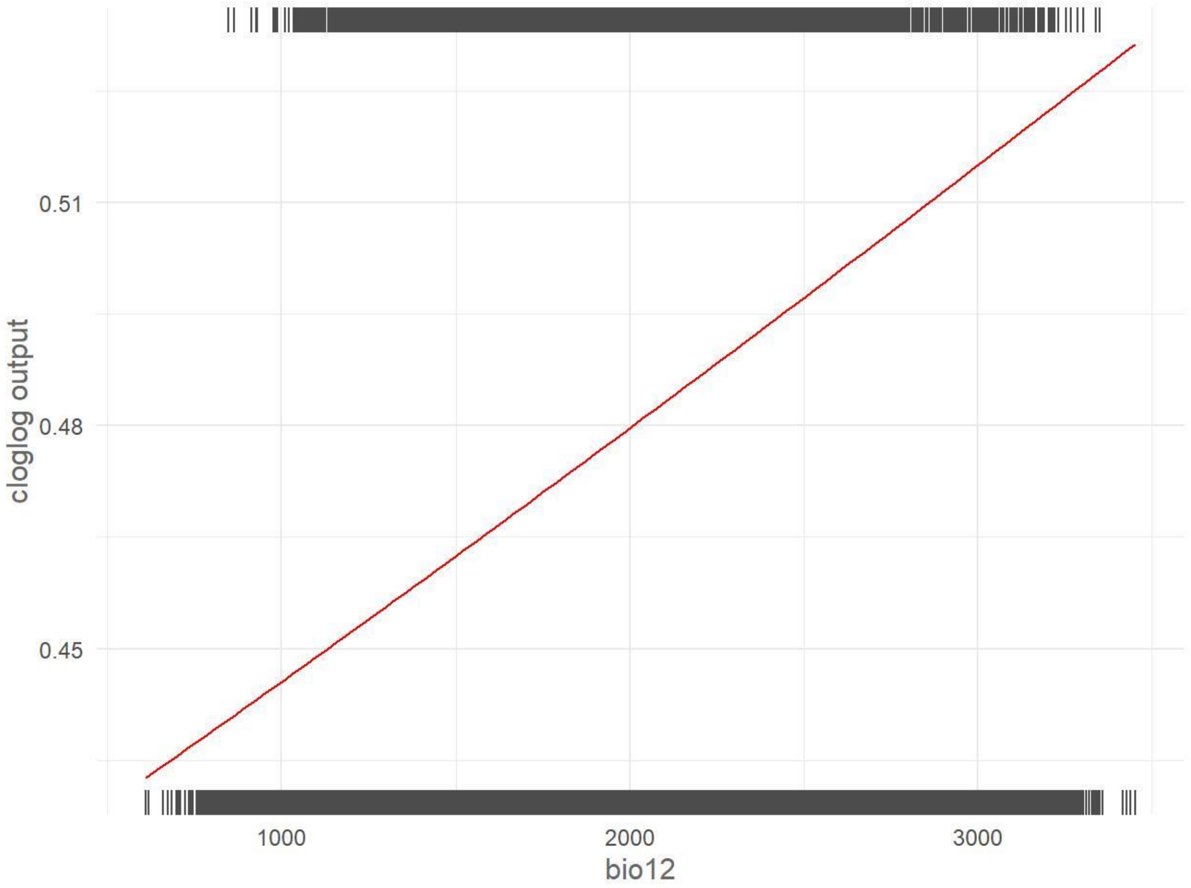
cloglog output
0.51
0.48
0.45
1000
2000
3000
bio12

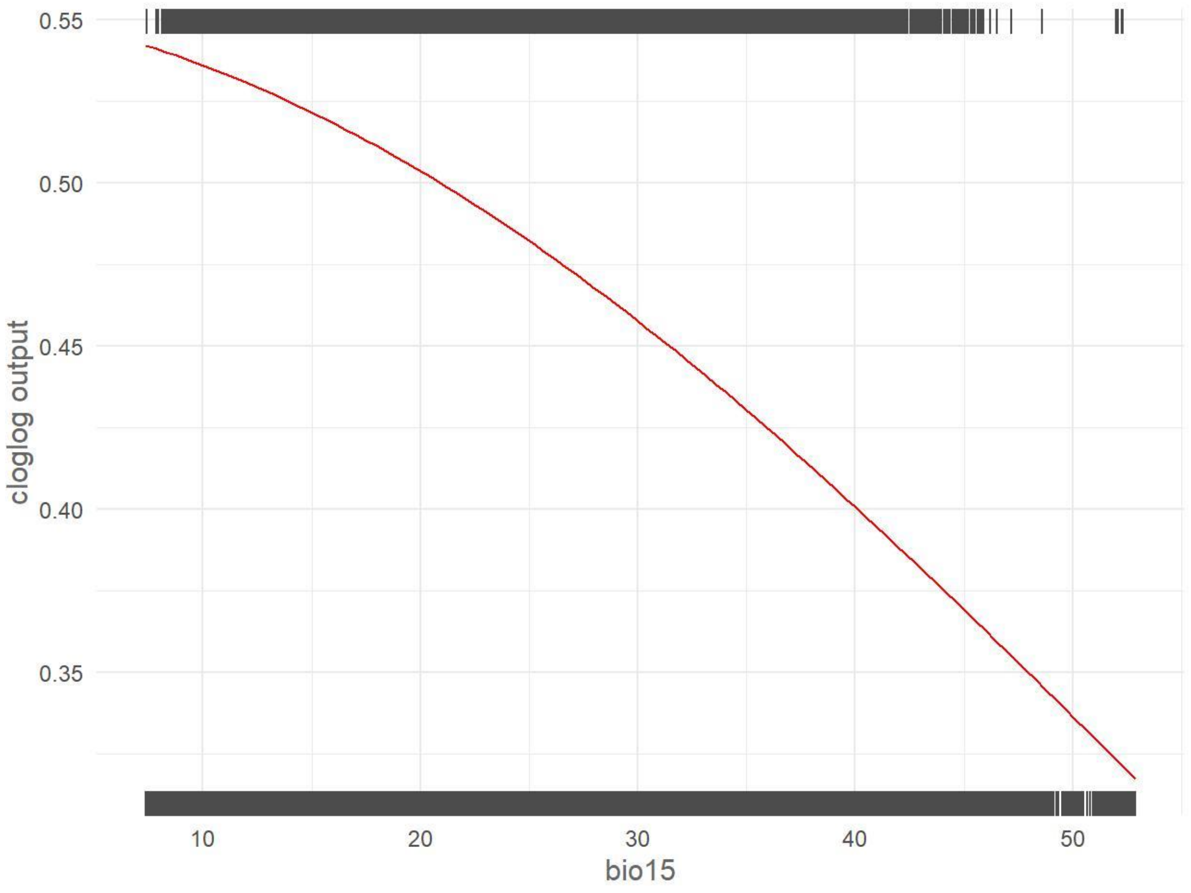
cloglog output
0.55
0.50
0.45
0.40
0.35
10
20
30
40
50
bio15

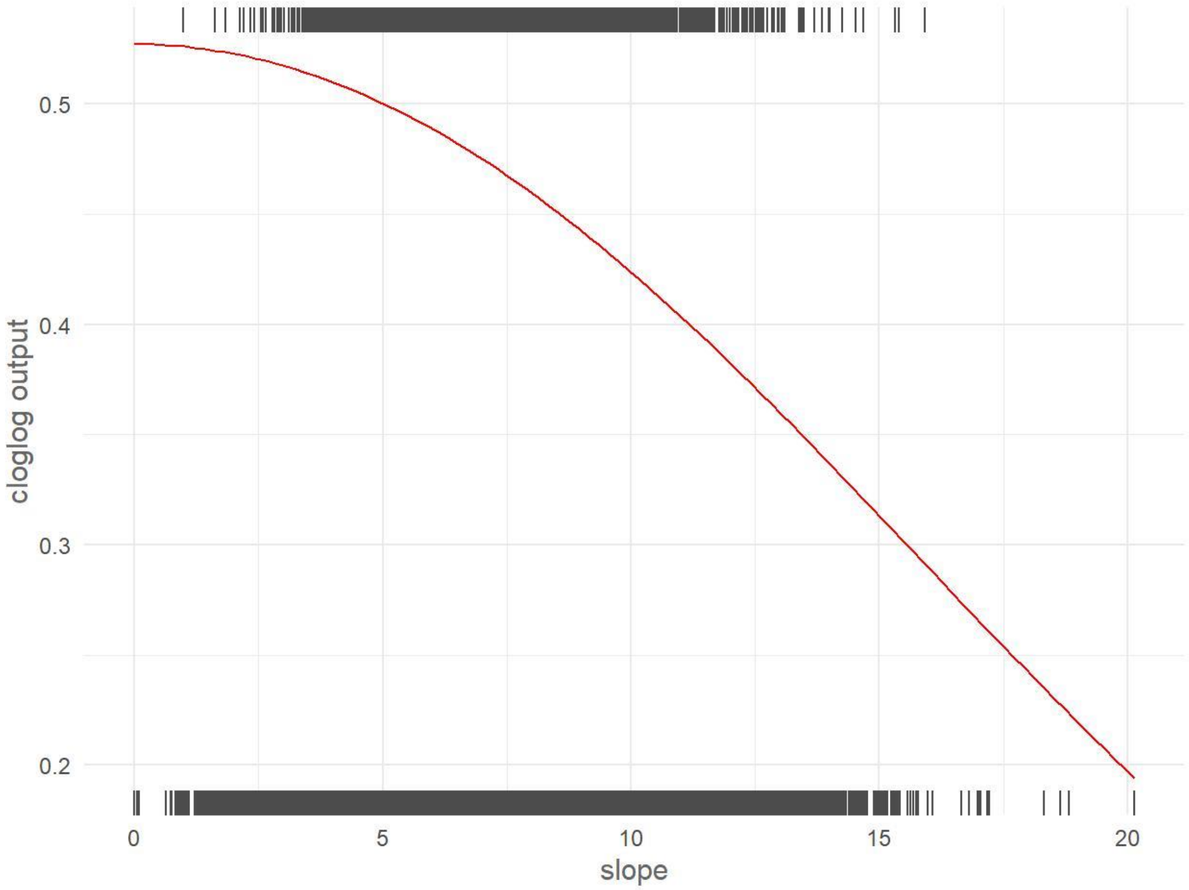

cloglog output
0.5
0.4
0.3
0.2
0
5
10
15
20
slope

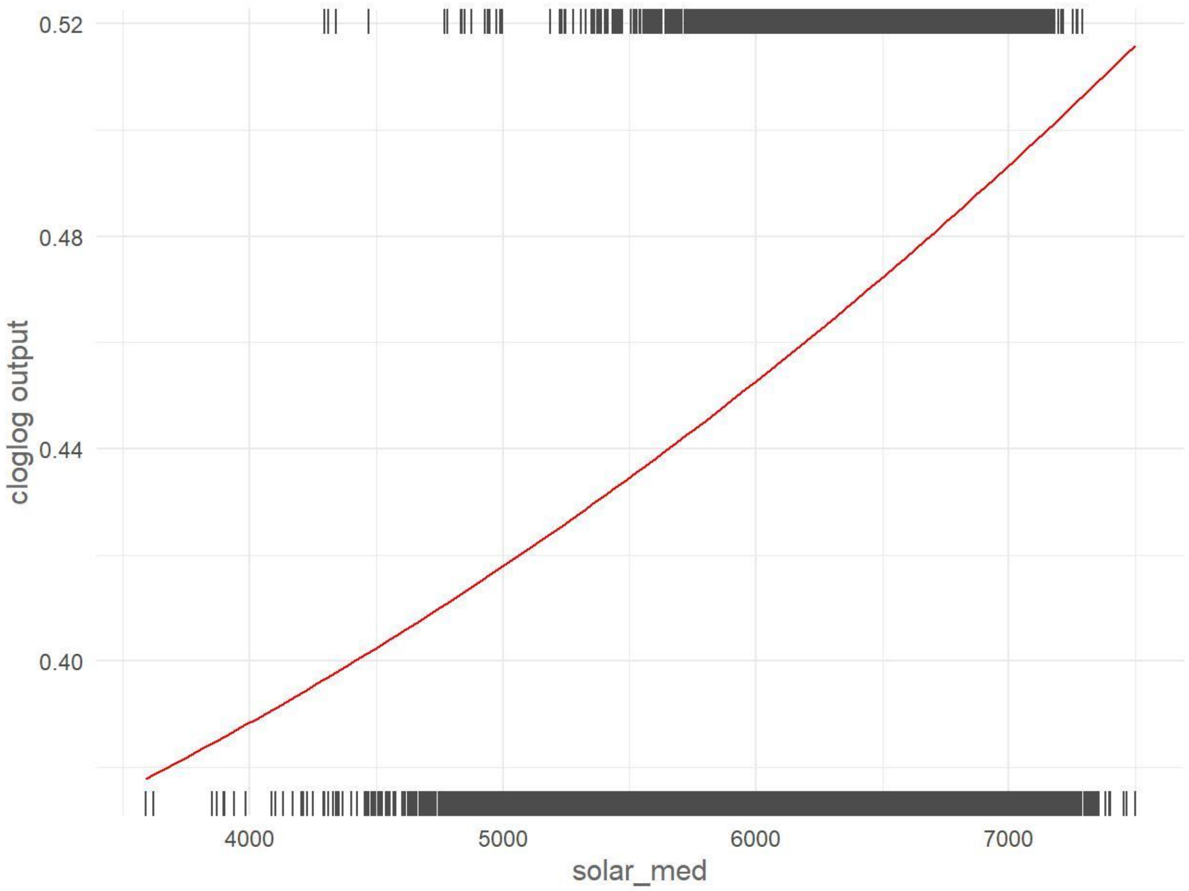

cloglog output
0.52
0.48
0.44
0.40
4000
5000
6000
7000
solar_med

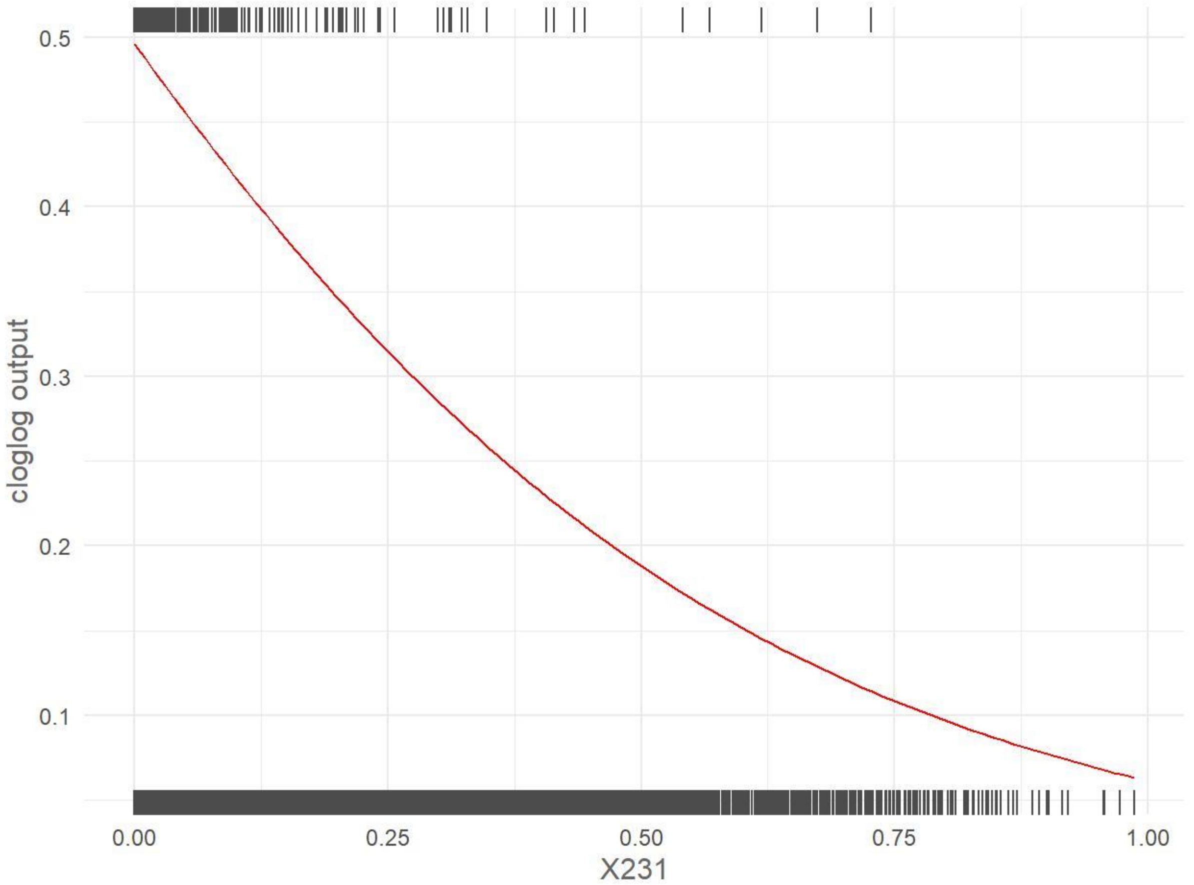

cloglog output
0.5
0.4
0.3
0.2
0.1
0.00
0.25
0.50
0.75
1.00
X231

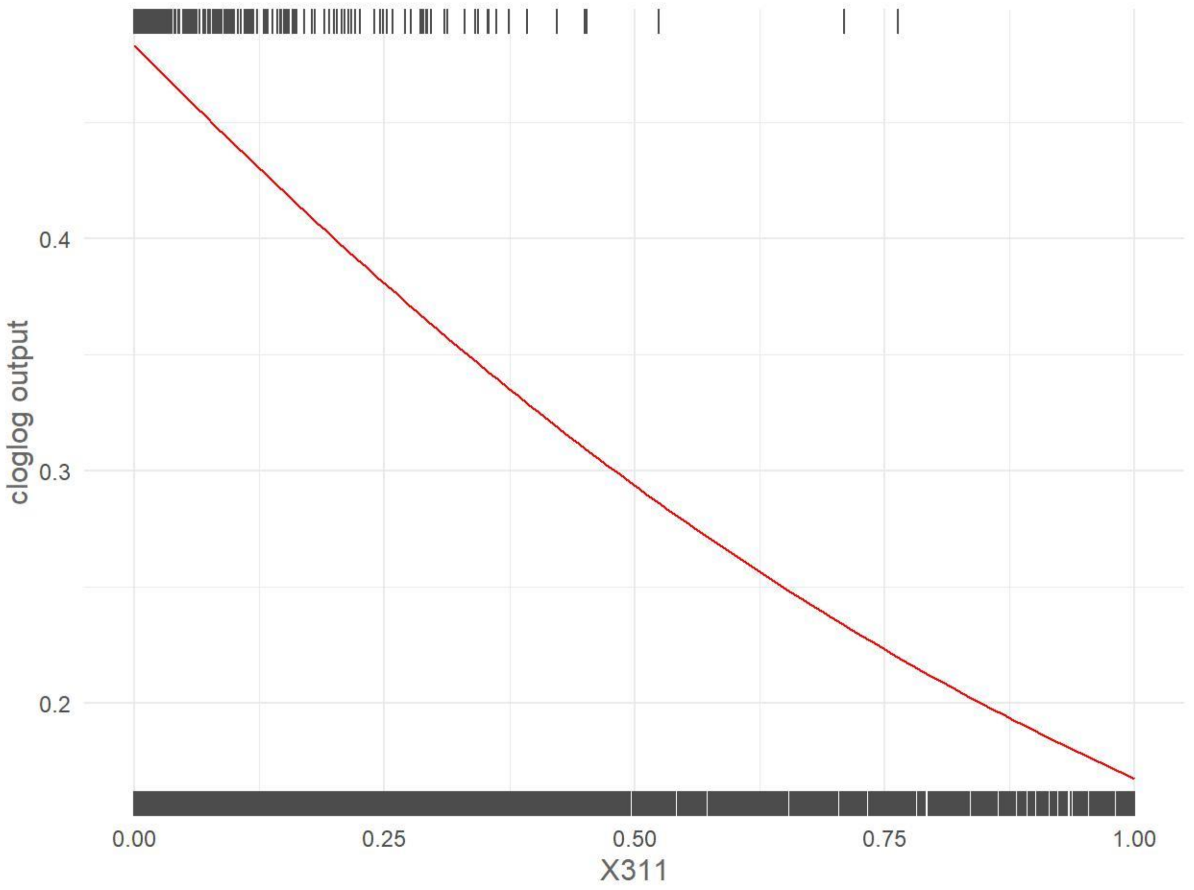

cloglog output
0.4
0.3
0.2
0.00
0.25
0.50
0.75
1.00
X311

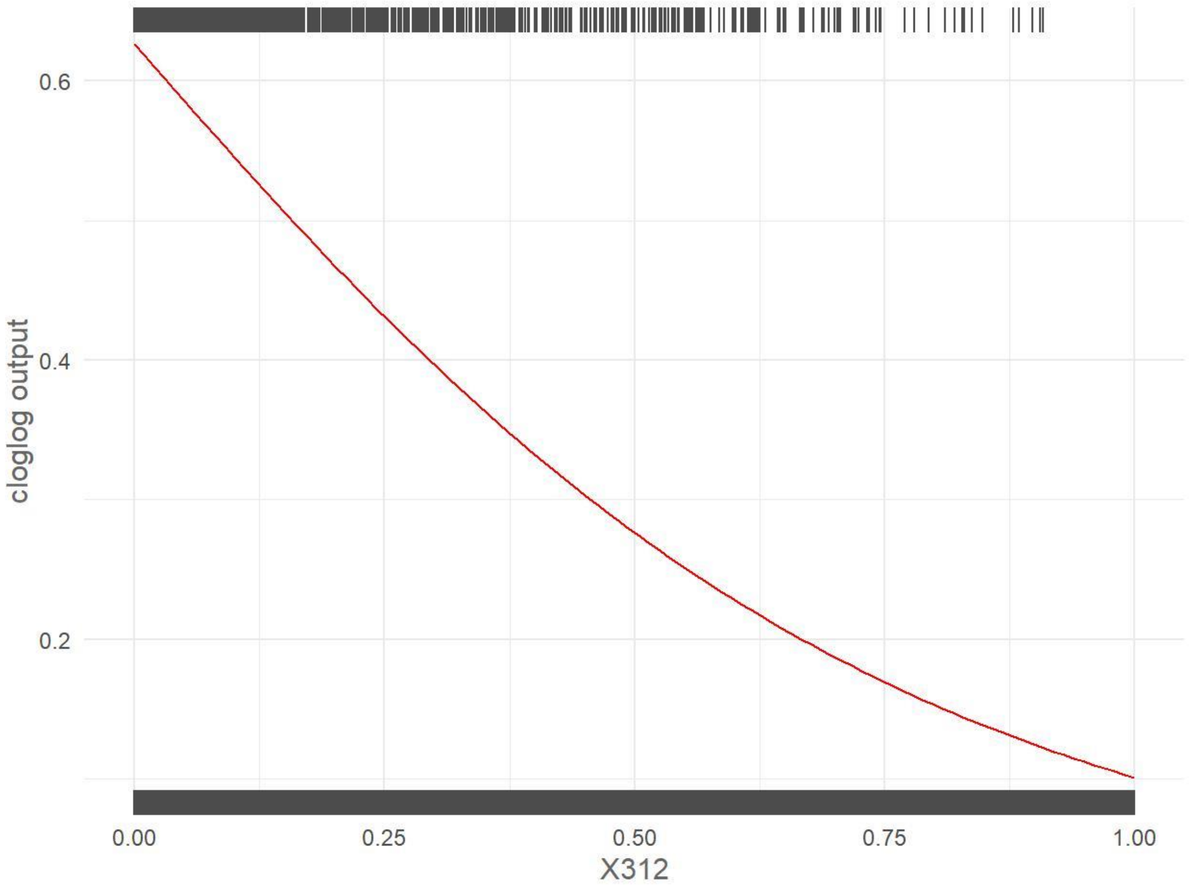
0.6
0.4
0.2
cloglog output
0.00
0.25
0.50
0.75
1.00
X312
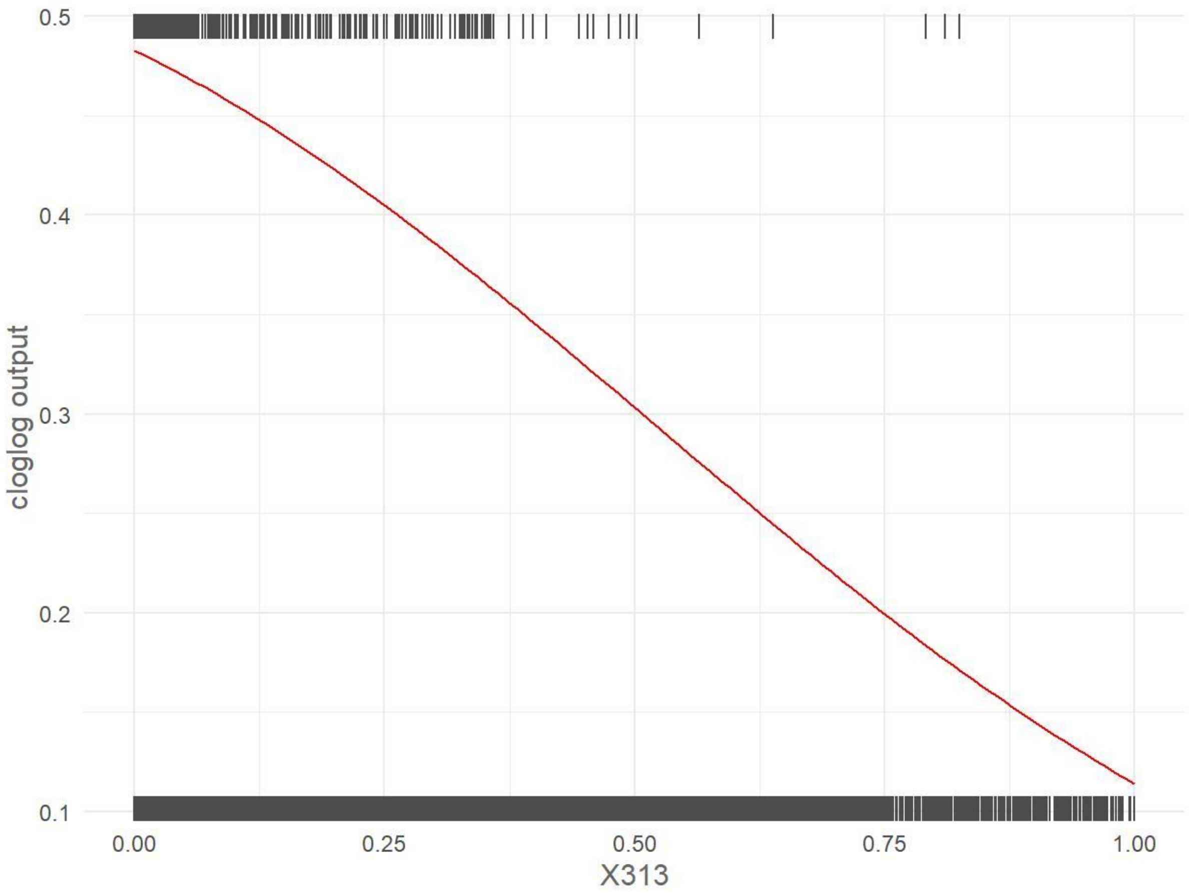
0.5
0.4
0.3
0.2
0.1
cloglog output
0.00
0.25
0.50
0.75
1.00
X313

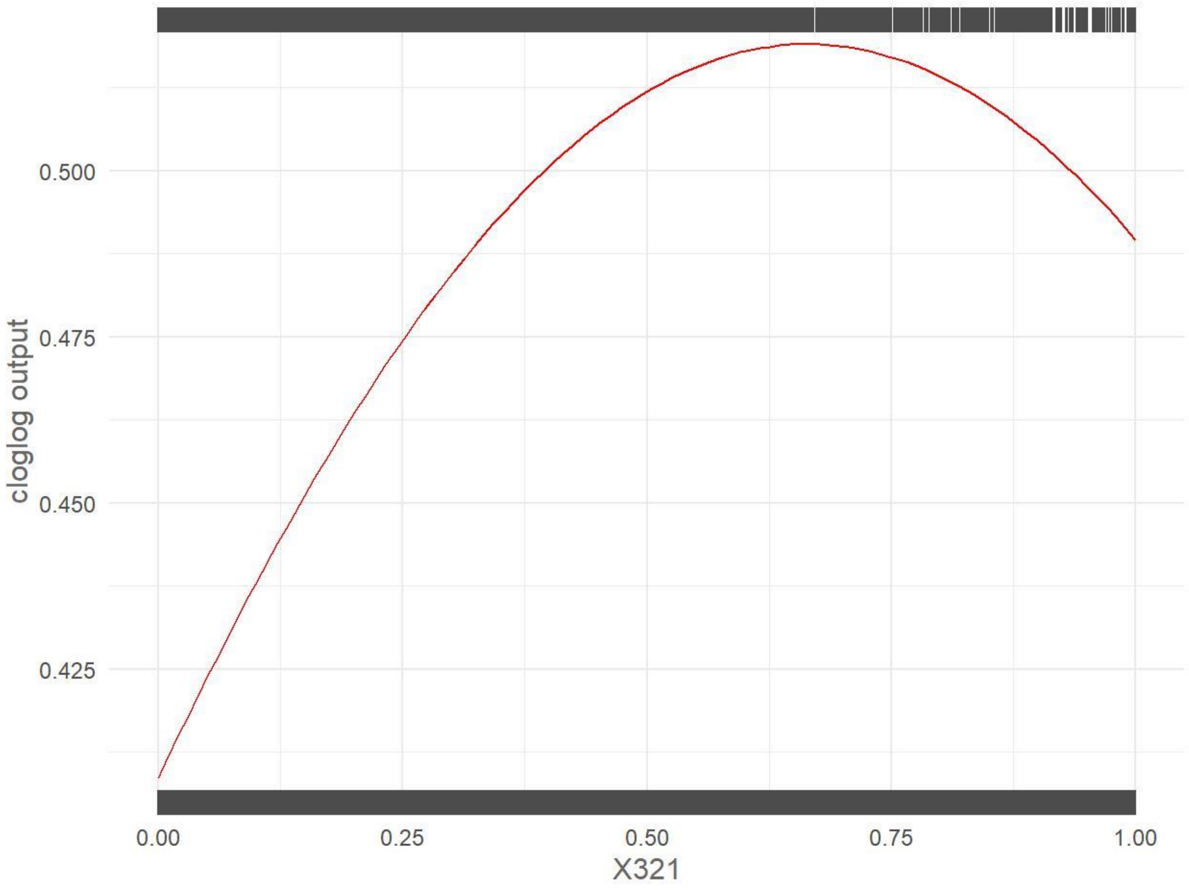

cloglog output
0.500
0.475
0.450
0.425
0.00
0.25
0.50
0.75
1.00
X321

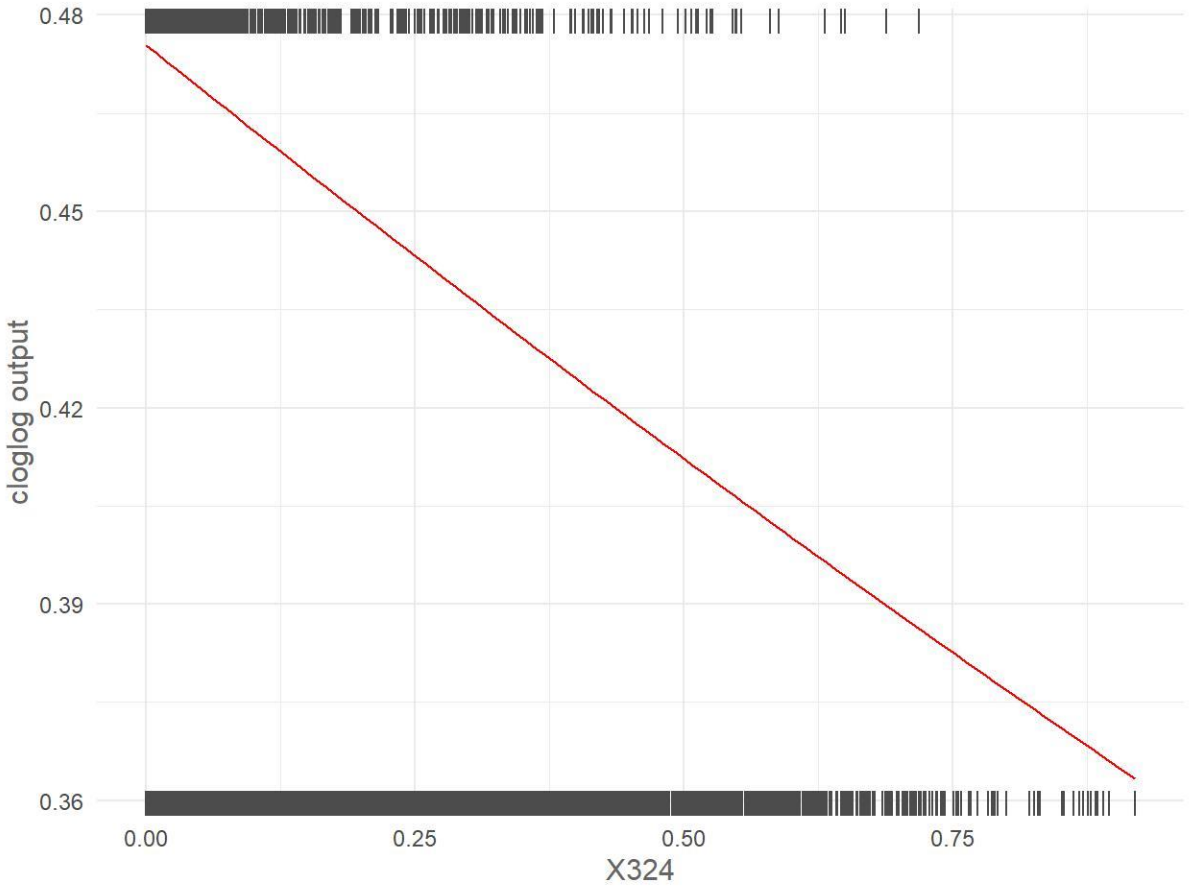

cloglog output
0.48
0.45
0.42
0.39
0.36
0.00
0.25
0.50
0.75
X324

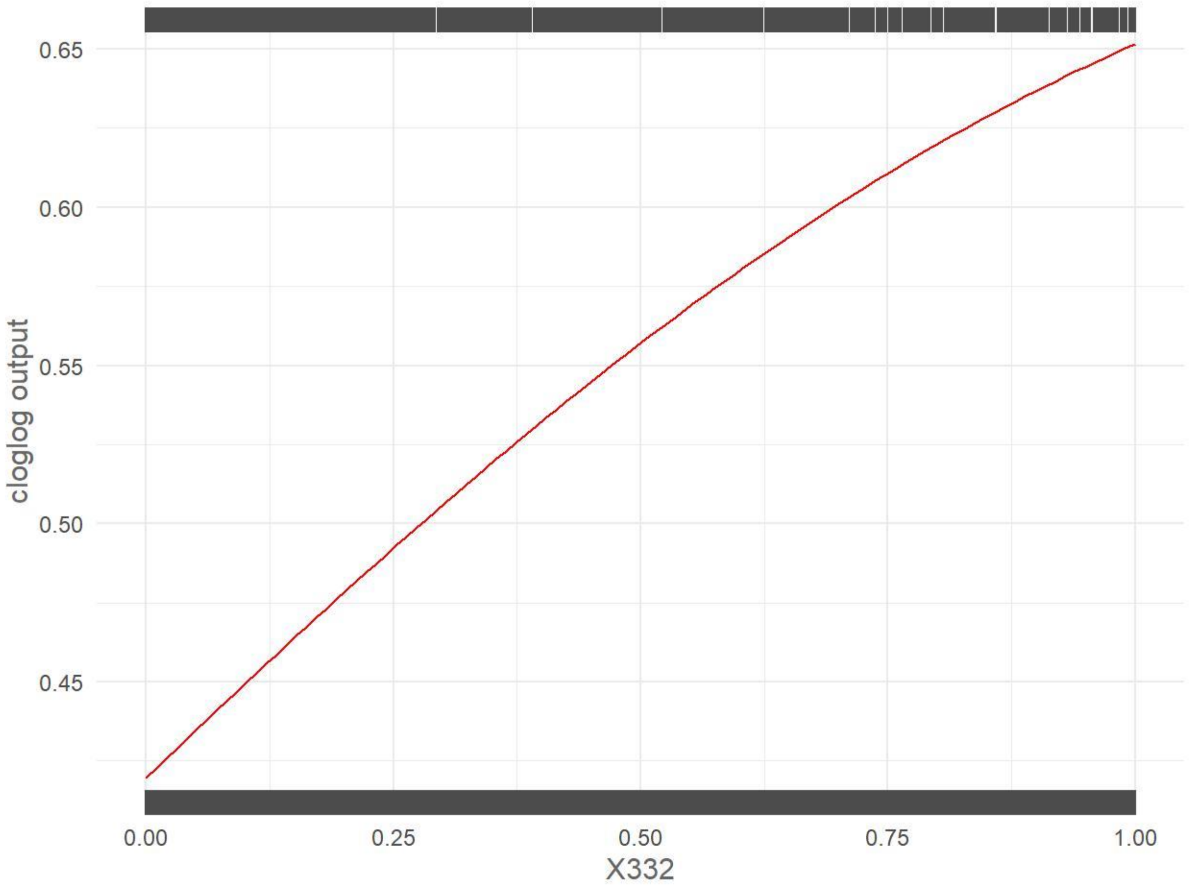
0.65
0.60
0.55
0.50
0.45
cloglog output
0.00
0.25
0.50
0.75
1.00
X332

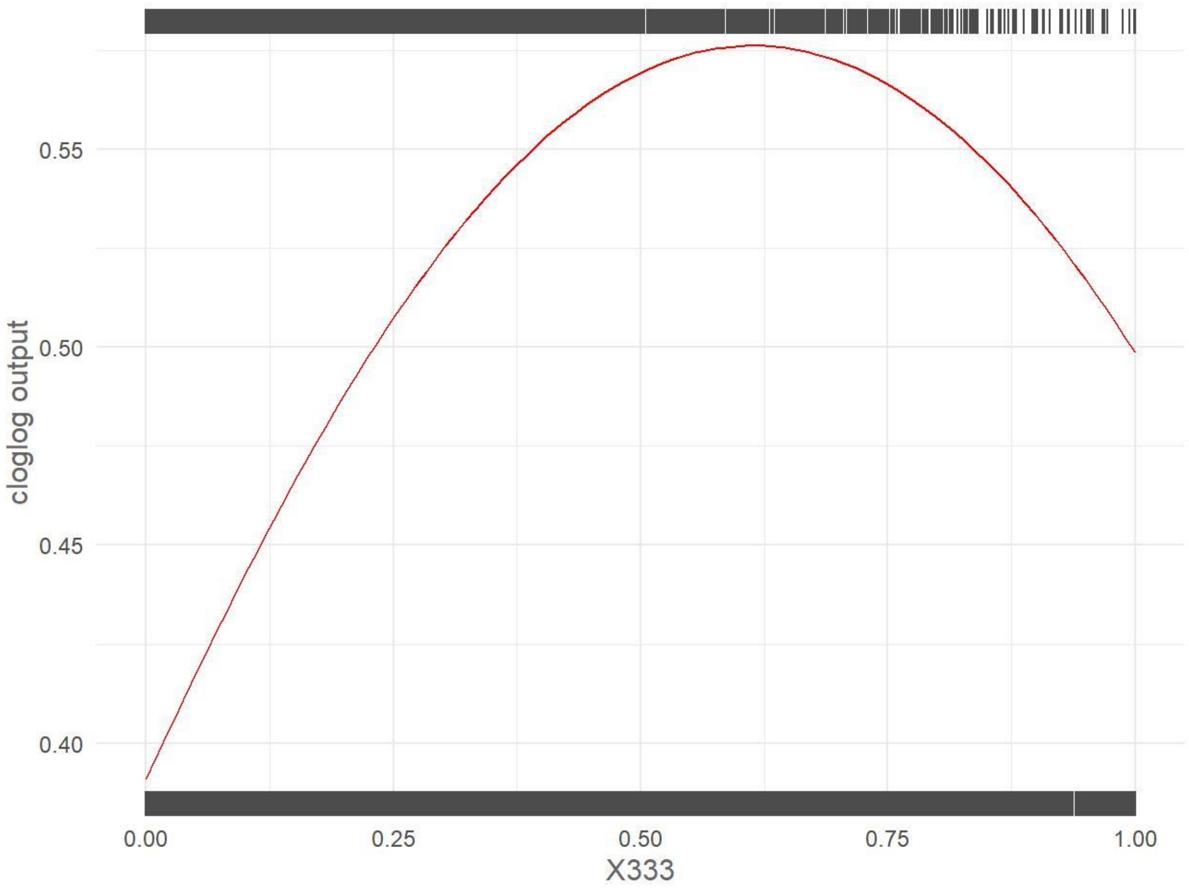
0.55
0.50
0.45
0.40
cloglog output
0.00
0.25
0.50
0.75
1.00
X333

Figure S4. Species-habitat relationships according to the MaxEnt model for white-winged snowfinch.

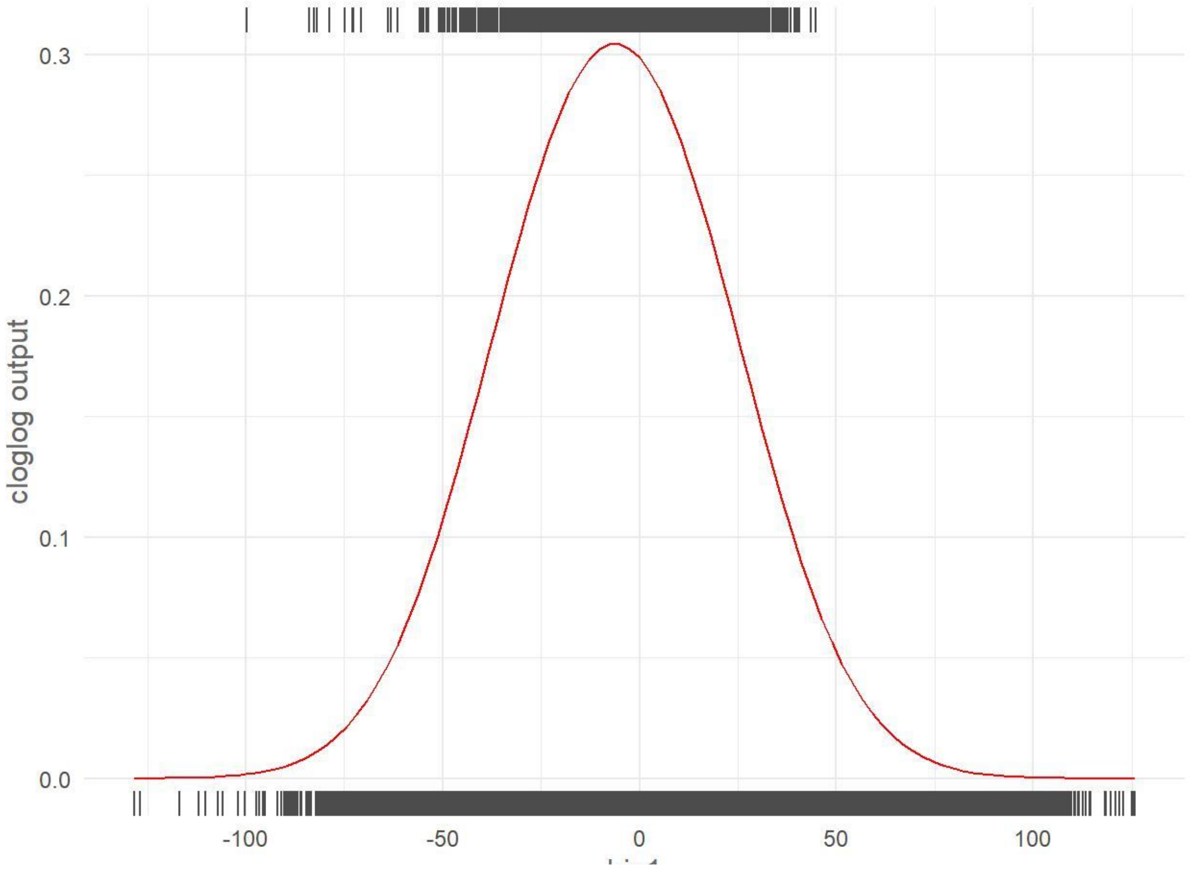
cloglog output
0.3
0.2
0.1
0.0
-100
-50
0
50
100

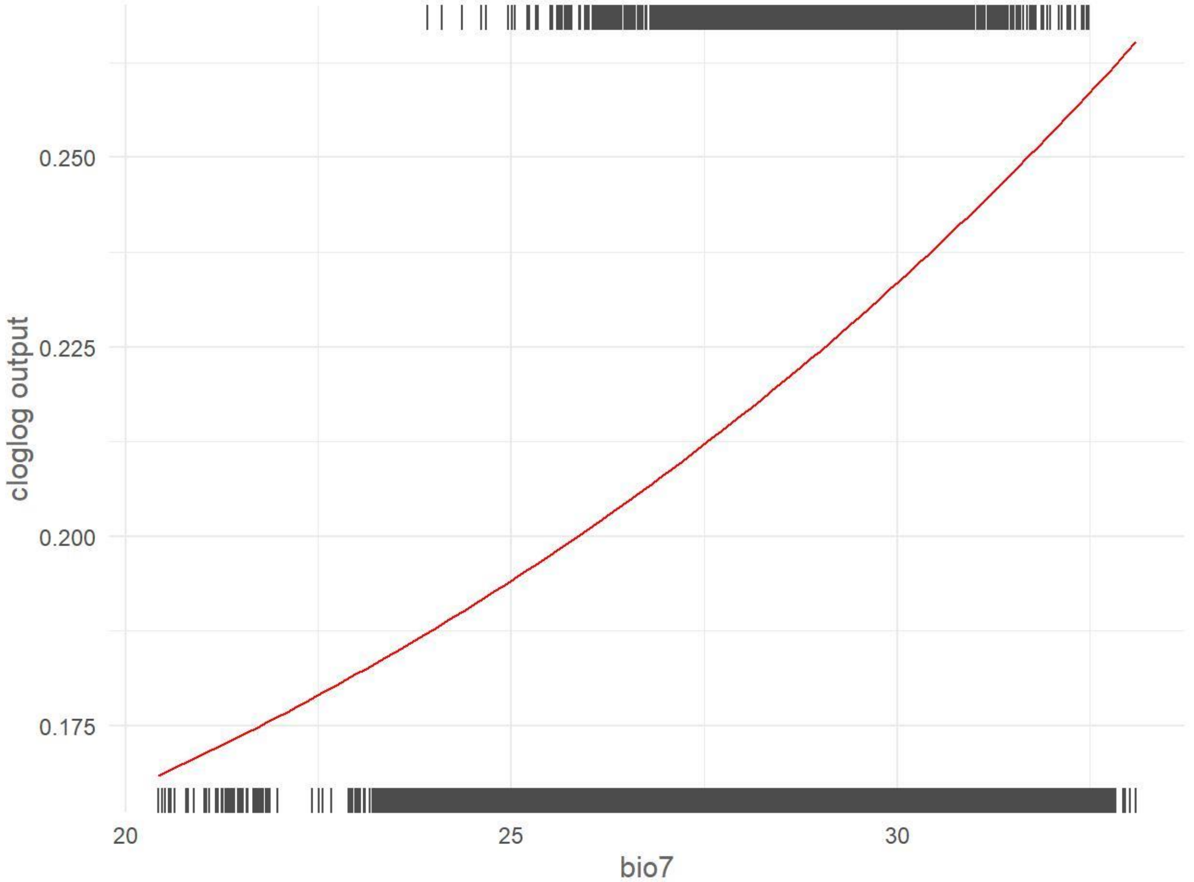
cloglog output
0.250
0.225
0.200
0.175
20
25
30
bio7

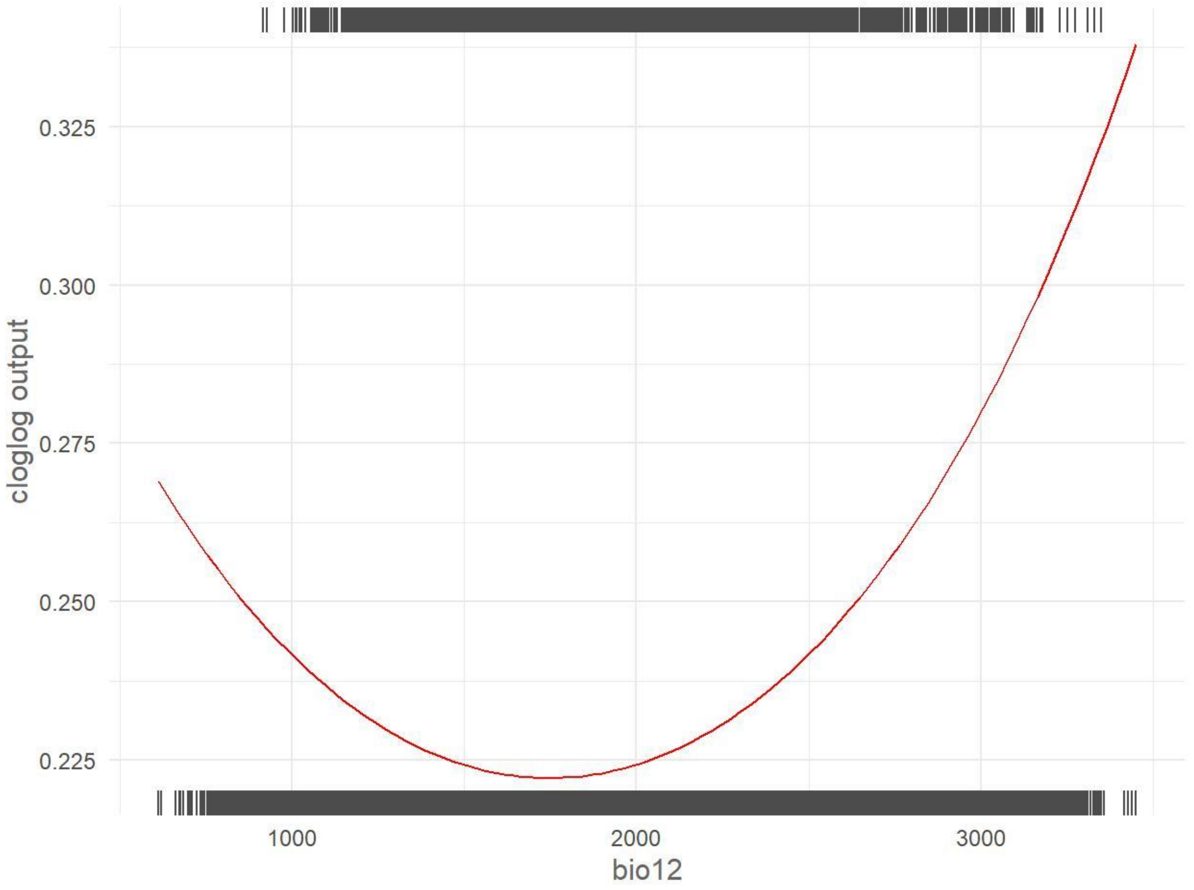

cloglog output
0.325
0.300
0.275
0.250
0.225
1000
2000
3000
bio12

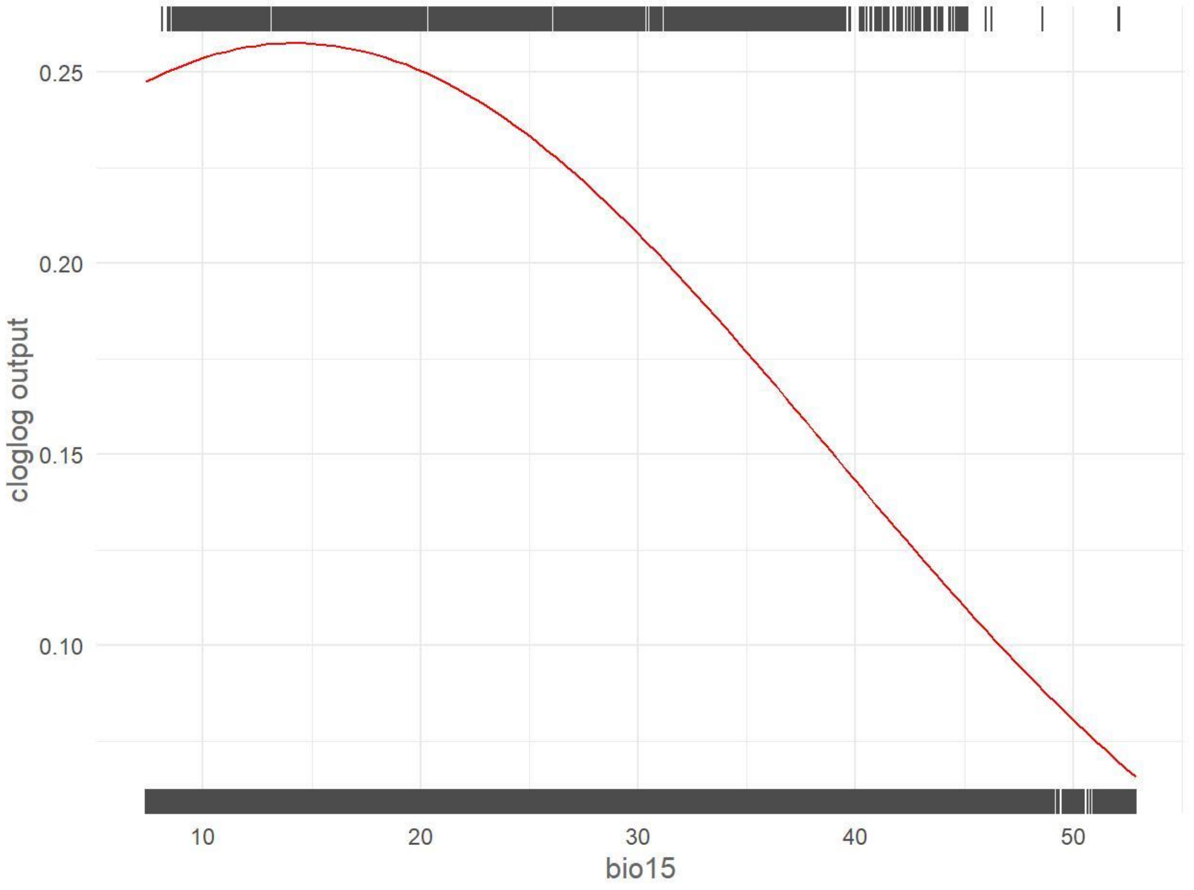

cloglog output
0.25
0.20
0.15
0.10
10
20
30
40
50
bio15

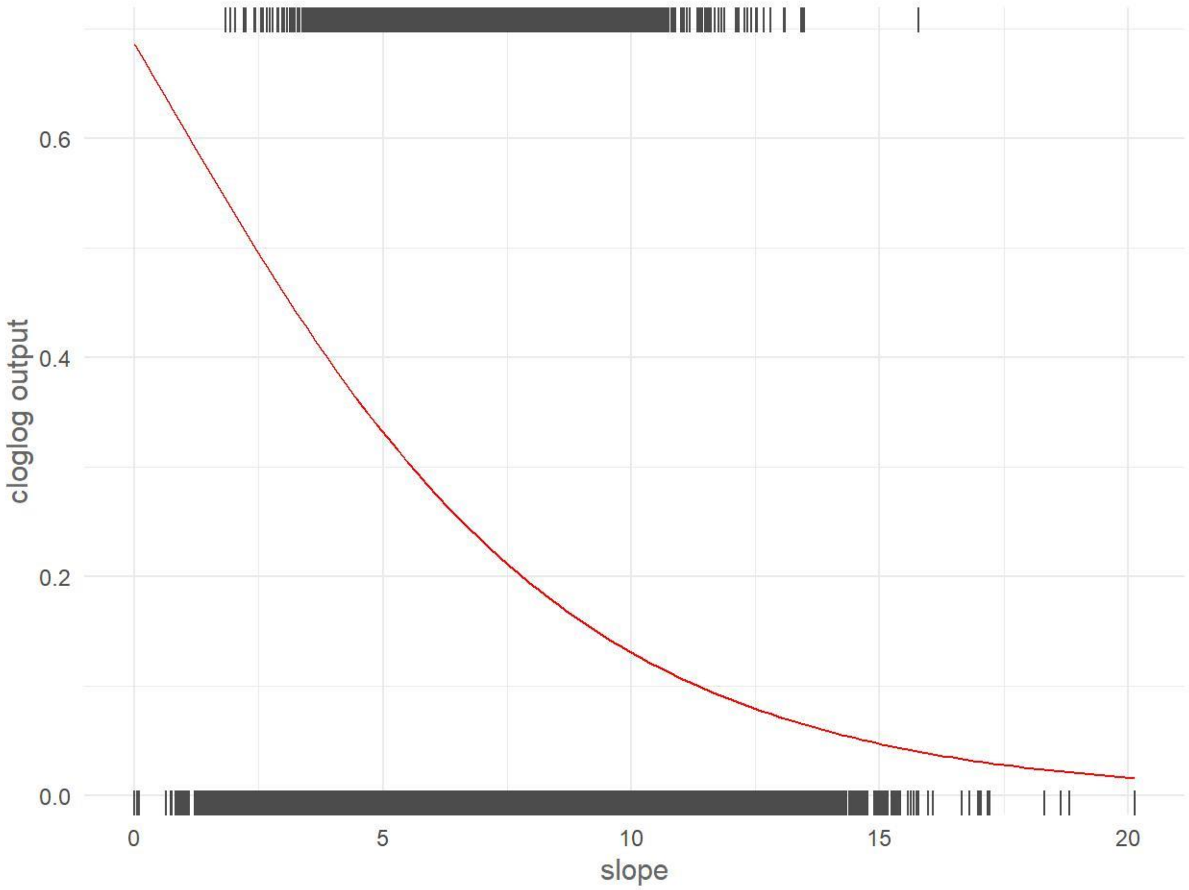

cloglog output
0.6
0.4
0.2
0.0
0
5
10
15
20
slope

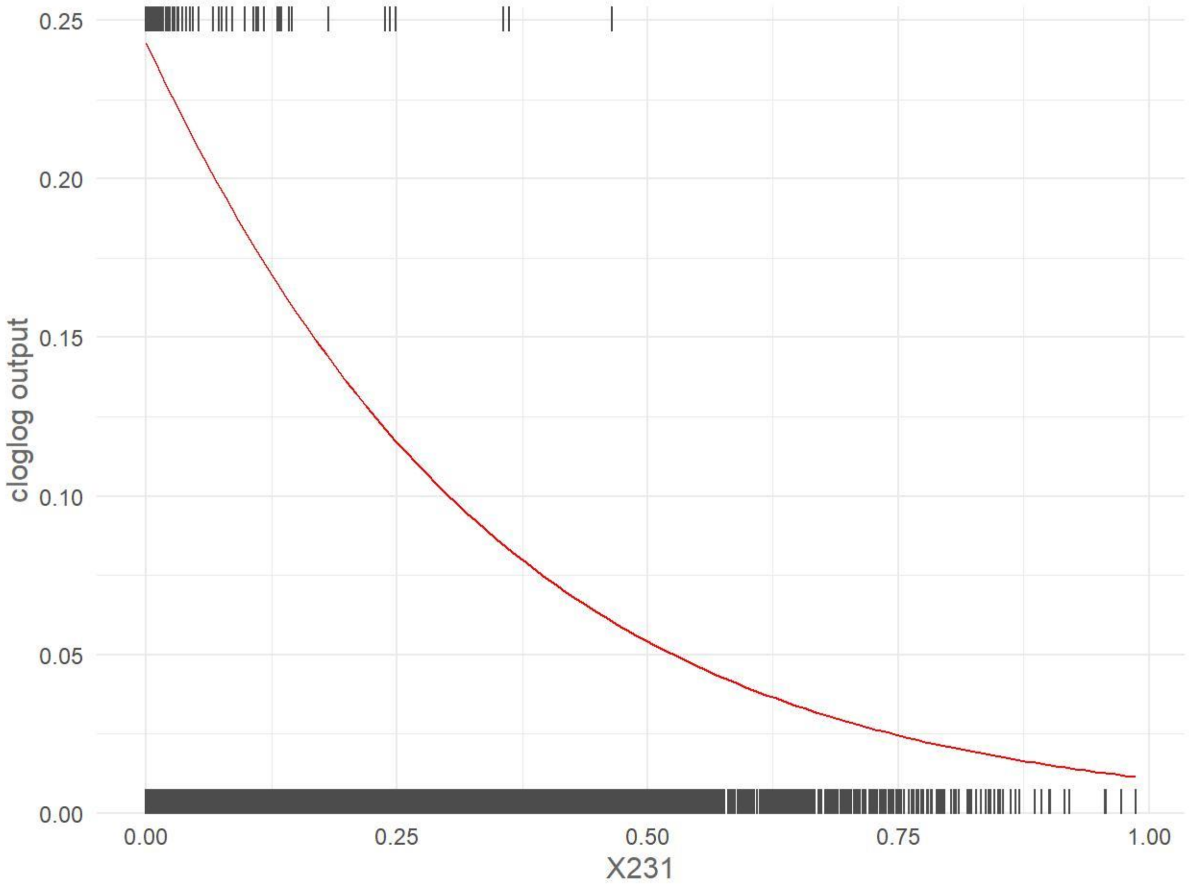

cloglog output
0.25
0.20
0.15
0.10
0.05
0.00
0.00
0.25
0.50
0.75
1.00
X231

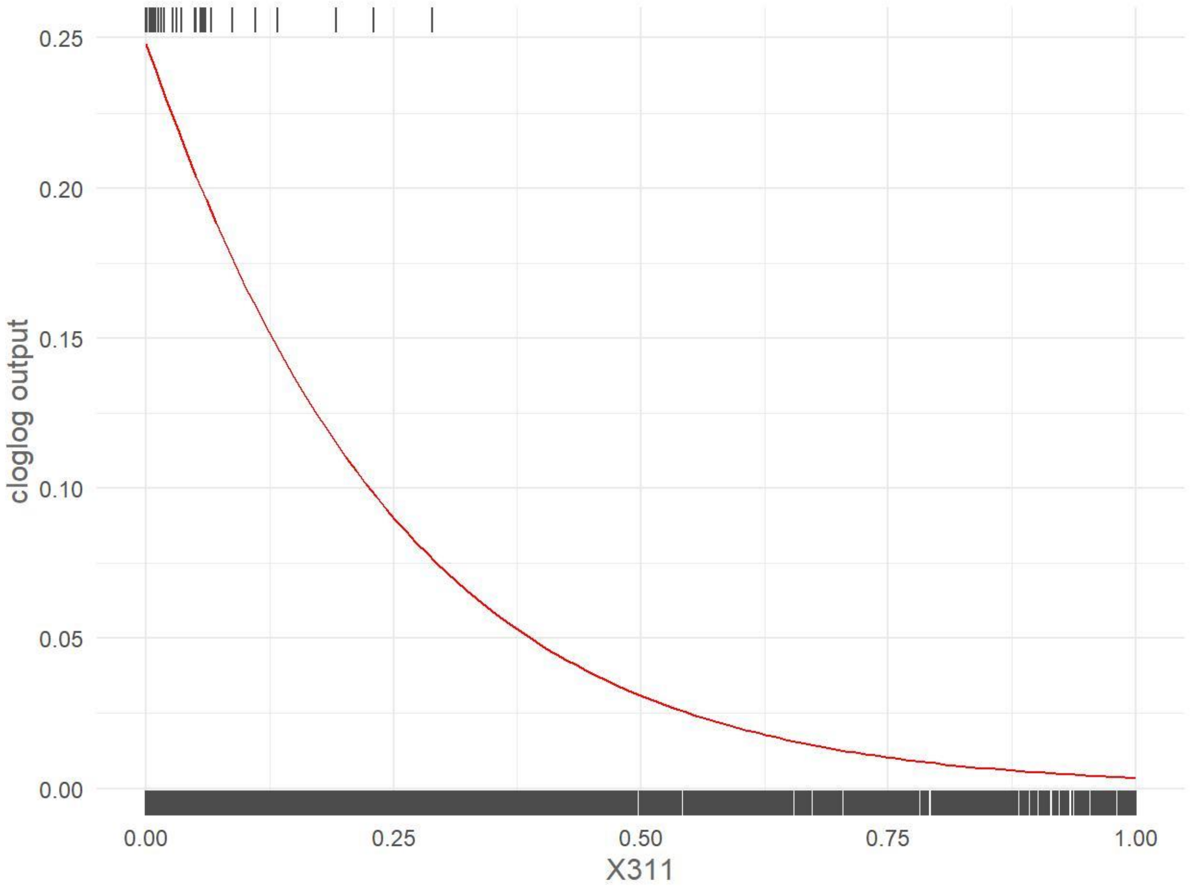

cloglog output
0.25
0.20
0.15
0.10
0.05
0.00
0.00
0.25
0.50
0.75
1.00
X311

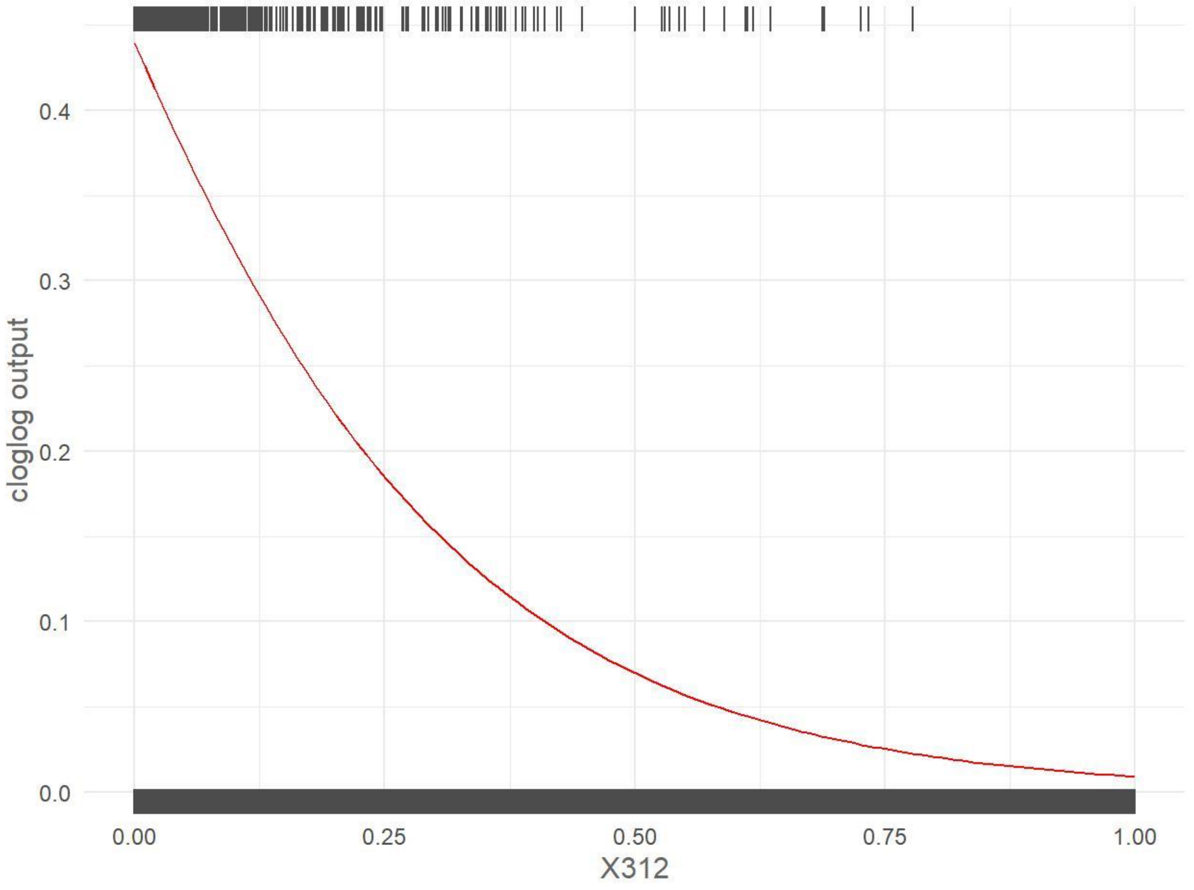

cloglog output
0.4
0.3
0.2
0.1
0.0
0.00
0.25
0.50
0.75
1.00
X312

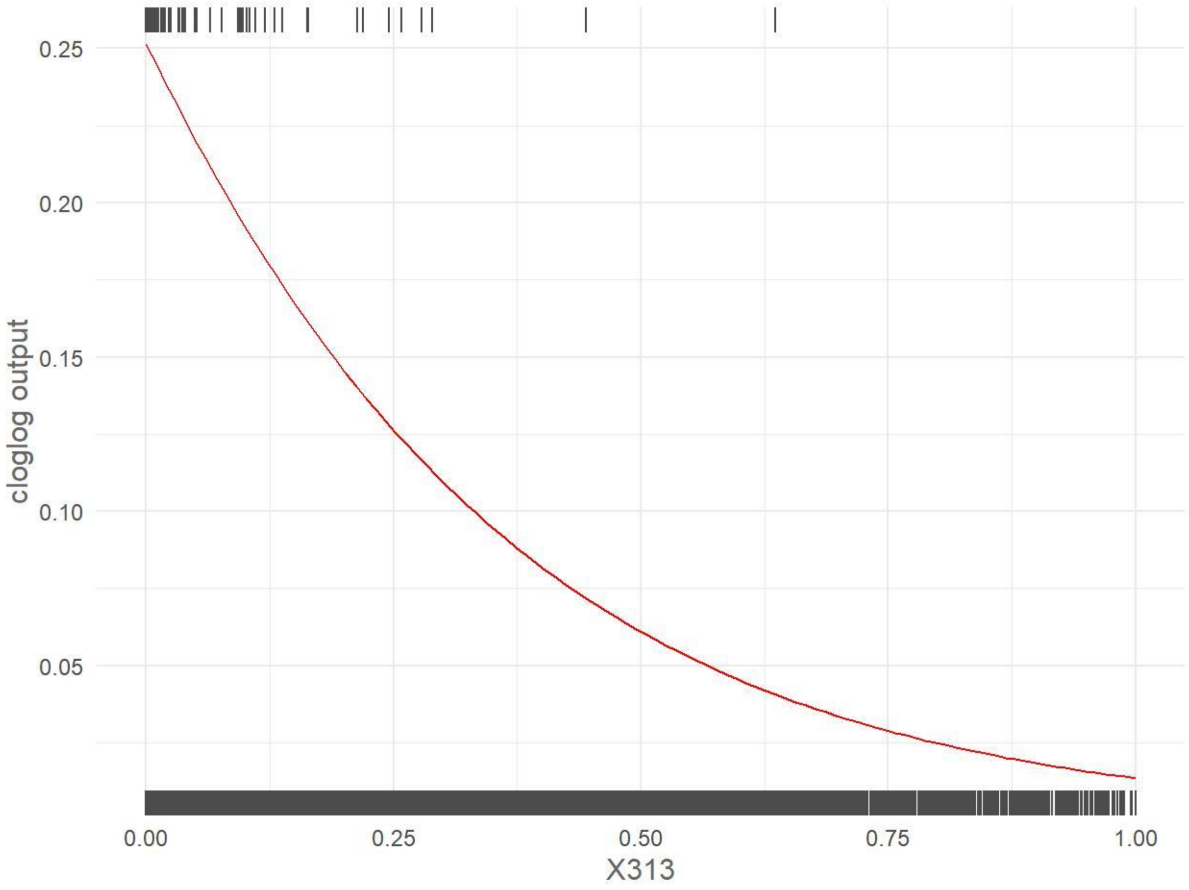
cloglog output
0.25
0.20
0.15
0.10
0.05
0.00
0.25
0.50
0.75
1.00
X313

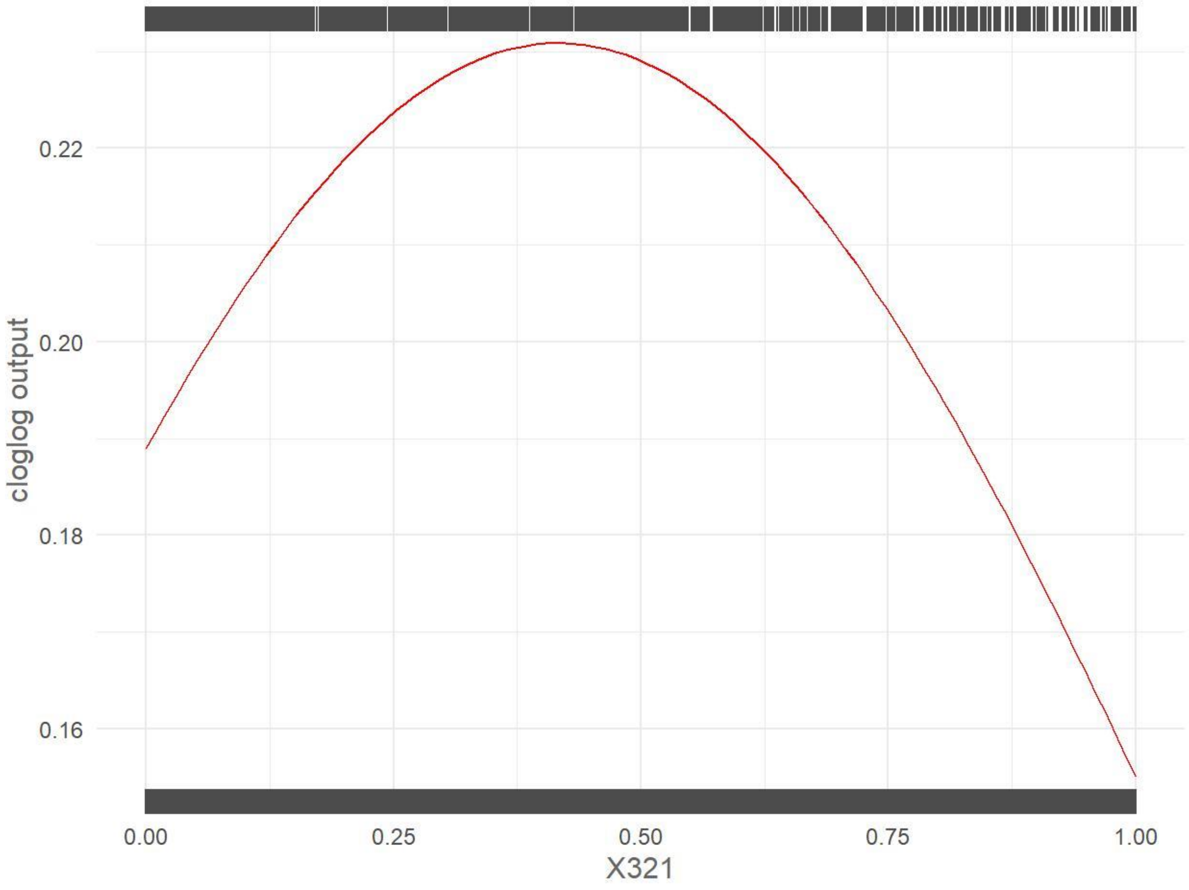
cloglog output
0.22
0.20
0.18
0.16
0.00
0.25
0.50
0.75
1.00
X321

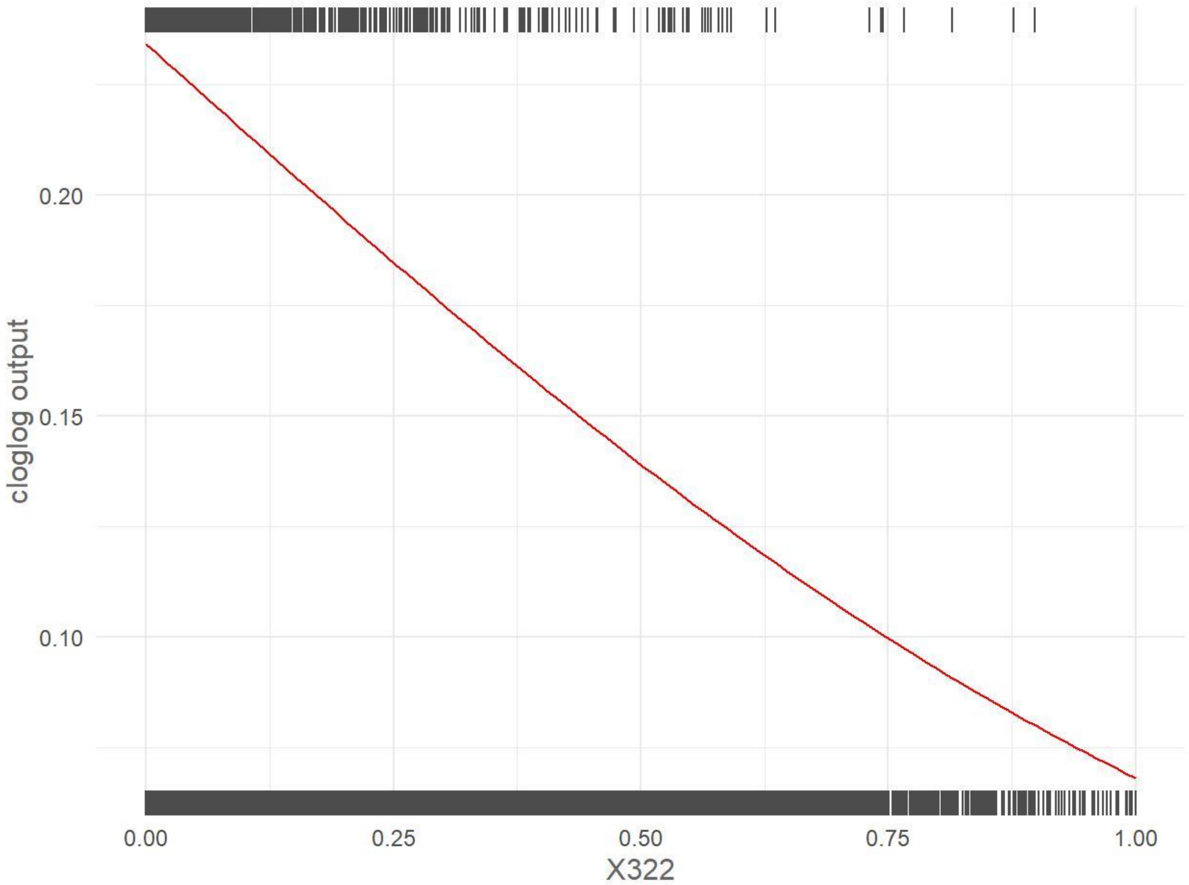
0.20
0.15
0.10
cloglog output
0.00
0.25
0.50
0.75
1.00
X322

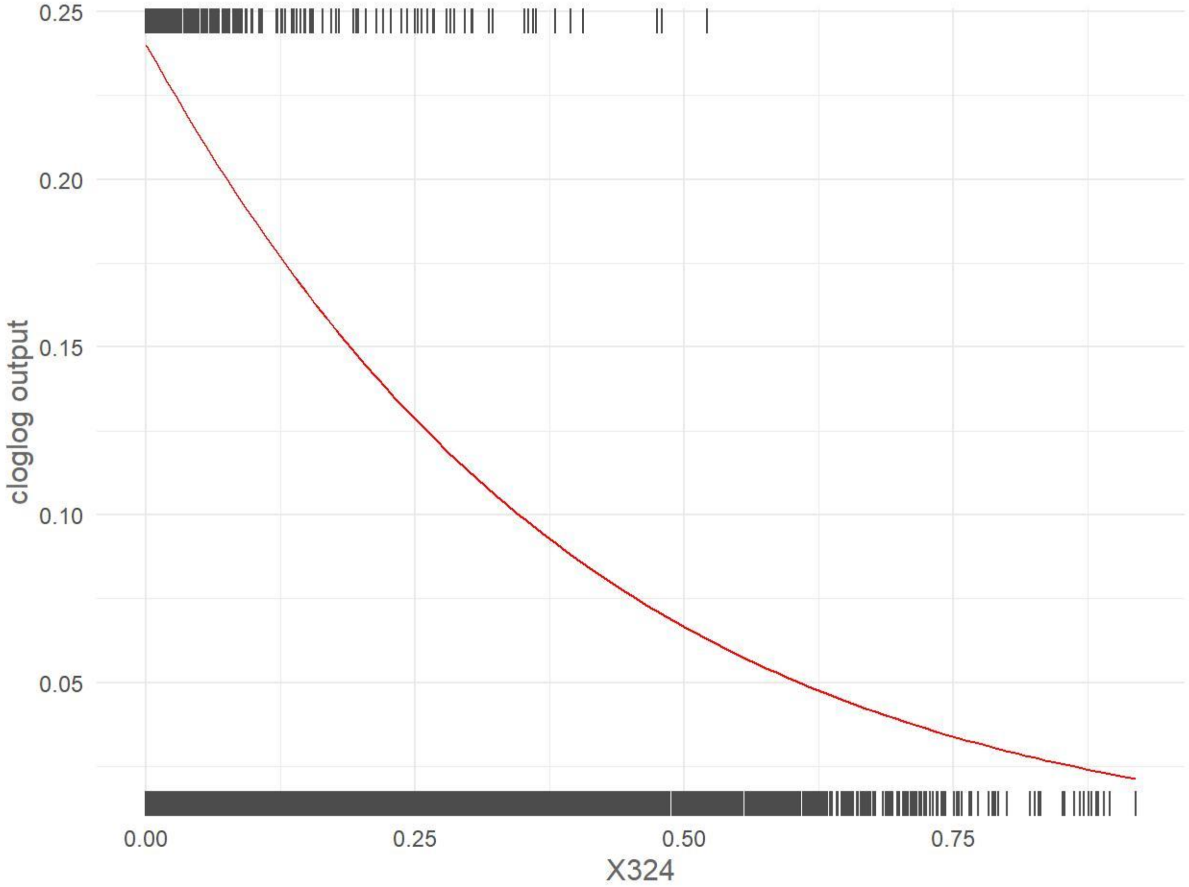
0.25
0.20
0.15
0.10
0.05
cloglog output
0.00
0.25
0.50
0.75
X324

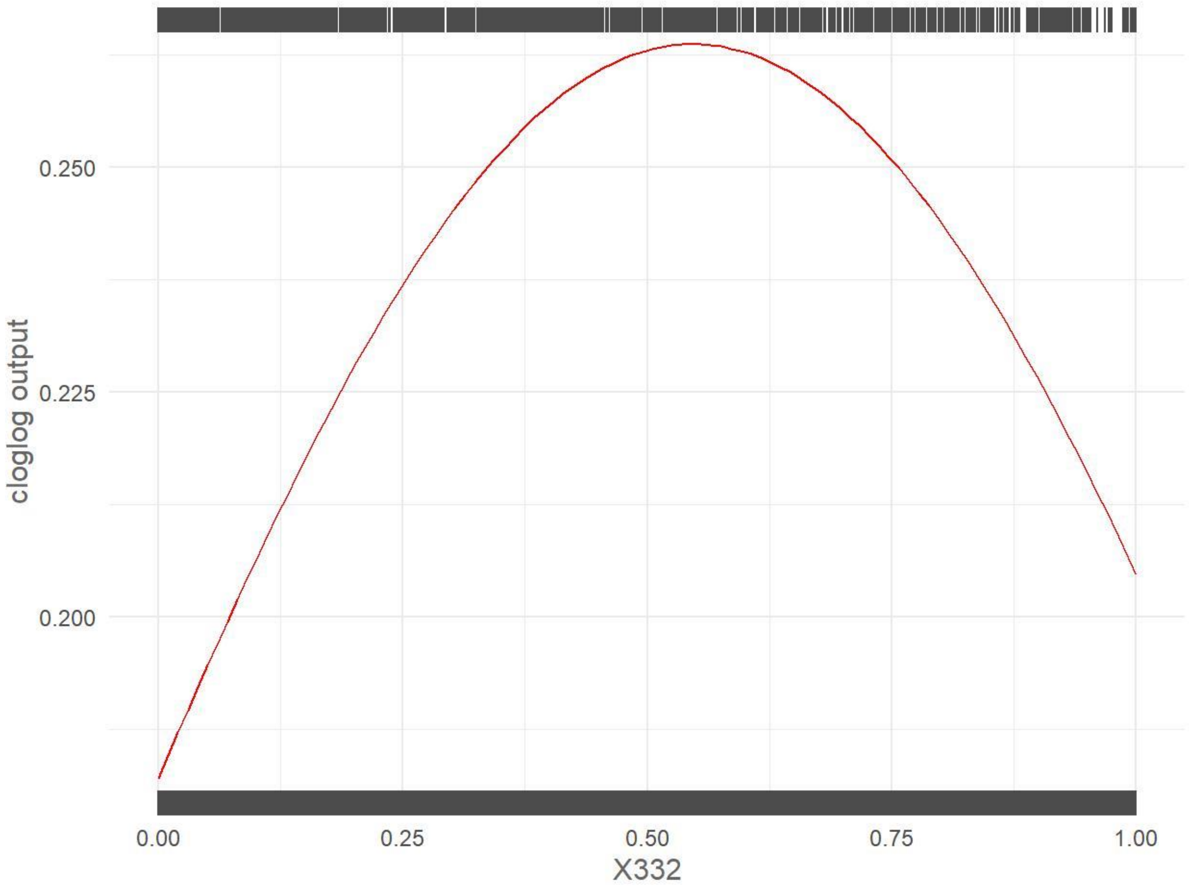
cloglog output
0.250
0.225
0.200
0.00
0.25
0.50
0.75
1.00
X332

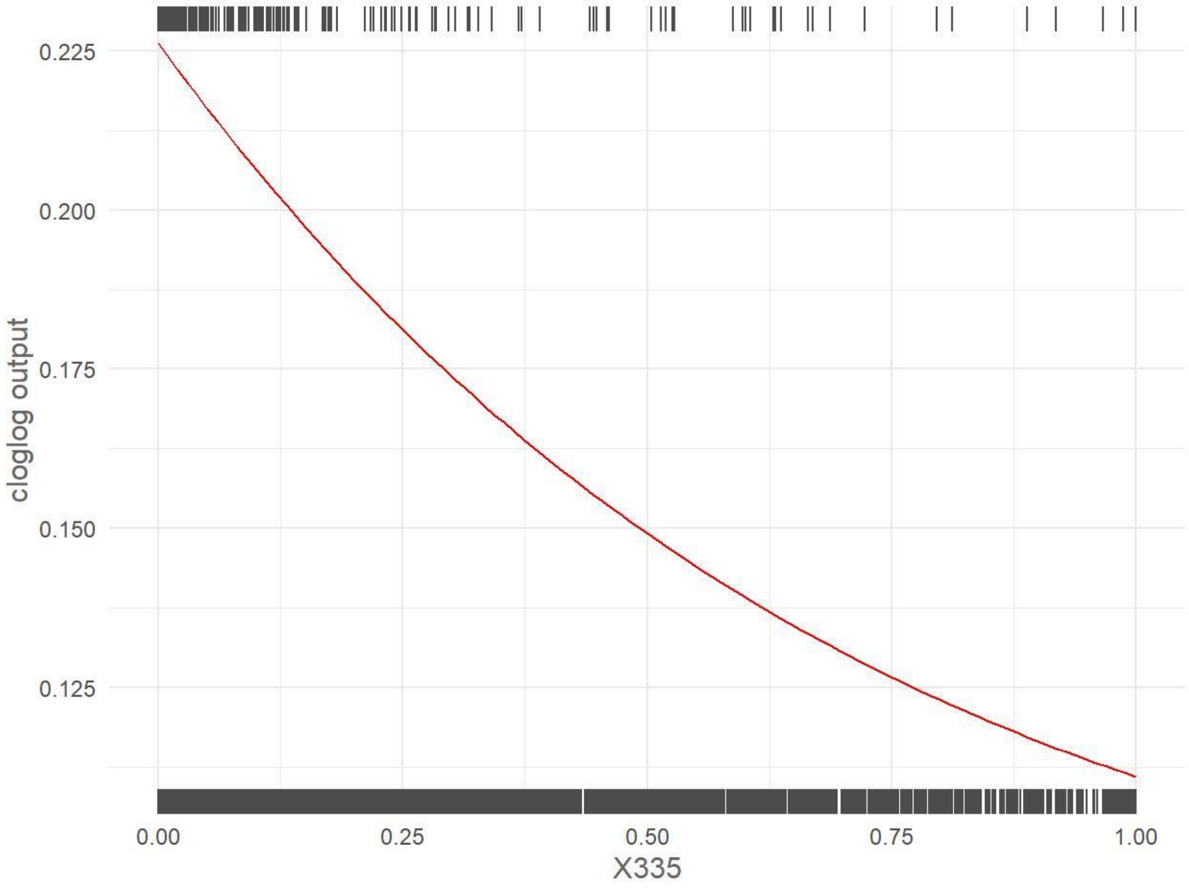
cloglog output
0.225
0.200
0.175
0.150
0.125
0.00
0.25
0.50
0.75
1.00
X335

Figure S5. Species-habitat relationships according to the Artificial Neural Network model for rock ptarmigan.

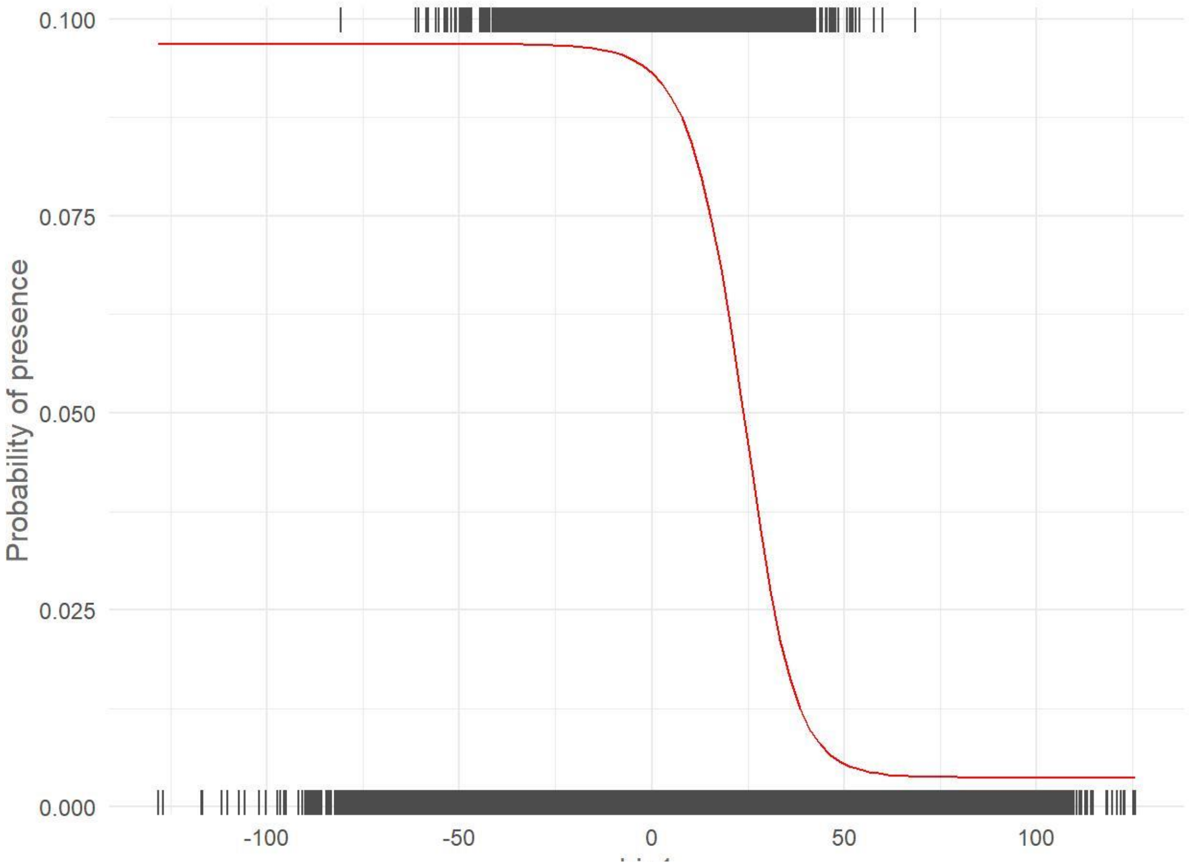
Probability of presence
0.100
0.075
0.050
0.025
0.000
-100
-50
0
50
100

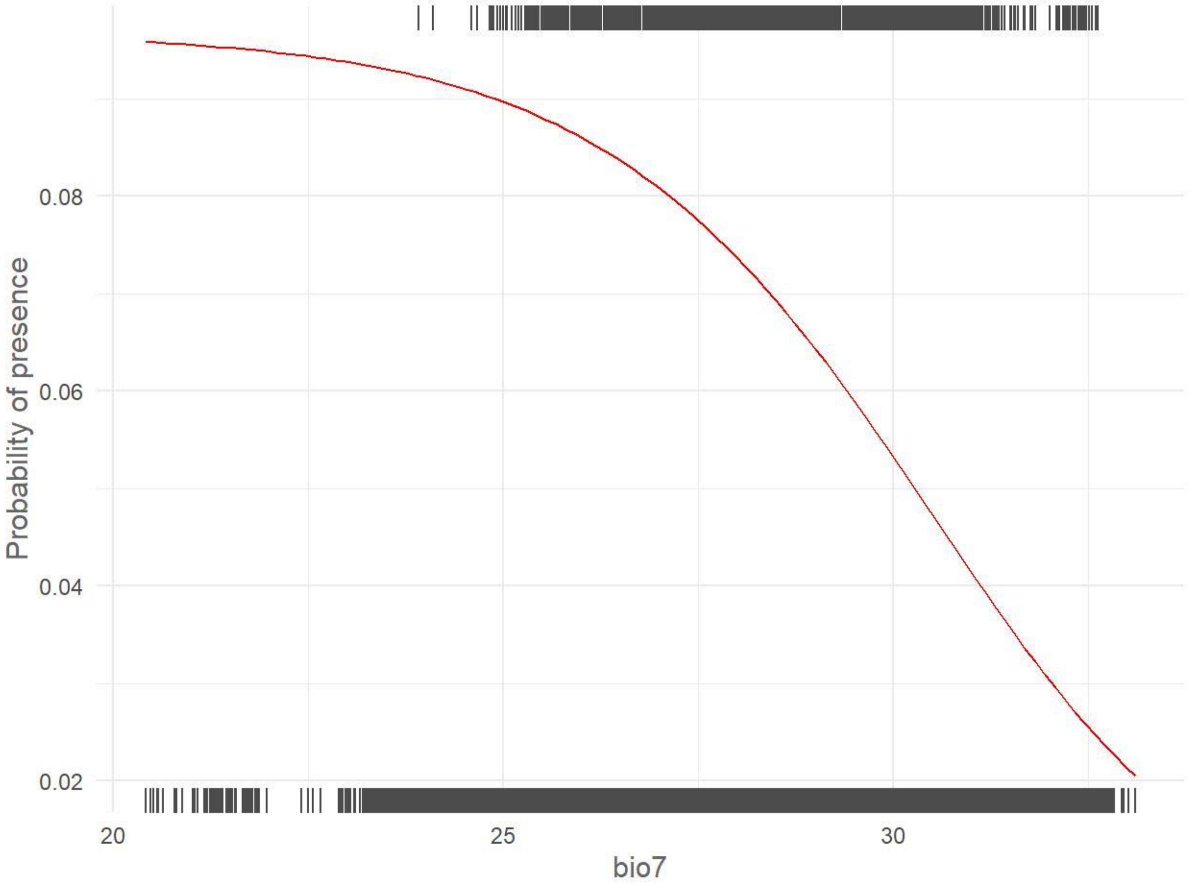
Probability of presence
0.08
0.06
0.04
0.02
20
25
30
bio7

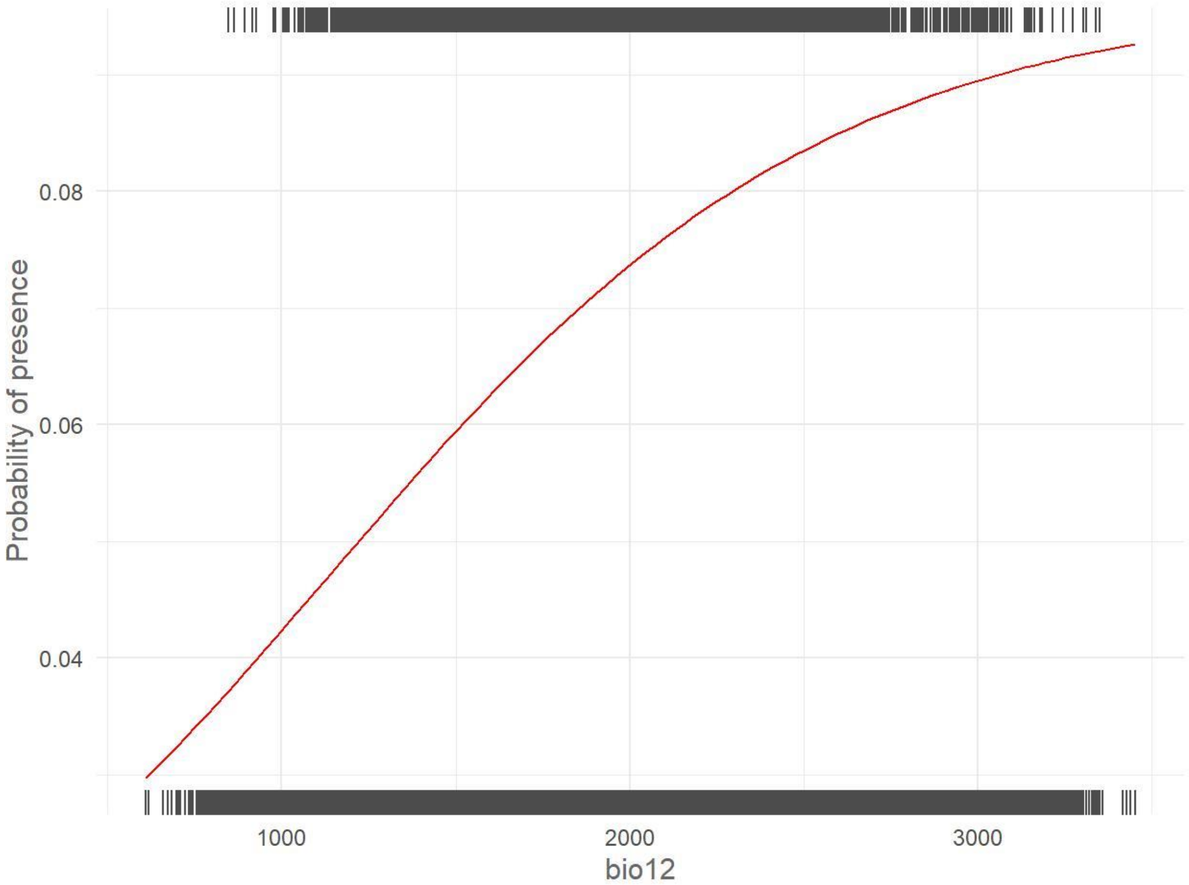
0.08
0.06
0.04
Probability of presence
1000
2000
3000
bio12

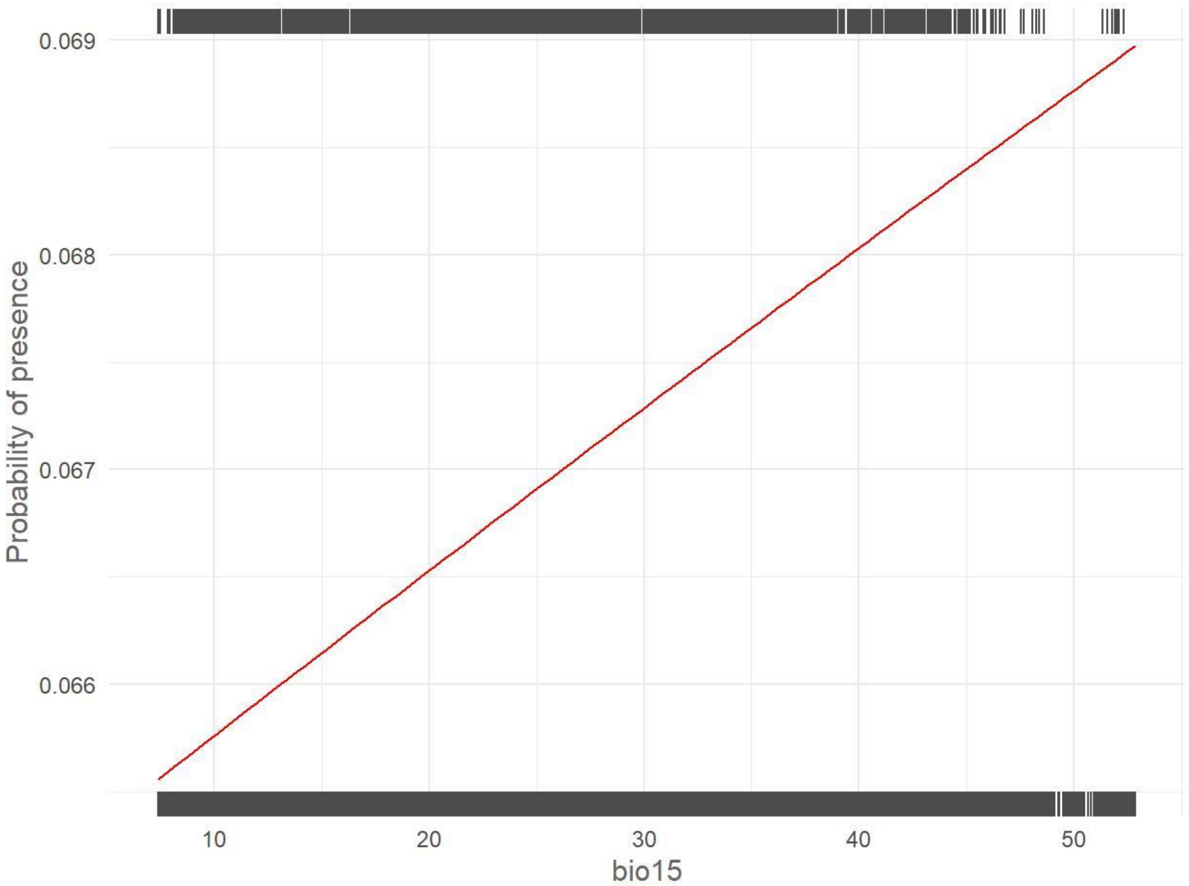
0.069
0.068
0.067
0.066
Probability of presence
10
20
30
40
50
bio15

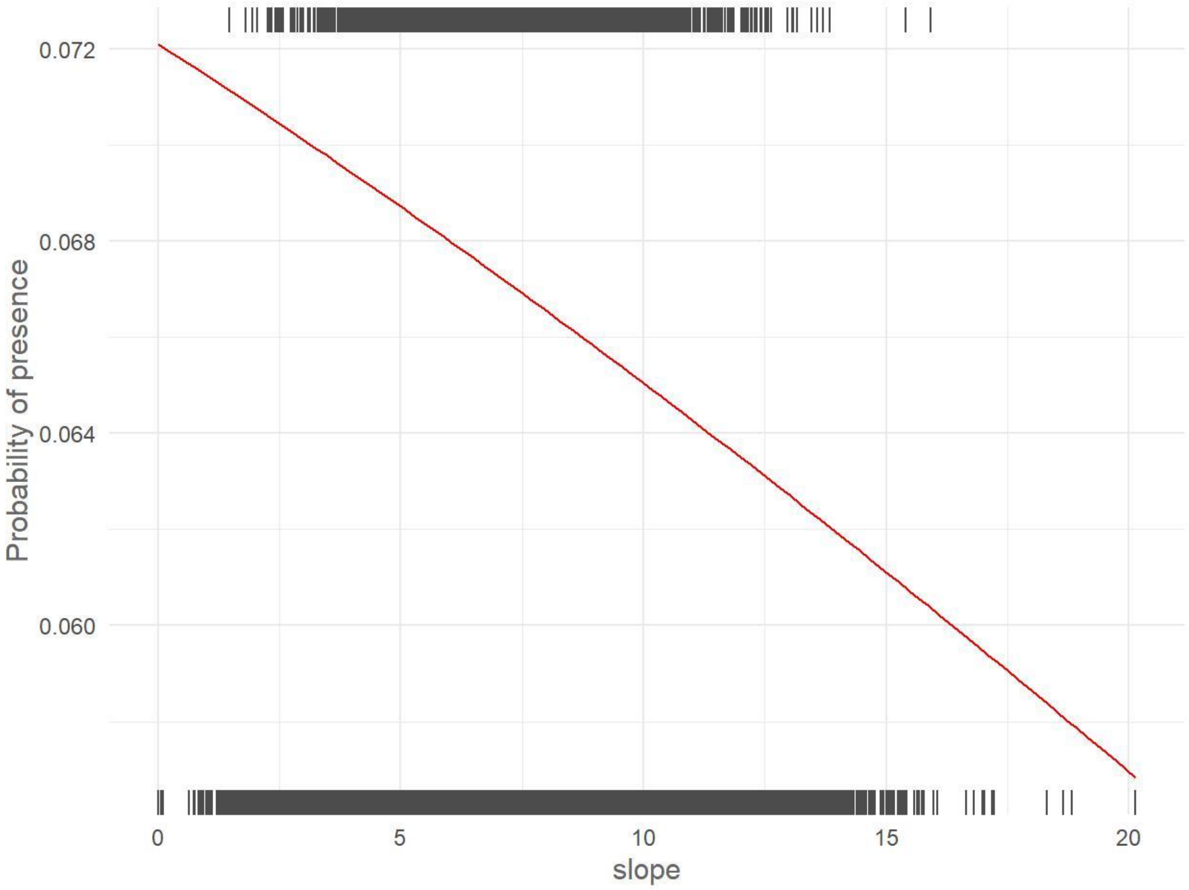
0.072
0.068
0.064
0.060
Probability of presence
0
5
10
15
20
slope

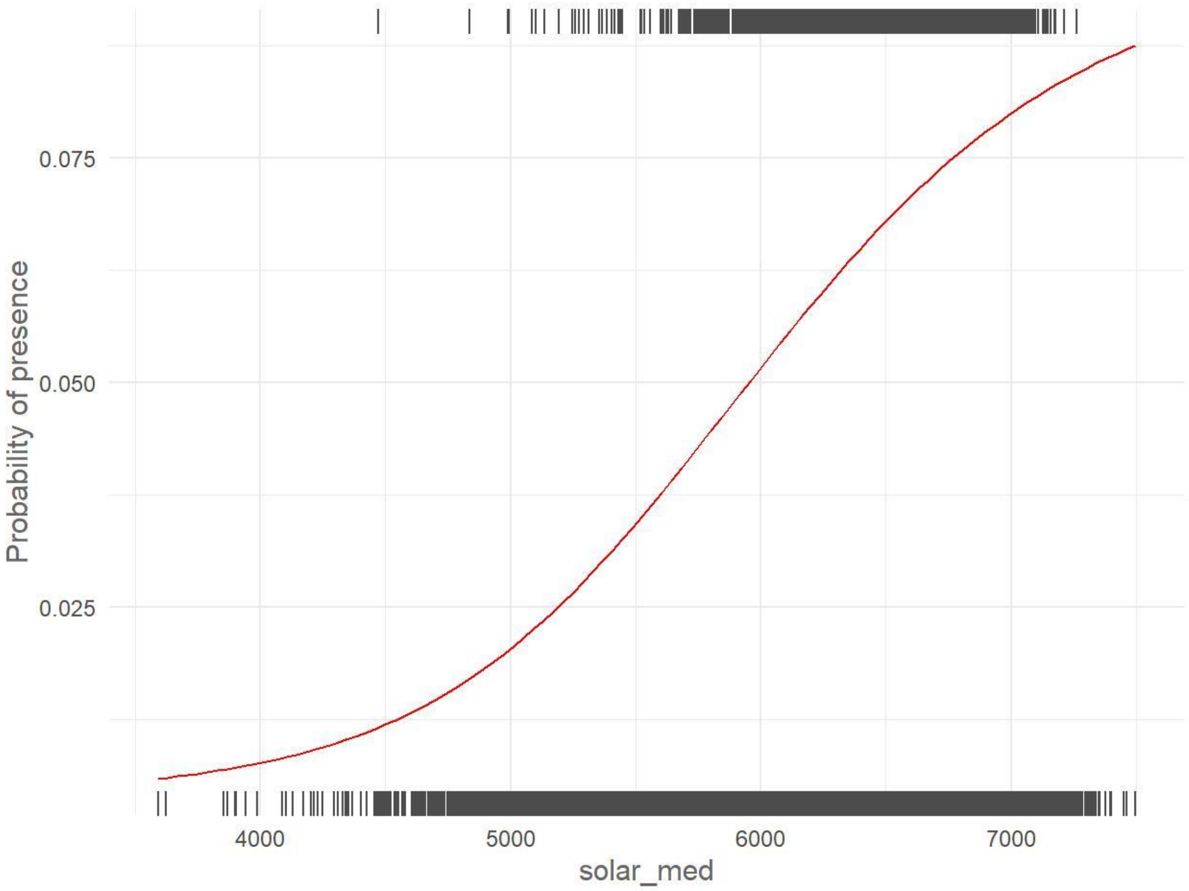
0.075
0.050
0.025
Probability of presence
4000
5000
6000
7000
solar_med

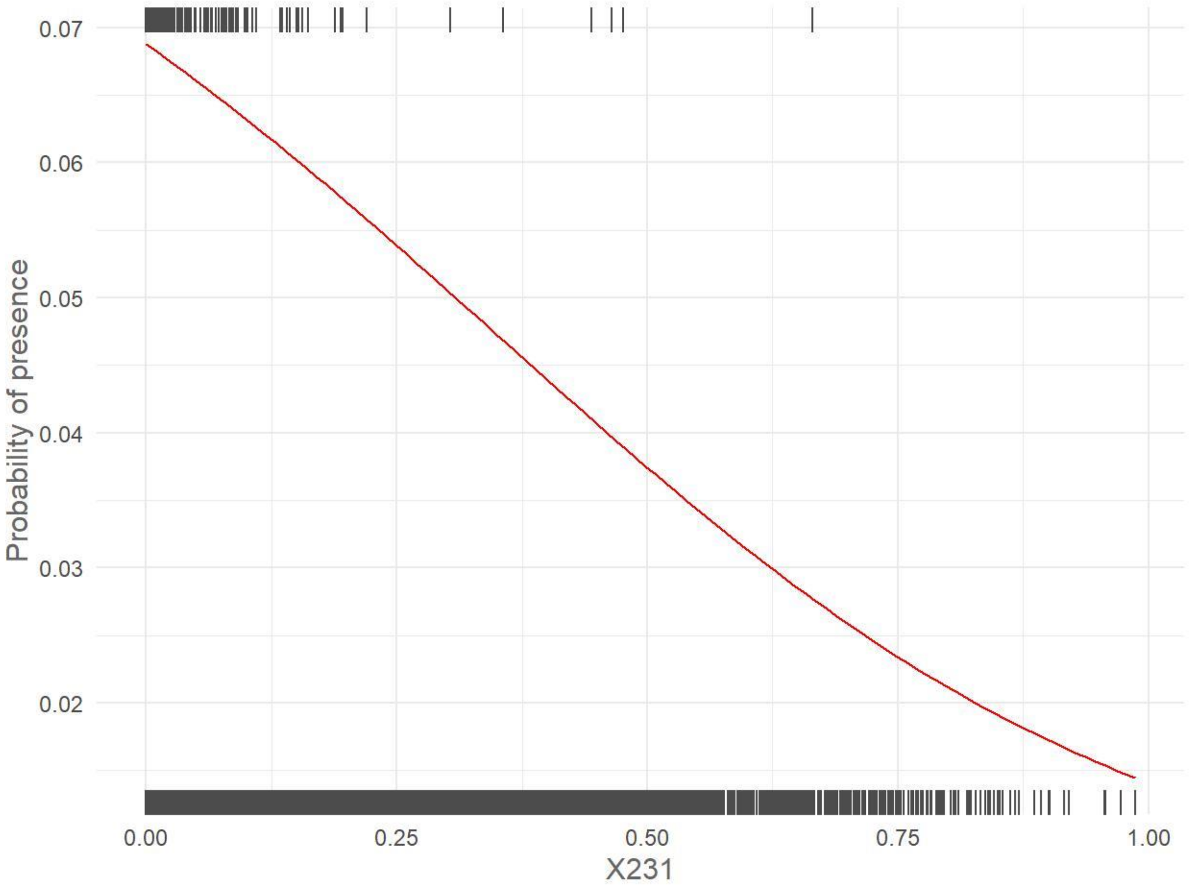
0.07
0.06
0.05
0.04
0.03
0.02
Probability of presence
0.00
0.25
0.50
0.75
1.00
X231

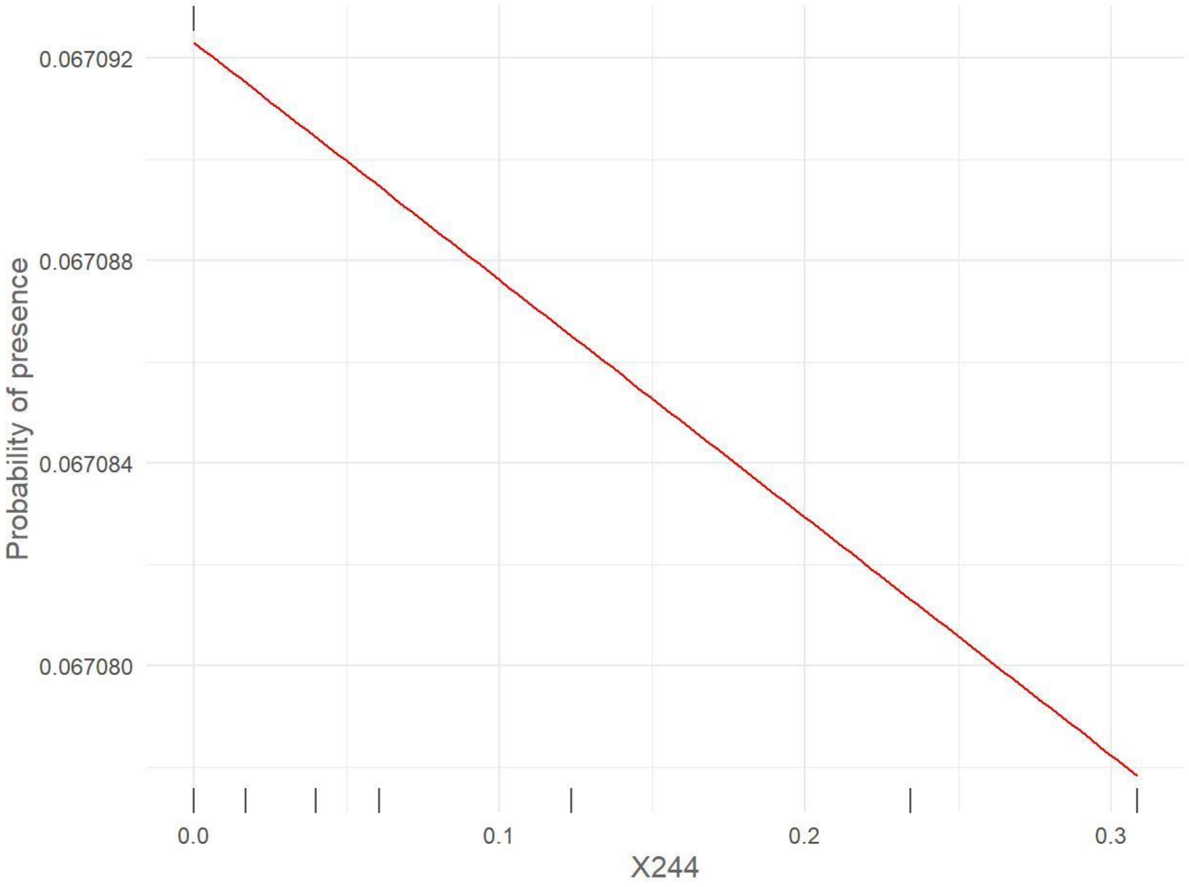
0.067092
0.067088
0.067084
0.067080
Probability of presence
0.0
0.1
0.2
0.3
X244

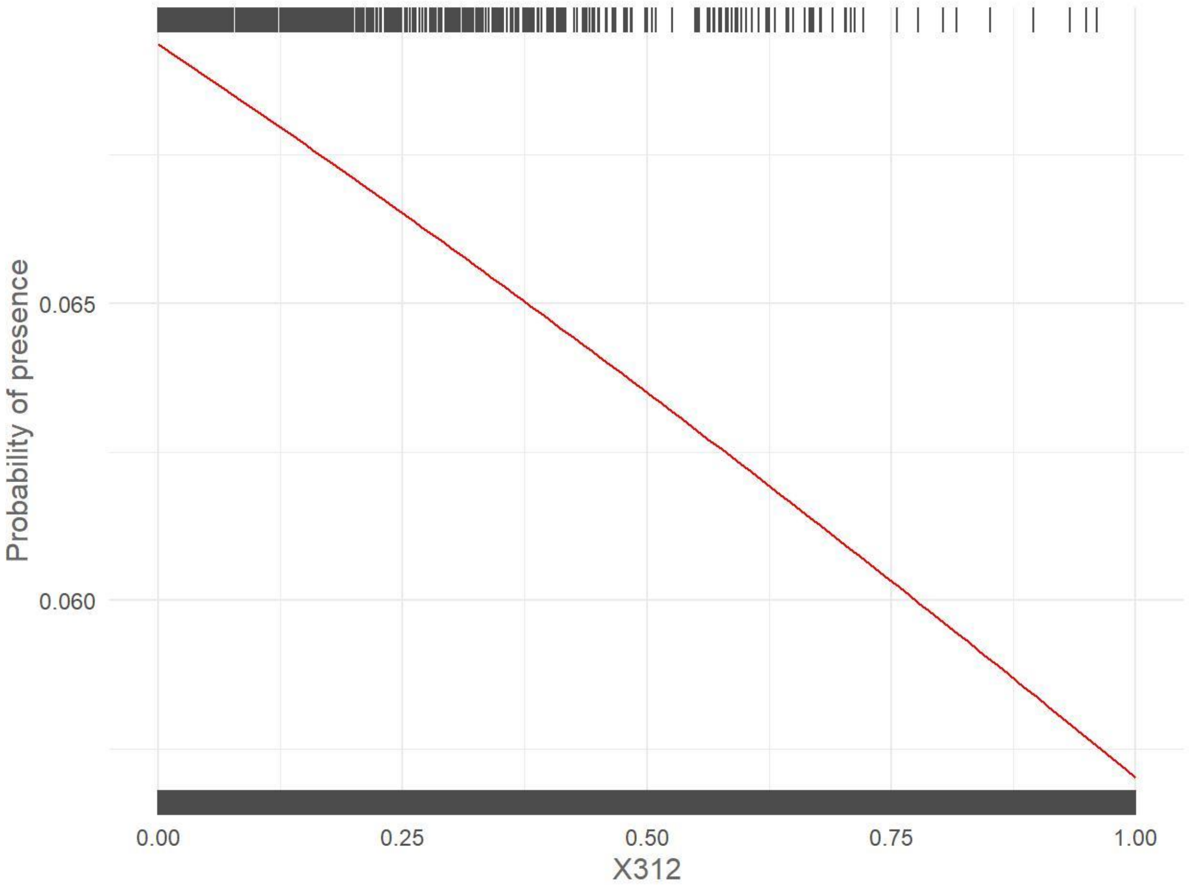

Probability of presence
0.065
0.060
0.00
0.25
0.50
0.75
1.00
X312

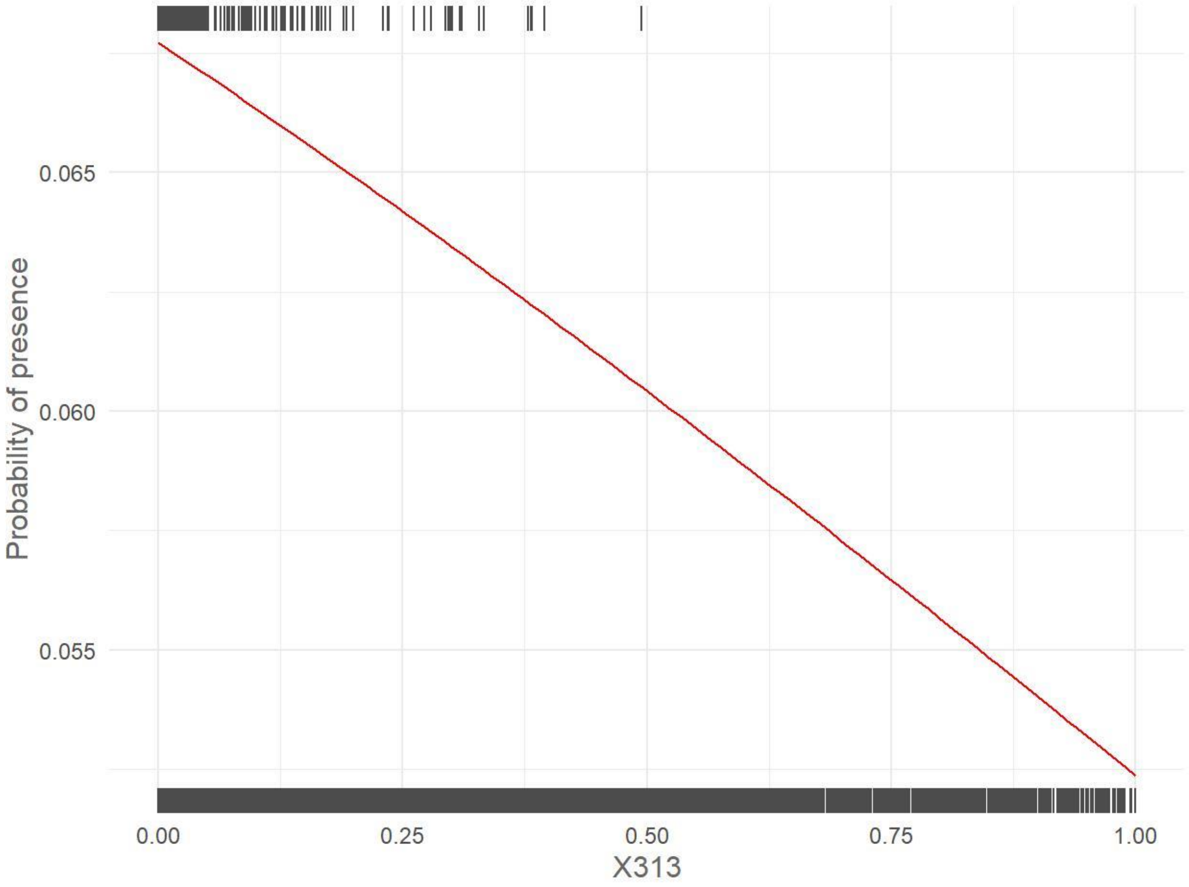

Probability of presence
0.065
0.060
0.055
0.00
0.25
0.50
0.75
1.00
X313

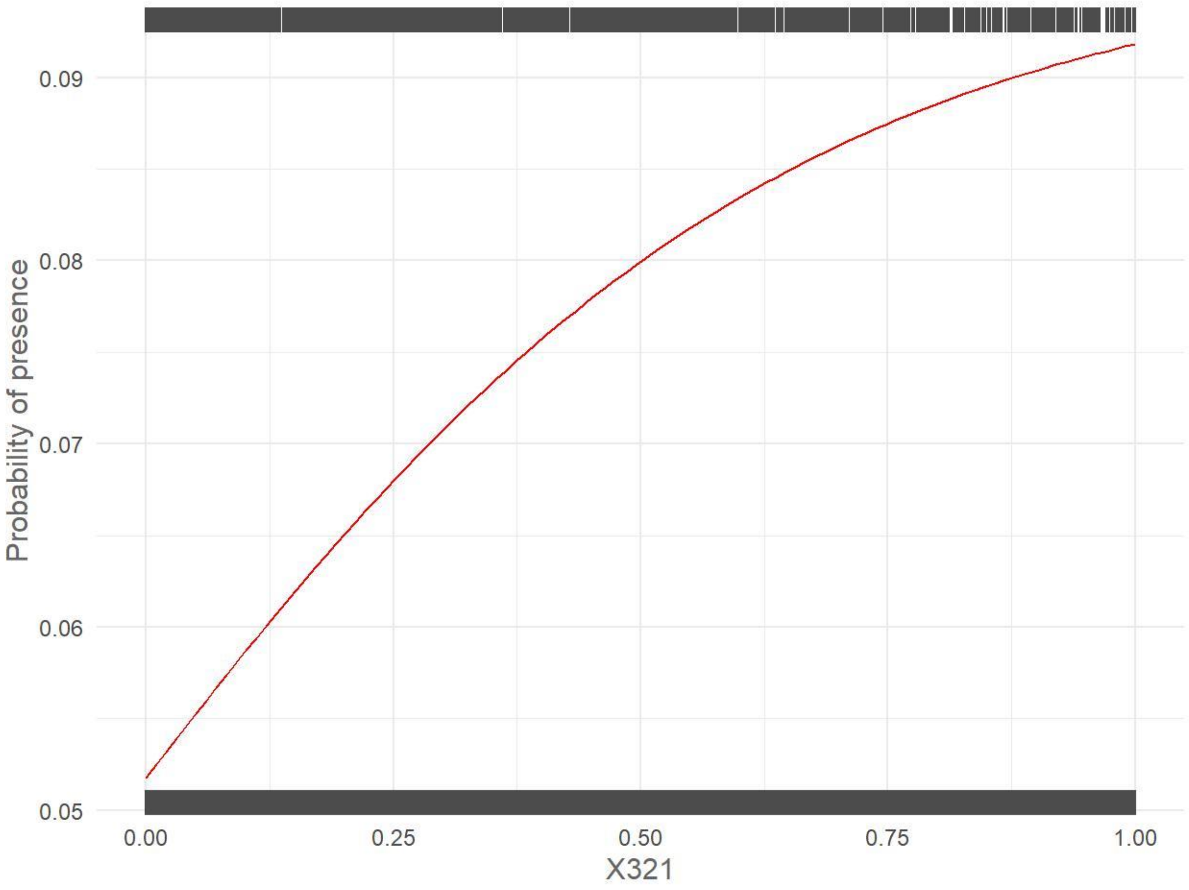

0.09
0.08
0.07
0.06
0.05
Probability of presence
0.00
0.25
0.50
0.75
1.00
X321

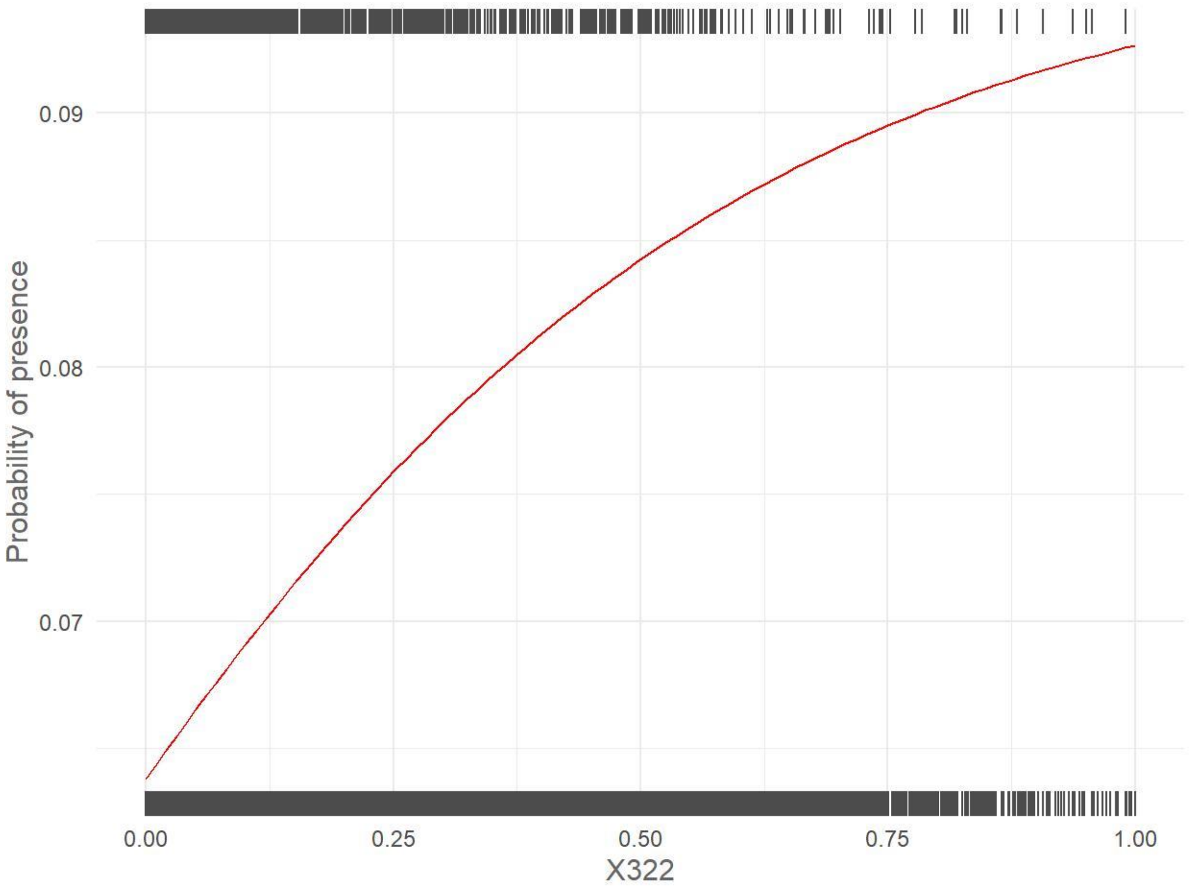

0.09
0.08
0.07
Probability of presence
0.00
0.25
0.50
0.75
1.00
X322

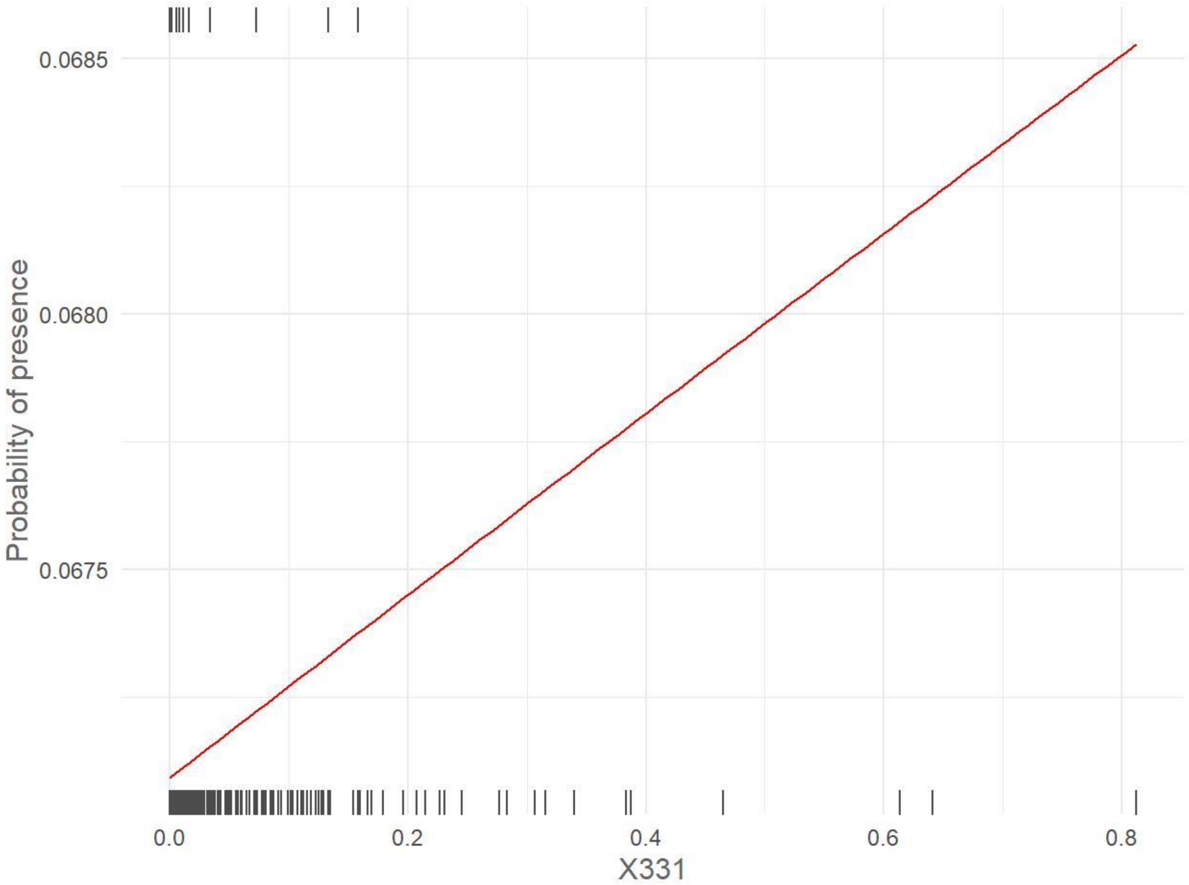

0.0685
0.0680
0.0675
Probability of presence
0.0
0.2
0.4
0.6
0.8
X331

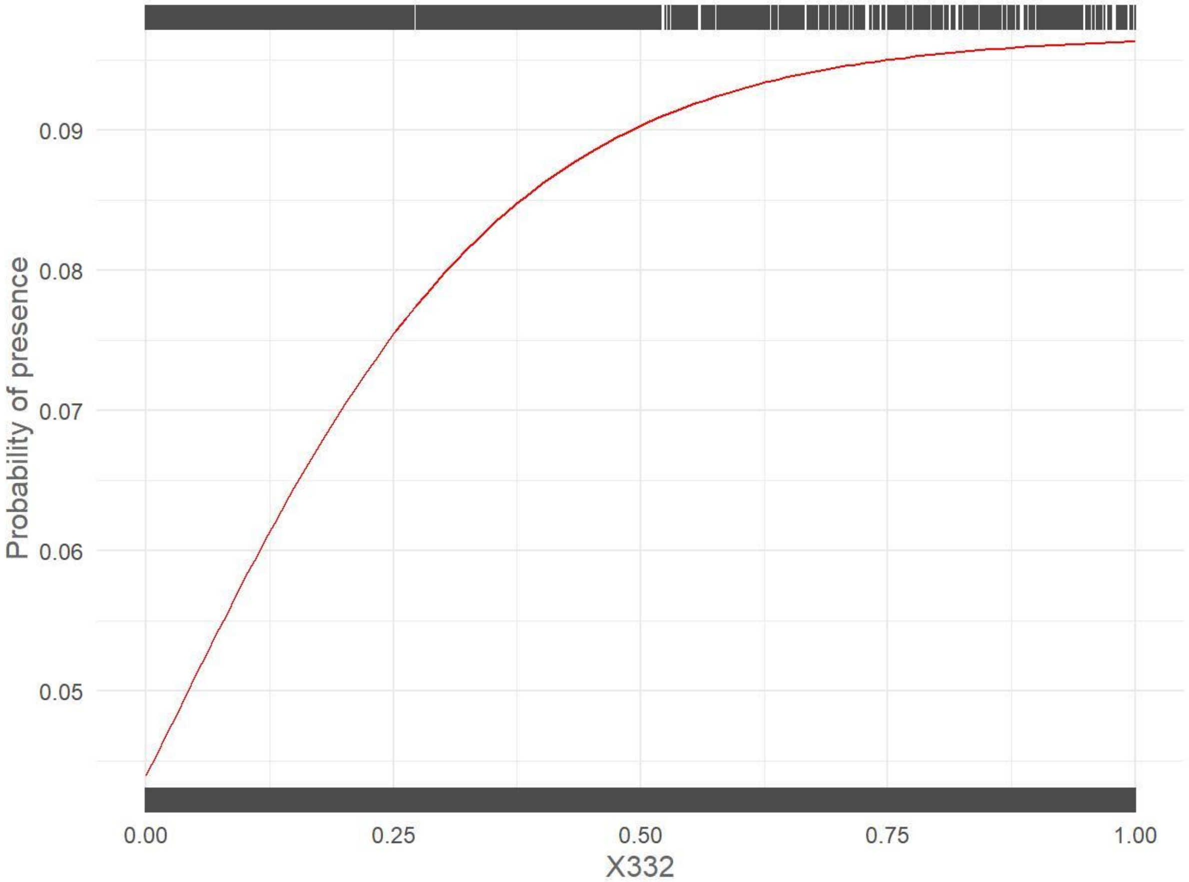

0.09
0.08
0.07
0.06
0.05
Probability of presence
0.00
0.25
0.50
0.75
1.00
X332

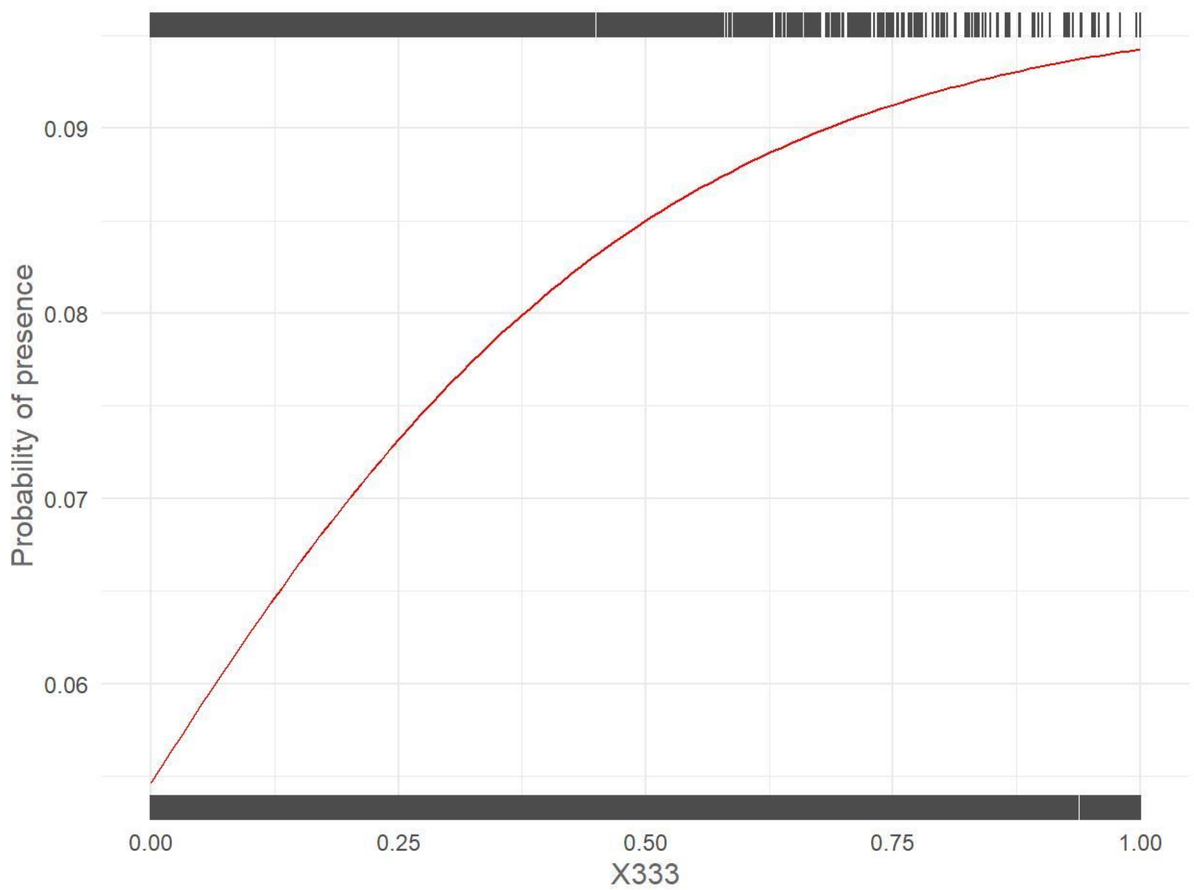
0.09
0.08
0.07
0.06
Probability of presence
0.00
0.25
0.50
0.75
1.00
X333
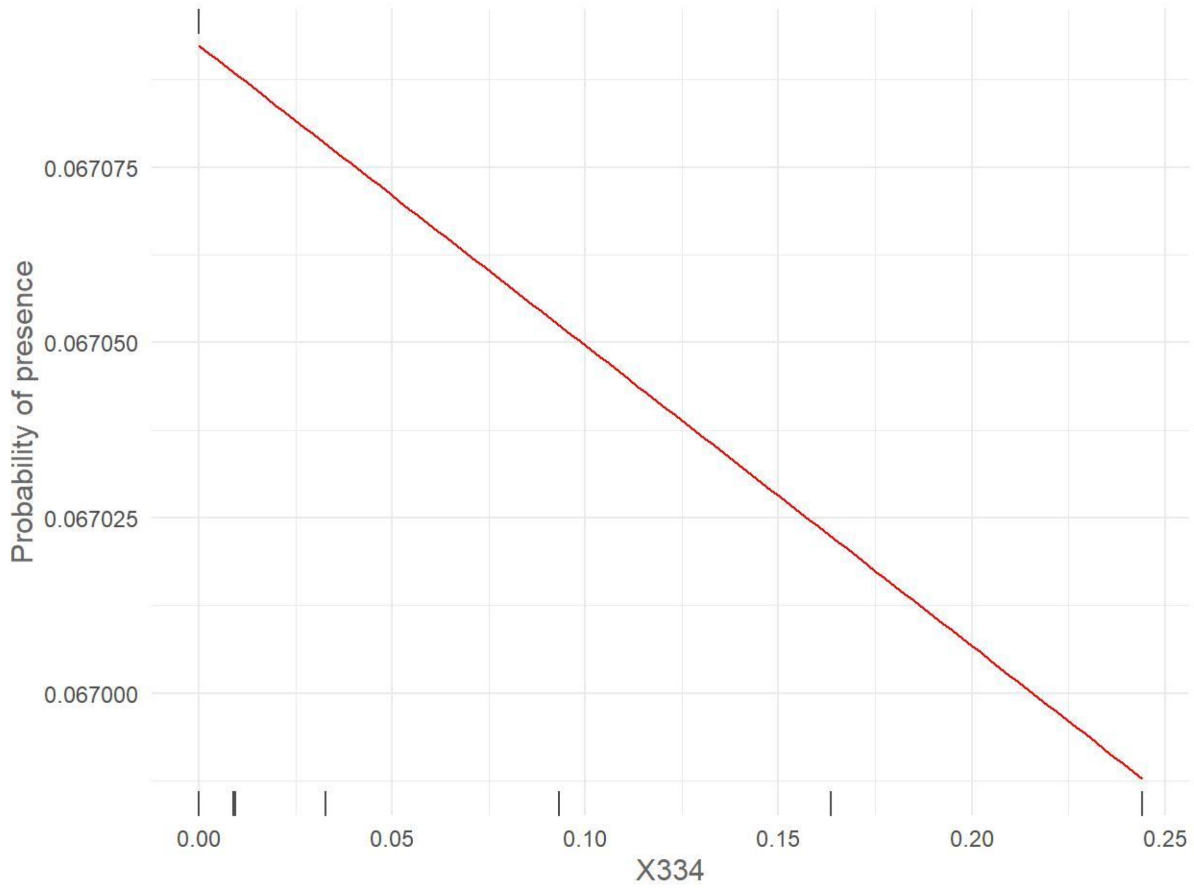
0.067075
0.067050
0.067025
0.067000
Probability of presence
0.00
0.05
0.10
0.15
0.20
0.25
X334

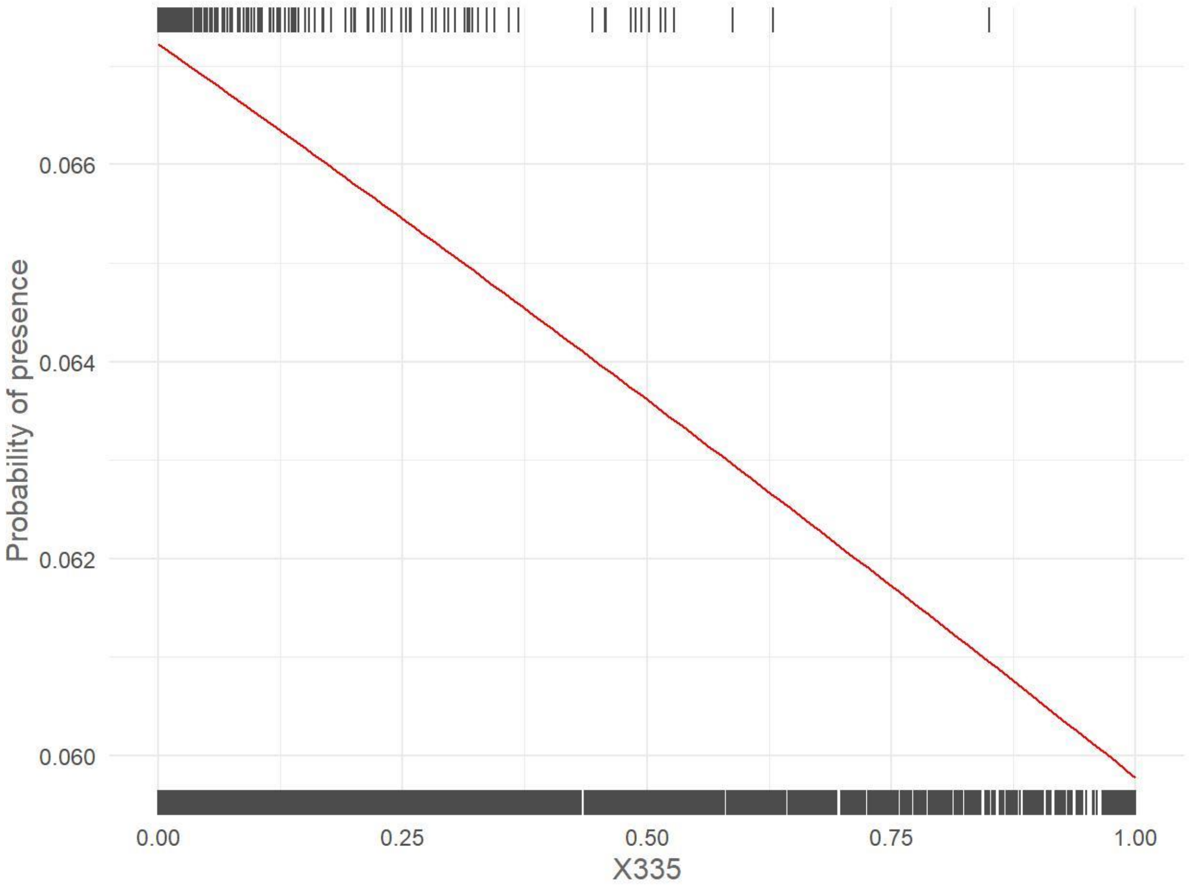

Probability of presence
0.066
0.064
0.062
0.060
0.00
0.25
0.50
0.75
1.00
X335

Figure S6. Species-habitat relationships according to the Artificial Neural Network model for water pipit.

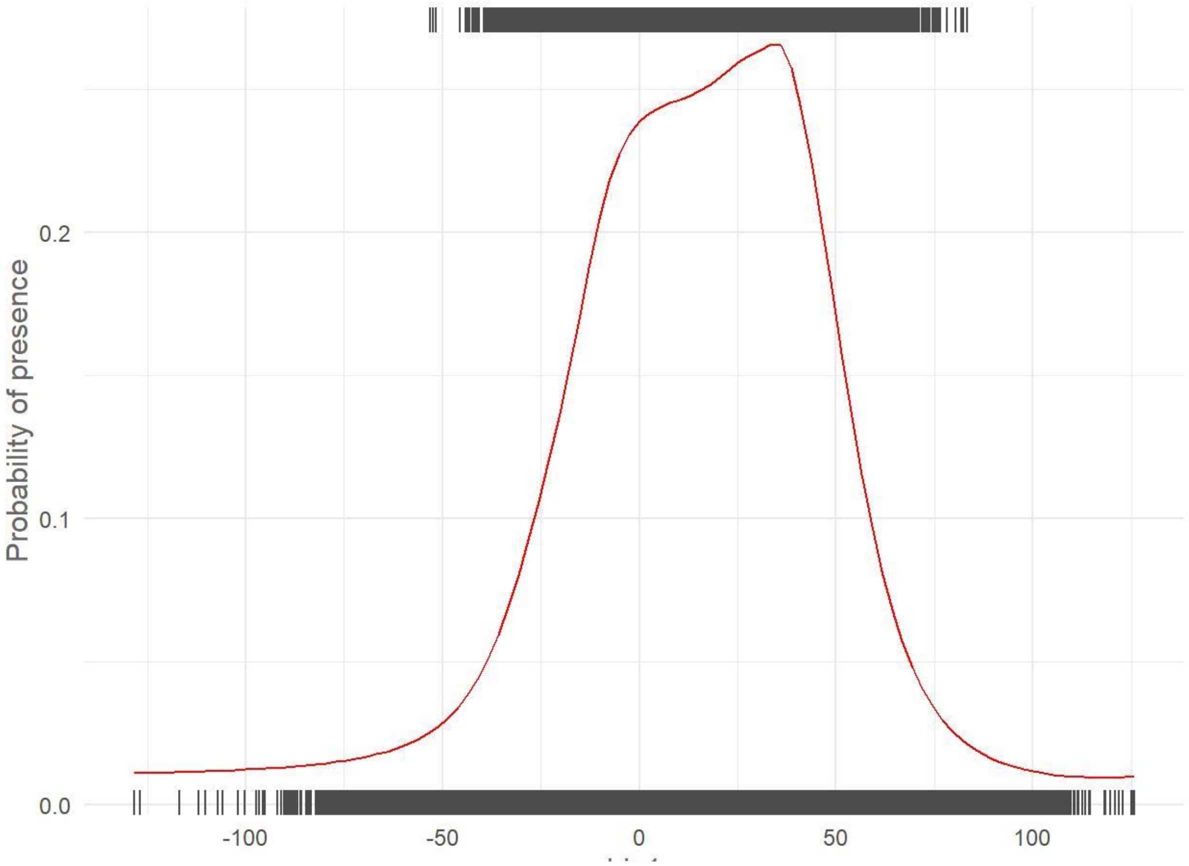
Probability of presence
0.0
0.1
0.2
-100
-50
0
50
100

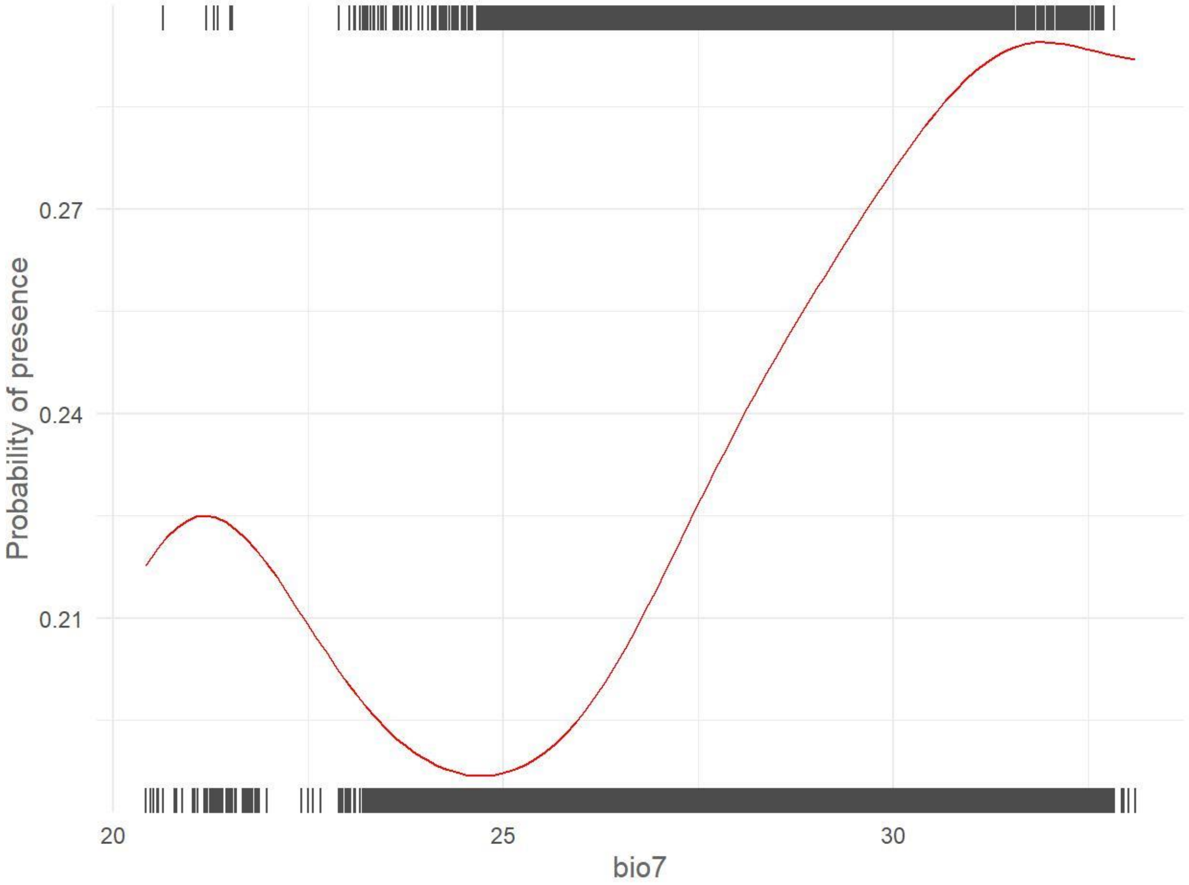
Probability of presence
0.21
0.24
0.27
20
25
30
bio7

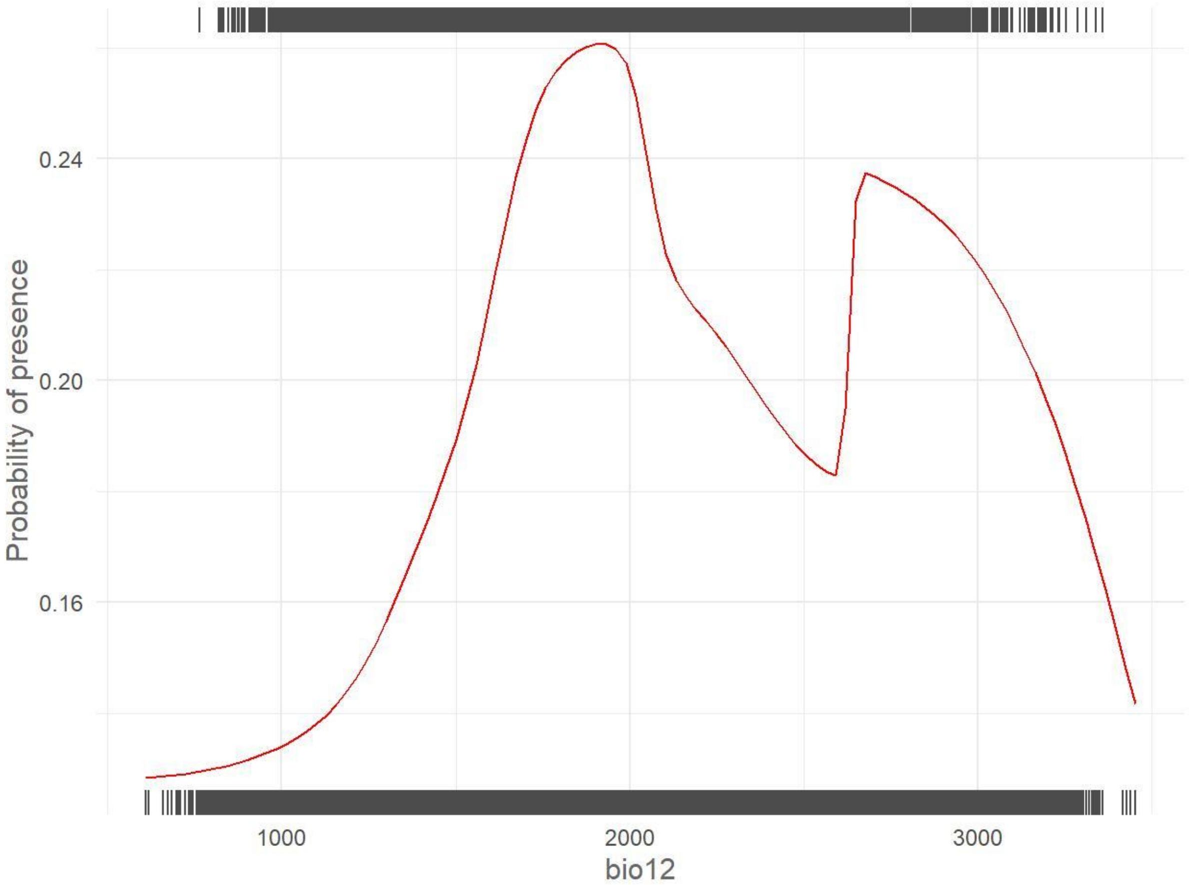

Probability of presence
0.24
0.20
0.16
1000
2000
3000
bio12

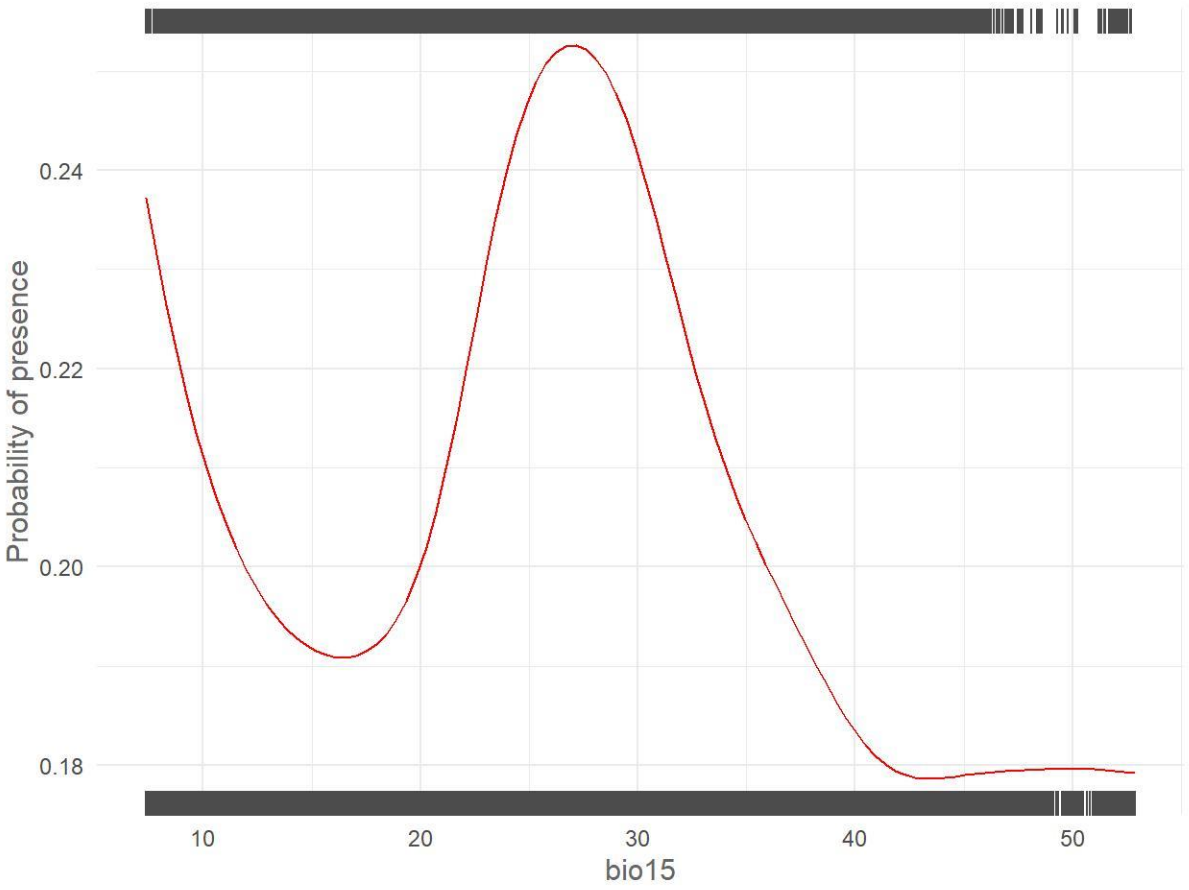

Probability of presence
0.24
0.22
0.20
0.18
10
20
30
40
50
bio15

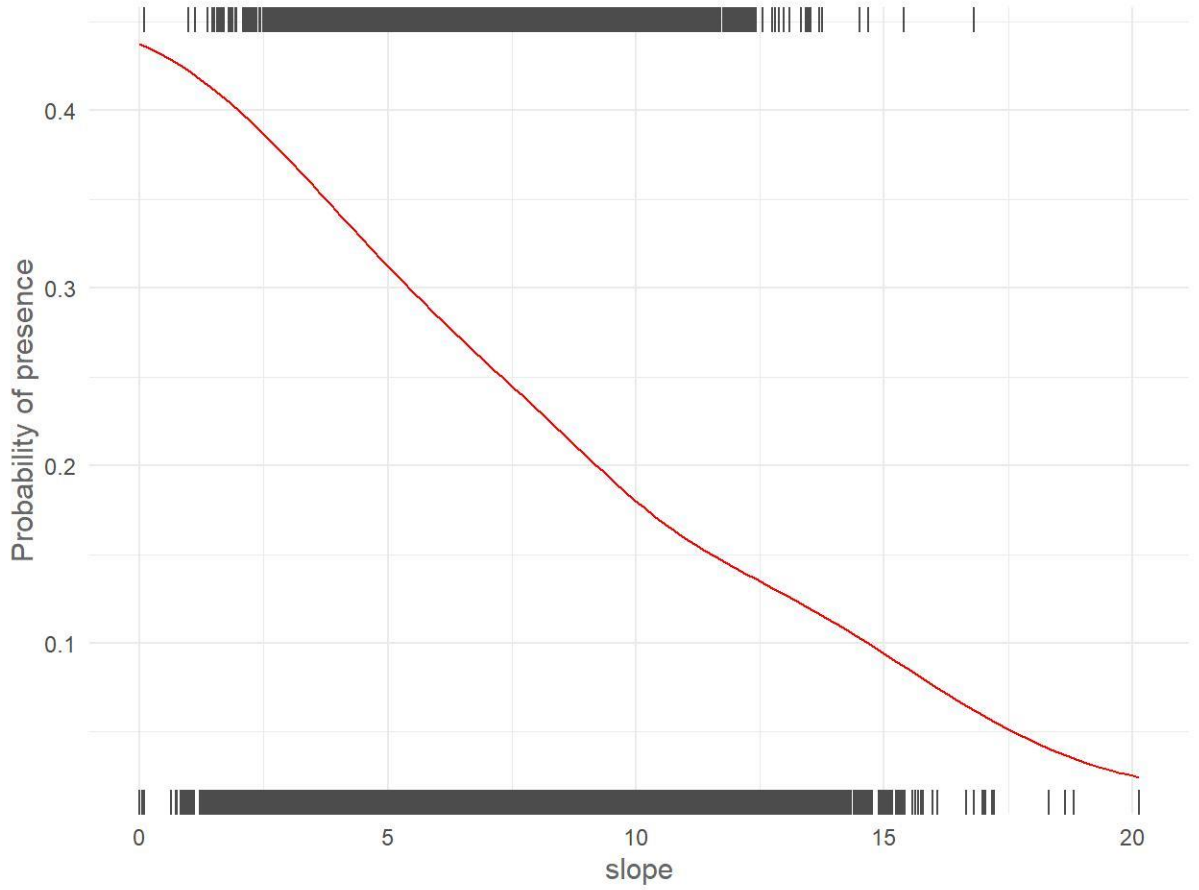

Probability of presence
0.4
0.3
0.2
0.1
0
5
10
15
20
slope

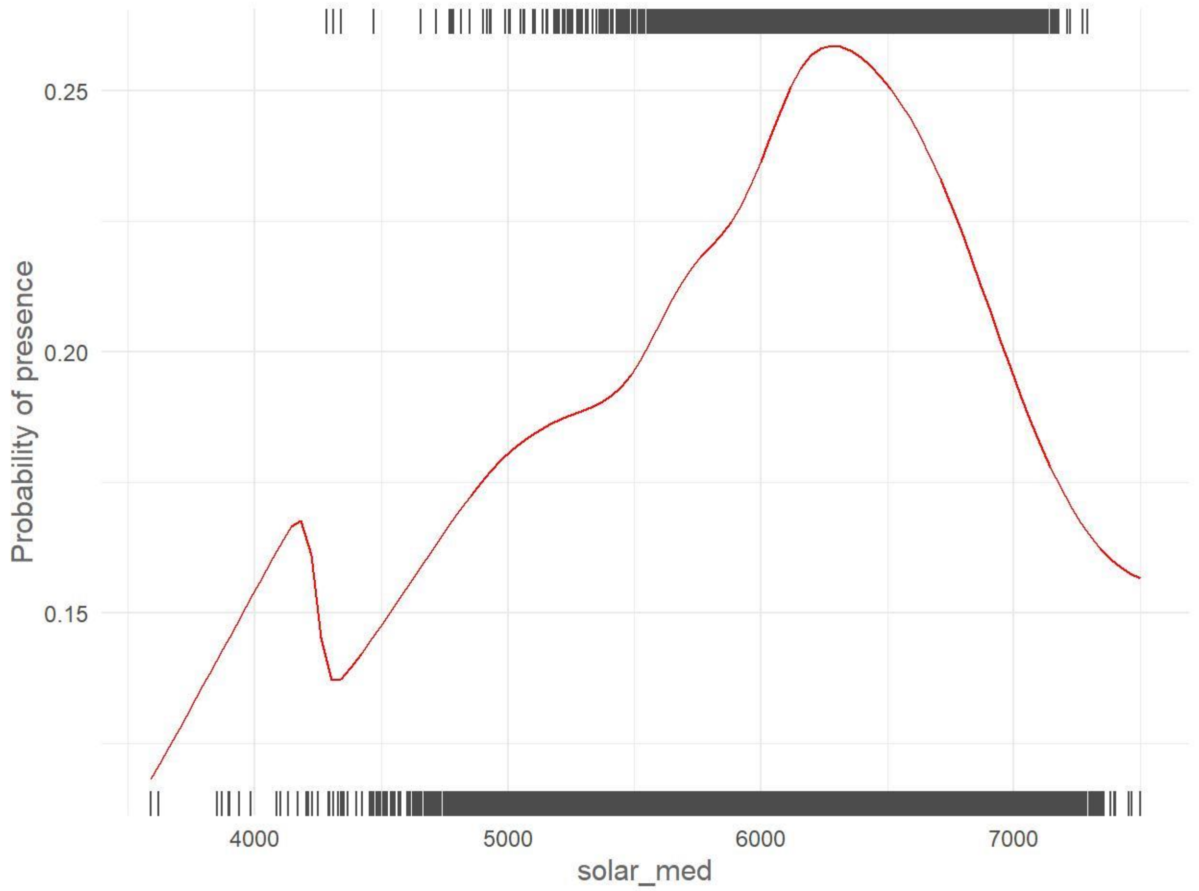

Probability of presence
0.25
0.20
0.15
4000
5000
6000
7000
solar_med

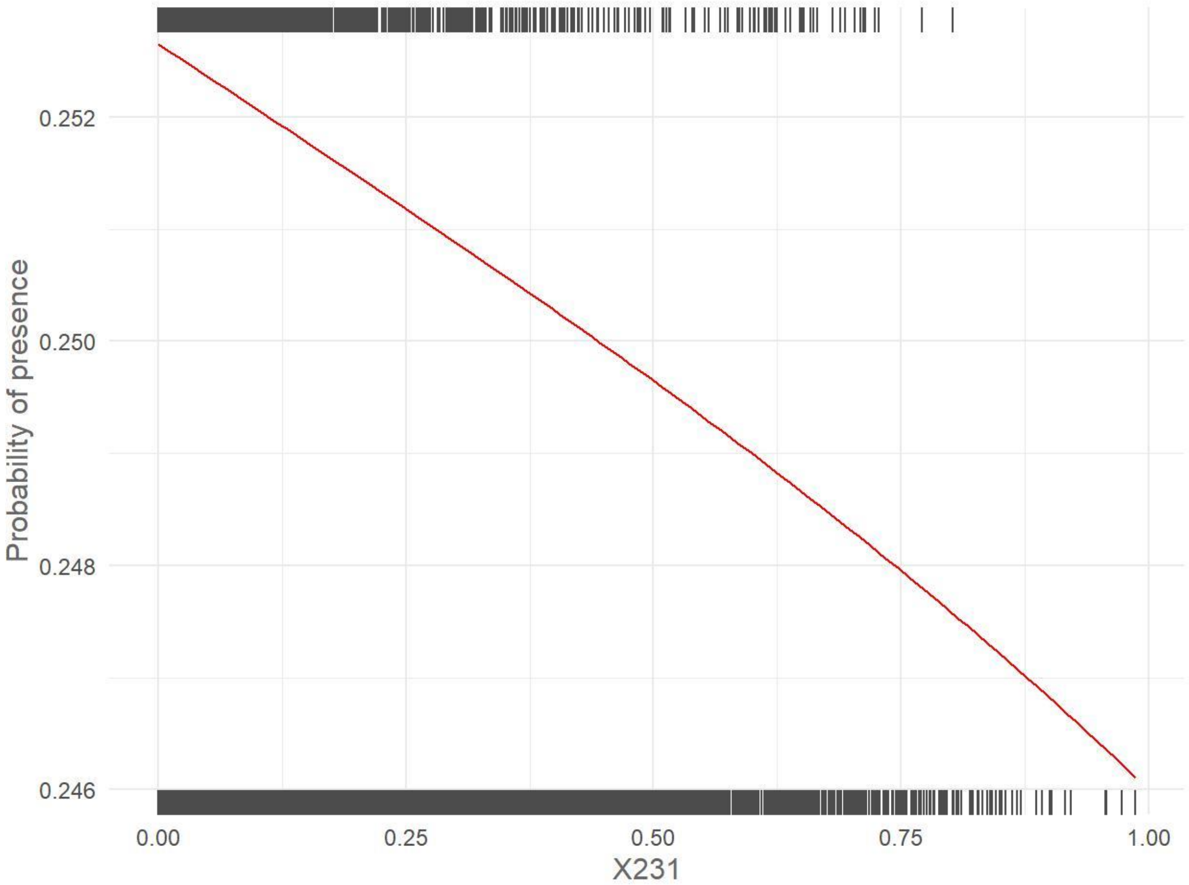
Probability of presence
0.252
0.250
0.248
0.246
0.00
0.25
0.50
0.75
1.00
X231

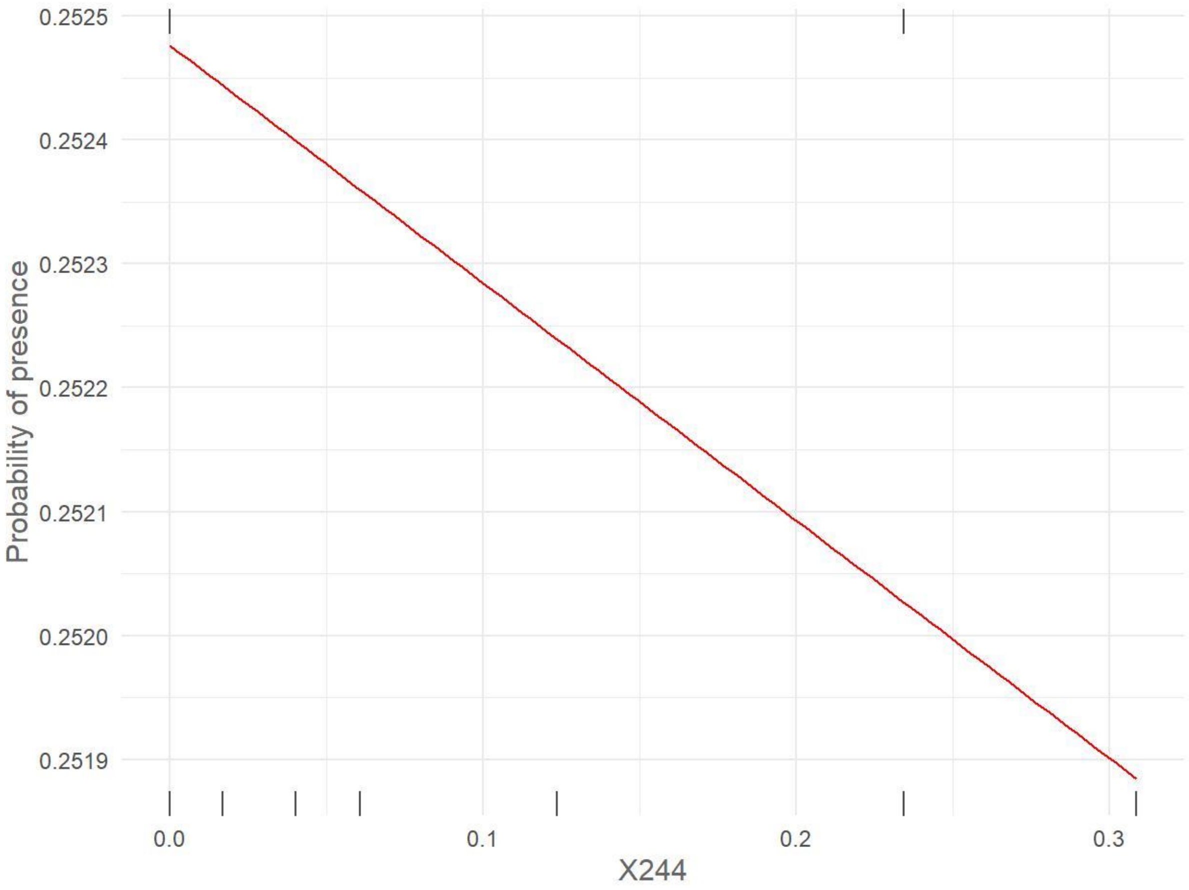
Probability of presence
0.2525
0.2524
0.2523
0.2522
0.2521
0.2520
0.2519
0.0
0.1
0.2
0.3
X244

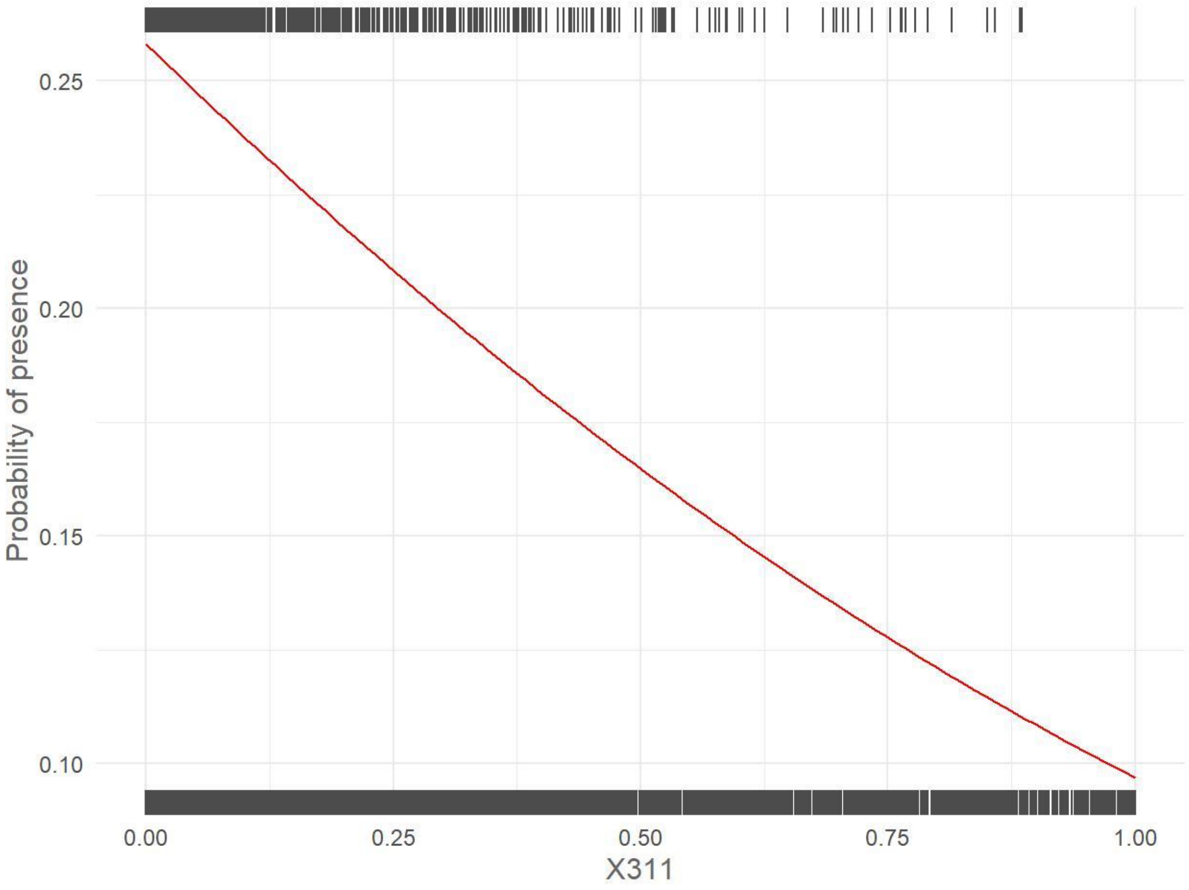

0.25
0.20
0.15
0.10
Probability of presence
0.00
0.25
0.50
0.75
1.00
X311

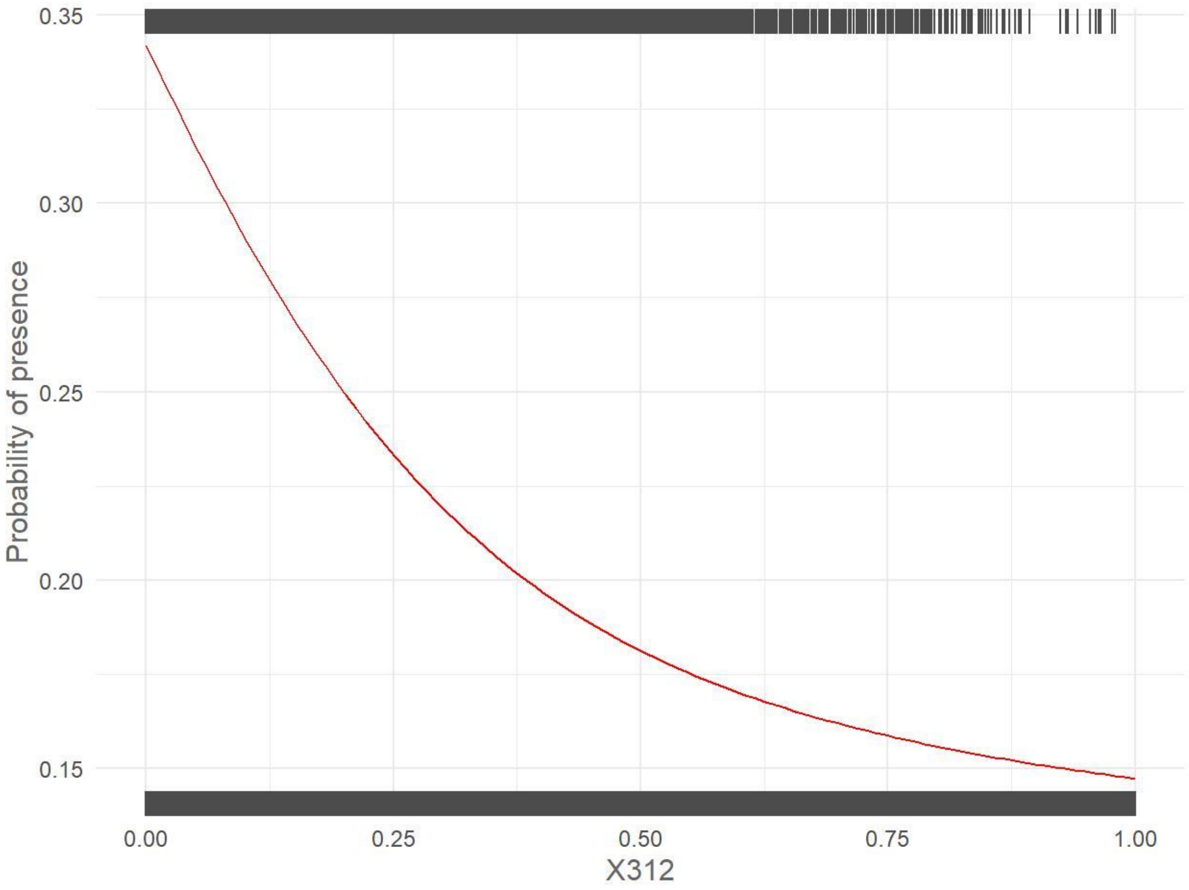

0.35
0.30
0.25
0.20
0.15
Probability of presence
0.00
0.25
0.50
0.75
1.00
X312

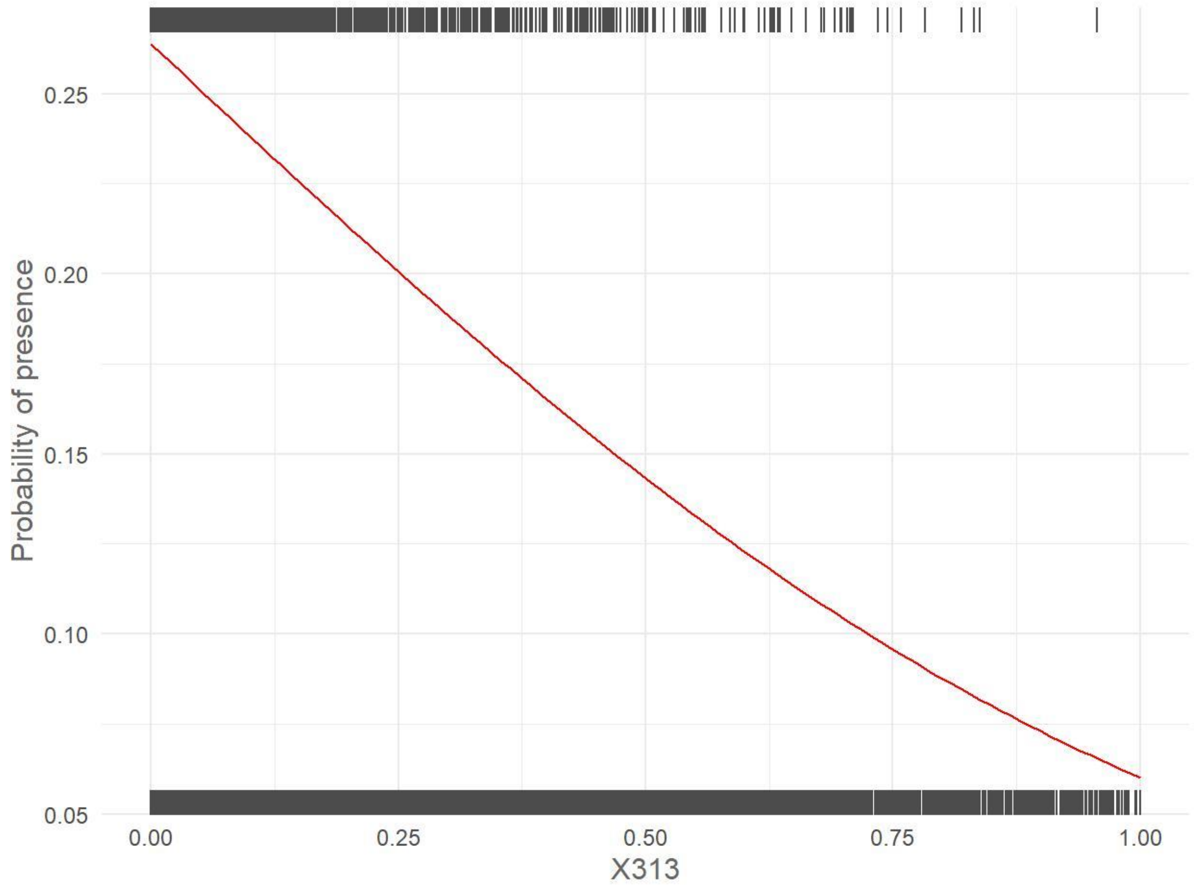
Probability of presence
0.25
0.20
0.15
0.10
0.05
0.00
0.25
0.50
0.75
1.00
X313

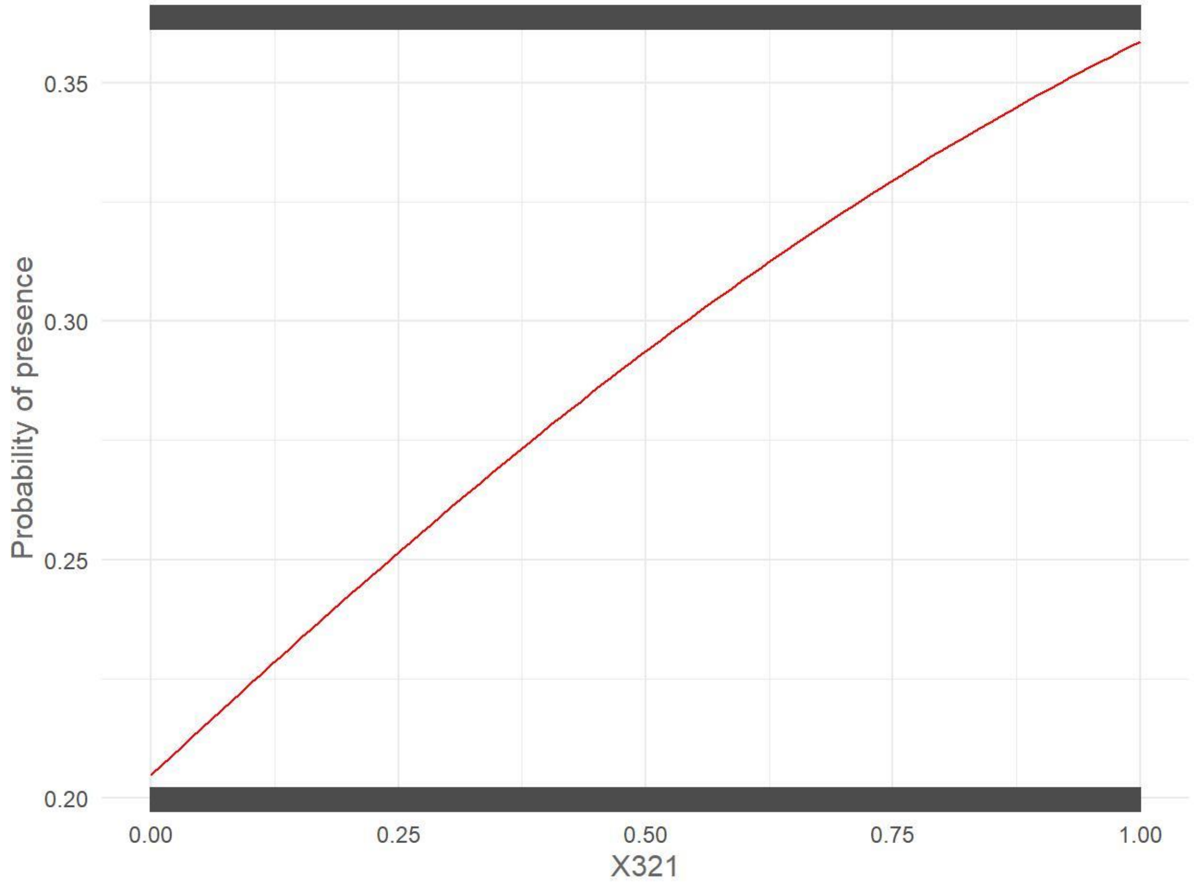
Probability of presence
0.35
0.30
0.25
0.20
0.00
0.25
0.50
0.75
1.00
X321

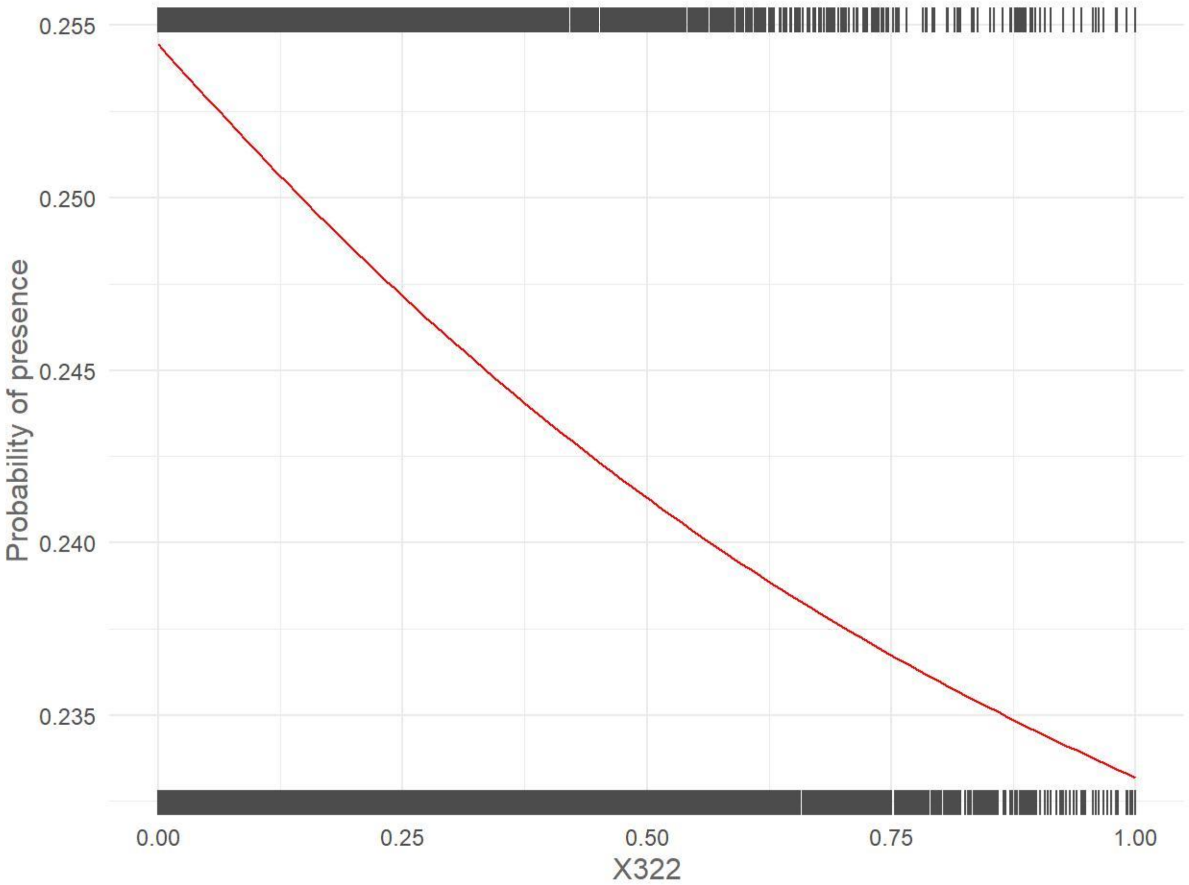
0.255
0.250
0.245
0.240
0.235
Probability of presence
0.00
0.25
0.50
0.75
1.00
X322

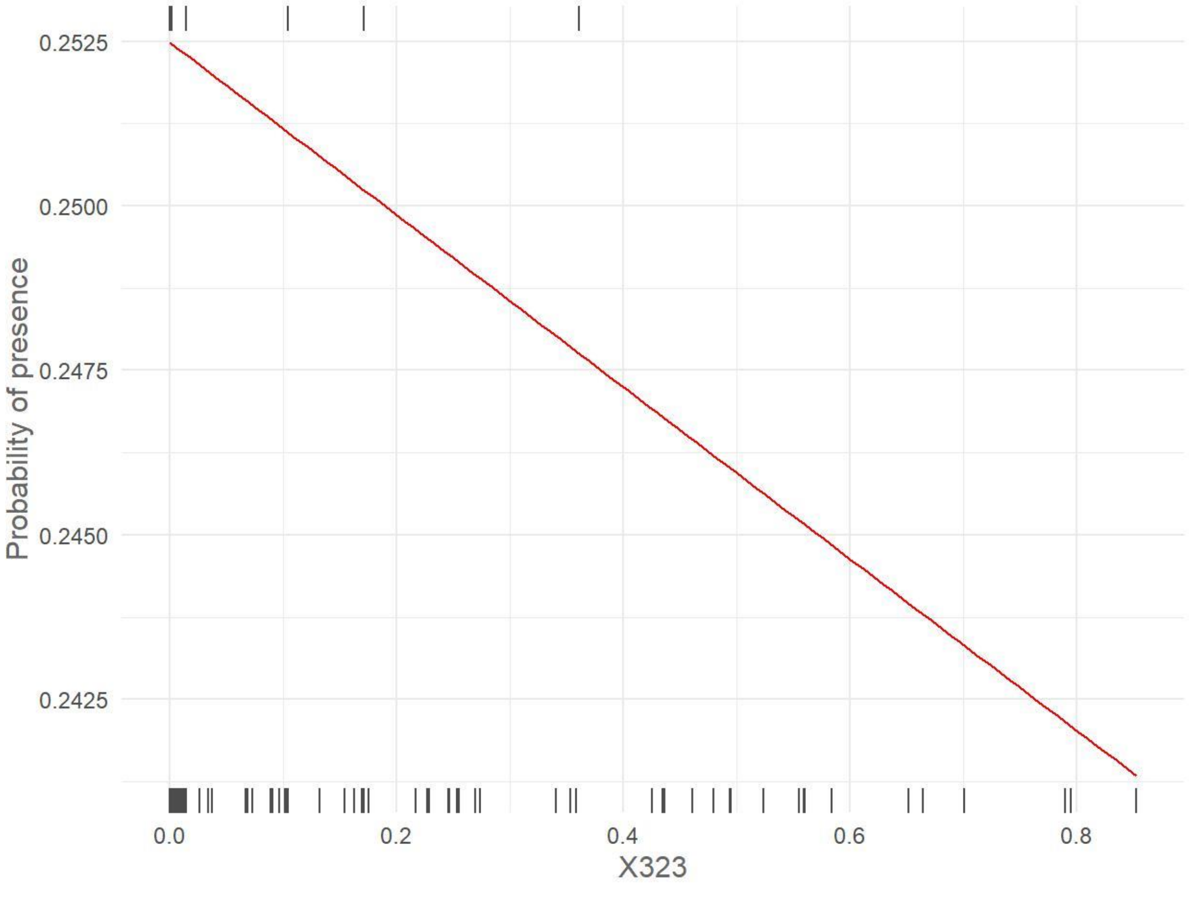
0.2525
0.2500
0.2475
0.2450
0.2425
Probability of presence
0.0
0.2
0.4
0.6
0.8
X323

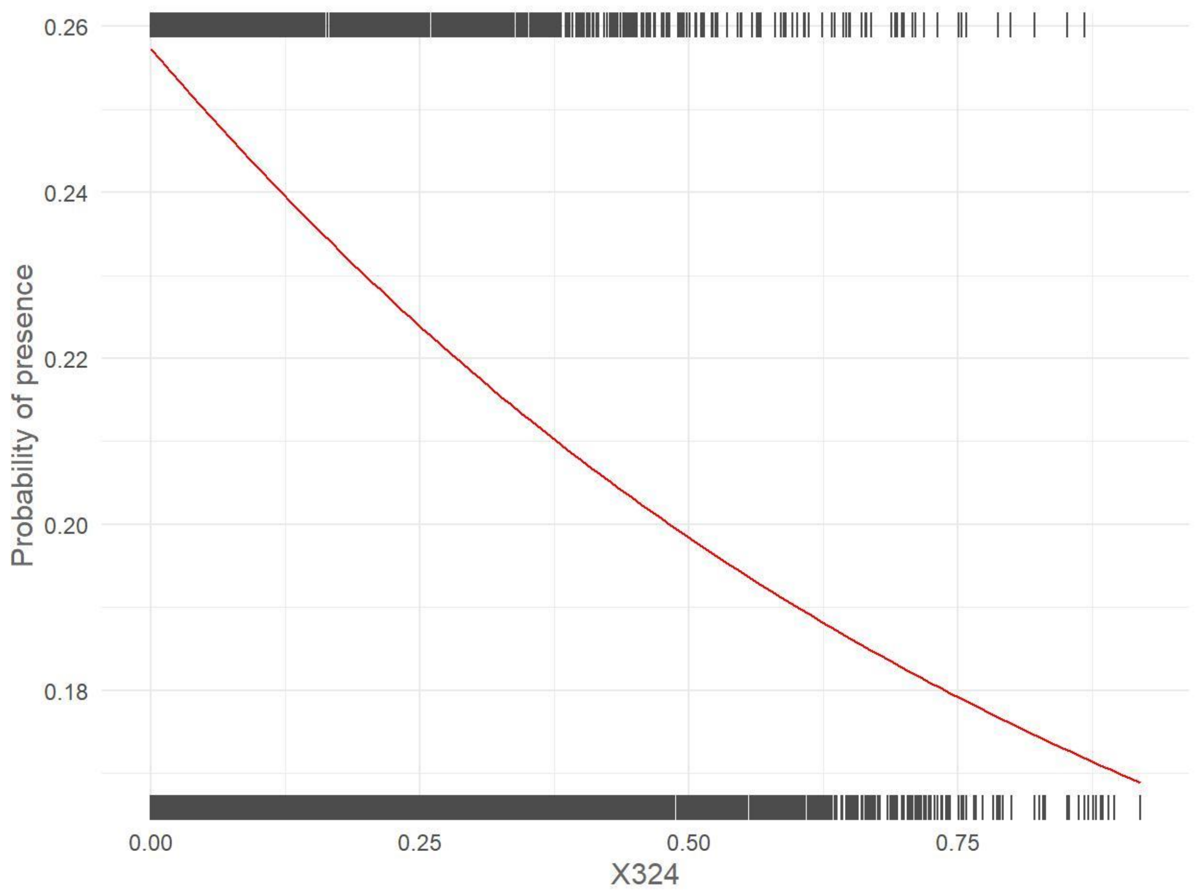
0.26
0.24
0.22
0.20
0.18
Probability of presence
0.00
0.25
0.50
0.75
X324

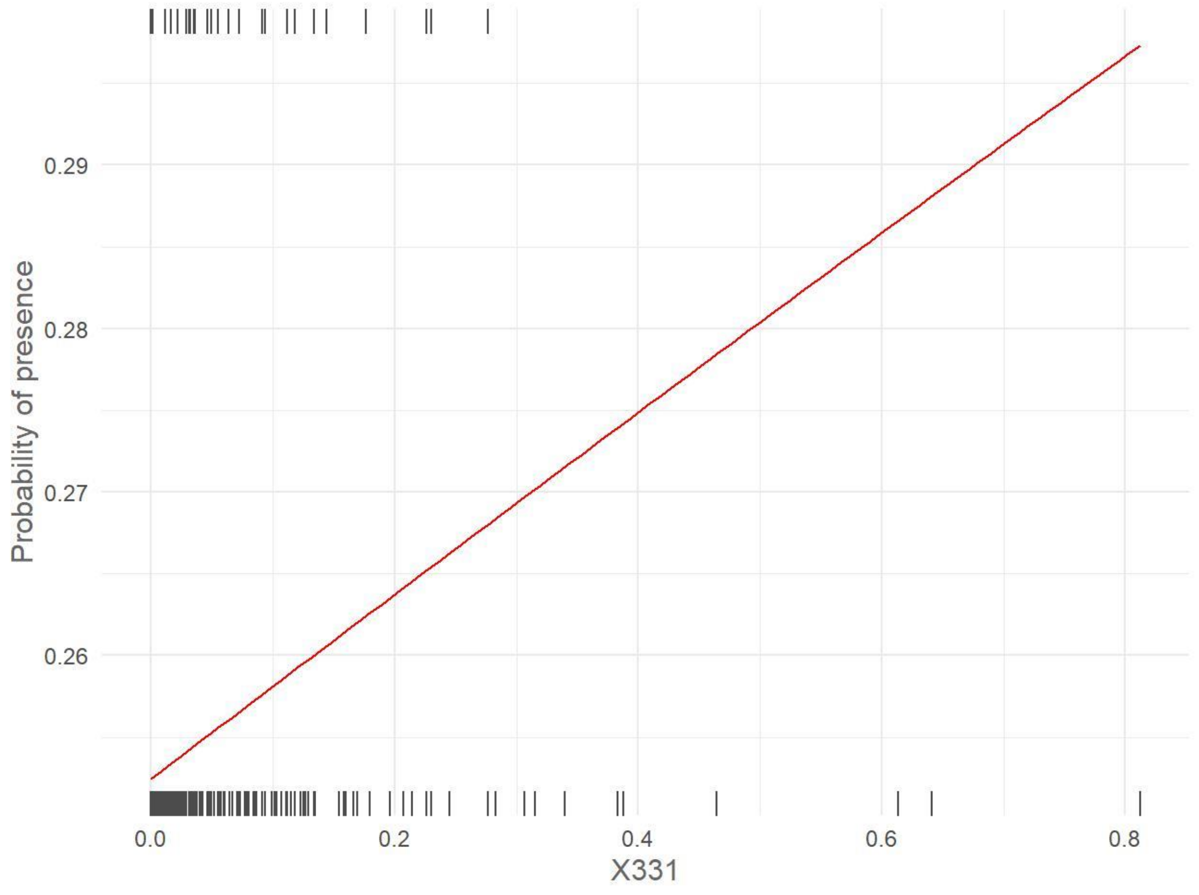
0.29
0.28
0.27
0.26
Probability of presence
0.0
0.2
0.4
0.6
0.8
X331

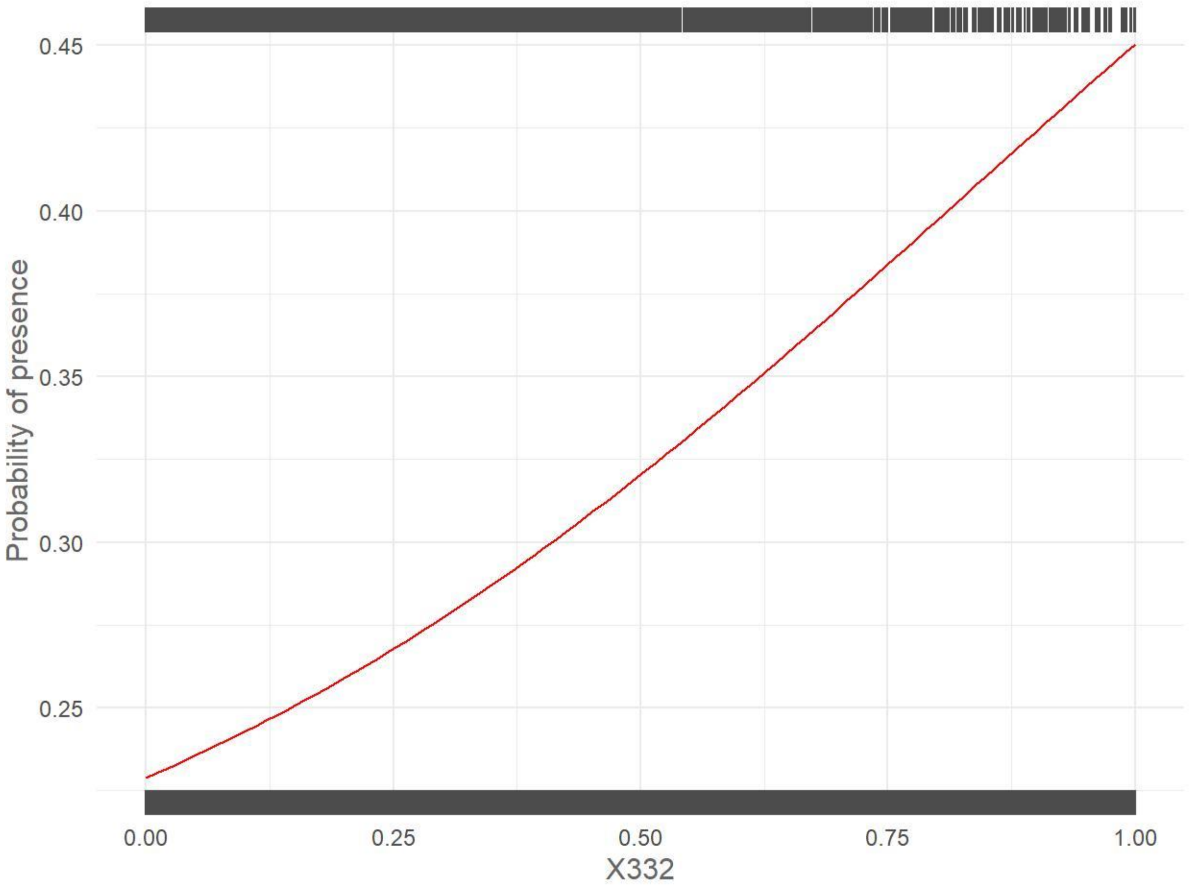
0.45
0.40
0.35
0.30
0.25
Probability of presence
0.00
0.25
0.50
0.75
1.00
X332

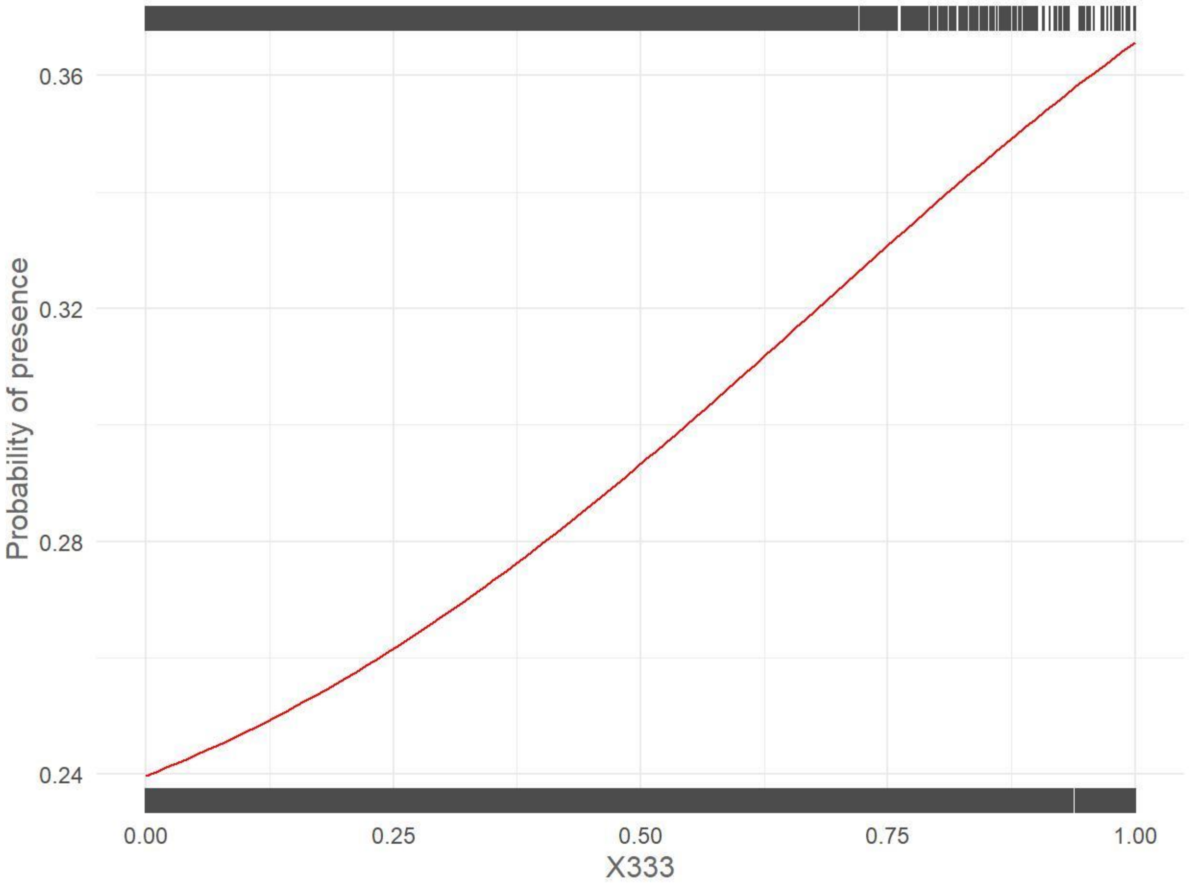
0.36
0.32
0.28
0.24
Probability of presence
0.00
0.25
0.50
0.75
1.00
X333

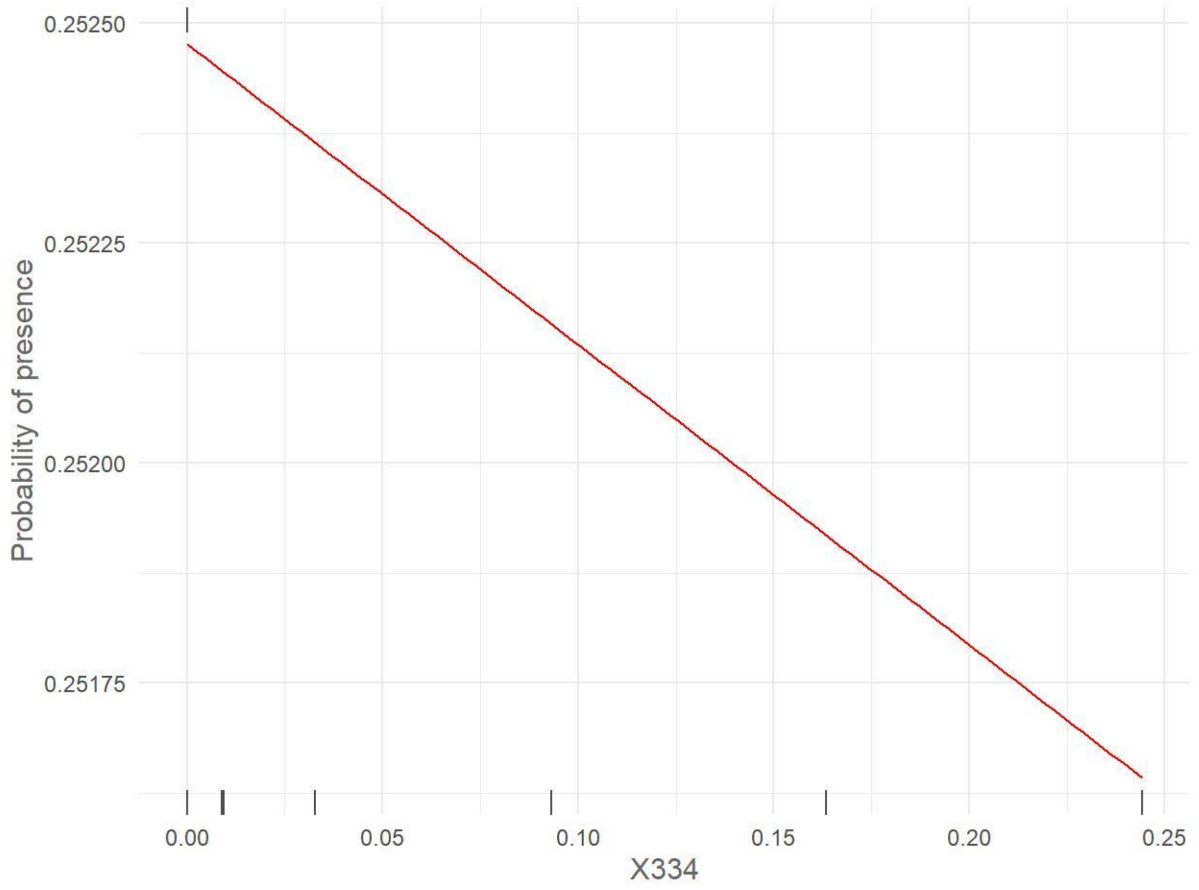

Probability of presence
0.25250
0.25225
0.25200
0.25175
0.00
0.05
0.10
0.15
0.20
0.25
X334

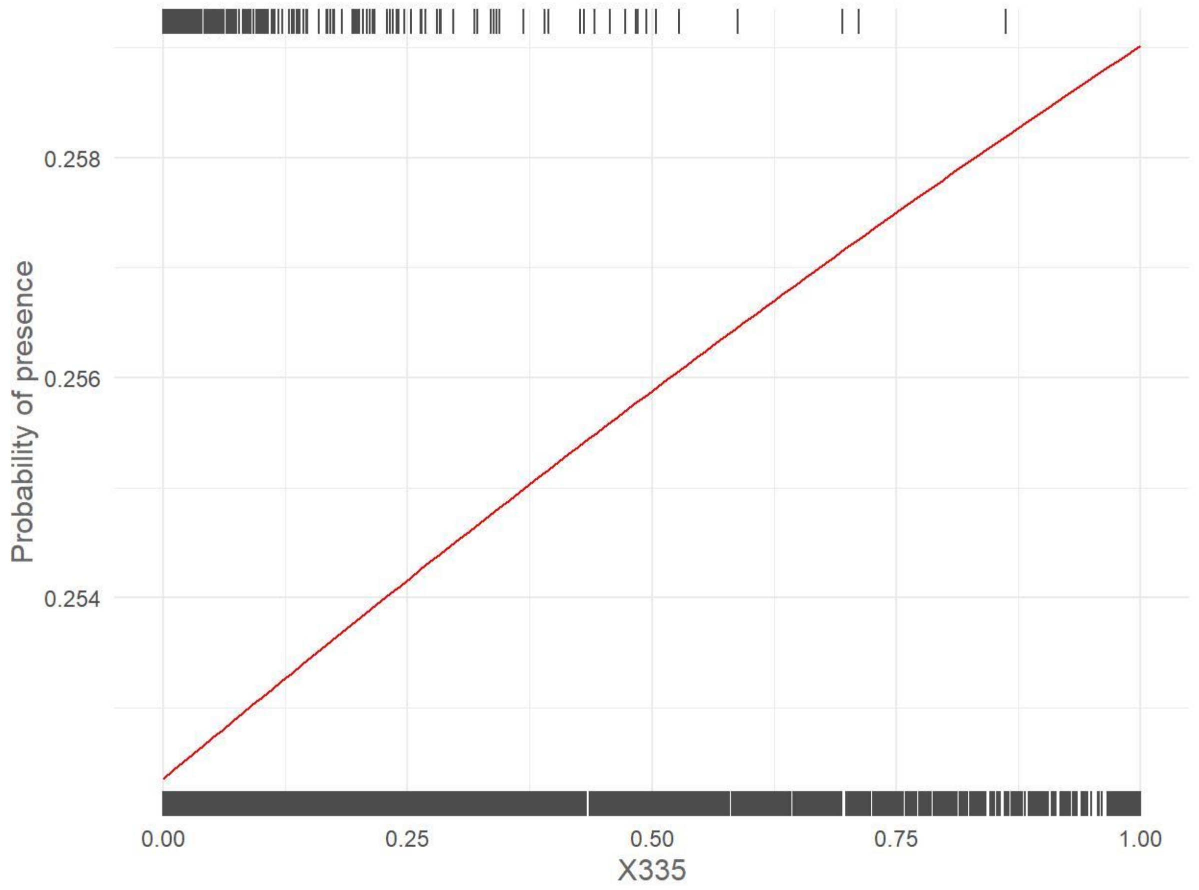

Probability of presence
0.258
0.256
0.254
0.00
0.25
0.50
0.75
1.00
X335

Figure S7. Species-habitat relationships according to the Artificial Neural Network model for alpine accentor.

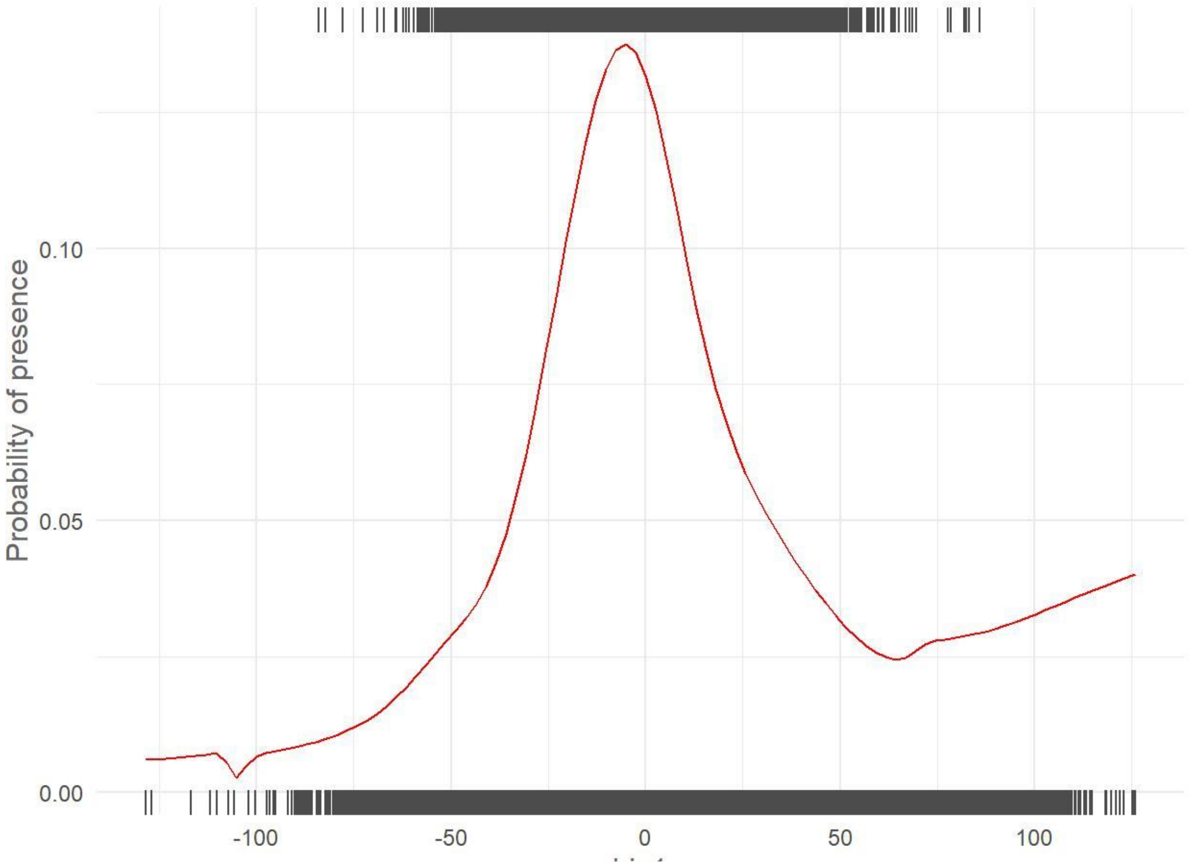

Probability of presence
0.10
0.05
0.00
-100
-50
0
50
100

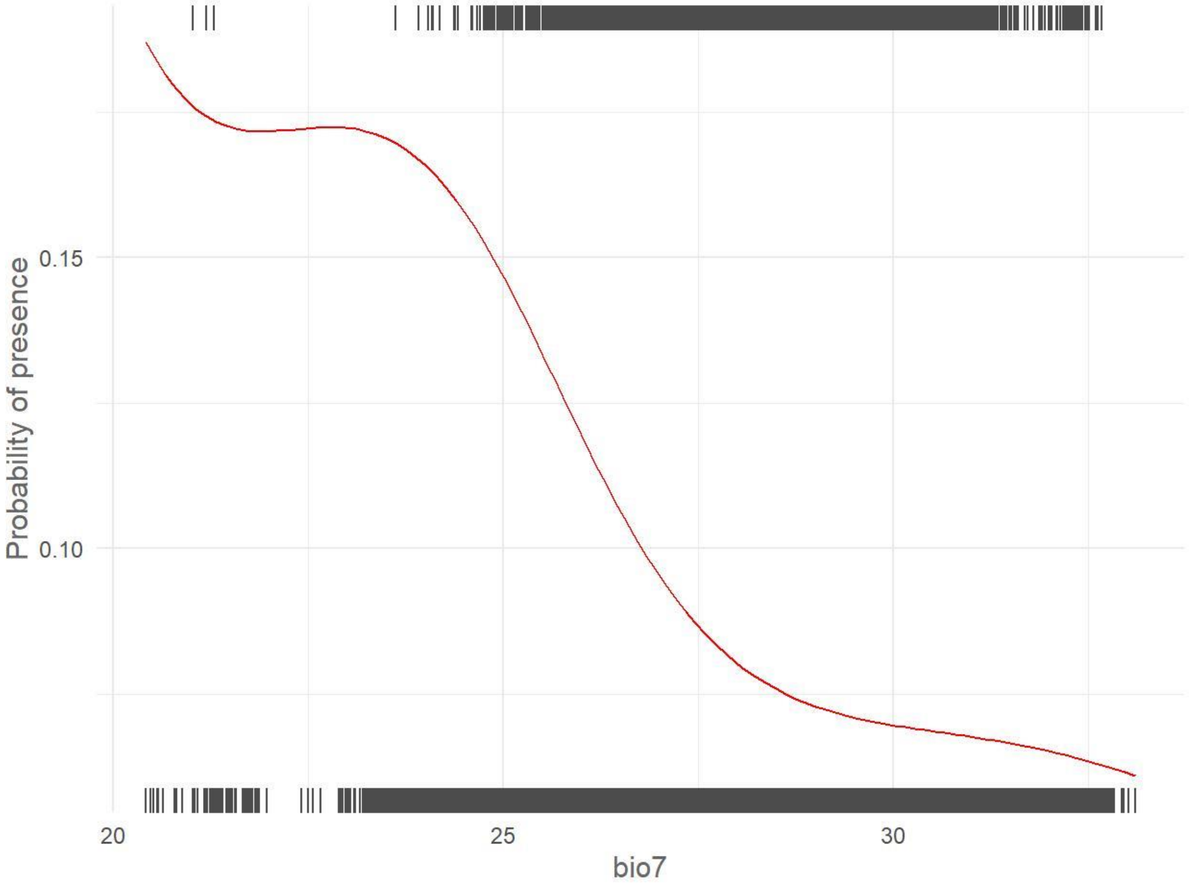

Probability of presence
0.15
0.10
20
25
30
bio7

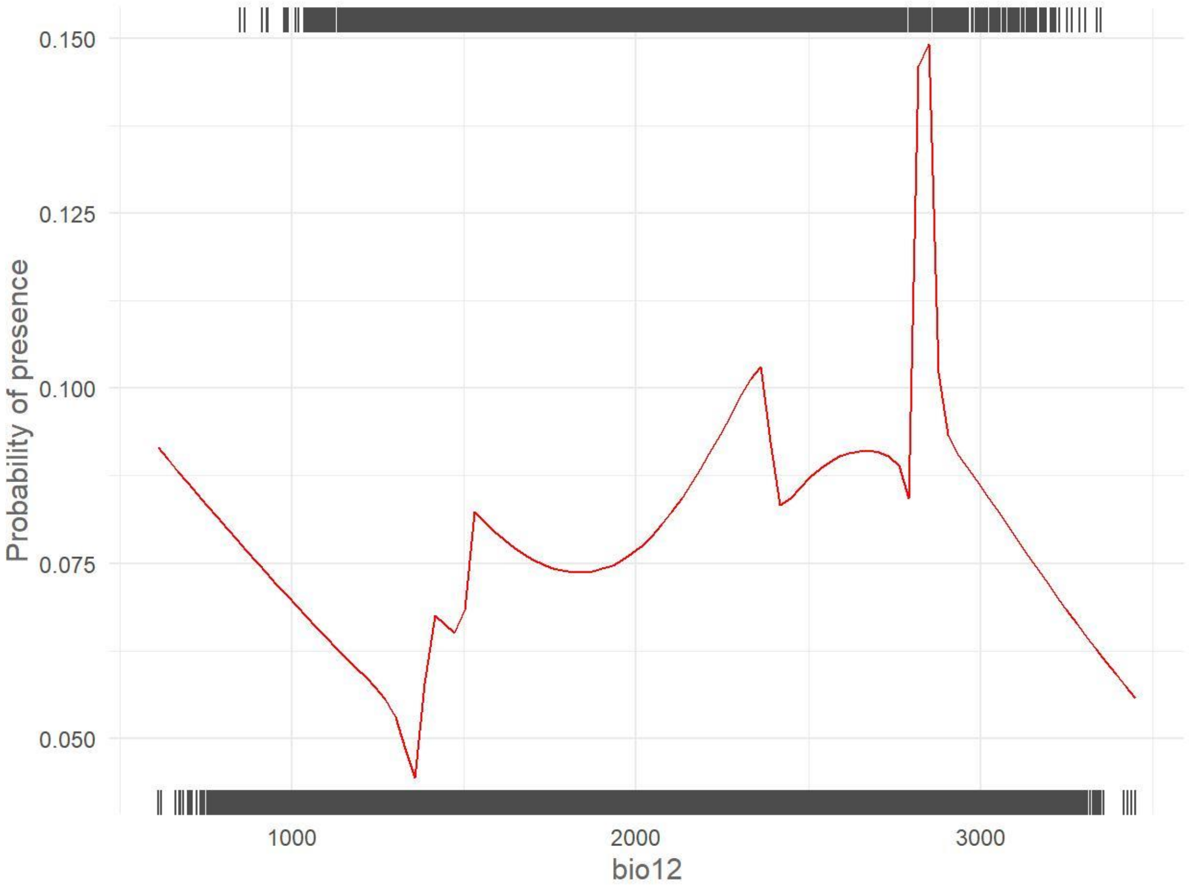
Probability of presence
0.150
0.125
0.100
0.075
0.050
1000
2000
3000
bio12

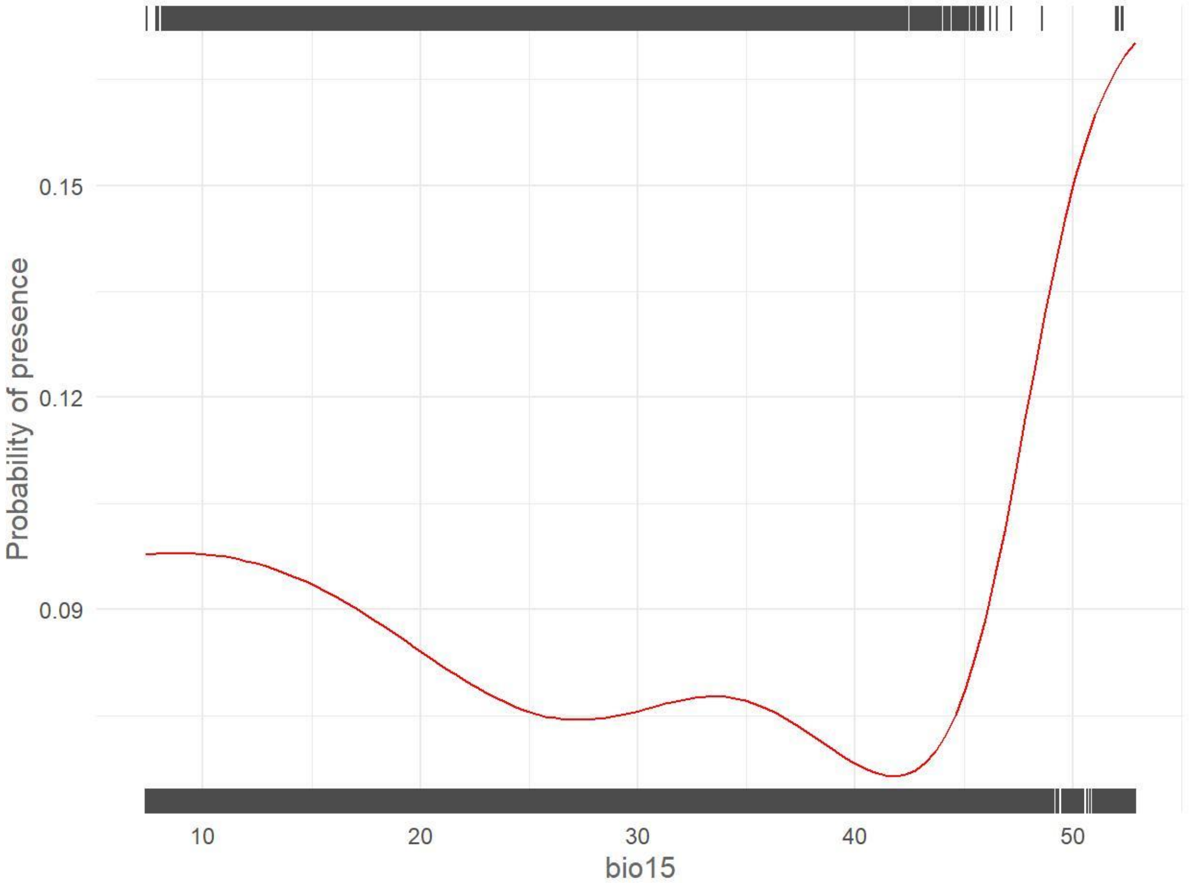
Probability of presence
0.15
0.12
0.09
10
20
30
40
50
bio15

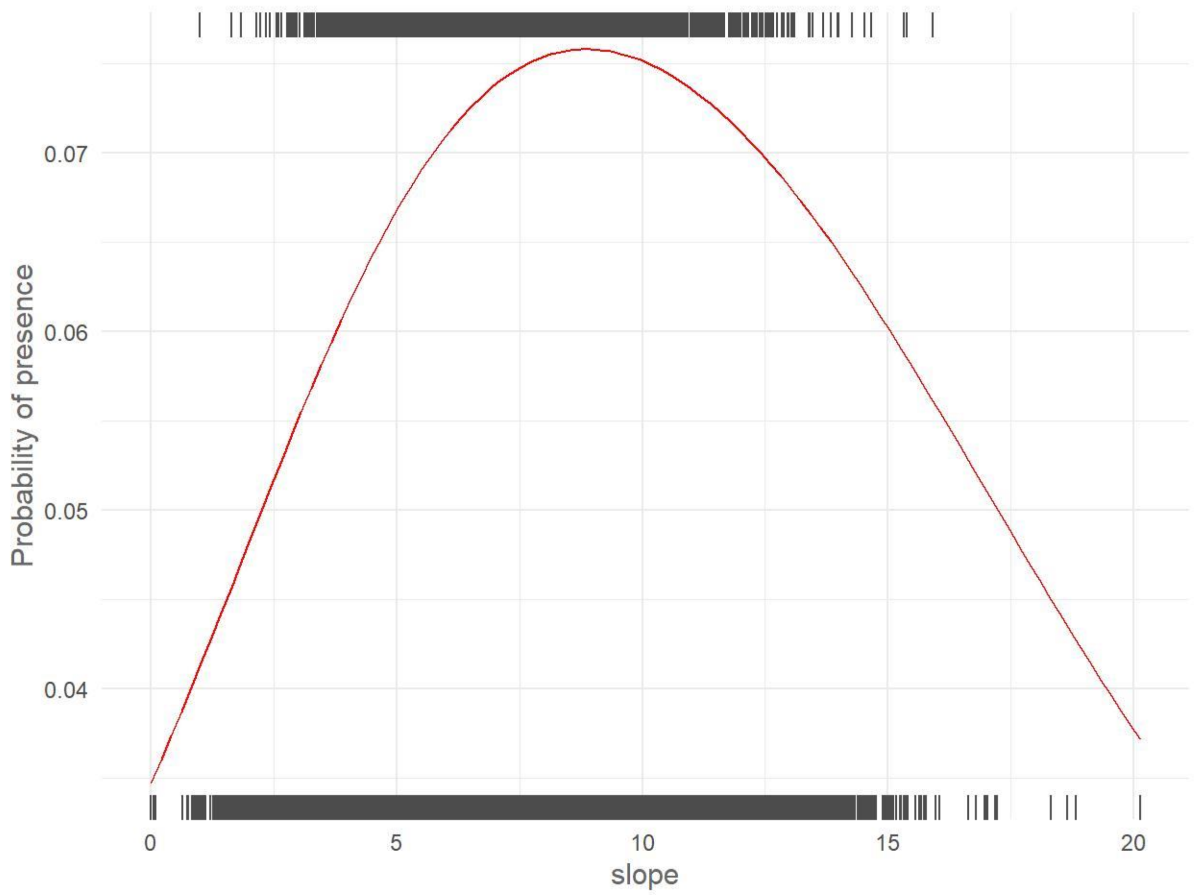

Probability of presence
0.07
0.06
0.05
0.04
0
5
10
15
20
slope

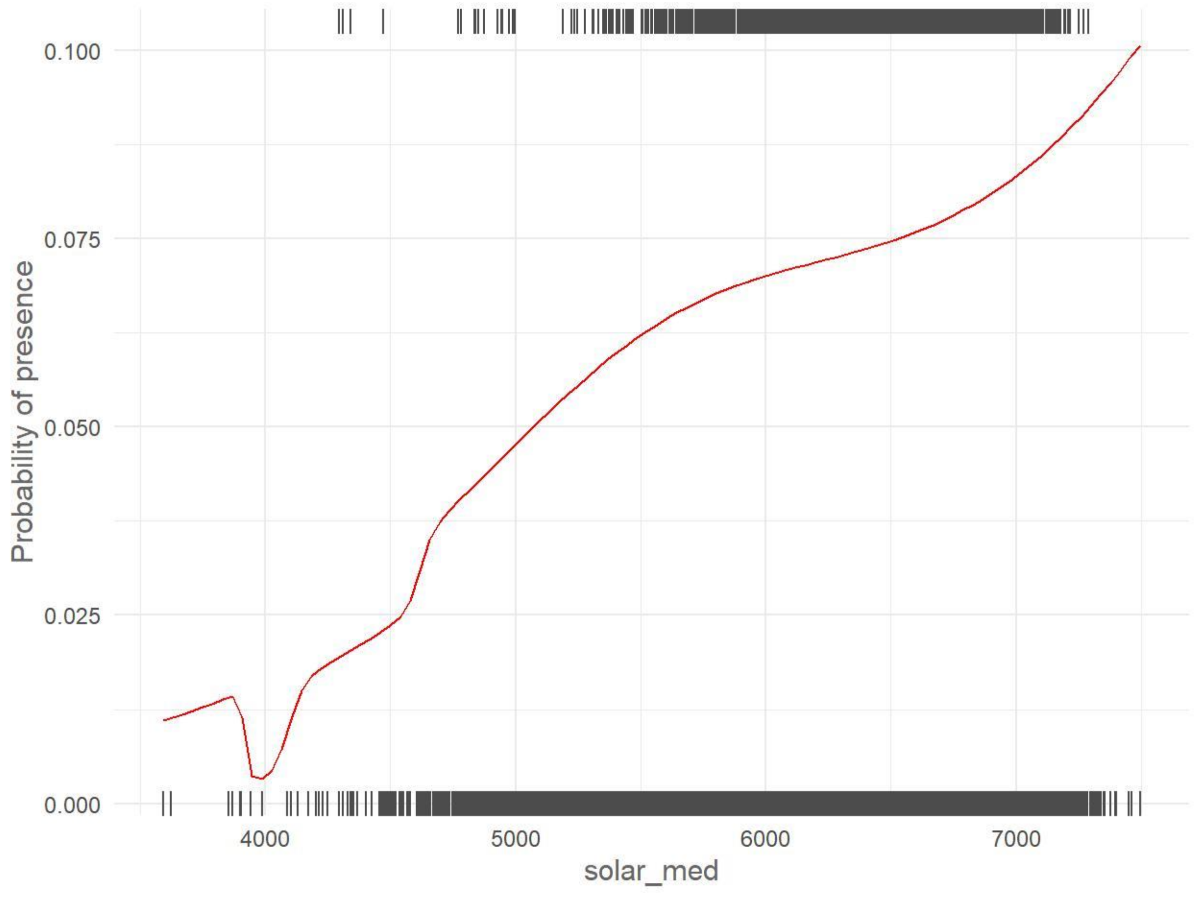

Probability of presence
0.100
0.075
0.050
0.025
0.000
4000
5000
6000
7000
solar_med

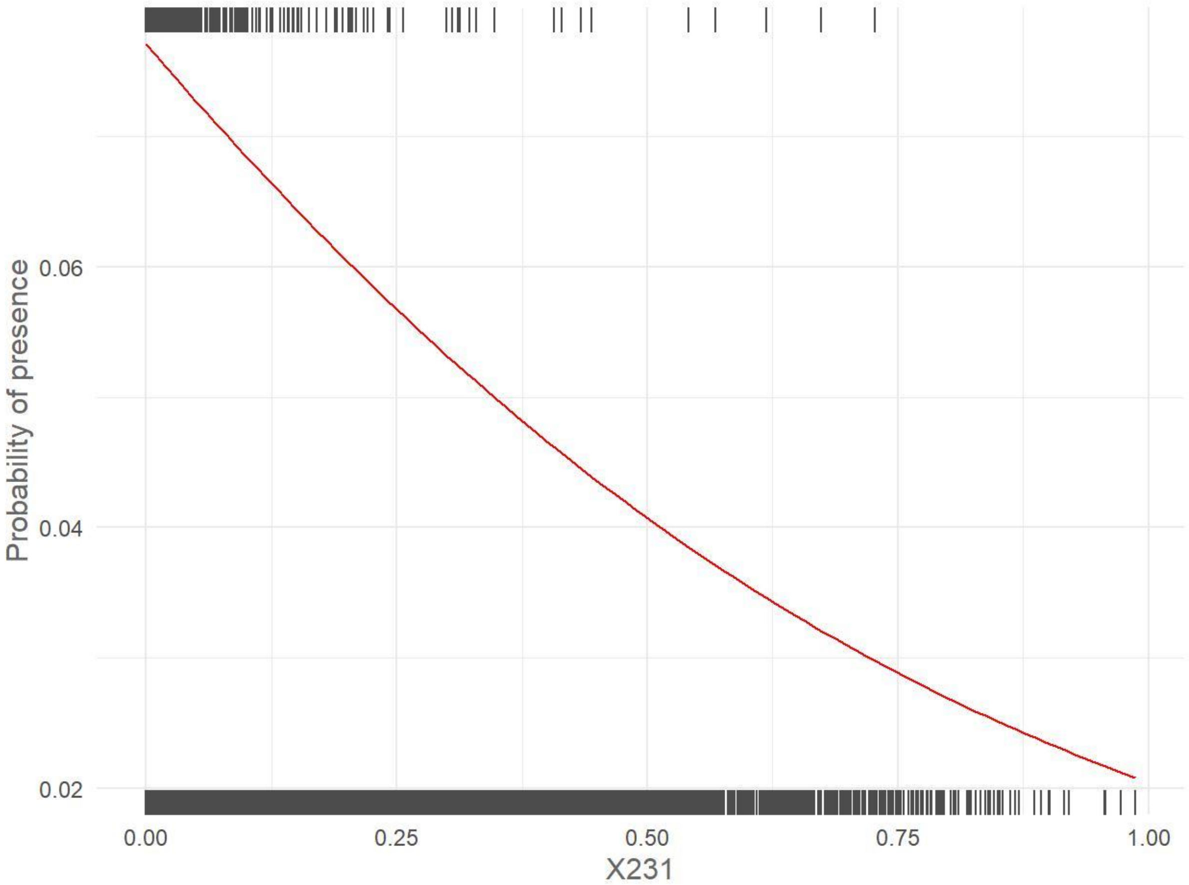
Probability of presence
0.06
0.04
0.02
0.00
0.25
0.50
0.75
1.00
X231

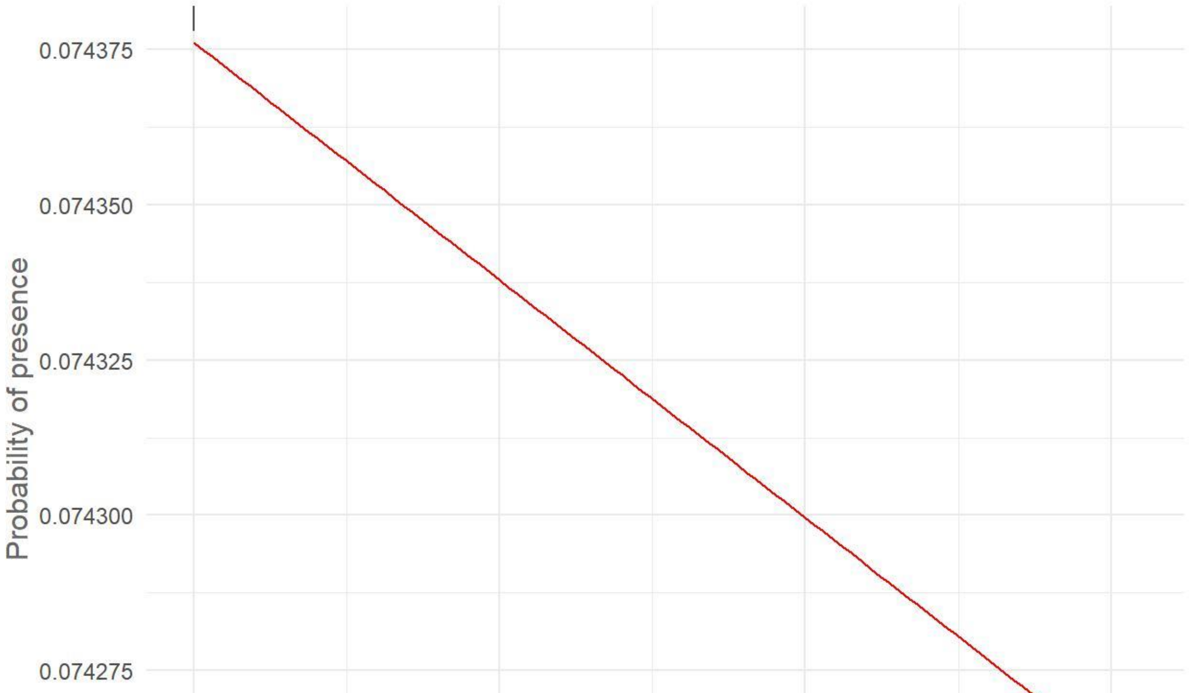
Probability of presence
0.074375
0.074350
0.074325
0.074300
0.074275
0.0
0.1
0.2
0.3
X244

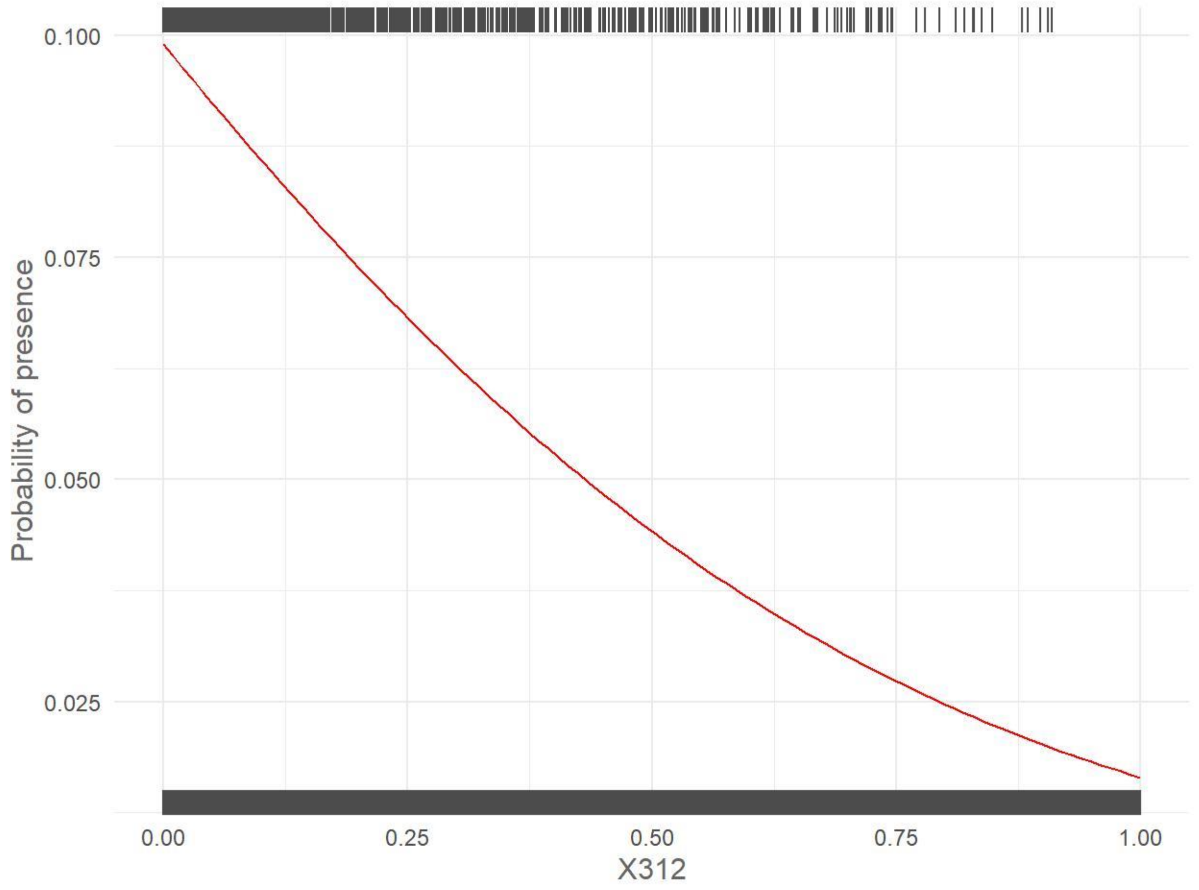
0.100
0.075
0.050
0.025
Probability of presence
0.00
0.25
0.50
0.75
1.00
X312

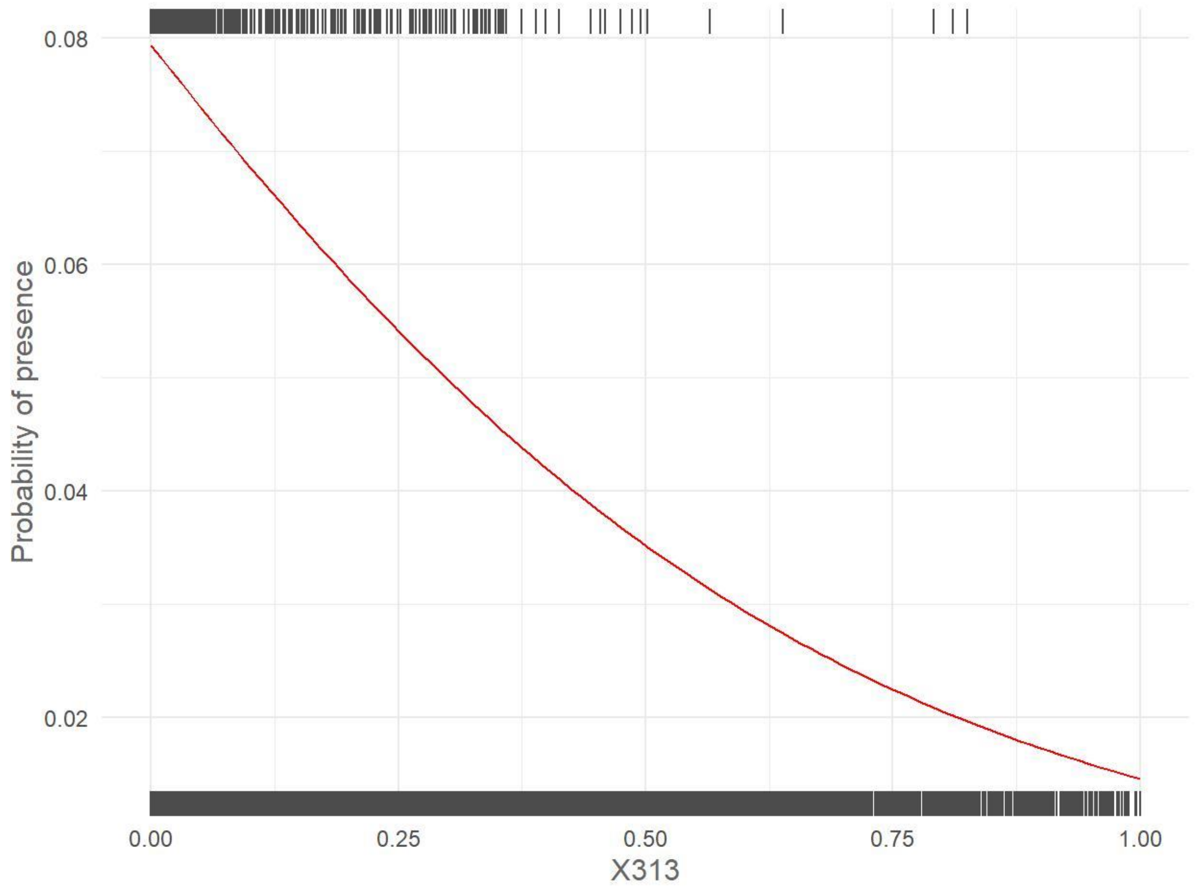
0.08
0.06
0.04
0.02
Probability of presence
0.00
0.25
0.50
0.75
1.00
X313

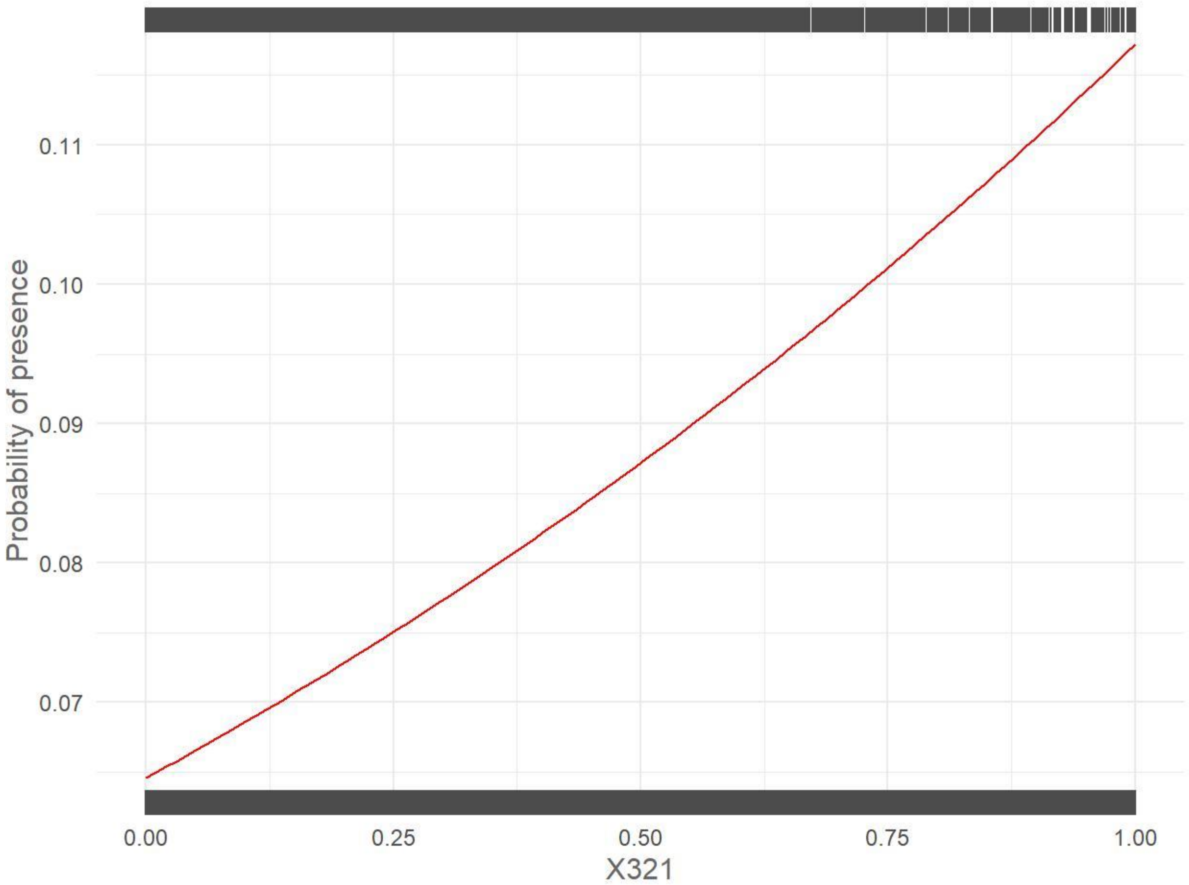

Probability of presence
0.11
0.10
0.09
0.08
0.07
0.00
0.25
0.50
0.75
1.00
X321

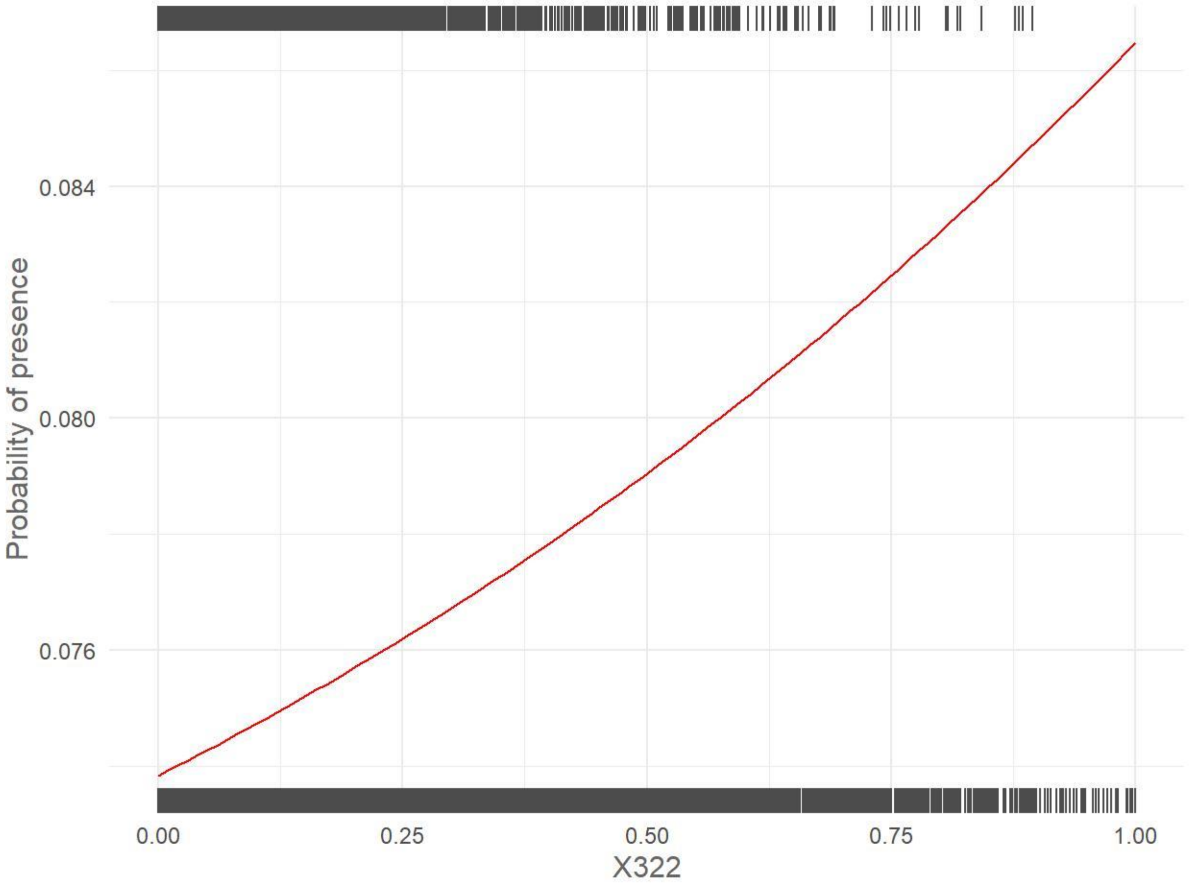

Probability of presence
0.084
0.080
0.076
0.00
0.25
0.50
0.75
1.00
X322

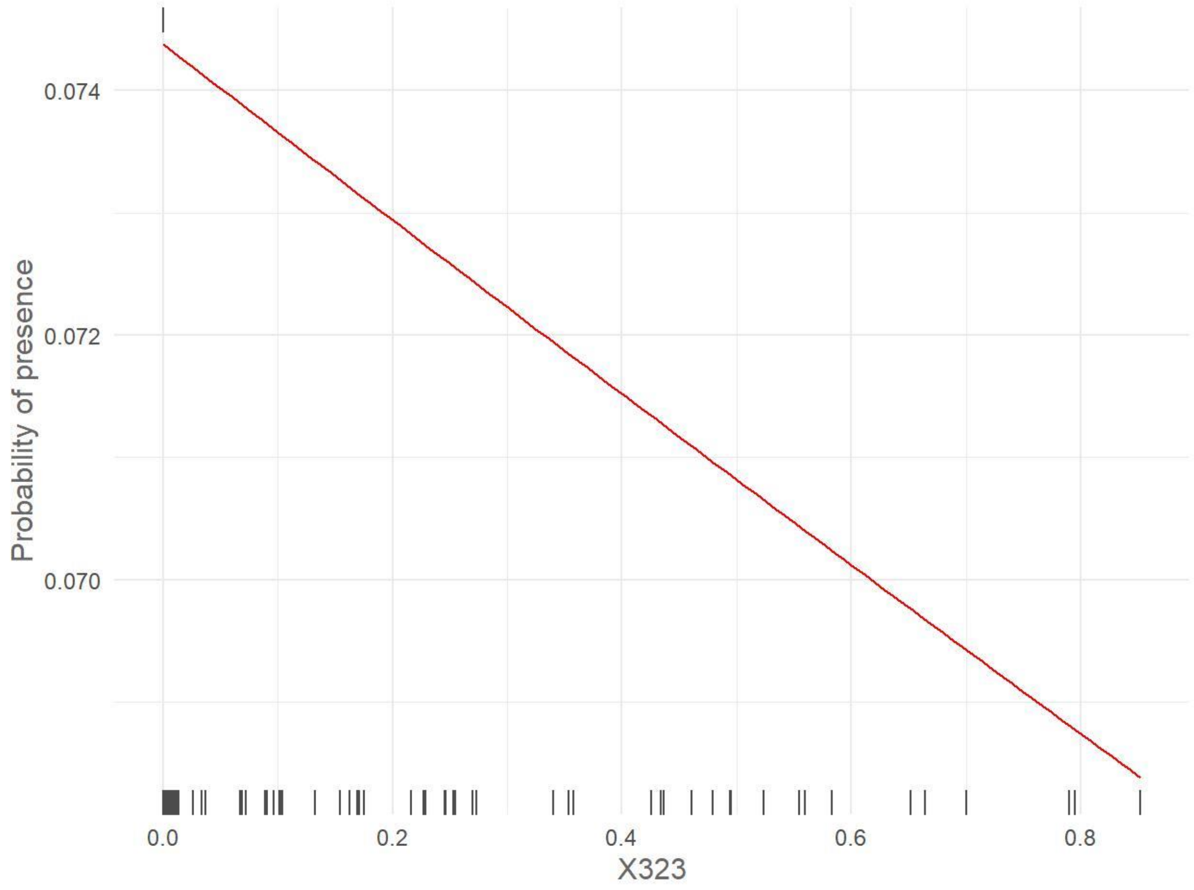
Probability of presence
0.074
0.072
0.070
0.0
0.2
0.4
0.6
0.8
X323

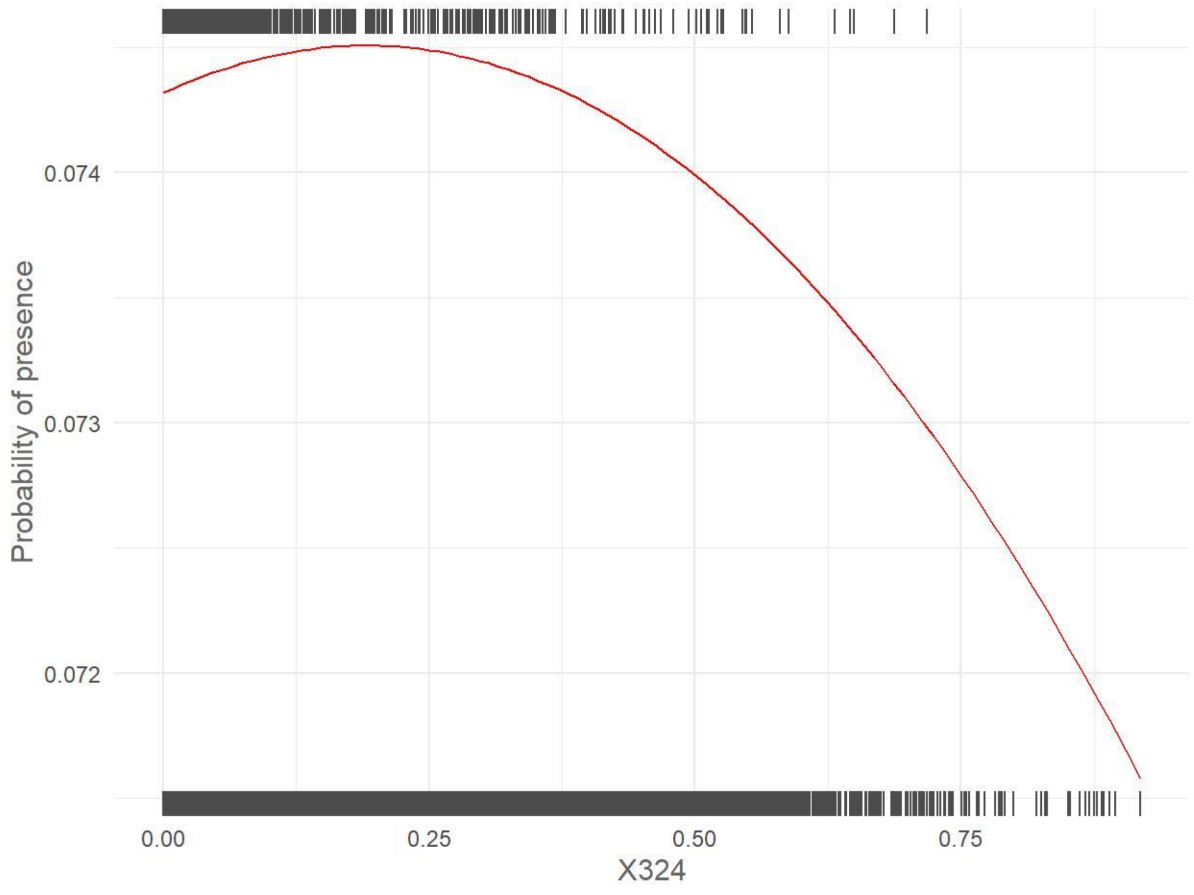
Probability of presence
0.074
0.073
0.072
0.00
0.25
0.50
0.75
X324

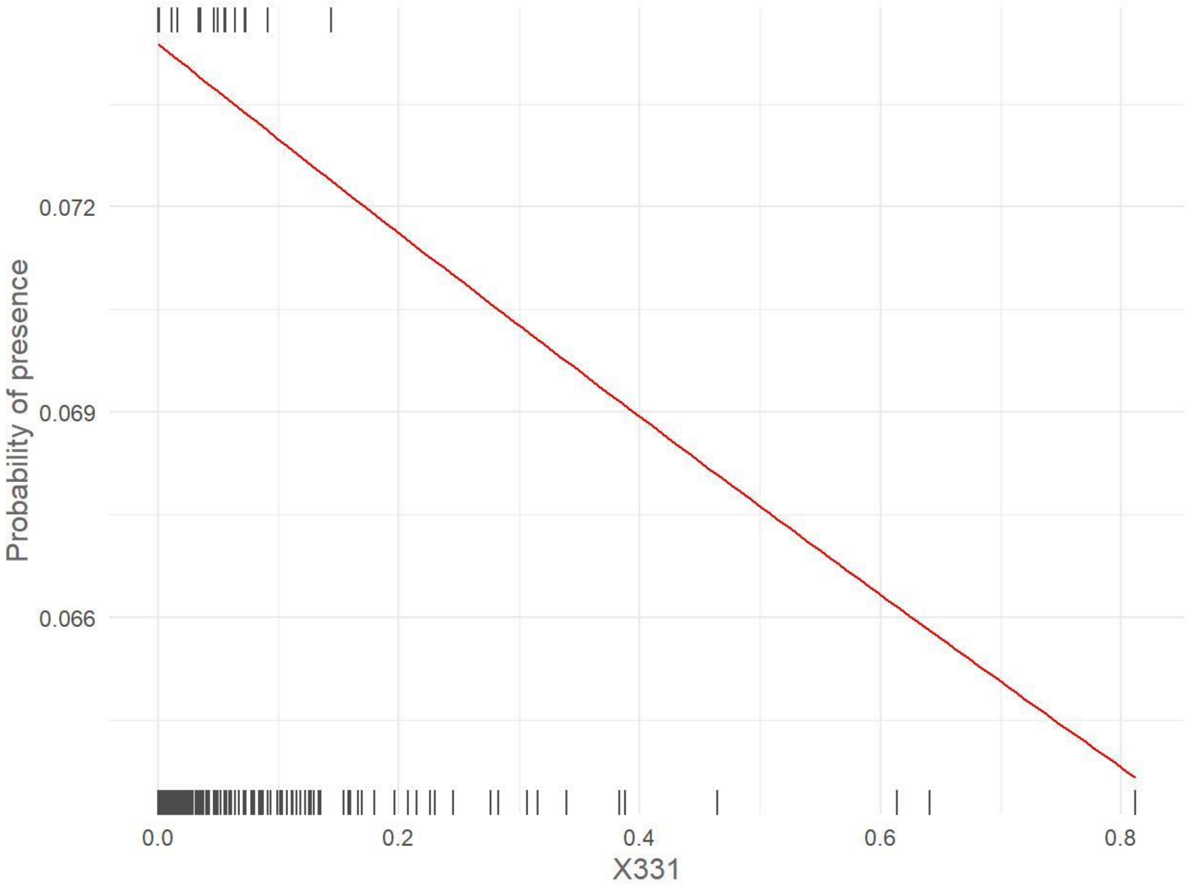
Probability of presence
0.072
0.069
0.066
0.0
0.2
0.4
0.6
0.8
X331

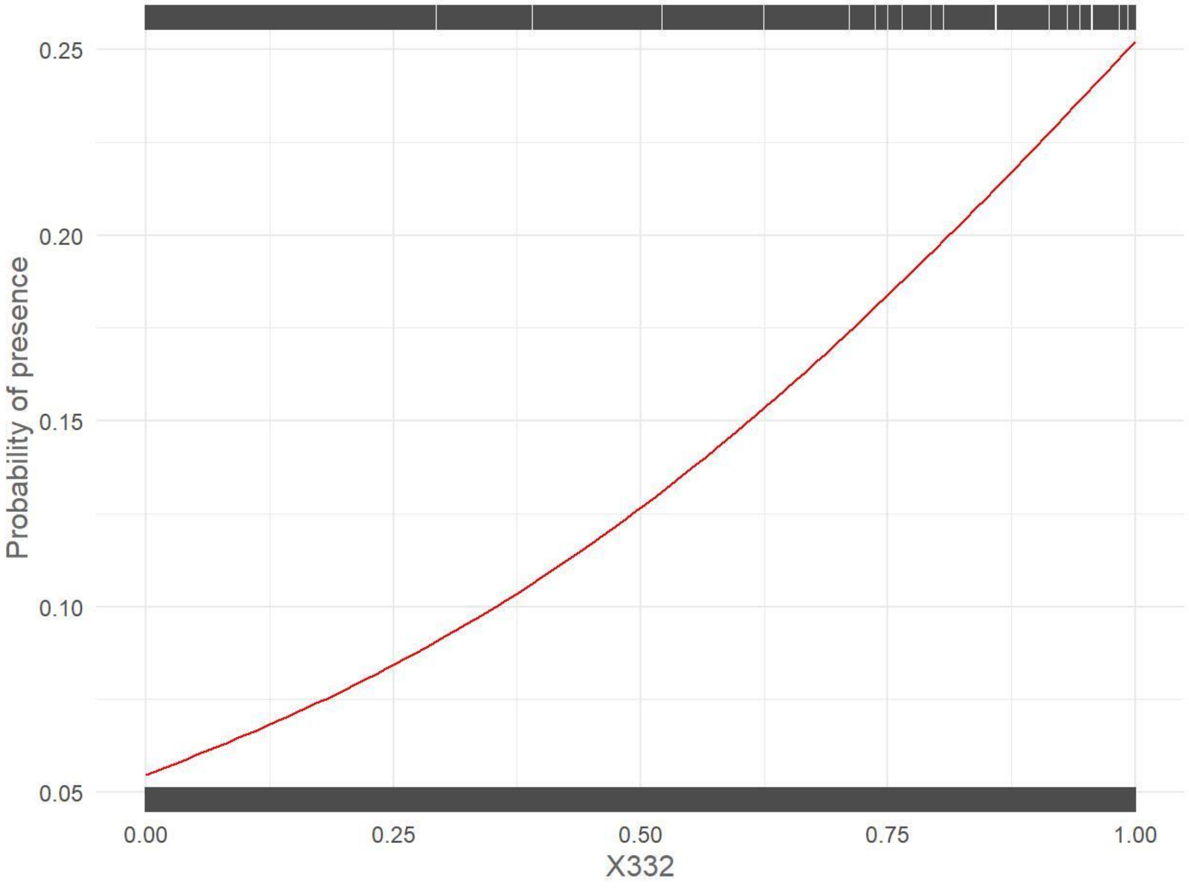
Probability of presence
0.25
0.20
0.15
0.10
0.05
0.00
0.25
0.50
0.75
1.00
X332

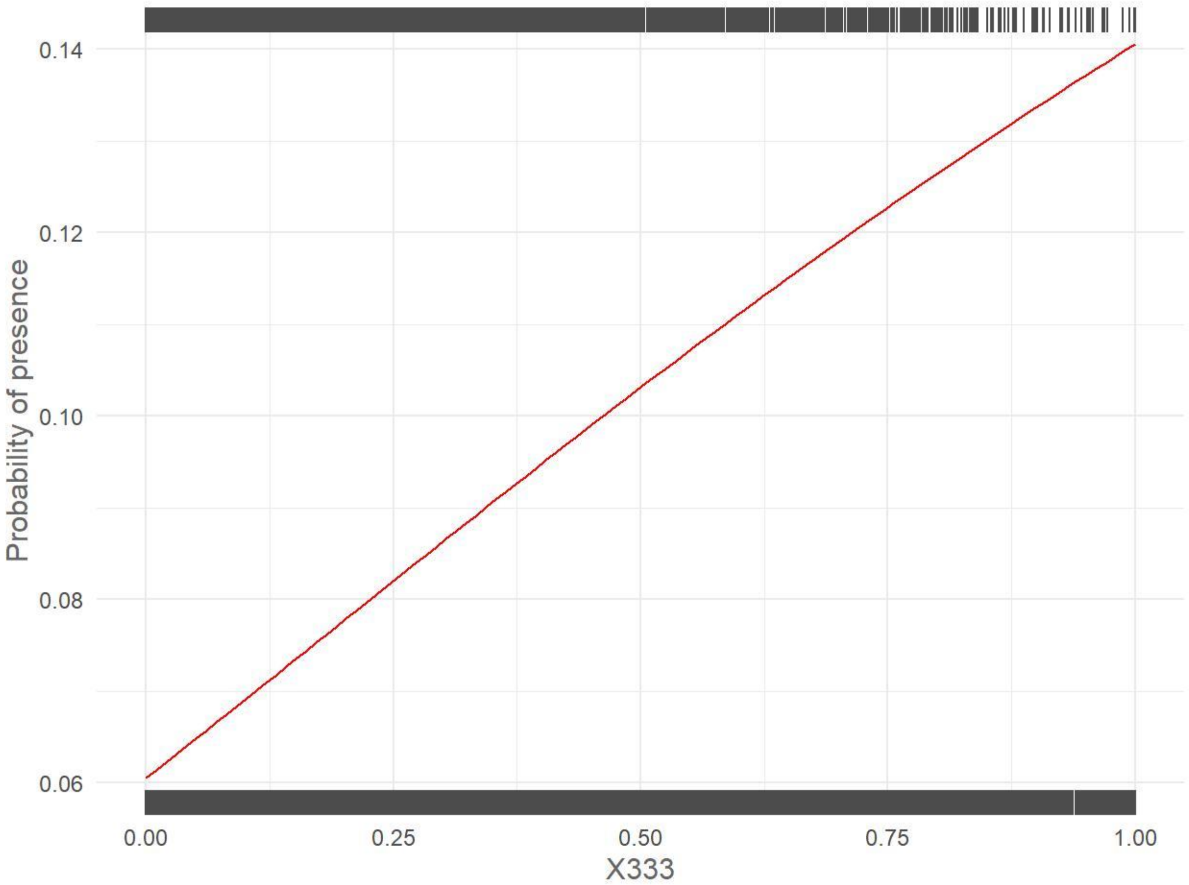
Probability of presence
0.14
0.12
0.10
0.08
0.06
0.00
0.25
0.50
0.75
1.00
X333

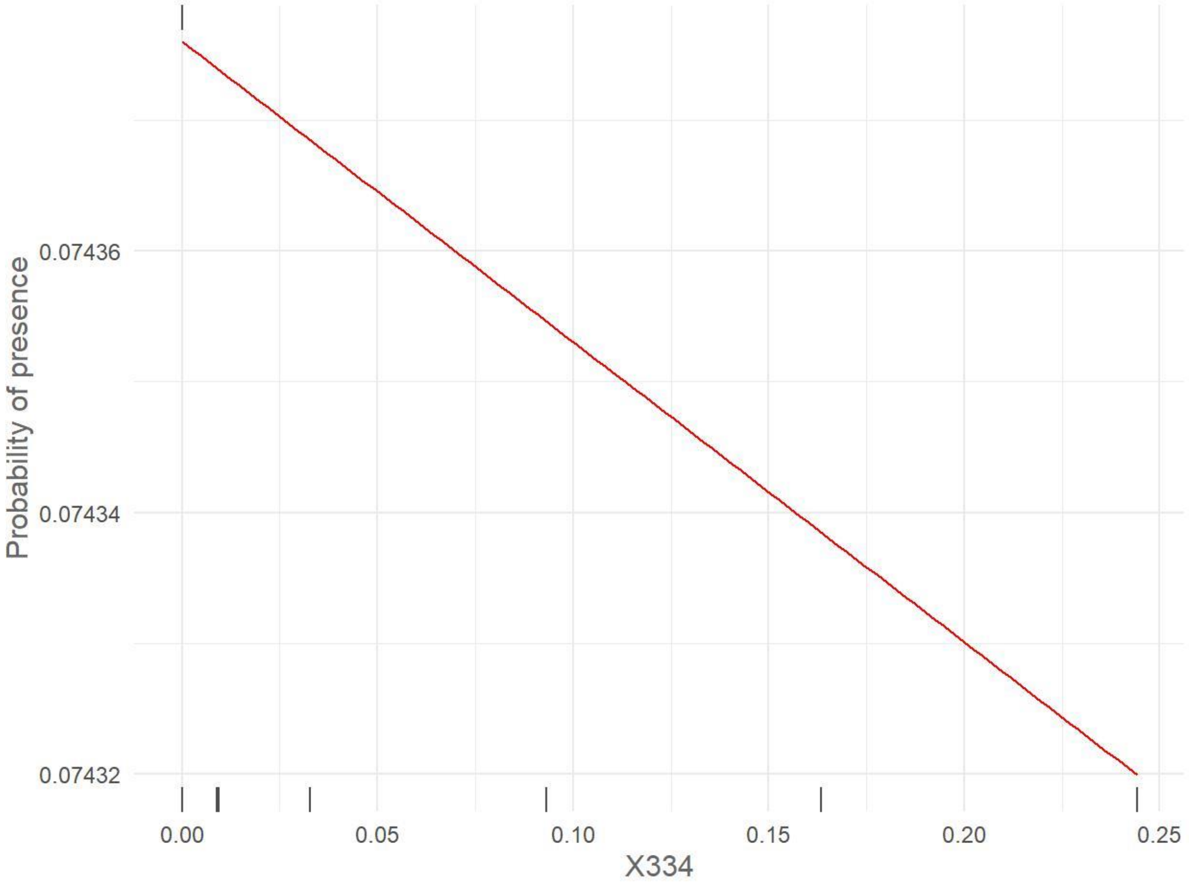
Probability of presence
0.07436
0.07434
0.07432
0.00
0.05
0.10
0.15
0.20
0.25
X334

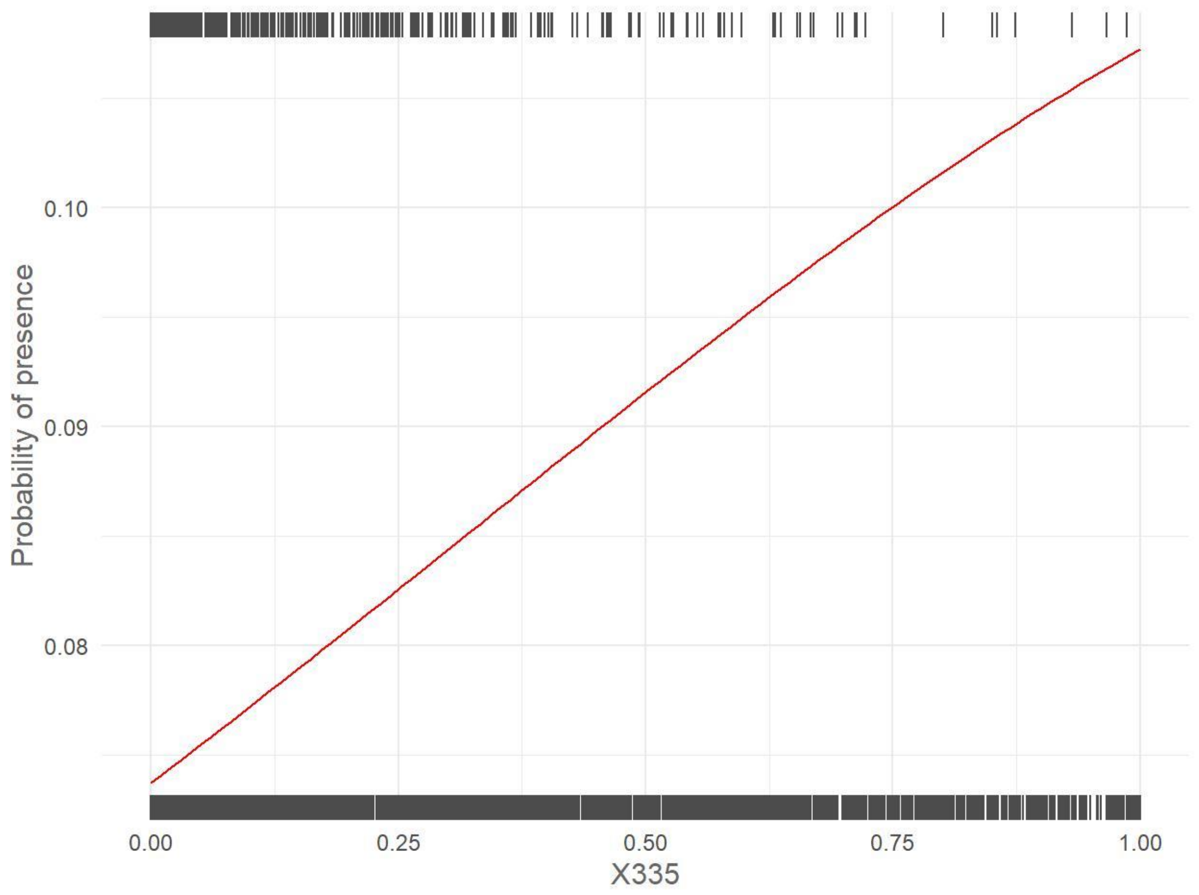

Probability of presence
0.10
0.09
0.08
0.00
0.25
0.50
0.75
1.00
X335

Figure S8. Species-habitat relationships according to the Artificial Neural Network model for white-winged snowfinch.

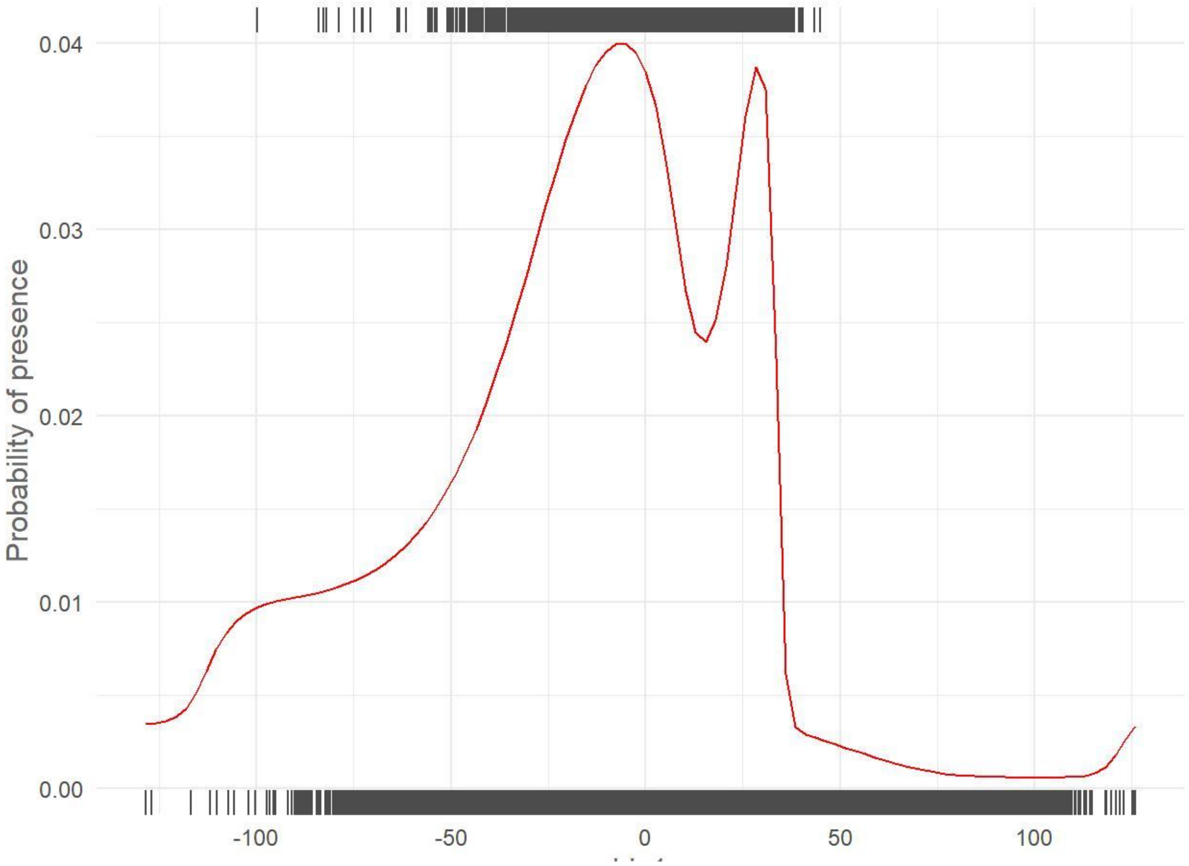
0.04
0.03
0.02
0.01
0.00
Probability of presence
-100
-50
0
50
100

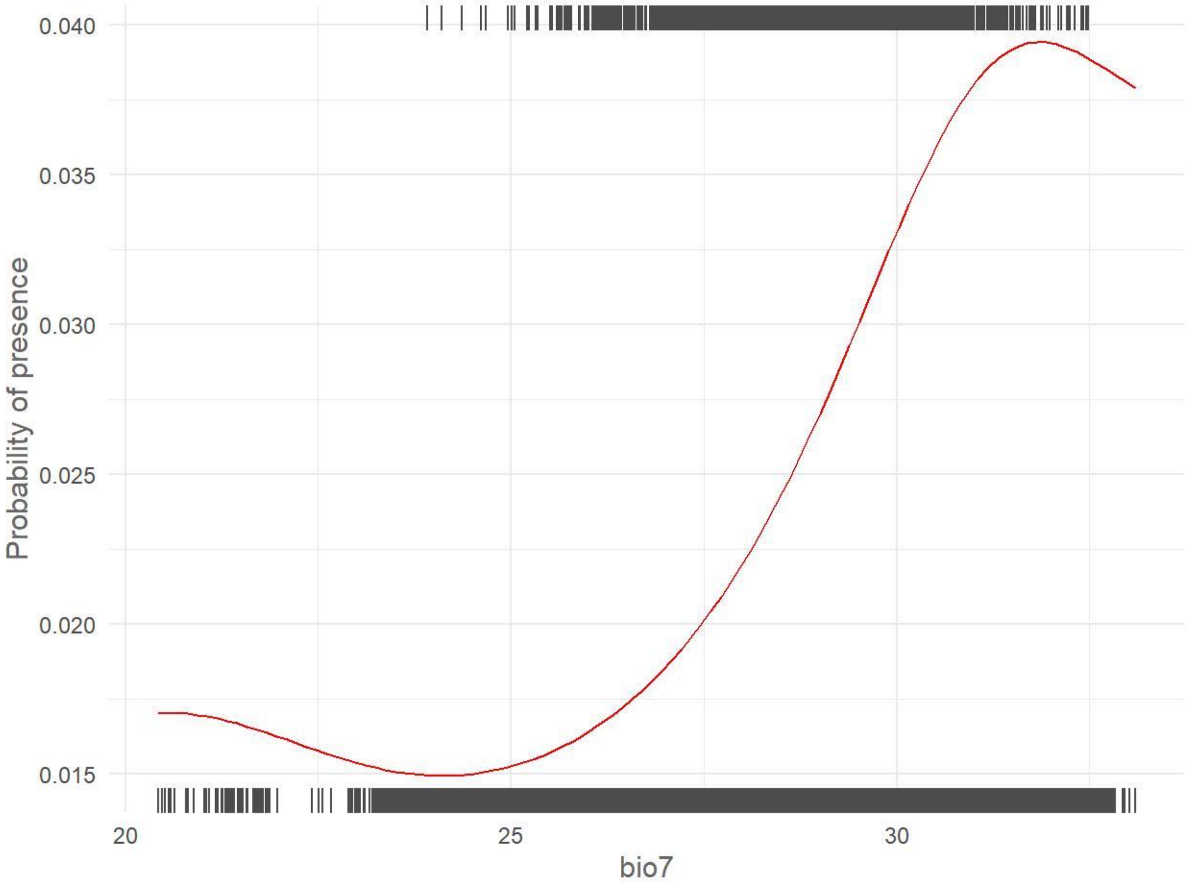
0.040
0.035
0.030
0.025
0.020
0.015
Probability of presence
20
25
30
bio7

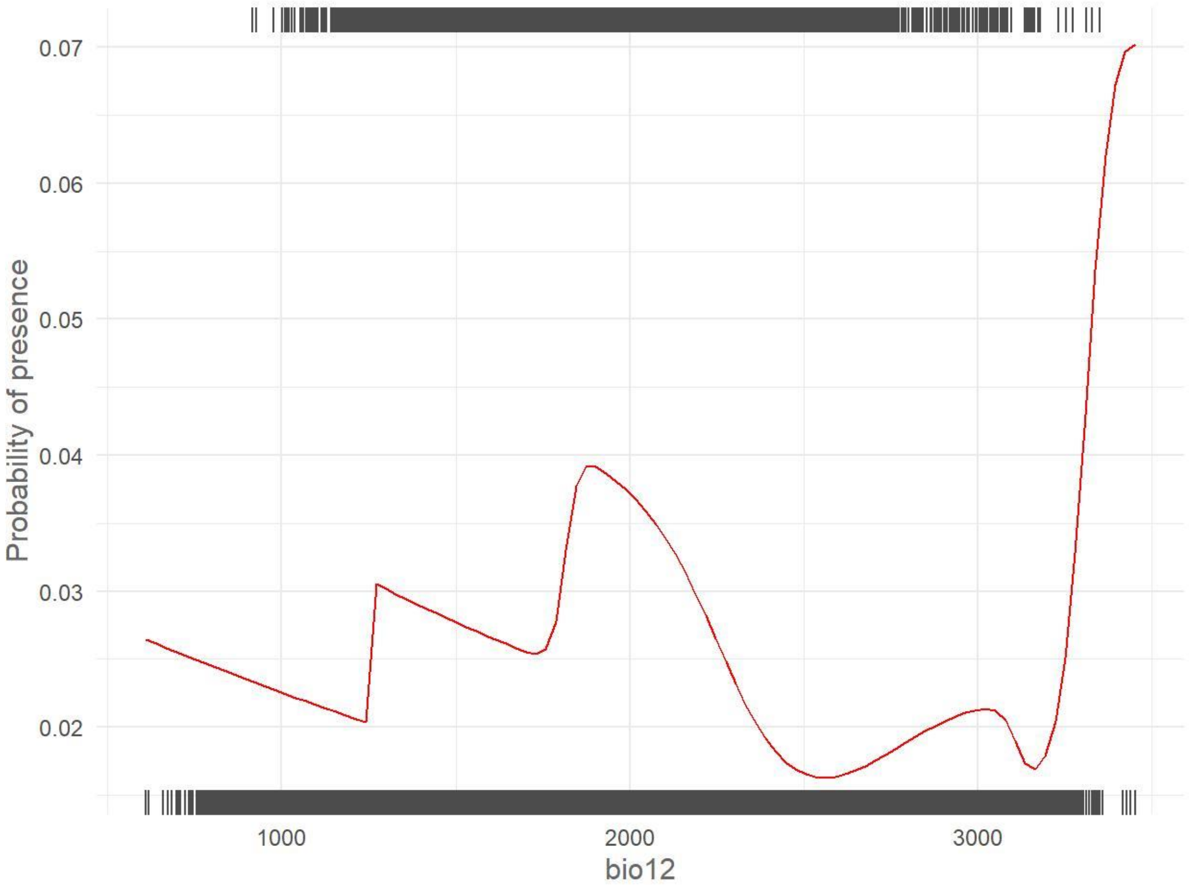
0.07
0.06
0.05
0.04
0.03
0.02
Probability of presence
1000
2000
3000
bio12

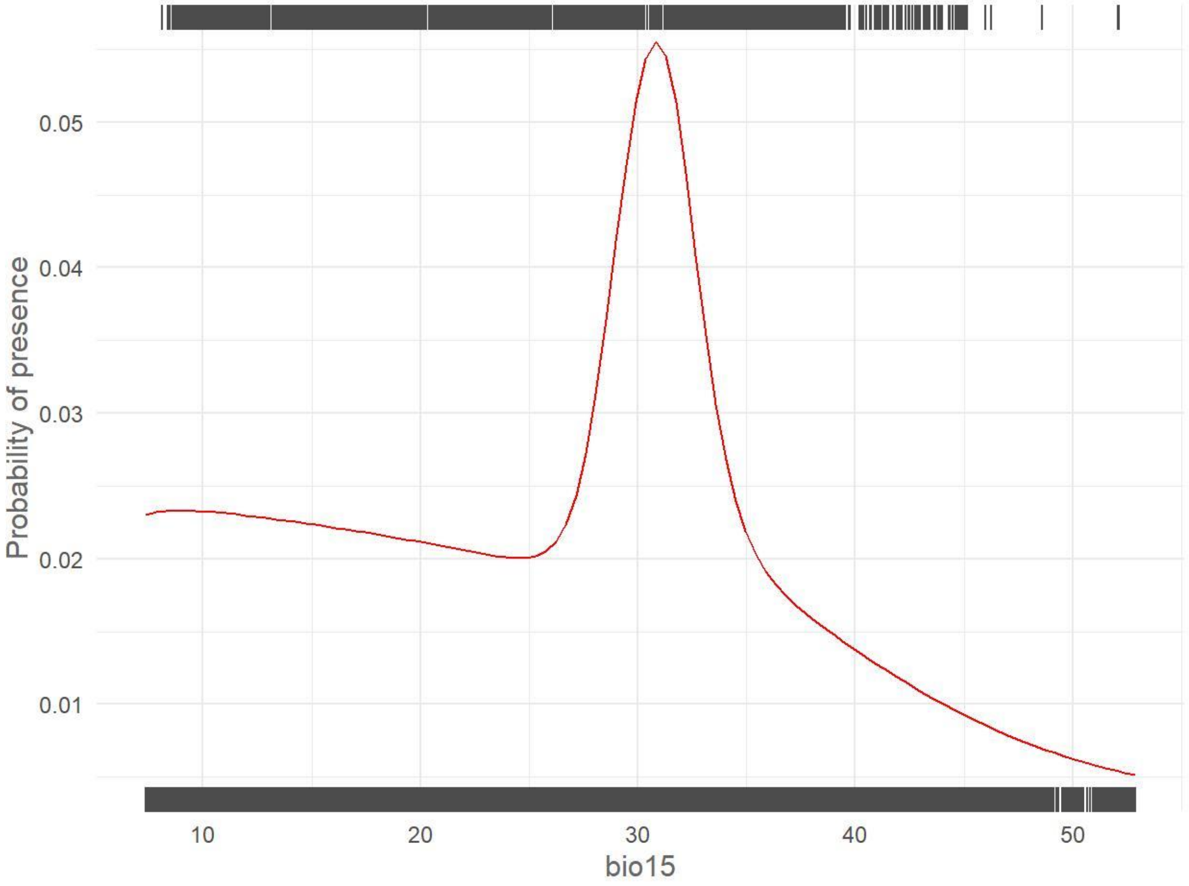
0.05
0.04
0.03
0.02
0.01
Probability of presence
10
20
30
40
50
bio15

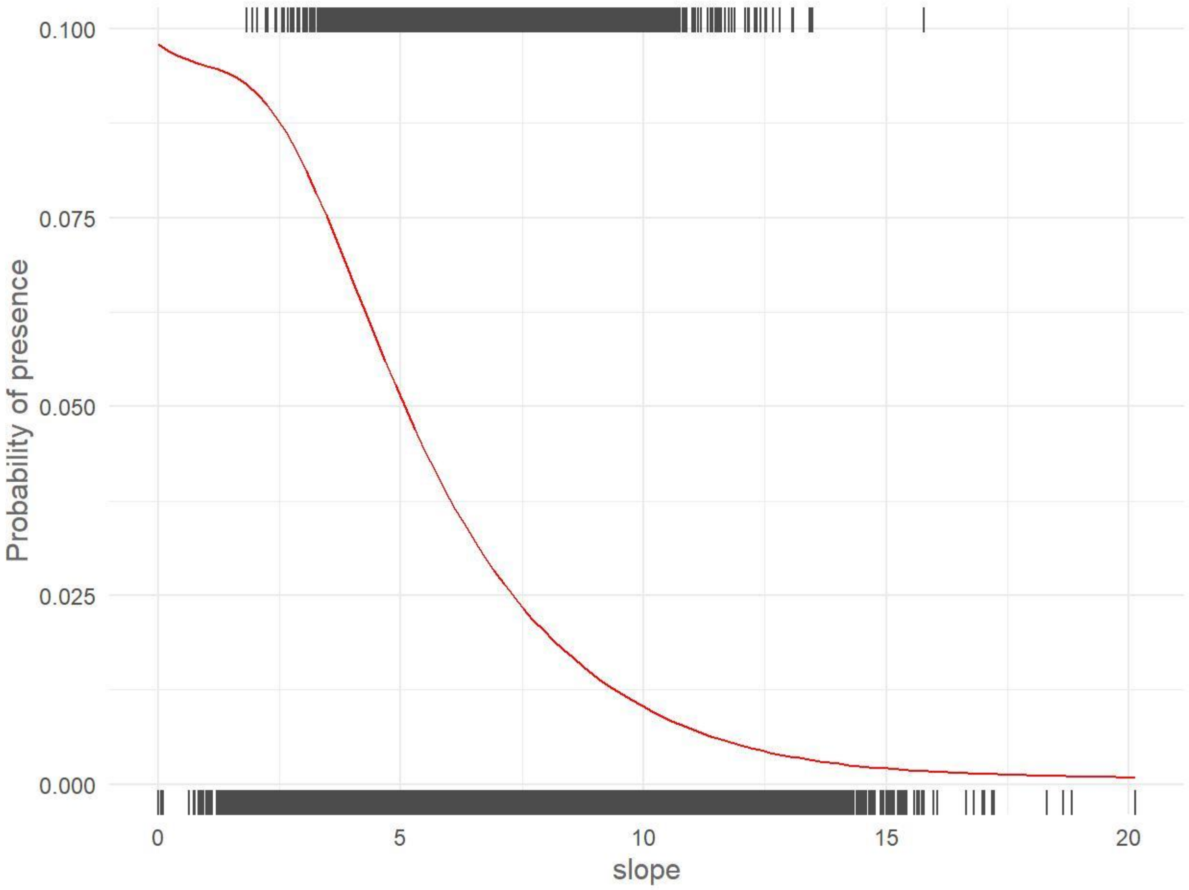

Probability of presence
0.100
0.075
0.050
0.025
0.000
0
5
10
15
20
slope

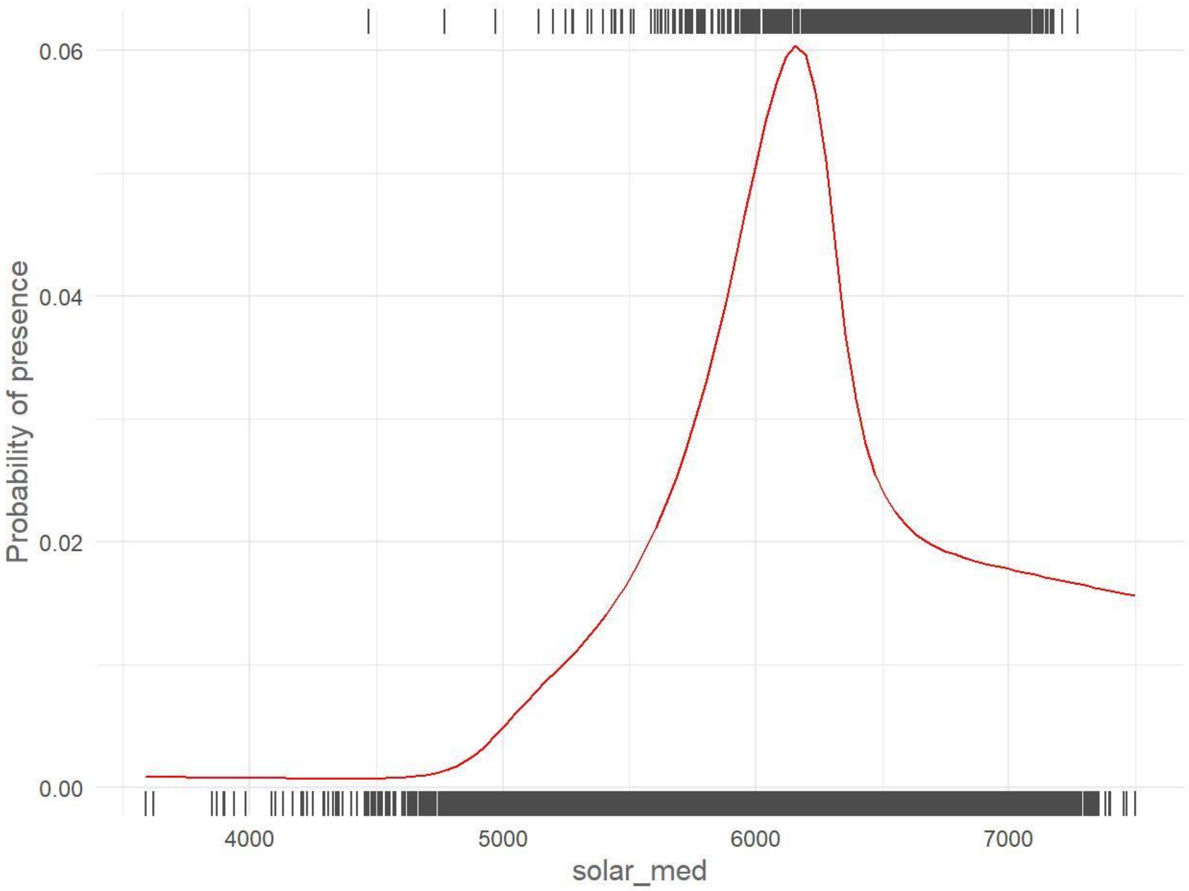

Probability of presence
0.06
0.04
0.02
0.00
4000
5000
6000
7000
solar_med

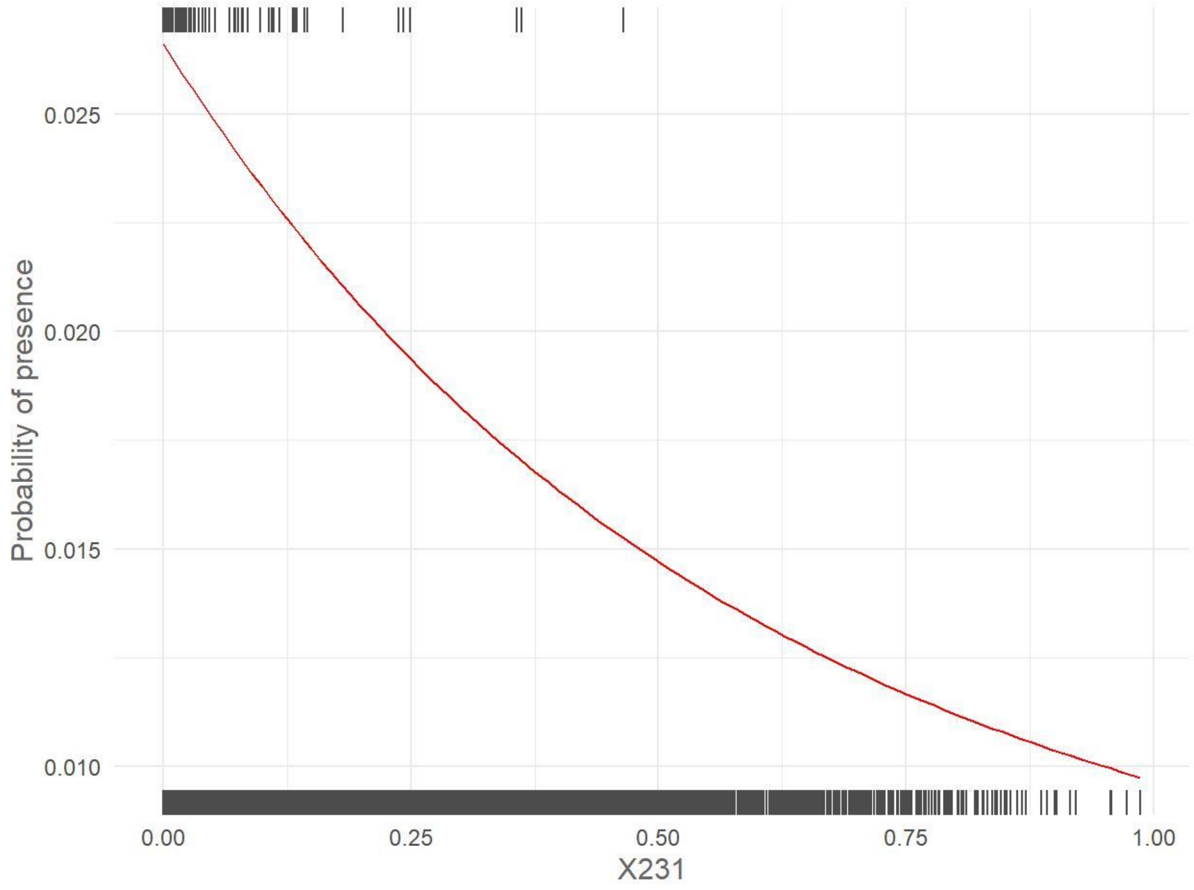

0.025
0.020
0.015
0.010
Probability of presence
0.00
0.25
0.50
0.75
1.00
X231

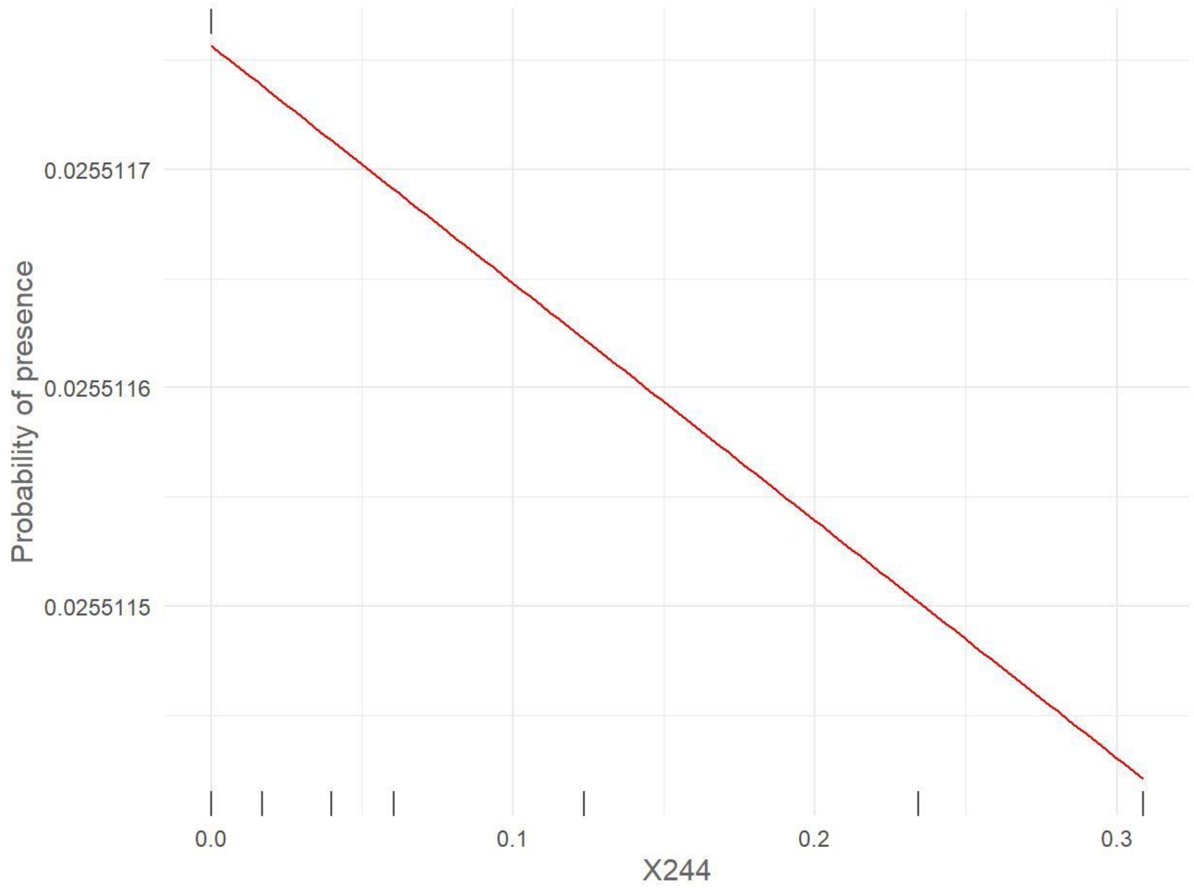

0.0255117
0.0255116
0.0255115
Probability of presence
0.0
0.1
0.2
0.3
X244

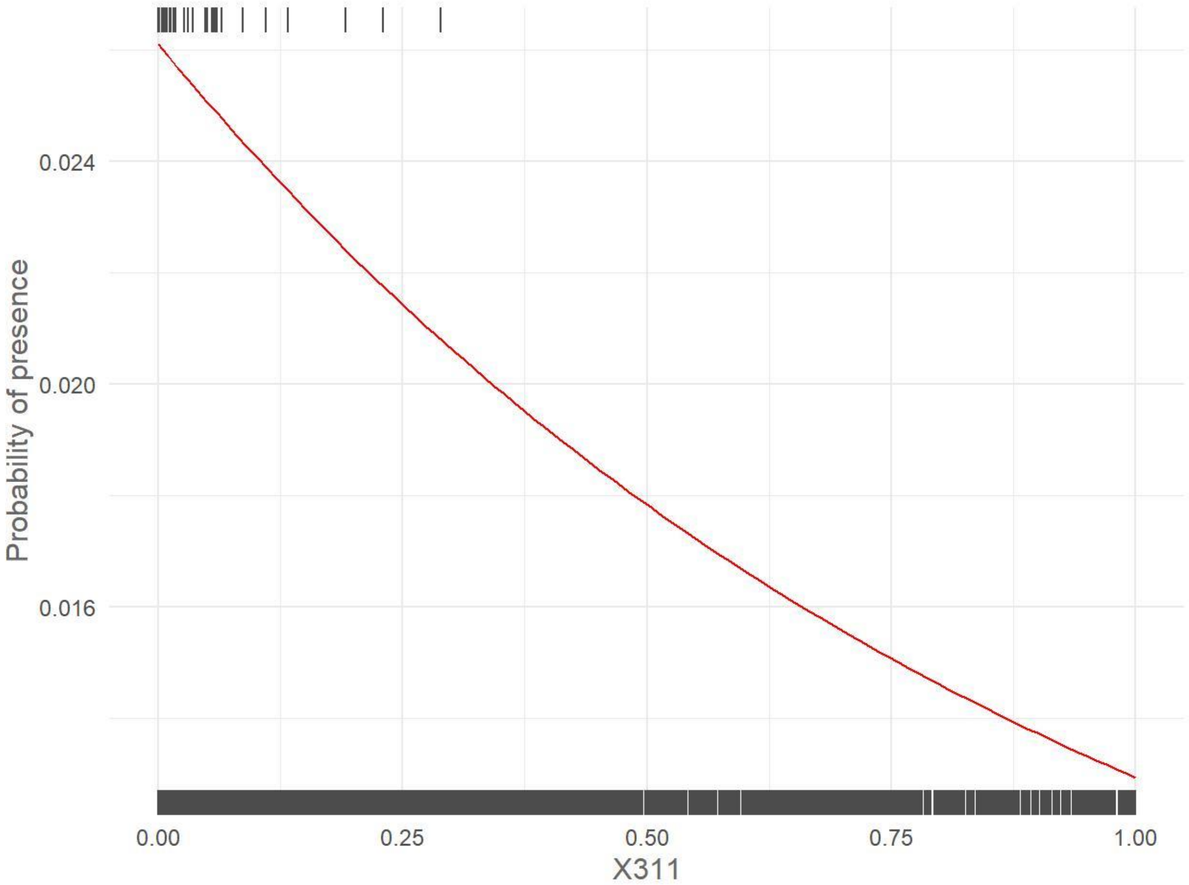

Probability of presence
0.024
0.020
0.016
0.00
0.25
0.50
0.75
1.00
X311

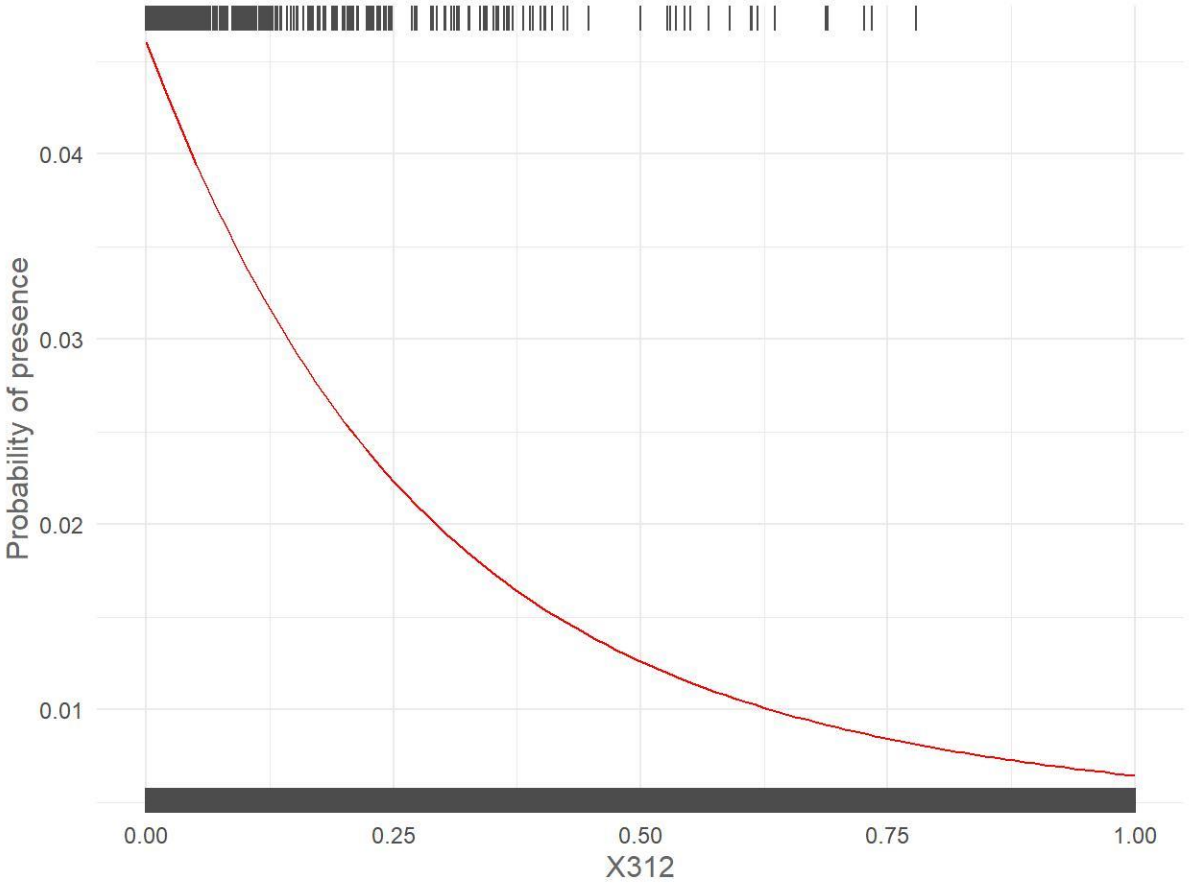

Probability of presence
0.04
0.03
0.02
0.01
0.00
0.25
0.50
0.75
1.00
X312

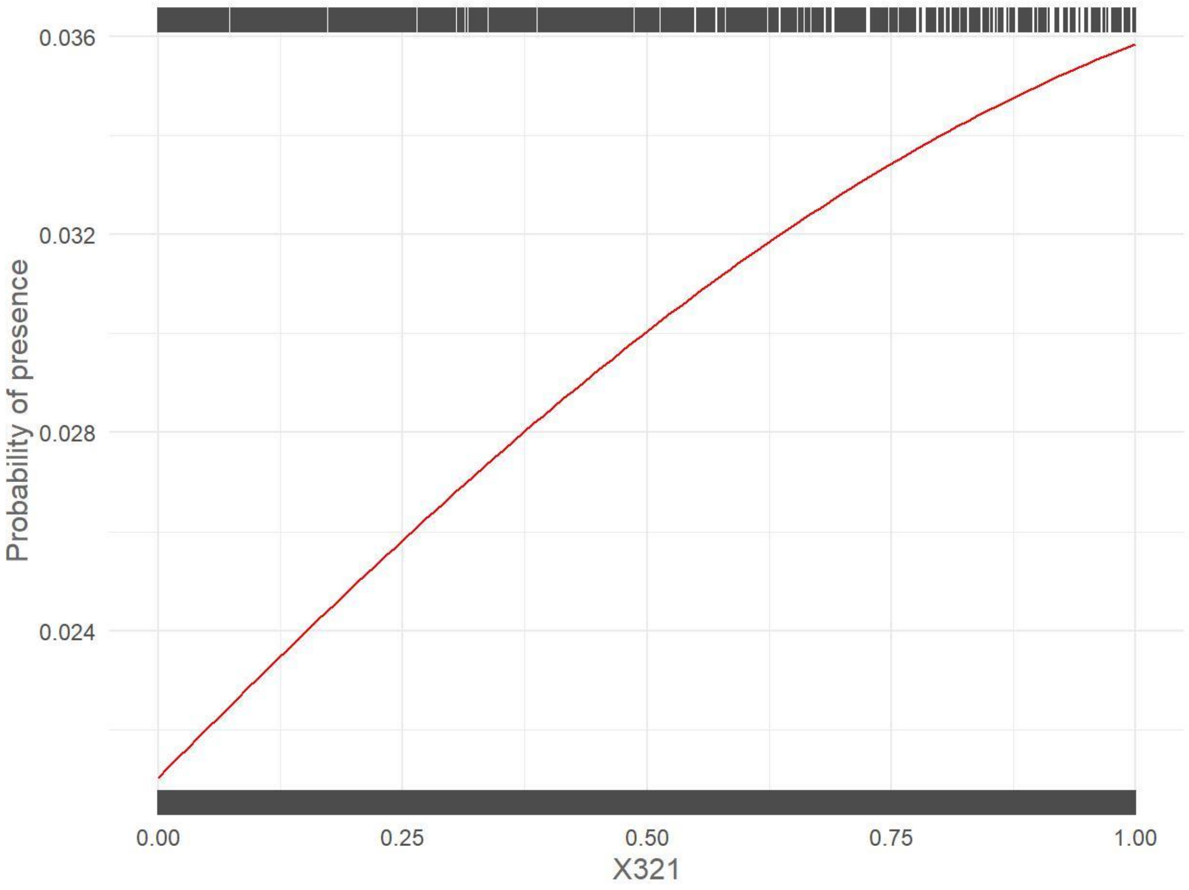
0.036
0.032
0.028
0.024
Probability of presence
0.00
0.25
0.50
0.75
1.00
X321

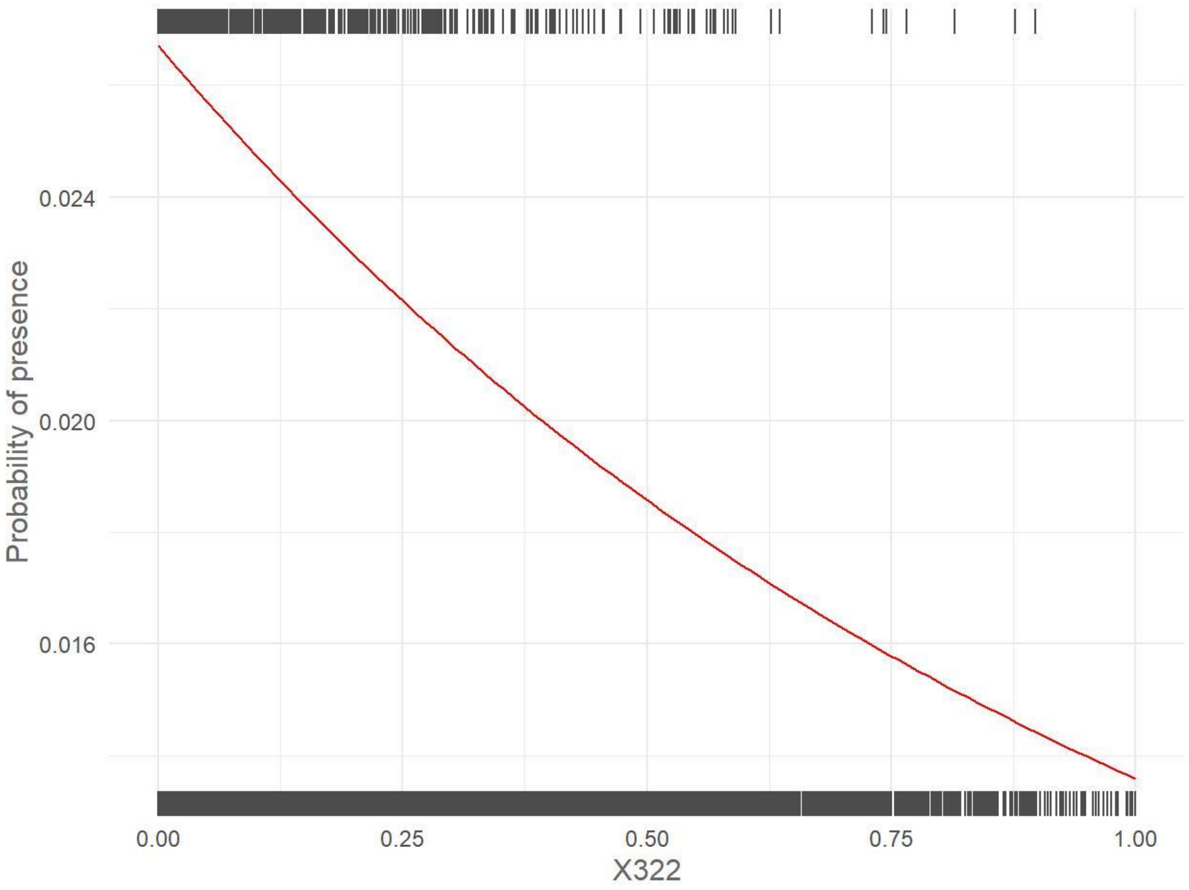
0.024
0.020
0.016
Probability of presence
0.00
0.25
0.50
0.75
1.00
X322

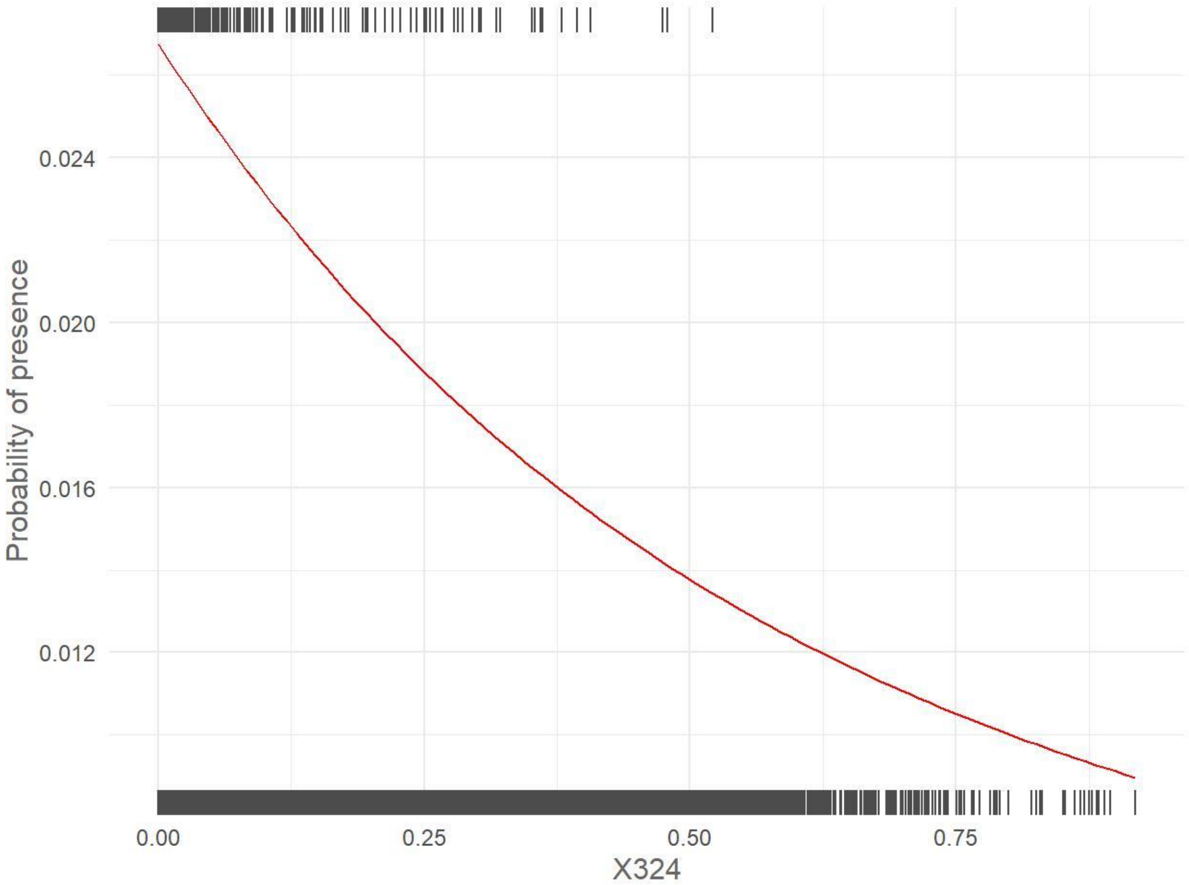
0.024
0.020
0.016
0.012
Probability of presence
0.00
0.25
0.50
0.75
X324

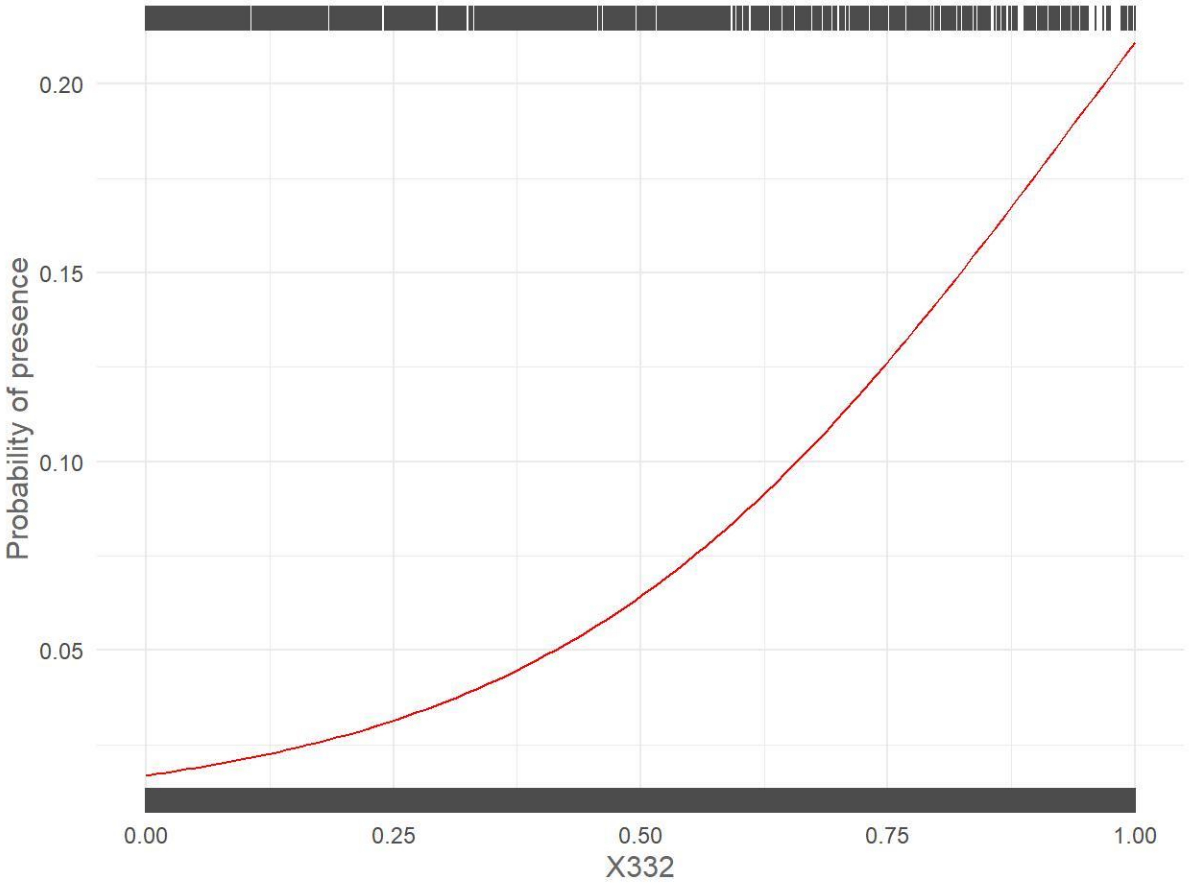
0.20
0.15
0.10
0.05
Probability of presence
0.00
0.25
0.50
0.75
1.00
X332

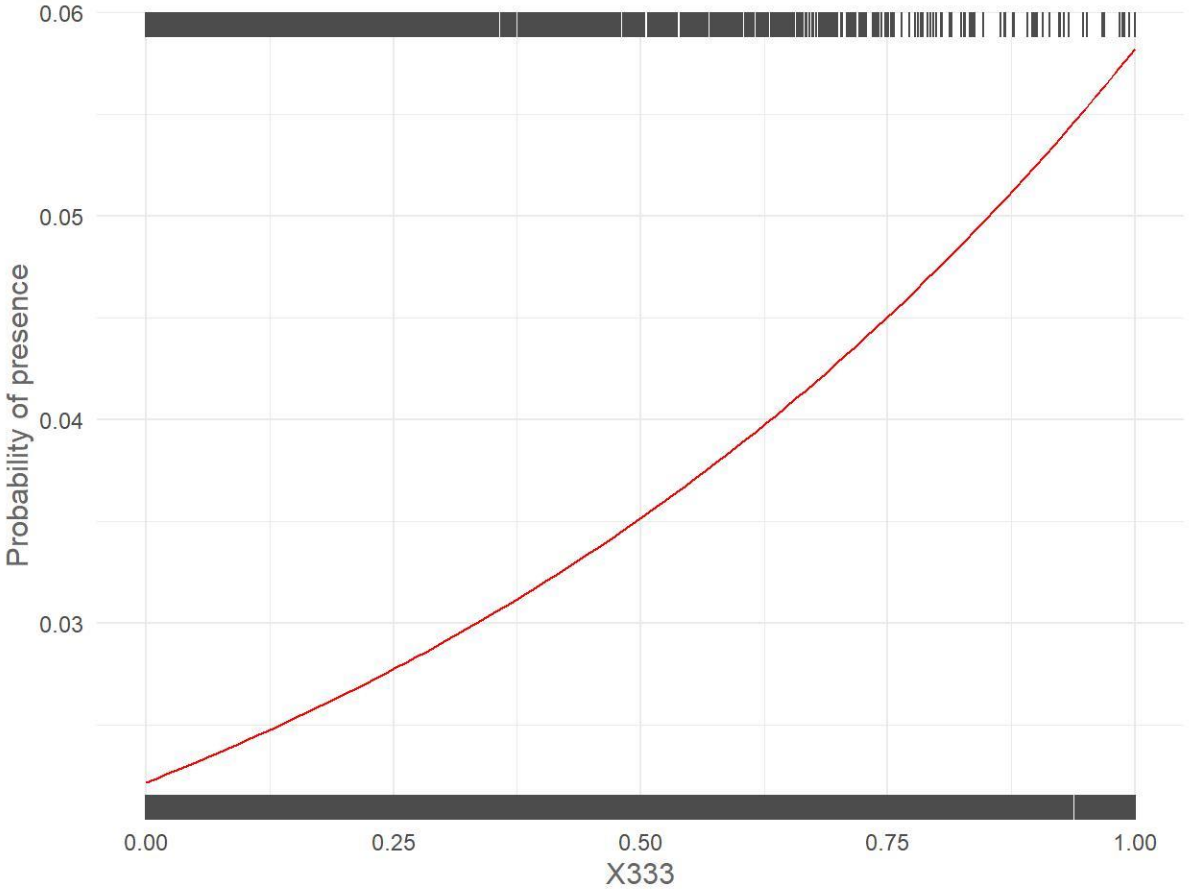
0.06
0.05
0.04
0.03
Probability of presence
0.00
0.25
0.50
0.75
1.00
X333

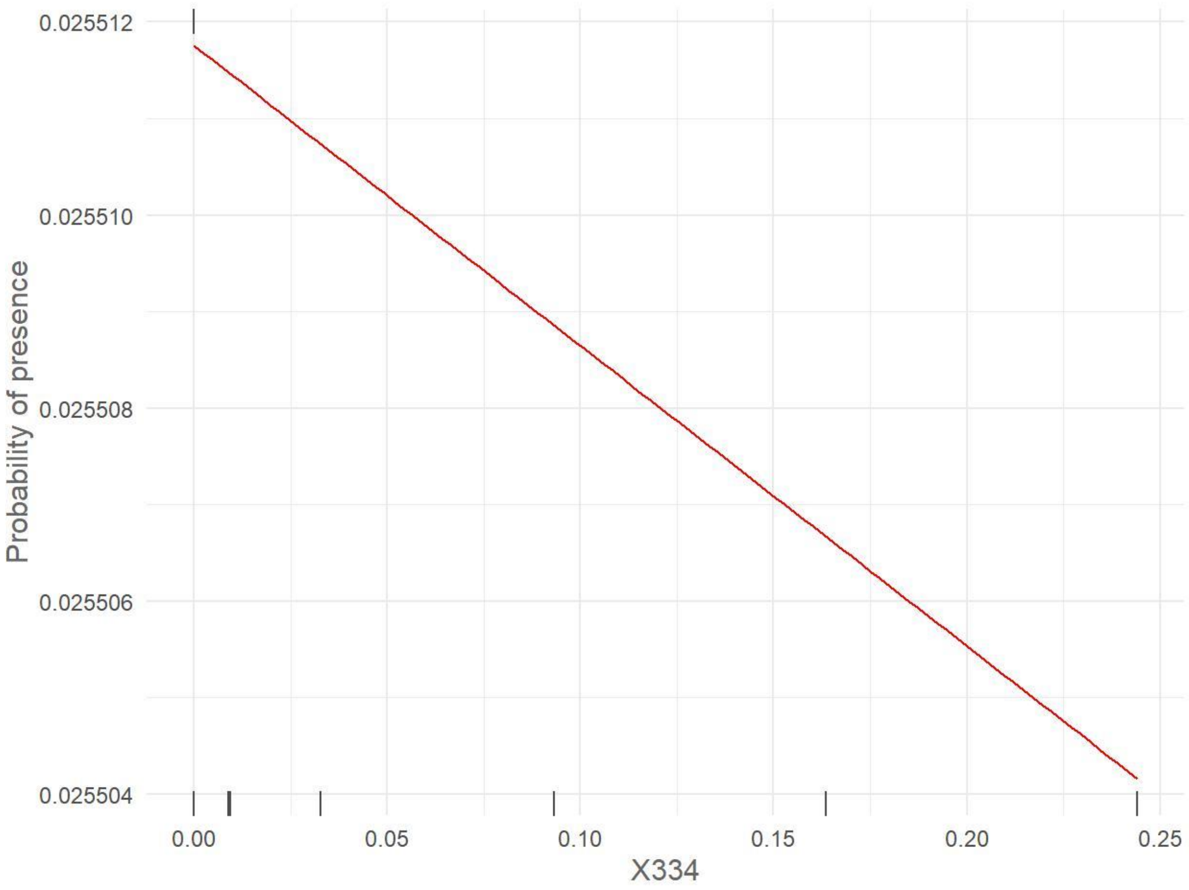
0.025512
0.025510
0.025508
0.025506
0.025504
Probability of presence
0.00
0.05
0.10
0.15
0.20
0.25
X334

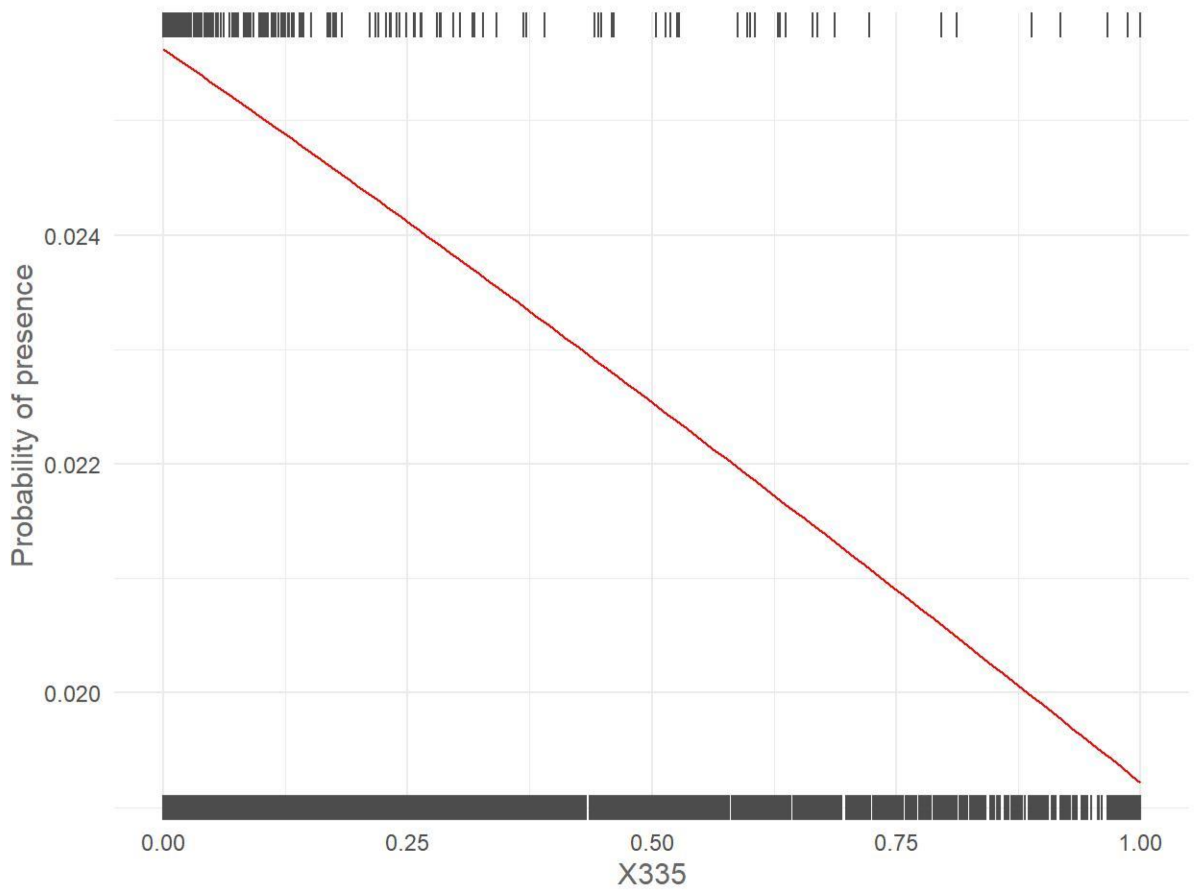
0.024
0.022
0.020
Probability of presence
0.00
0.25
0.50
0.75
1.00
X335

Figure S9. Species-habitat relationships according to the Boosted Regression Trees model for rock ptarmigan.

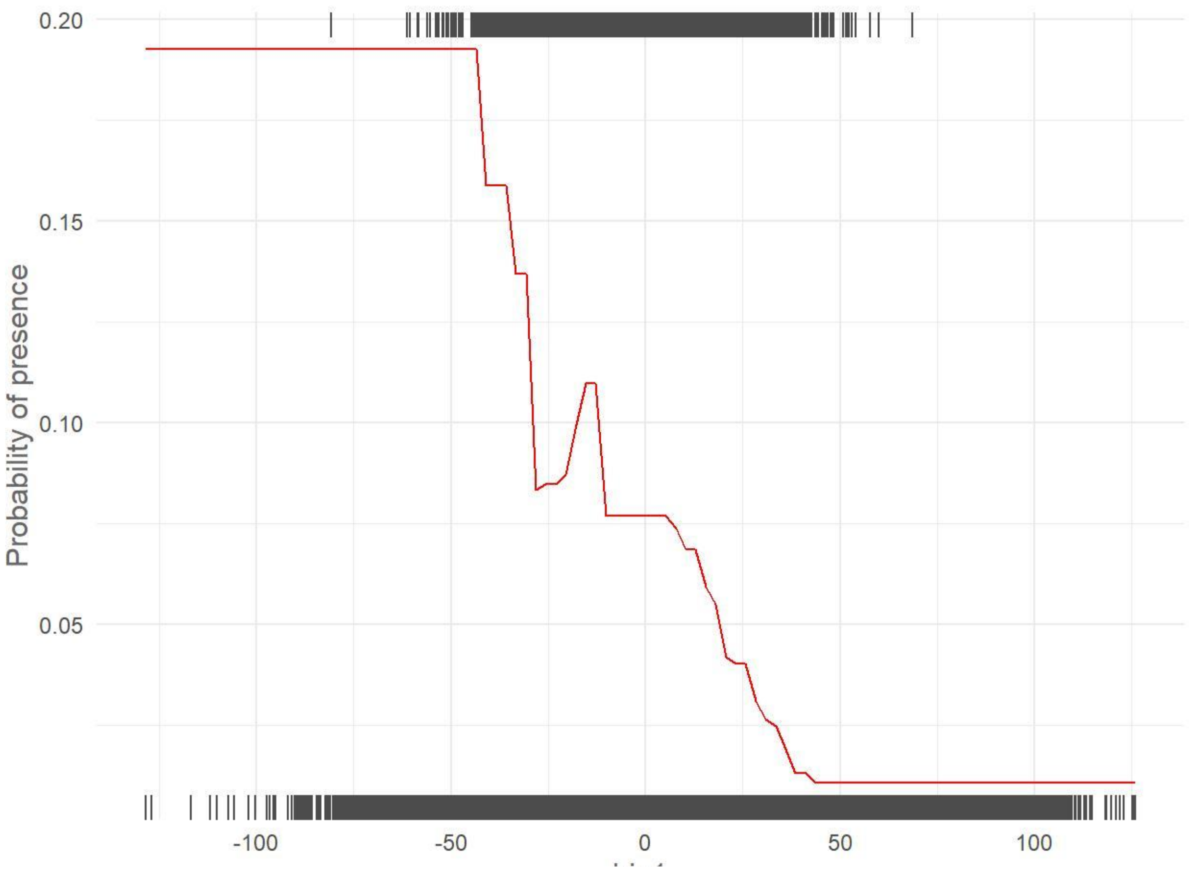
0.20
0.15
0.10
0.05
Probability of presence
-100
-50
0
50
100

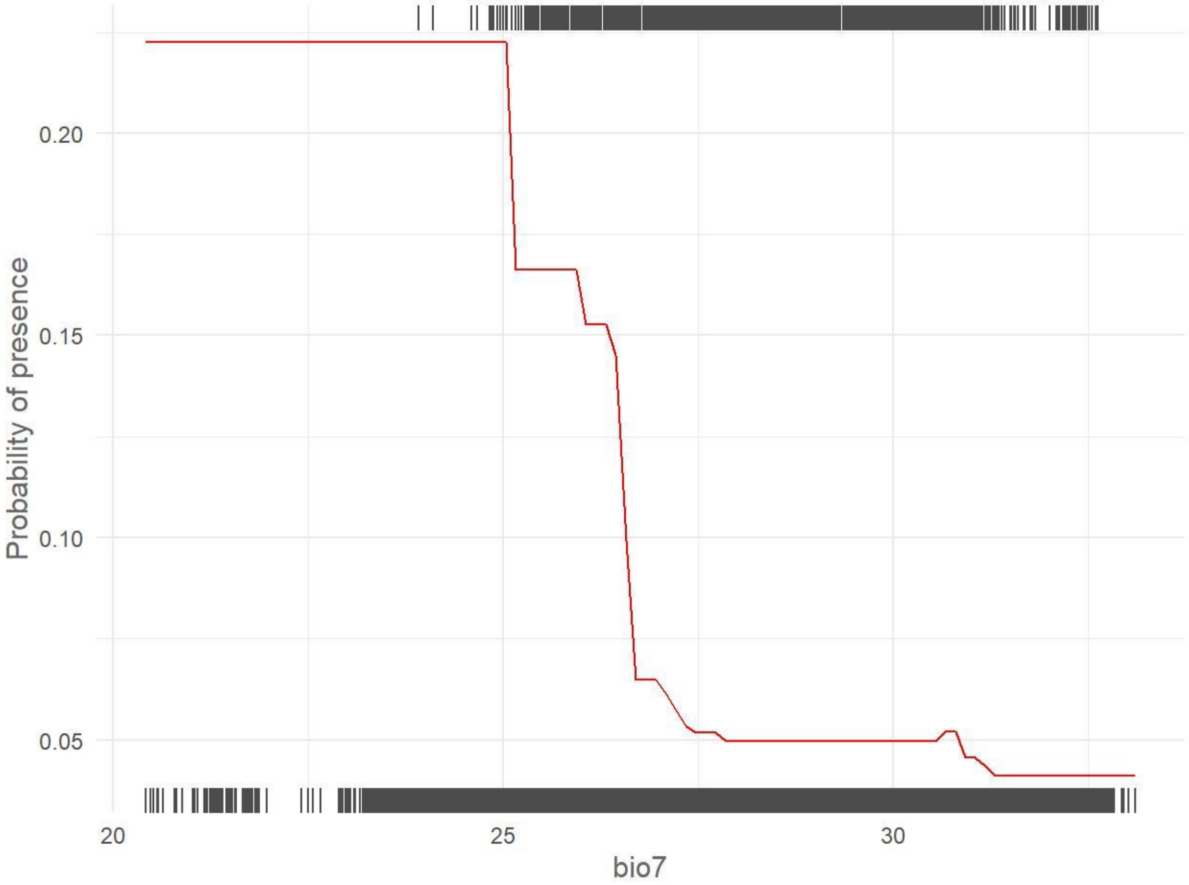
0.20
0.15
0.10
0.05
Probability of presence
20
25
30
bio7

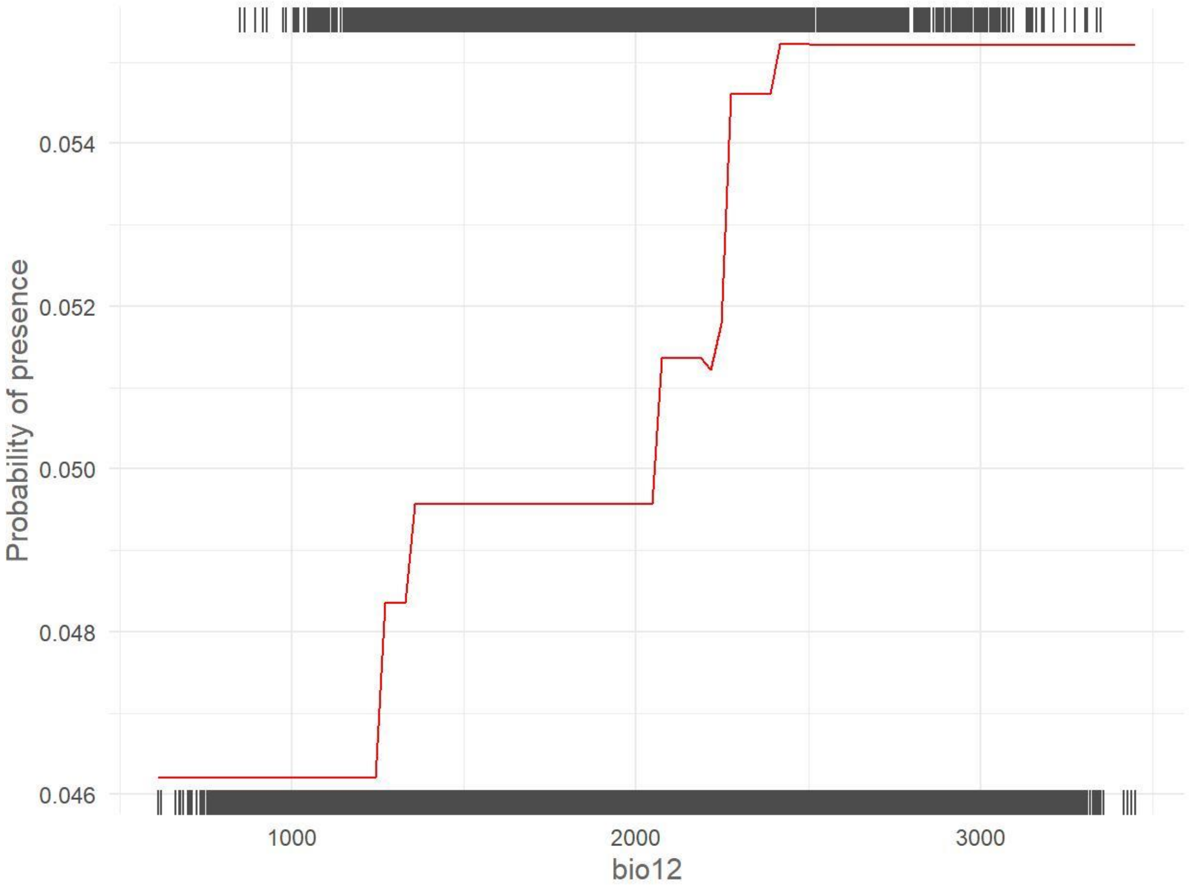

0.054
0.052
0.050
0.048
0.046
Probability of presence
1000
2000
3000
bio12

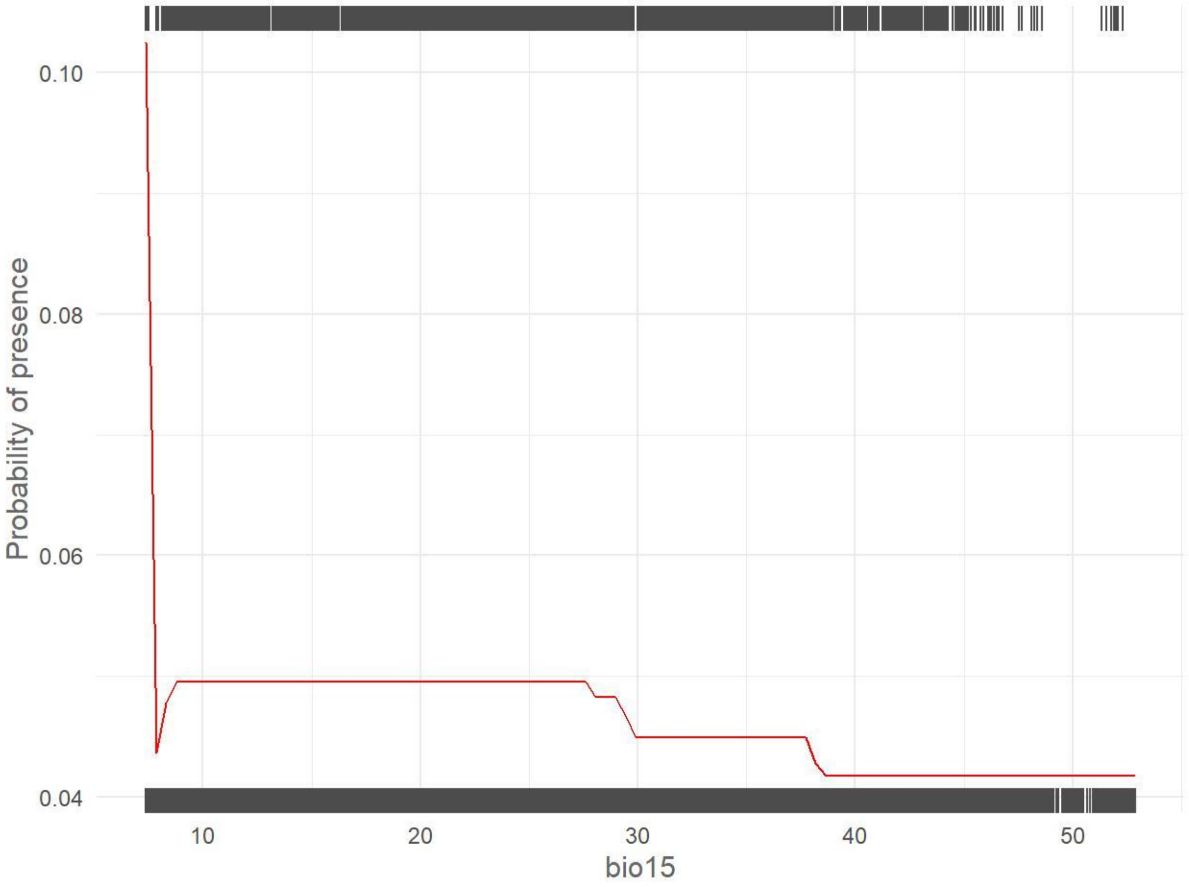

0.10
0.08
0.06
0.04
Probability of presence
10
20
30
40
50
bio15

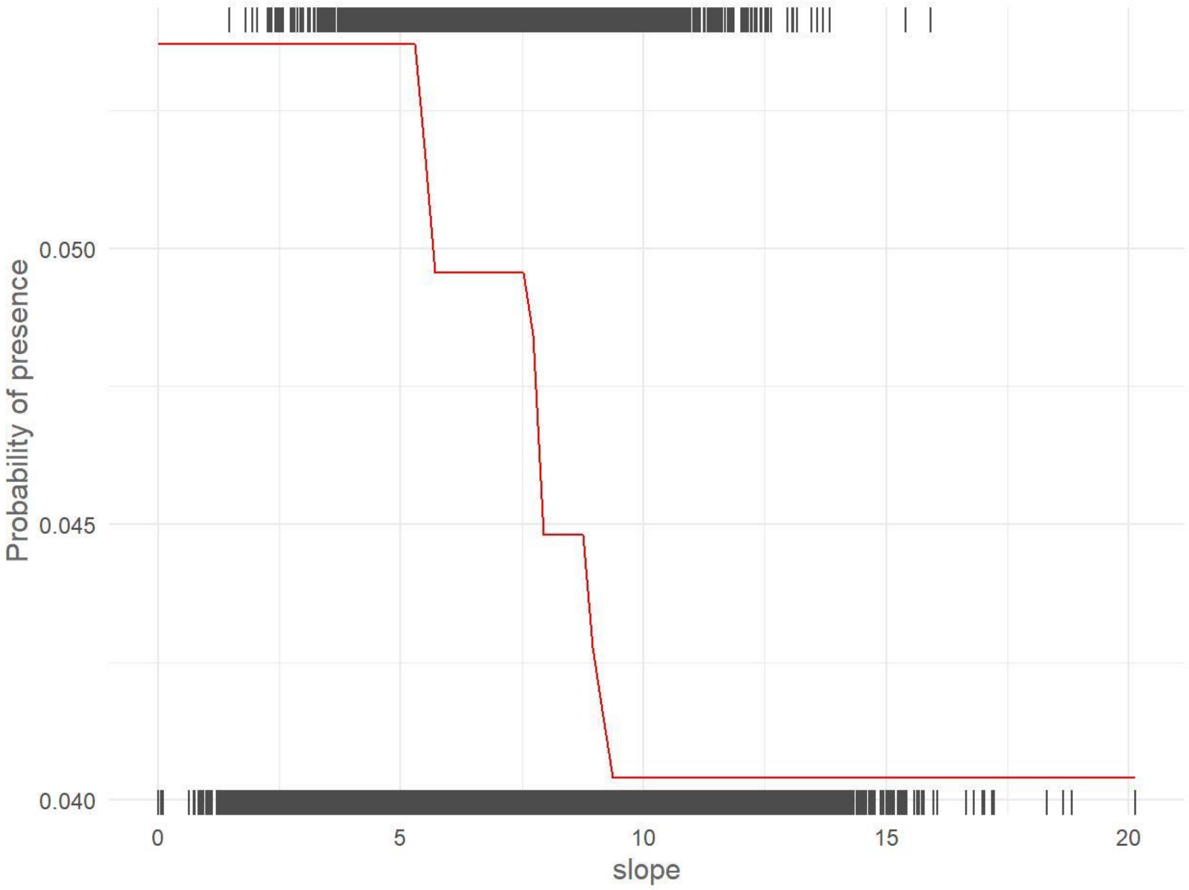
Probability of presence
0.050
0.045
0.040
0
5
10
15
20
slope

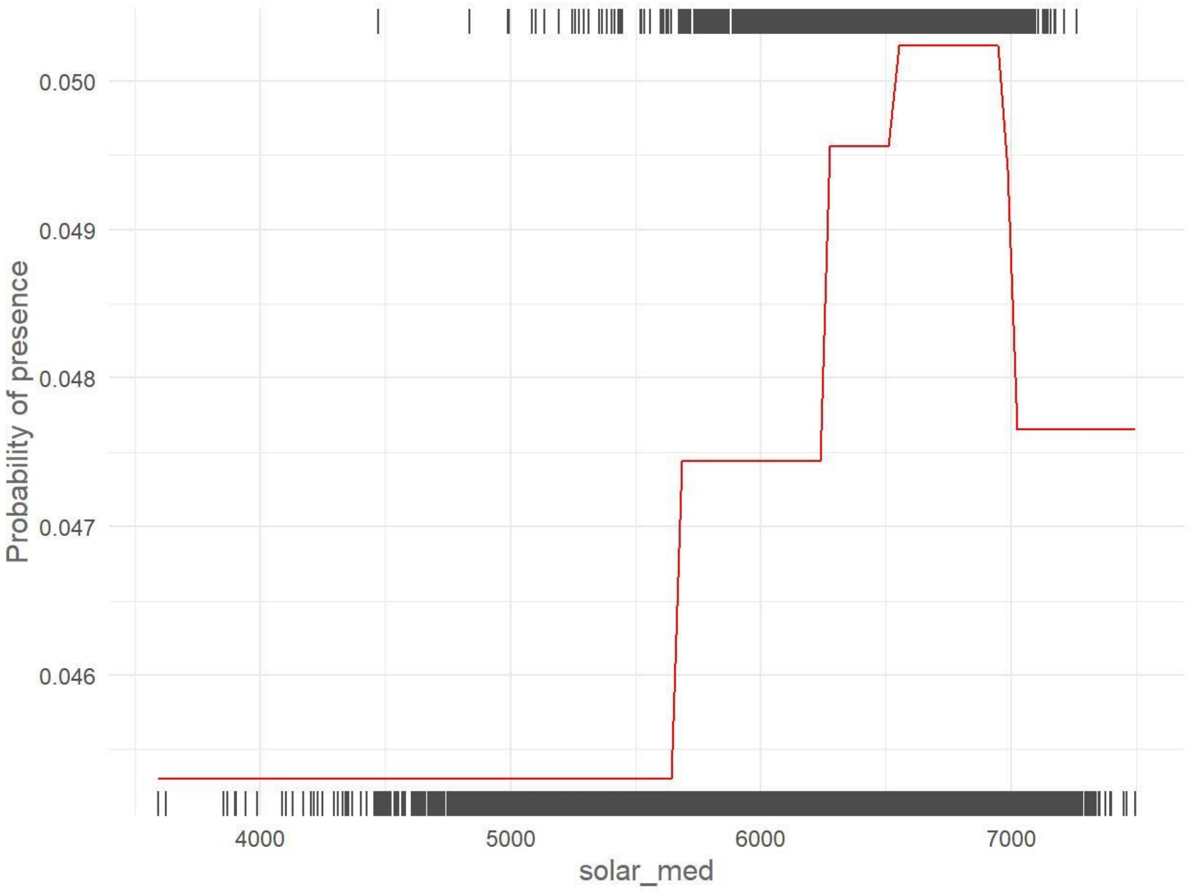
Probability of presence
0.050
0.049
0.048
0.047
0.046
4000
5000
6000
7000
solar_med

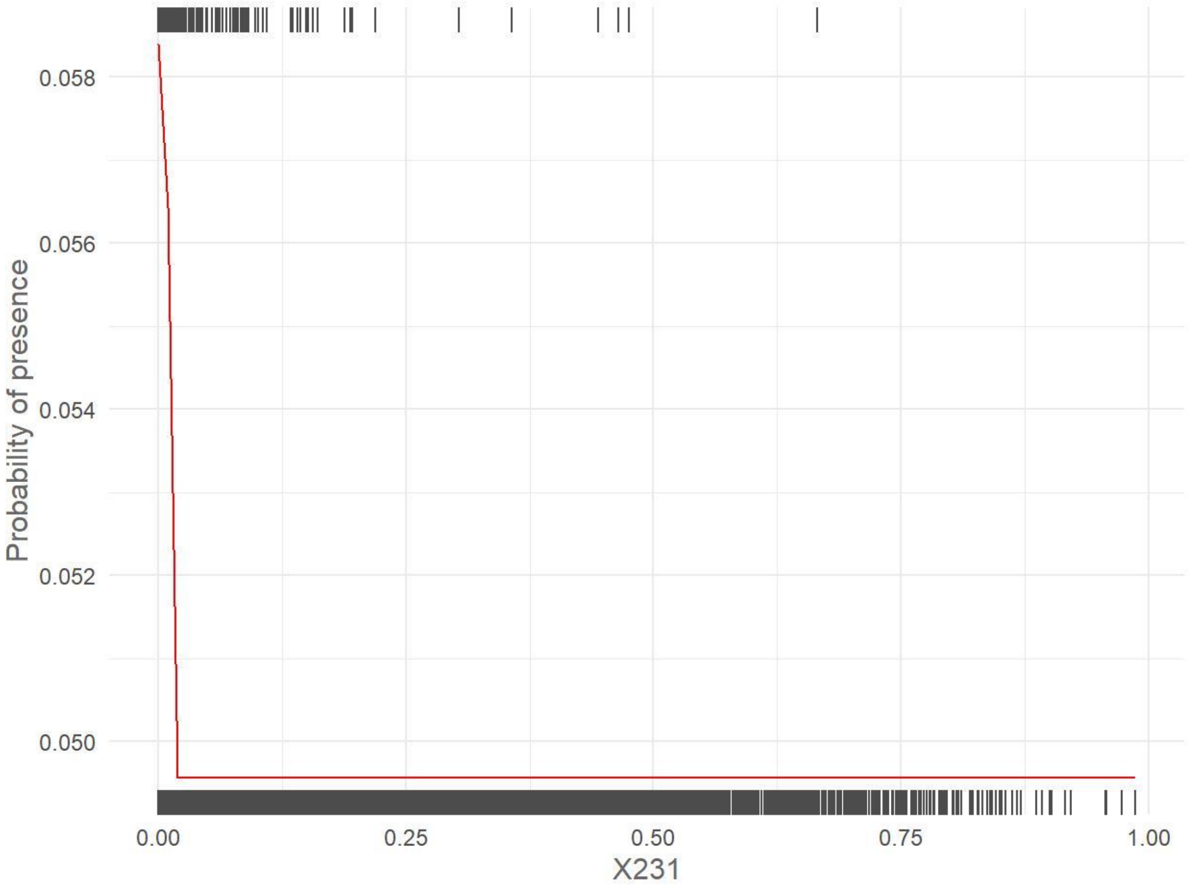
0.058
0.056
0.054
0.052
0.050
Probability of presence
0.00
0.25
0.50
0.75
1.00
X231

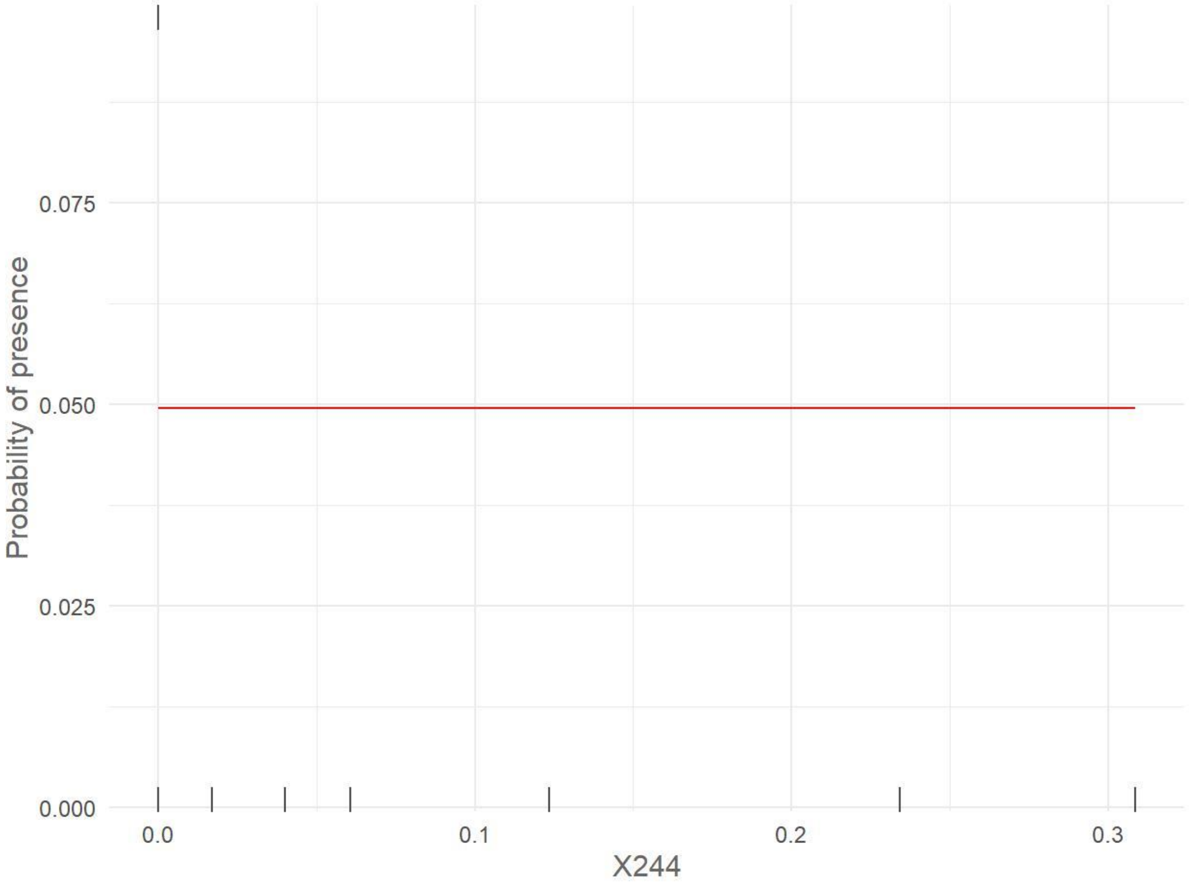
0.075
0.050
0.025
0.000
Probability of presence
0.0
0.1
0.2
0.3
X244

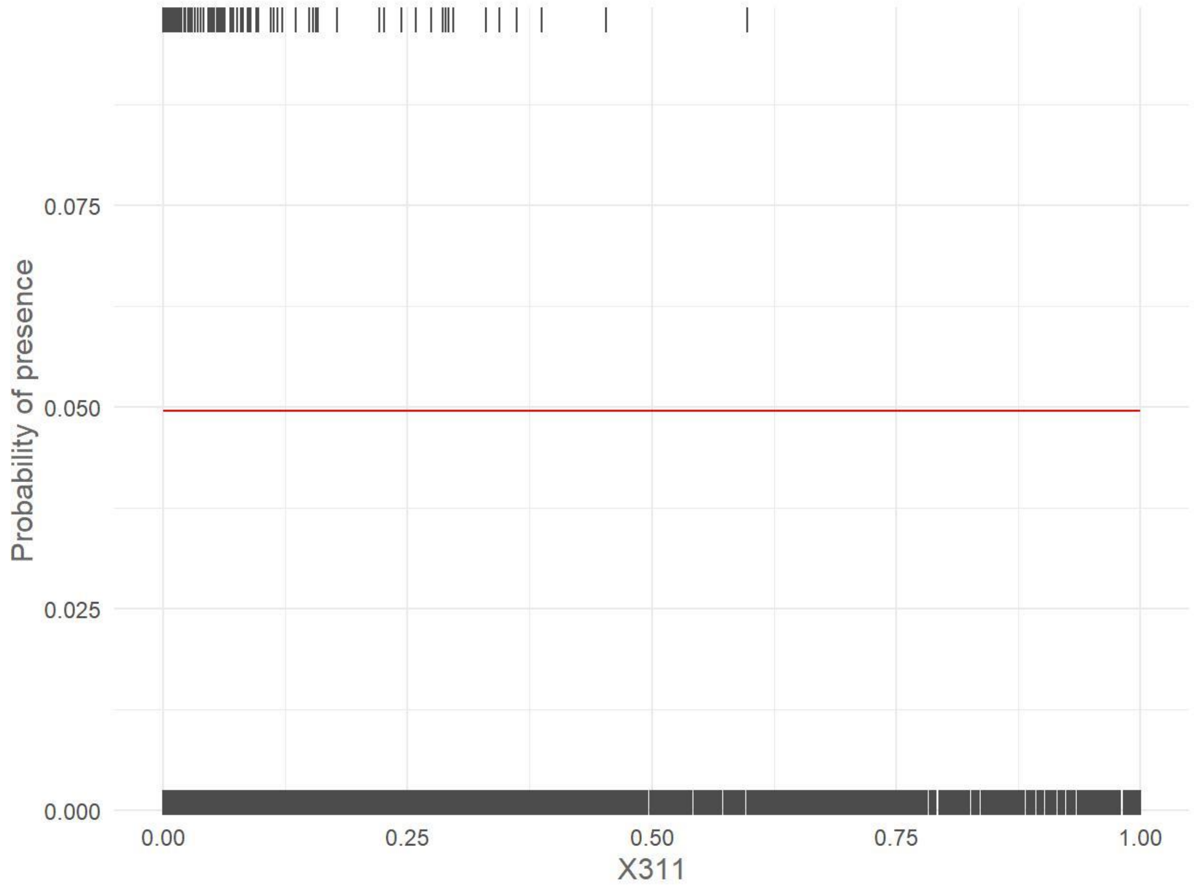
0.075
0.050
0.025
0.000
Probability of presence
0.00
0.25
0.50
0.75
1.00
X311

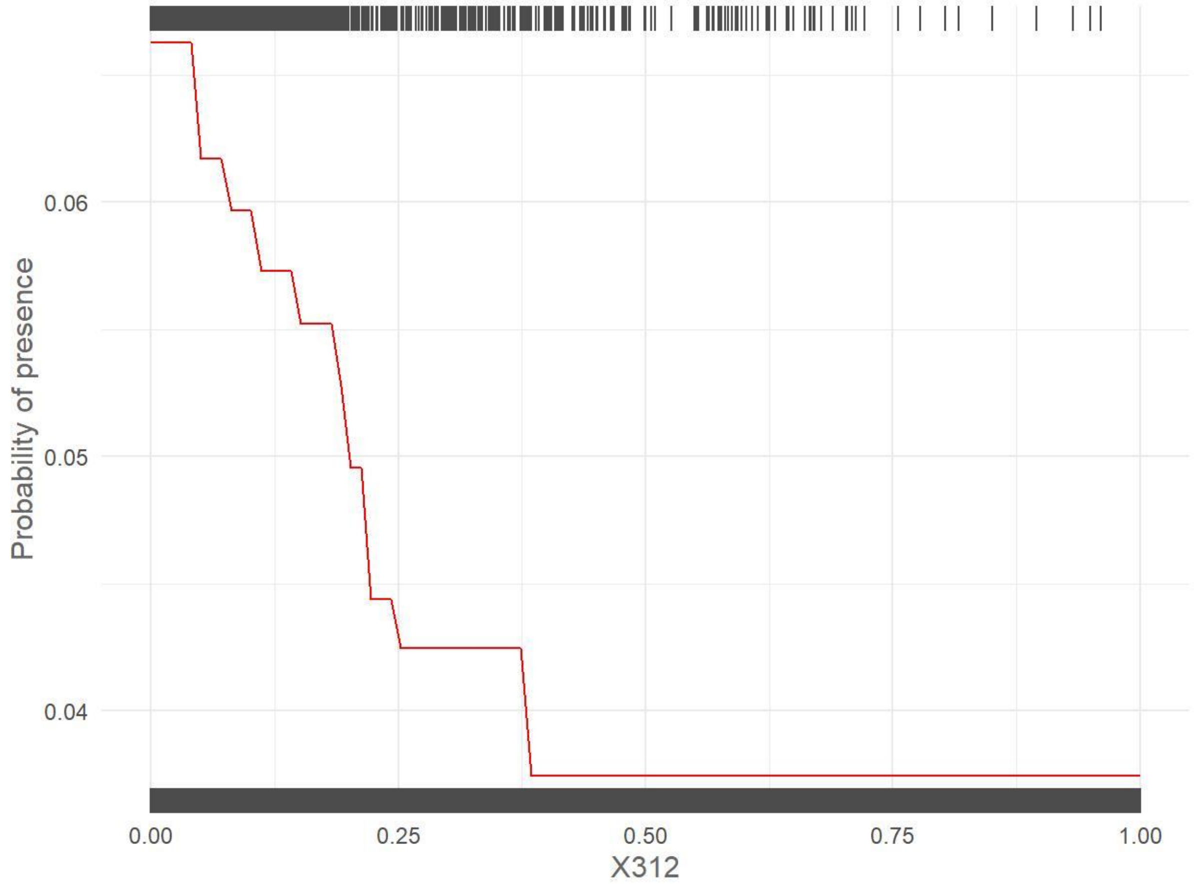
0.06
0.05
0.04
Probability of presence
0.00
0.25
0.50
0.75
1.00
X312

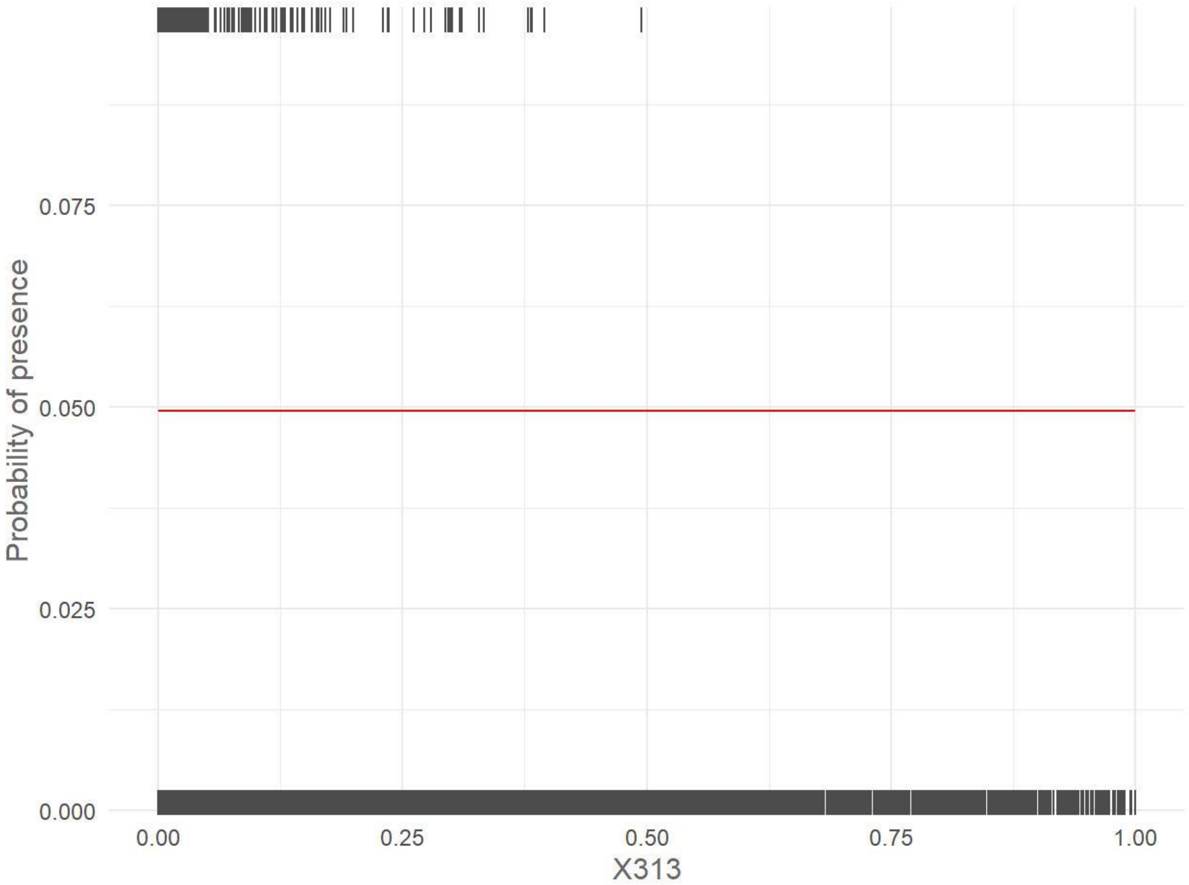
Probability of presence
0.075
0.050
0.025
0.000
0.00
0.25
0.50
0.75
1.00
X313

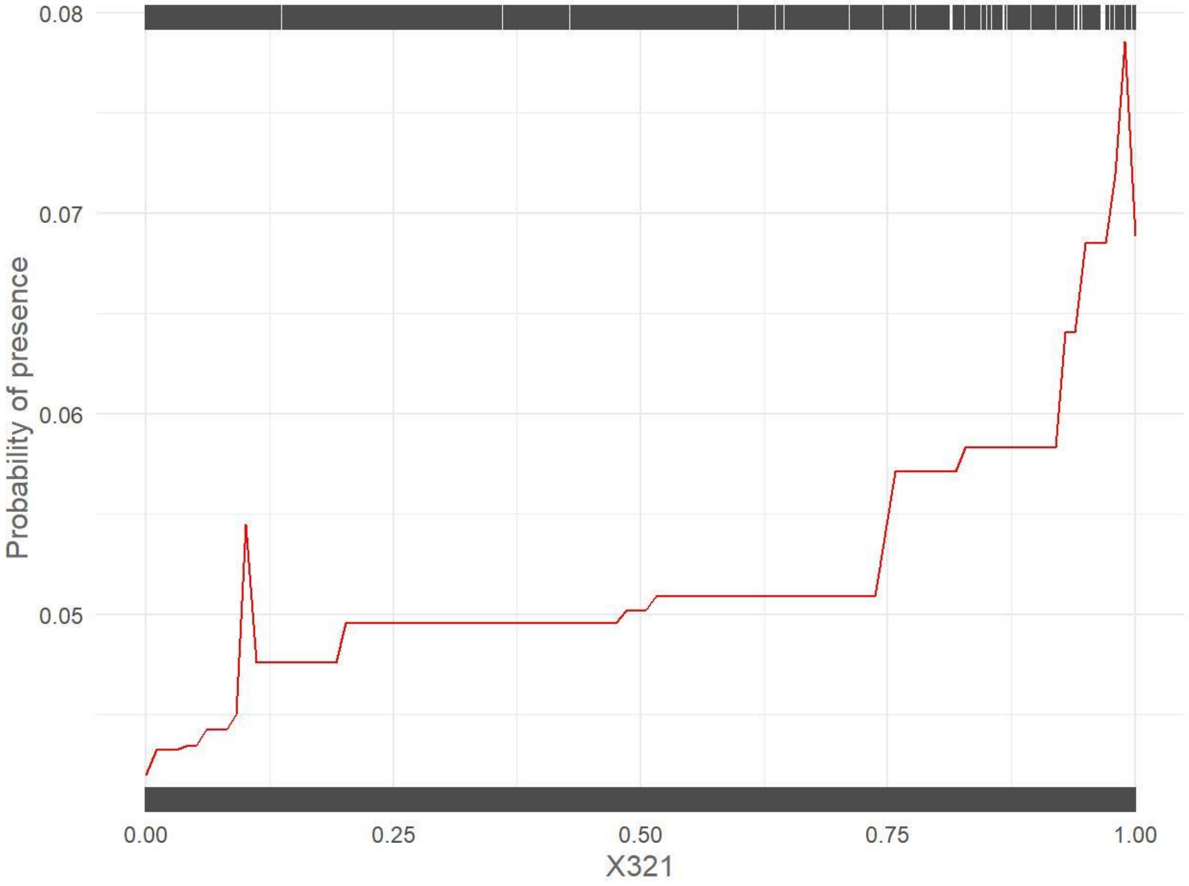
Probability of presence
0.08
0.07
0.06
0.05
0.00
0.25
0.50
0.75
1.00
X321

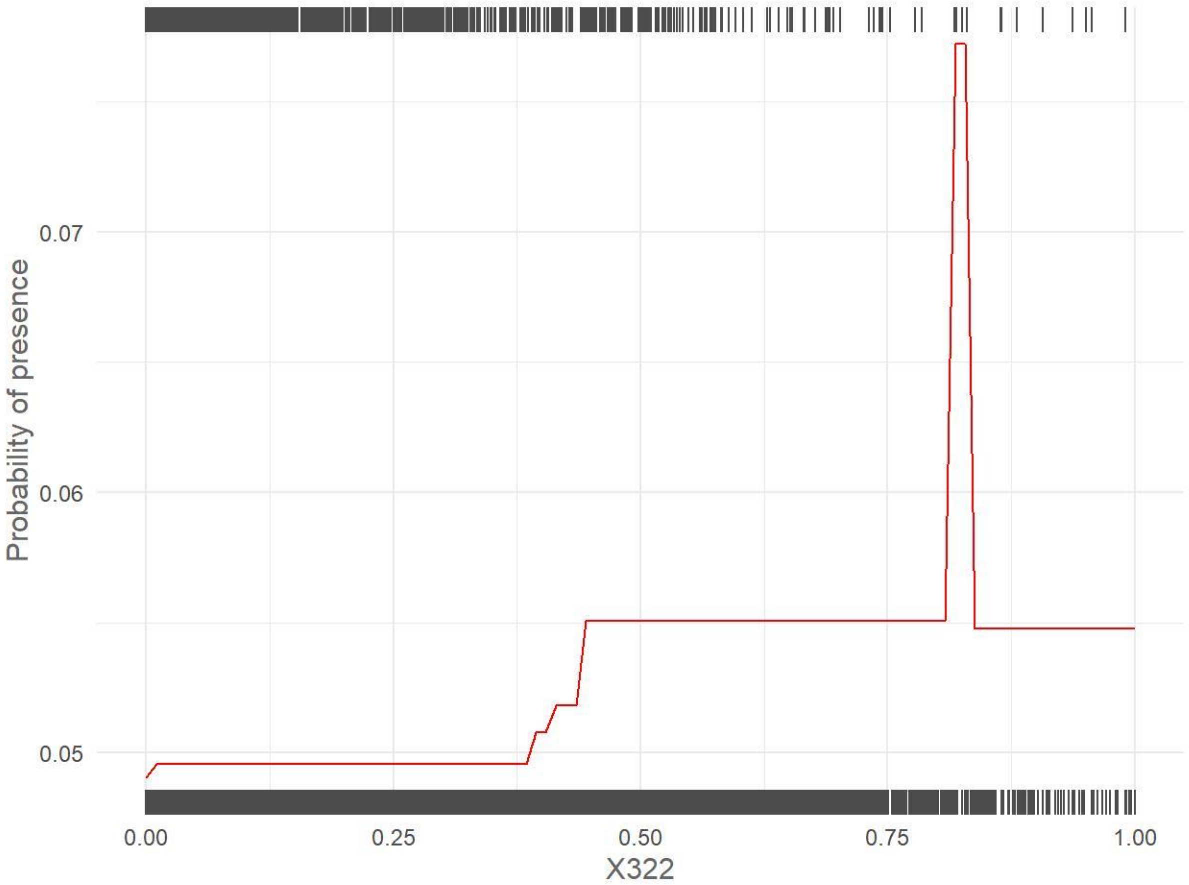
Probability of presence
0.07
0.06
0.05
0.00
0.25
0.50
0.75
1.00
X322

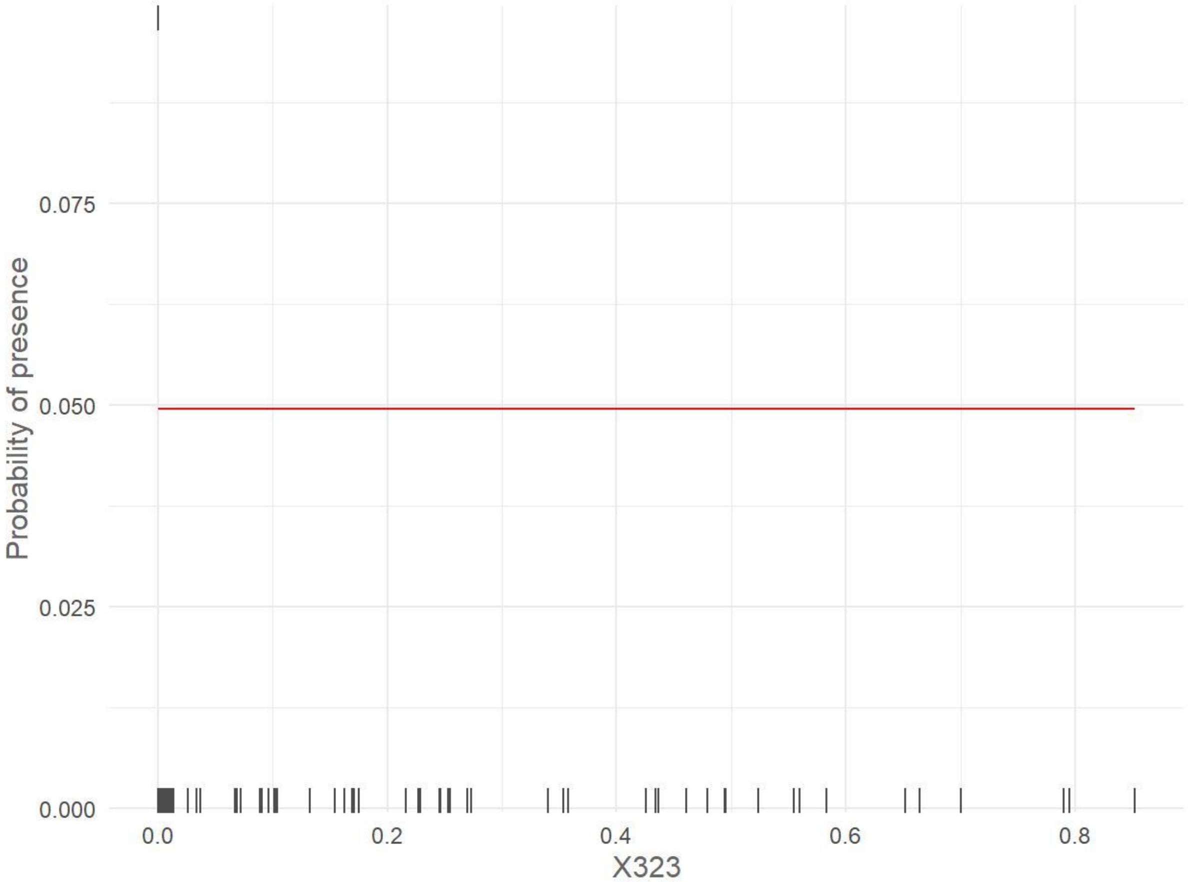
Probability of presence
0.075
0.050
0.025
0.000
0.0
0.2
0.4
0.6
0.8
X323

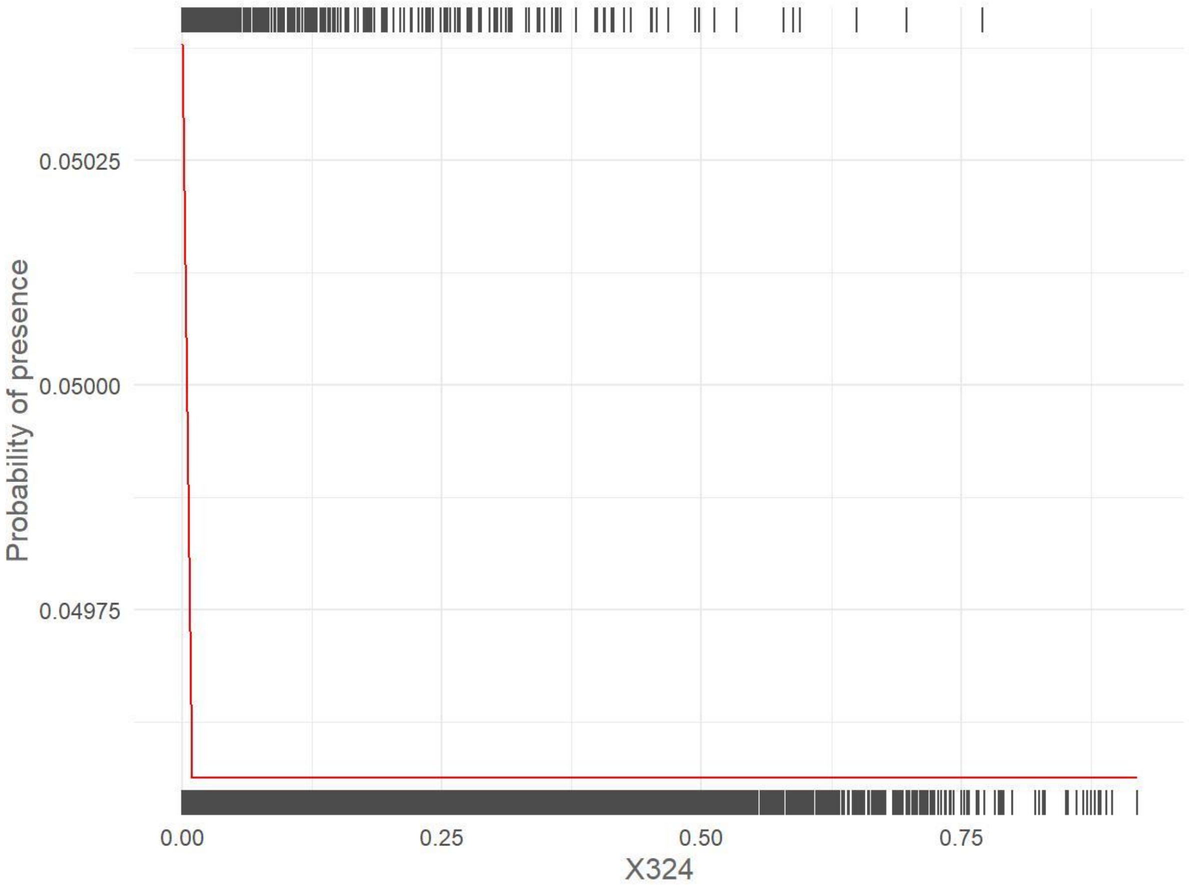
Probability of presence
0.05025
0.05000
0.04975
0.00
0.25
0.50
0.75
X324

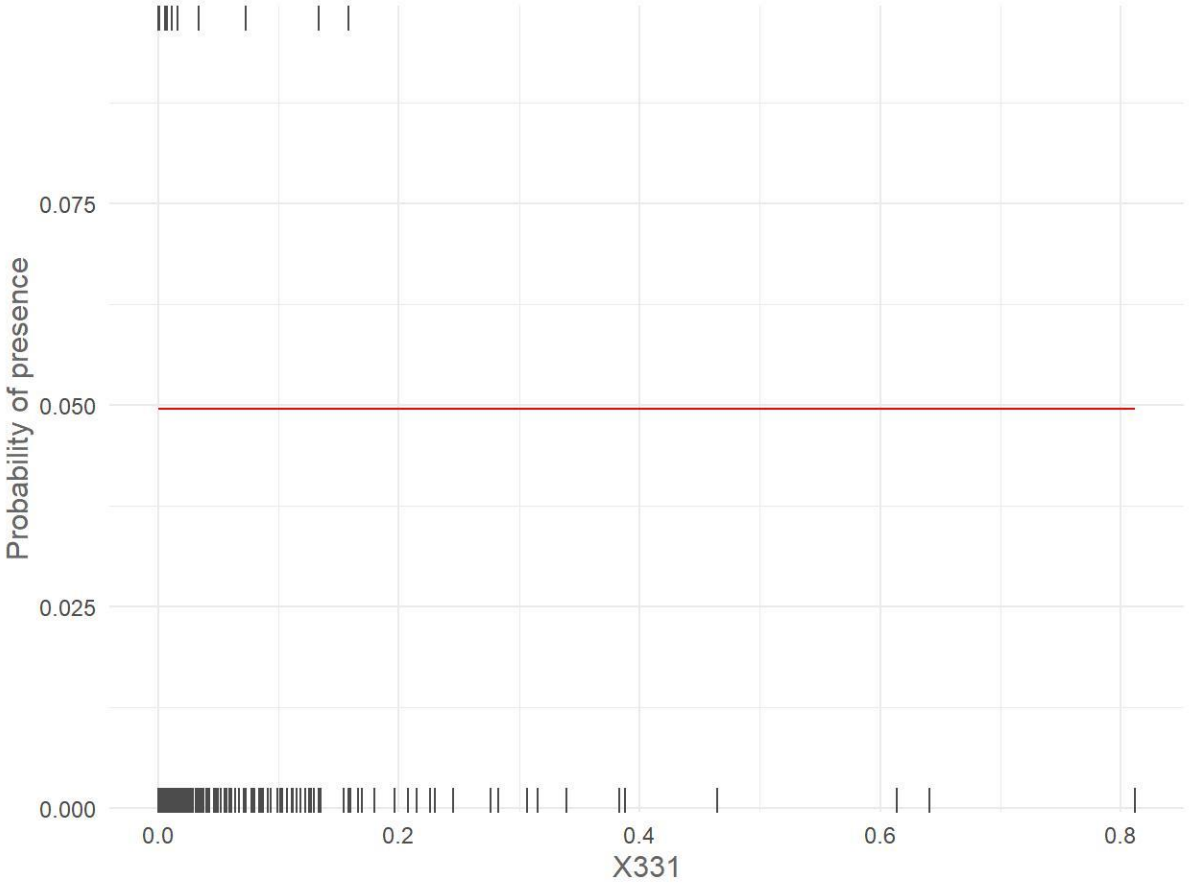
Probability of presence
0.075
0.050
0.025
0.000
0.0
0.2
0.4
0.6
0.8
X331

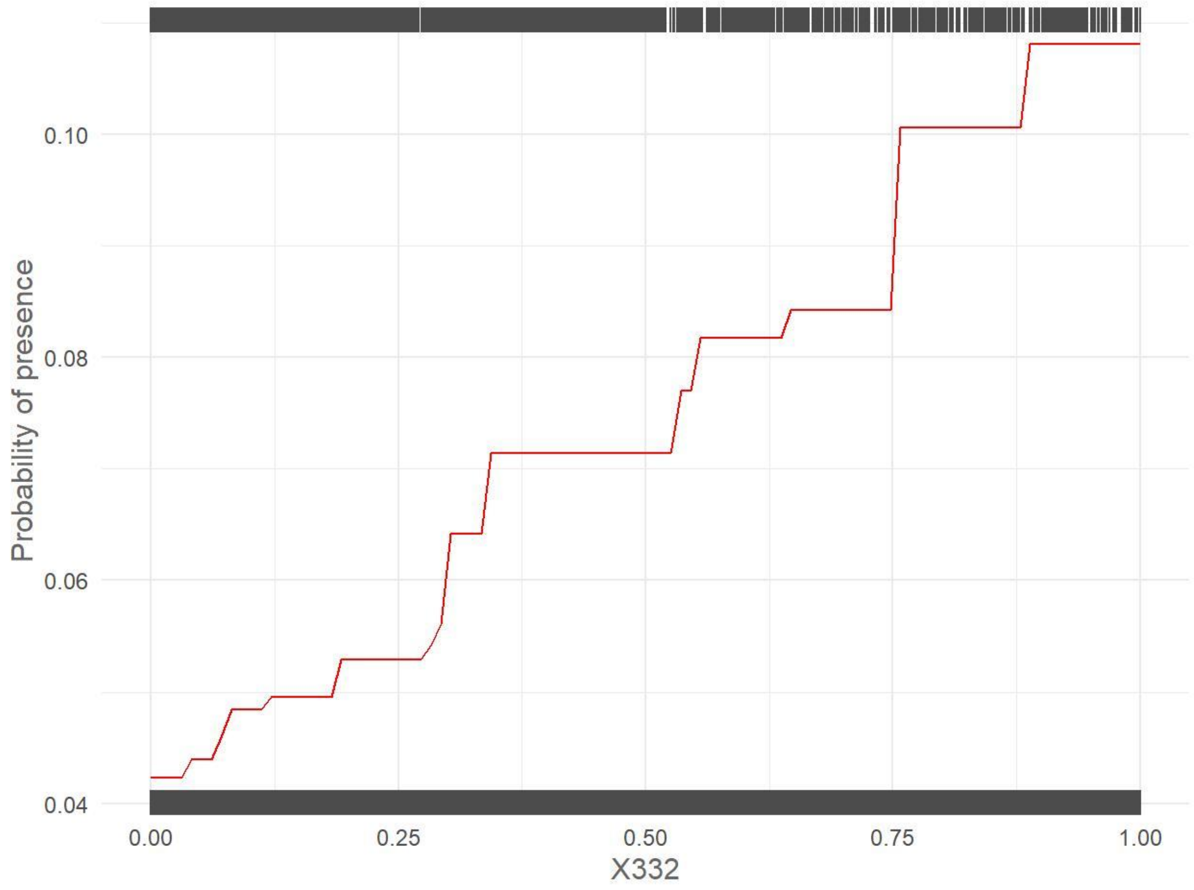
Probability of presence
0.10
0.08
0.06
0.04
0.00
0.25
0.50
0.75
1.00
X332

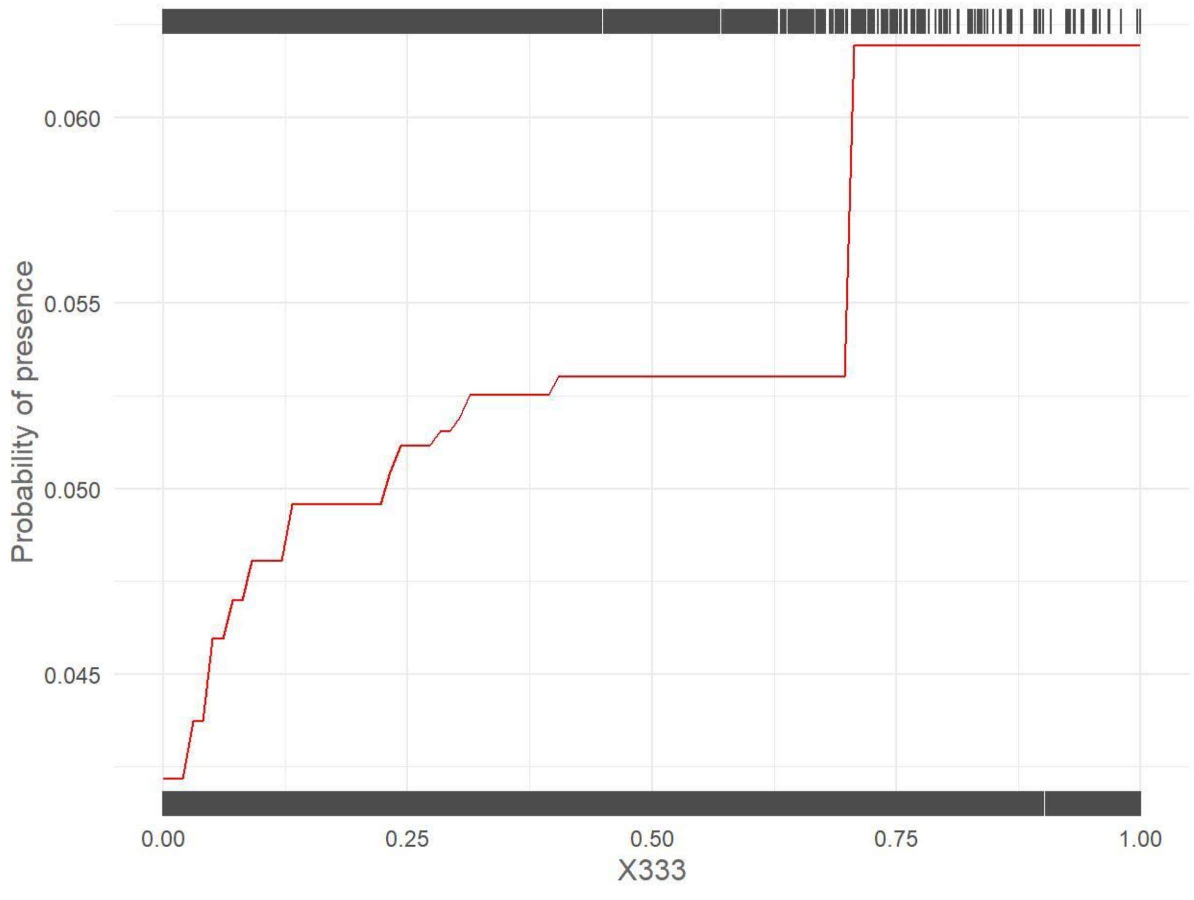
Probability of presence
0.060
0.055
0.050
0.045
0.00
0.25
0.50
0.75
1.00
X333

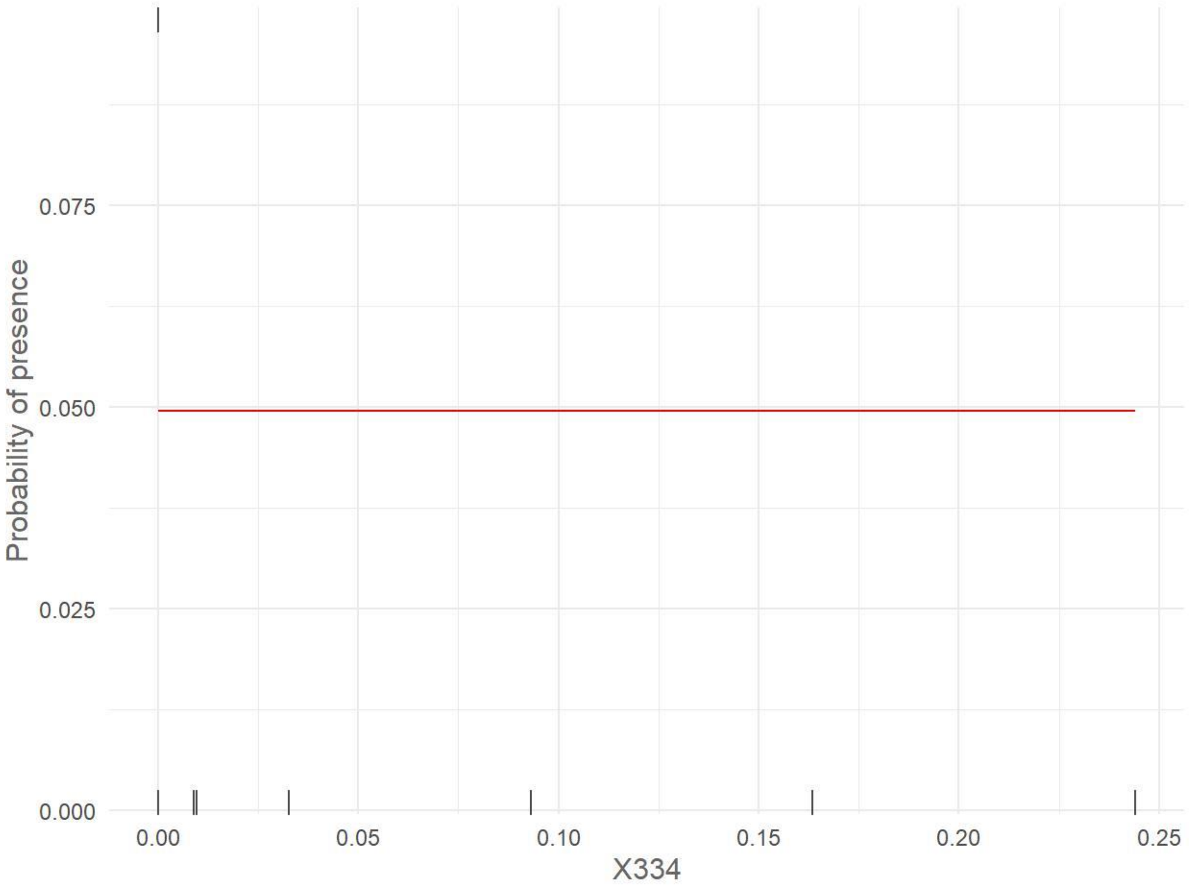

Probability of presence
0.000
0.025
0.050
0.075
0.00
0.05
0.10
0.15
0.20
0.25
X334

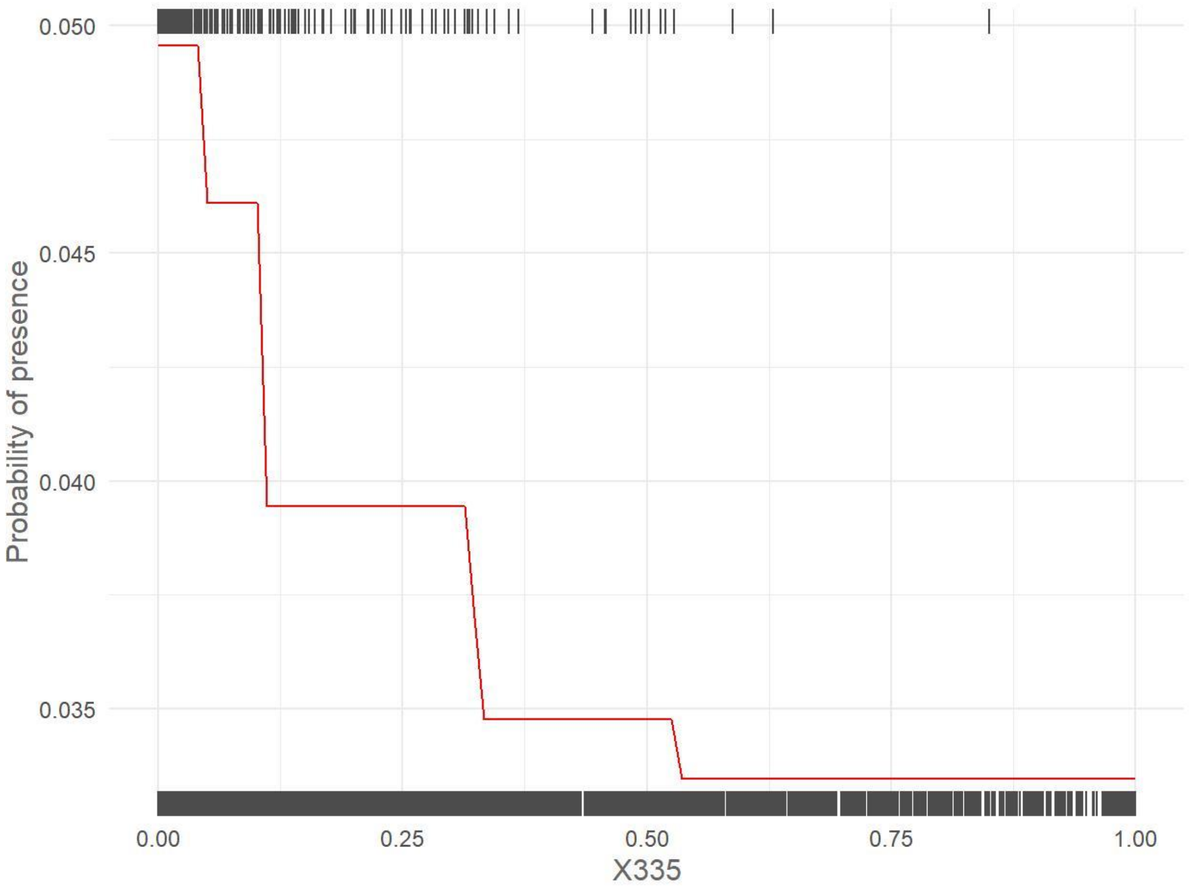

Probability of presence
0.035
0.040
0.045
0.050
0.00
0.25
0.50
0.75
1.00
X335

Figure S10. Species-habitat relationships according to the Boosted Regression Trees model for water pipit.

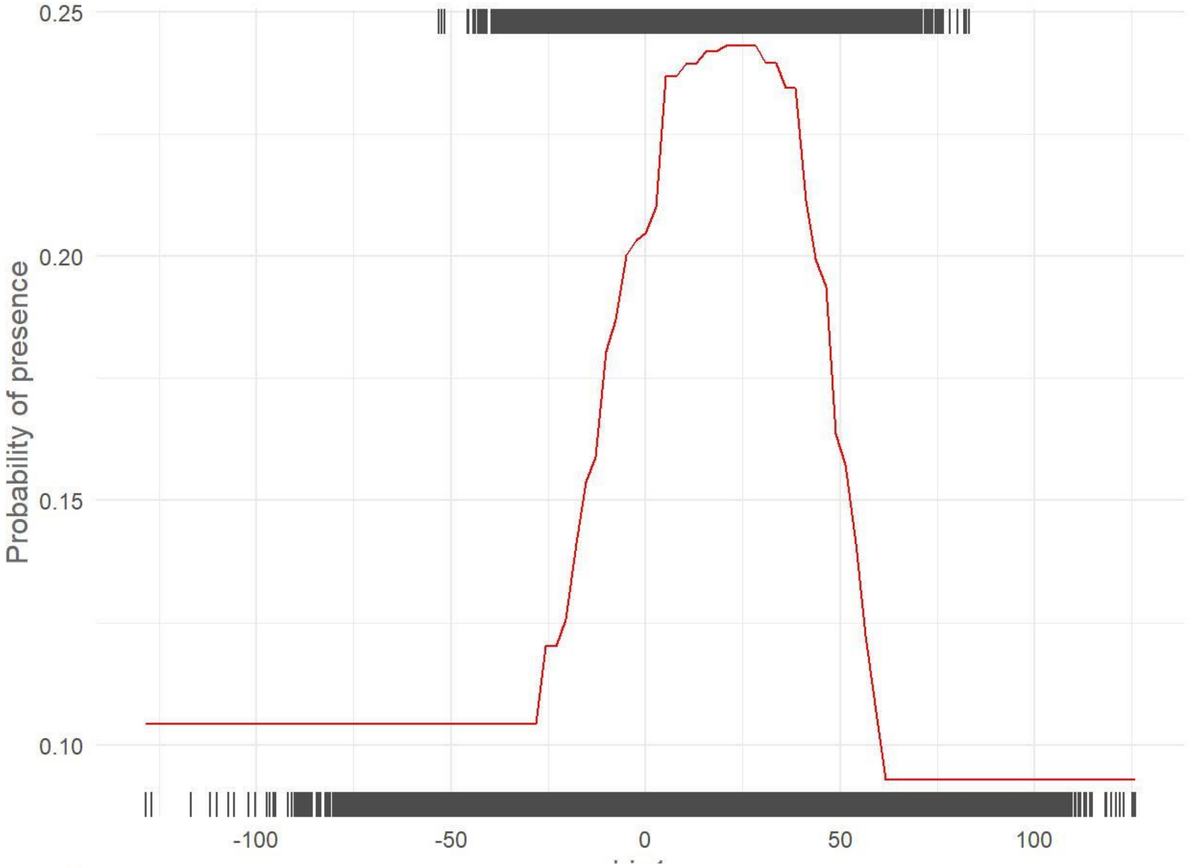
Probability of presence
0.25
0.20
0.15
0.10
-100
-50
0
50
100

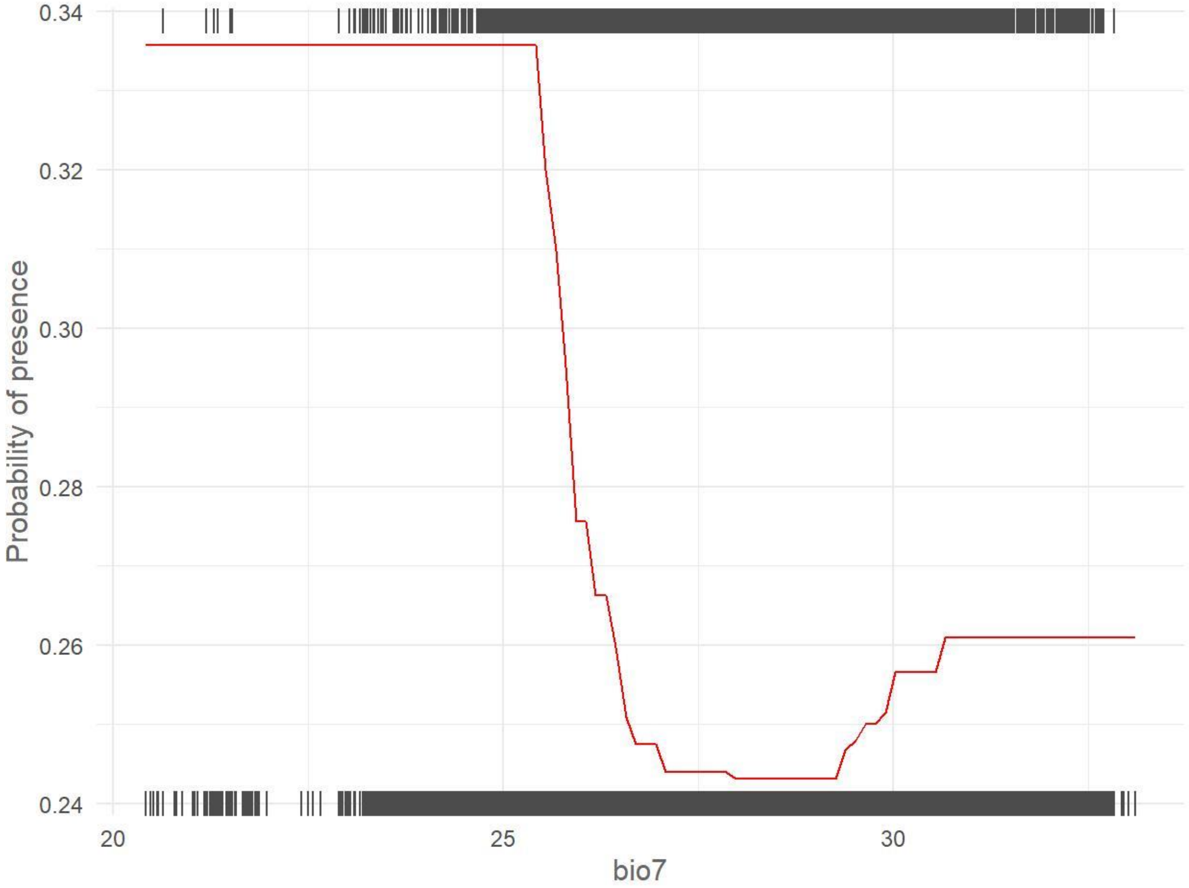
Probability of presence
0.34
0.32
0.30
0.28
0.26
0.24
20
25
30
bio7

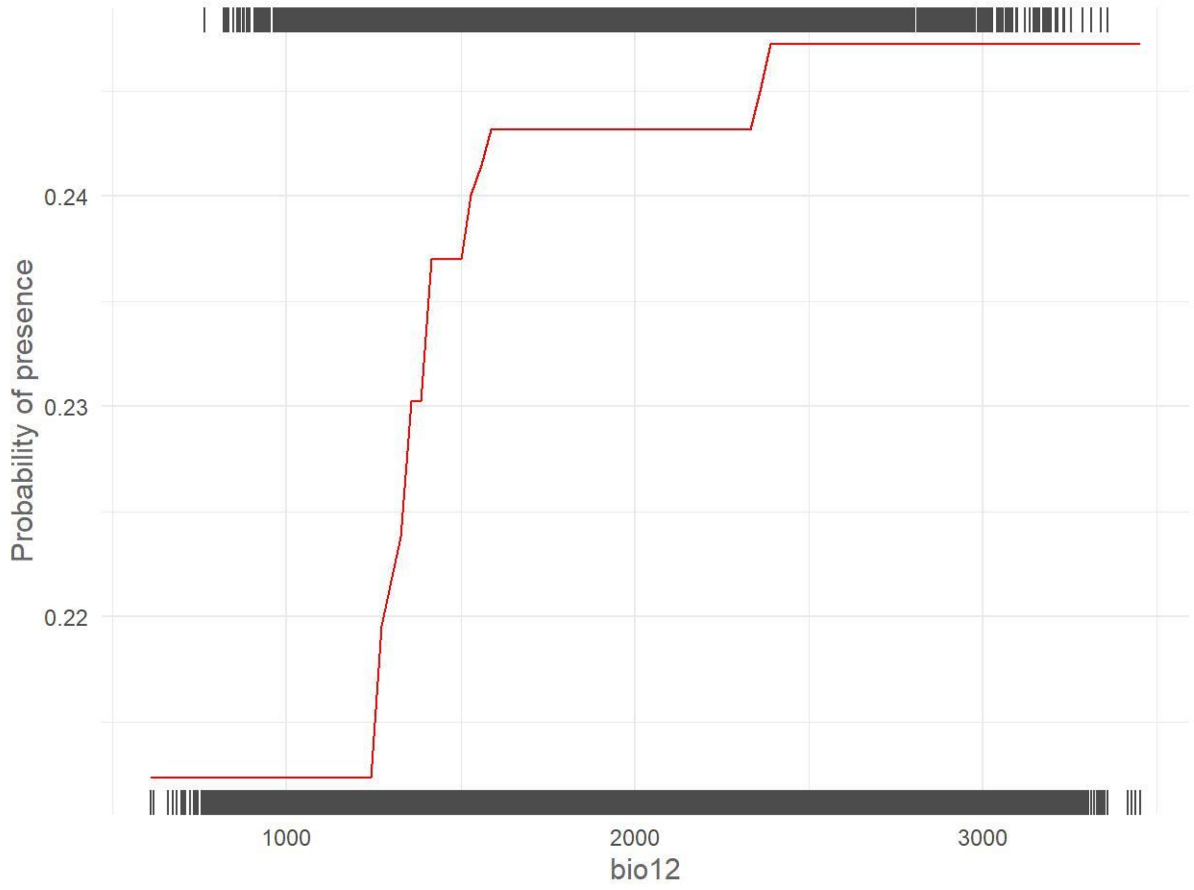
Probability of presence
0.24
0.23
0.22
1000
2000
3000
bio12

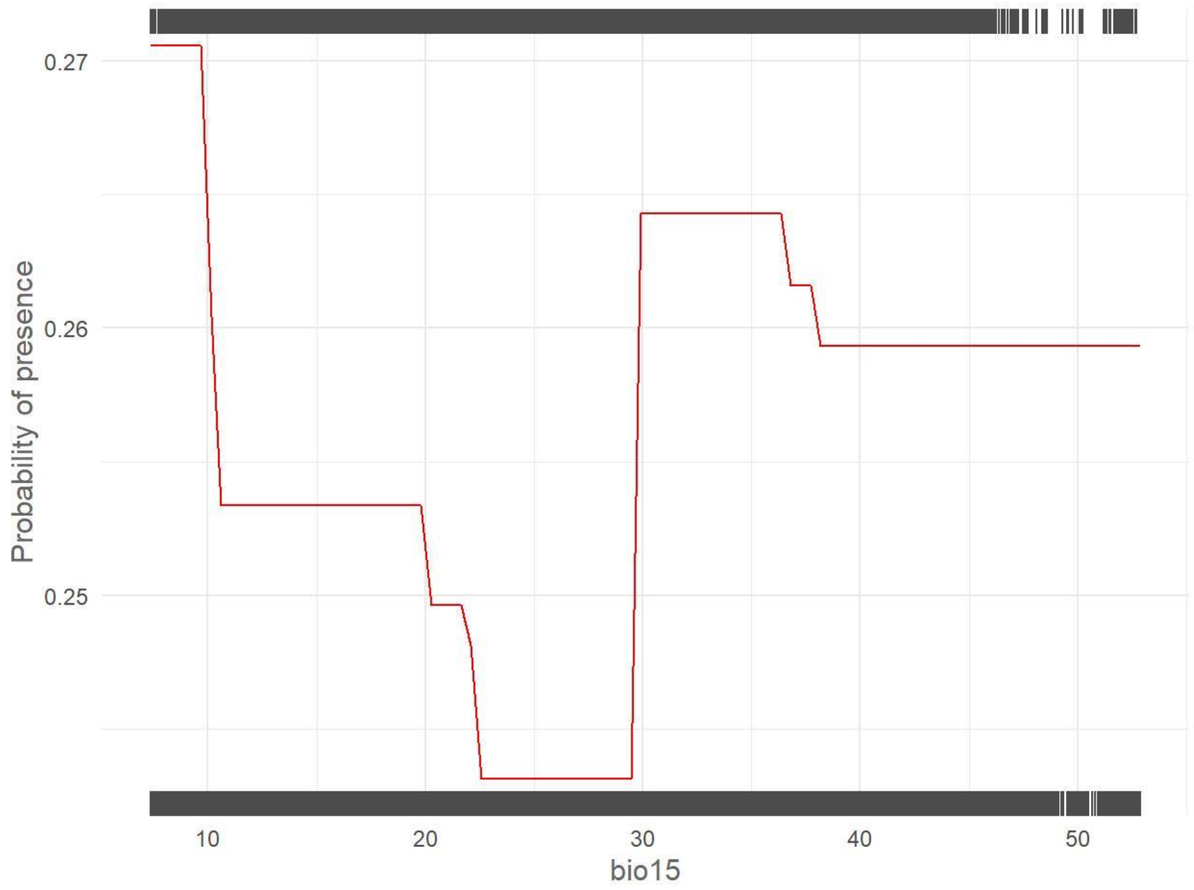
Probability of presence
0.27
0.26
0.25
10
20
30
40
50
bio15

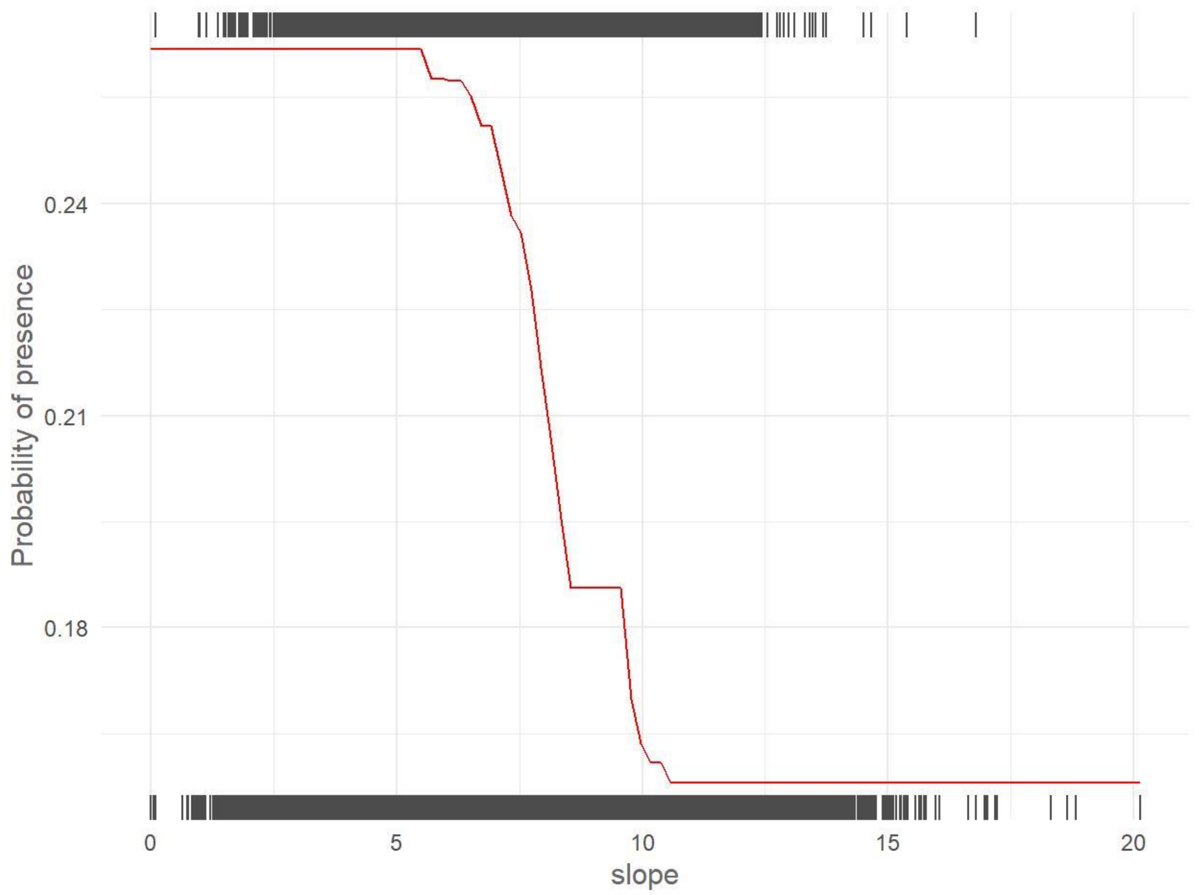

Probability of presence
0.24
0.21
0.18
0
5
10
15
20
slope

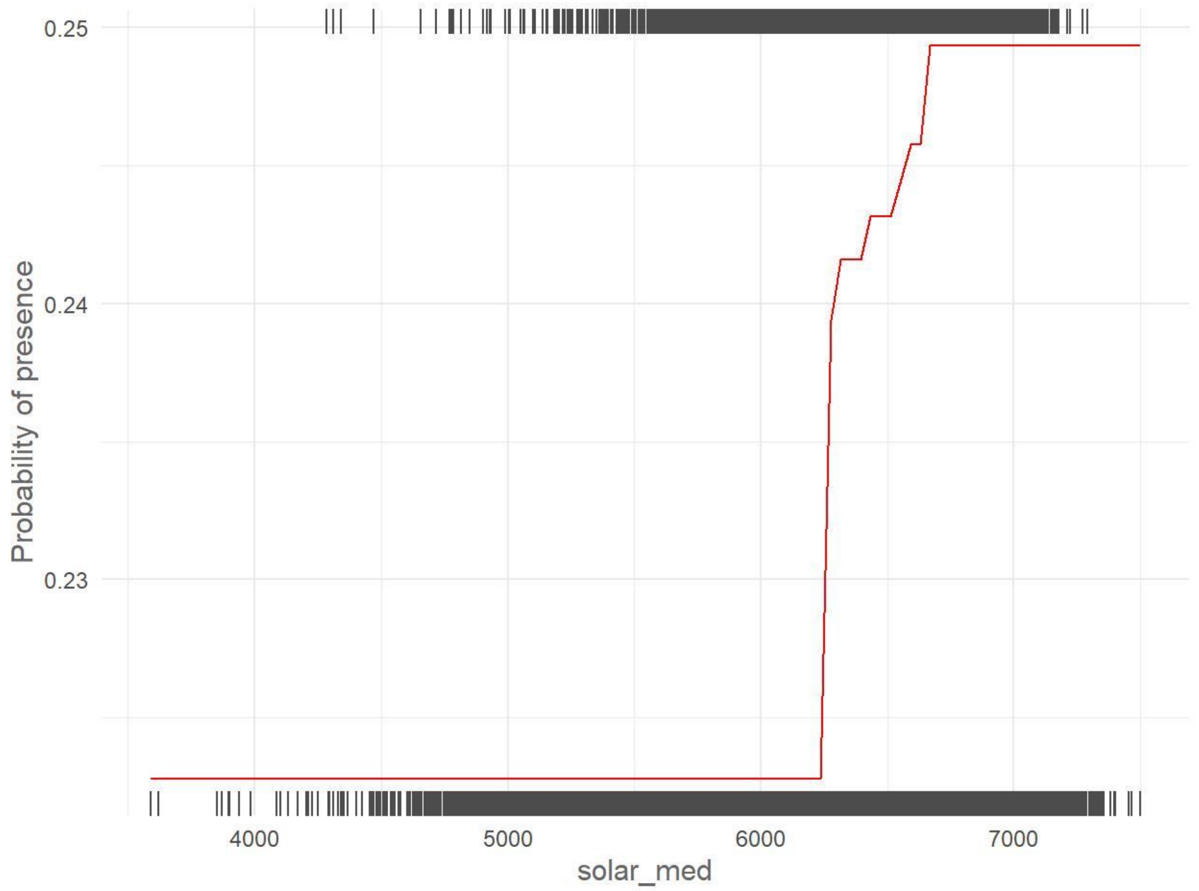

Probability of presence
0.25
0.24
0.23
4000
5000
6000
7000
solar_med

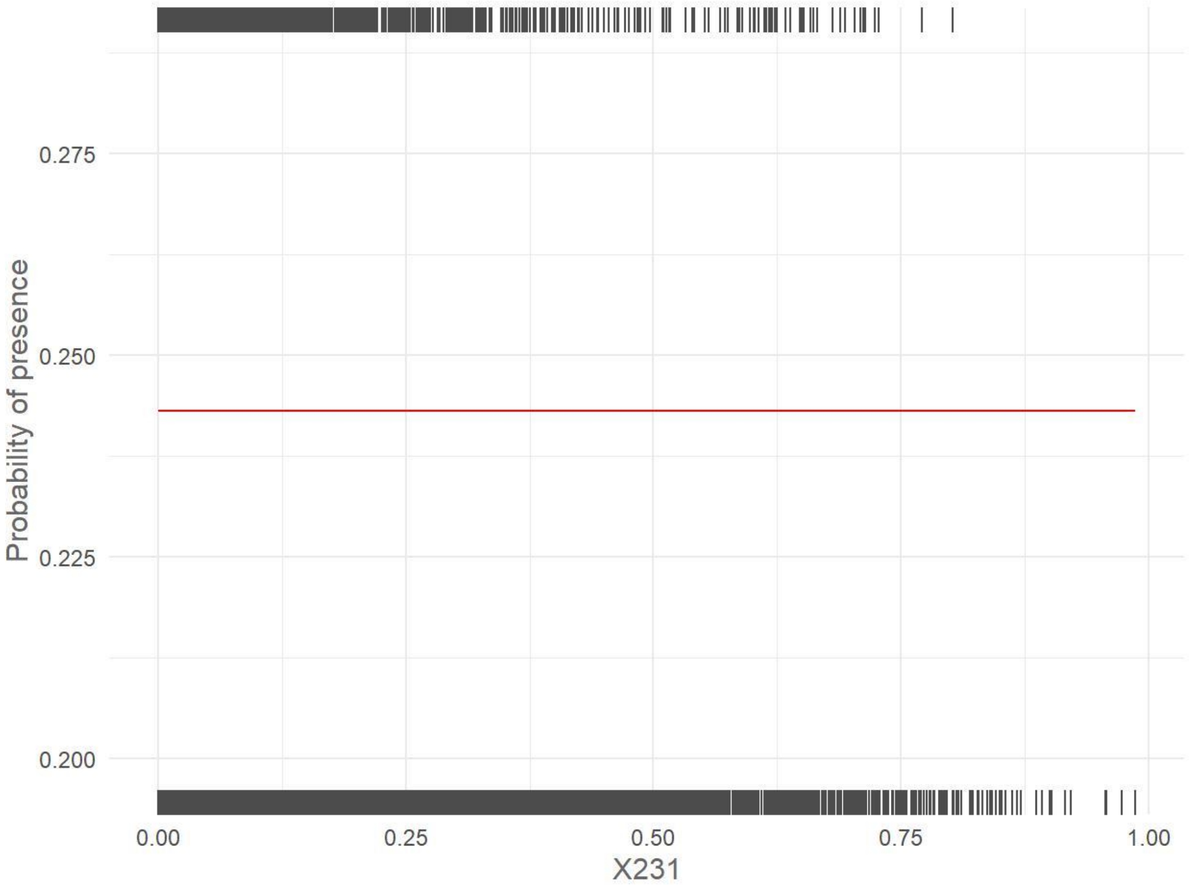
0.275
0.250
0.225
0.200
Probability of presence
0.00
0.25
0.50
0.75
1.00
X231

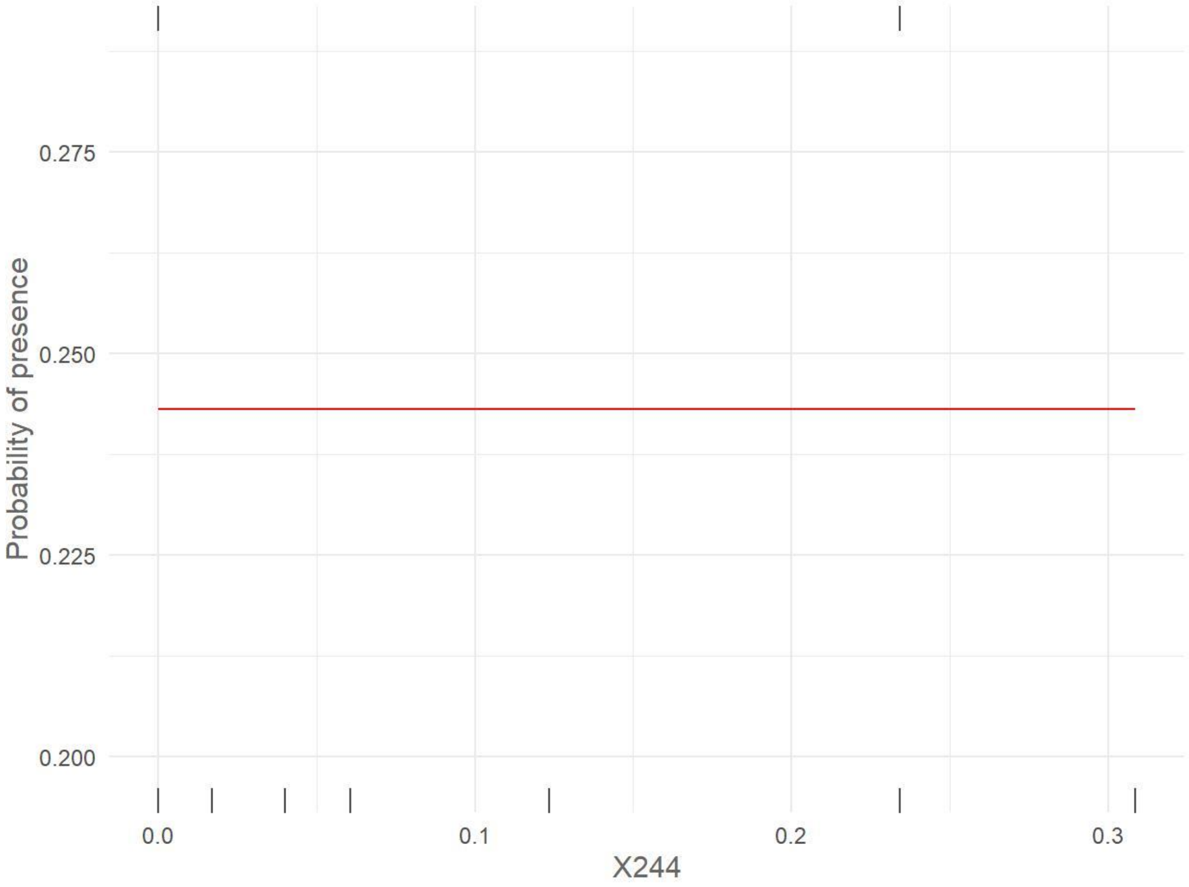
0.275
0.250
0.225
0.200
Probability of presence
0.0
0.1
0.2
0.3
X244

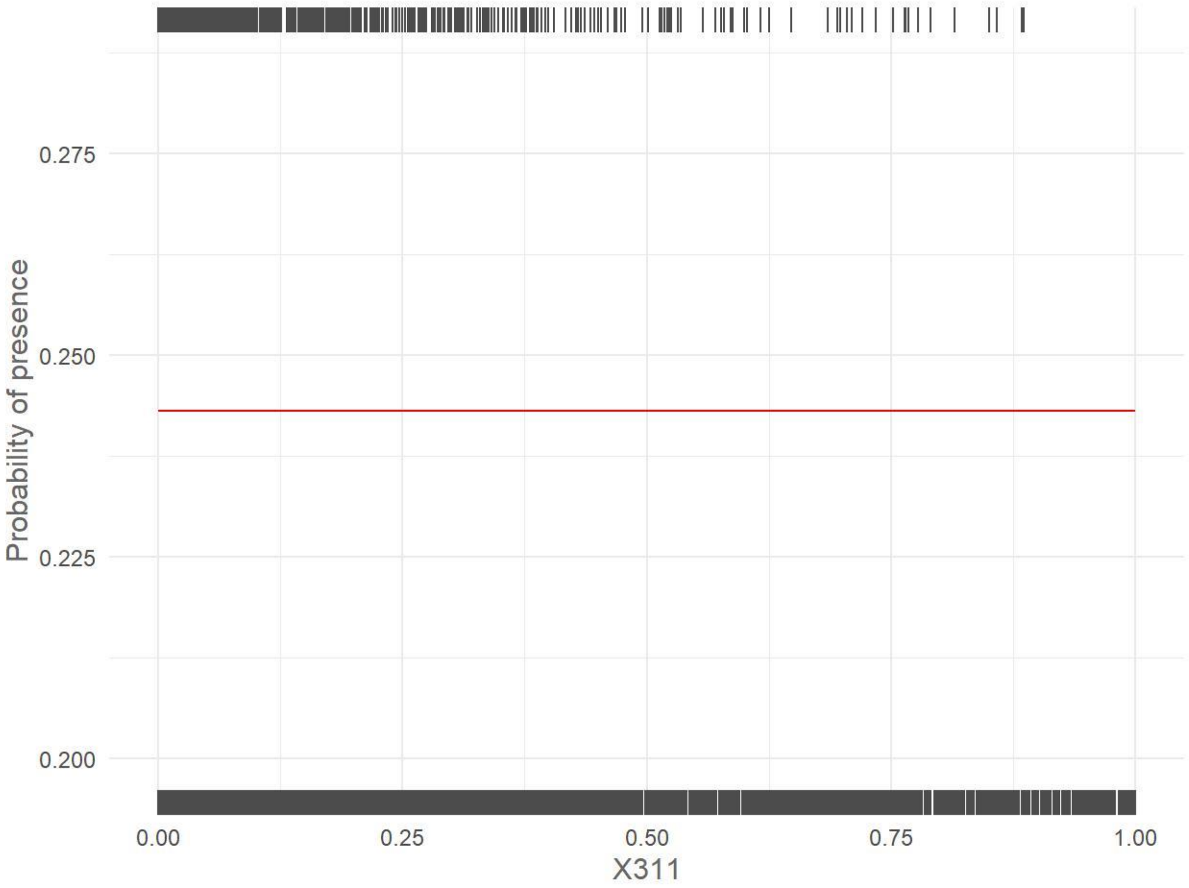

Probability of presence
0.275
0.250
0.225
0.200
0.00
0.25
0.50
0.75
1.00
X311

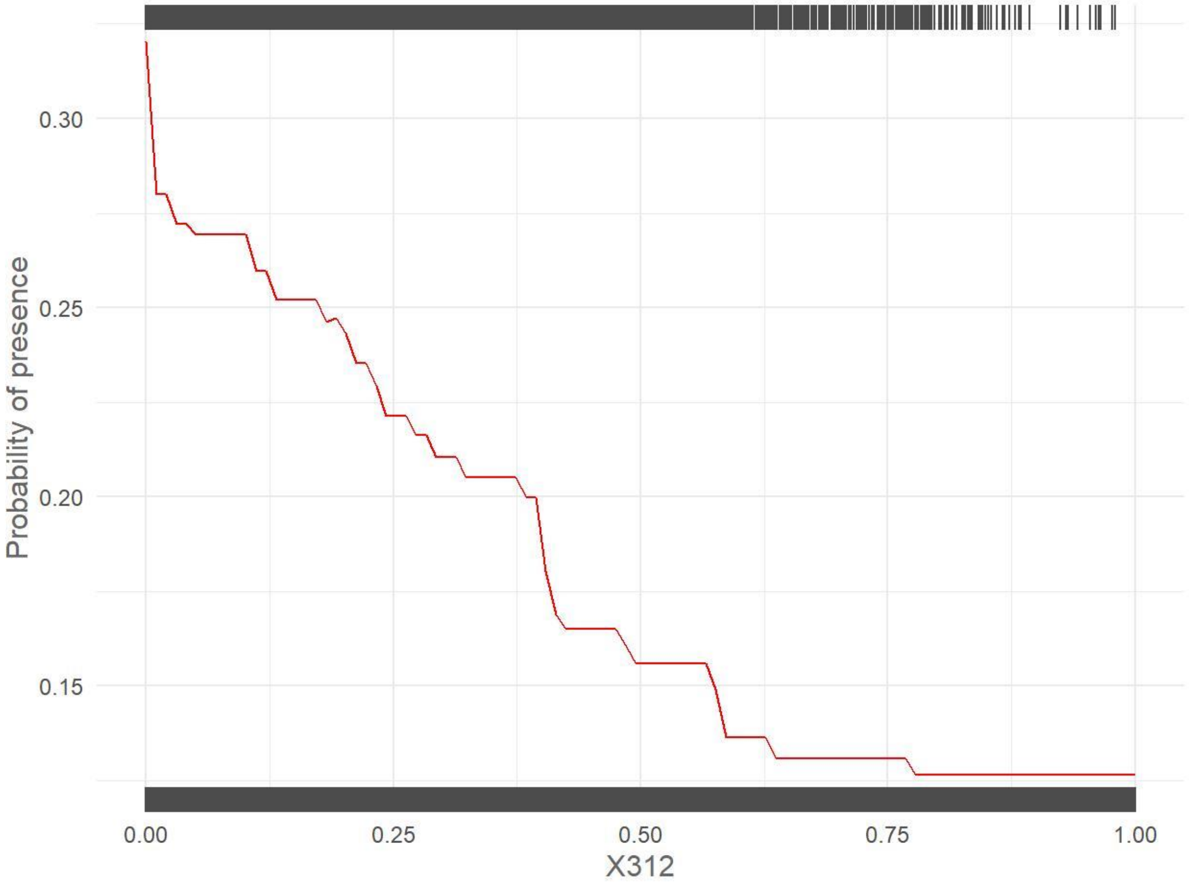

Probability of presence
0.30
0.25
0.20
0.15
0.00
0.25
0.50
0.75
1.00
X312

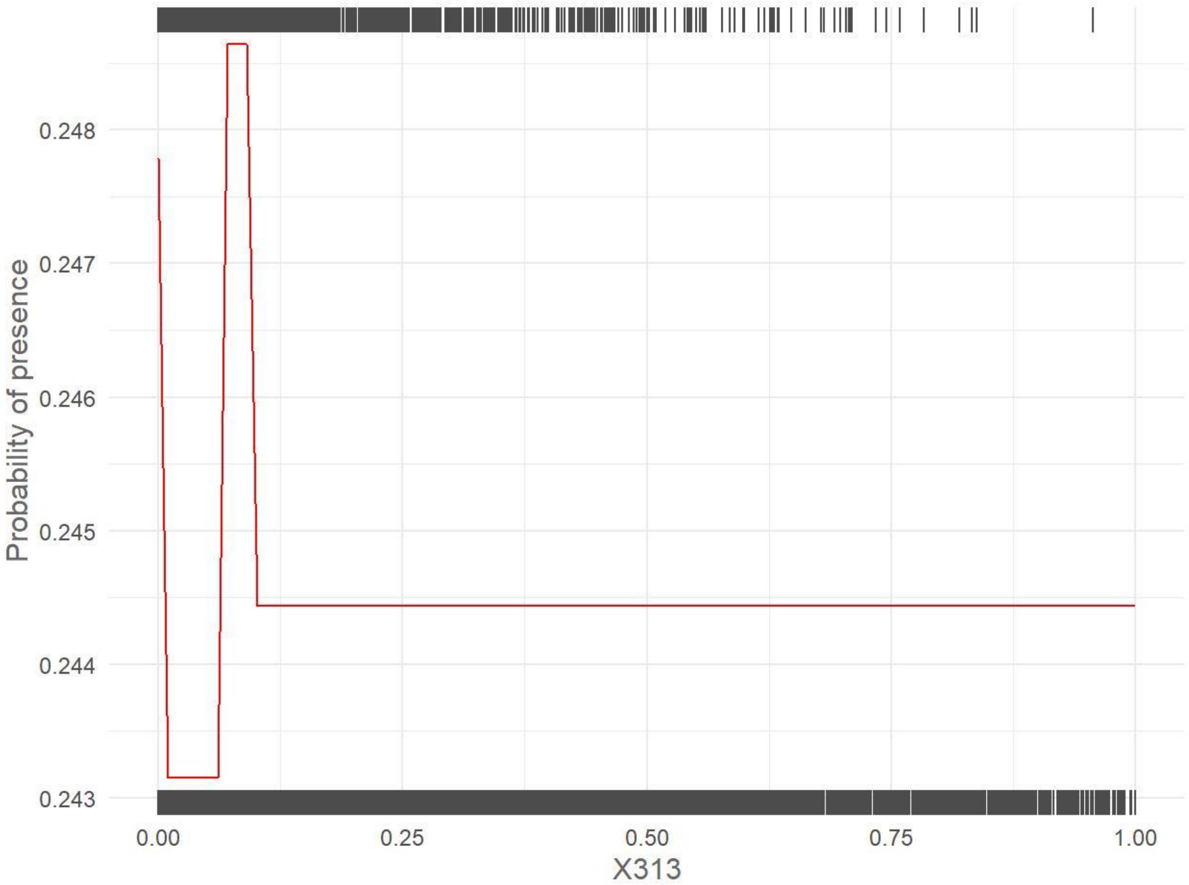
Probability of presence
0.248
0.247
0.246
0.245
0.244
0.243
0.00
0.25
0.50
0.75
1.00
X313

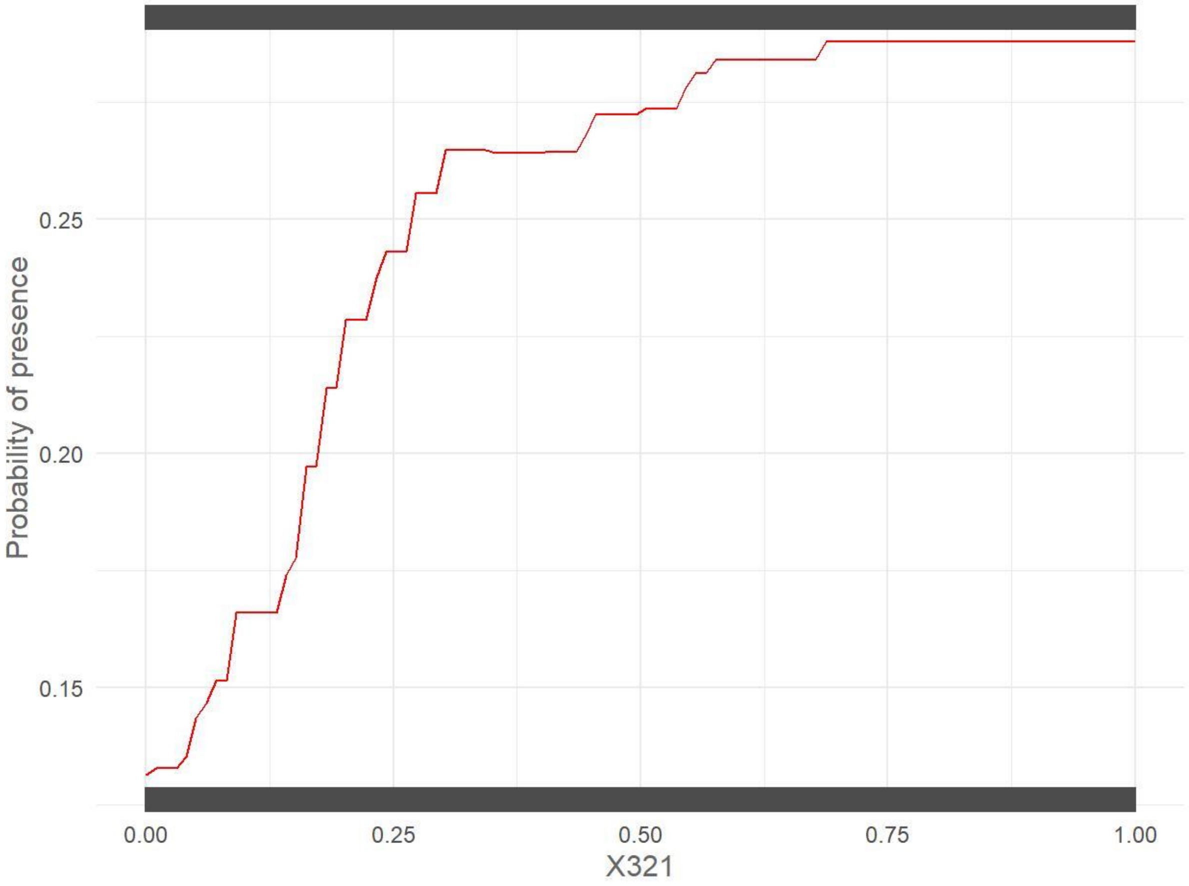
Probability of presence
0.25
0.20
0.15
0.00
0.25
0.50
0.75
1.00
X321

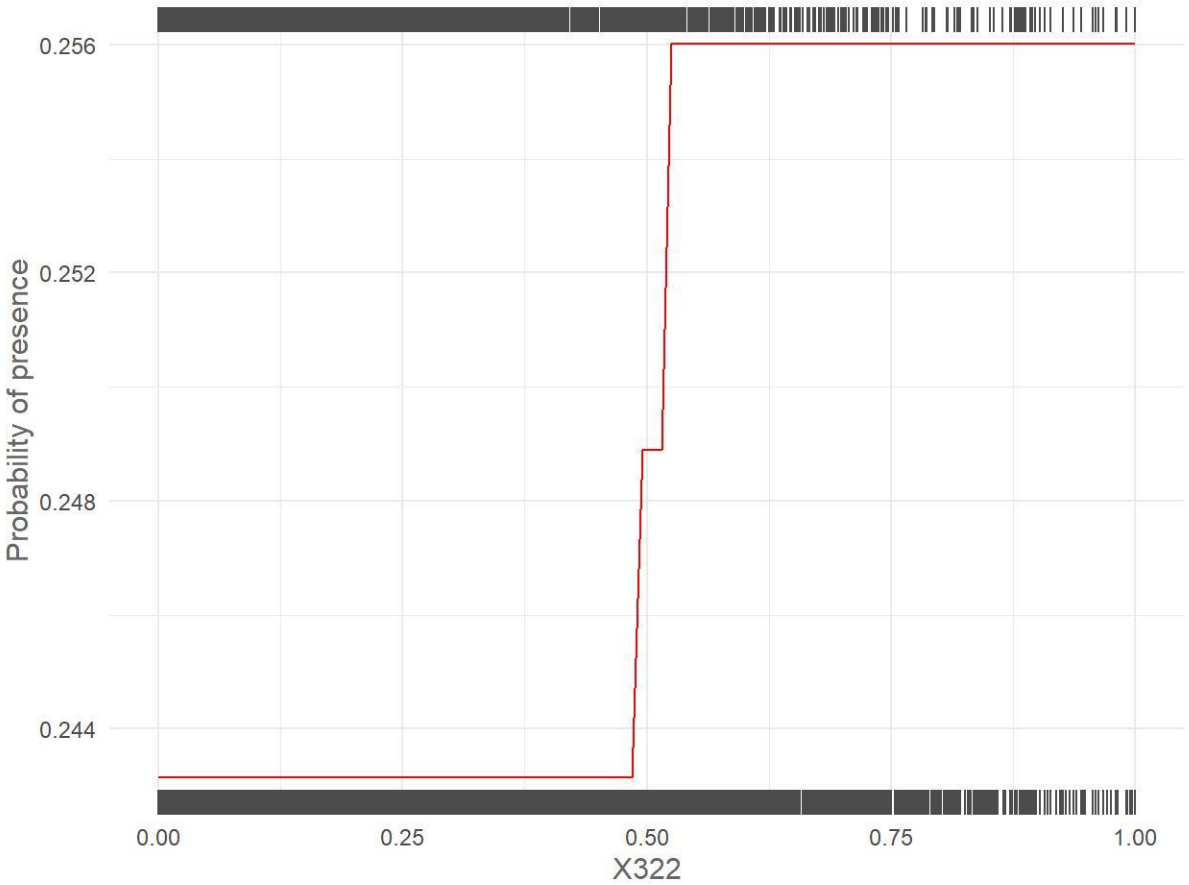

0.256
0.252
0.248
0.244
Probability of presence
0.00
0.25
0.50
0.75
1.00
X322

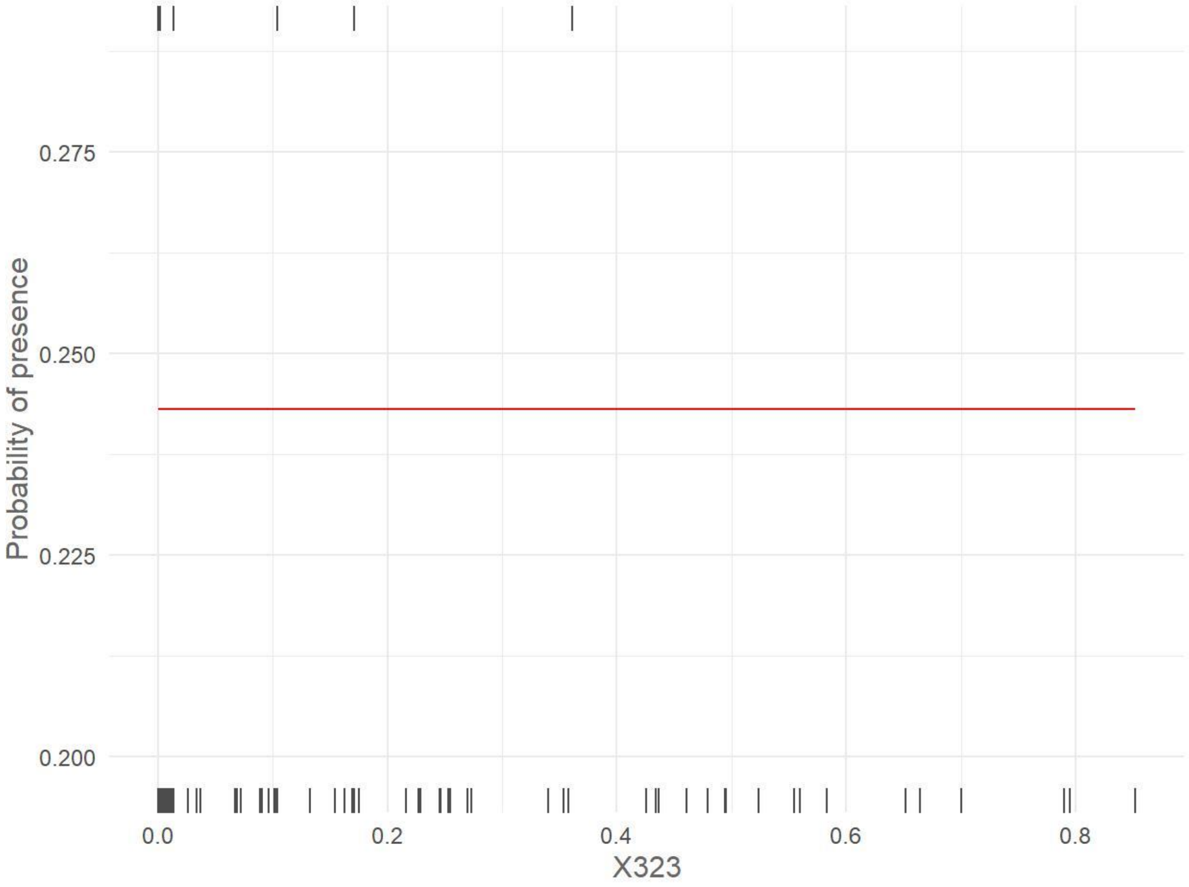

0.275
0.250
0.225
0.200
Probability of presence
0.0
0.2
0.4
0.6
0.8
X323

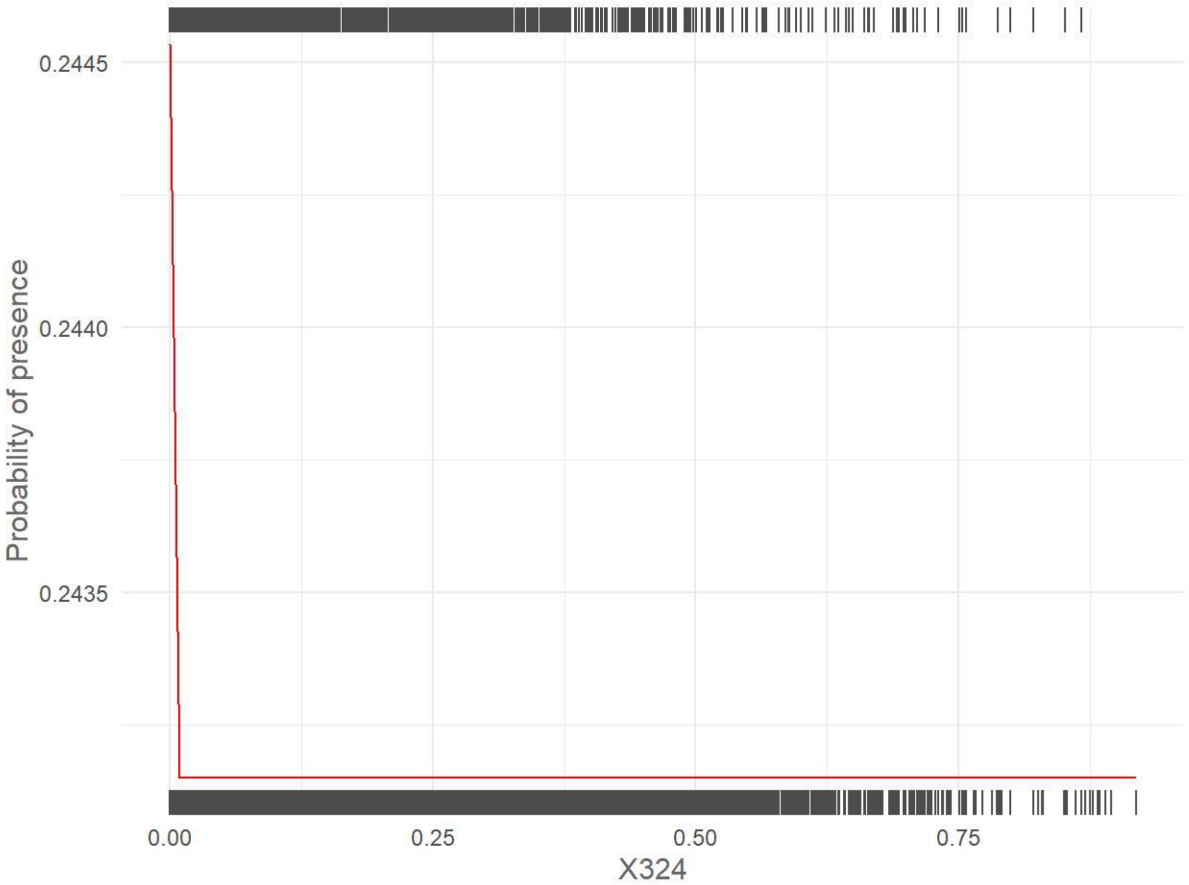

0.2445
0.2440
0.2435
Probability of presence
0.00
0.25
0.50
0.75
X324

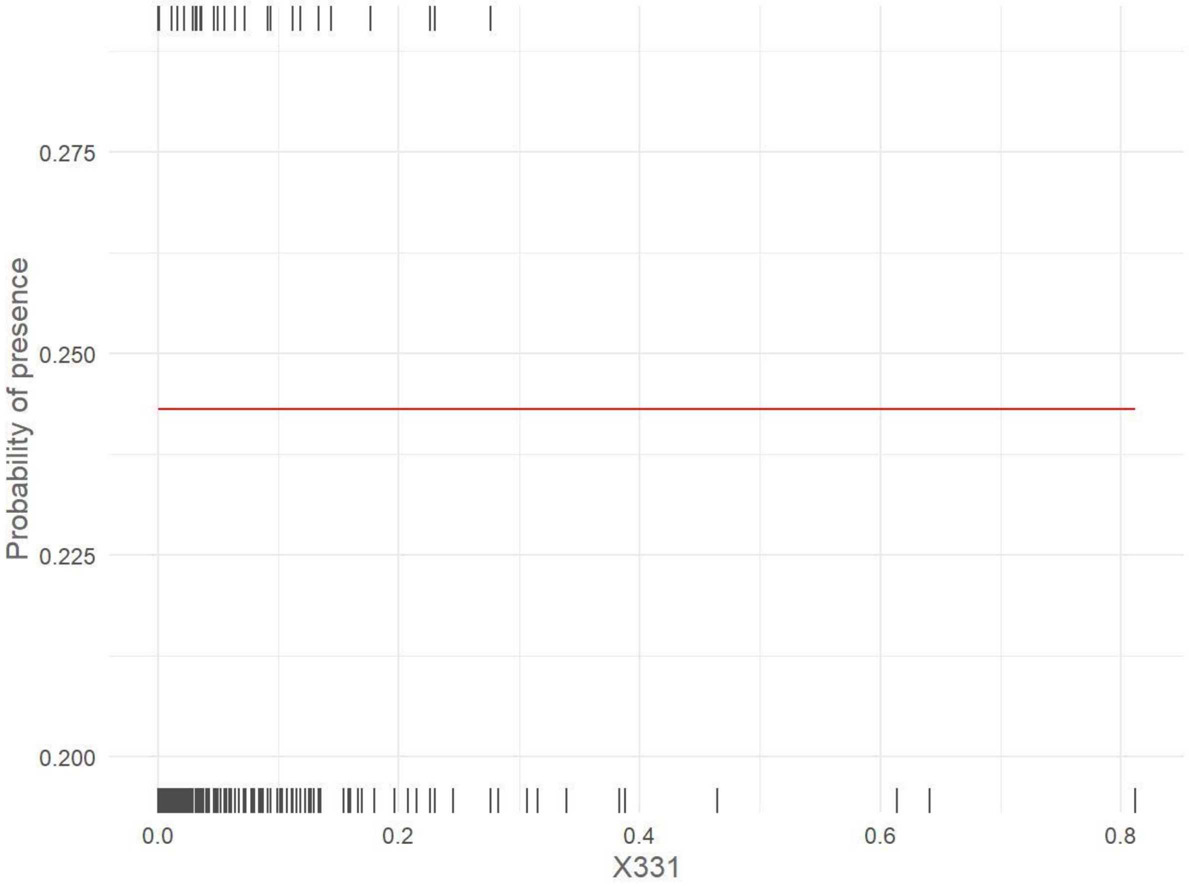

0.275
0.250
0.225
0.200
Probability of presence
0.0
0.2
0.4
0.6
0.8
X331

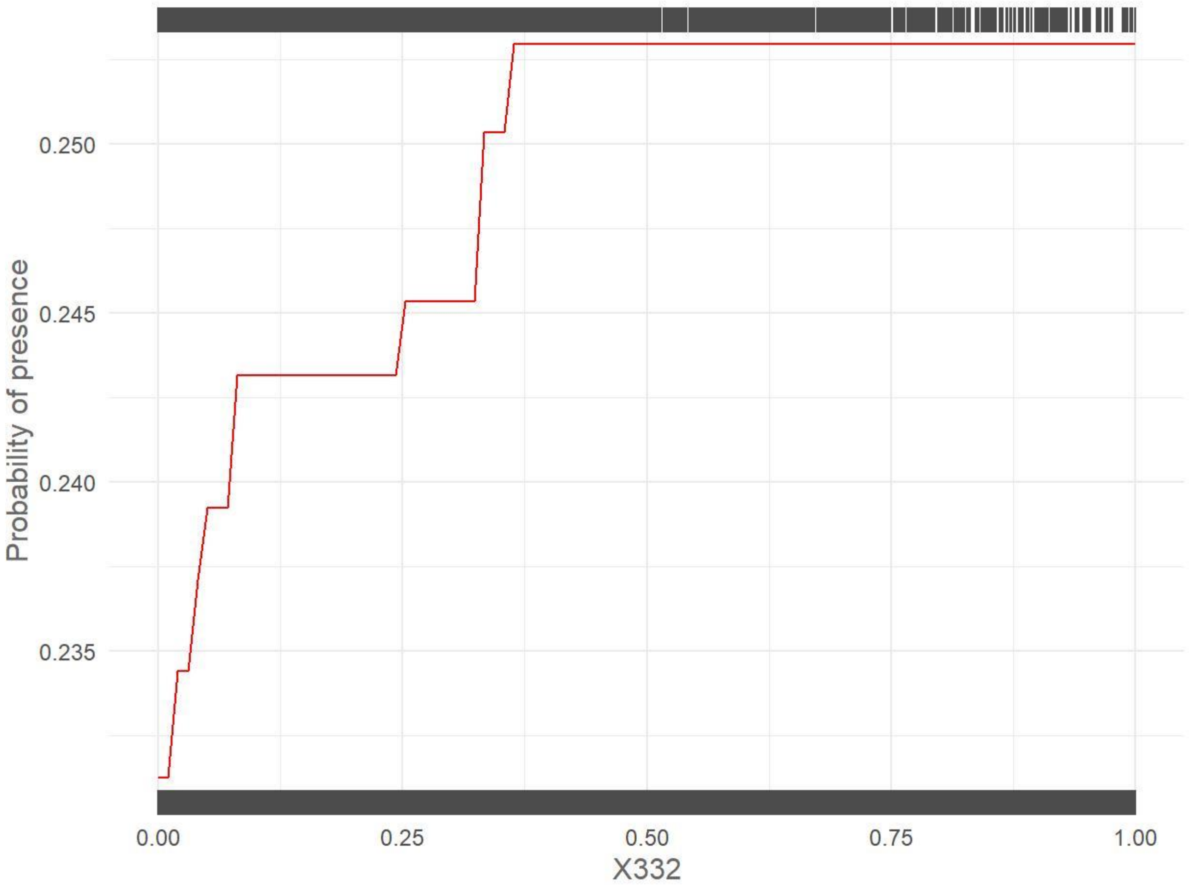

0.250
0.245
0.240
0.235
Probability of presence
0.00
0.25
0.50
0.75
1.00
X332

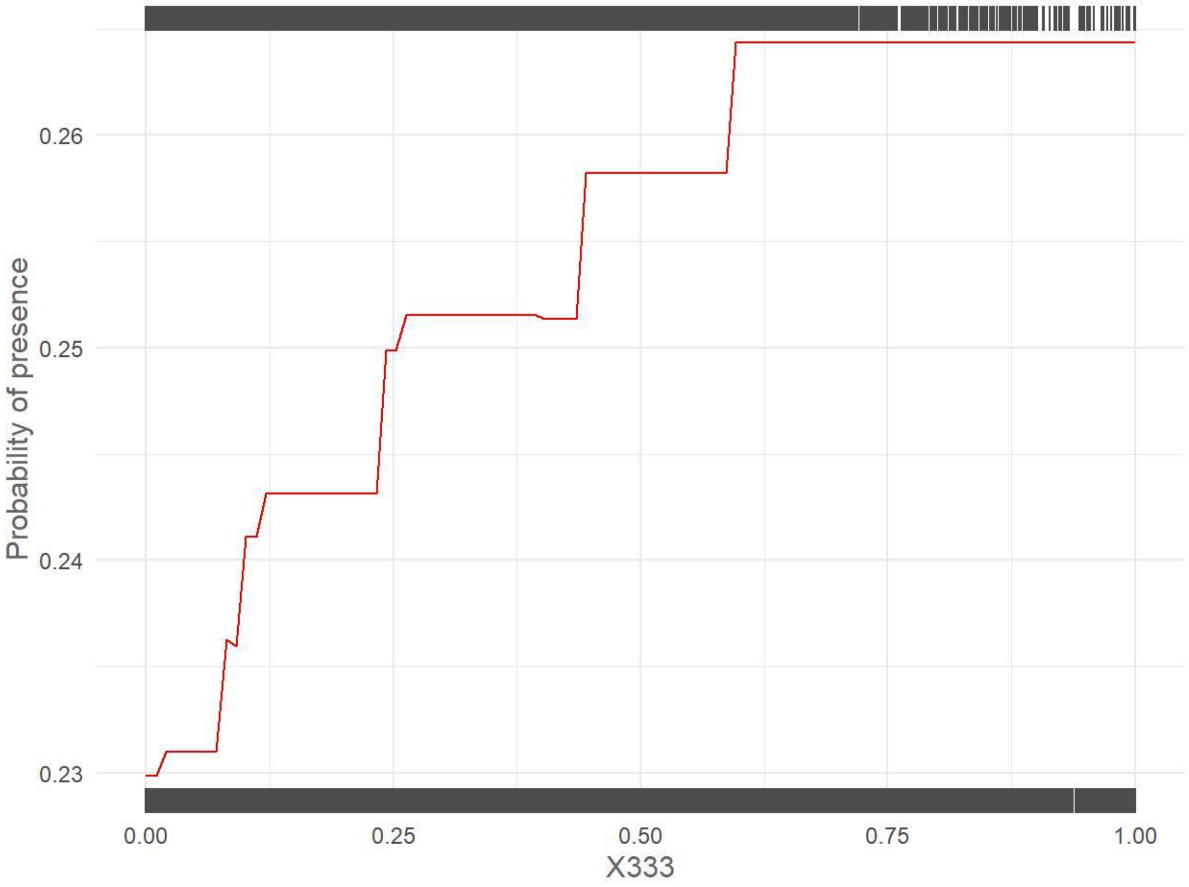

0.26
0.25
0.24
0.23
Probability of presence
0.00
0.25
0.50
0.75
1.00
X333

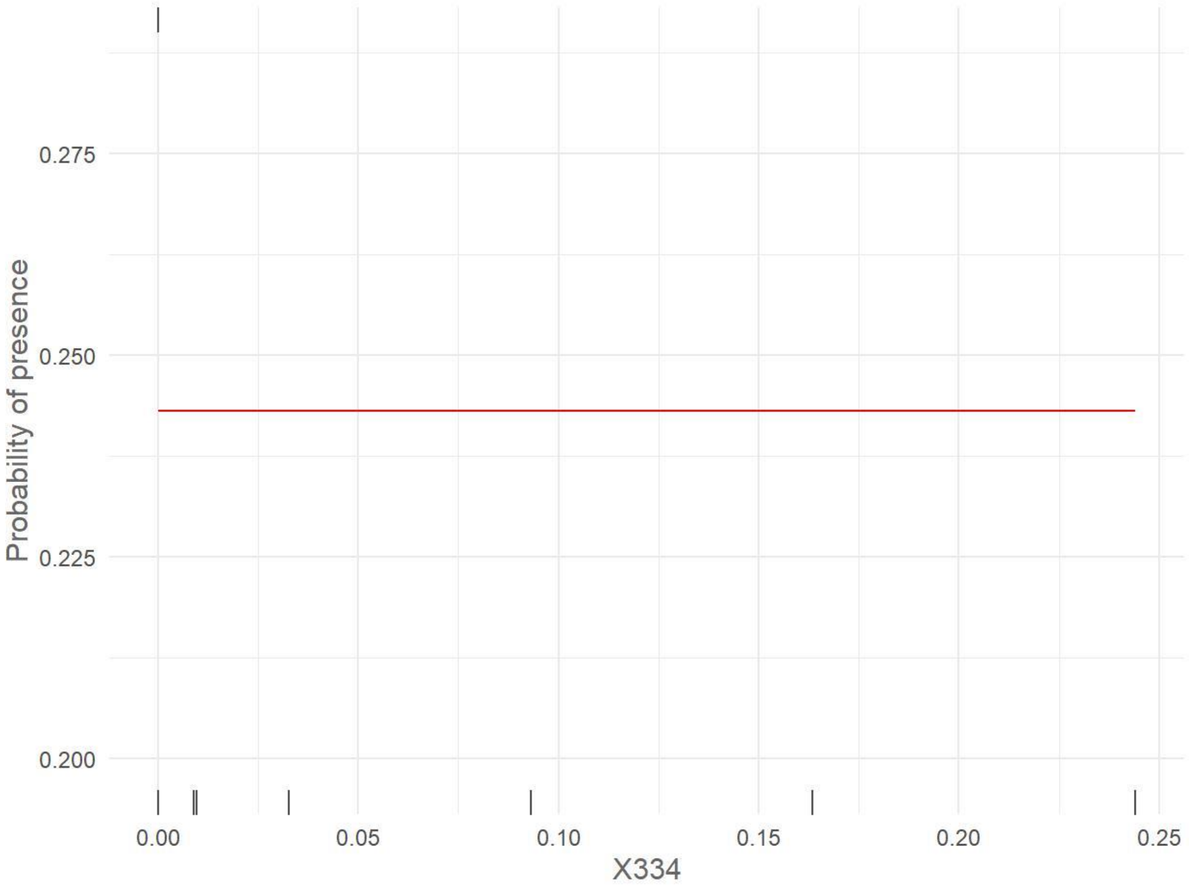

Probability of presence
0.275
0.250
0.225
0.200
0.00
0.05
0.10
0.15
0.20
0.25
X334

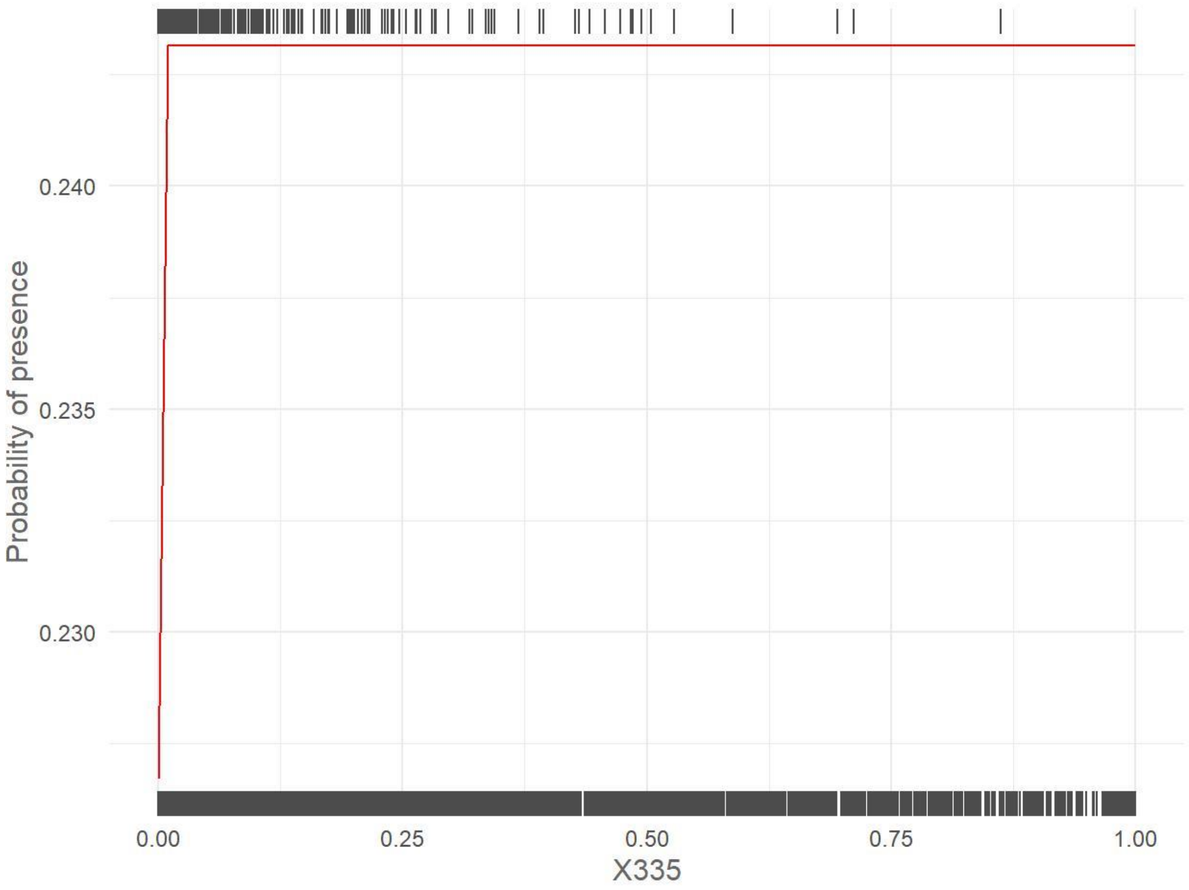

Probability of presence
0.240
0.235
0.230
0.00
0.25
0.50
0.75
1.00
X335

Figure S11. Species-habitat relationships according to the Boosted Regression Trees model for alpine accentor.

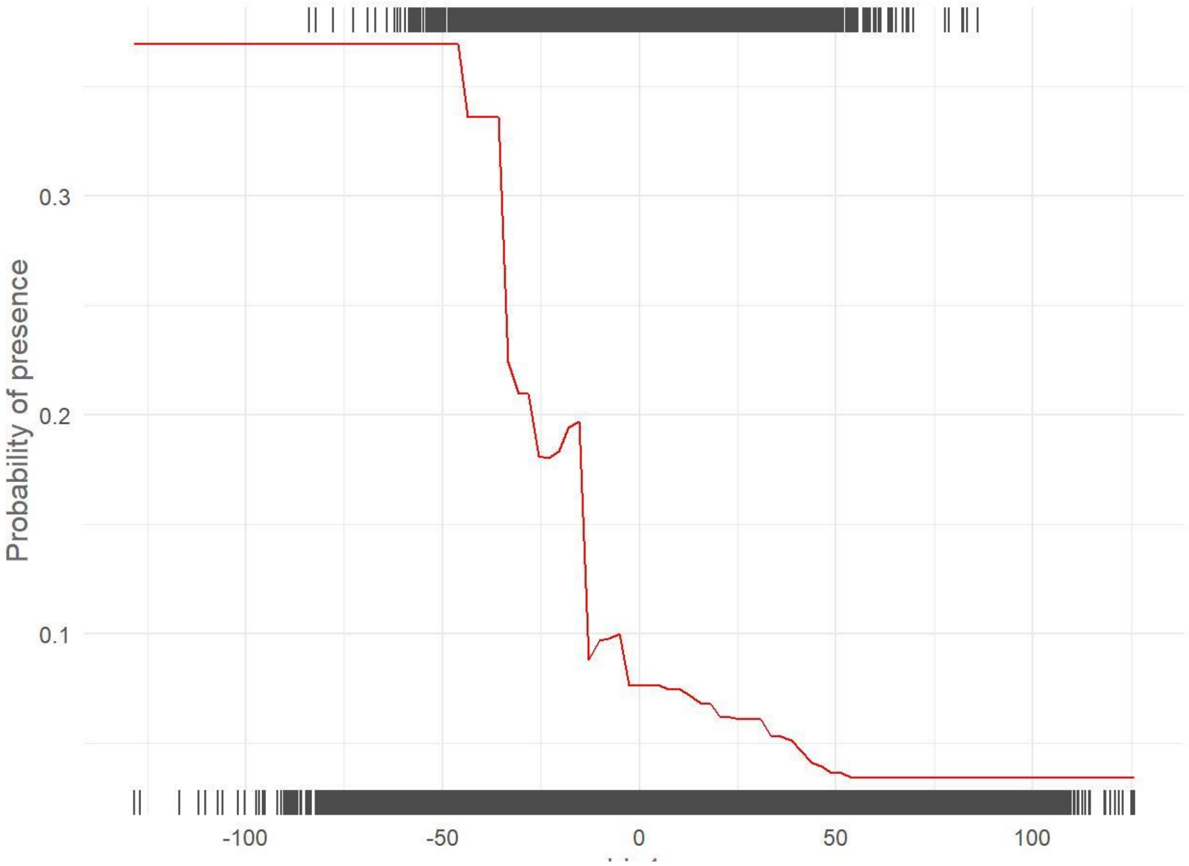

Probability of presence
0.3
0.2
0.1
-100
-50
0
50
100

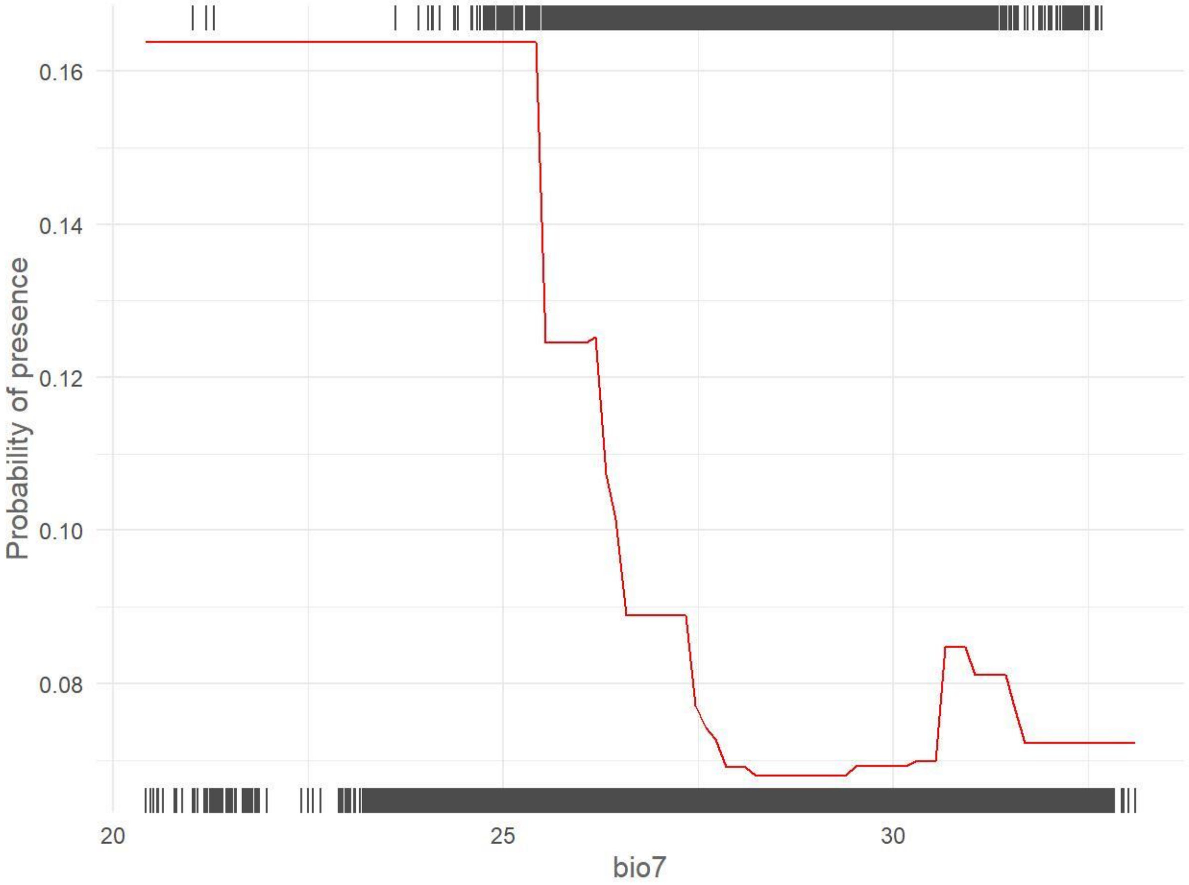

Probability of presence
0.16
0.14
0.12
0.10
0.08
20
25
30
bio7

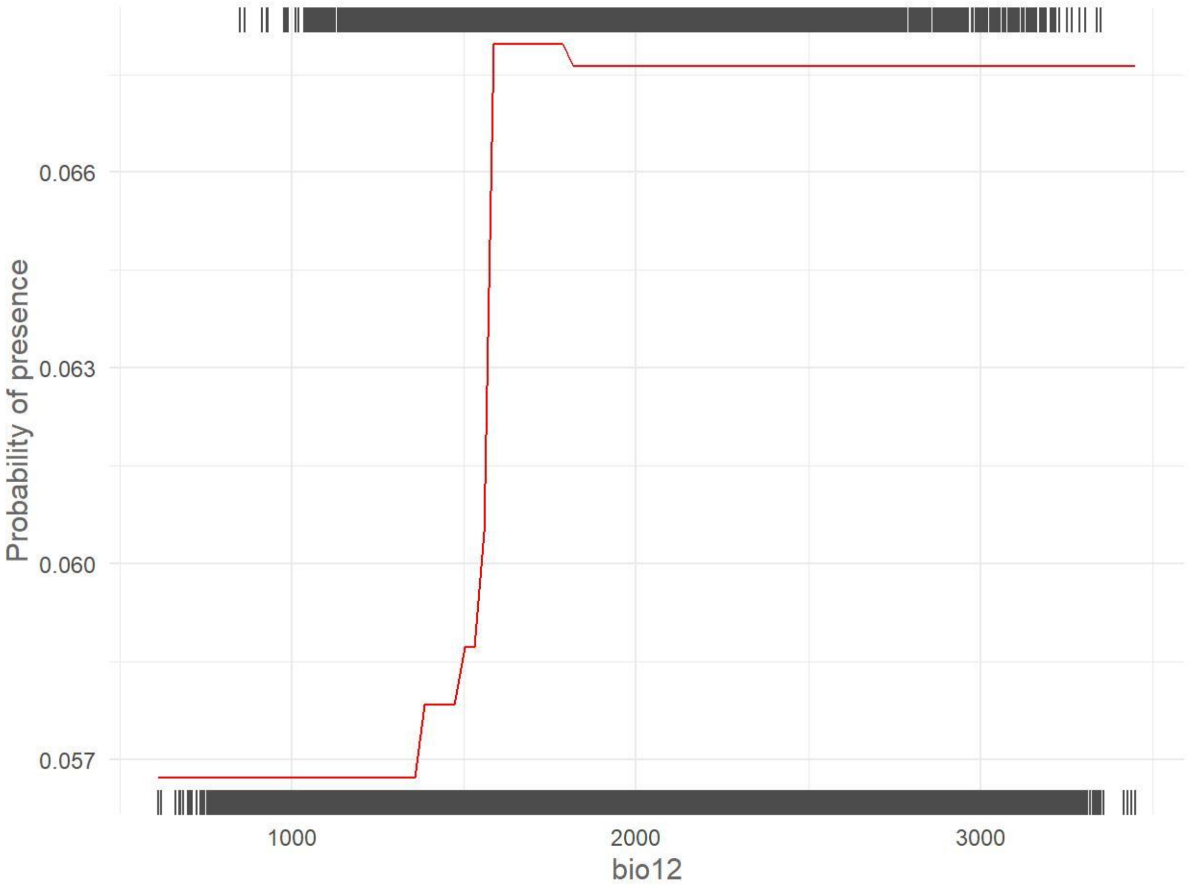
Probability of presence
0.066
0.063
0.060
0.057
1000
2000
3000
bio12

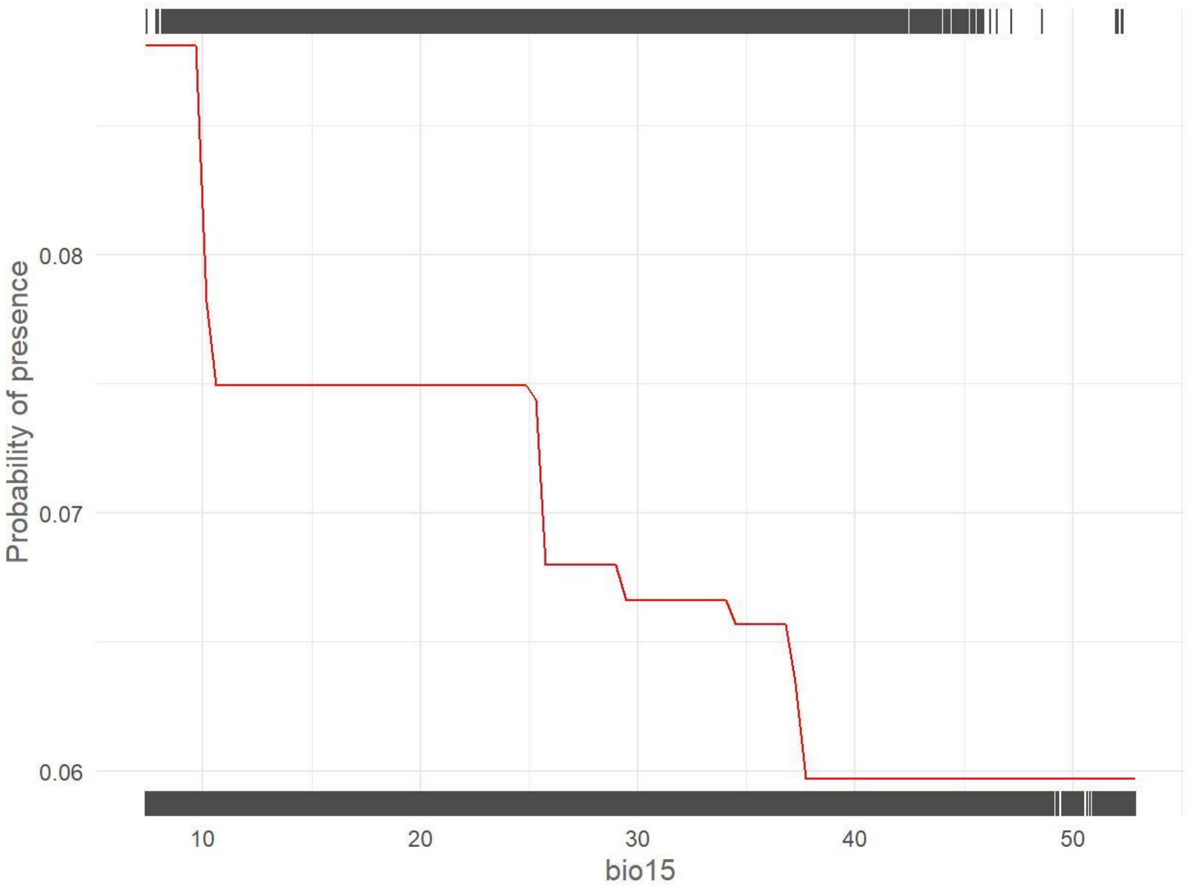
Probability of presence
0.08
0.07
0.06
10
20
30
40
50
bio15

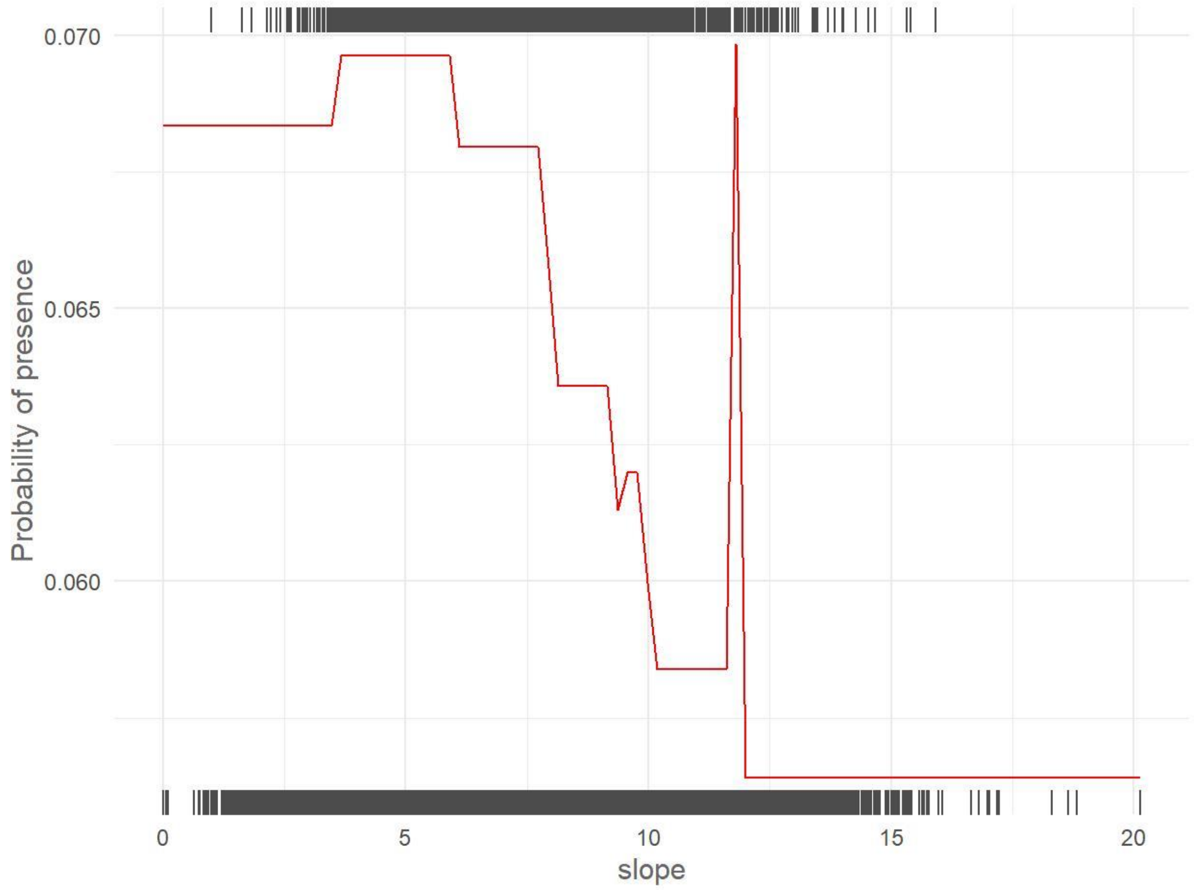
0.070
0.065
0.060
Probability of presence
0
5
10
15
20
slope
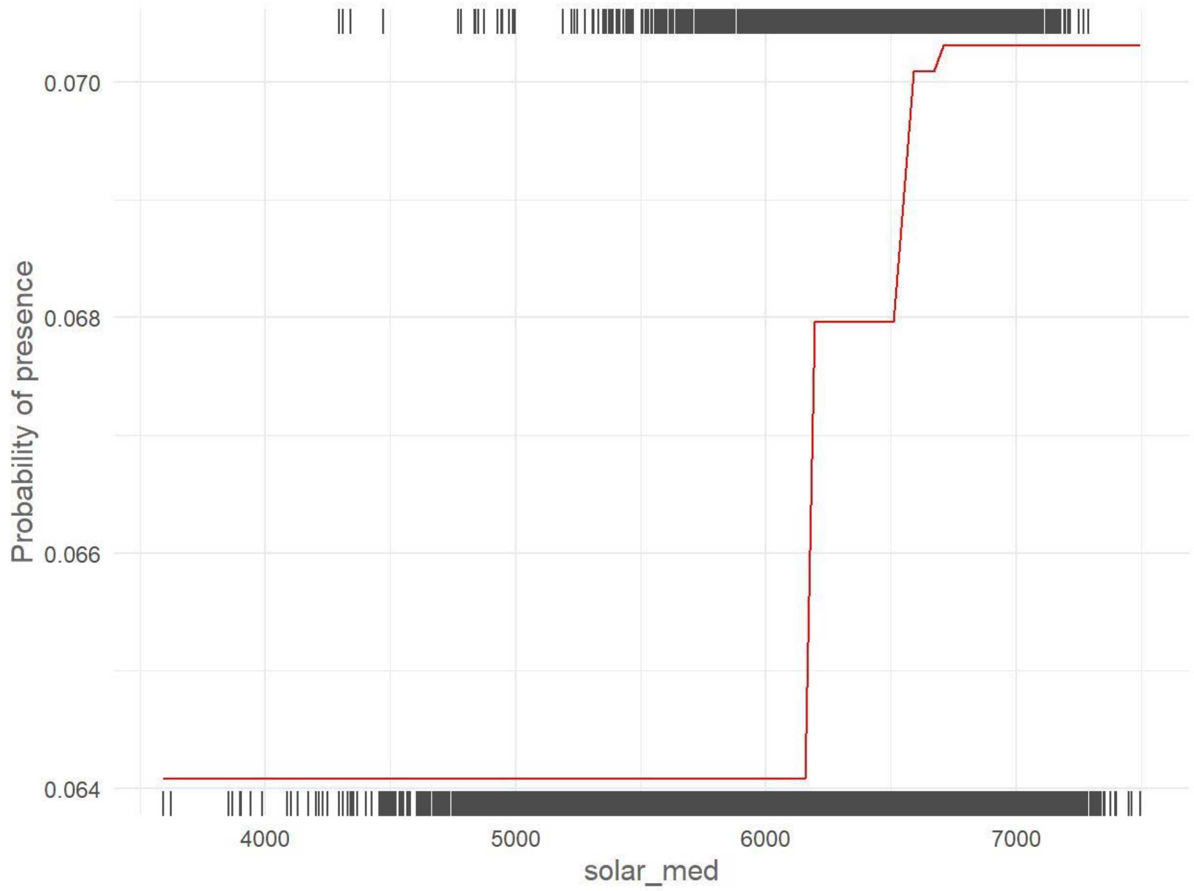
0.070
0.068
0.066
0.064
Probability of presence
4000
5000
6000
7000
solar_med

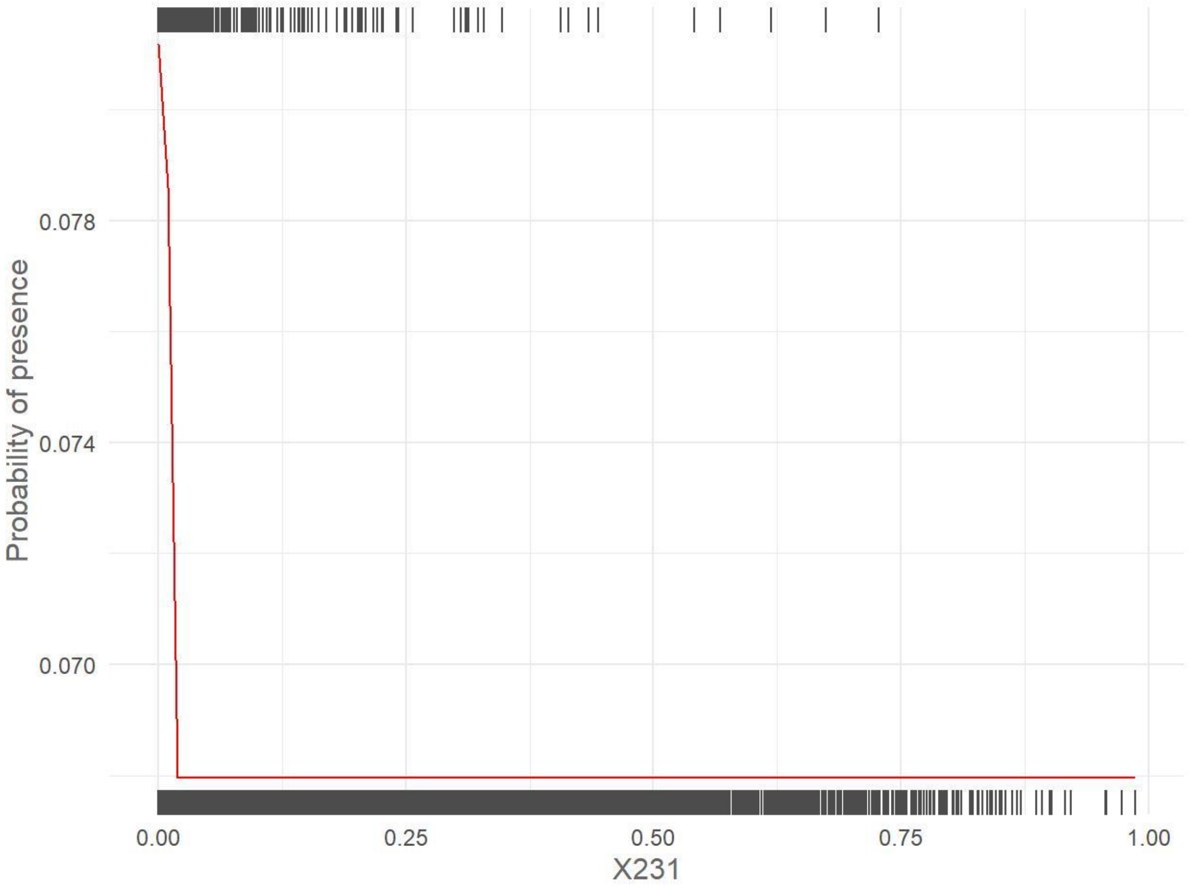

Probability of presence
0.078
0.074
0.070
0.00
0.25
0.50
0.75
1.00
X231

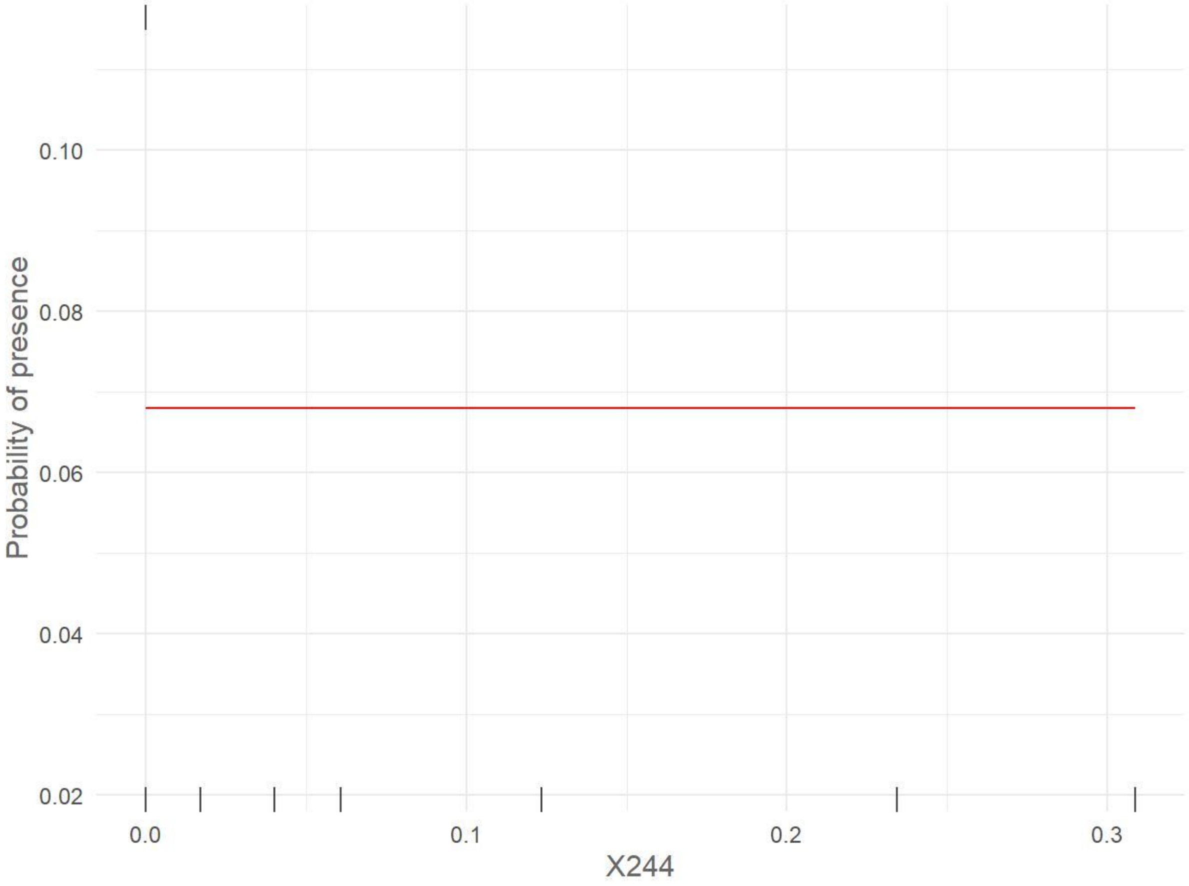

Probability of presence
0.10
0.08
0.06
0.04
0.02
0.0
0.1
0.2
0.3
X244

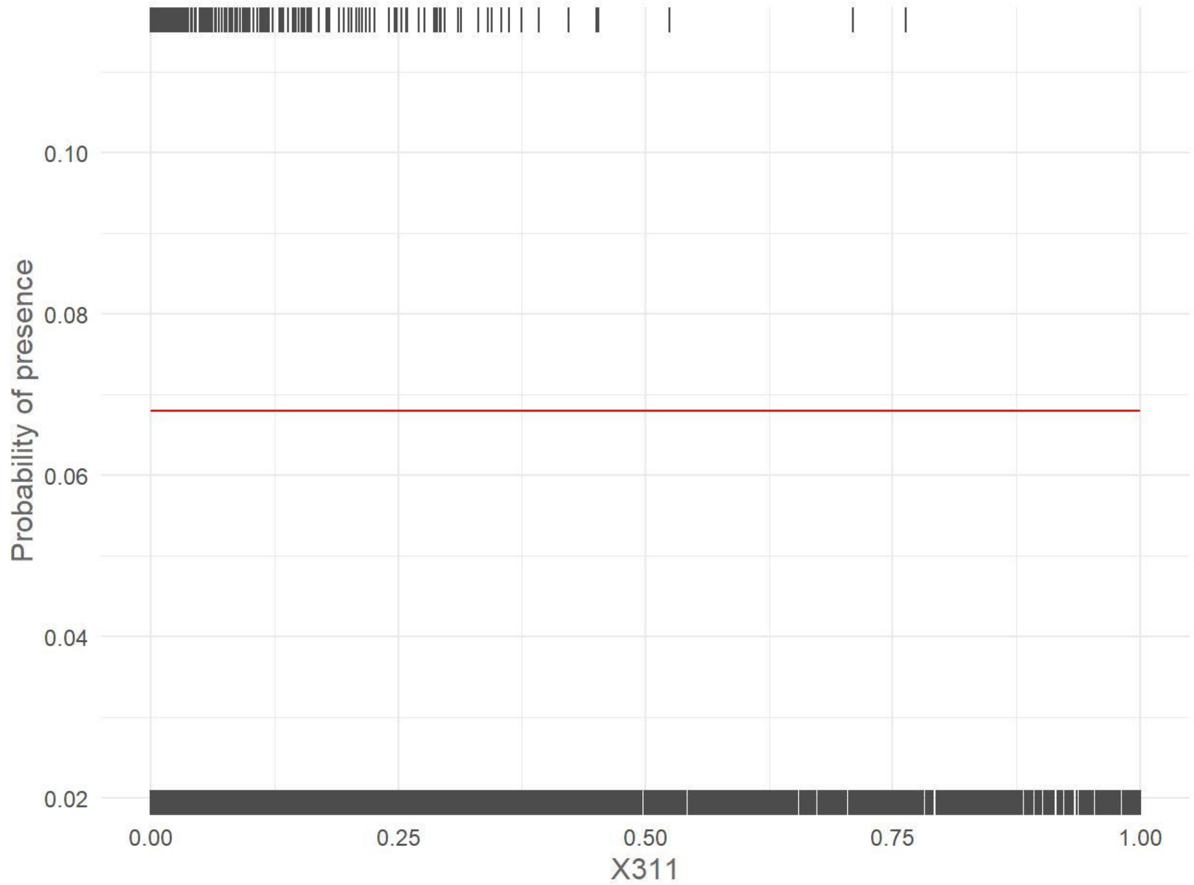
0.10
0.08
0.06
0.04
0.02
Probability of presence
0.00
0.25
0.50
0.75
1.00
X311

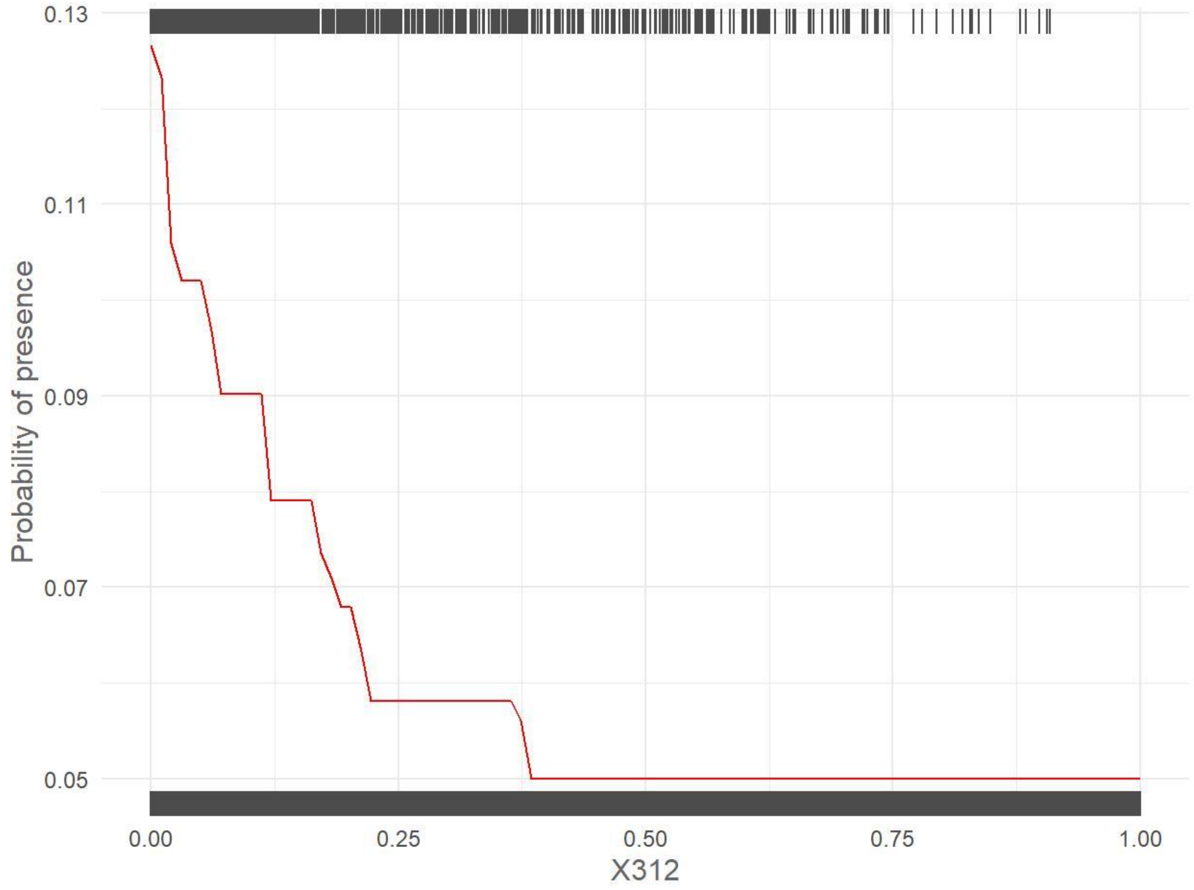
0.13
0.11
0.09
0.07
0.05
Probability of presence
0.00
0.25
0.50
0.75
1.00
X312

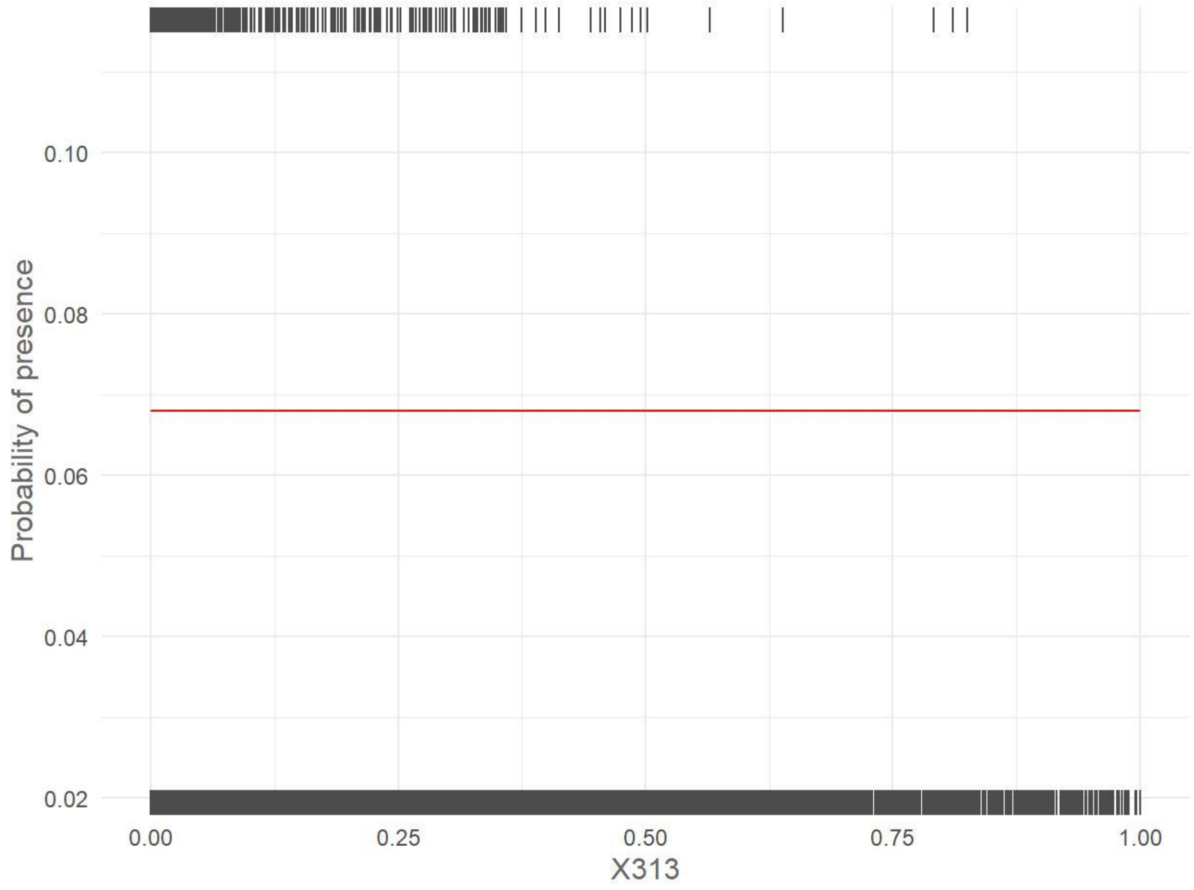

0.10
0.08
0.06
0.04
0.02
Probability of presence
0.00
0.25
0.50
0.75
1.00
X313

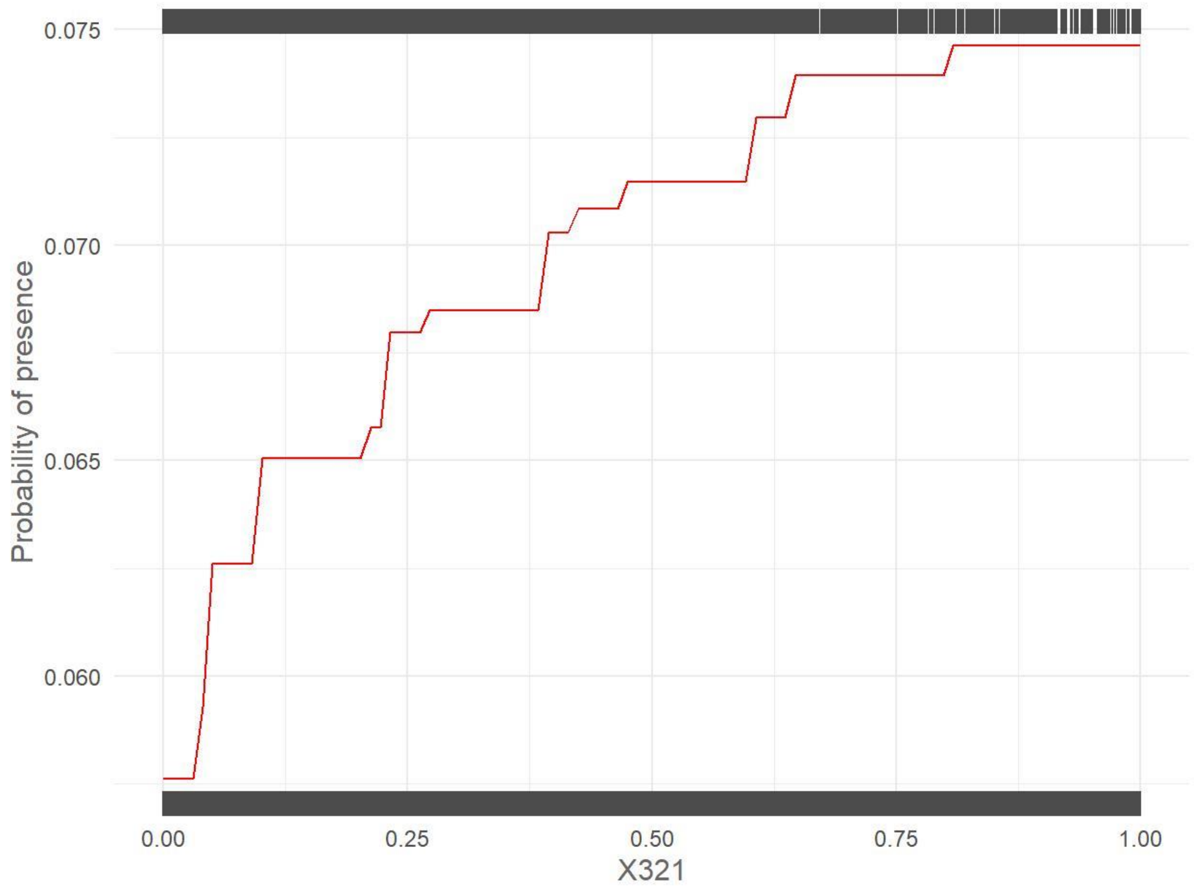

0.075
0.070
0.065
0.060
Probability of presence
0.00
0.25
0.50
0.75
1.00
X321

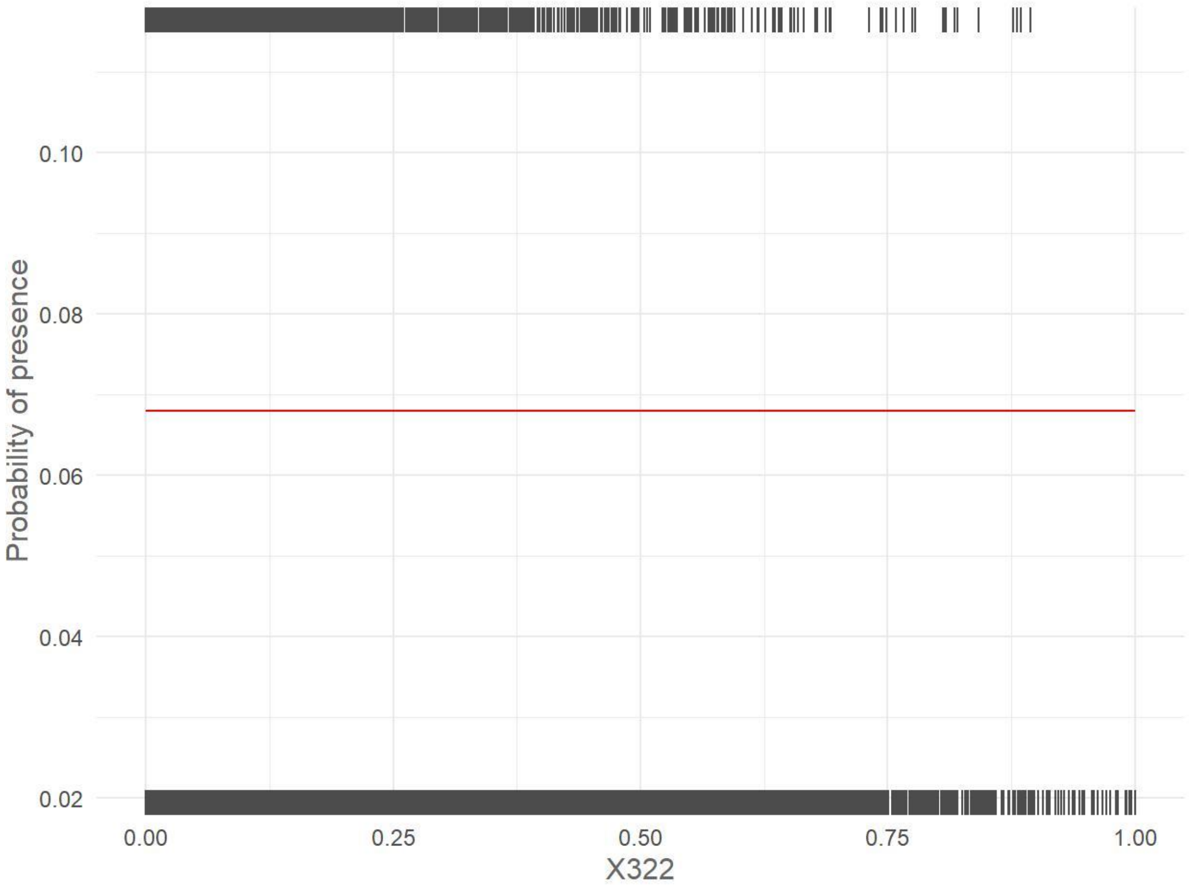

Probability of presence
0.10
0.08
0.06
0.04
0.02
0.00
0.25
0.50
0.75
1.00
X322

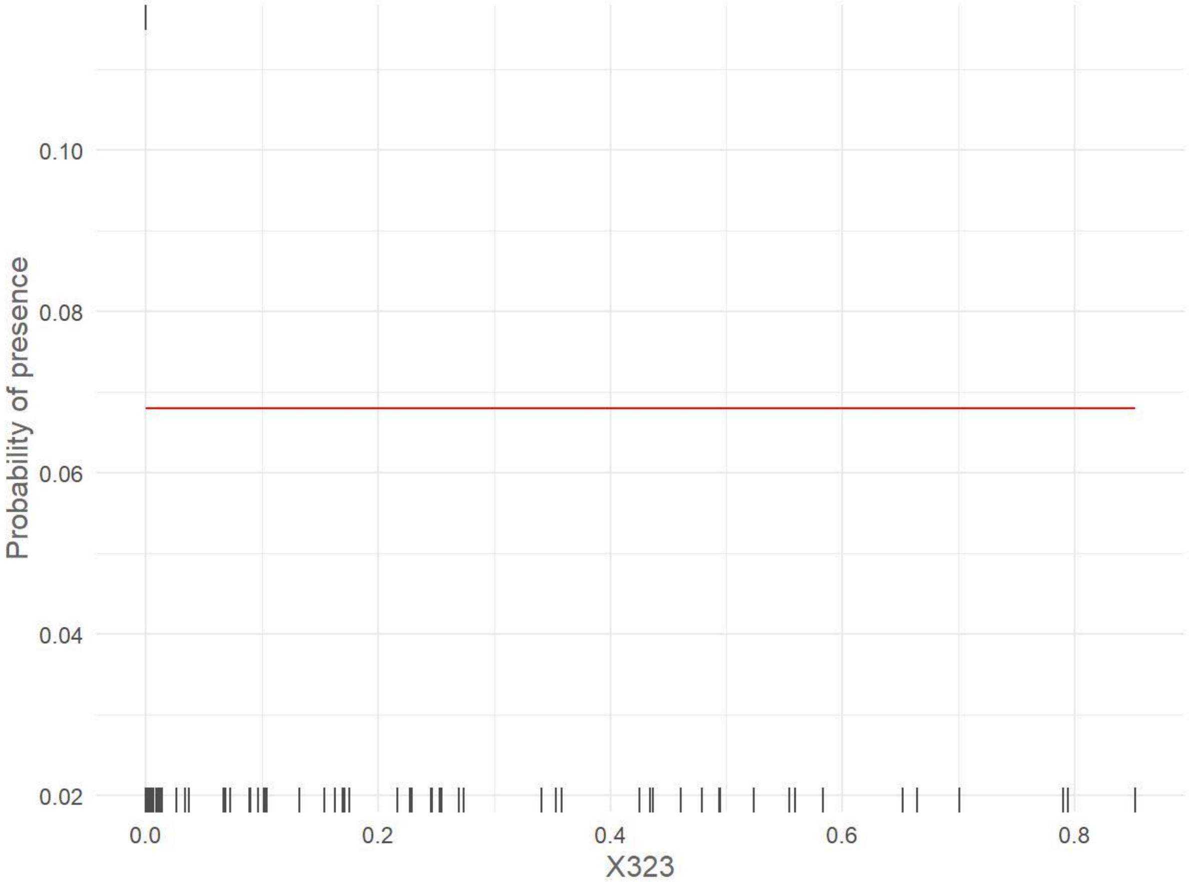

Probability of presence
0.10
0.08
0.06
0.04
0.02
0.0
0.2
0.4
0.6
0.8
X323

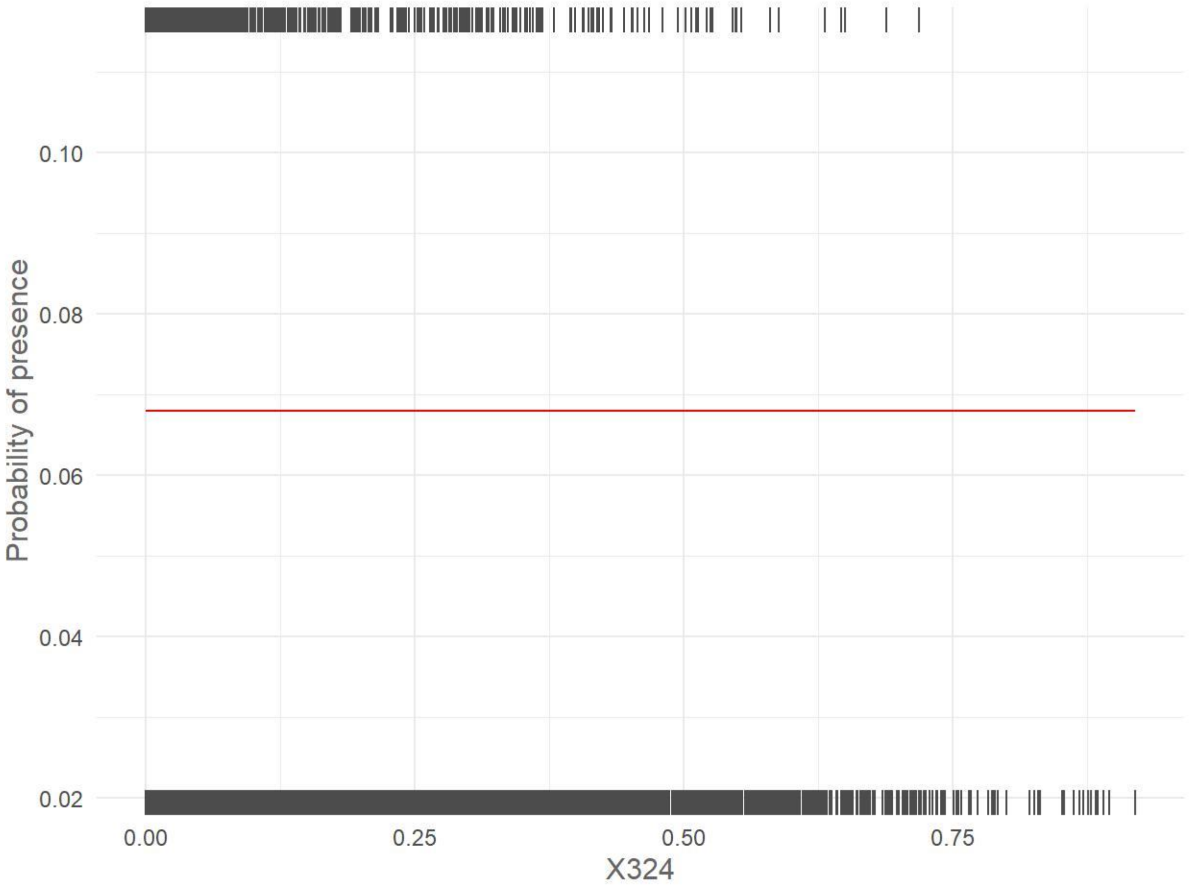

0.10
0.08
0.06
0.04
0.02
Probability of presence
0.00
0.25
0.50
0.75
X324

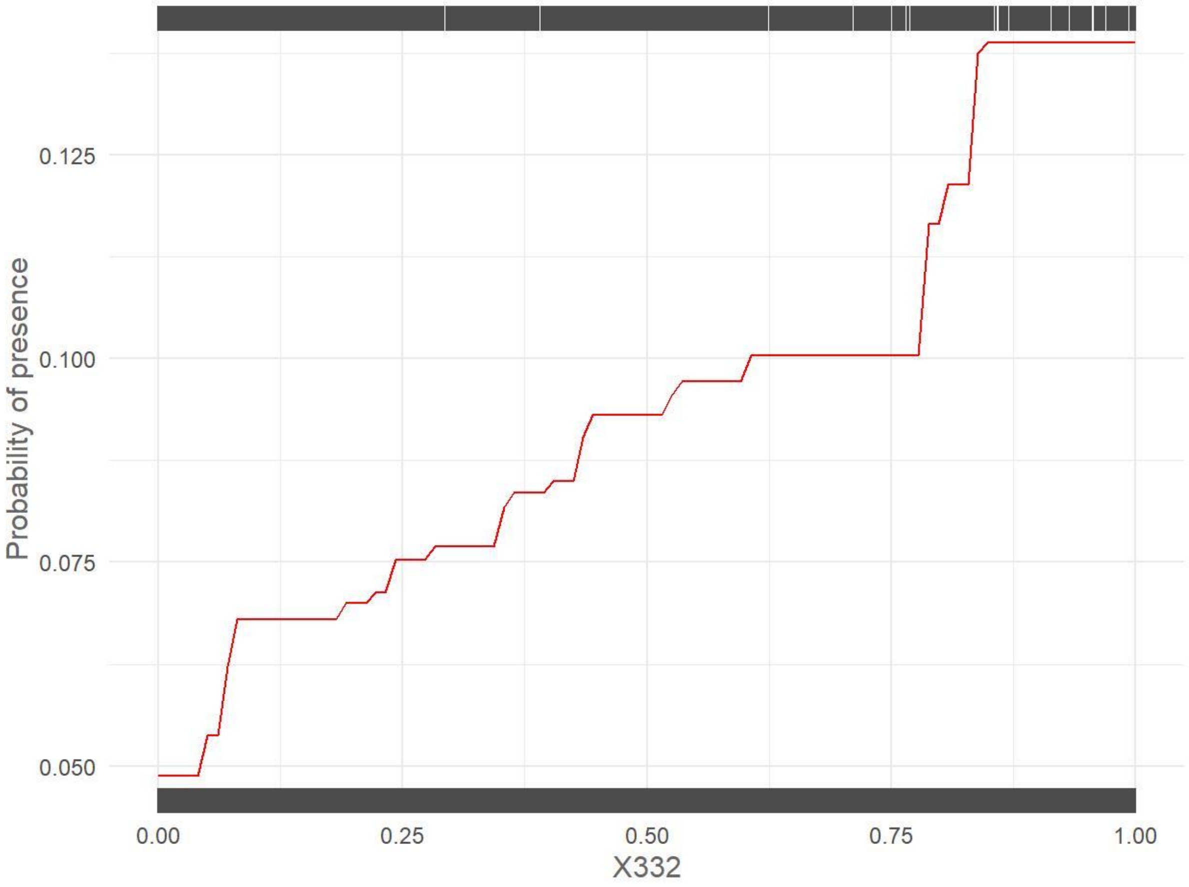

0.125
0.100
0.075
0.050
Probability of presence
0.00
0.25
0.50
0.75
1.00
X332

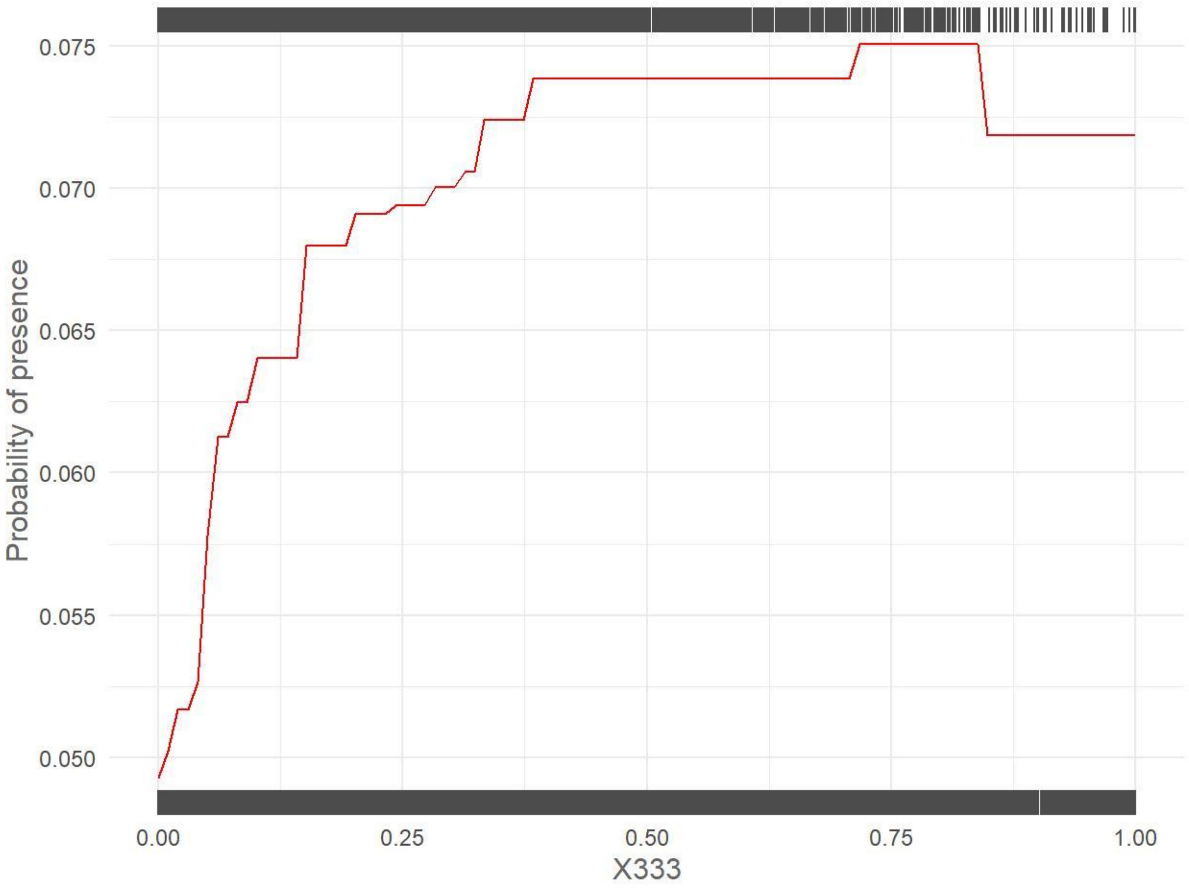

Probability of presence
0.075
0.070
0.065
0.060
0.055
0.050
0.00
0.25
0.50
0.75
1.00
X333

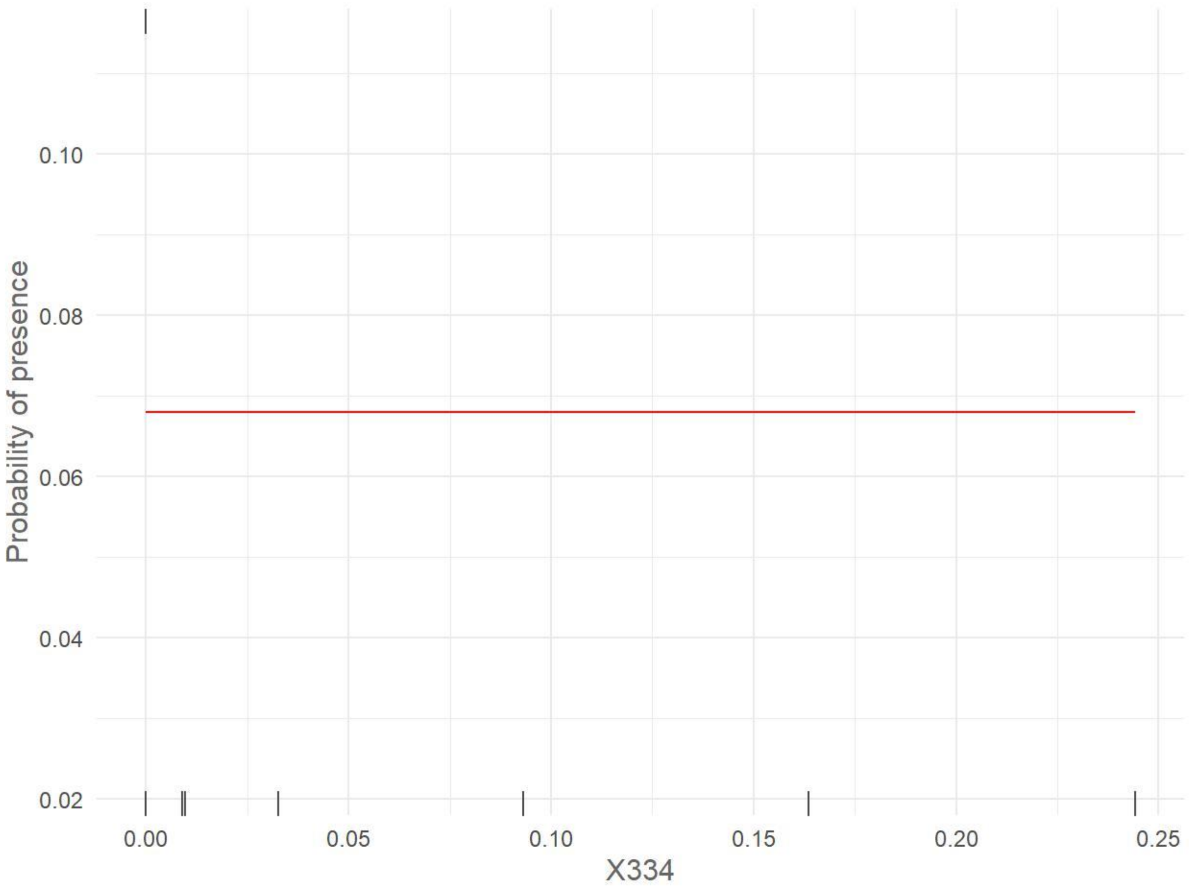

Probability of presence
0.10
0.08
0.06
0.04
0.02
0.00
0.05
0.10
0.15
0.20
0.25
X334

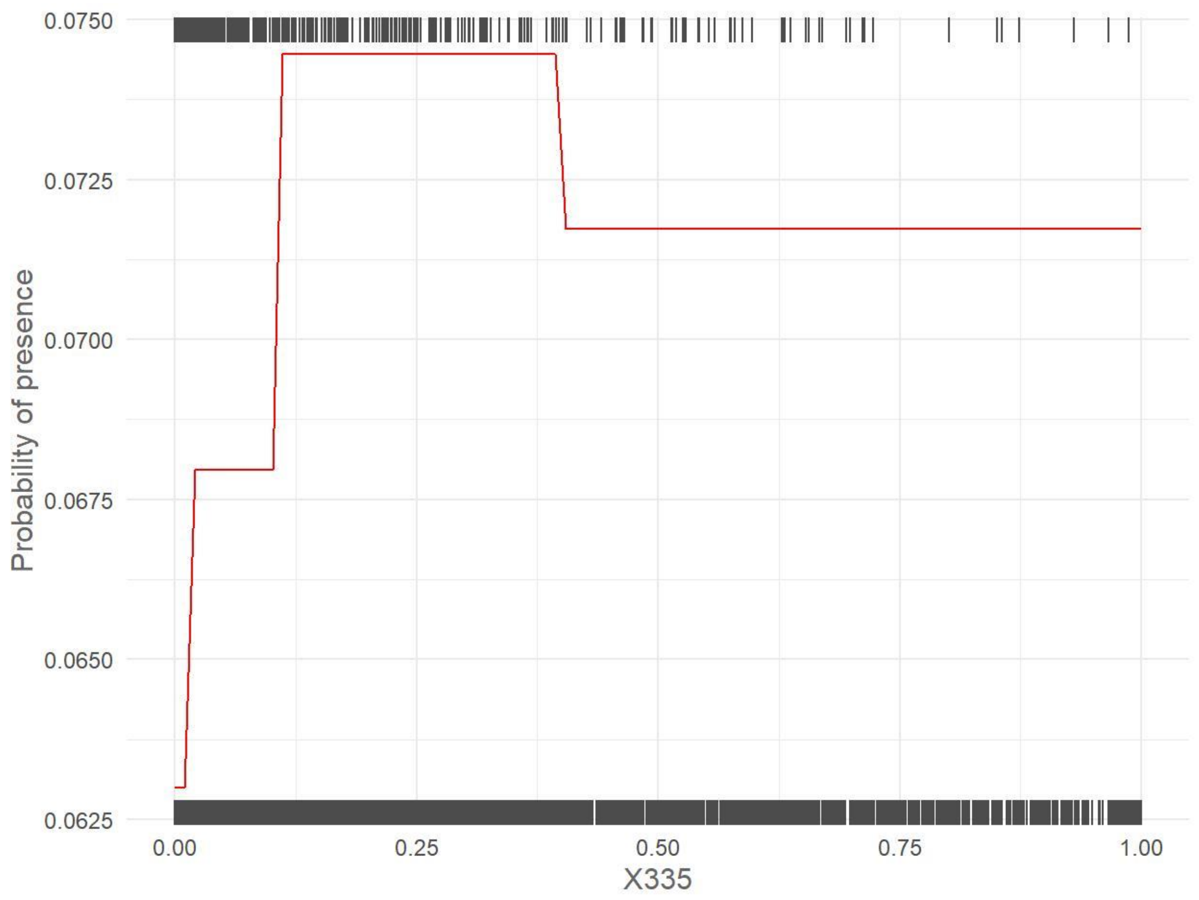

0.0750
0.0725
0.0700
0.0675
0.0650
0.0625
Probability of presence
0.00
0.25
0.50
0.75
1.00
X335

Figure S12. Species-habitat relationships according to the Boosted Regression Trees model for white-winged snowfinch.

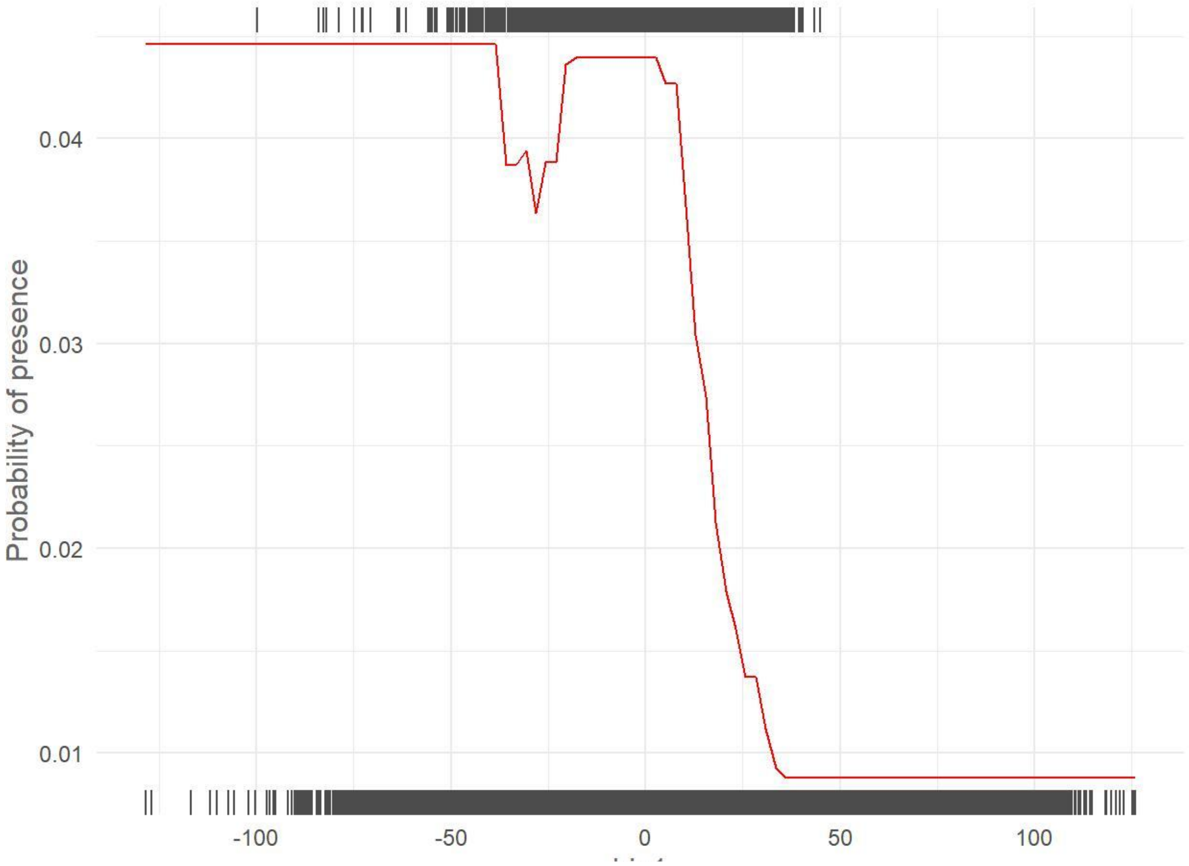
Probability of presence
0.04
0.03
0.02
0.01
-100
-50
0
50
100

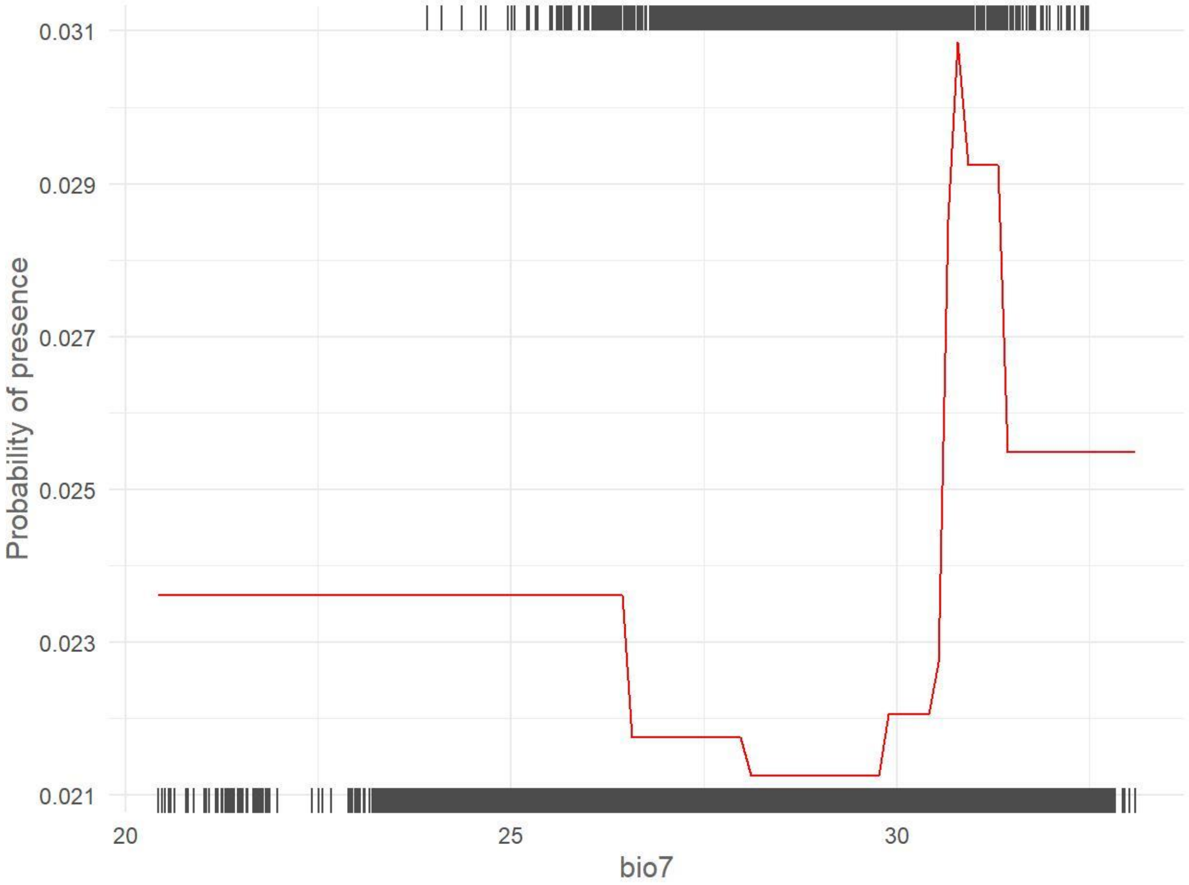
Probability of presence
0.031
0.029
0.027
0.025
0.023
0.021
20
25
30
bio7

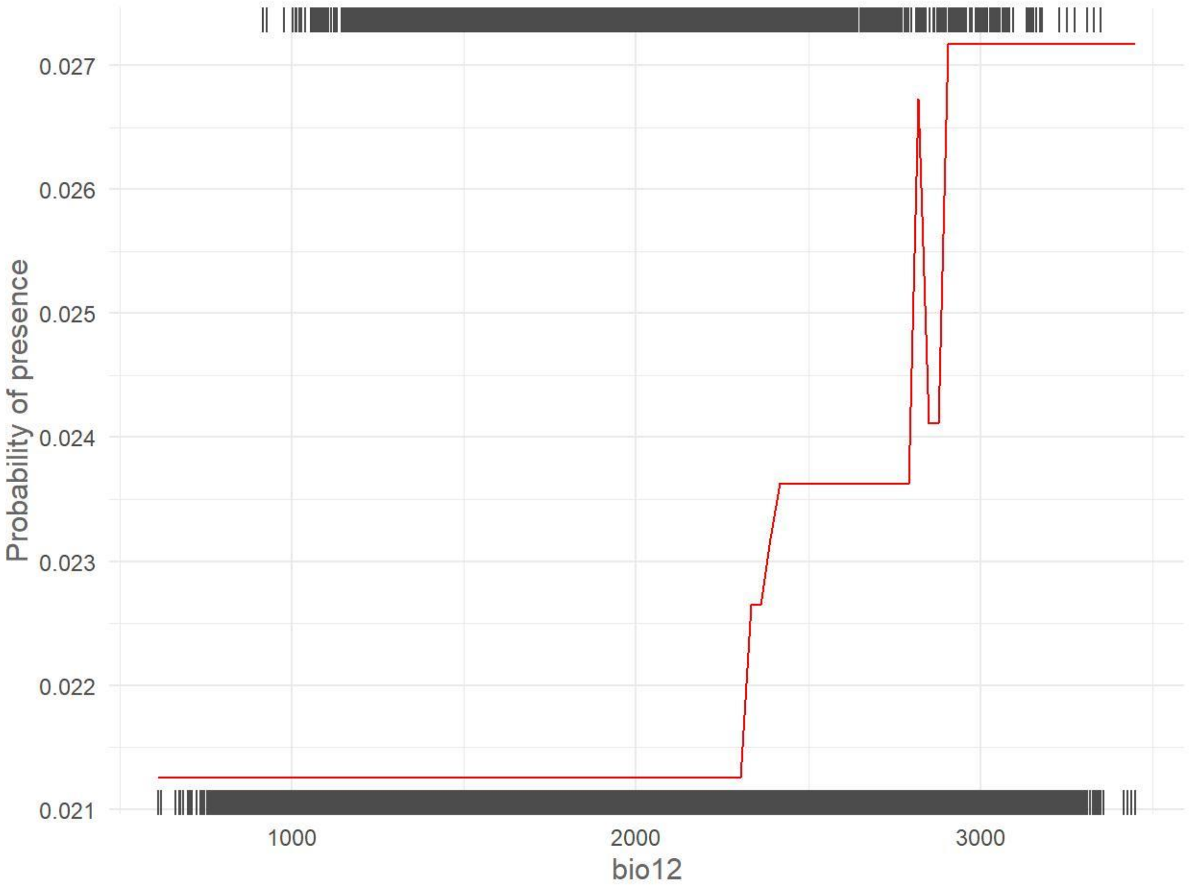
0.027
0.026
0.025
0.024
0.023
0.022
0.021
Probability of presence
1000
2000
3000
bio12

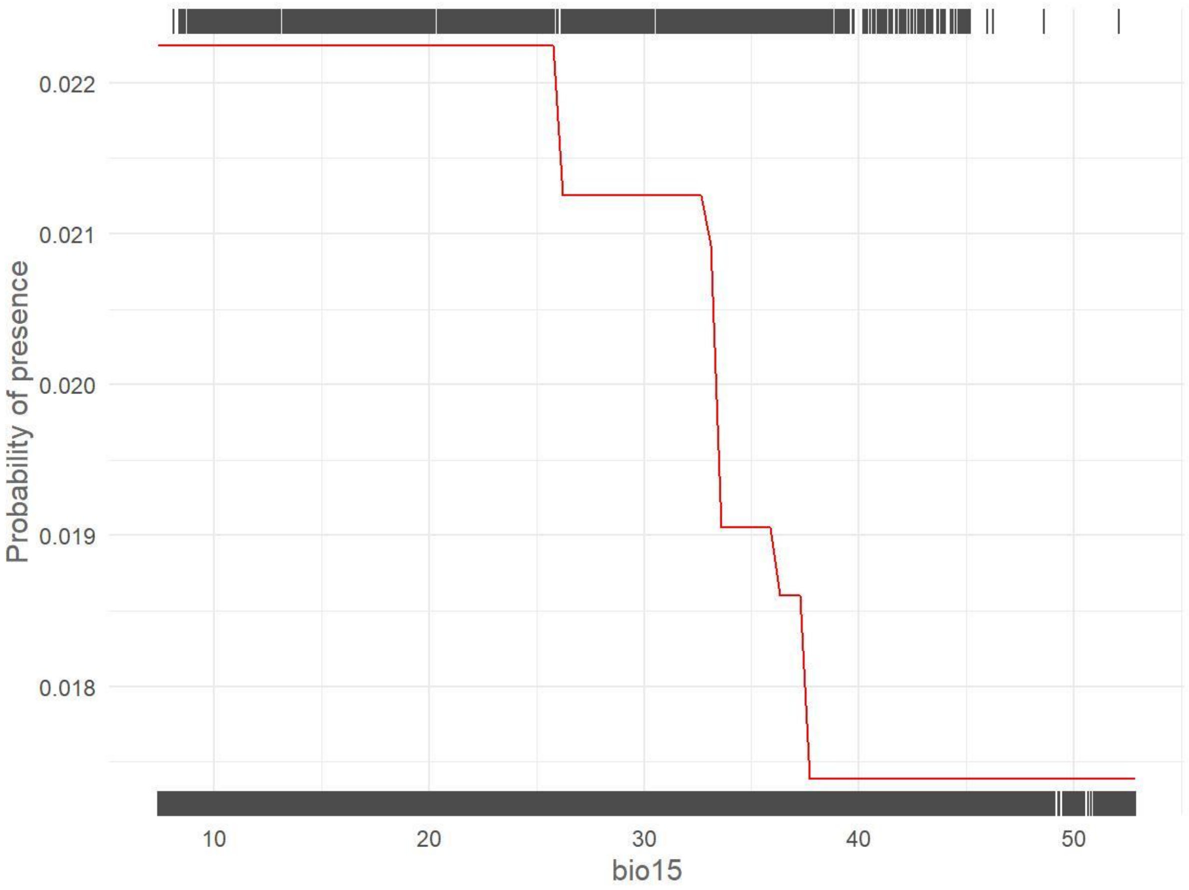
0.022
0.021
0.020
0.019
0.018
Probability of presence
10
20
30
40
50
bio15

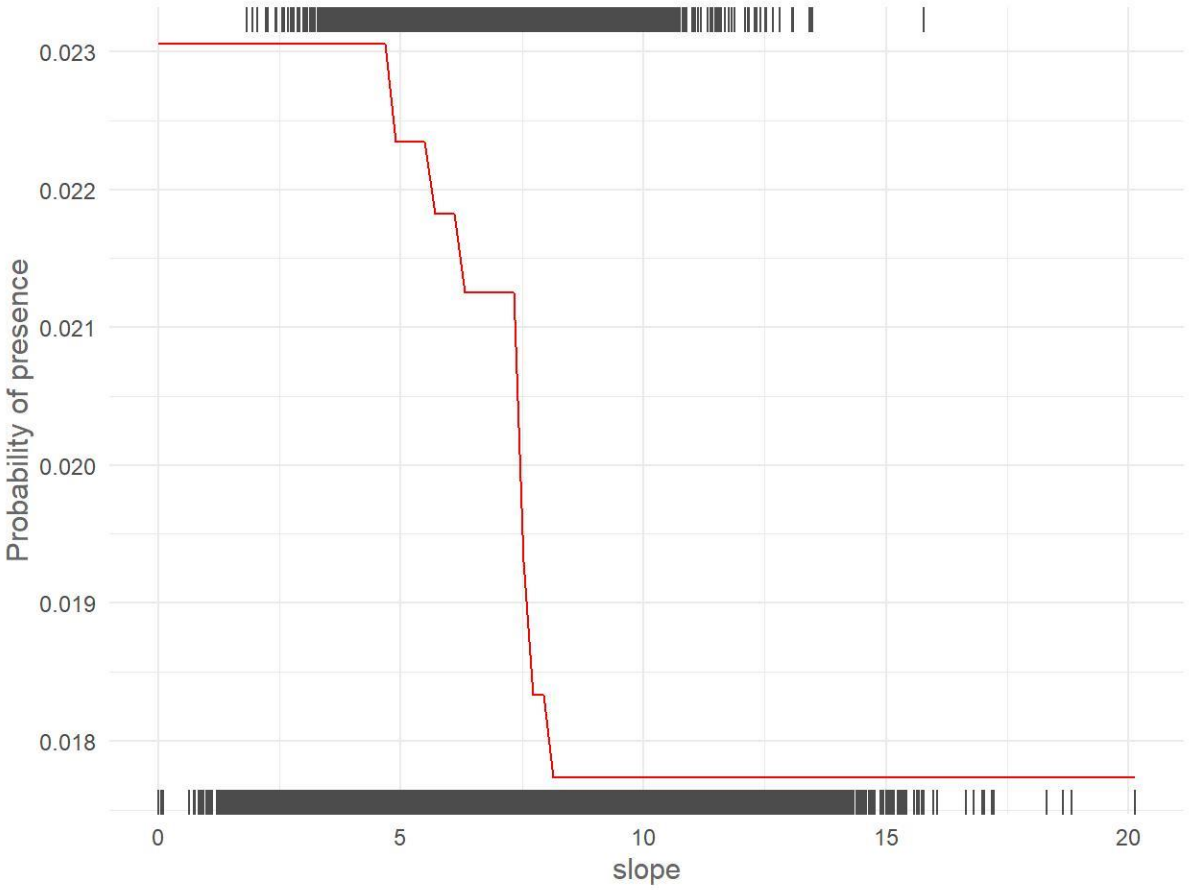
Probability of presence
0.023
0.022
0.021
0.020
0.019
0.018
0
5
10
15
20
slope

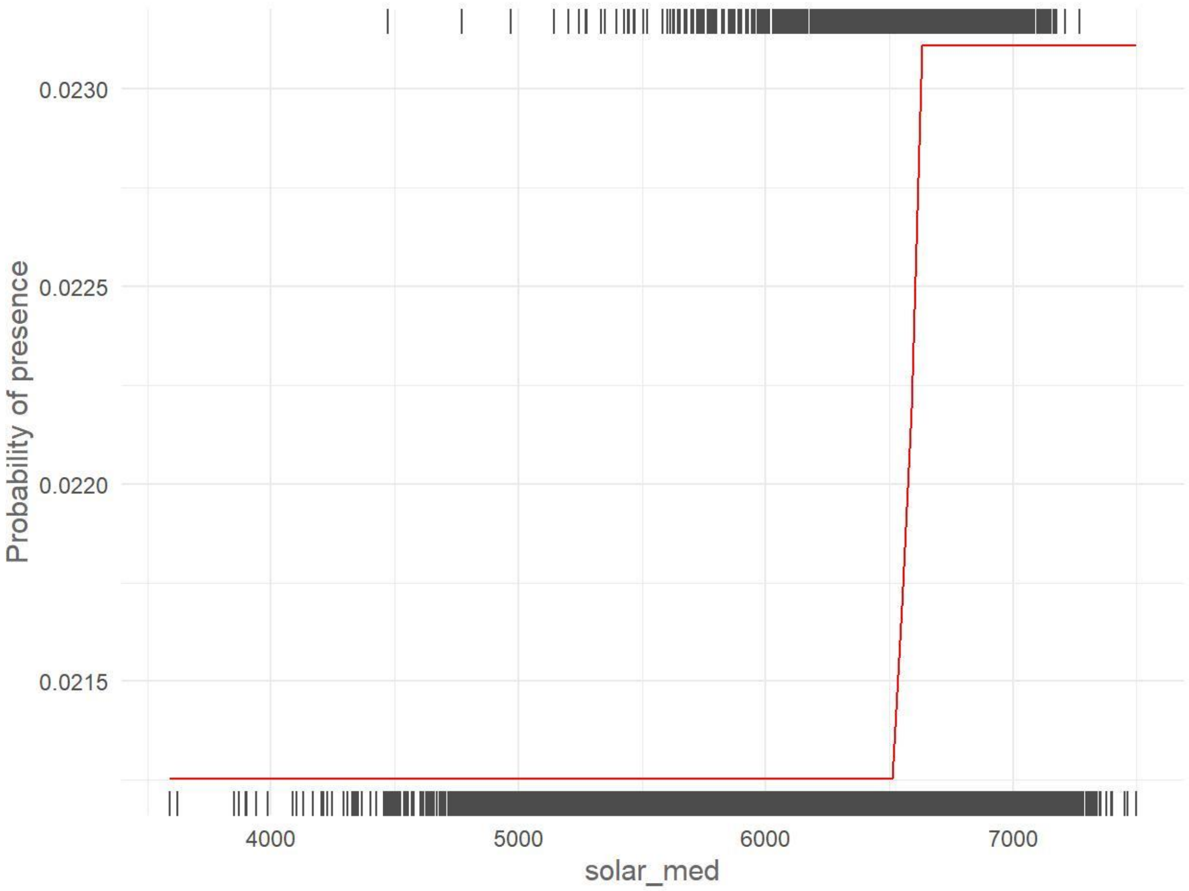
Probability of presence
0.0230
0.0225
0.0220
0.0215
4000
5000
6000
7000
solar_med

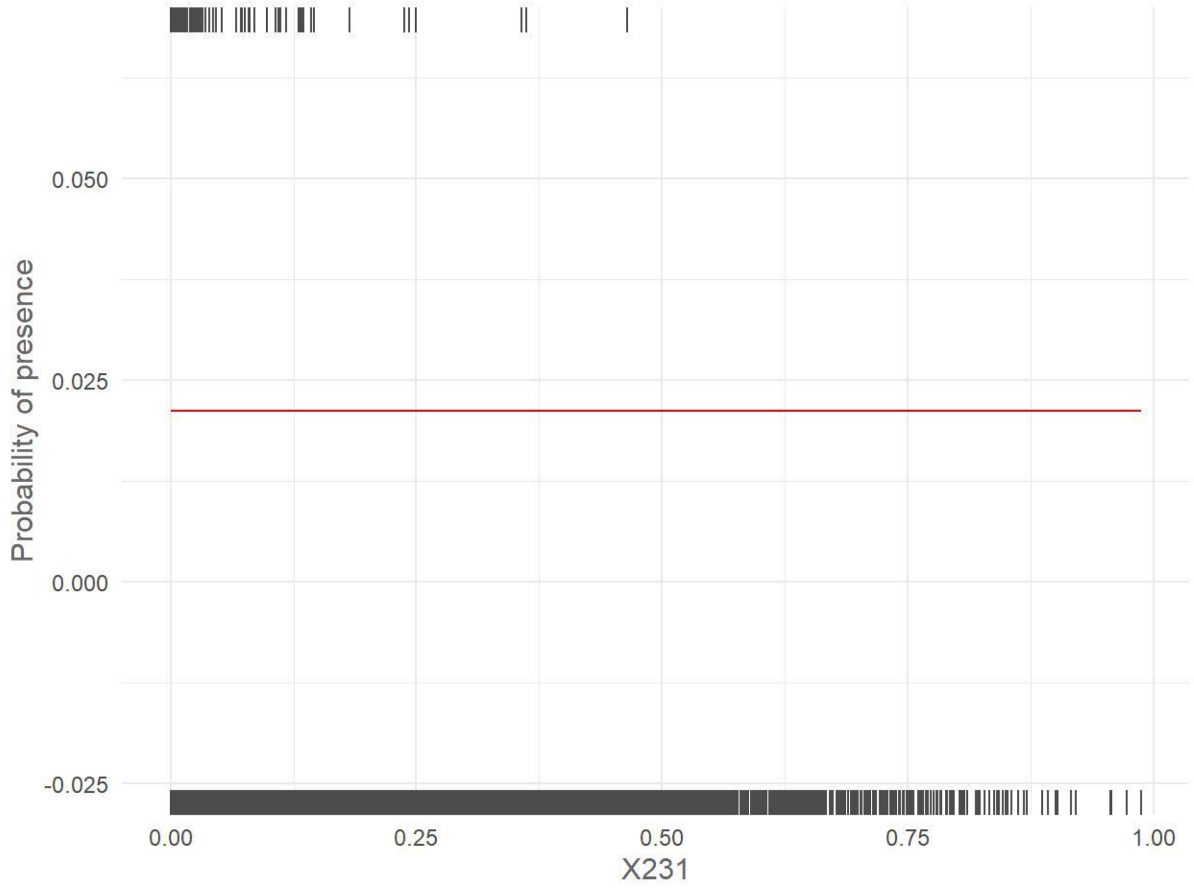

0.050
0.025
0.000
-0.025
Probability of presence
0.00
0.25
0.50
0.75
1.00
X231

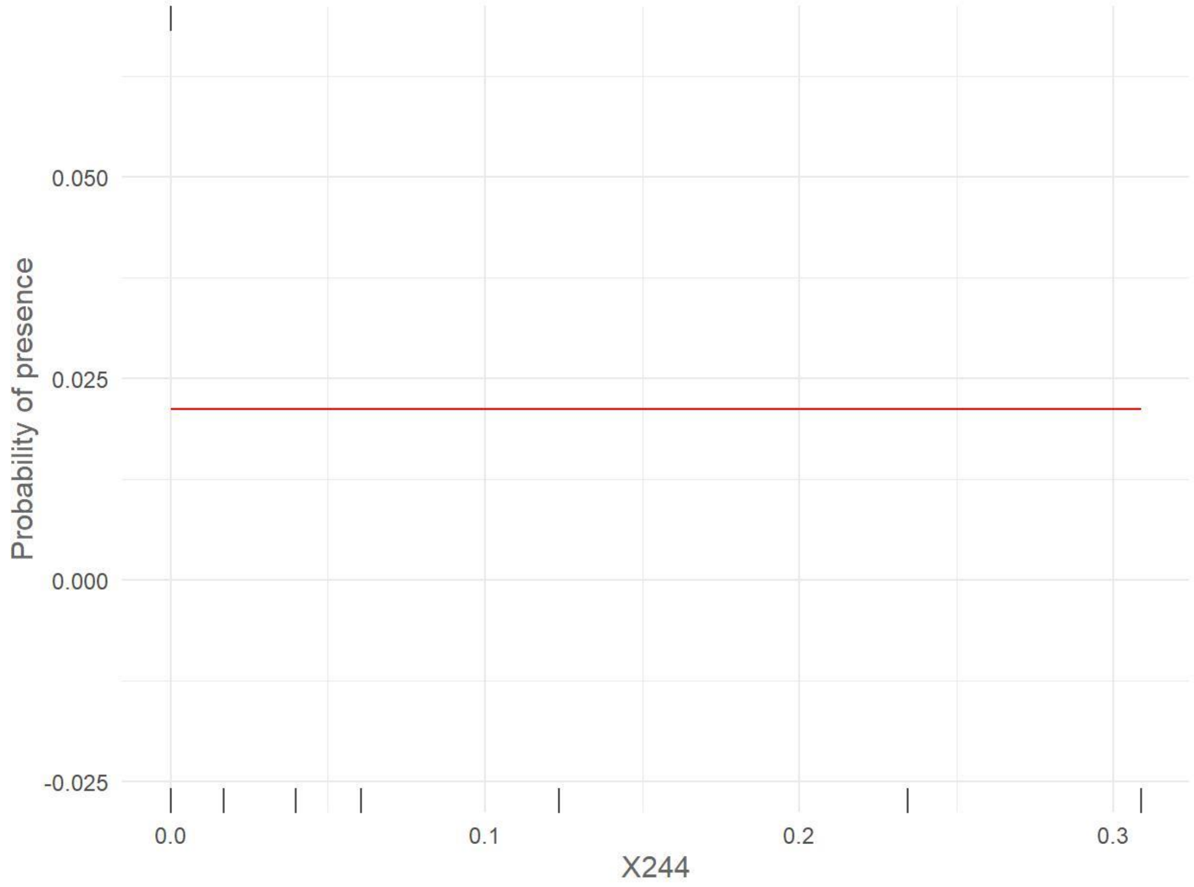

0.050
0.025
0.000
-0.025
Probability of presence
0.0
0.1
0.2
0.3
X244

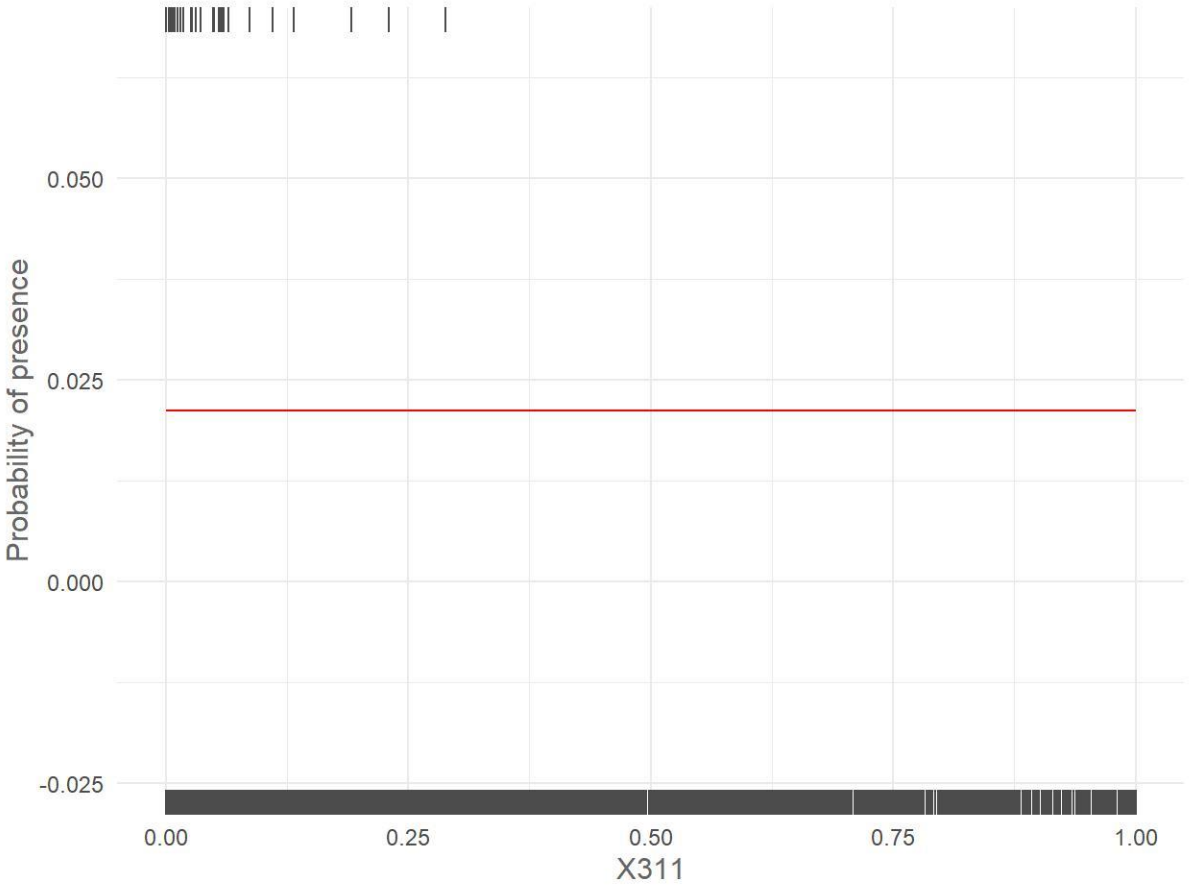
0.050
0.025
0.000
-0.025
Probability of presence
0.00
0.25
0.50
0.75
1.00
X311

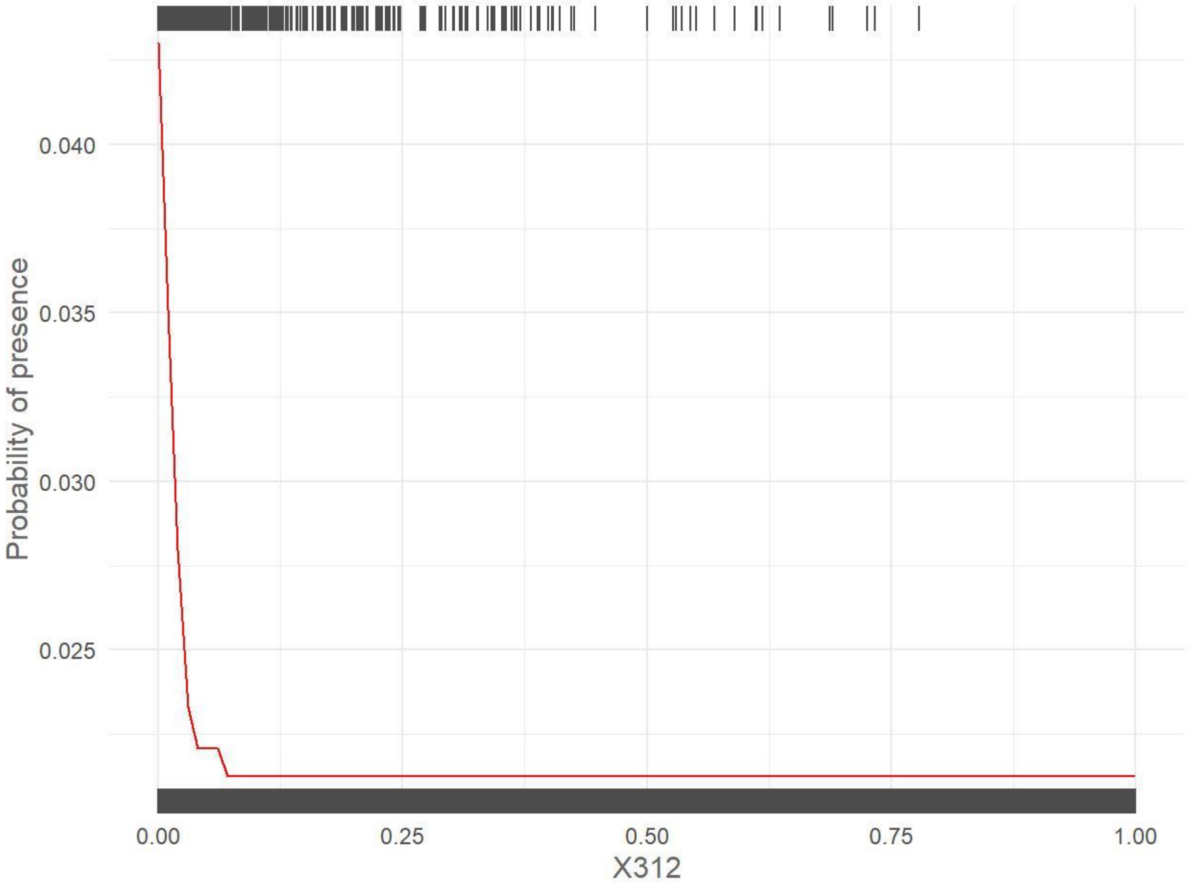
0.040
0.035
0.030
0.025
Probability of presence
0.00
0.25
0.50
0.75
1.00
X312

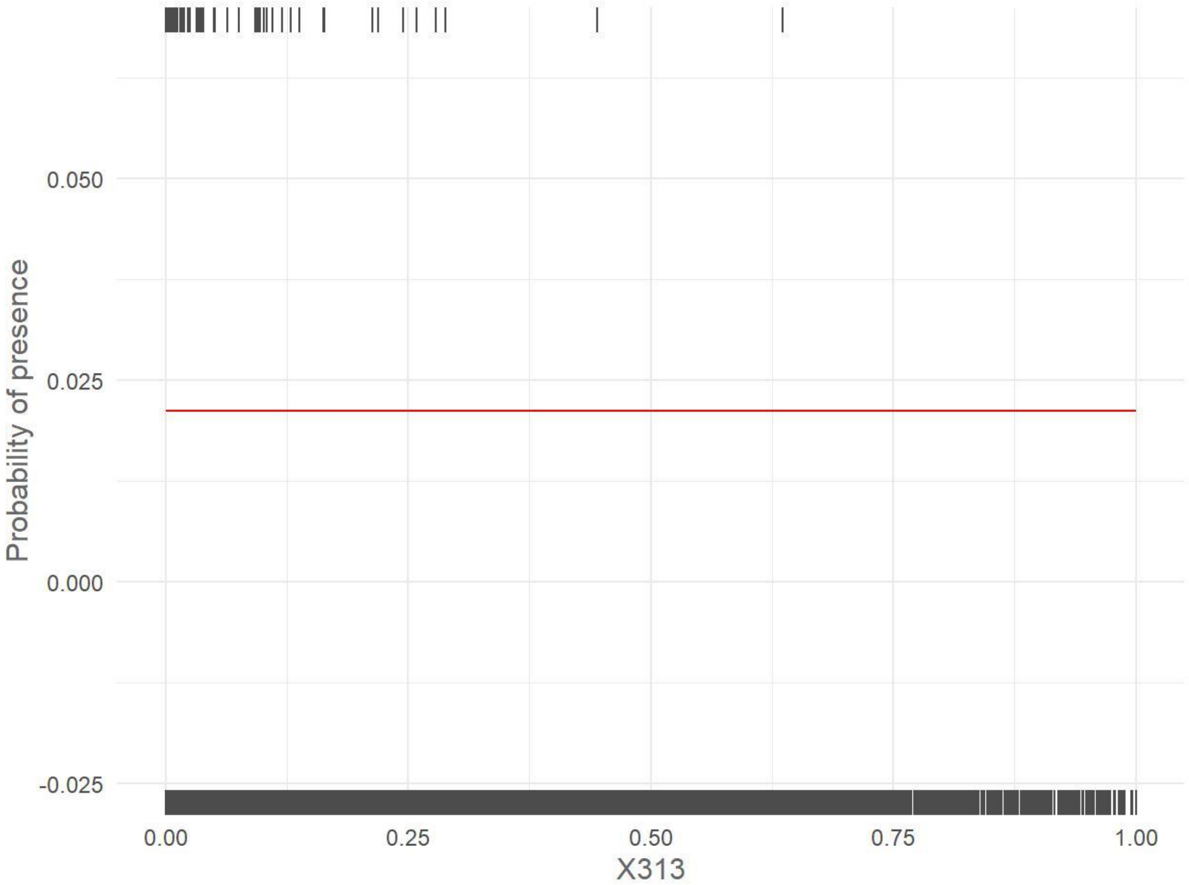

0.050
0.025
0.000
-0.025
Probability of presence
0.00
0.25
0.50
0.75
1.00
X313

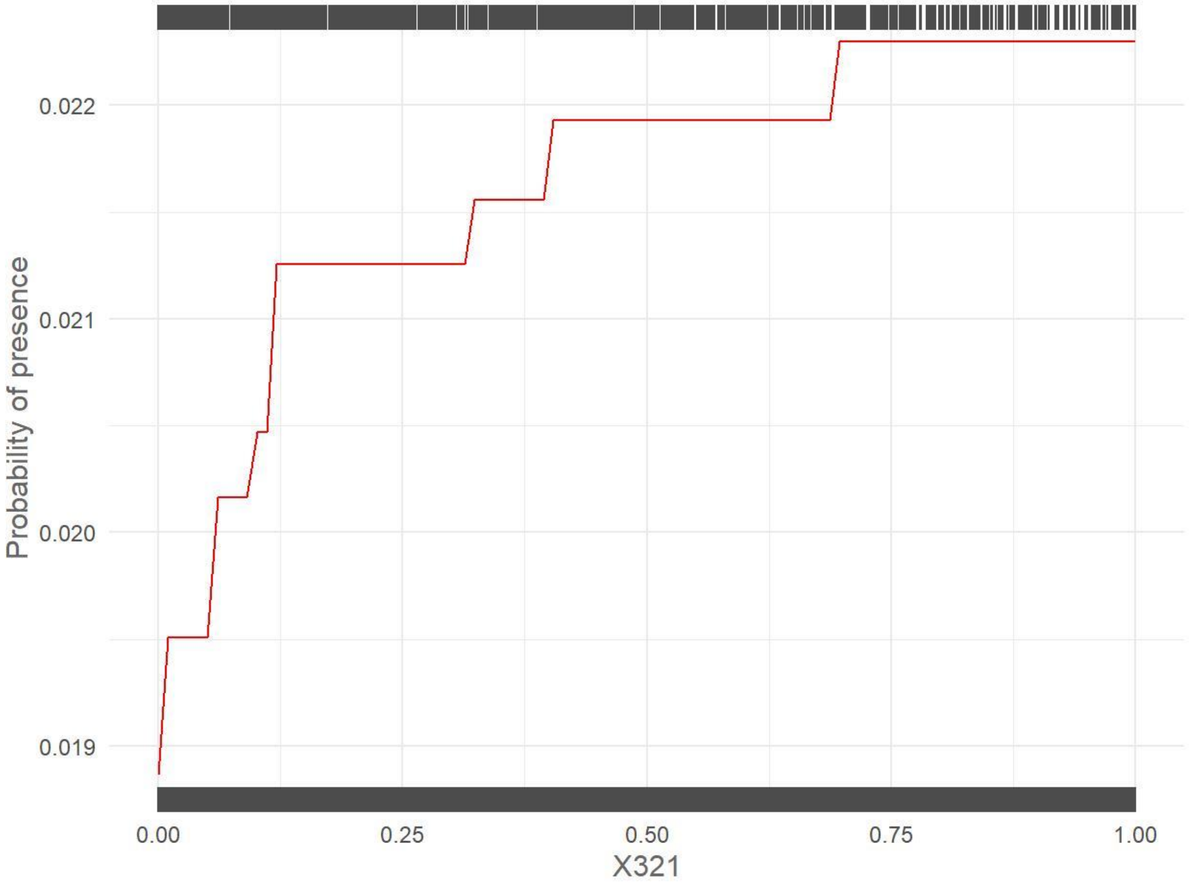

0.022
0.021
0.020
0.019
Probability of presence
0.00
0.25
0.50
0.75
1.00
X321

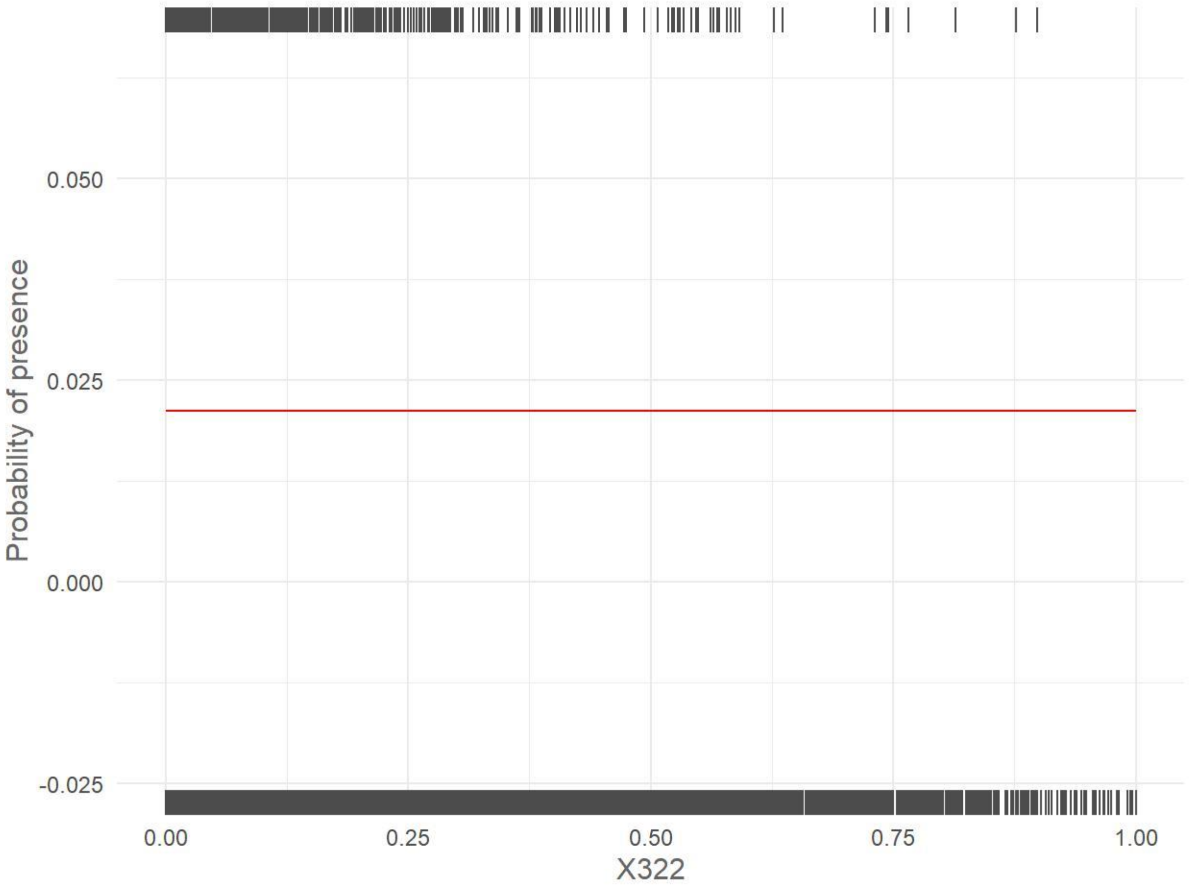
0.050
0.025
0.000
-0.025
Probability of presence
0.00
0.25
0.50
0.75
1.00
X322

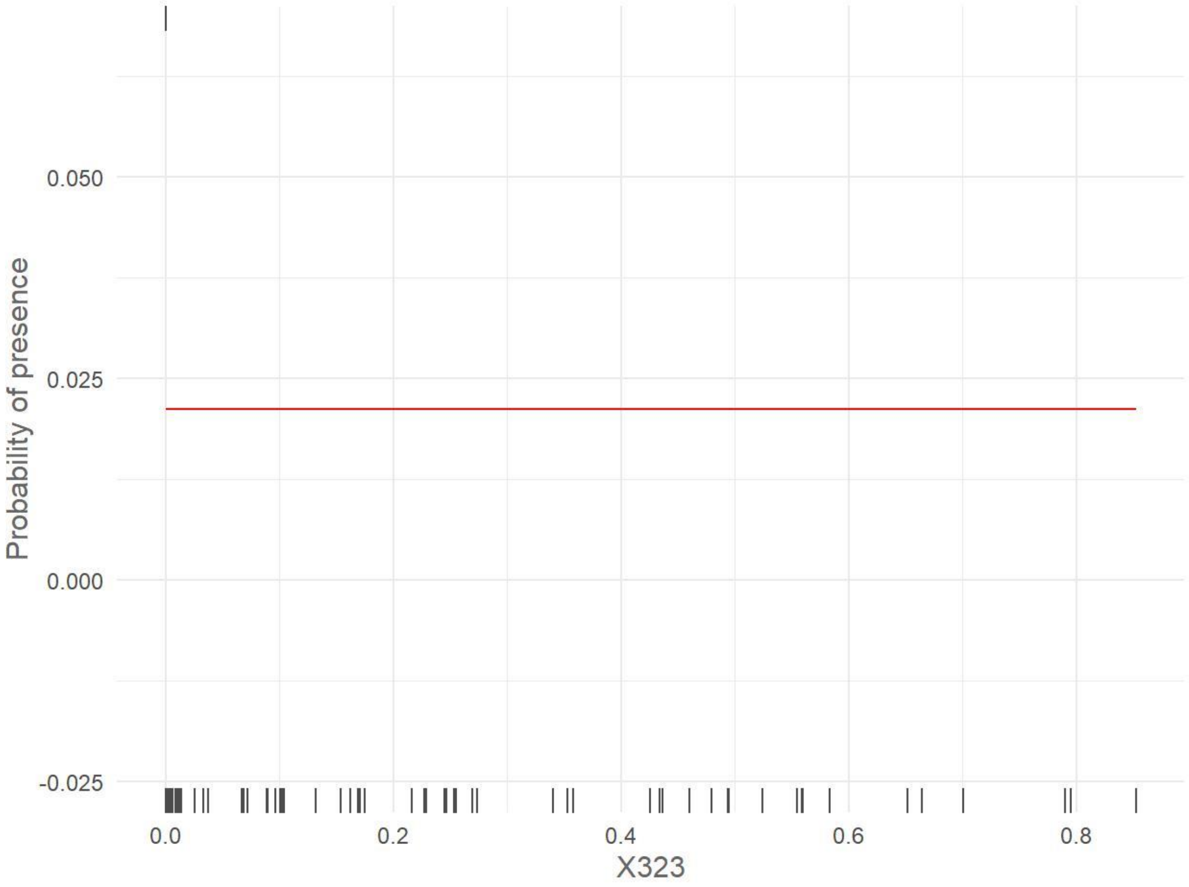
0.050
0.025
0.000
-0.025
Probability of presence
0.0
0.2
0.4
0.6
0.8
X323

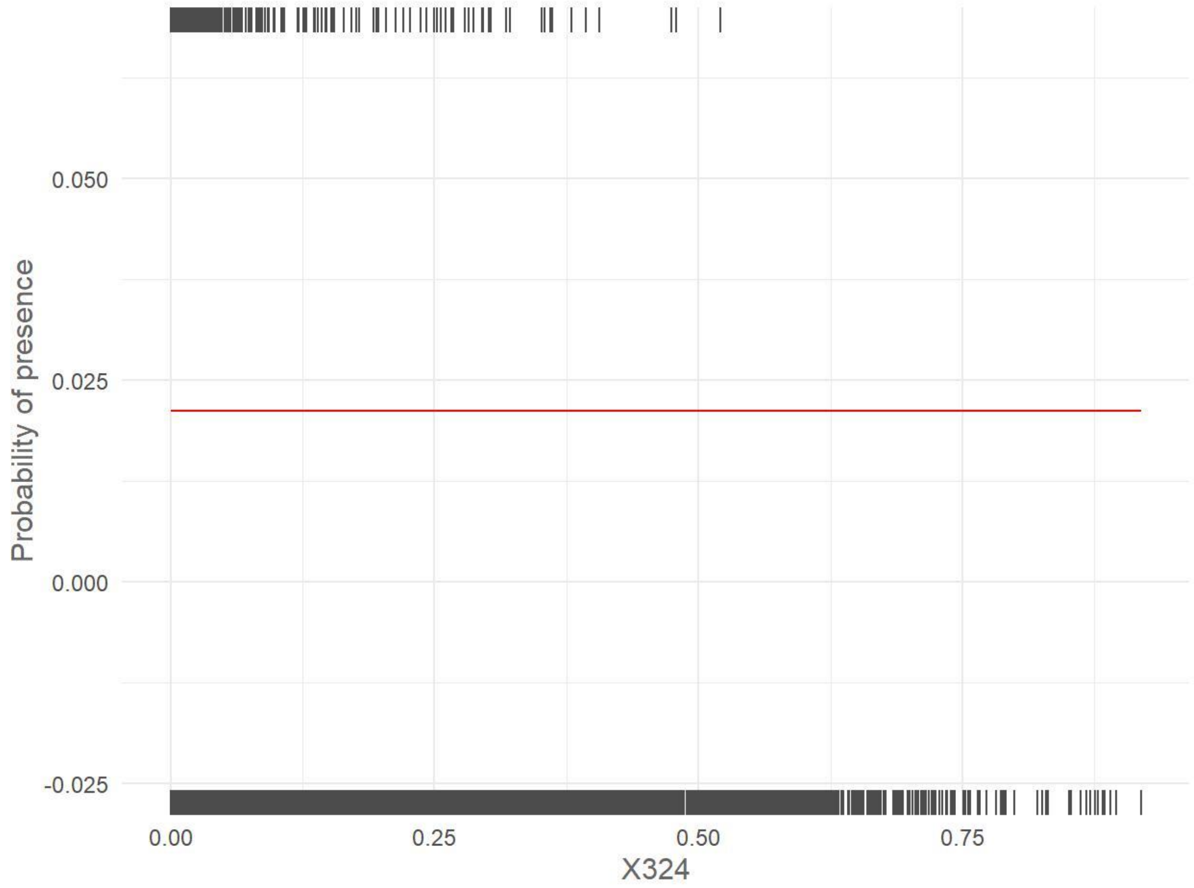

0.050
0.025
0.000
-0.025
Probability of presence
0.00
0.25
0.50
0.75
X324

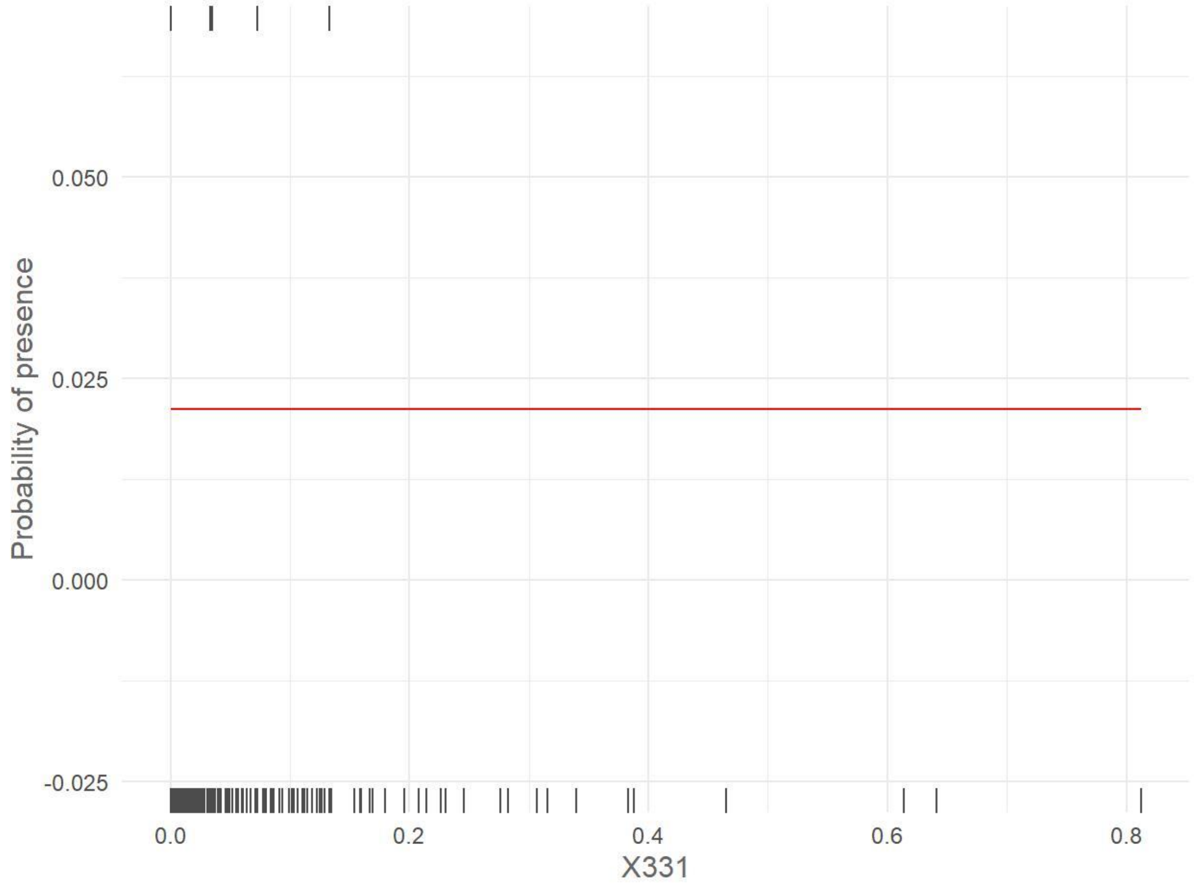

0.050
0.025
0.000
-0.025
Probability of presence
0.0
0.2
0.4
0.6
0.8
X331

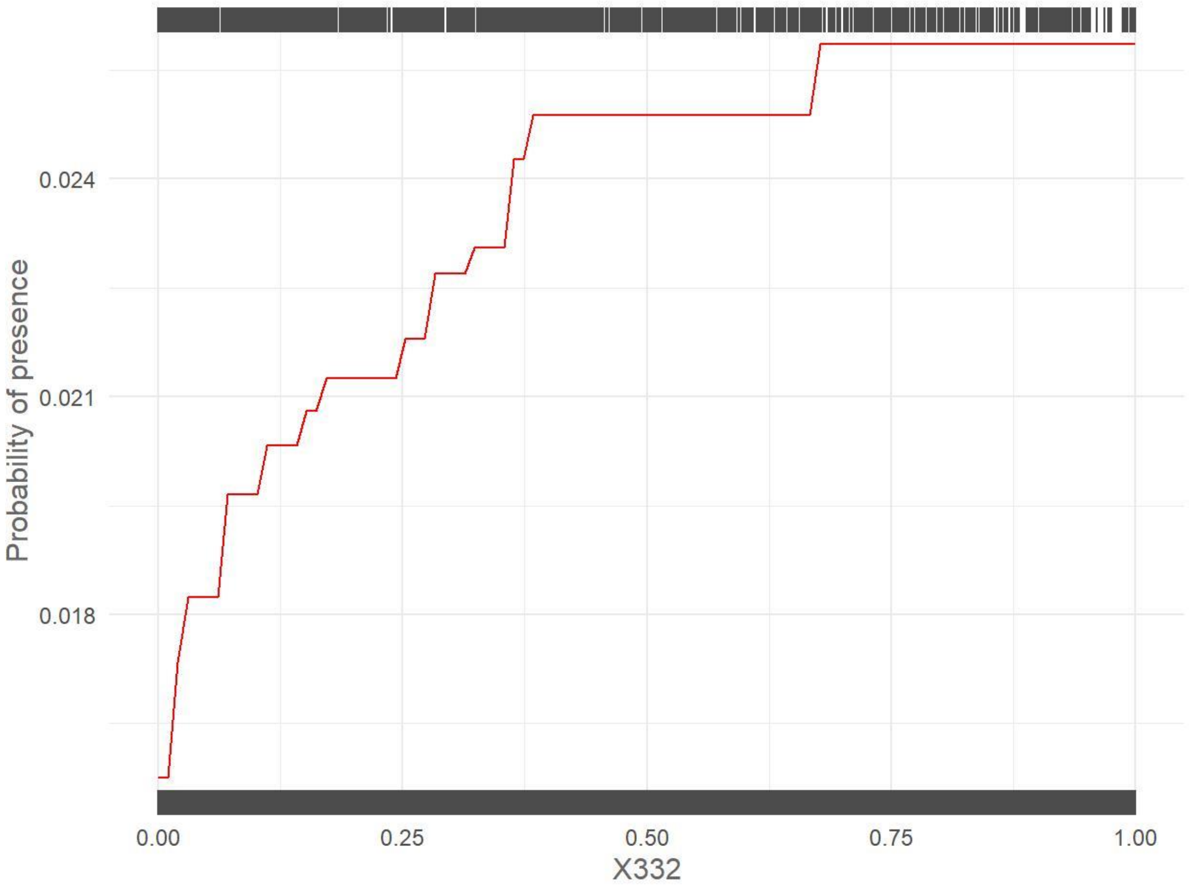

0.024
0.021
0.018
Probability of presence
0.00
0.25
0.50
0.75
1.00
X332

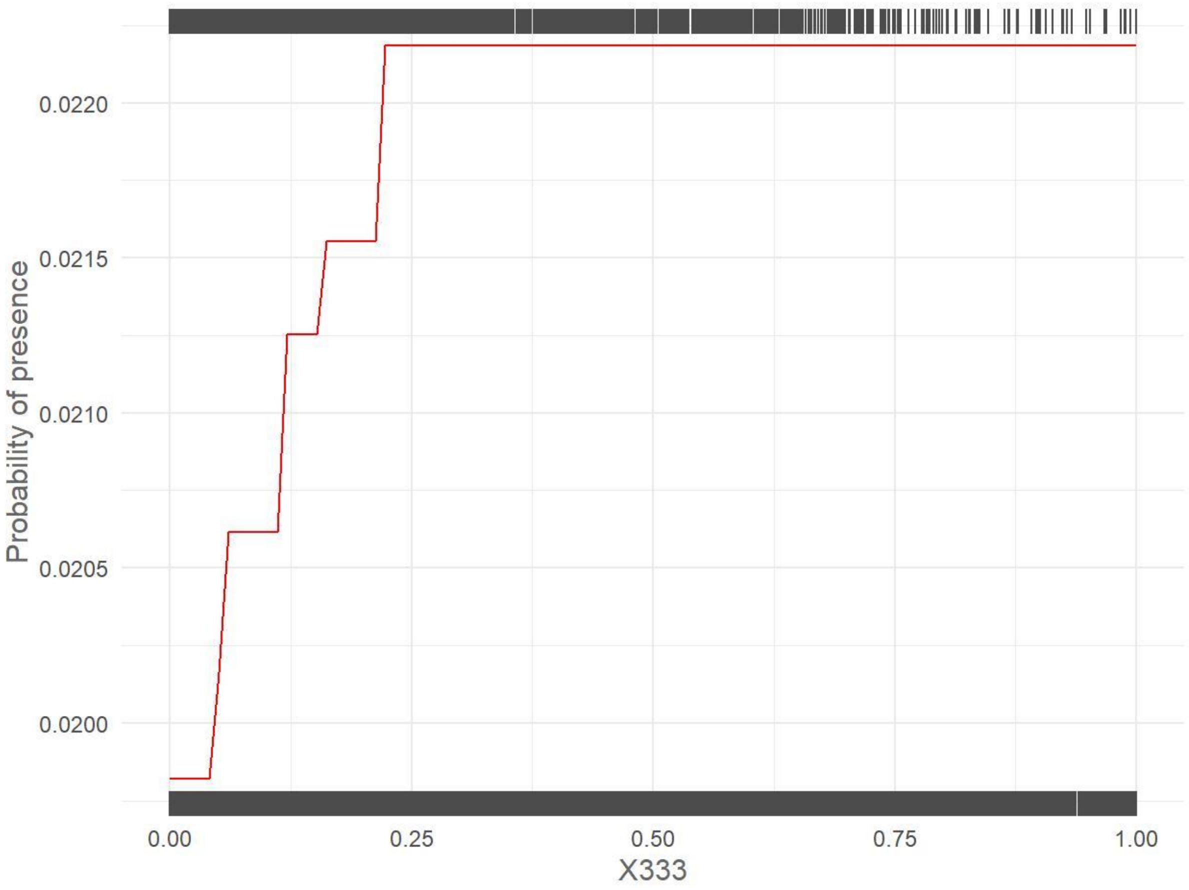

0.0220
0.0215
0.0210
0.0205
0.0200
Probability of presence
0.00
0.25
0.50
0.75
1.00
X333

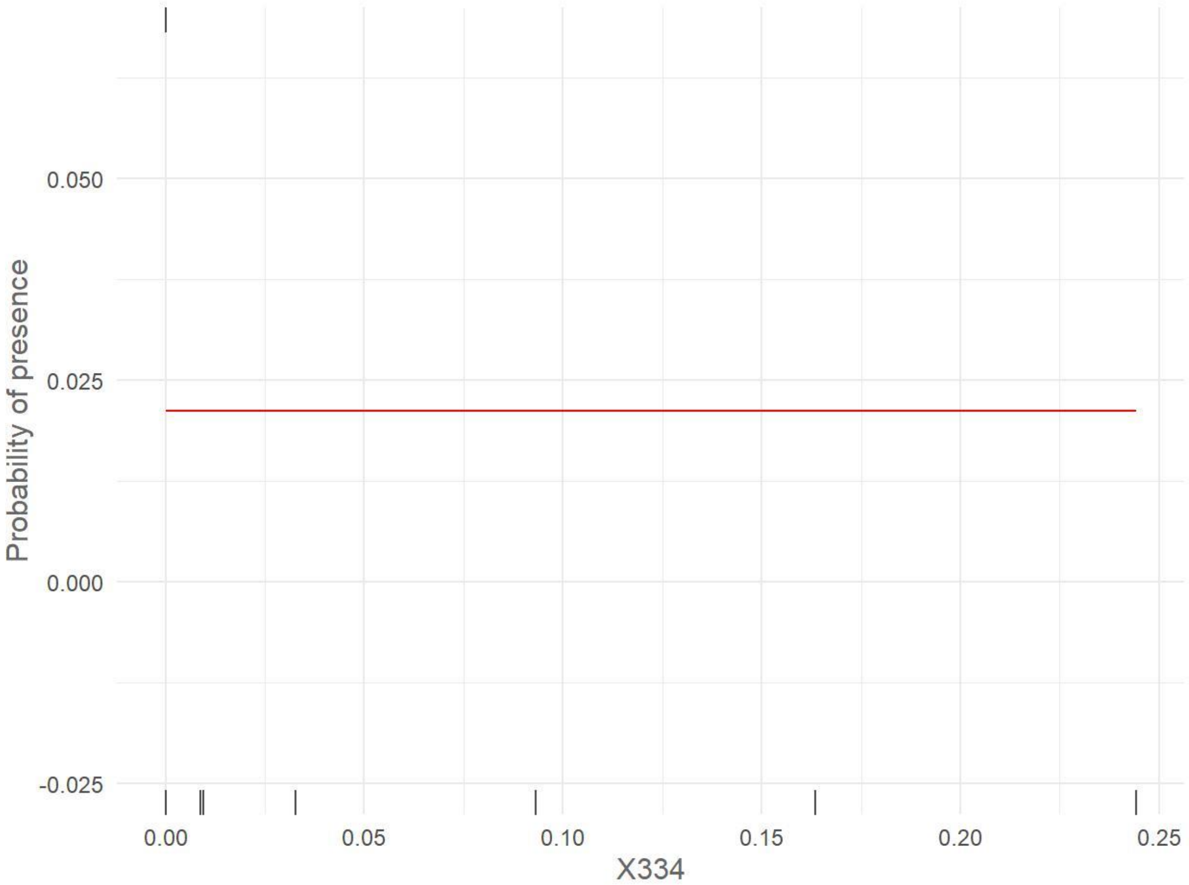
0.050
0.025
0.000
-0.025
Probability of presence
0.00
0.05
0.10
0.15
0.20
0.25
X334

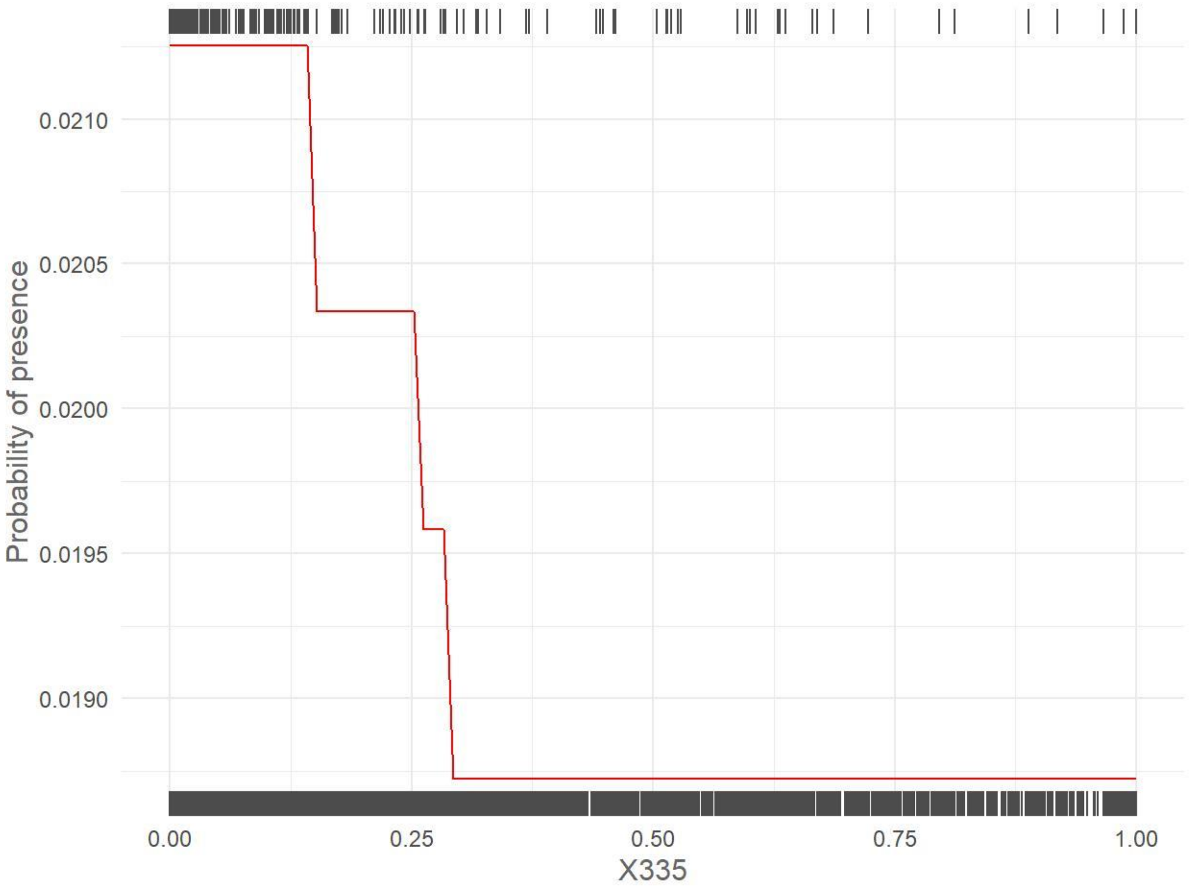
0.0210
0.0205
0.0200
0.0195
0.0190
Probability of presence
0.00
0.25
0.50
0.75
1.00
X335

Figure S13. Species-habitat relationships according to the Random Forest model for rock ptarmigan.

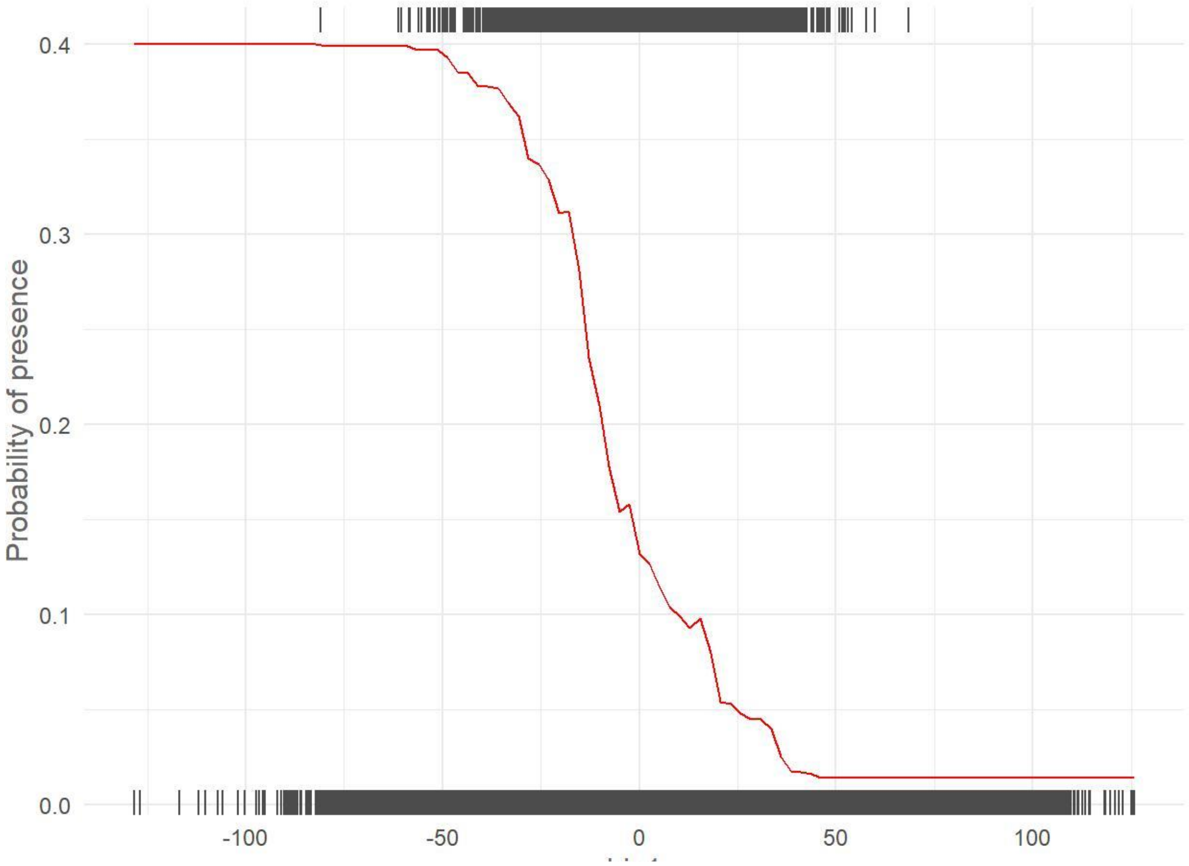
Probability of presence
0.4
0.3
0.2
0.1
0.0
-100
-50
0
50
100

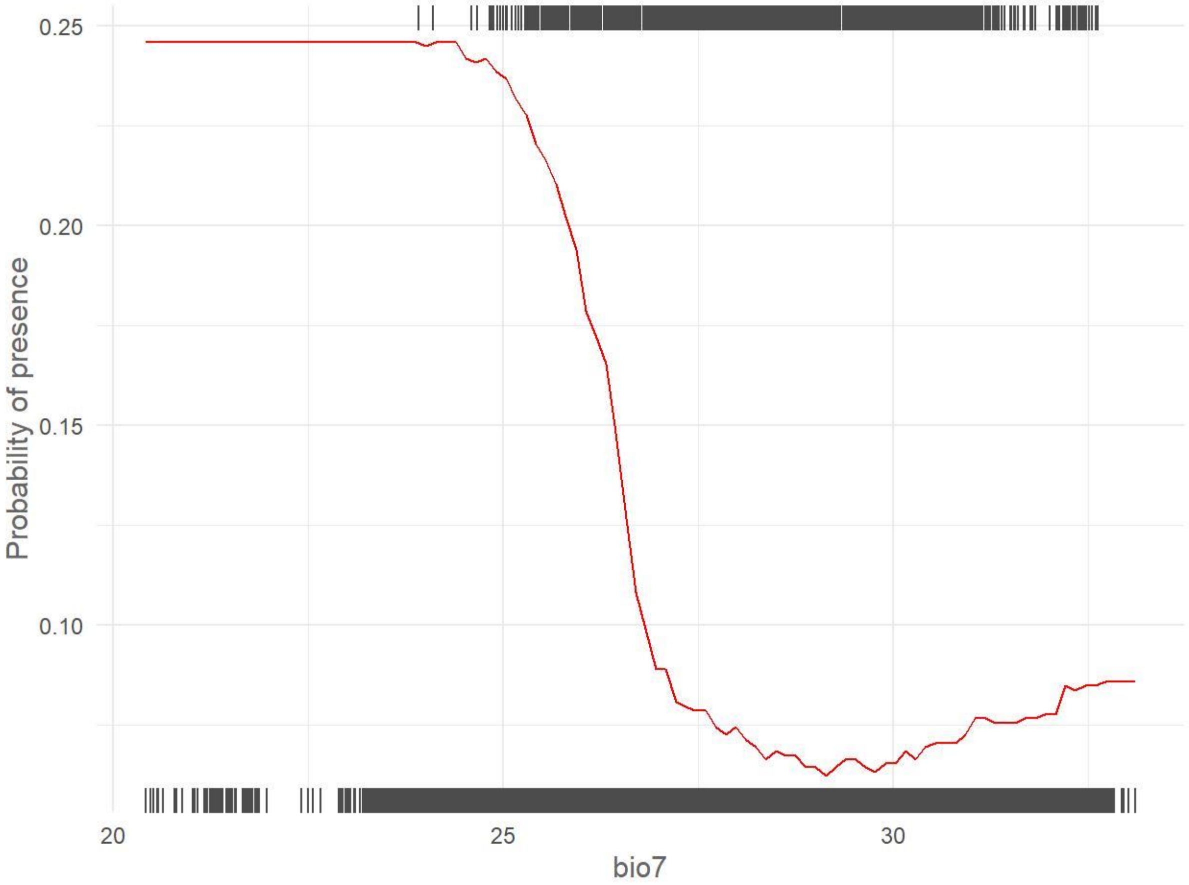
Probability of presence
0.25
0.20
0.15
0.10
20
25
30
bio7

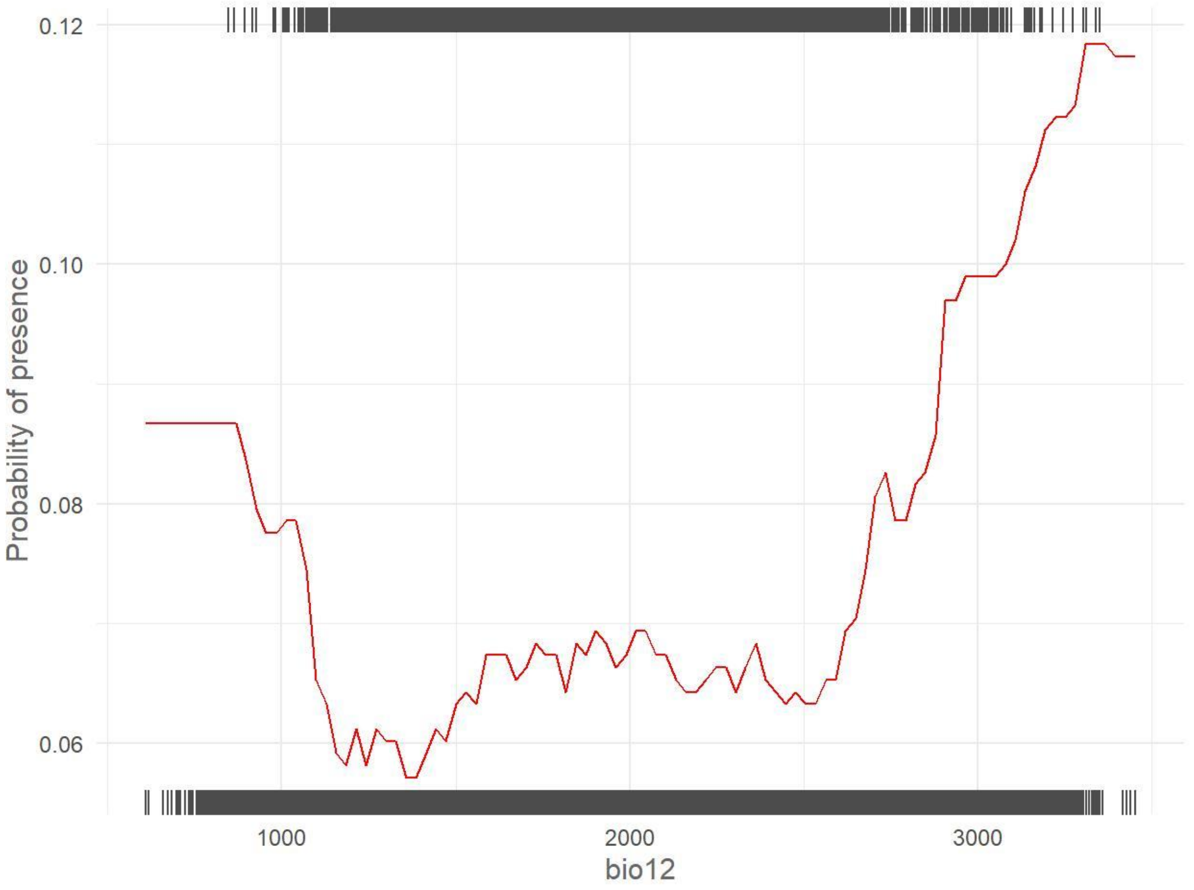

0.12
0.10
0.08
0.06
Probability of presence
1000
2000
3000
bio12

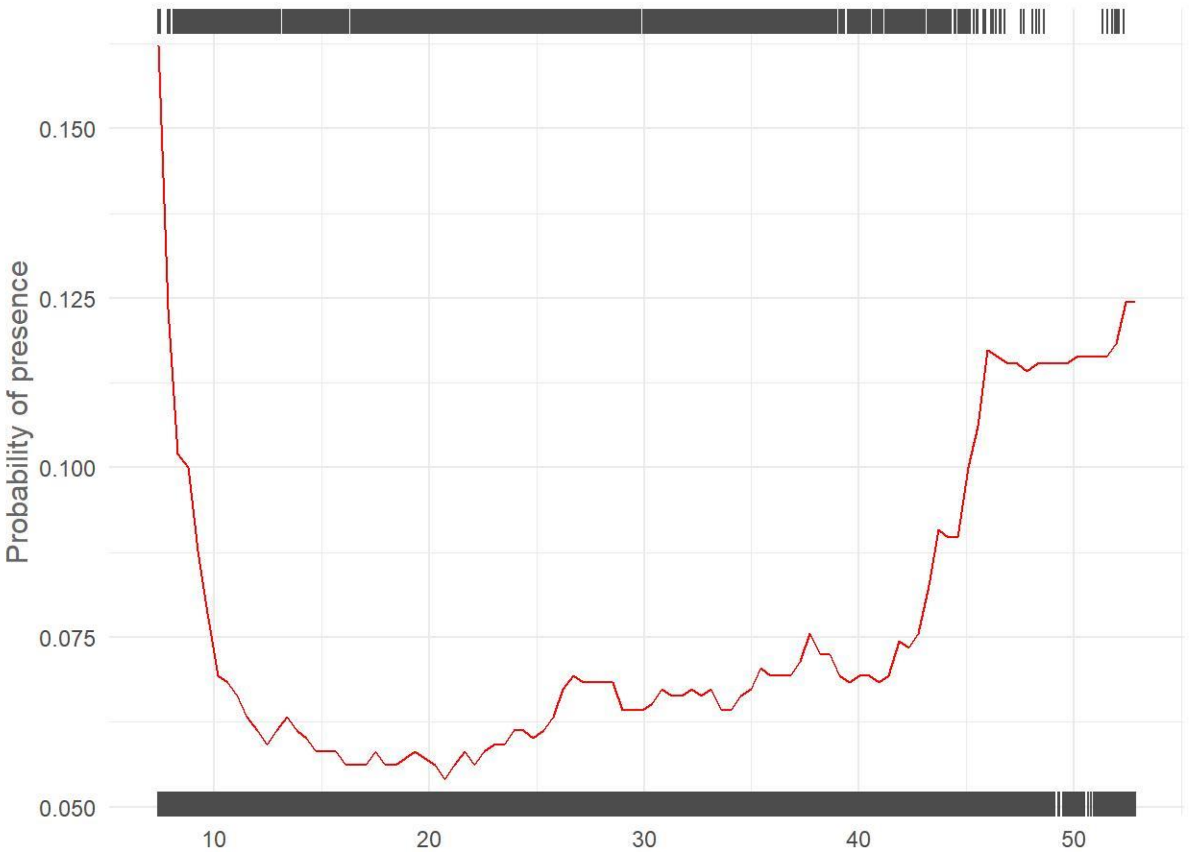

0.150
0.125
0.100
0.075
0.050
Probability of presence
10
20
30
40
50
bio15

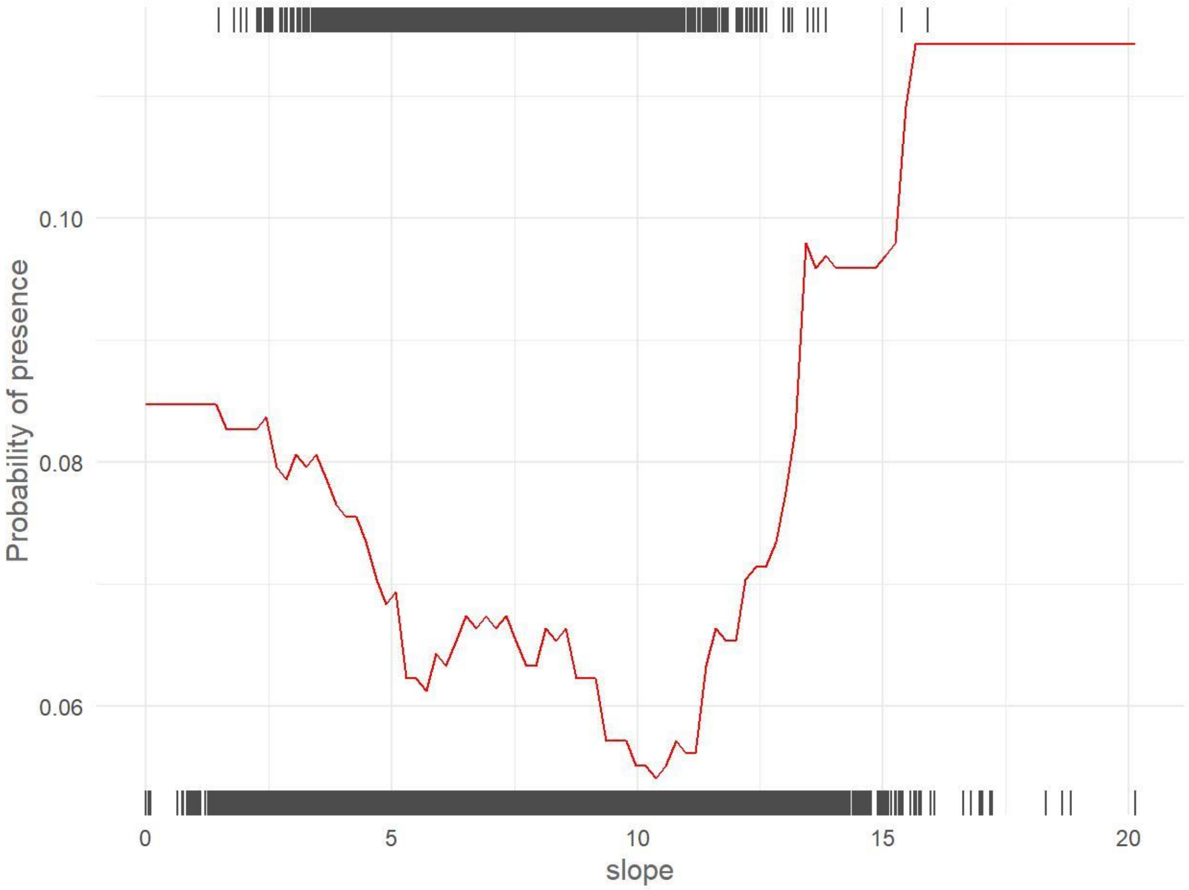

Probability of presence
0.10
0.08
0.06
0
5
10
15
20
slope

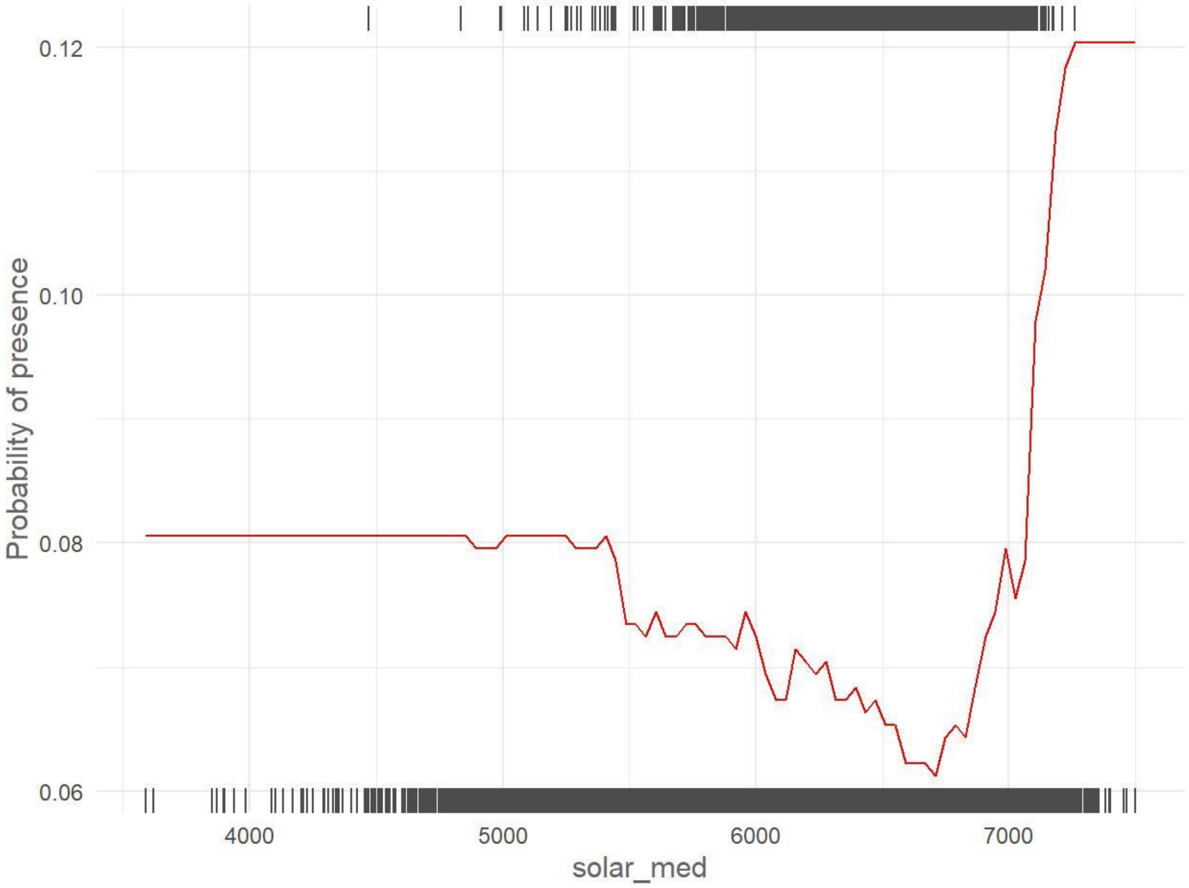

Probability of presence
0.12
0.10
0.08
0.06
4000
5000
6000
7000
solar_med

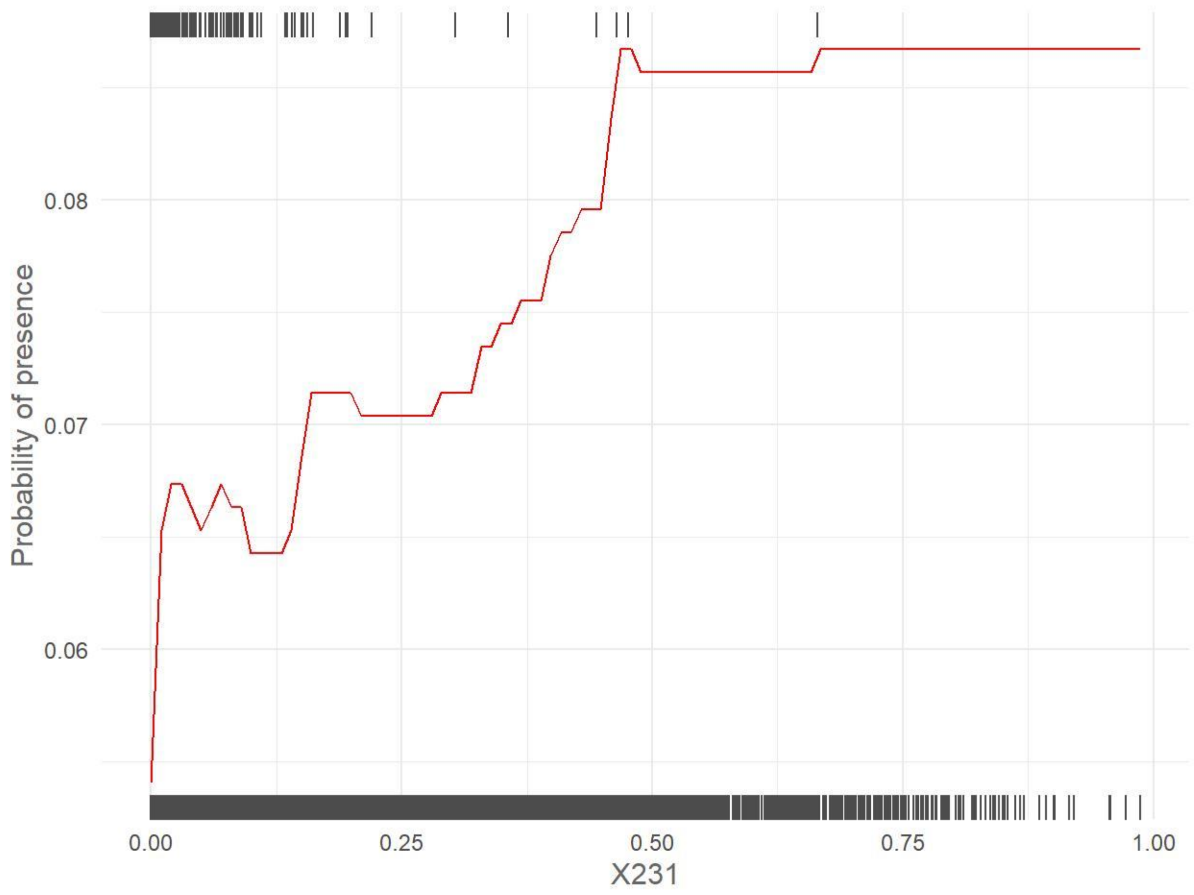

Probability of presence
0.08
0.07
0.06
0.00
0.25
0.50
0.75
1.00
X231

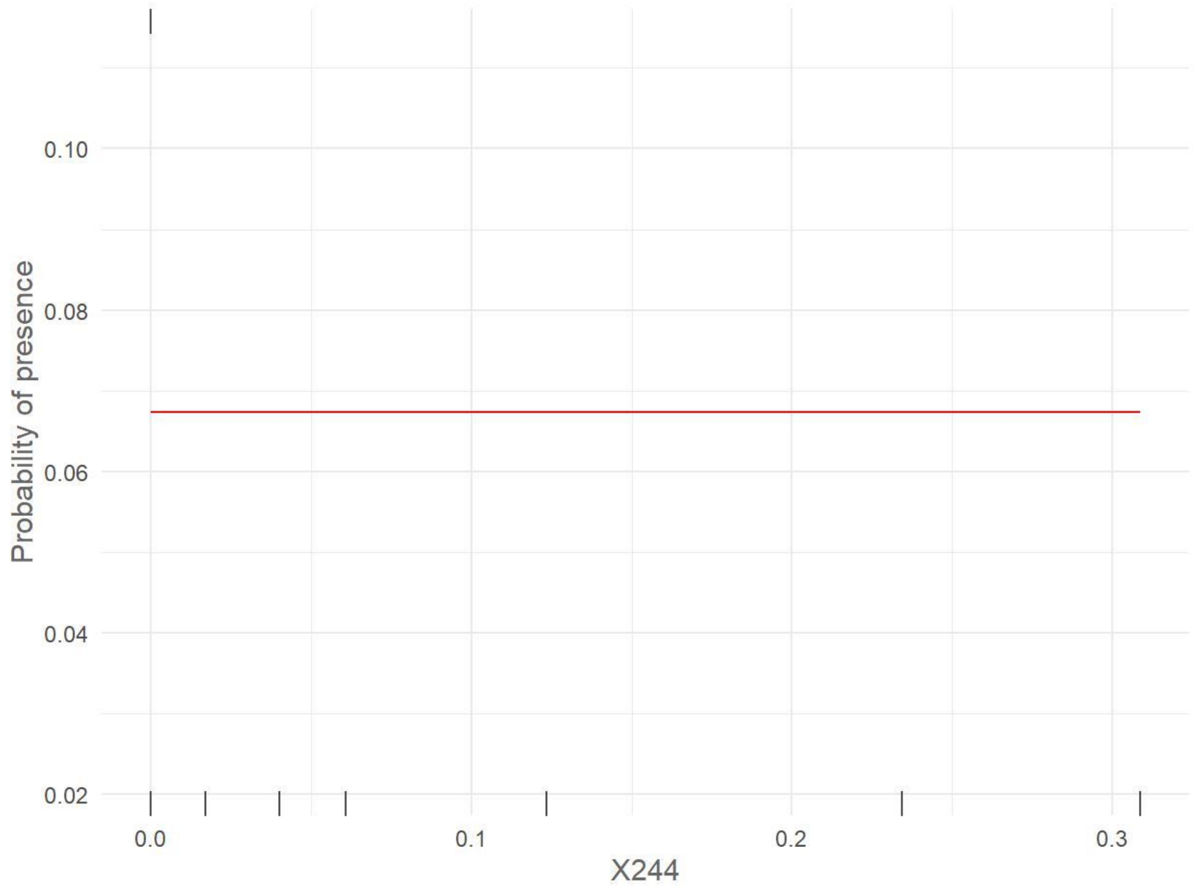

Probability of presence
0.10
0.08
0.06
0.04
0.02
0.0
0.1
0.2
0.3
X244

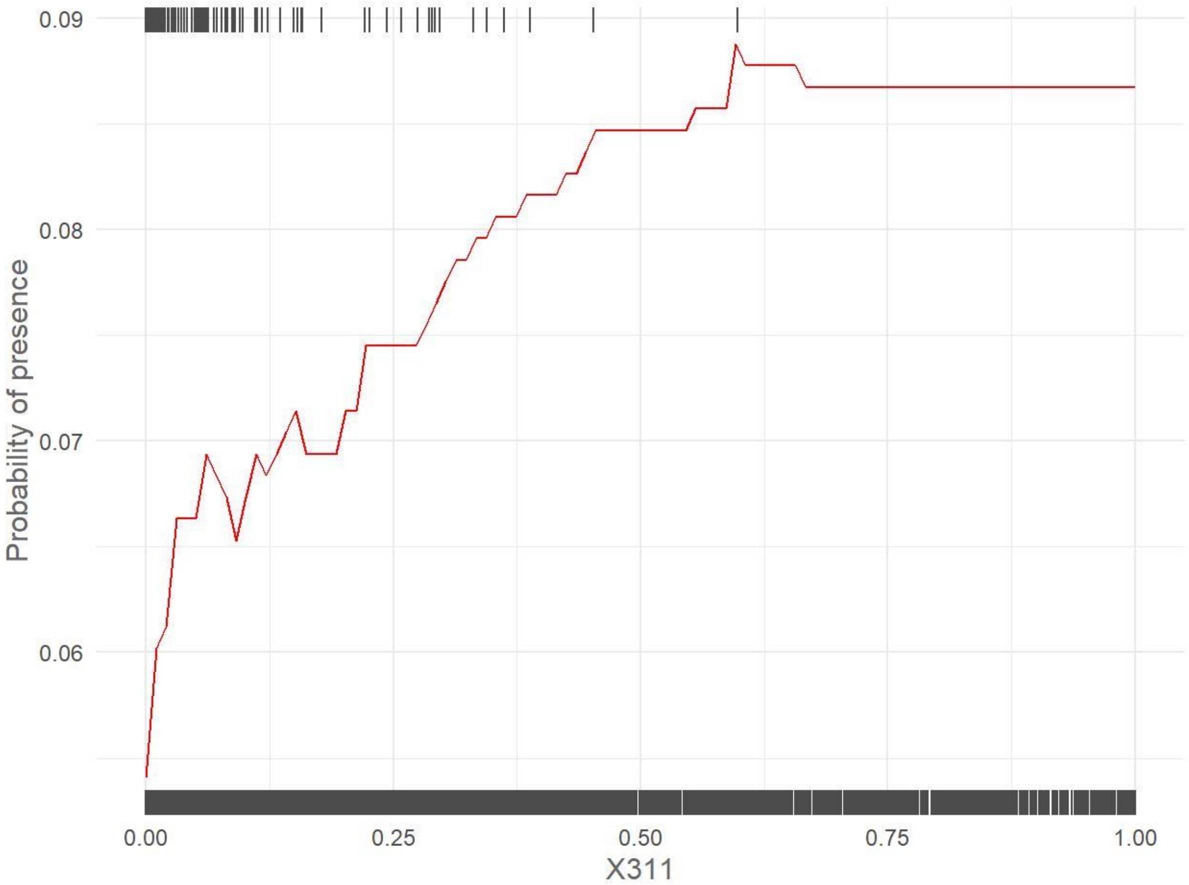
0.09
0.08
0.07
0.06
Probability of presence
0.00
0.25
0.50
0.75
1.00
X311

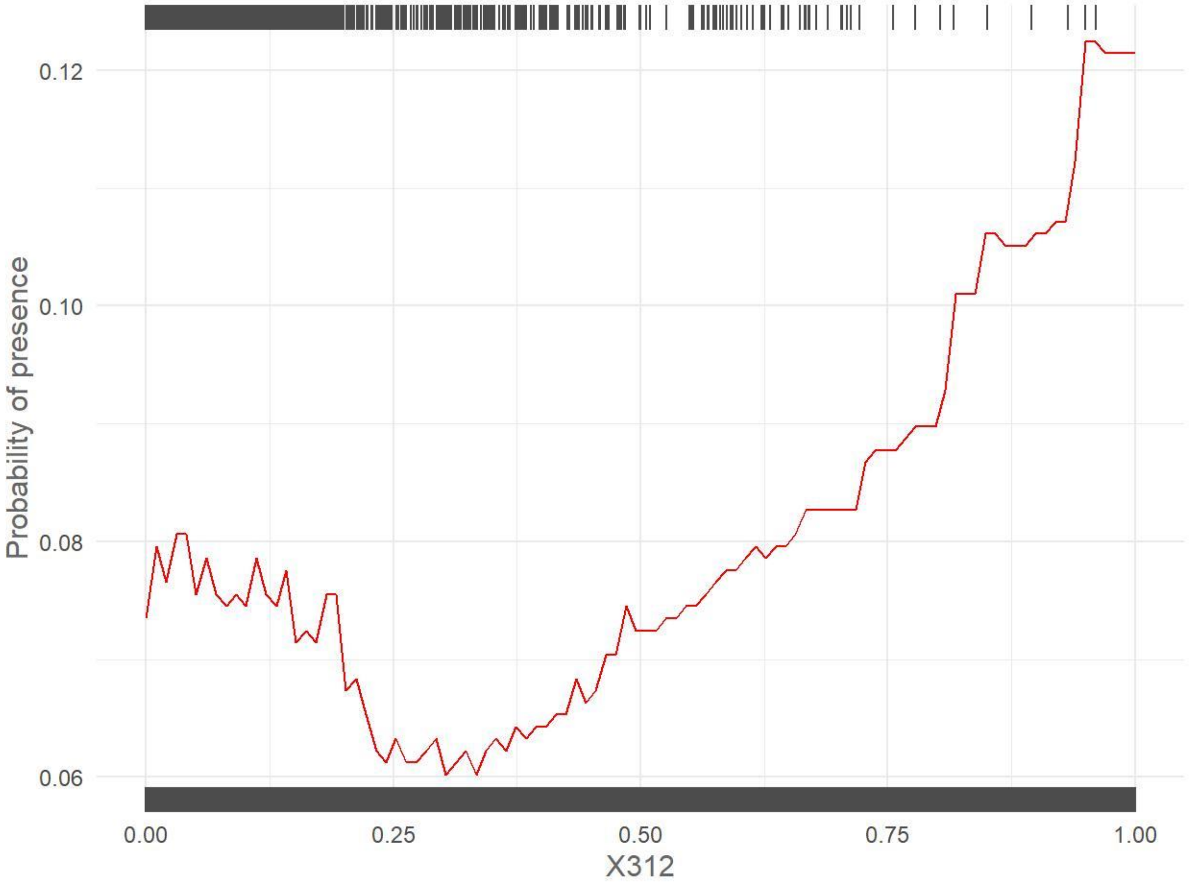
0.12
0.10
0.08
0.06
Probability of presence
0.00
0.25
0.50
0.75
1.00
X312

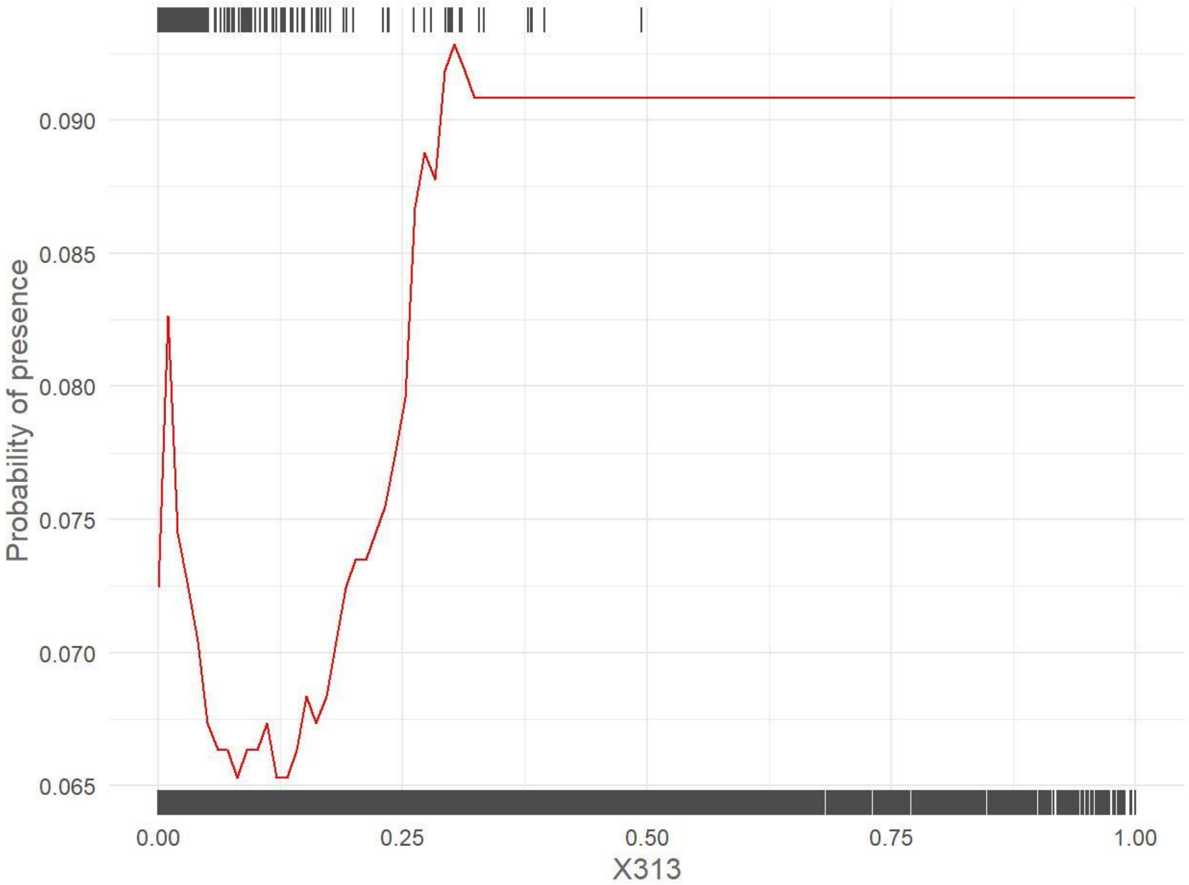
0.090
0.085
0.080
0.075
0.070
0.065
Probability of presence
0.00
0.25
0.50
0.75
1.00
X313

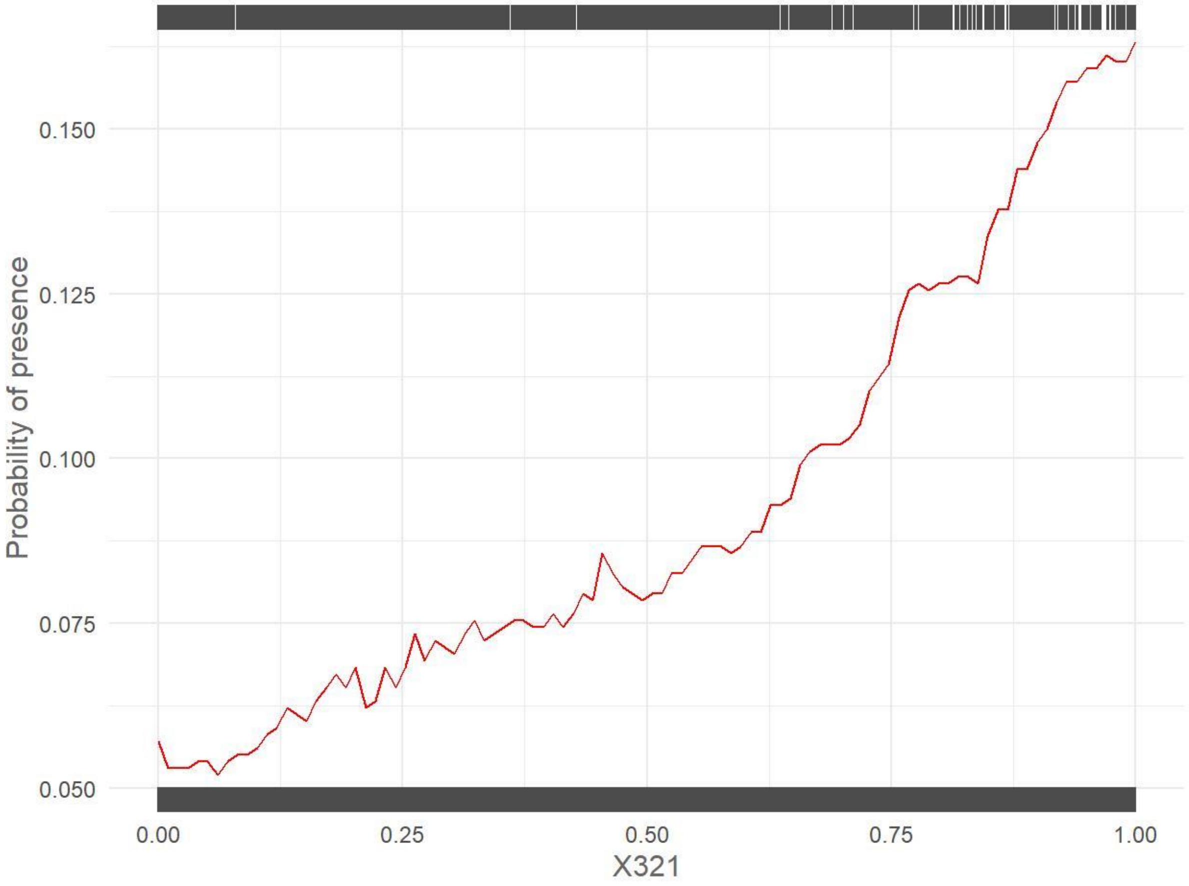
0.150
0.125
0.100
0.075
0.050
Probability of presence
0.00
0.25
0.50
0.75
1.00
X321

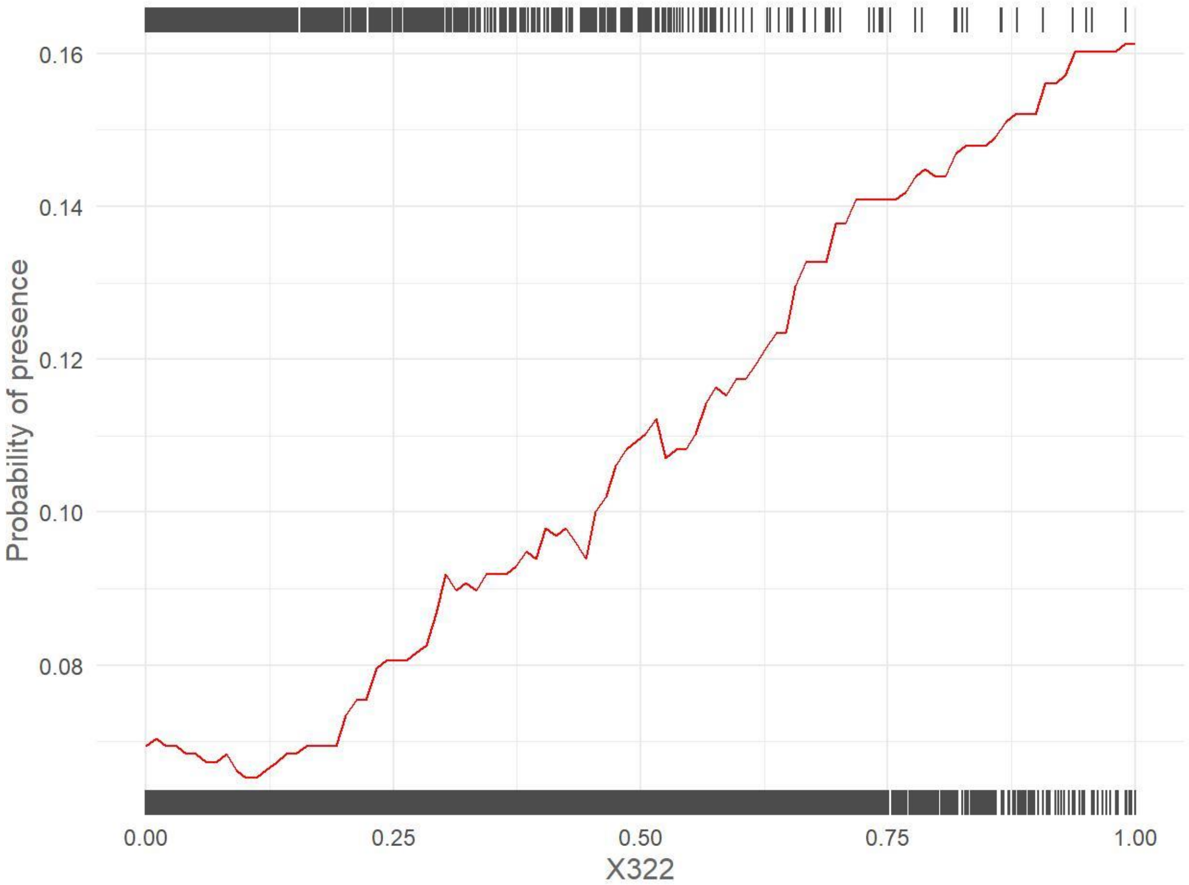

0.16
0.14
0.12
0.10
0.08
Probability of presence
0.00
0.25
0.50
0.75
1.00
X322

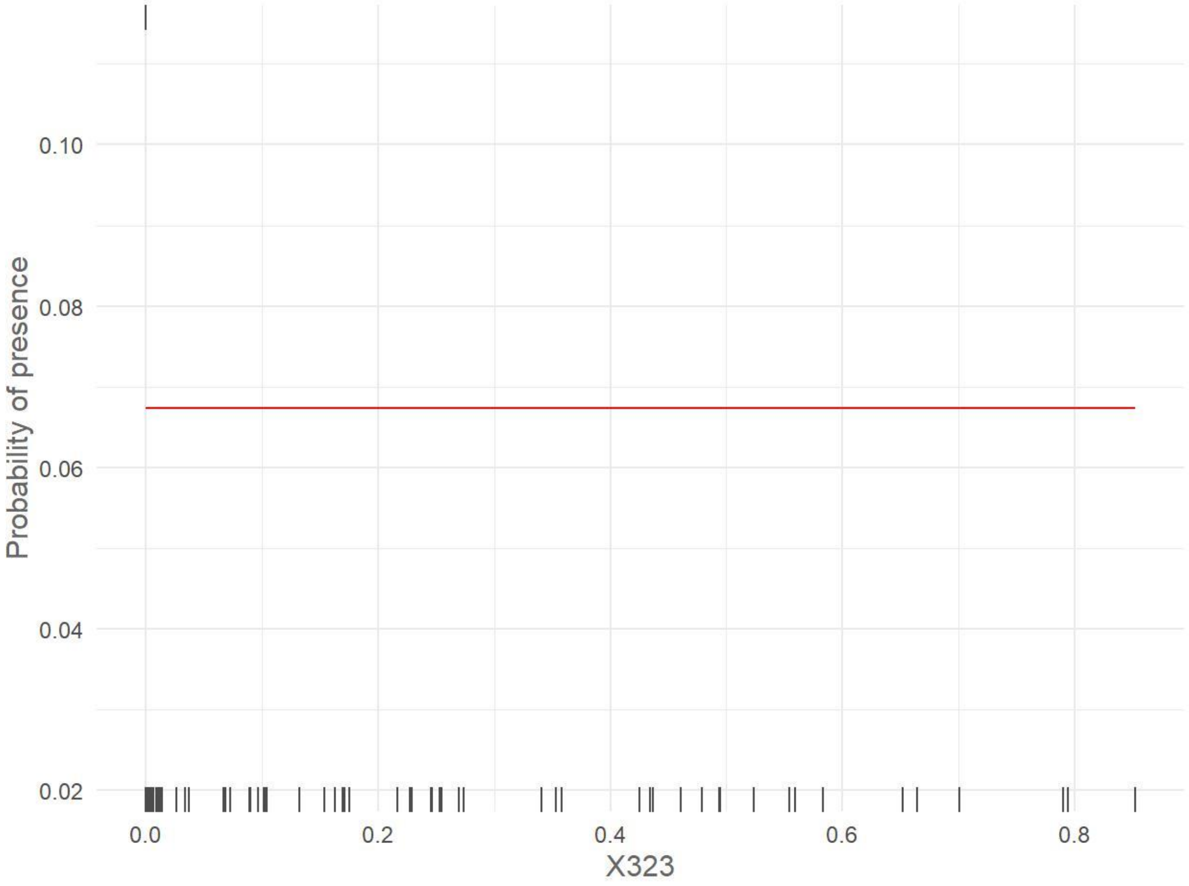

0.10
0.08
0.06
0.04
0.02
Probability of presence
0.0
0.2
0.4
0.6
0.8
X323

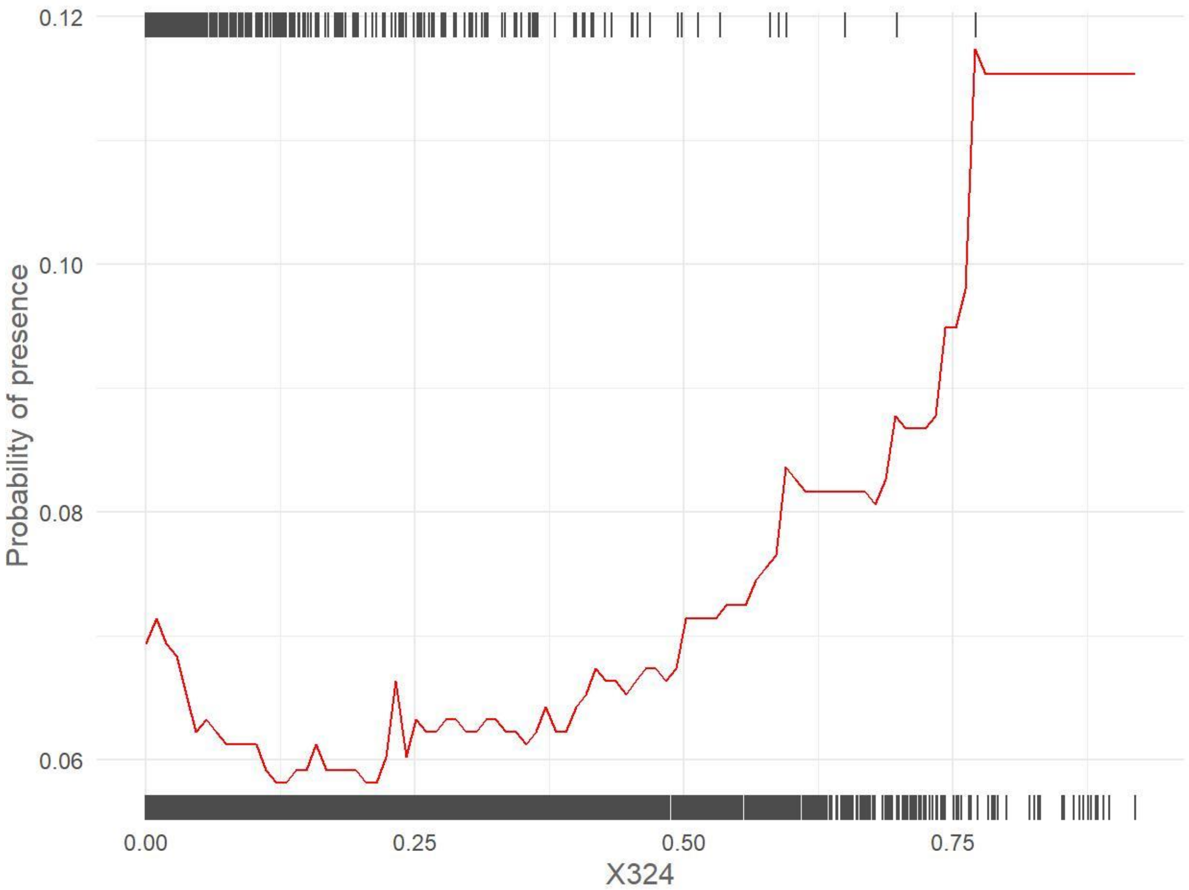
0.12
0.10
0.08
0.06
Probability of presence
0.00
0.25
0.50
0.75
X324

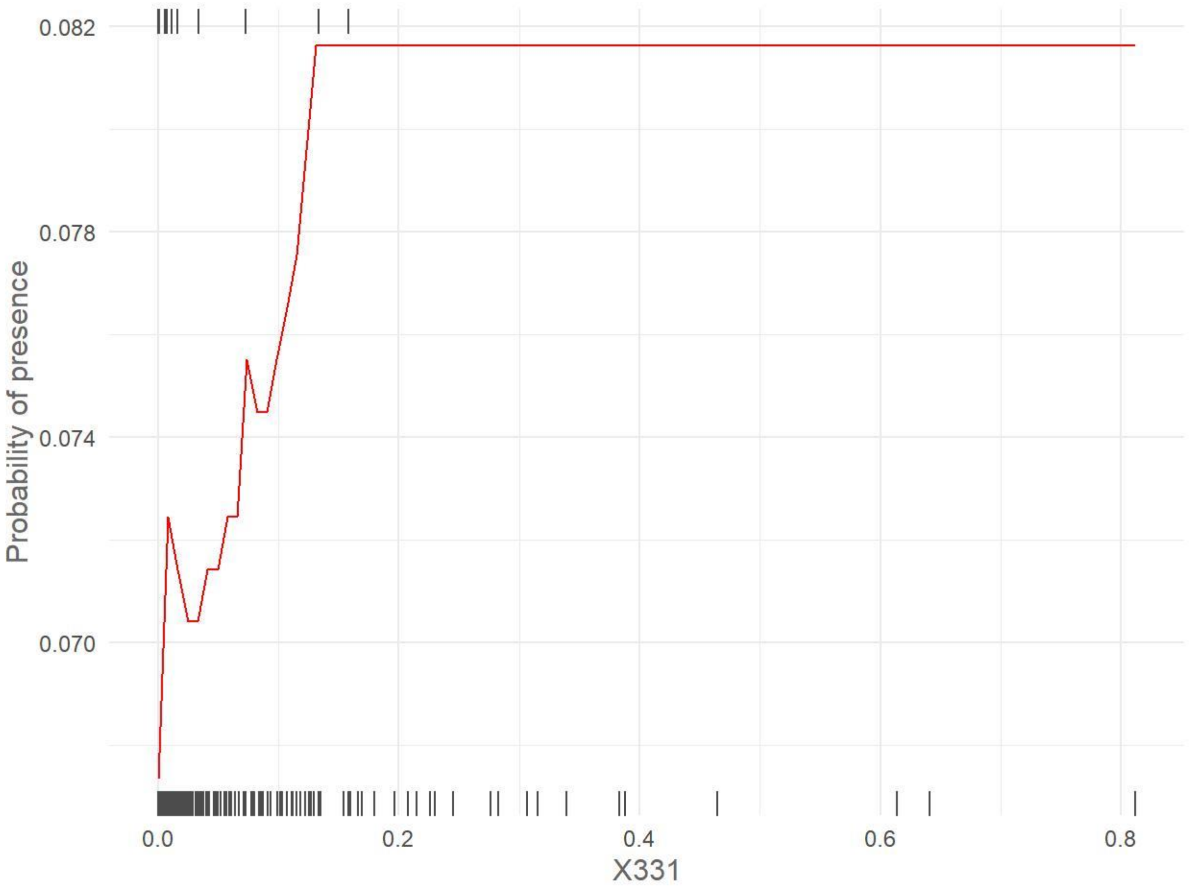
0.082
0.078
0.074
0.070
Probability of presence
0.0
0.2
0.4
0.6
0.8
X331

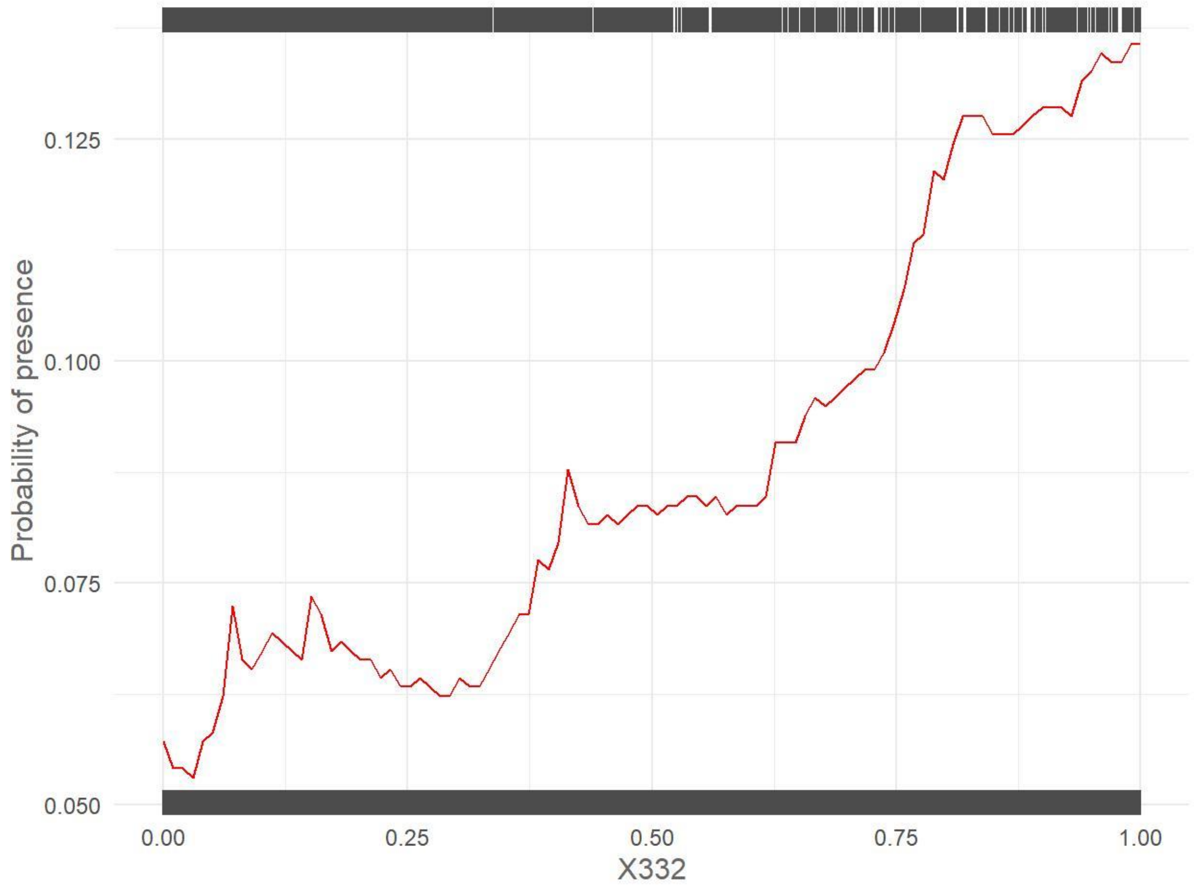
Probability of presence
0.125
0.100
0.075
0.050
0.00
0.25
0.50
0.75
1.00
X332

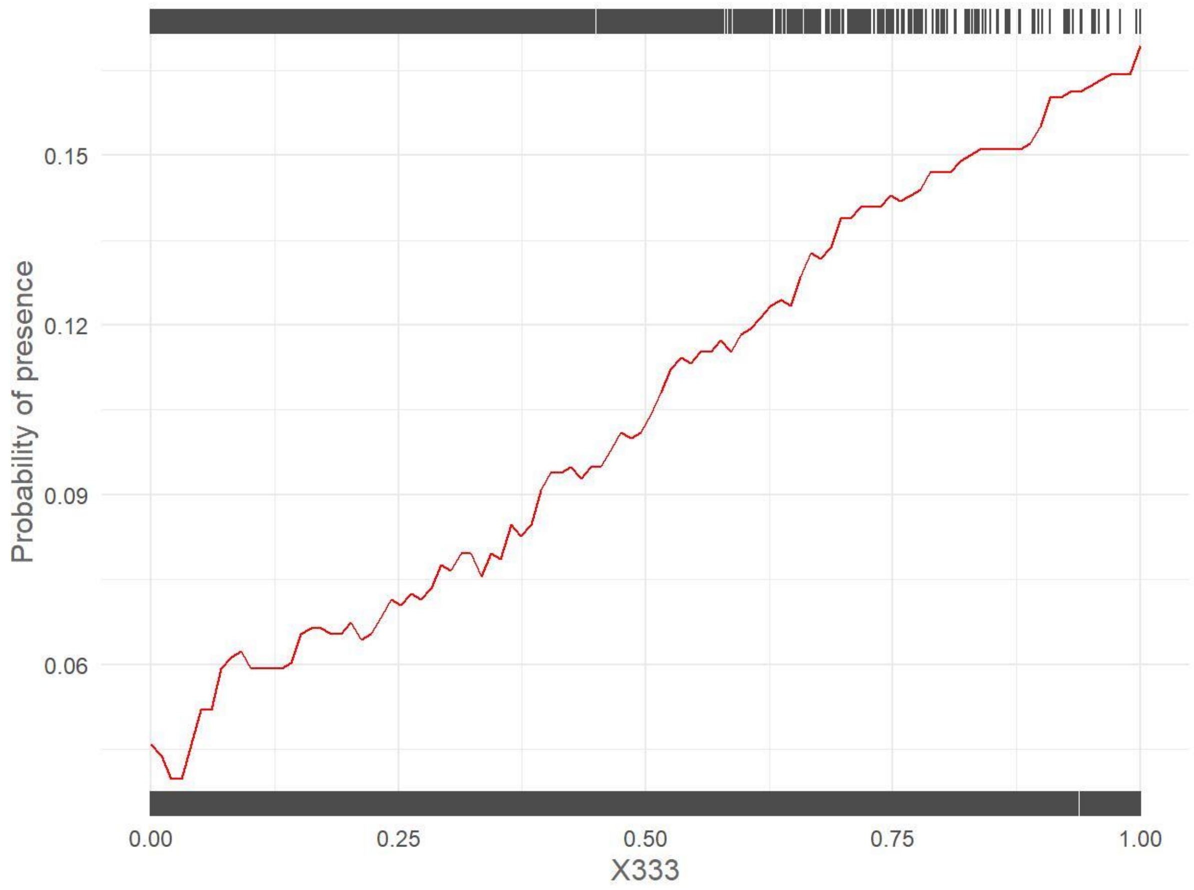
Probability of presence
0.15
0.12
0.09
0.06
0.00
0.25
0.50
0.75
1.00
X333

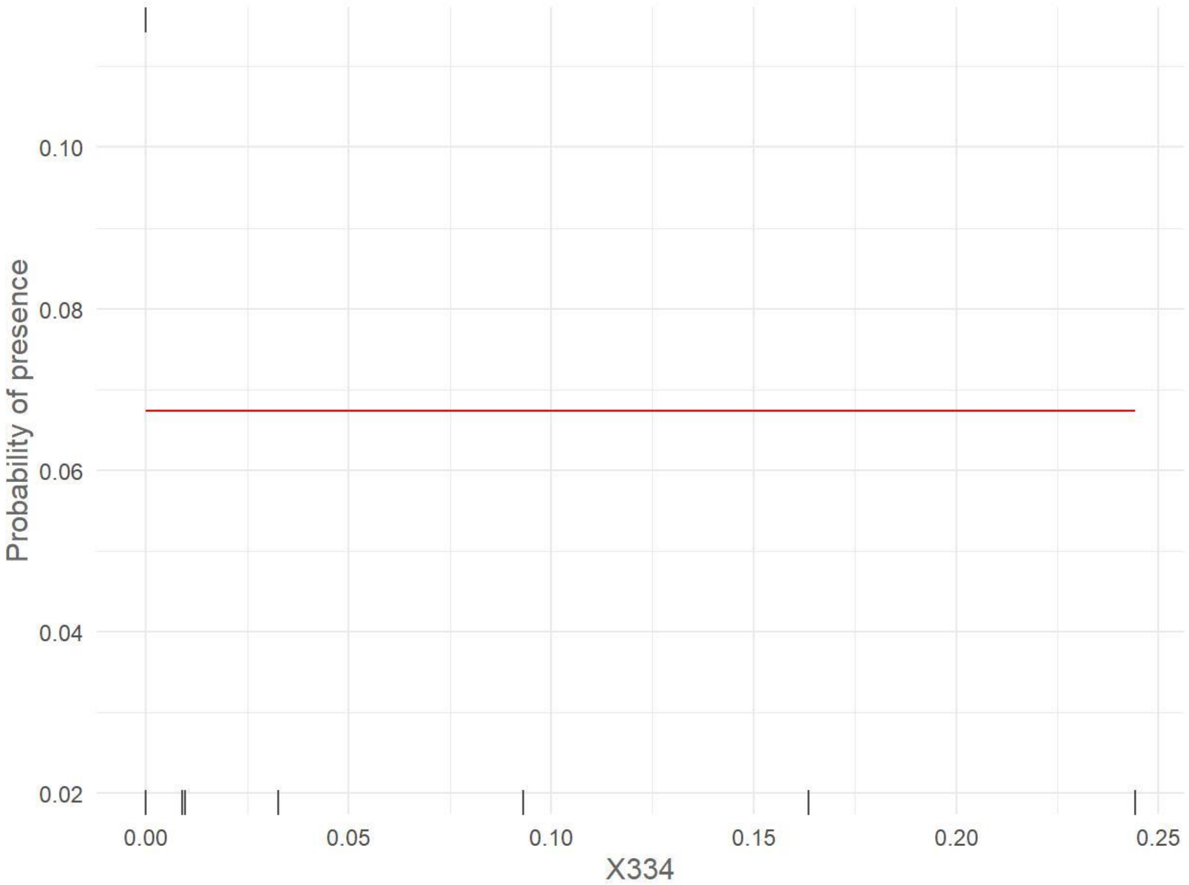
Probability of presence
0.10
0.08
0.06
0.04
0.02
0.00
0.05
0.10
0.15
0.20
0.25
X334

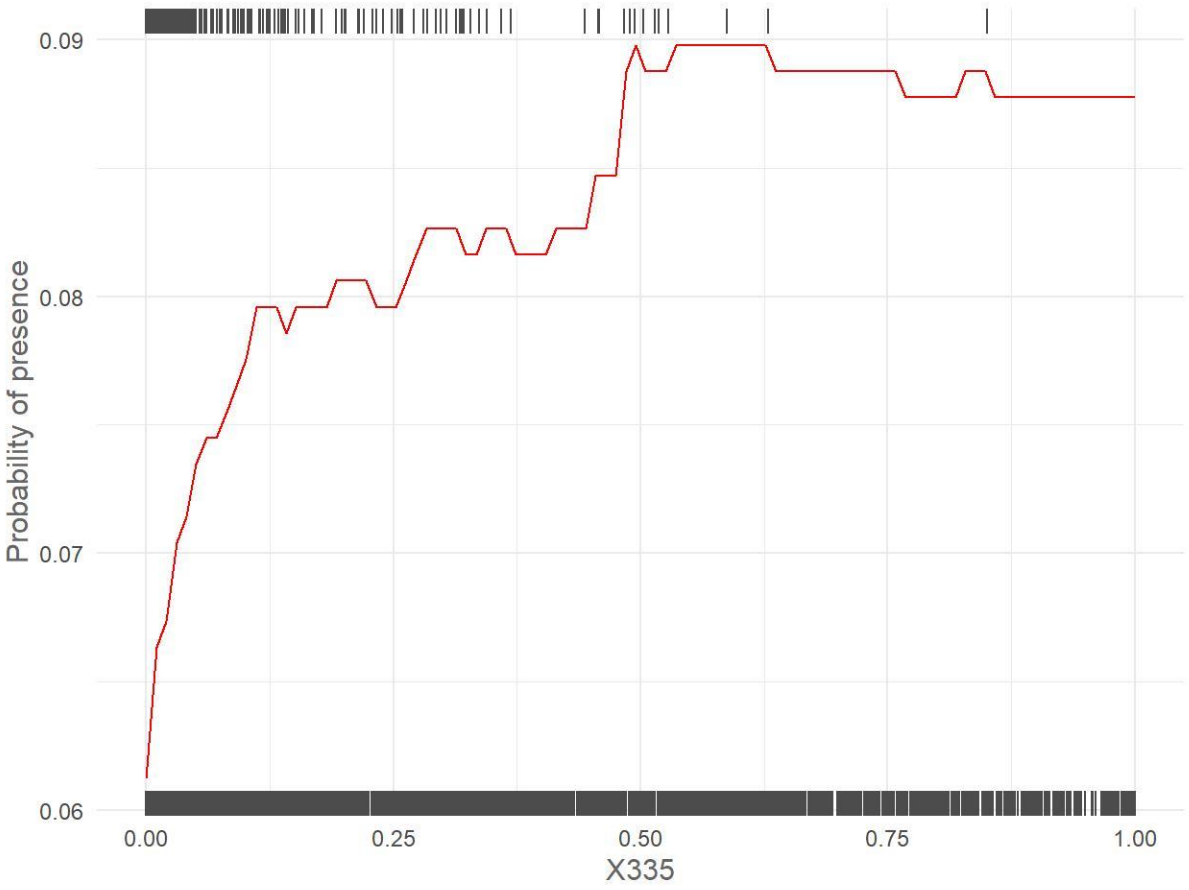
Probability of presence
0.09
0.08
0.07
0.06
0.00
0.25
0.50
0.75
1.00
X335

Figure S14. Species-habitat relationships according to the Random Forest model for water pipit.

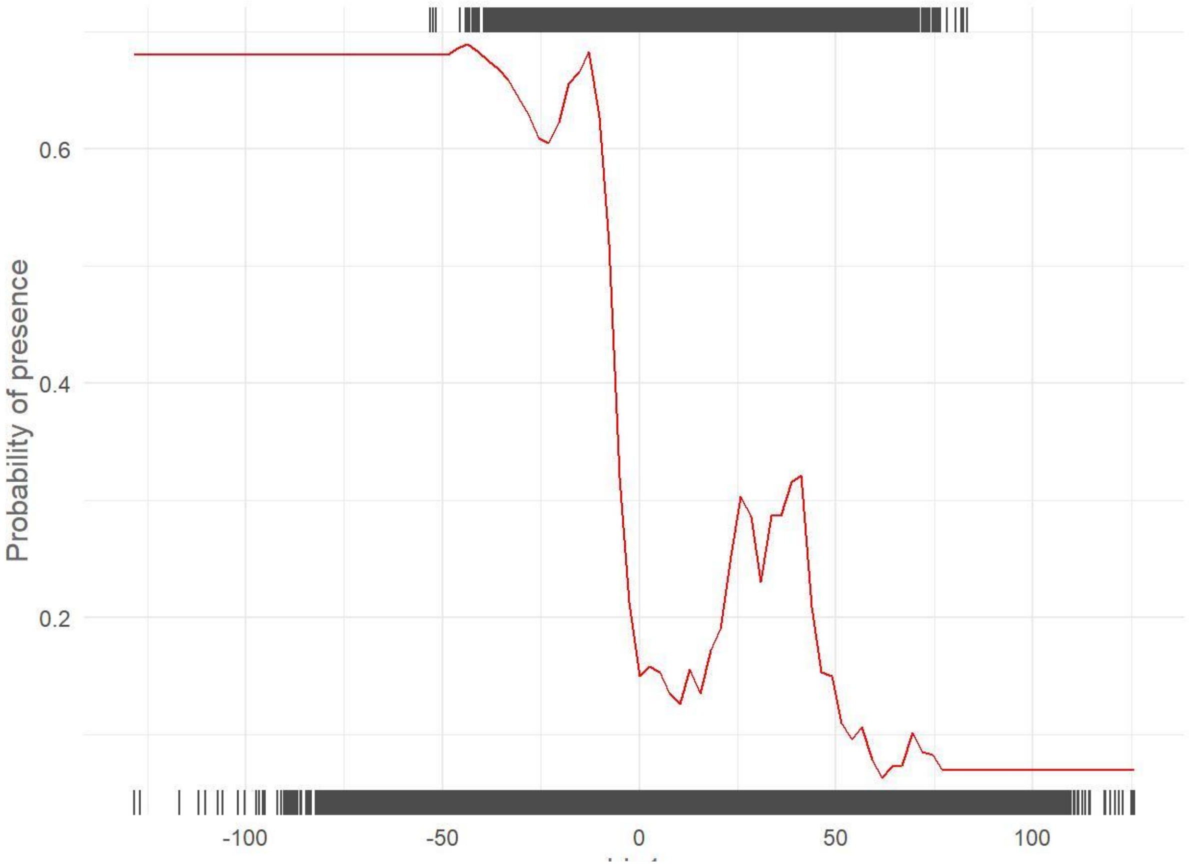
Probability of presence
0.6
0.4
0.2
-100
-50
0
50
100

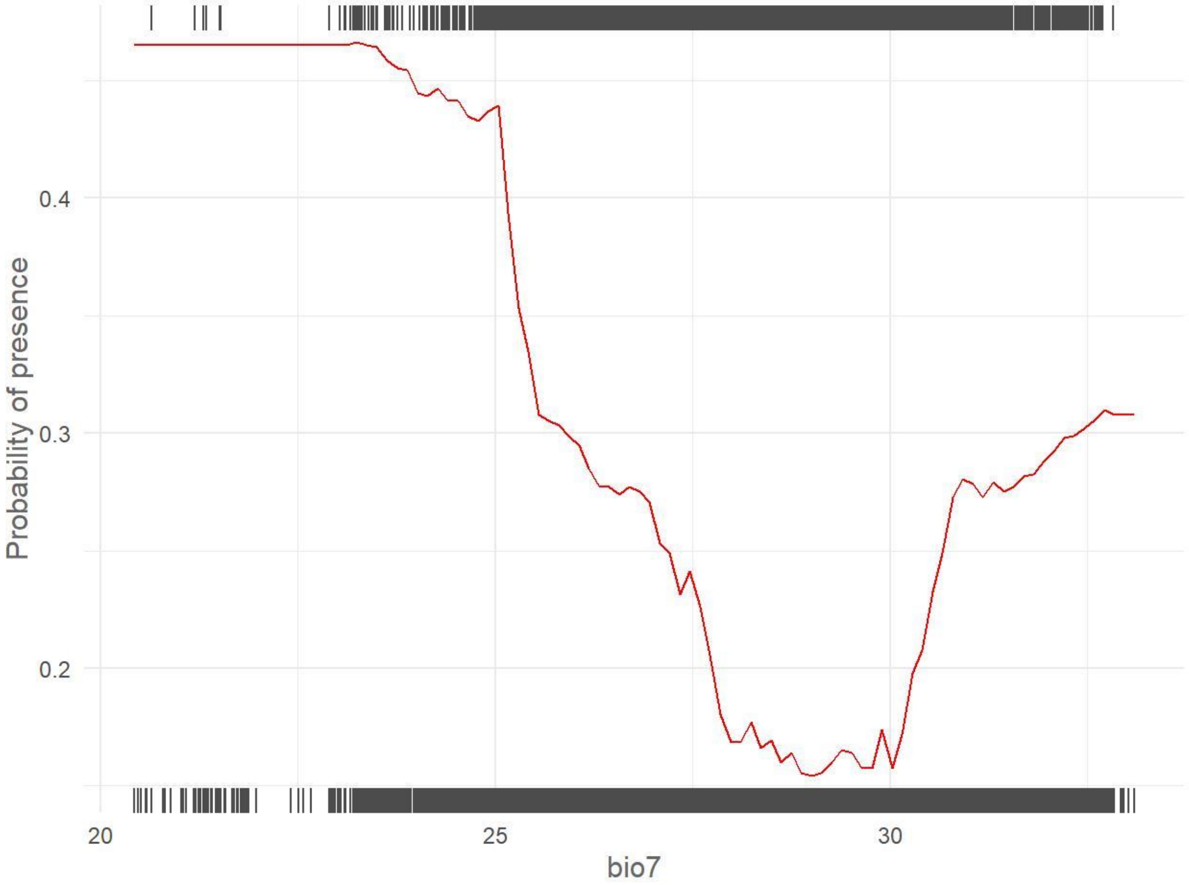
Probability of presence
0.4
0.3
0.2
20
25
30
bio7

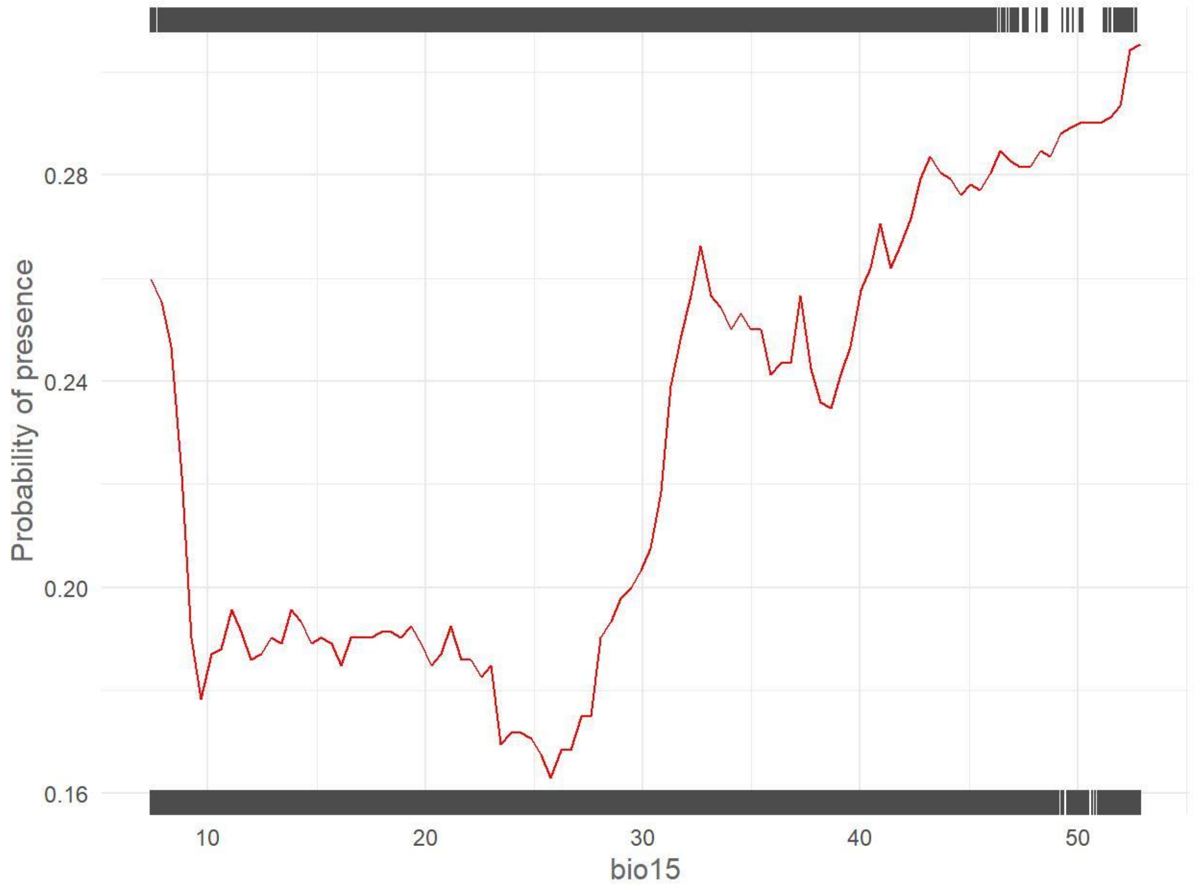
Probability of presence
0.16
0.20
0.24
0.28
10
20
30
40
50
bio15

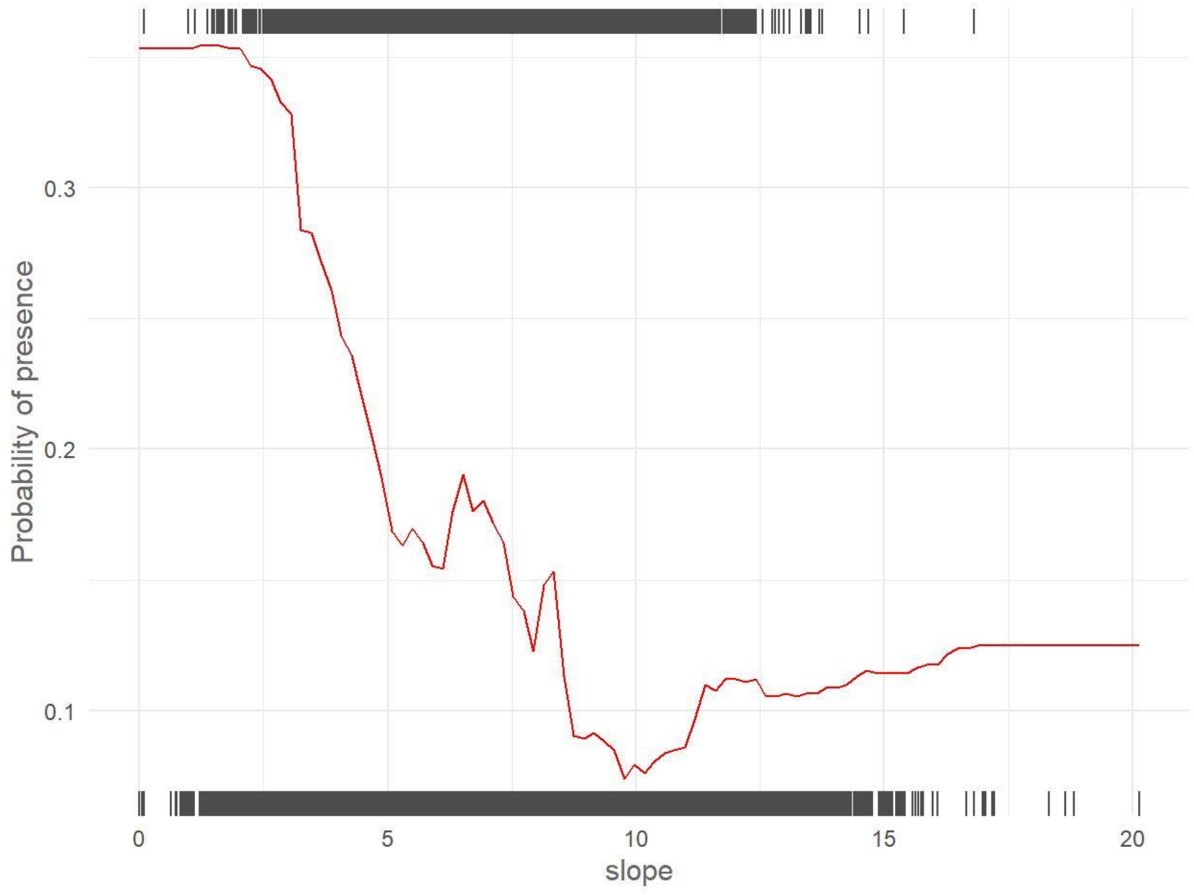
Probability of presence
0.1
0.2
0.3
0
5
10
15
20
slope

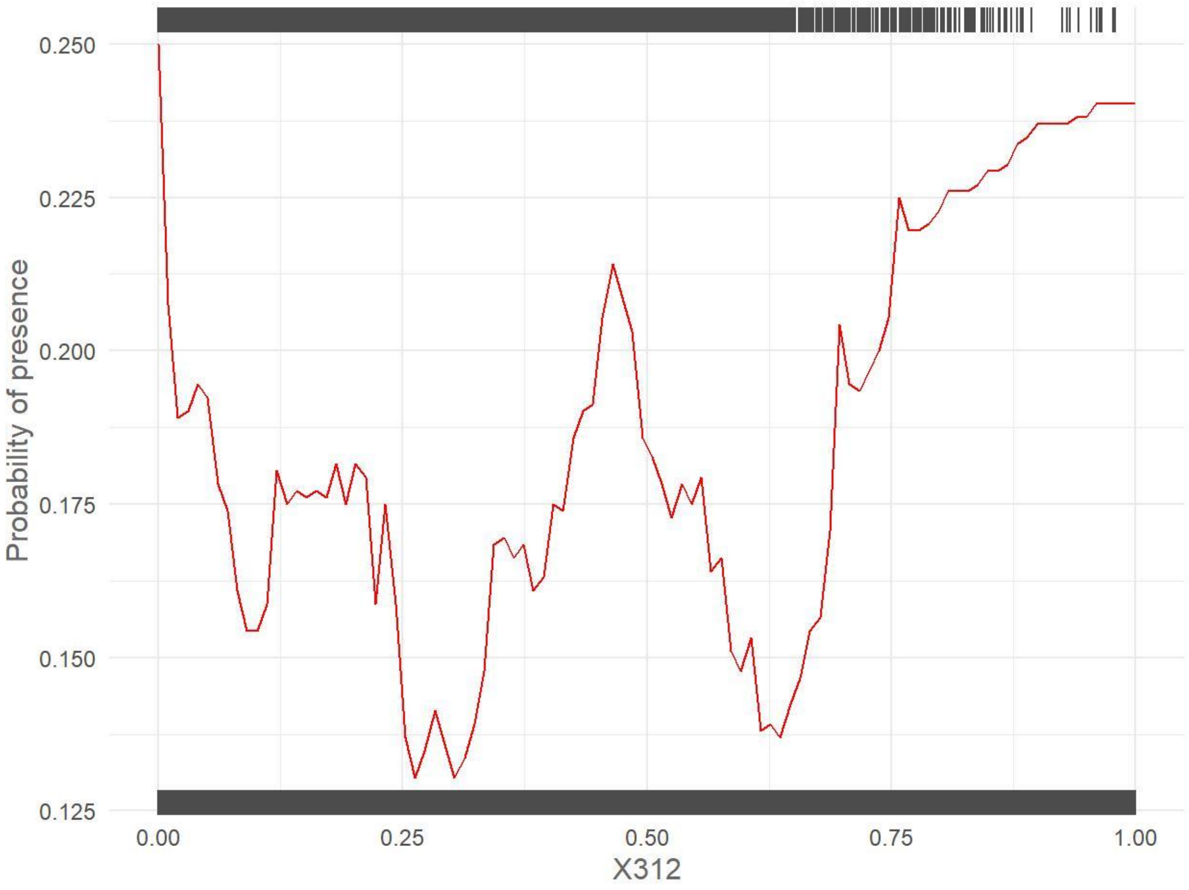
Probability of presence
0.250
0.225
0.200
0.175
0.150
0.125
0.00
0.25
0.50
0.75
1.00
X312

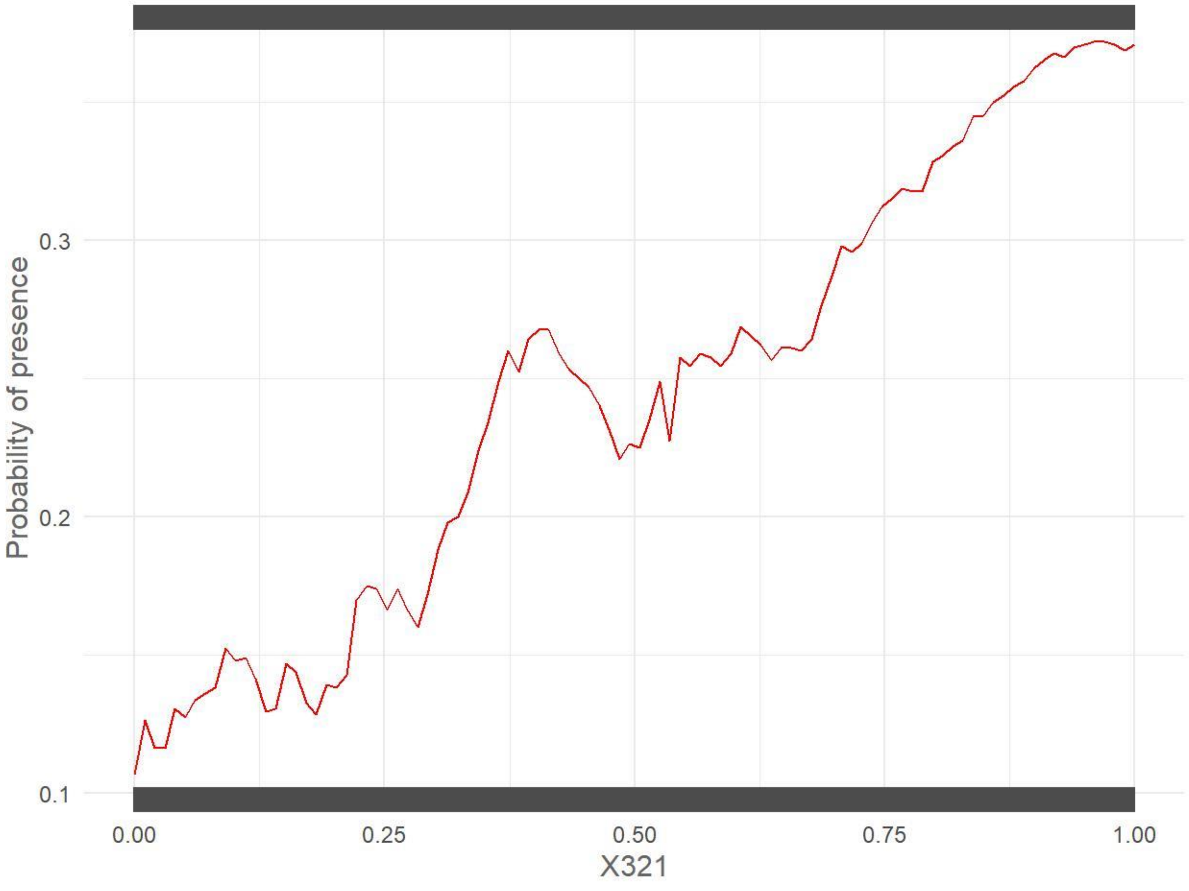
Probability of presence
0.3
0.2
0.1
0.00
0.25
0.50
0.75
1.00
X321

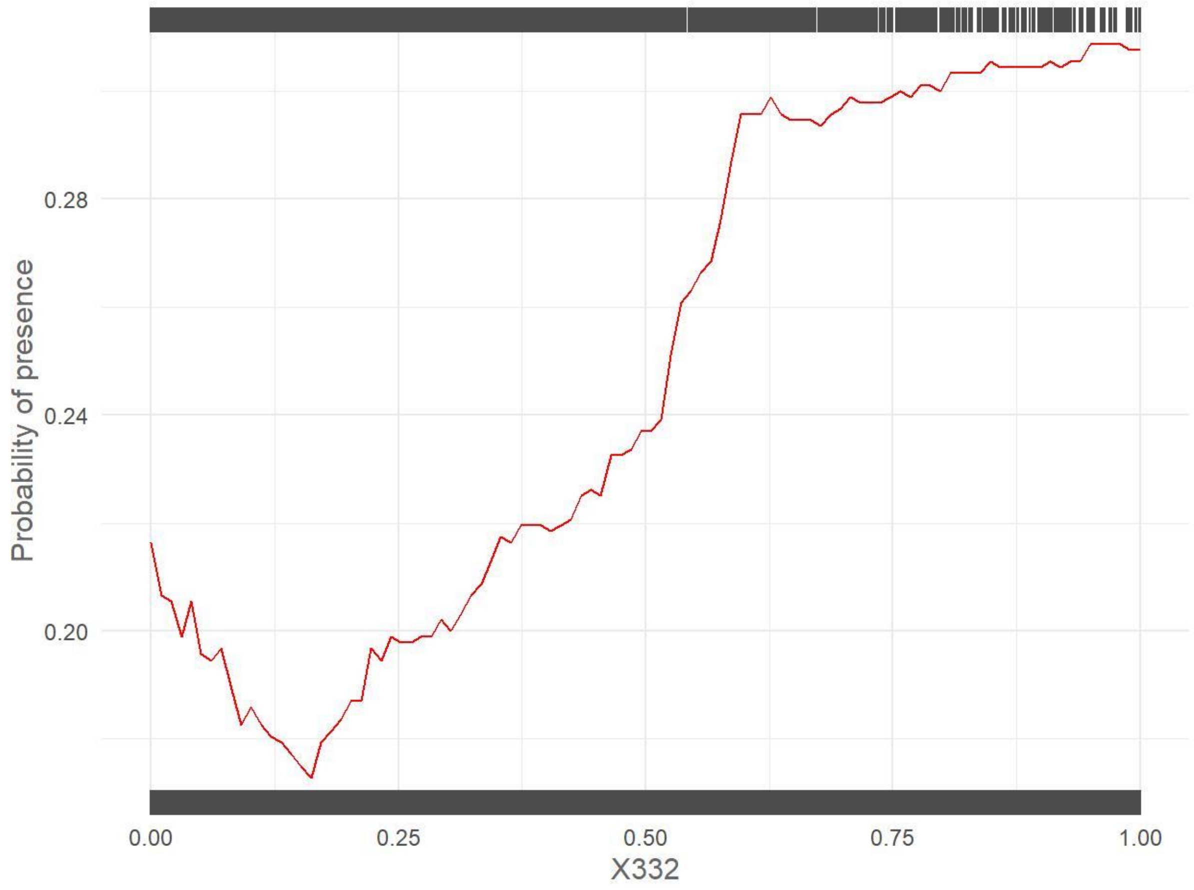
Probability of presence
0.20
0.24
0.28
0.00
0.25
0.50
0.75
1.00
X332

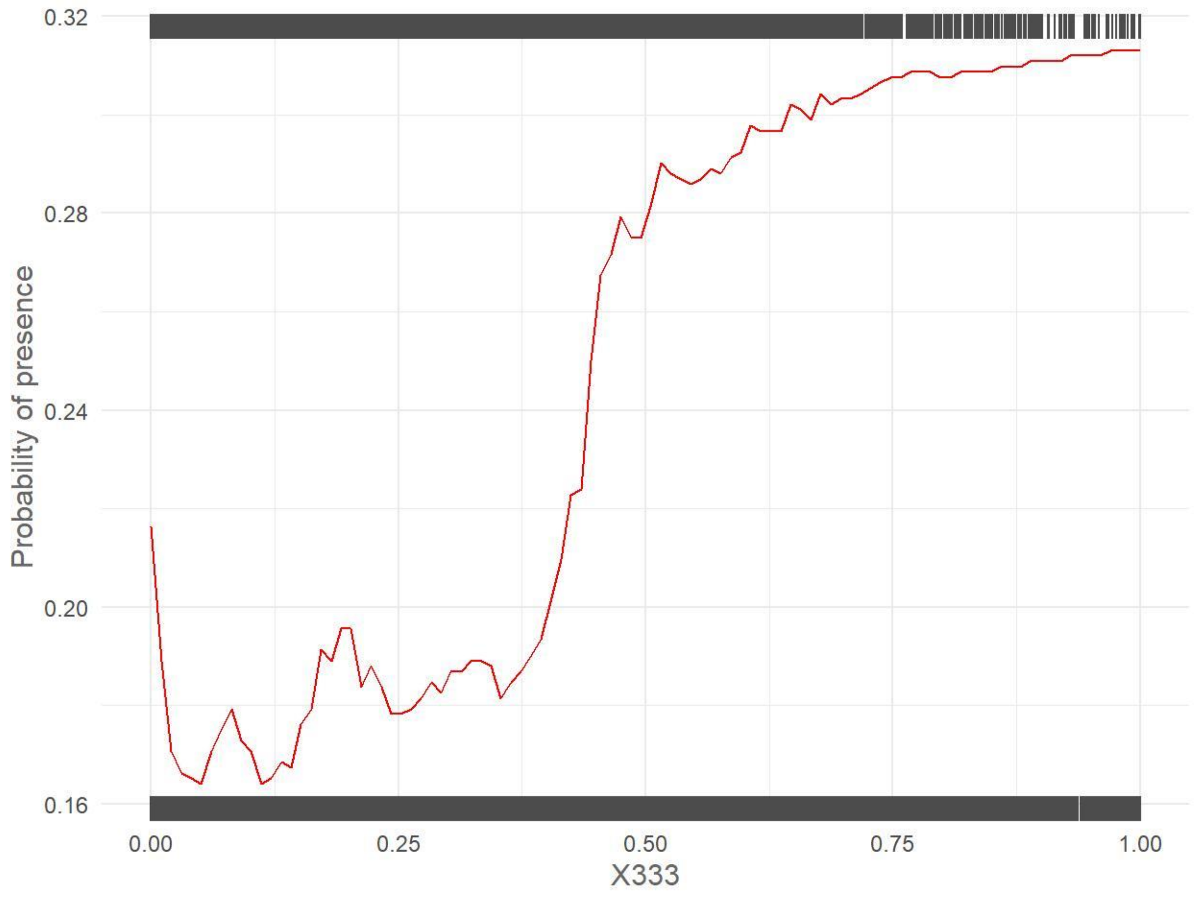
Probability of presence
0.16
0.20
0.24
0.28
0.32
0.00
0.25
0.50
0.75
1.00
X333

Figure S15. Species-habitat relationships according to the Random Forest model for alpine accentor.

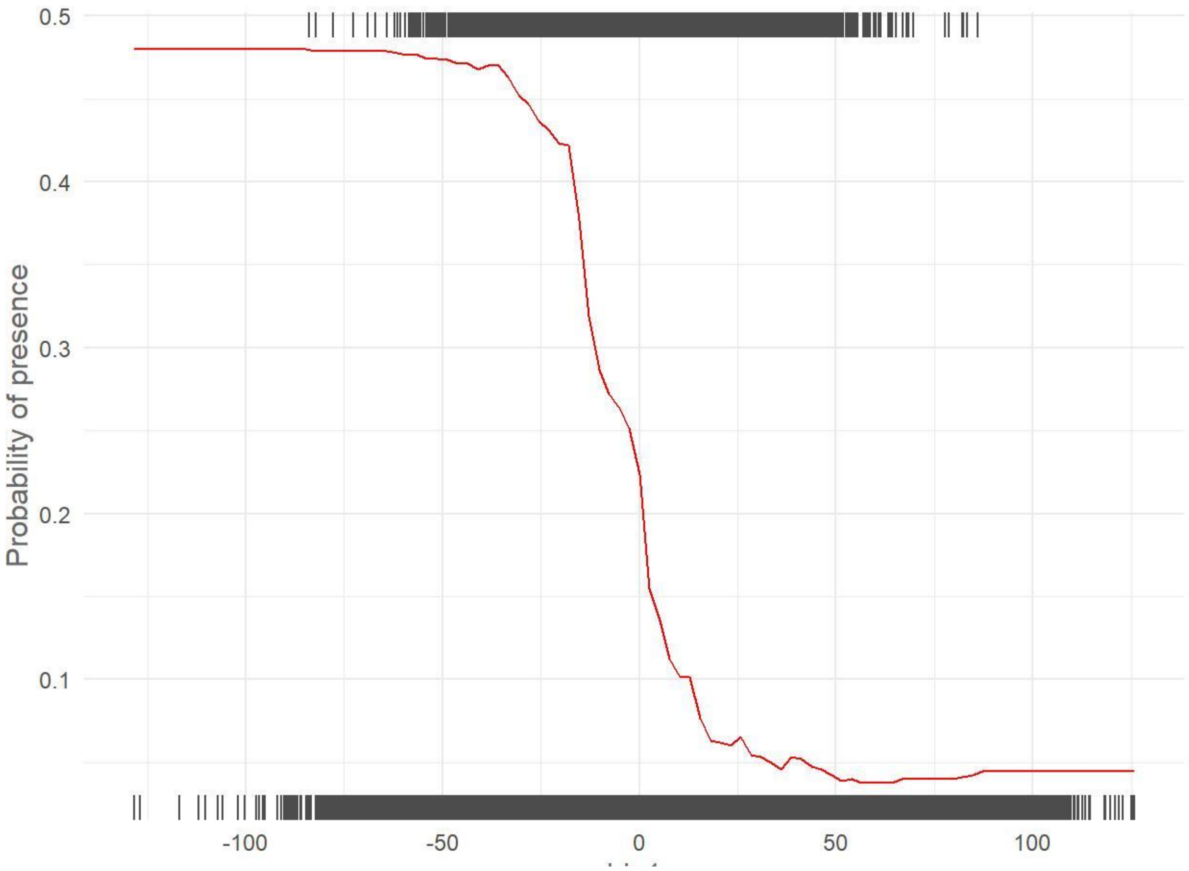
Probability of presence
0.5
0.4
0.3
0.2
0.1
-100
-50
0
50
100

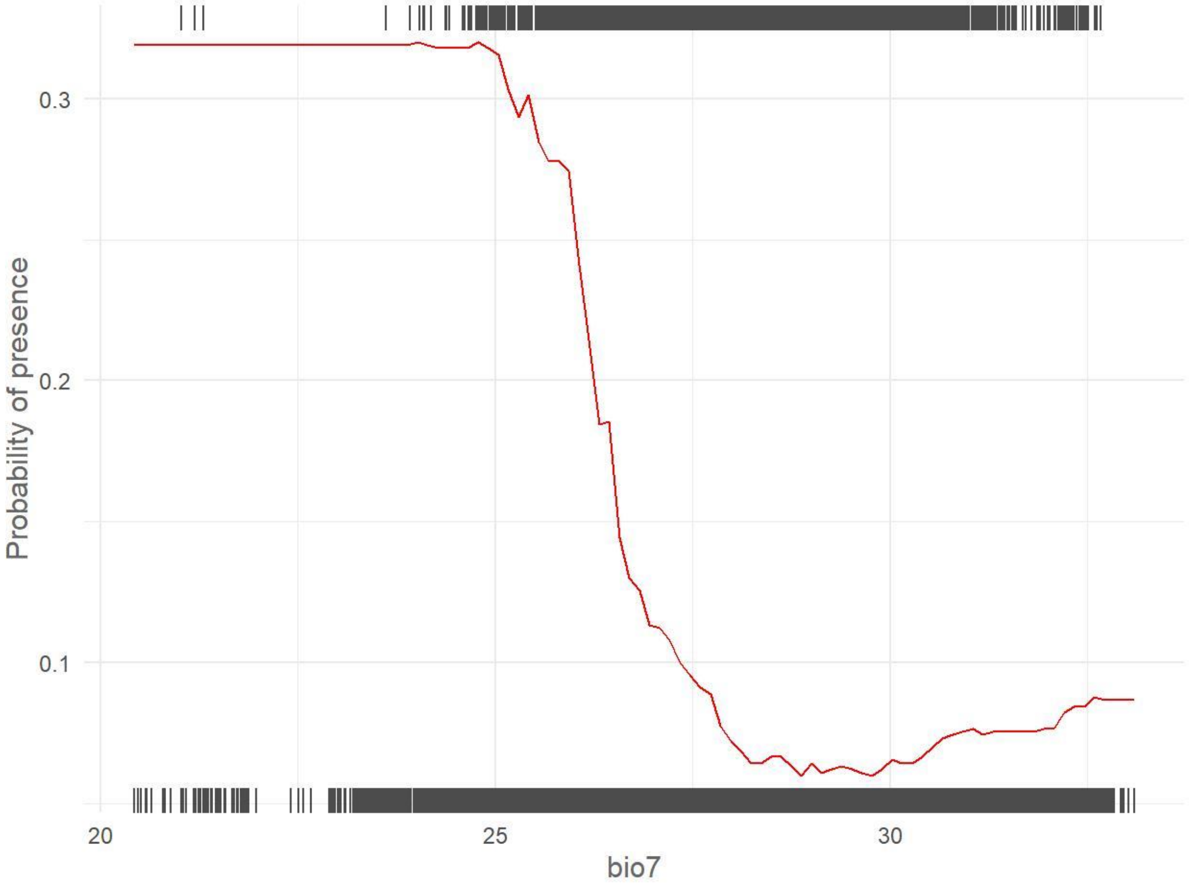
Probability of presence
0.3
0.2
0.1
20
25
30
bio7

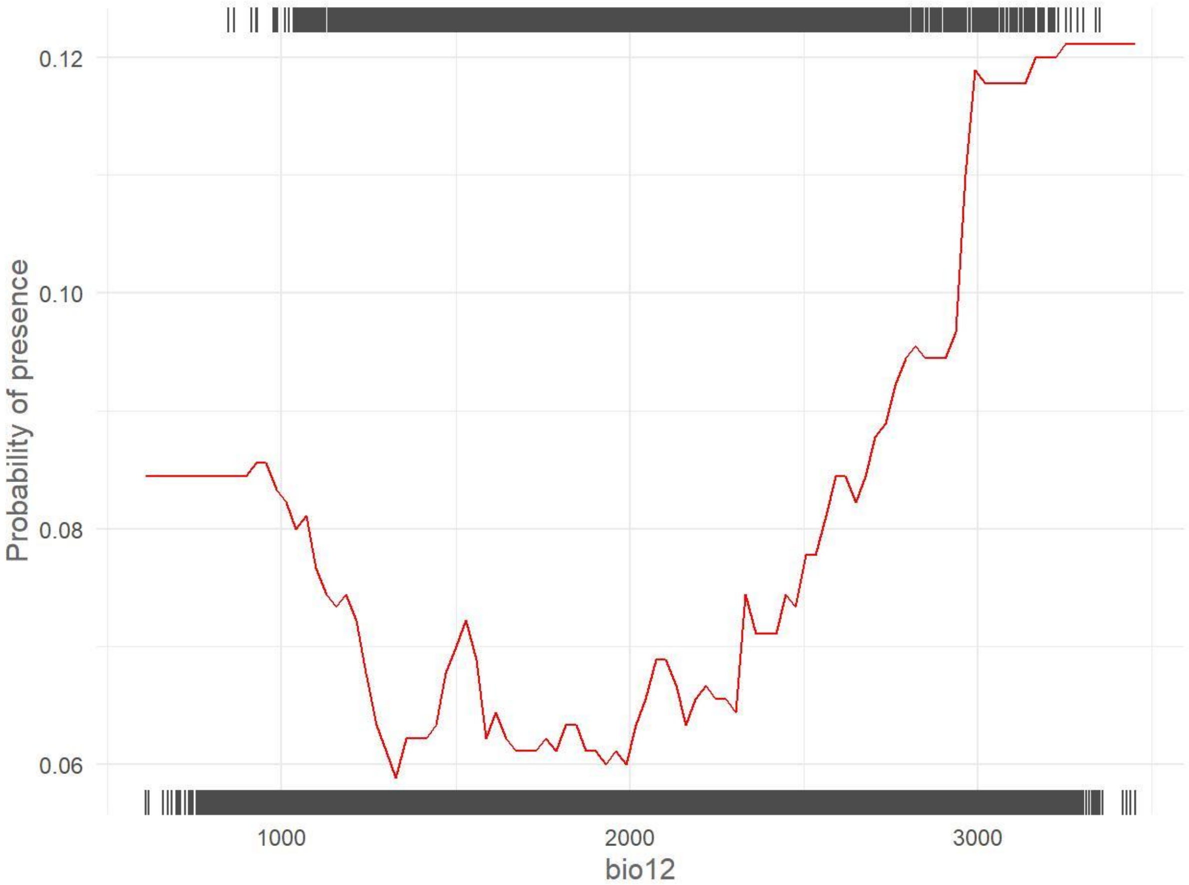

0.12
0.10
0.08
0.06
Probability of presence
1000
2000
3000
bio12

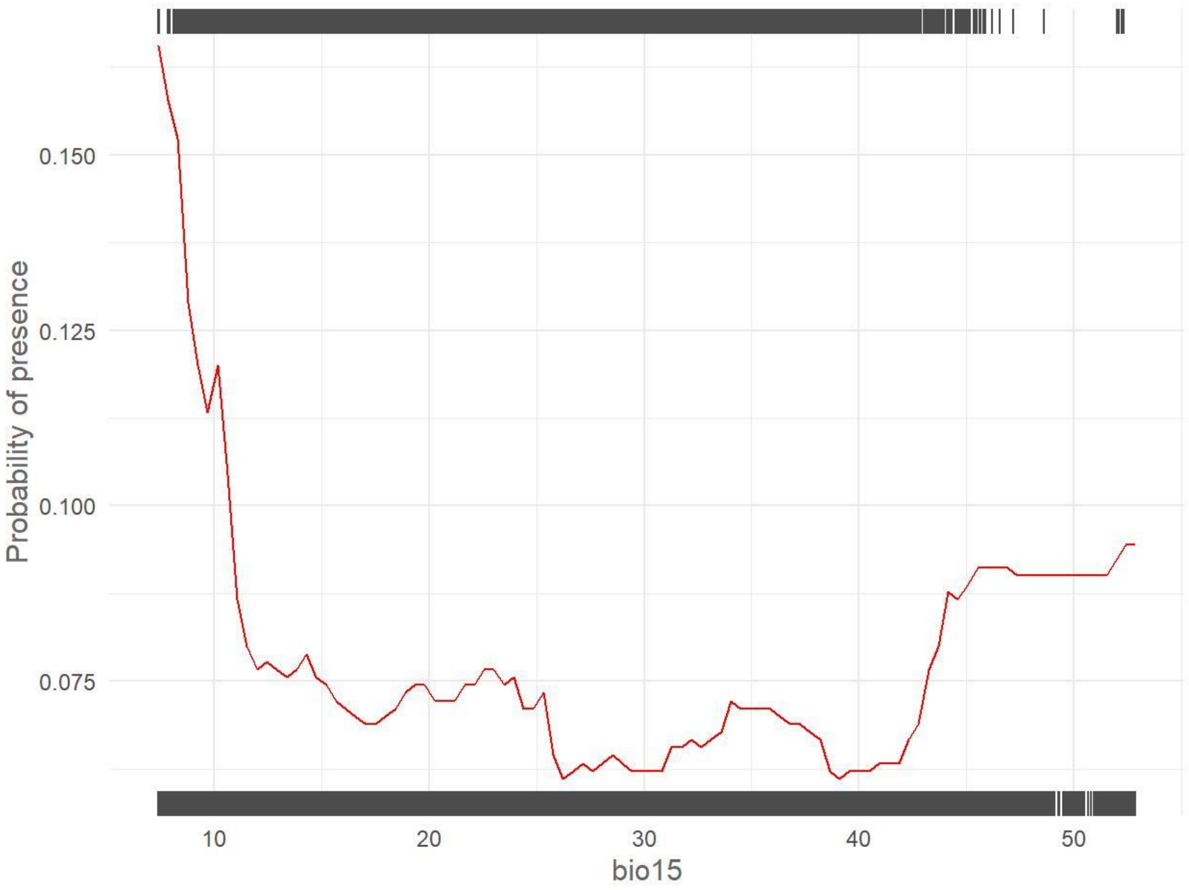

0.150
0.125
0.100
0.075
Probability of presence
10
20
30
40
50
bio15

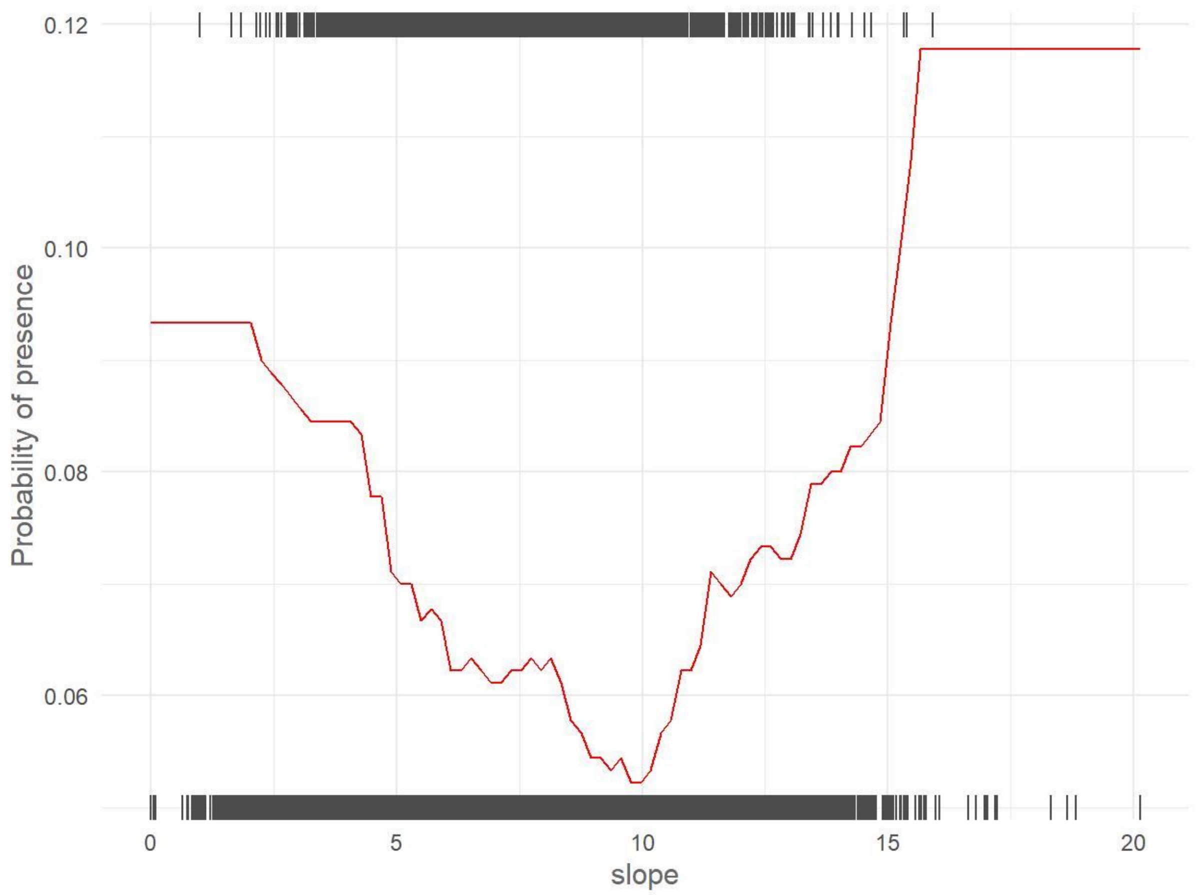

0.12
0.10
0.08
0.06
Probability of presence
0
5
10
15
20
slope

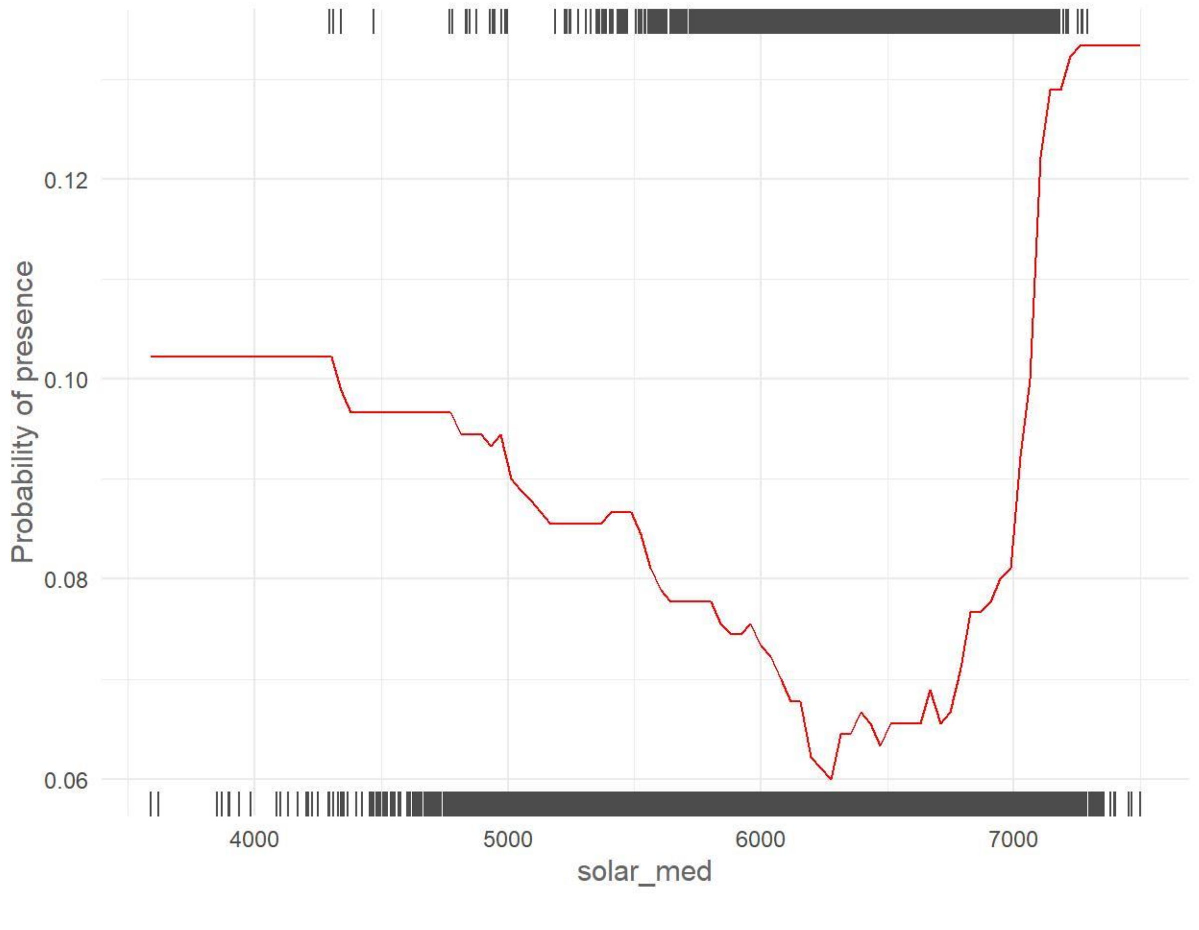

0.12
0.10
0.08
0.06
Probability of presence
4000
5000
6000
7000
solar_med

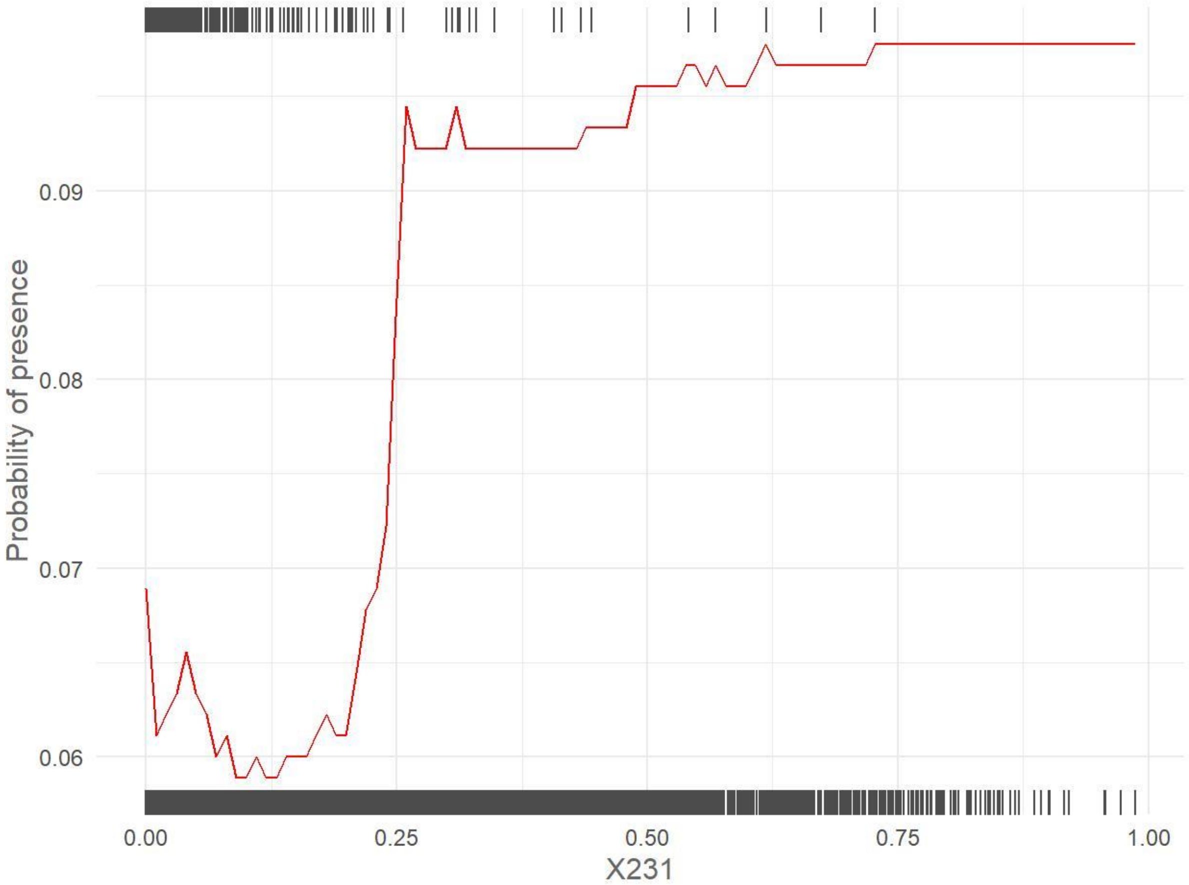
0.09
0.08
0.07
0.06
Probability of presence
0.00
0.25
0.50
0.75
1.00
X231

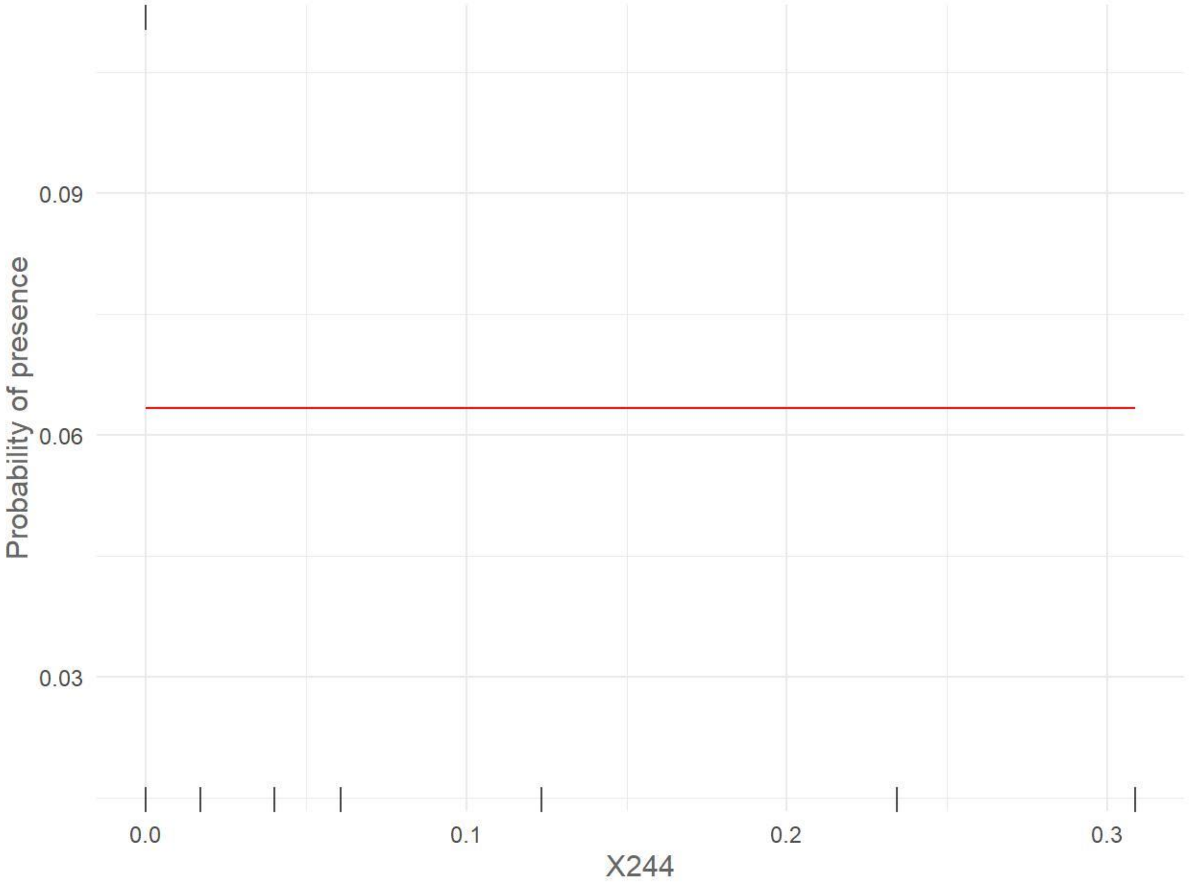
0.09
0.06
0.03
Probability of presence
0.0
0.1
0.2
0.3
X244

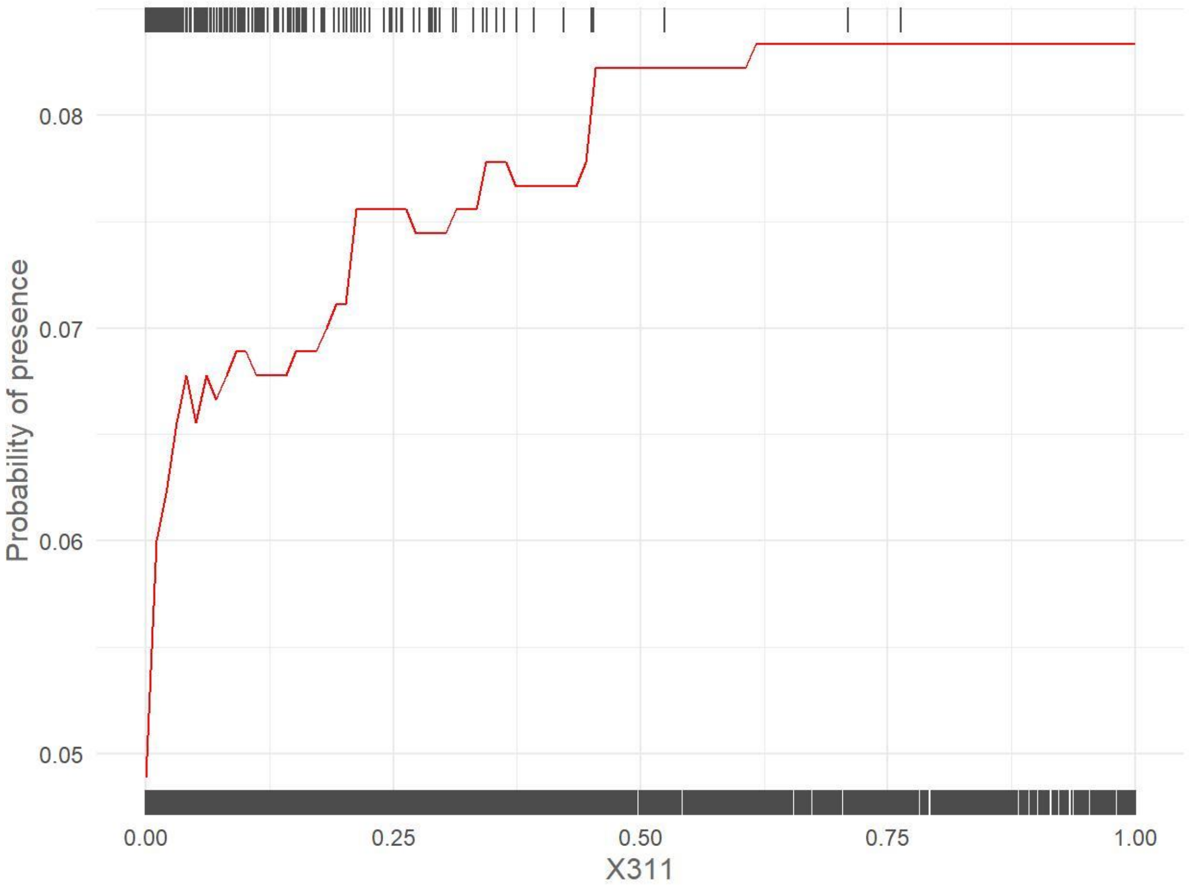
Probability of presence
0.05
0.06
0.07
0.08
0.00
0.25
0.50
0.75
1.00
X311

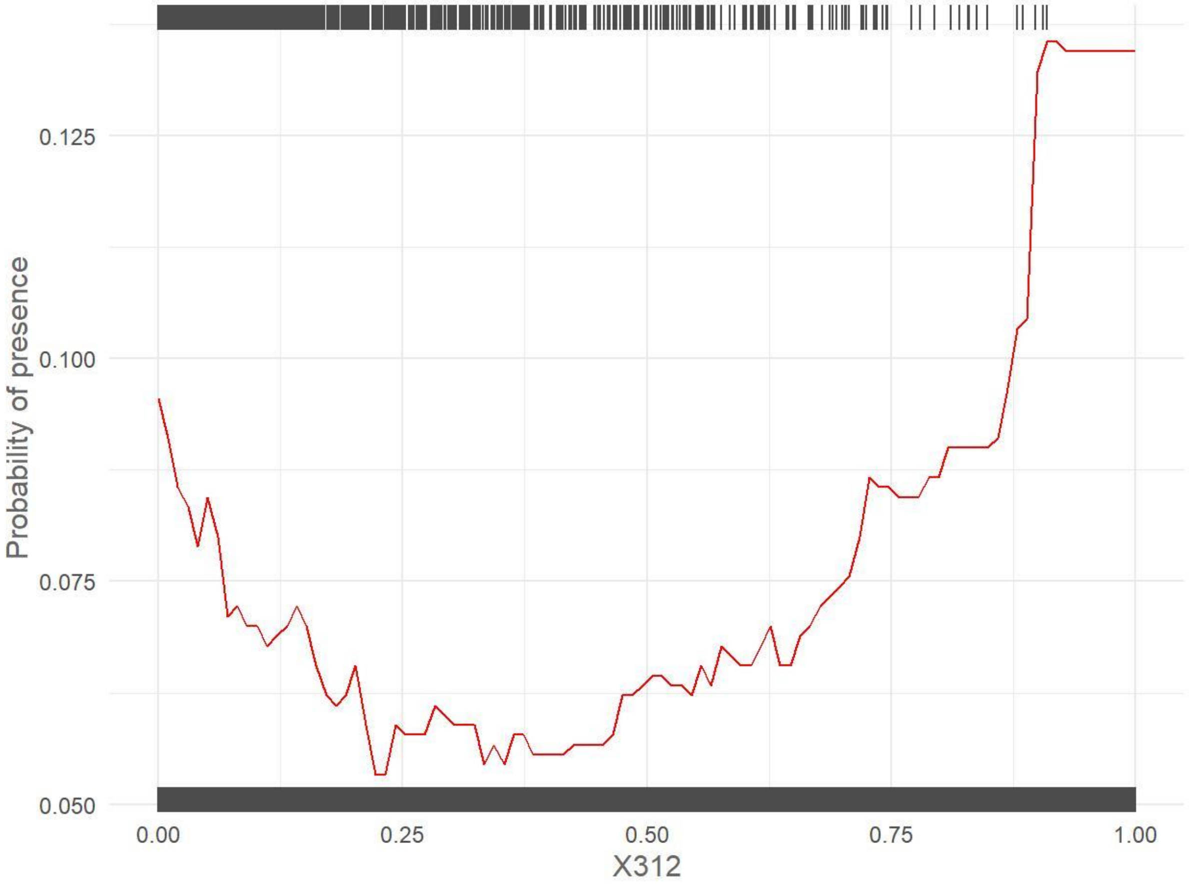
Probability of presence
0.050
0.075
0.100
0.125
0.00
0.25
0.50
0.75
1.00
X312

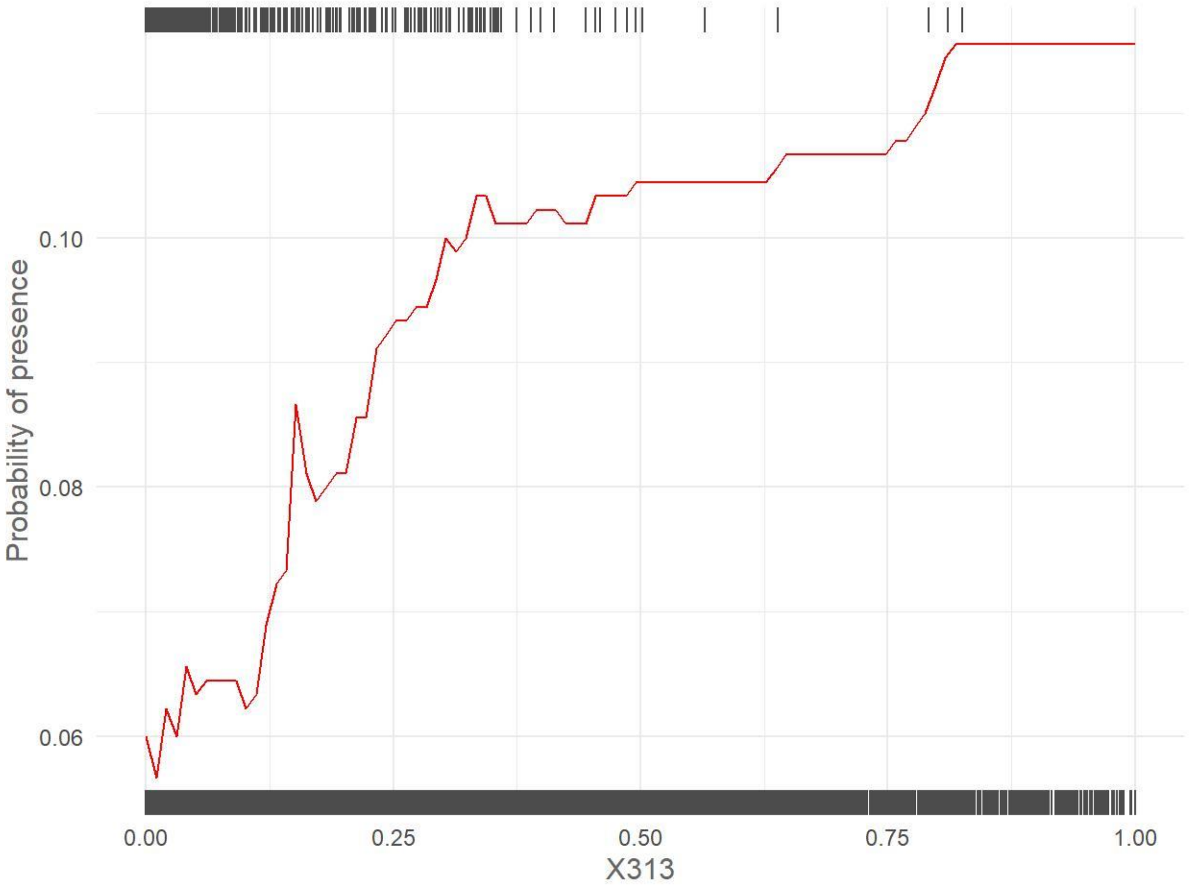

Probability of presence
0.06
0.08
0.10
0.00
0.25
0.50
0.75
1.00
X313

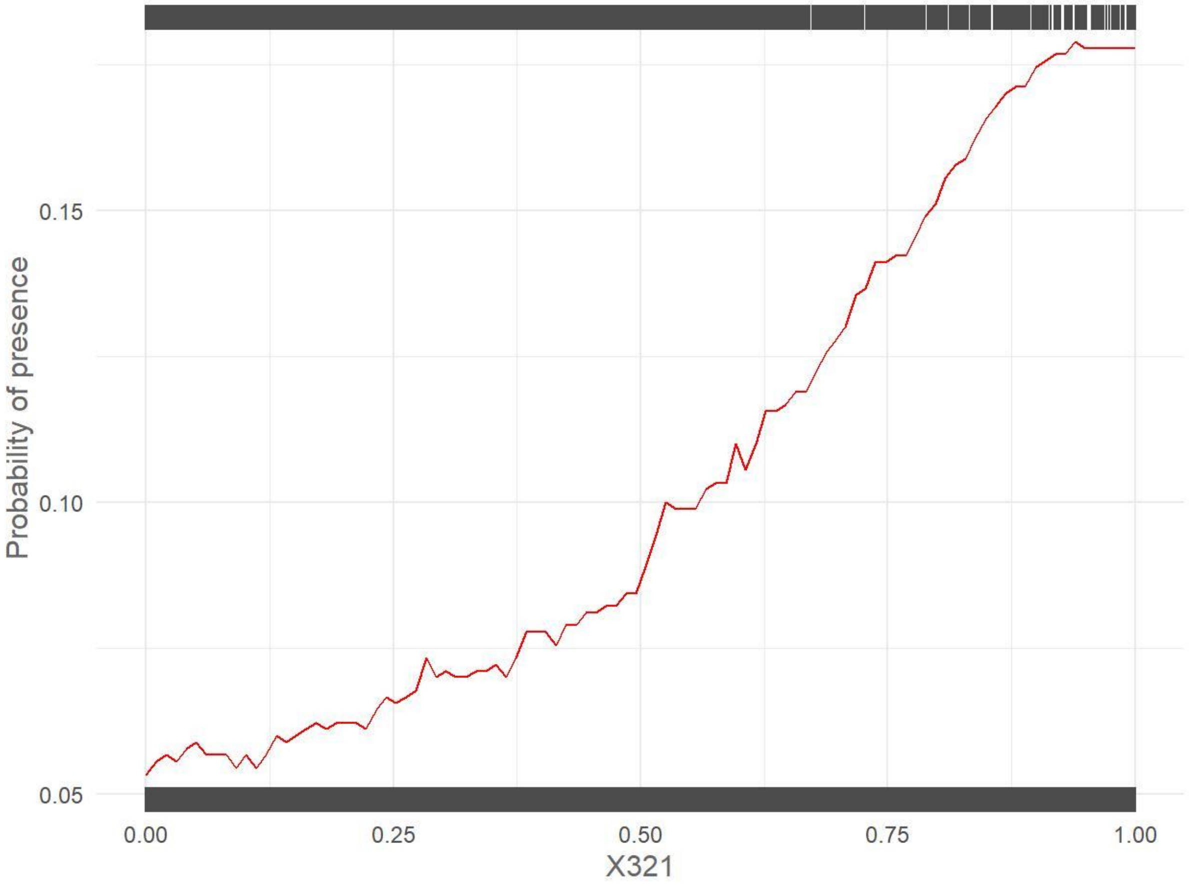

Probability of presence
0.05
0.10
0.15
0.00
0.25
0.50
0.75
1.00
X321

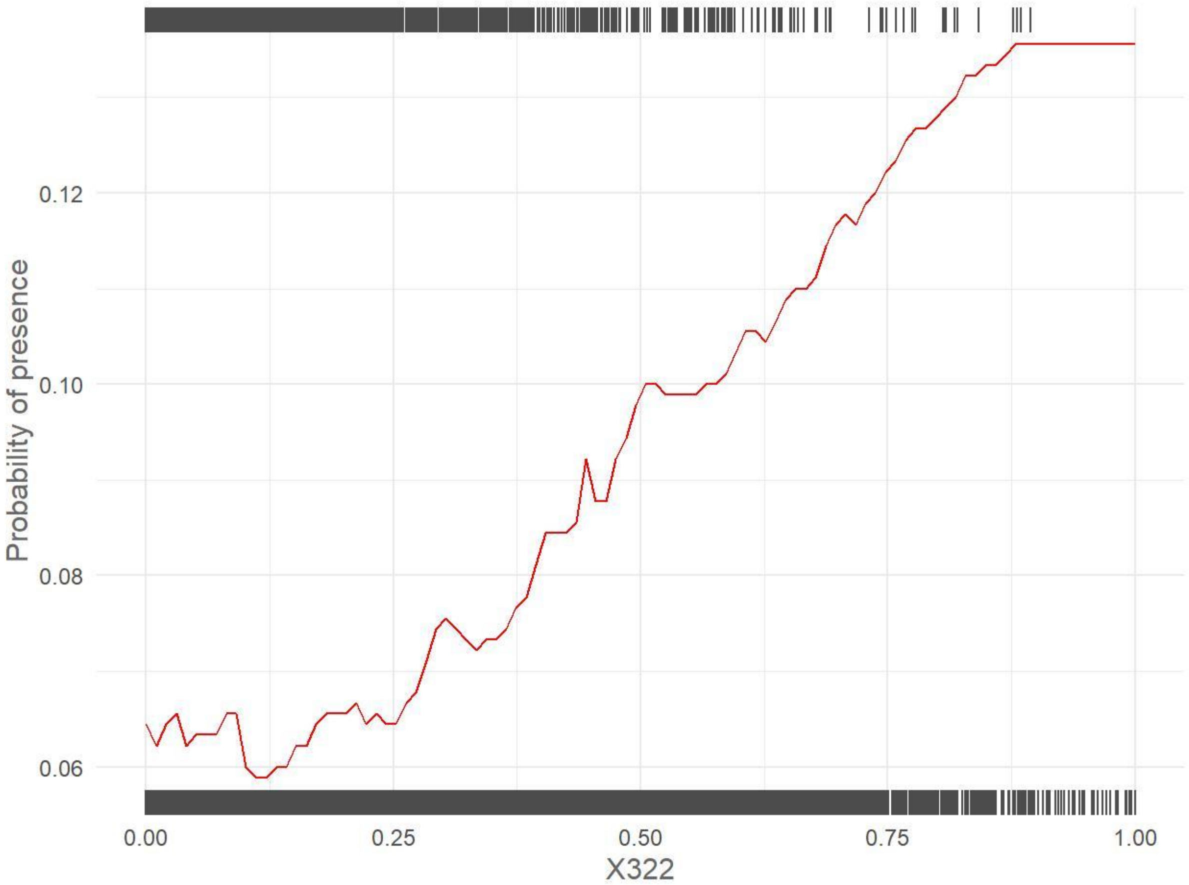
Probability of presence
0.06
0.08
0.10
0.12
0.00
0.25
0.50
0.75
1.00
X322

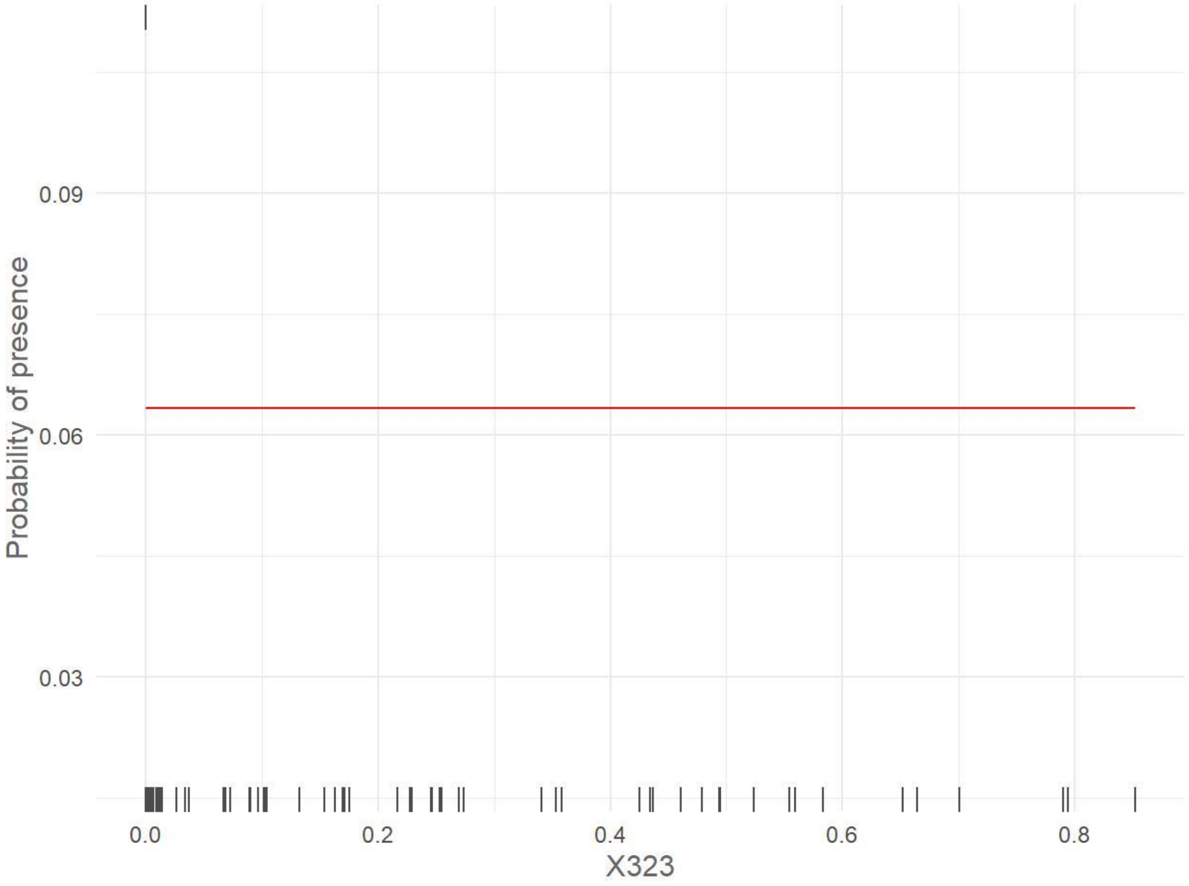
Probability of presence
0.03
0.06
0.09
0.0
0.2
0.4
0.6
0.8
X323

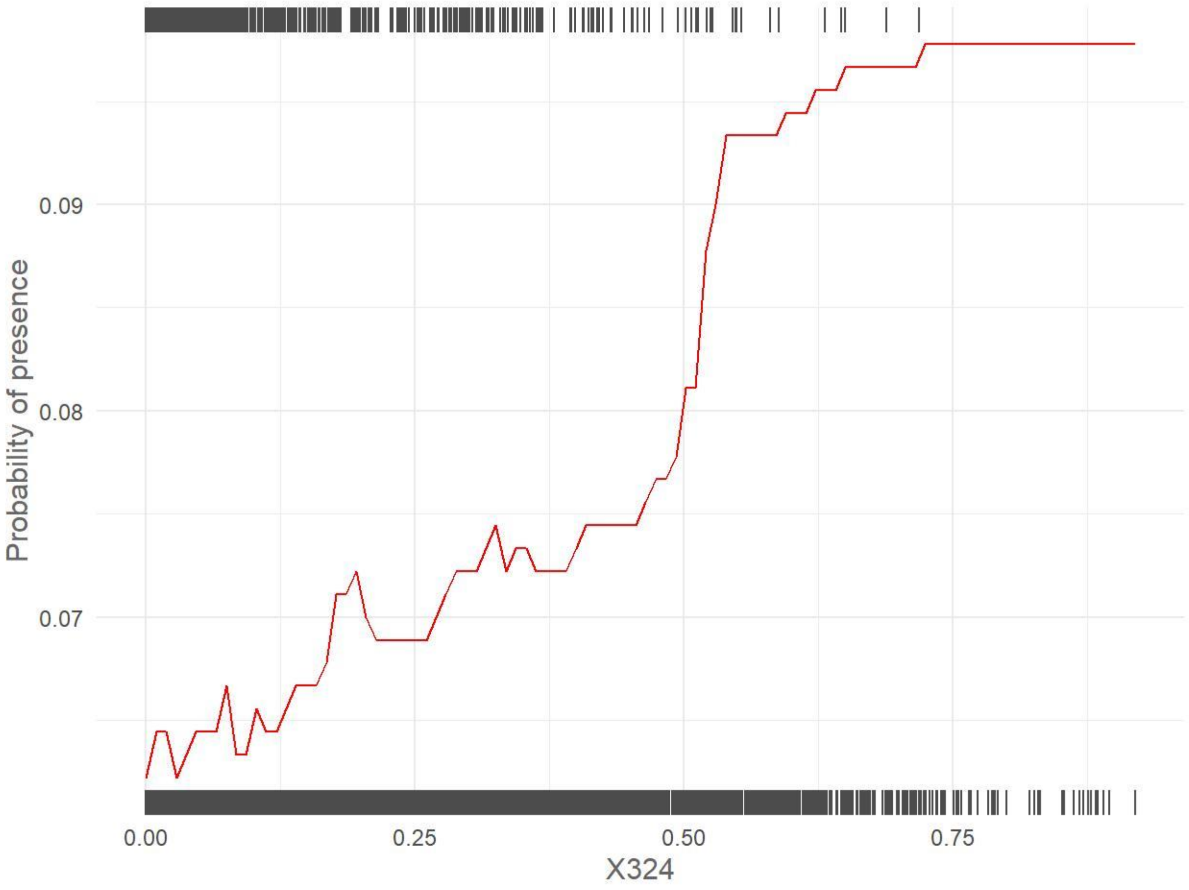

Probability of presence
0.09
0.08
0.07
0.00
0.25
0.50
0.75
X324

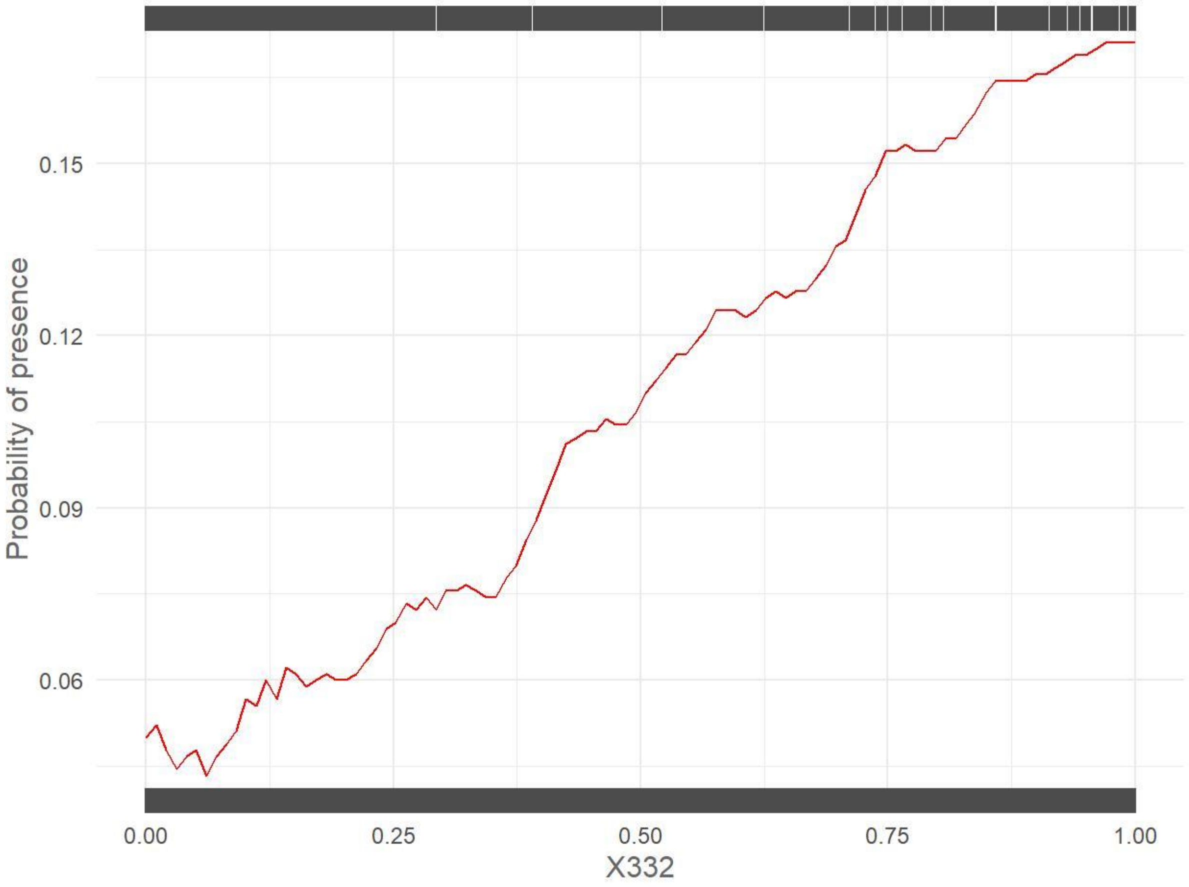

Probability of presence
0.15
0.12
0.09
0.06
0.00
0.25
0.50
0.75
1.00
X332

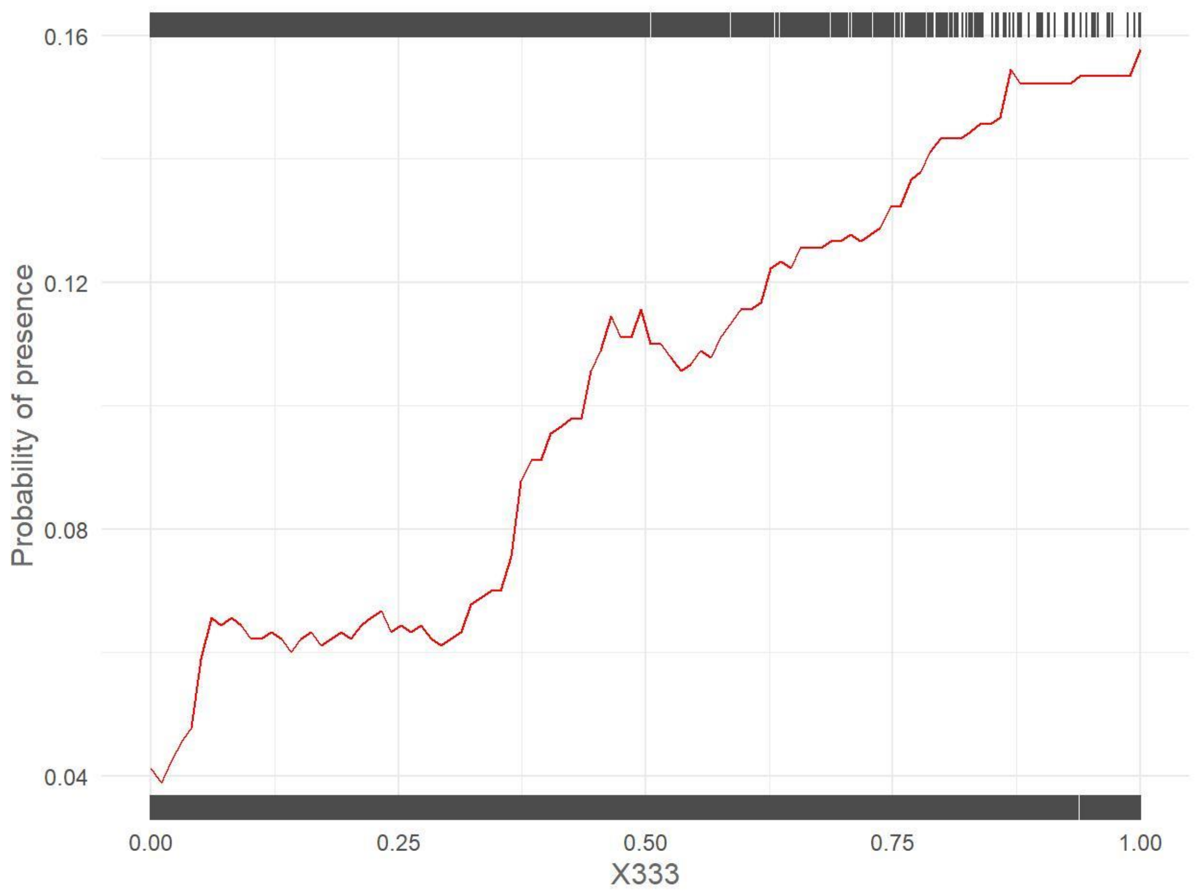

Probability of presence
0.16
0.12
0.08
0.04
0.00
0.25
0.50
0.75
1.00
X333

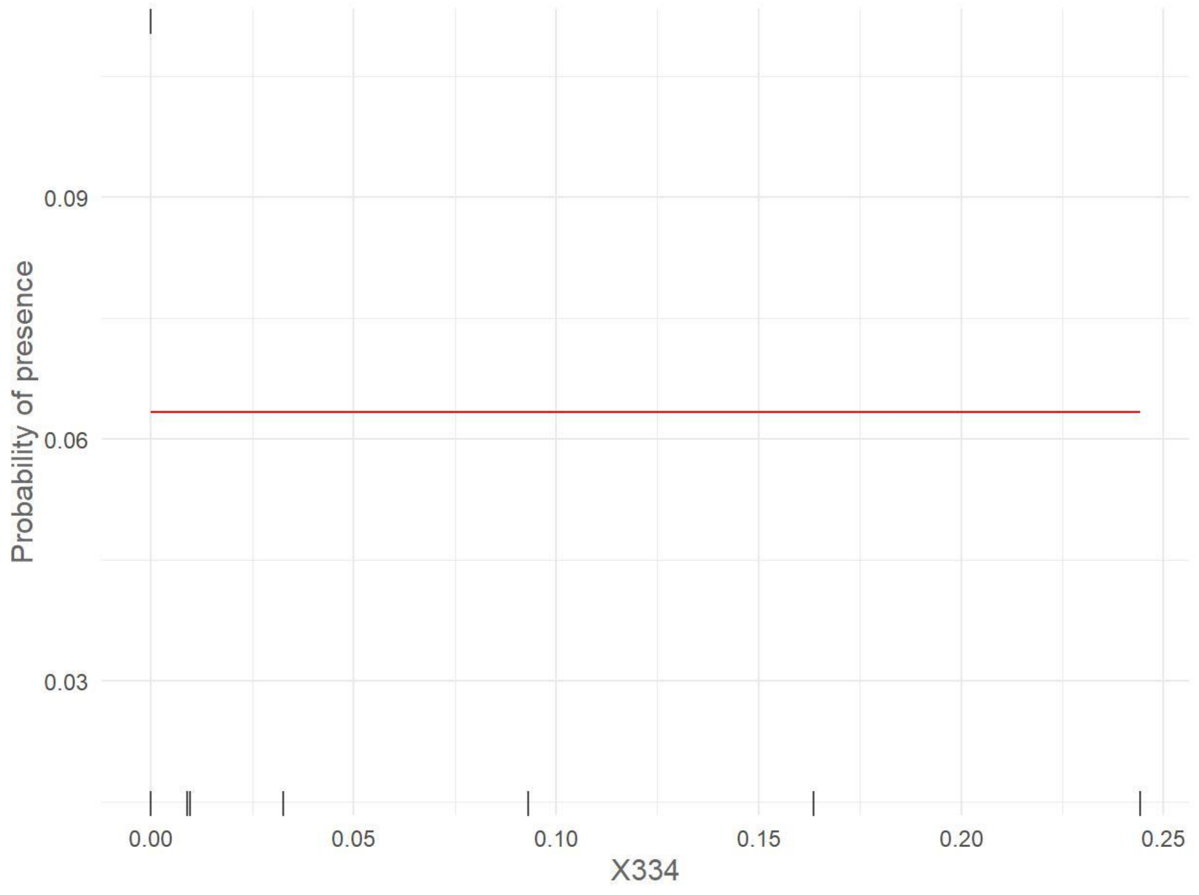

Probability of presence
0.09
0.06
0.03
0.00
0.05
0.10
0.15
0.20
0.25
X334

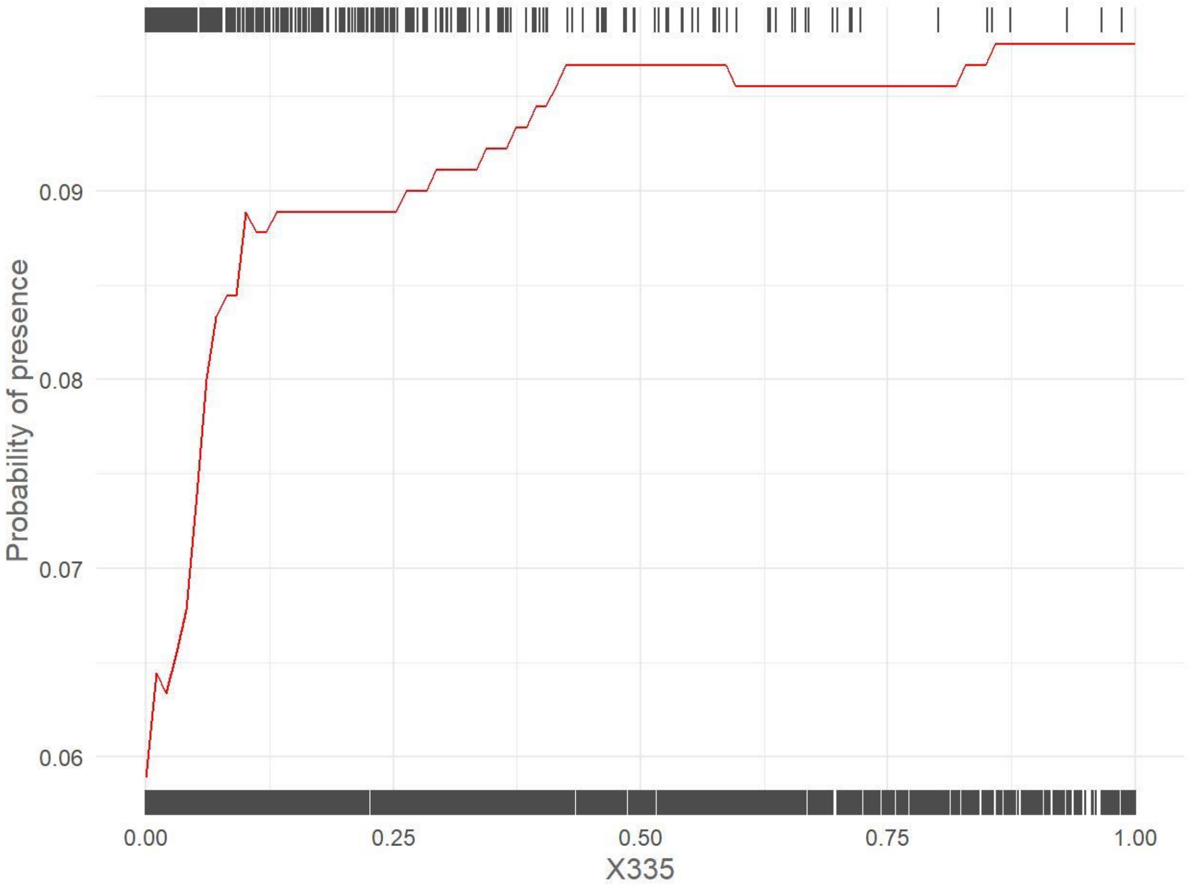

Probability of presence
0.06
0.07
0.08
0.09
0.00
0.25
0.50
0.75
1.00
X335

Figure S16. Species-habitat relationships according to the Random Forest model for white-winged snowfinch.

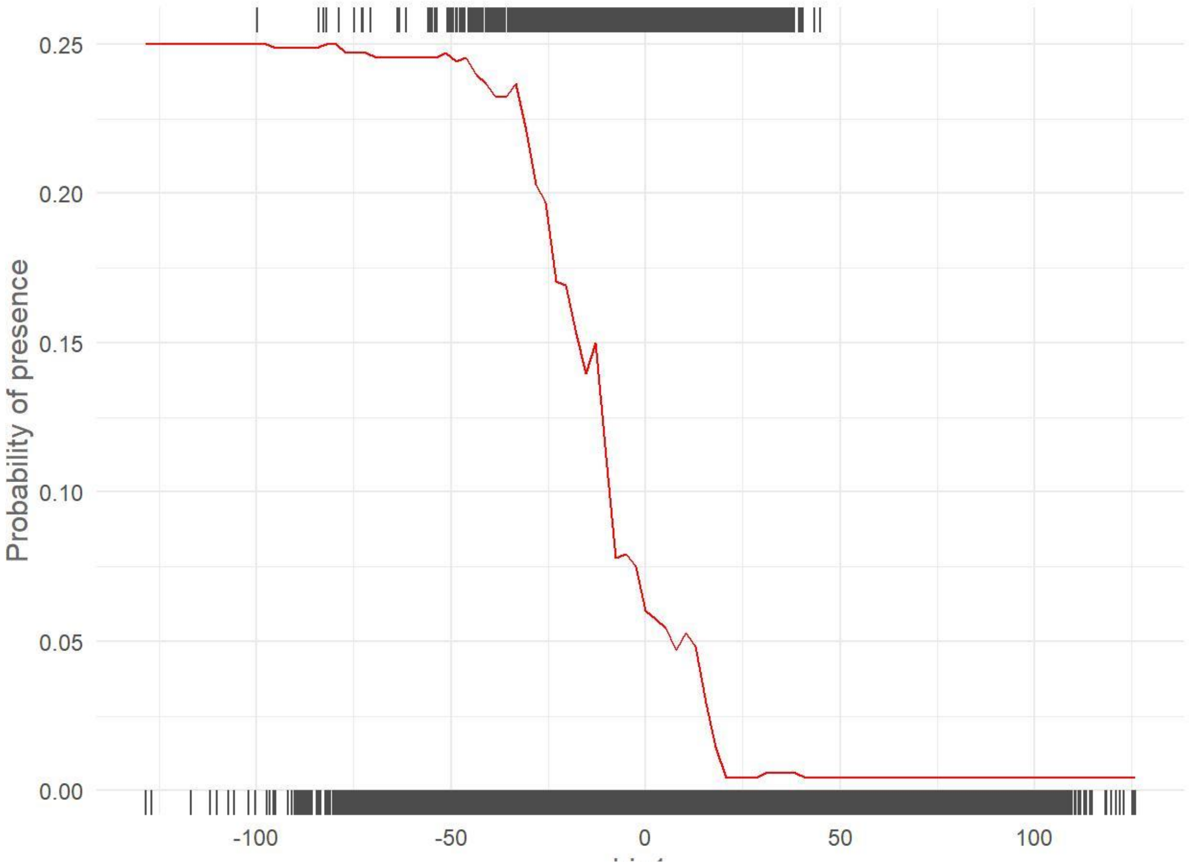
Probability of presence
0.25
0.20
0.15
0.10
0.05
0.00
-100
-50
0
50
100

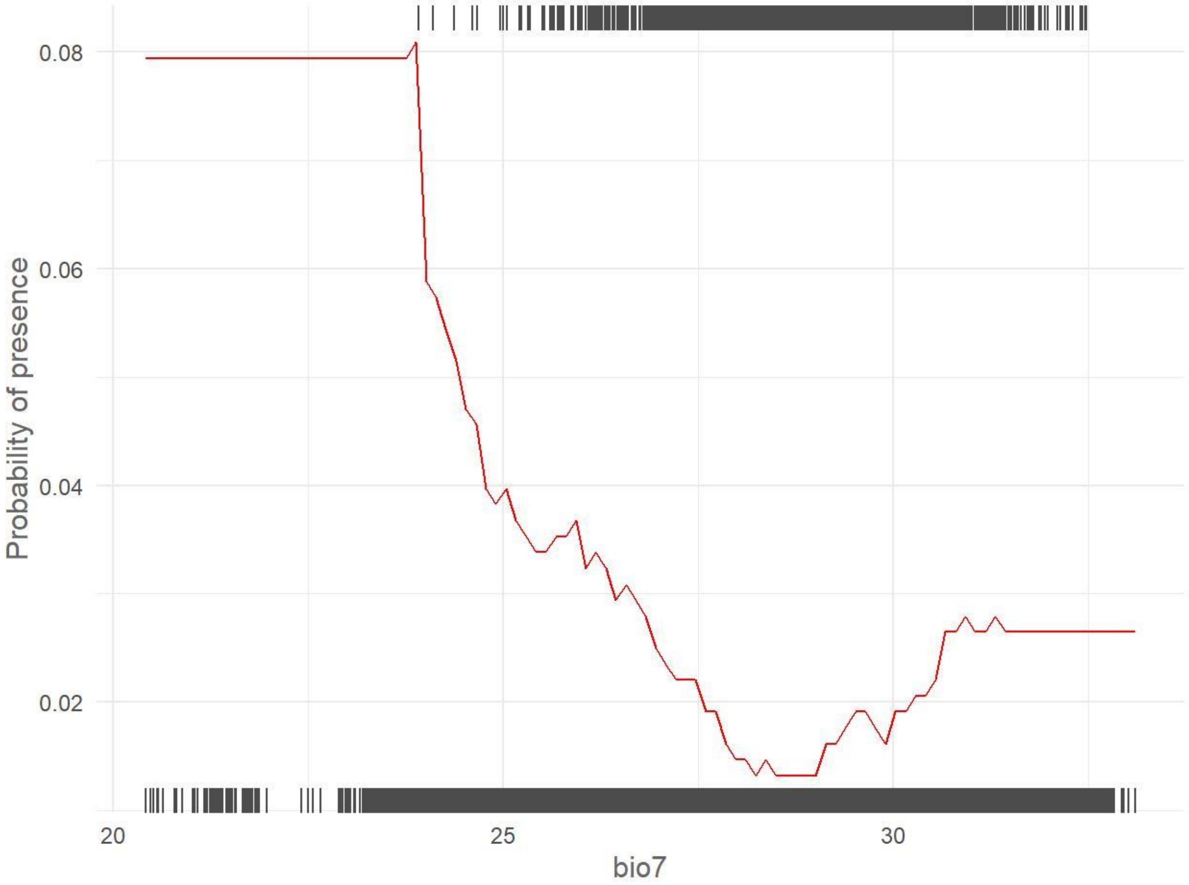
Probability of presence
0.08
0.06
0.04
0.02
20
25
30
bio7

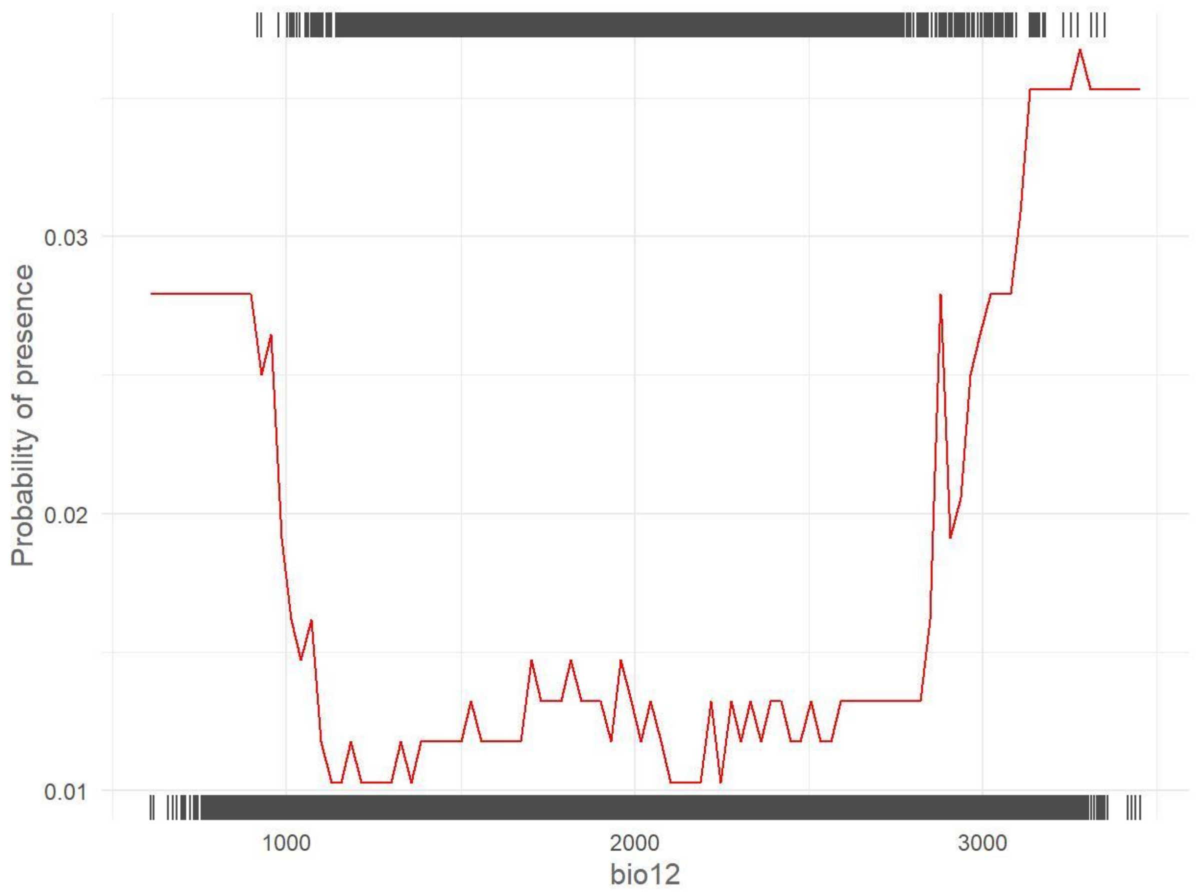

Probability of presence
0.01
0.02
0.03
1000
2000
3000
bio12

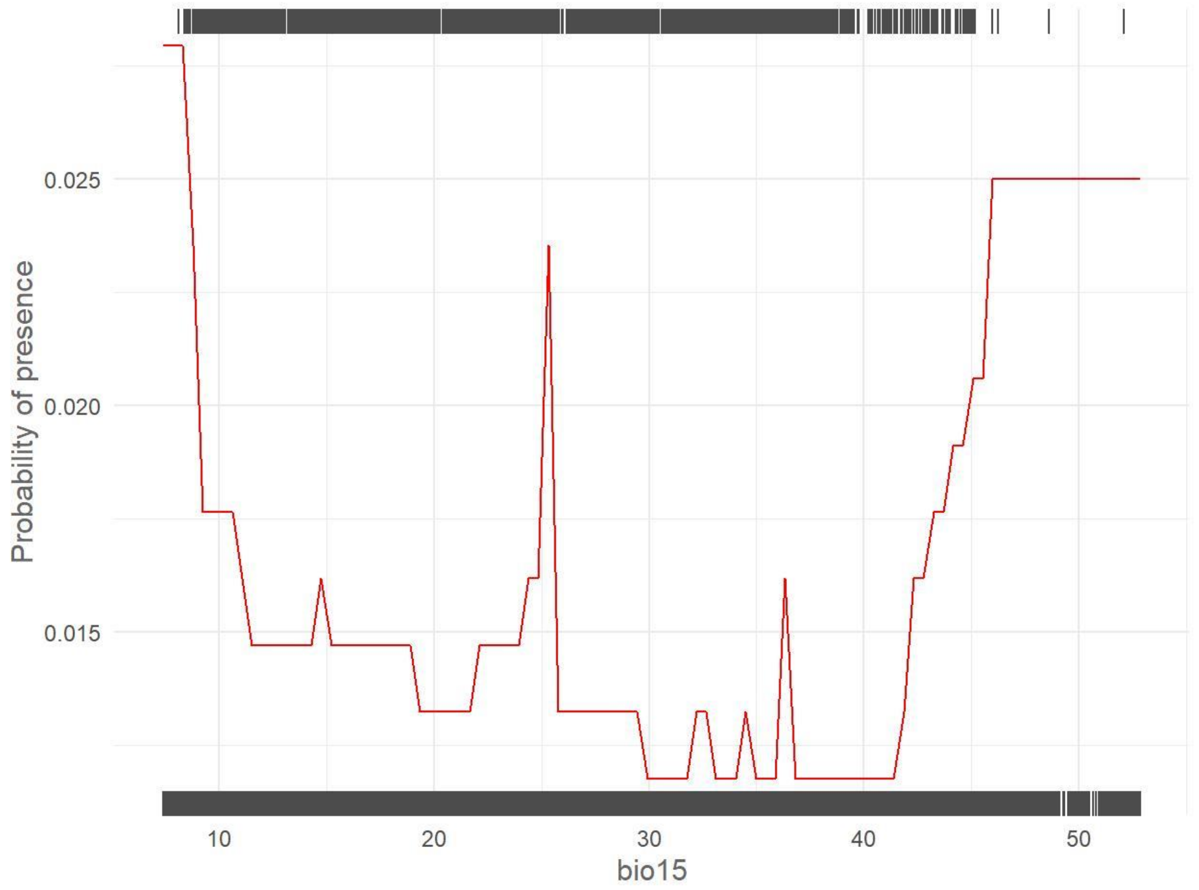

Probability of presence
0.015
0.020
0.025
10
20
30
40
50
bio15

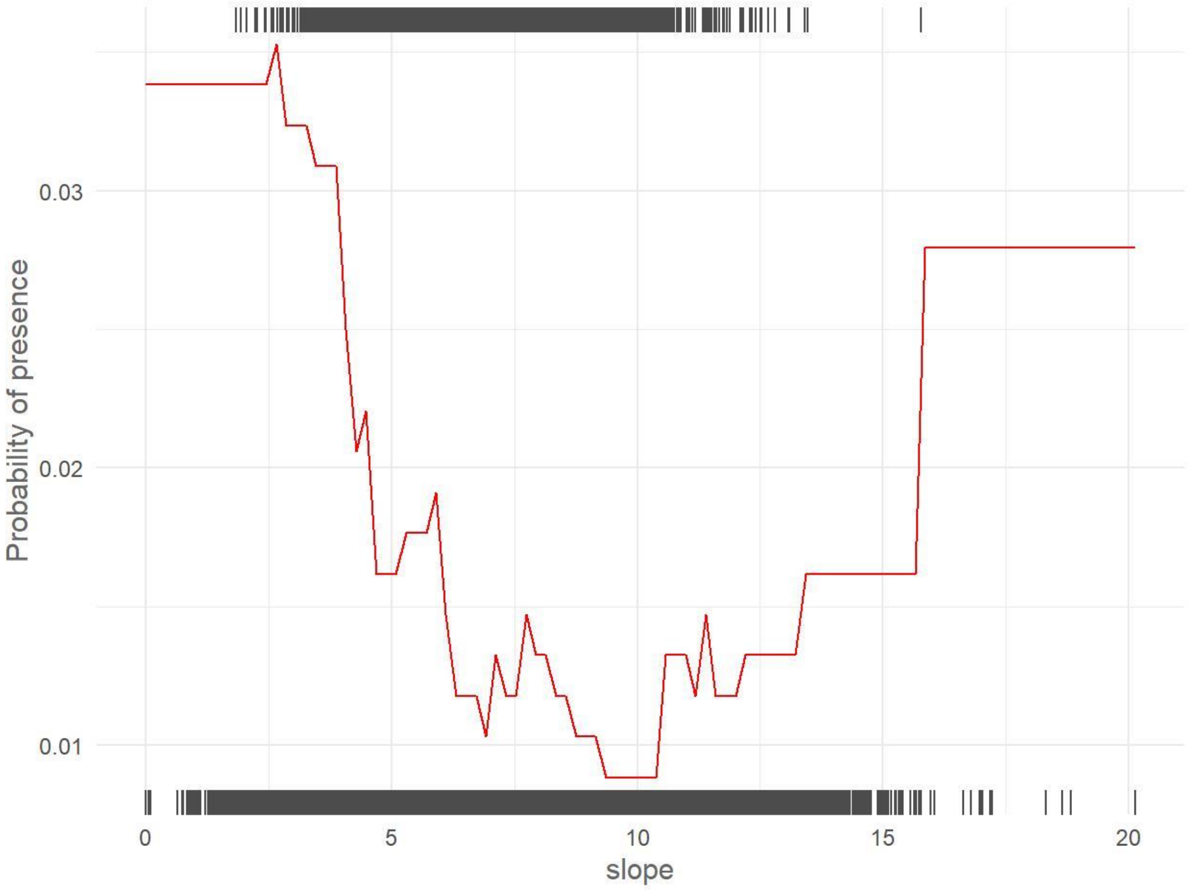

Probability of presence
0.03
0.02
0.01
0
5
10
15
20
slope

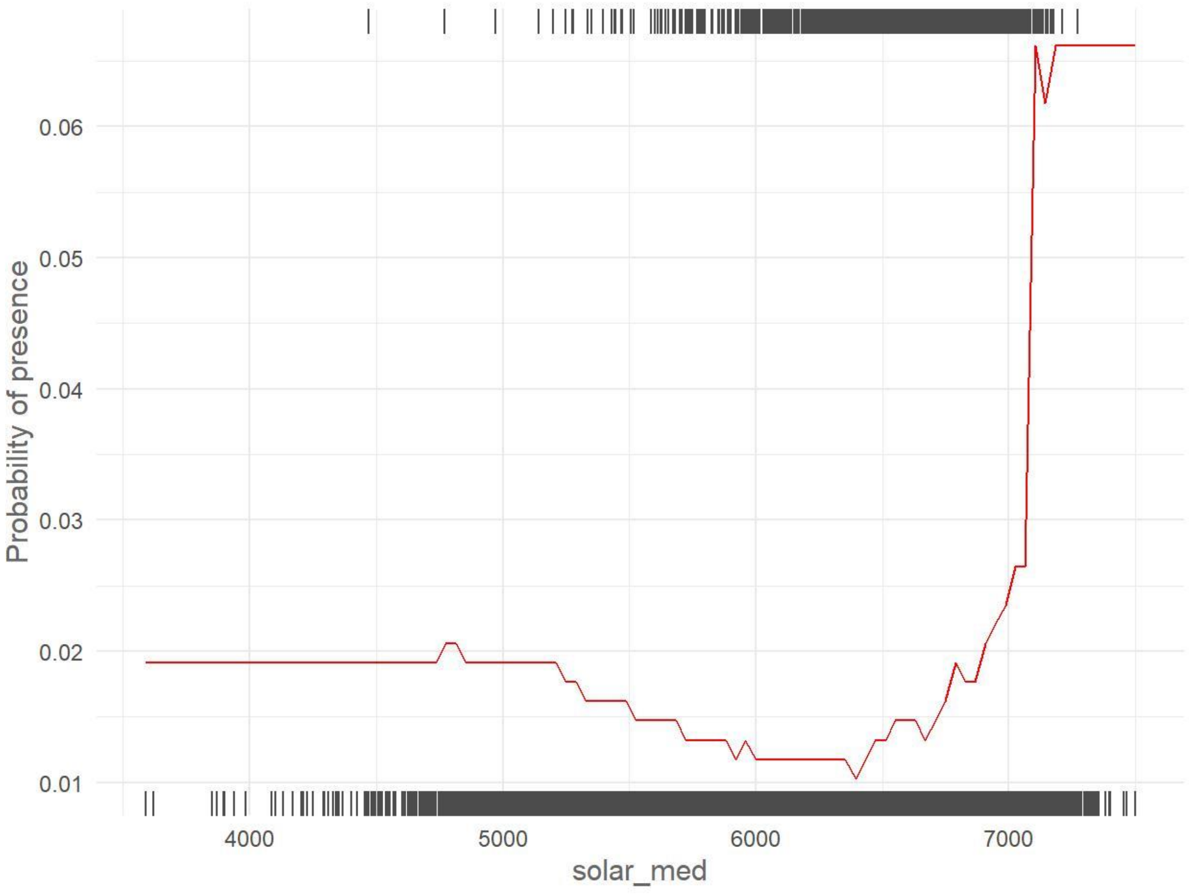

Probability of presence
0.06
0.05
0.04
0.03
0.02
0.01
4000
5000
6000
7000
solar_med

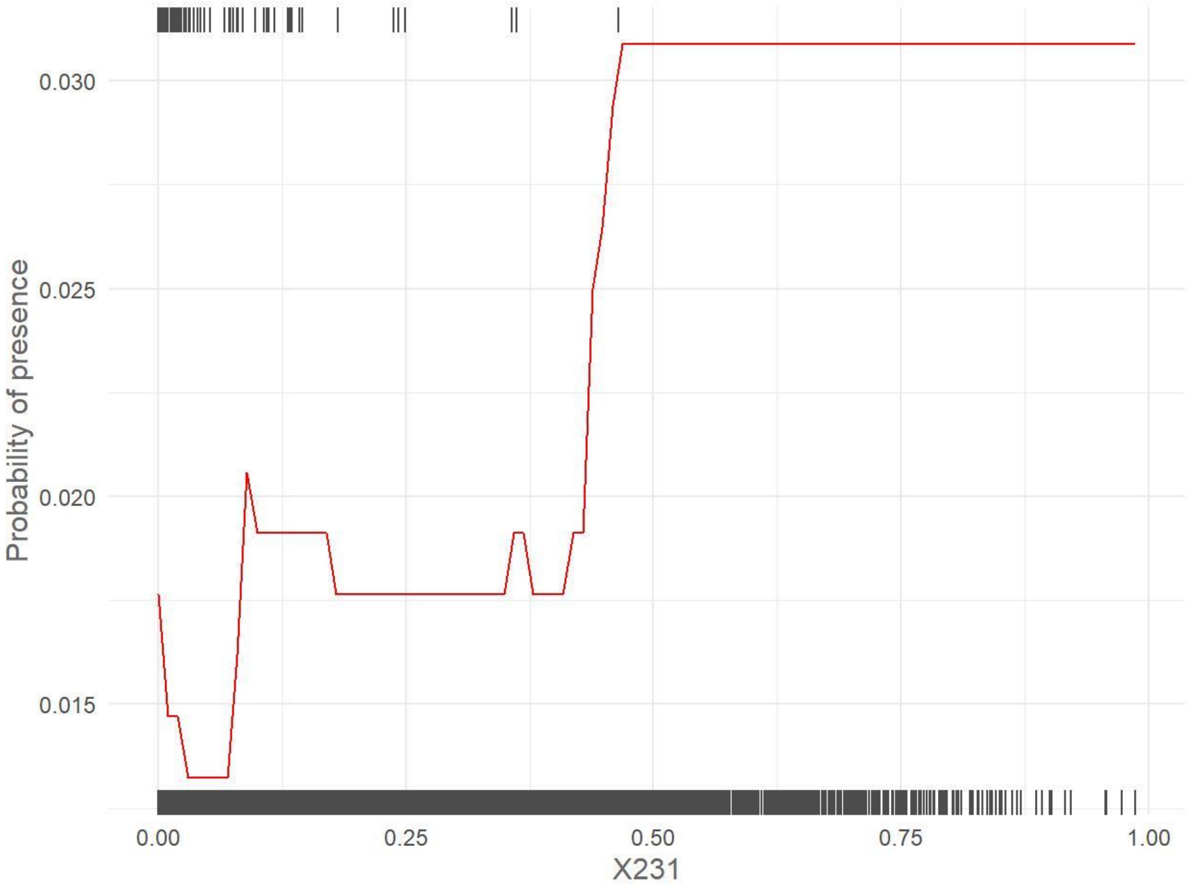

0.030
0.025
0.020
0.015
Probability of presence
0.00
0.25
0.50
0.75
1.00
X231

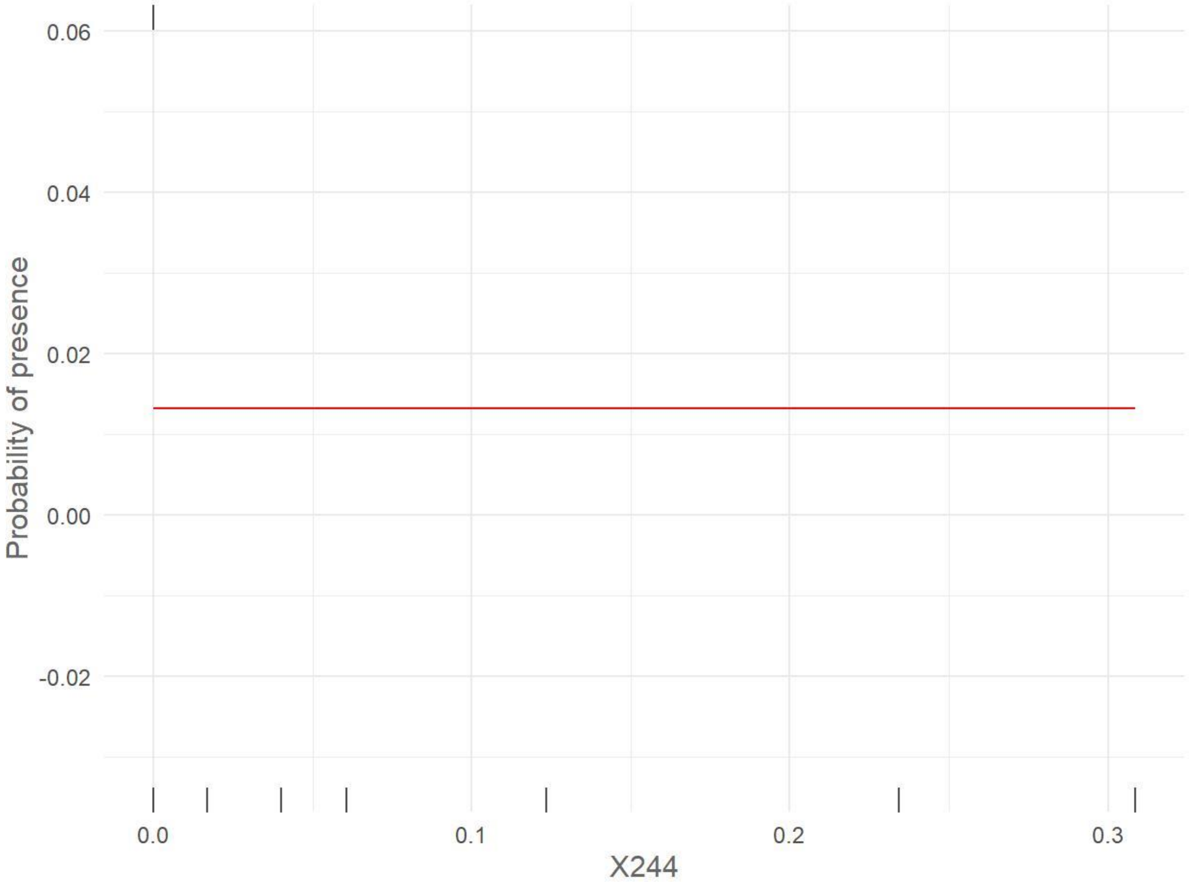

0.06
0.04
0.02
0.00
-0.02
Probability of presence
0.0
0.1
0.2
0.3
X244

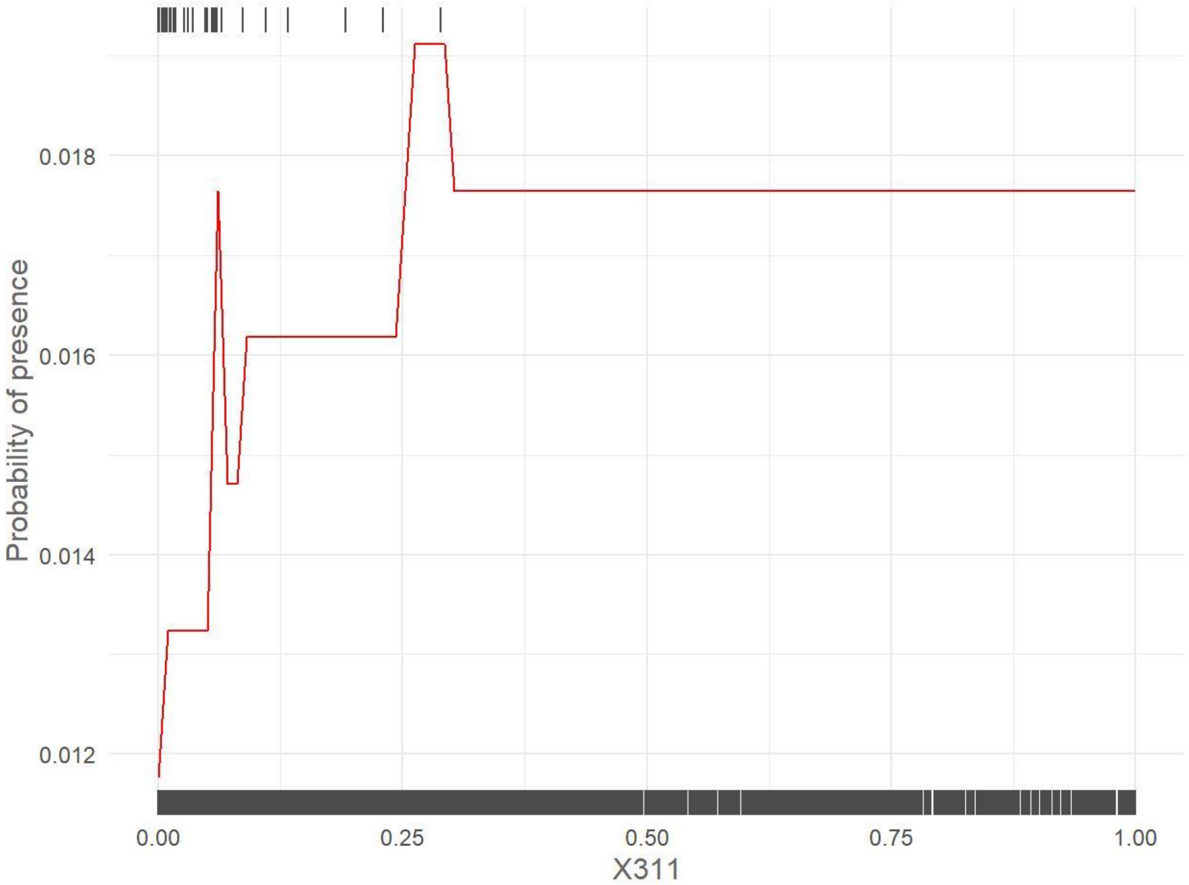
Probability of presence
0.018
0.016
0.014
0.012
0.00
0.25
0.50
0.75
1.00
X311

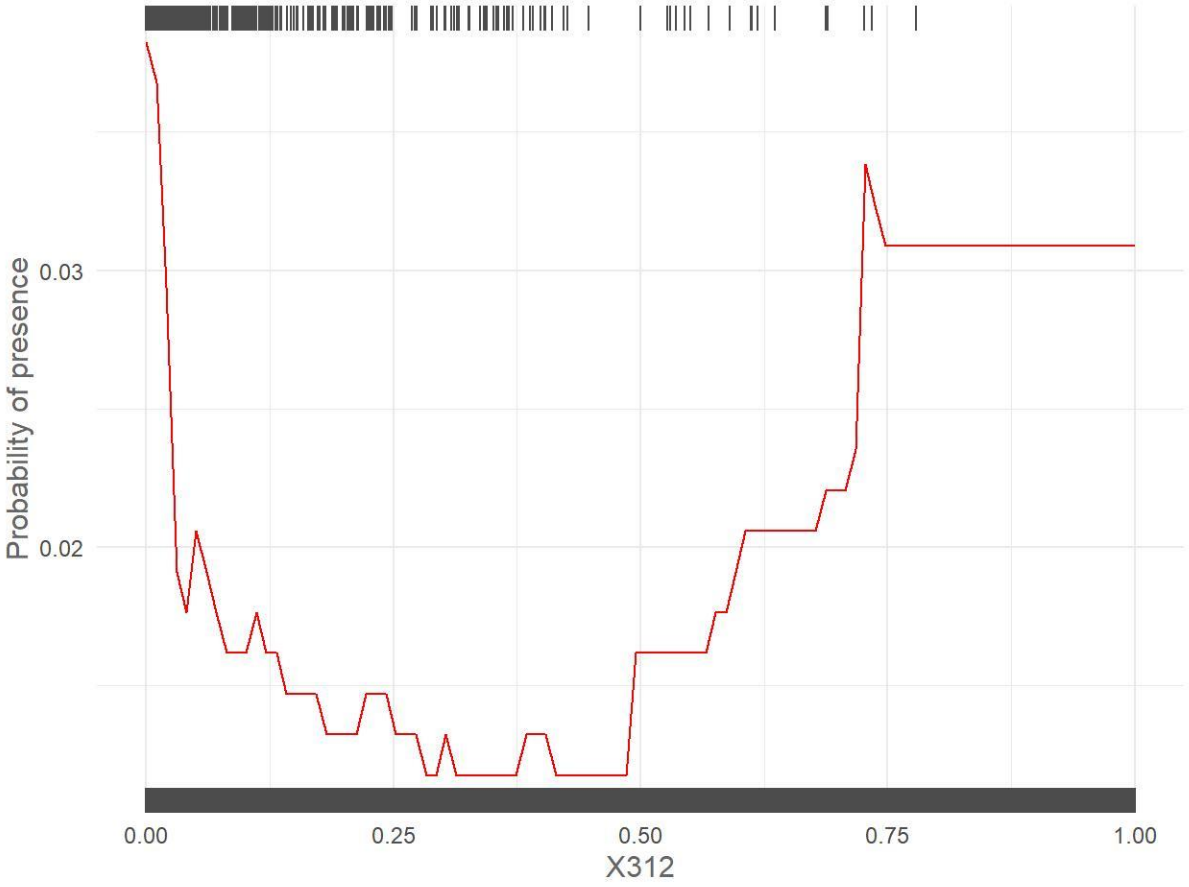
Probability of presence
0.03
0.02
0.00
0.25
0.50
0.75
1.00
X312

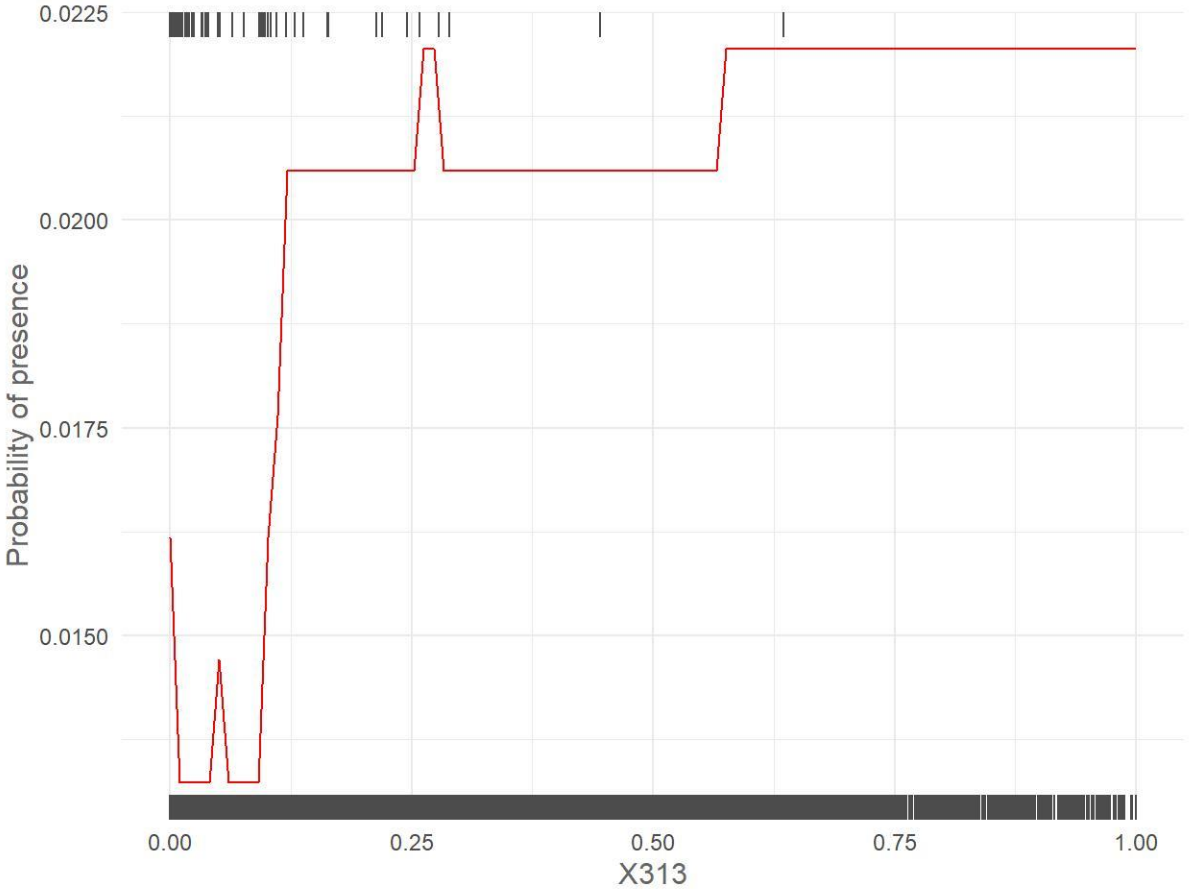
0.0225
0.0200
0.0175
0.0150
Probability of presence
0.00
0.25
0.50
0.75
1.00
X313

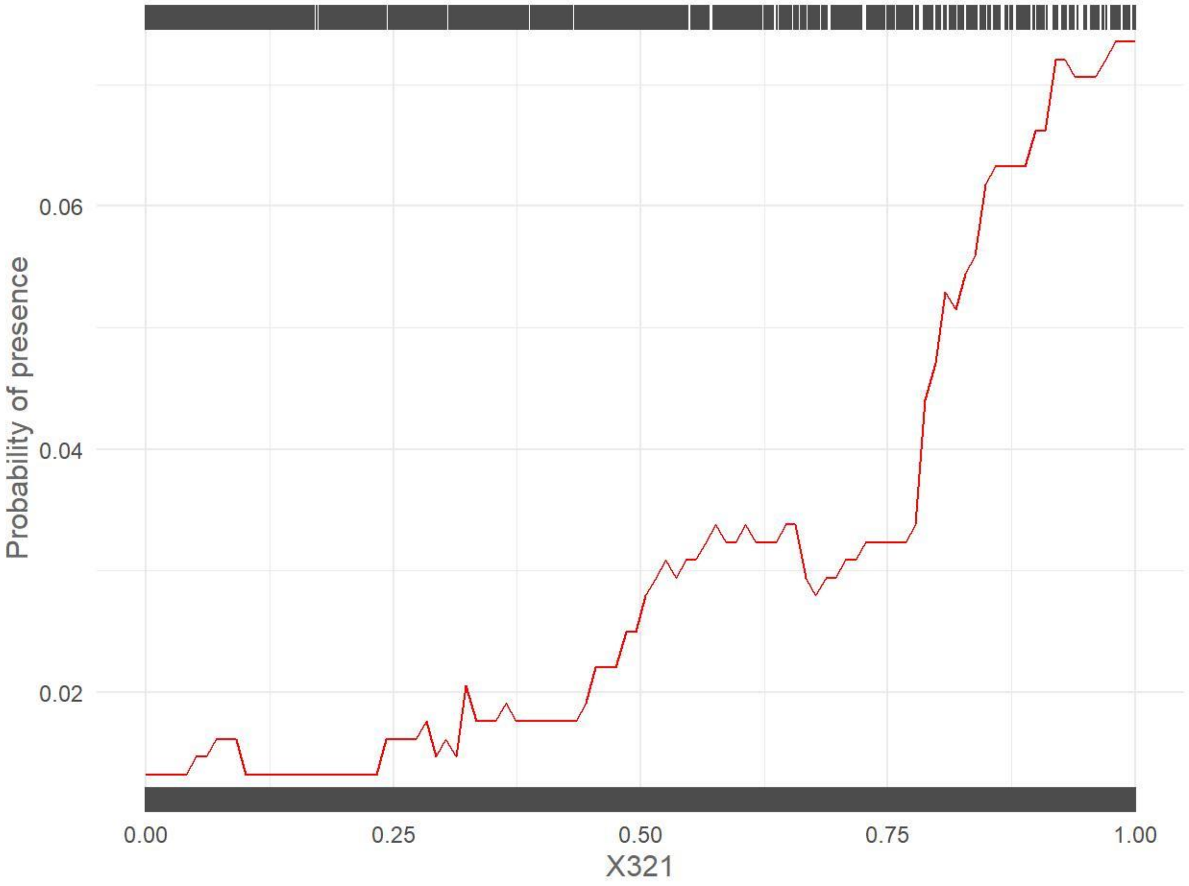
0.06
0.04
0.02
Probability of presence
0.00
0.25
0.50
0.75
1.00
X321

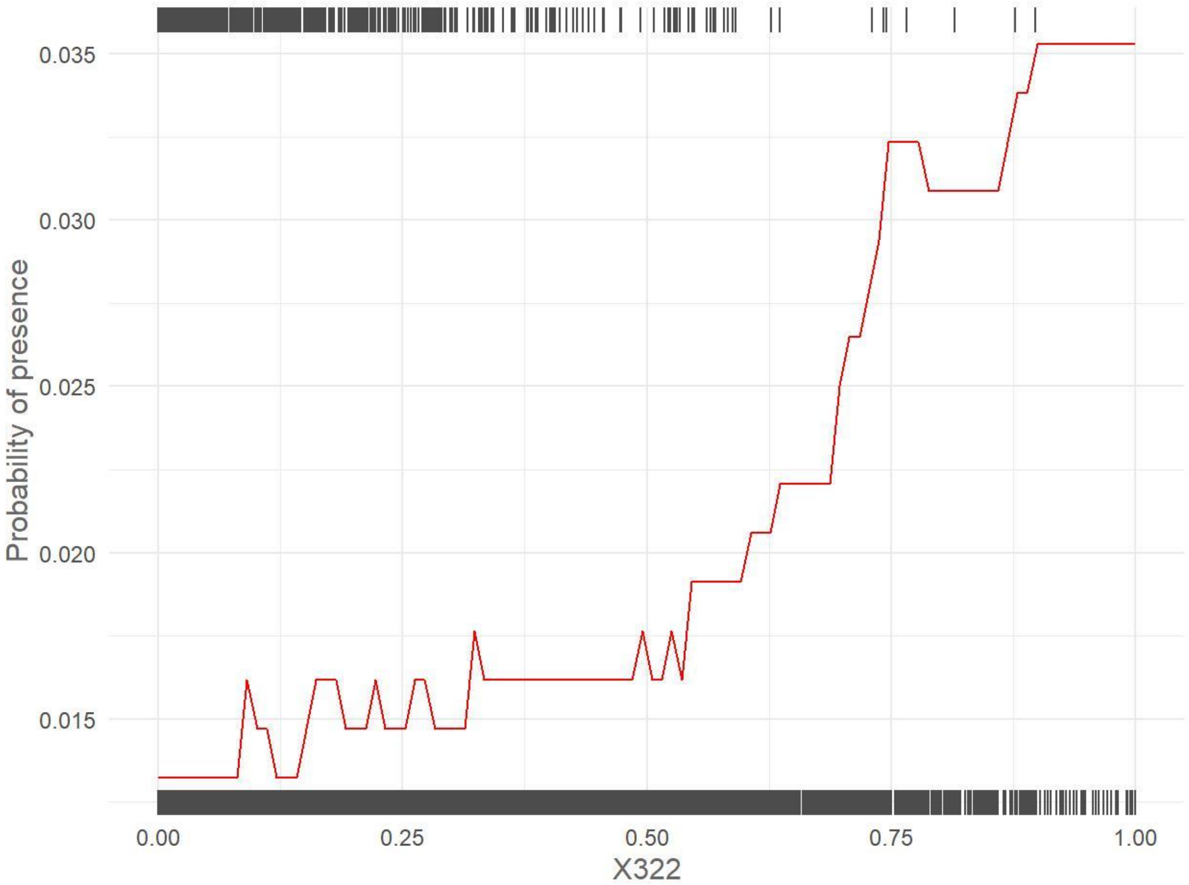

0.035
0.030
0.025
0.020
0.015
Probability of presence
0.00
0.25
0.50
0.75
1.00
X322

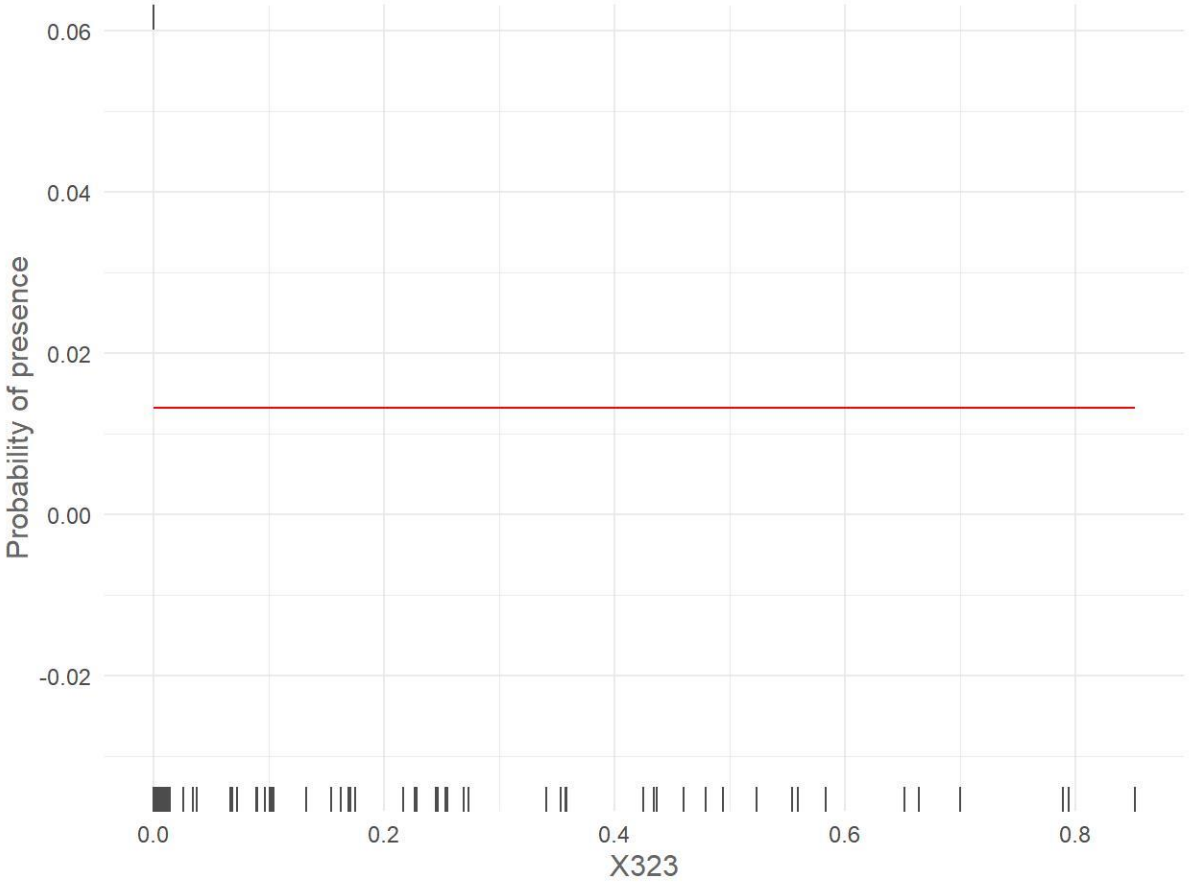

0.06
0.04
0.02
0.00
-0.02
Probability of presence
0.0
0.2
0.4
0.6
0.8
X323

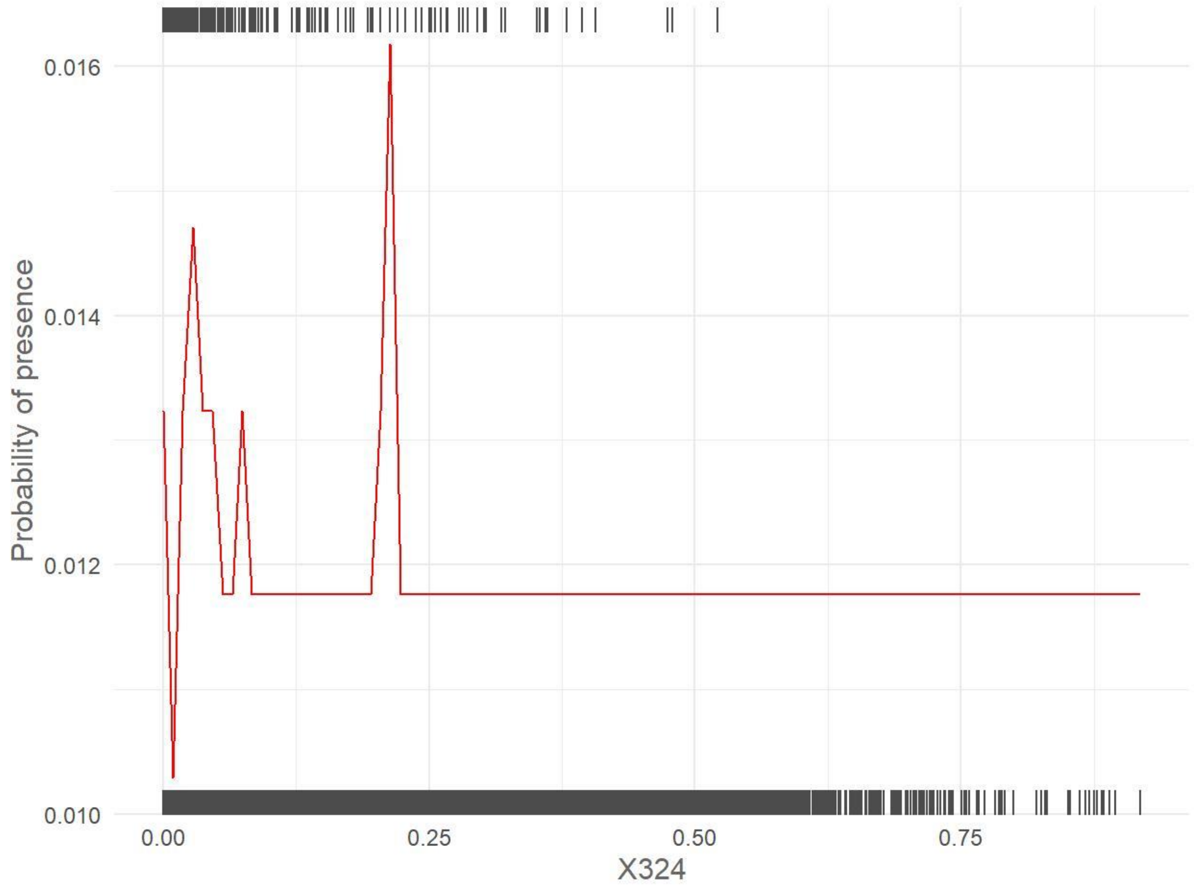

0.016
0.014
0.012
0.010
Probability of presence
0.00
0.25
0.50
0.75
X324

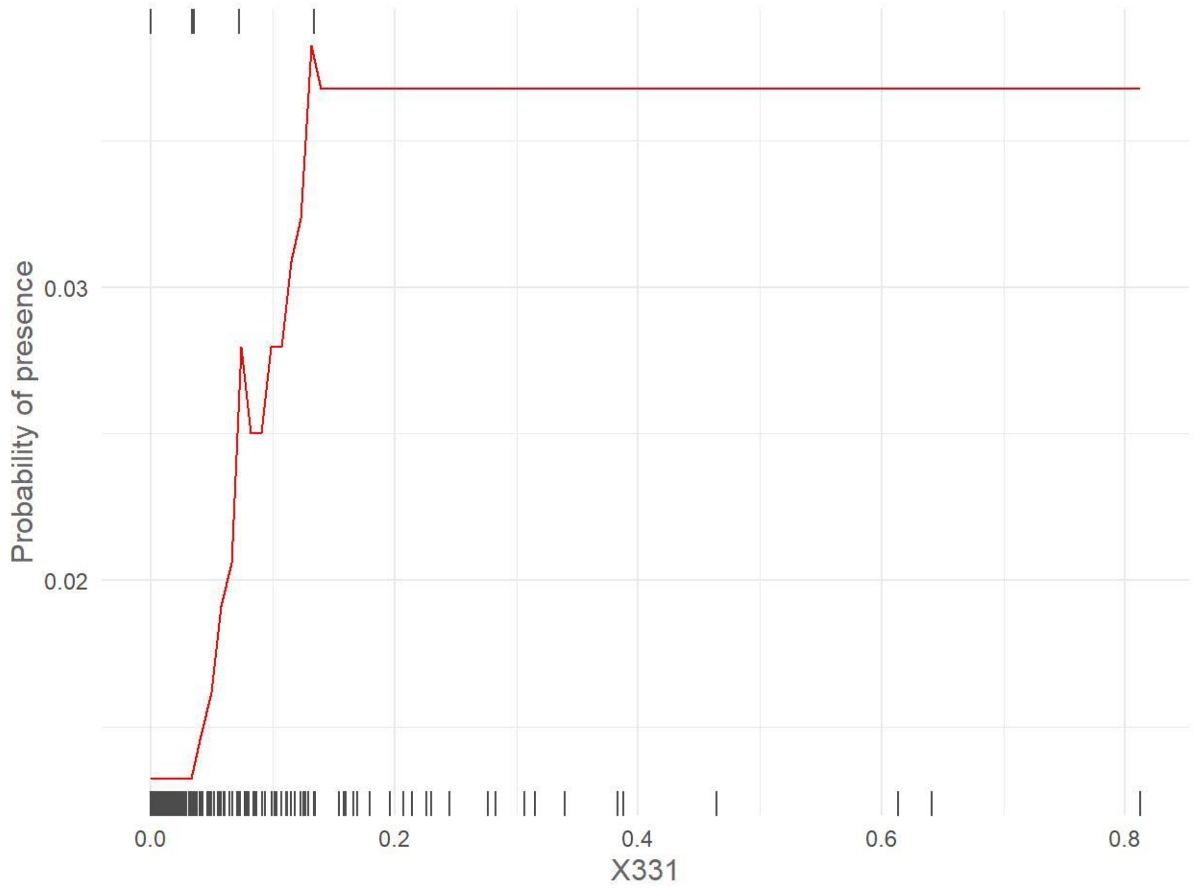

0.03
0.02
Probability of presence
0.0
0.2
0.4
0.6
0.8
X331

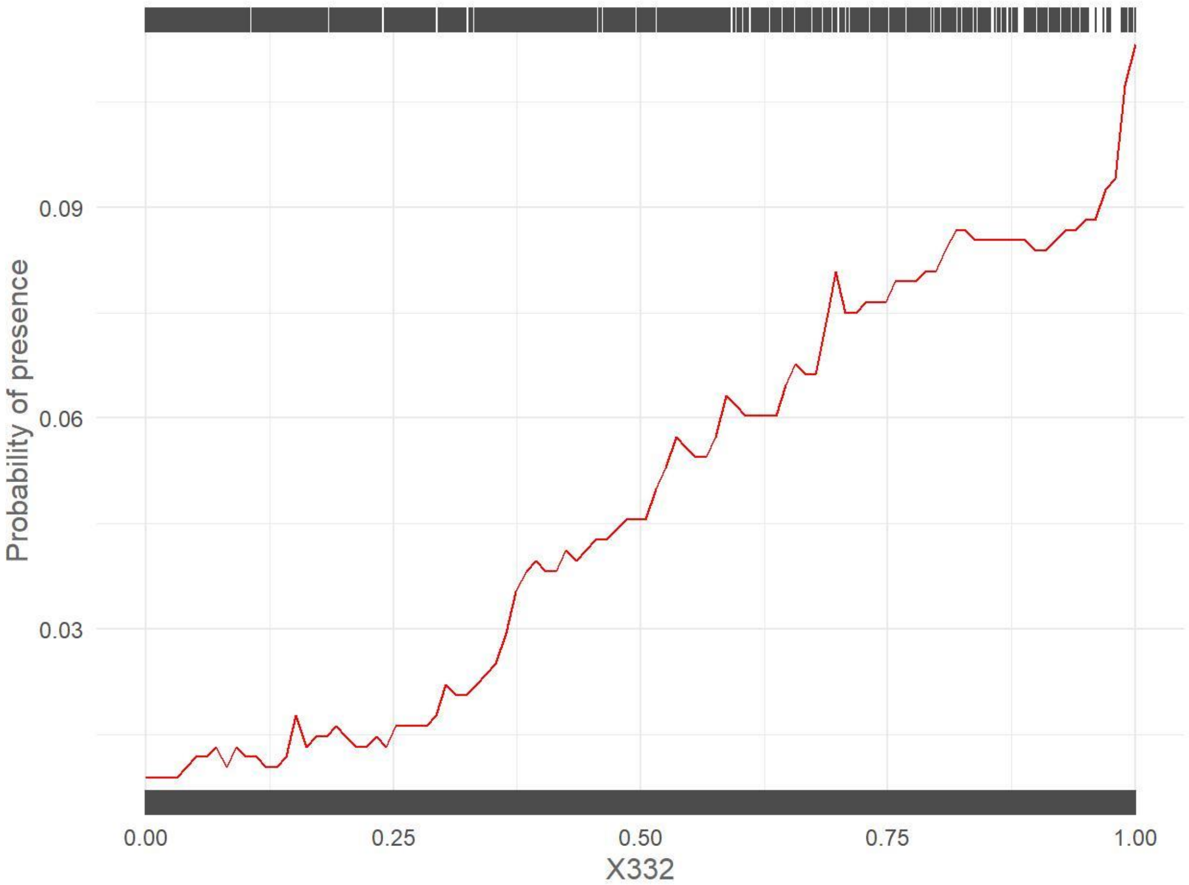

Probability of presence
0.09
0.06
0.03
0.00
0.25
0.50
0.75
1.00
X332

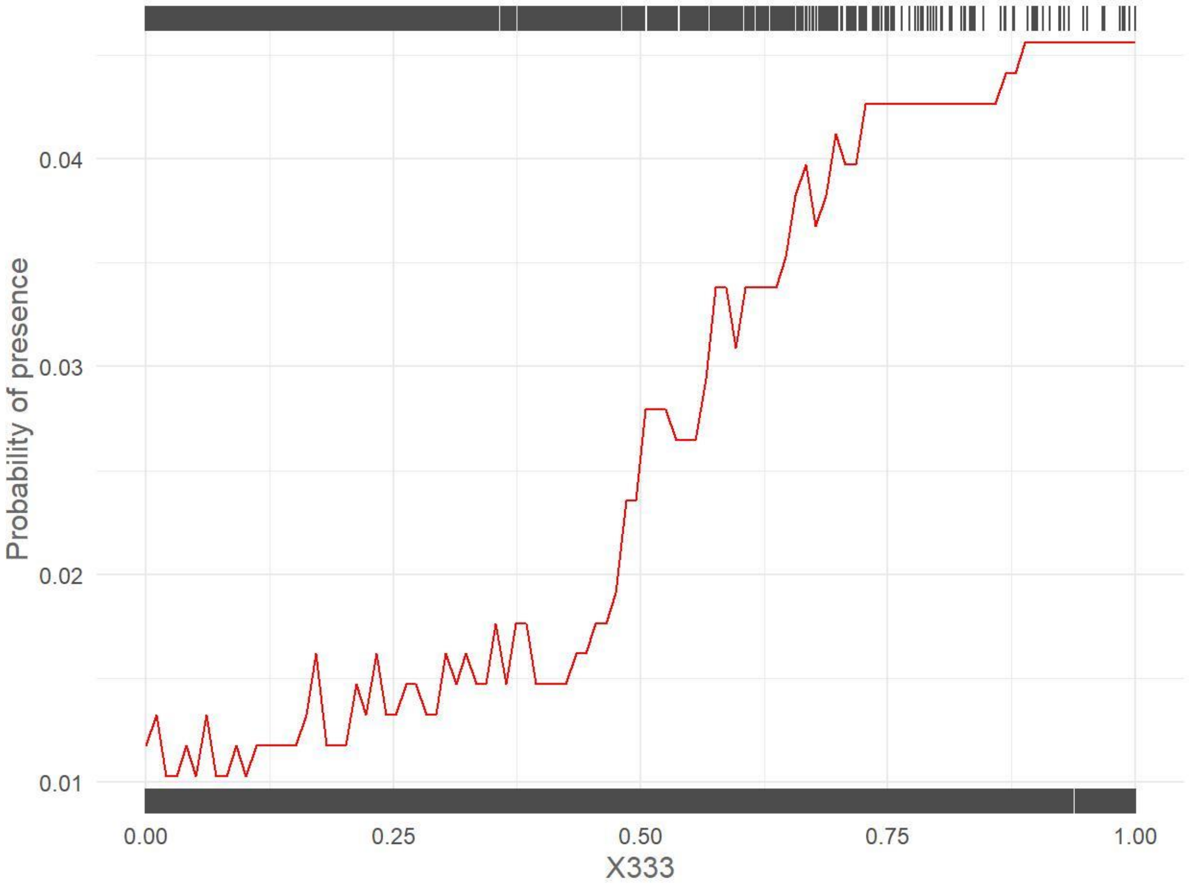

Probability of presence
0.04
0.03
0.02
0.01
0.00
0.25
0.50
0.75
1.00
X333

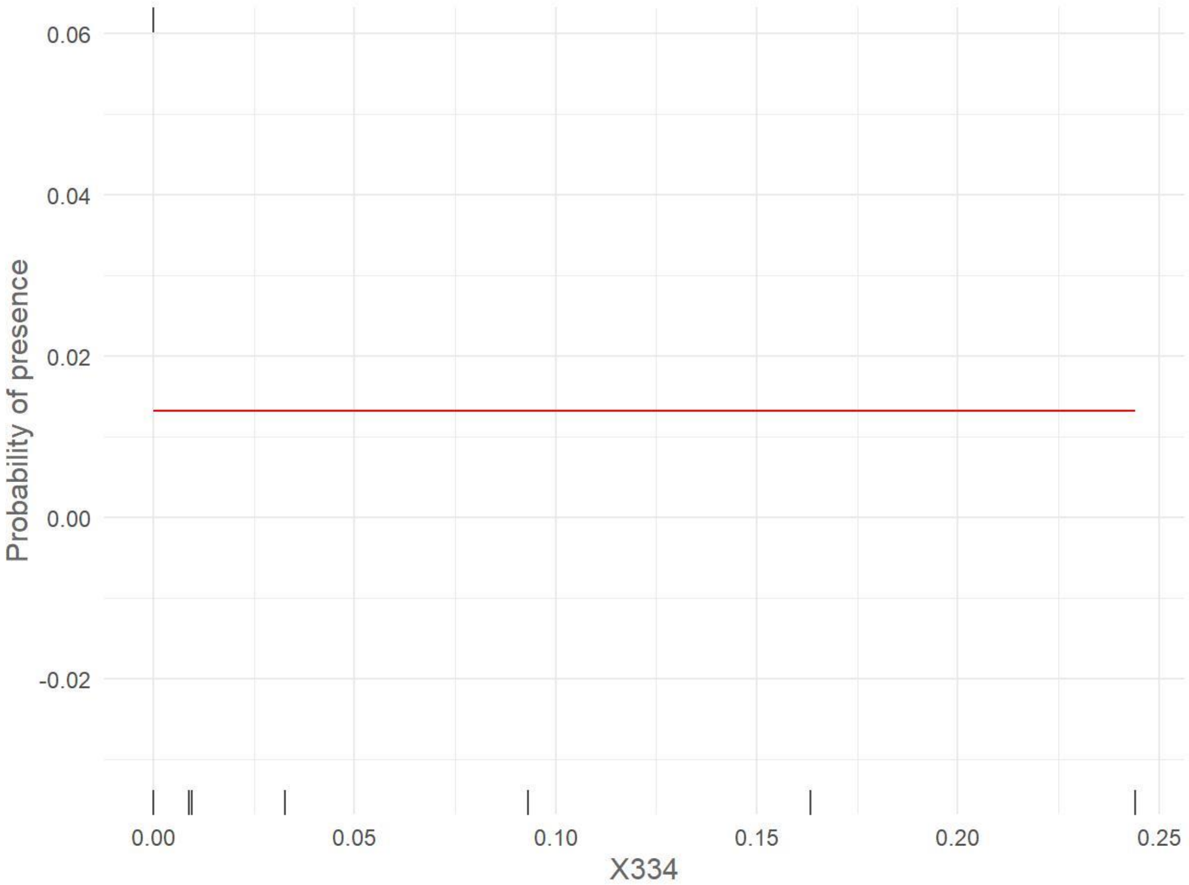
0.06
0.04
0.02
0.00
-0.02
Probability of presence
0.00
0.05
0.10
0.15
0.20
0.25
X334

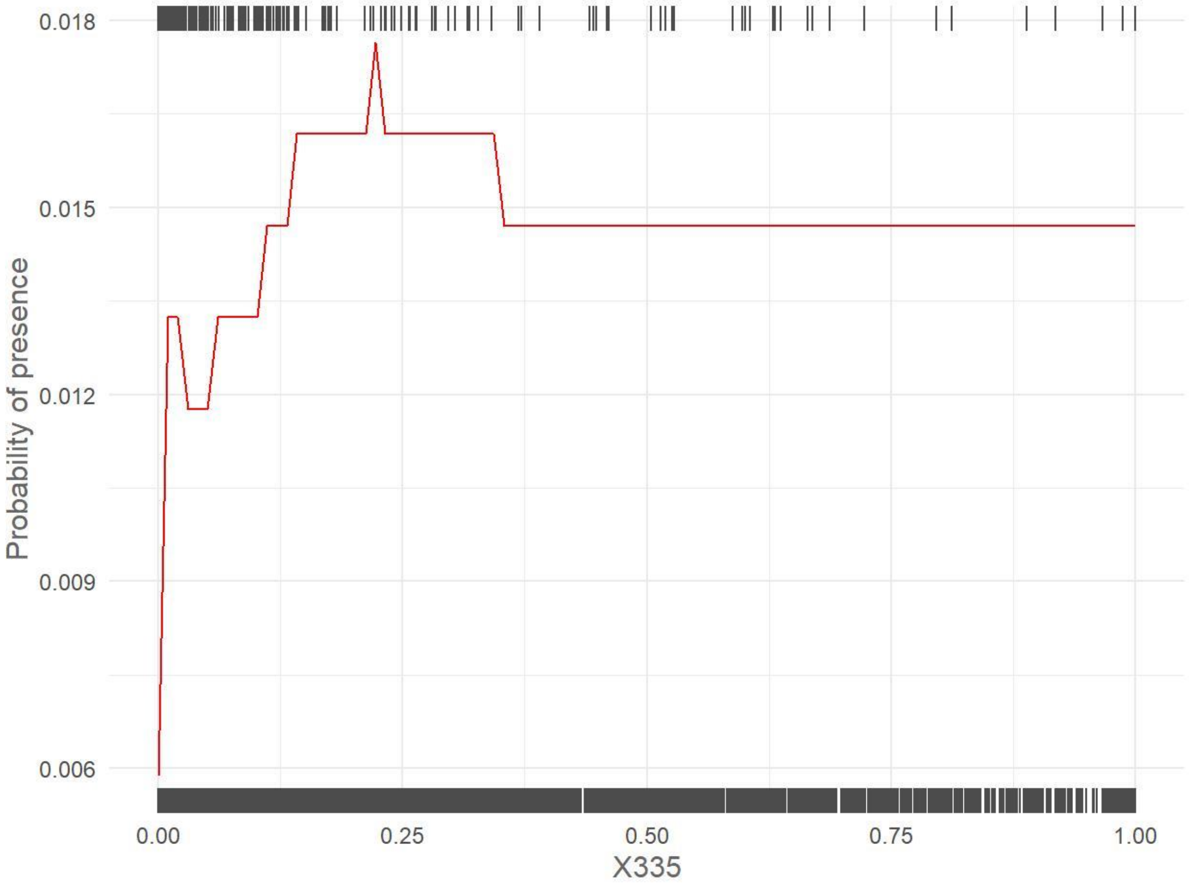
0.018
0.015
0.012
0.009
0.006
Probability of presence
0.00
0.25
0.50
0.75
1.00
X335

Appendix 2: Observed and predicted current species distribution

The observed breeding distribution of the target species (shown in blue) is derived from BirdLife International shapefile of bird distribution. The predicted habitat suitability is shown as the continuous output provided by distribution models, using the cloglog transformation for MaxEnt models, and the occurrence probability for all the other models. Note that predictions do not cover North Africa (even if it is partially displayed in the maps). In each map and for each model, the lowest values of suitability are displayed as ‘zero’ (white) to allow a better visual evaluation of the distribution of suitable sites. For all species, suitability is shown in red: the darkest the colour, the highest the suitability for the target species.

Consistency between observed and predicted distribution is estimated according to a qualitative and expert-based interpretation of the concordance between observed (taking into account recent updates provided by the new European Atlas of breeding birds, Keller et al., 2020) and predicted distribution (habitat suitability), with particular reference to areas outside the Alps, given that the main aim of this part of the work was to evaluate the reliability of the models for extrapolation purposes. We considered the validation over distant areas as the best method for such assessment of extrapolation potential.

References cited in this section:

Brambilla, M., Resano-Mayor, J., Arlettaz, R., Bettega, C., Binggeli, A., Bogliani, G., Braunisch, V., Celada, C., Chamberlain, D., Carricaburu, J.C., Delgado, M.D.M., Fontanilles, P., Kmecl, P., Korner, F., Lindner, R., Pedrini, P., Pöhacker, J., Rubinič, B., Schano, C., Scridel, D., Strinella, E., Teufelbauer, N., De Gabriel Hernando, M., 2020b. Potential distribution of a climate sensitive species, the White-winged Snowfinch *Montifringilla nivalis* in Europe. Bird Conserv. Int. 30, 522–532. https://doi.org/10.1017/S0959270920000027

Keller, V., Herrando, S., Voříšek, P., Franch, M., Kipson, M., Milanesi, P., Martí, D., Anton, M., Klvaňová, A., Kalyakin, M. V., Bauer, H.-G., P.B. Foppen, R., 2020. European Breeding Bird Atlas 2: Distribution, Abundance and Change. European Bird Census Council & Lynx Edicions, Barcelona.

MaxEnt models

Figure S17. Current observed (blue polygons; according to BirdLife International shapefile) and predicted habitat suitability according to the MaxEnt model for rock ptarmigan. Almost perfect consistency between the two.

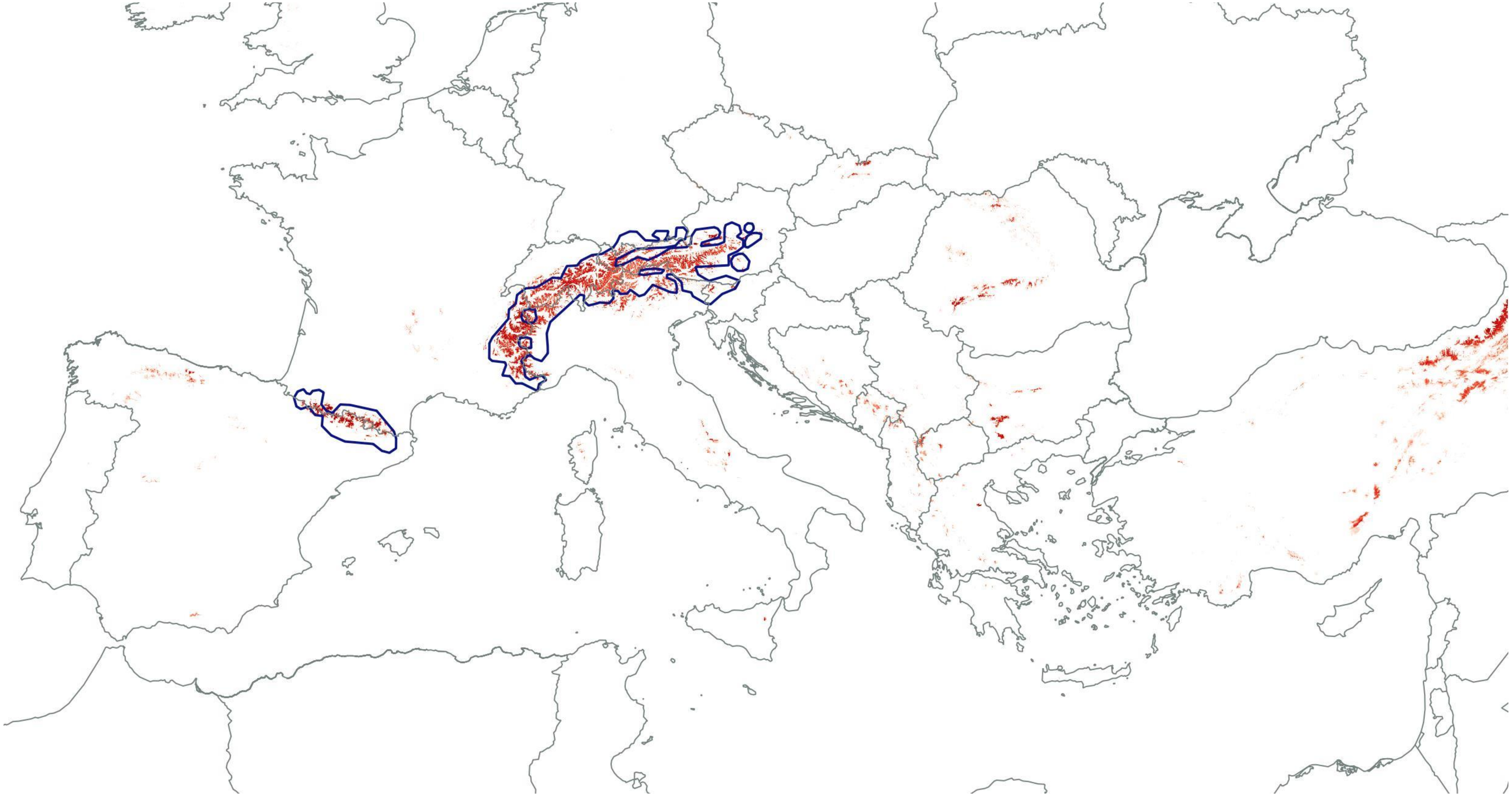

Figure S18. Current observed (blue polygons; according to BirdLife International shapefile) and predicted habitat suitability according to the MaxEnt model for water pipit. The distribution in Sardinia (a single small patch, not visible in the current map) and central-southern Italy is much more restricted than suggested by BirdLife shapefile and actually mirrors quite well the model (Keller et al., 2020). Most of occupied areas according to BirdLife shapefile include suitable patches.

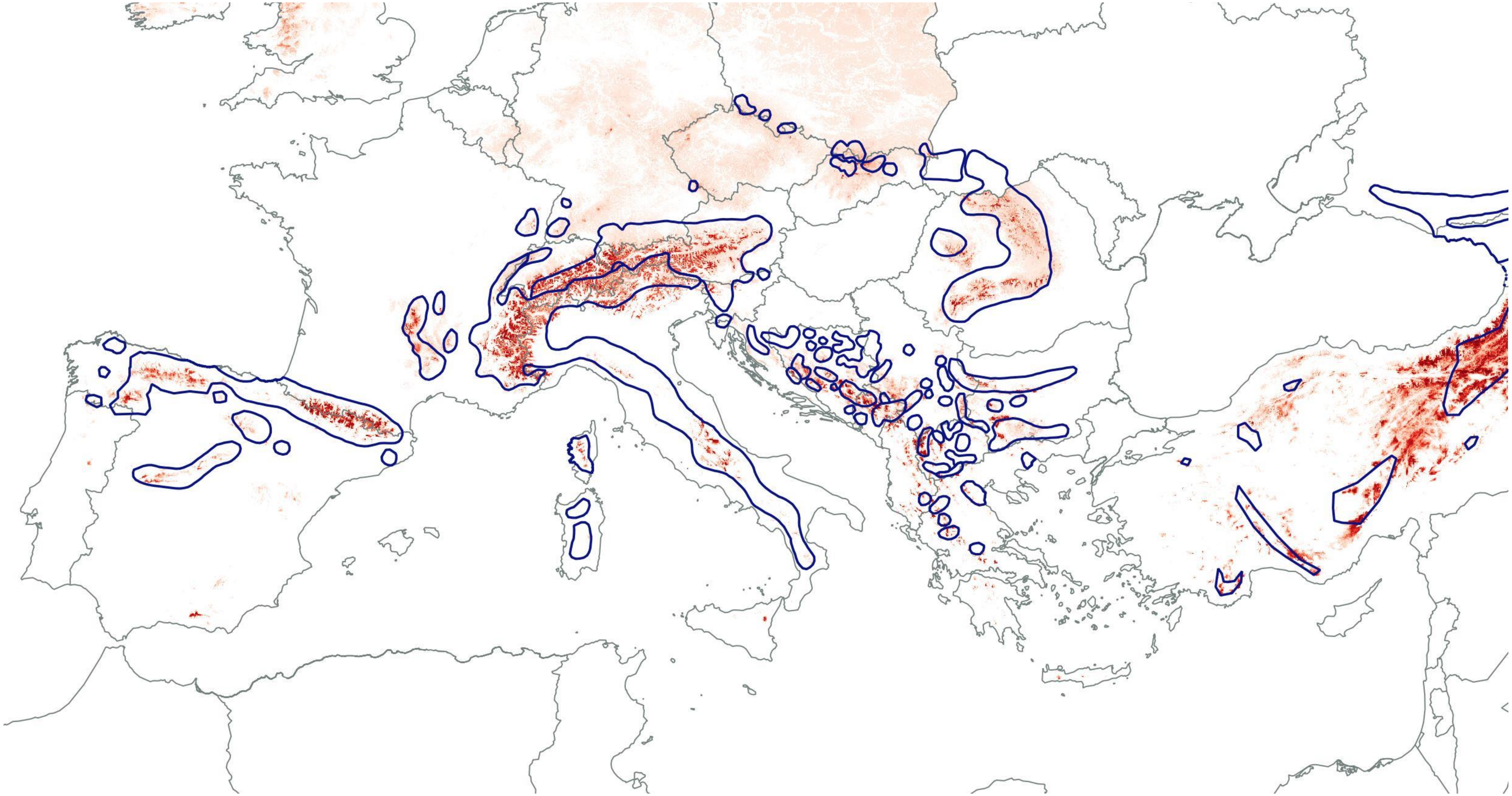

Figure S19. Current observed (blue polygons; according to BirdLife International shapefile) and predicted habitat suitability according to the MaxEnt model for alpine accentor. Generally high consistency between the two; some discrepancies might be due to coarse resolution of the shapefile (occupied areas close to suitable patches but not including them, e.g. central France, Corsica, southern and central Spain; cf. Keller et al., 2020).

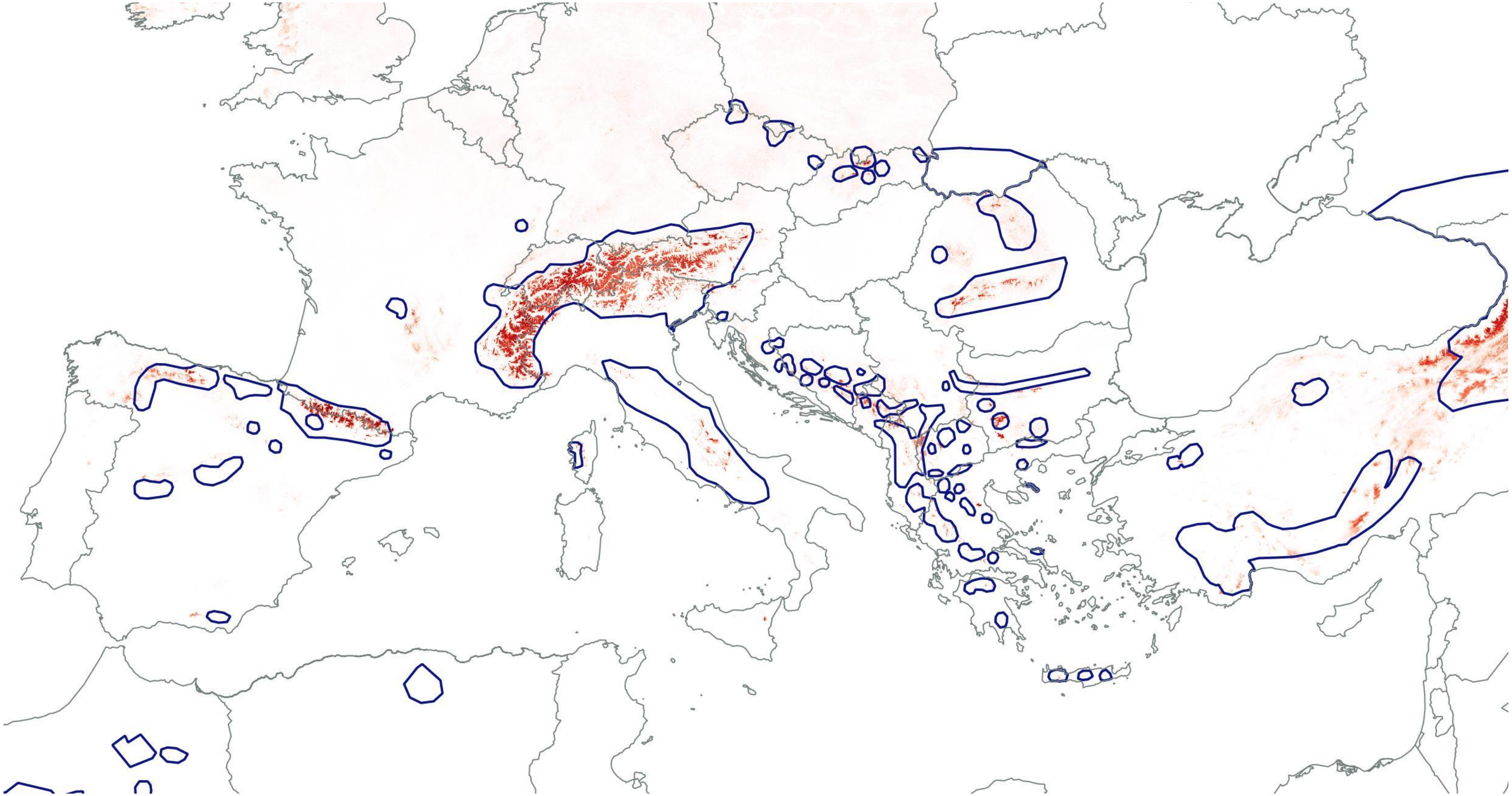

Figure S20. Current observed (blue polygons; according to BirdLife International shapefile) and predicted habitat suitability according to the MaxEnt model for white-winged snowfinch. Main inconsistencies are found in eastern Europe and are likely to be due at least partially to imprecise drawing of BirdLife shapefile (Brambilla et al., 2020; Keller et al., 2020). All other occurrence areas (even the more isolated ones) host suitable patches (even if hardly/not visible at this scale in Cantabrian Mountains and Corsica).

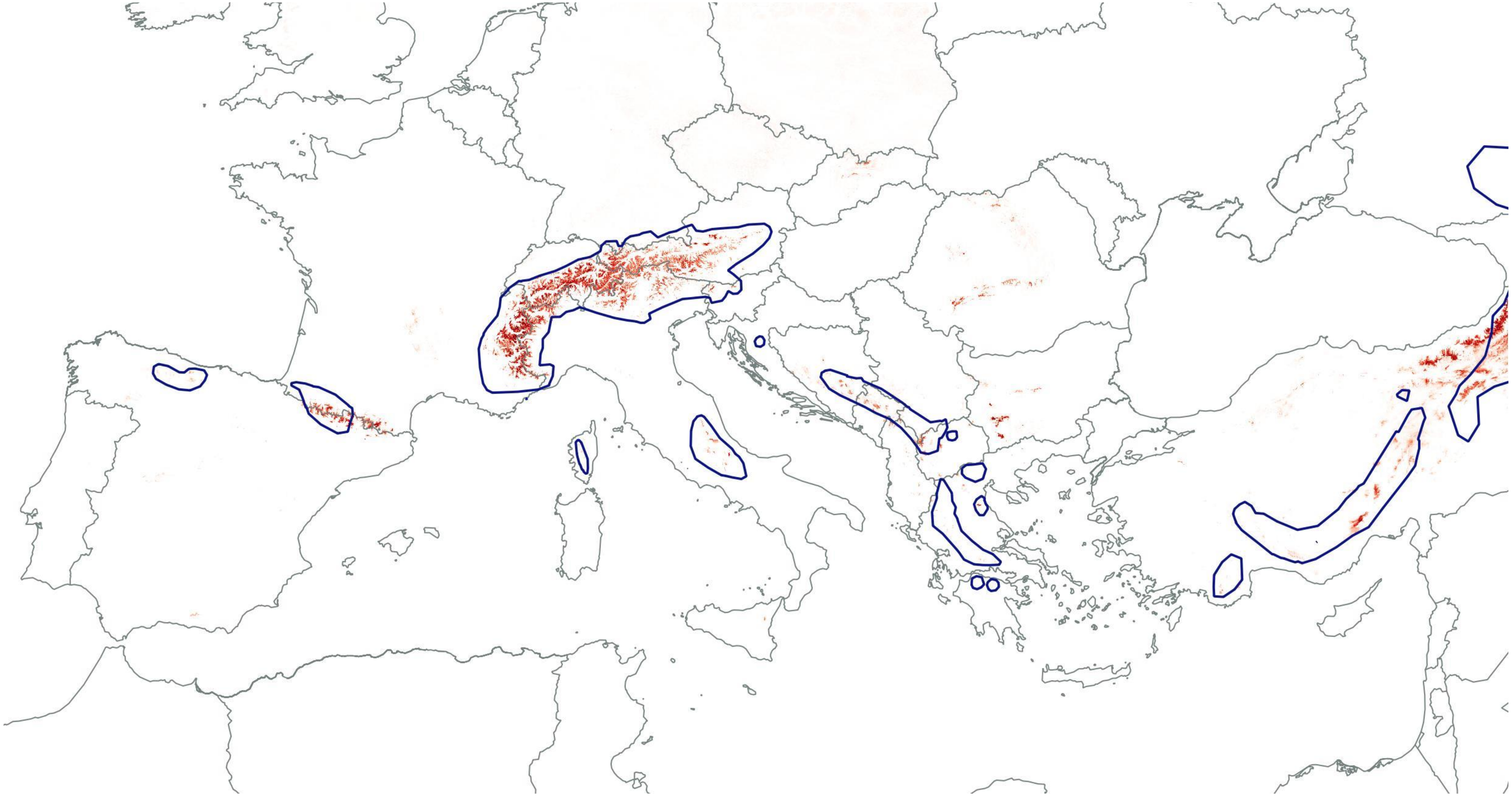

Artificial Neural Network models

Figure S21. Current observed (blue polygons; according to BirdLife International shapefile) and predicted habitat suitability according to the Artificial Neural Network model for rock ptarmigan. Almost perfect concordance.

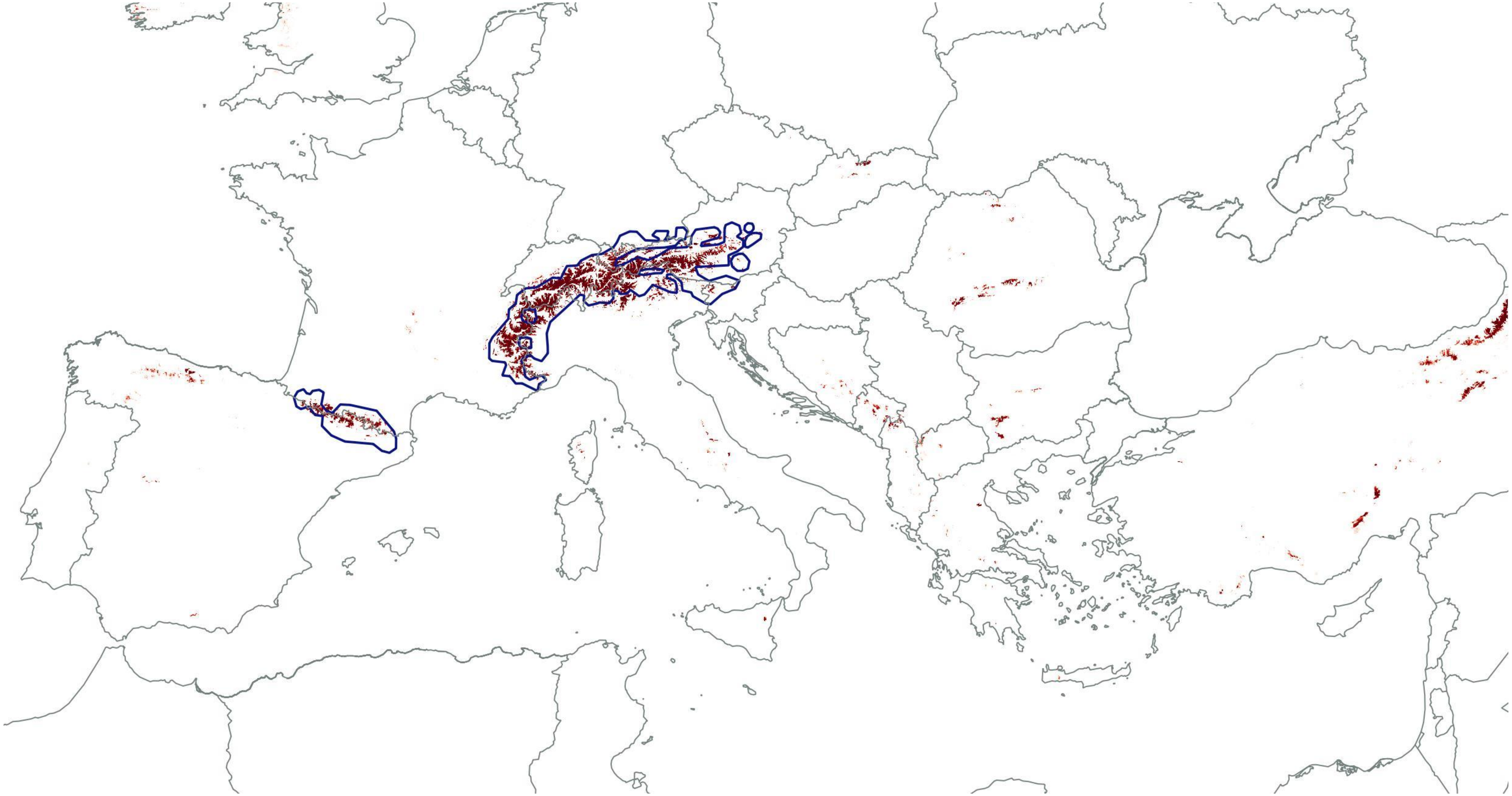

Figure S22. Current observed (blue polygons; according to BirdLife International shapefile) and predicted habitat suitability according to the Artificial Neural Network model for water pipit. The modelled range is slightly underestimated but the consistency is still high.

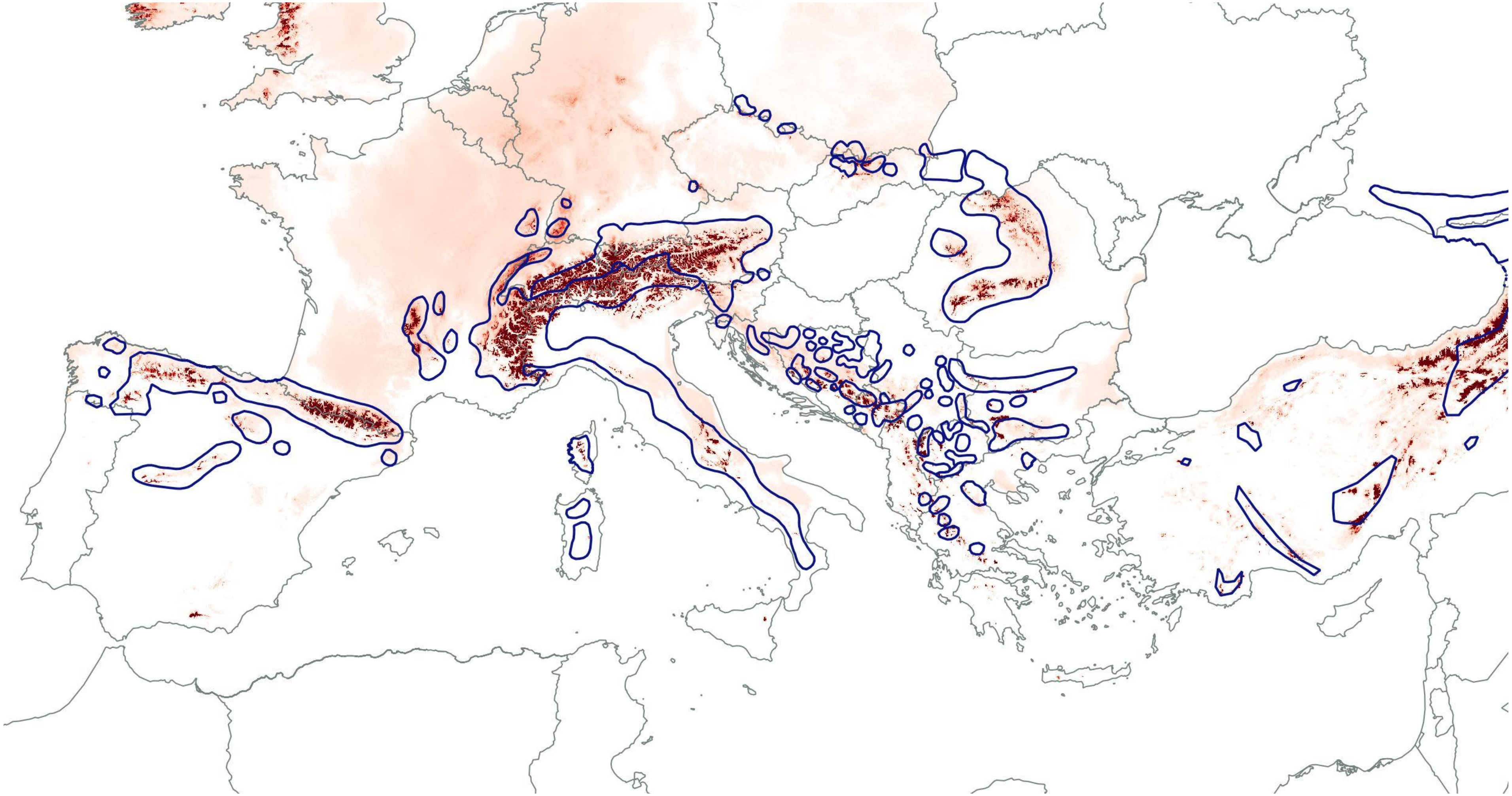

Figure S23. Current observed (blue polygons; according to BirdLife International shapefile) and predicted habitat suitability according to the Artificial Neural Network model for alpine accentor. The modelled range is only slightly underestimated.

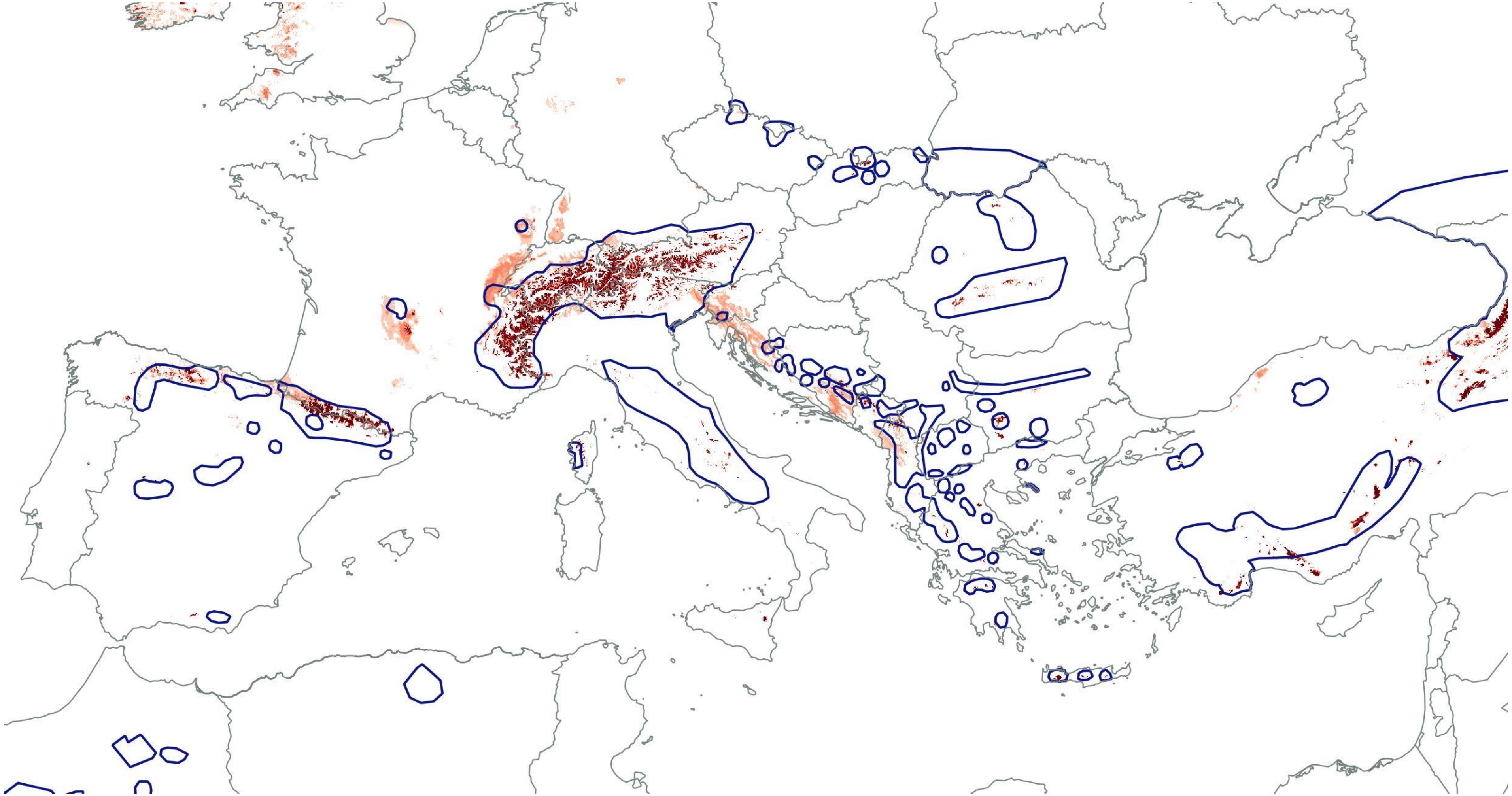

Figure S24. Current observed (blue polygons; according to BirdLife International shapefile) and predicted habitat suitability according to the Artificial Neural Network model for white-winged snowfinch. The modelled range is slightly underestimated.

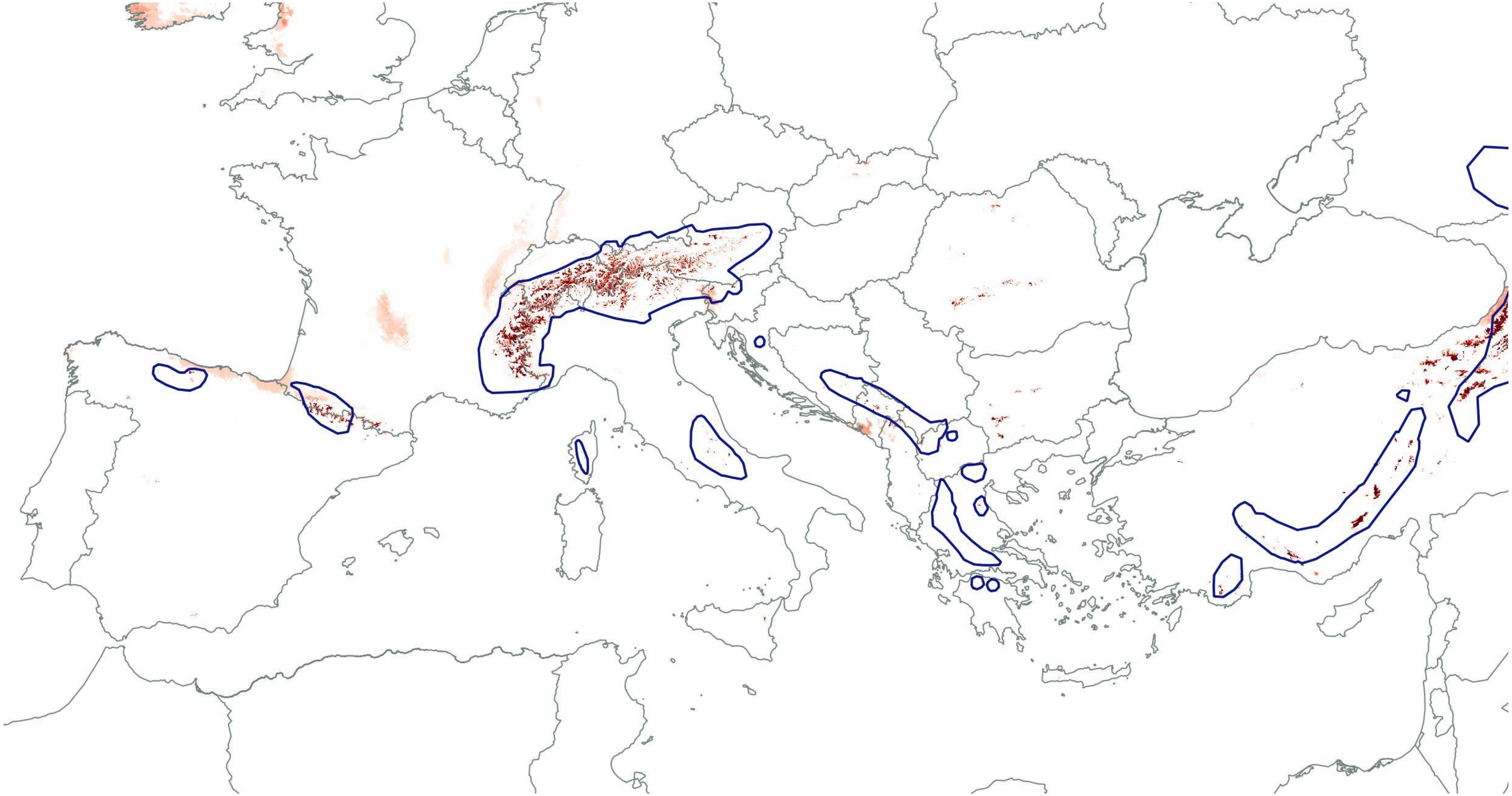

Boosted Regression Trees

Figure S25. Current observed (blue polygons; according to BirdLife International shapefile) and predicted habitat suitability according to the Boosted Regression Trees model for rock ptarmigan. There is a good concordance in general.

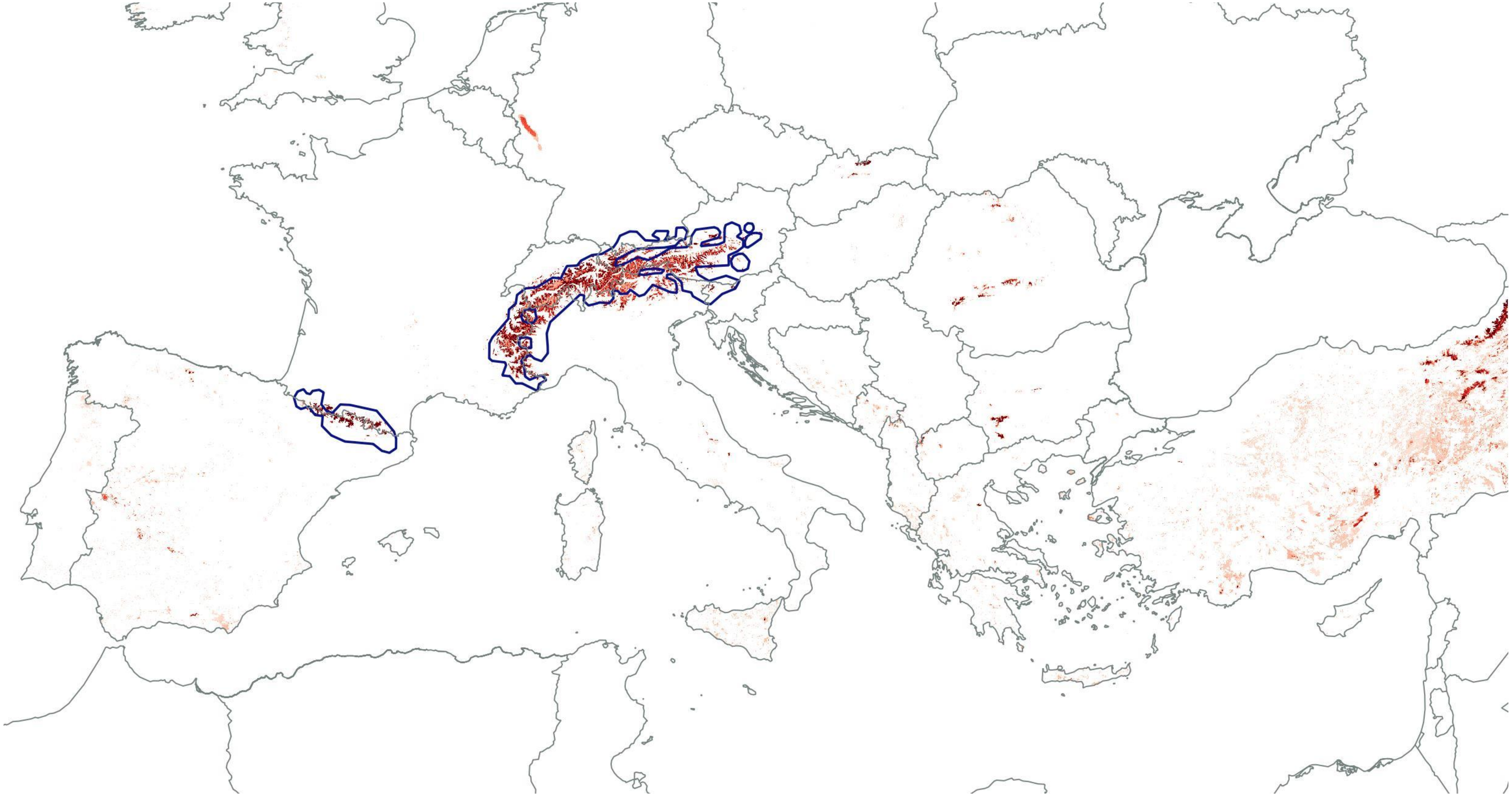

Figure S26. Current observed (blue polygons; according to BirdLife International shapefile) and predicted habitat suitability according to the Boosted Regression Trees model for water pipit. The modelled suitability is overestimated outside the true distribution of the species, while it is slightly underestimated within some occurrence areas.

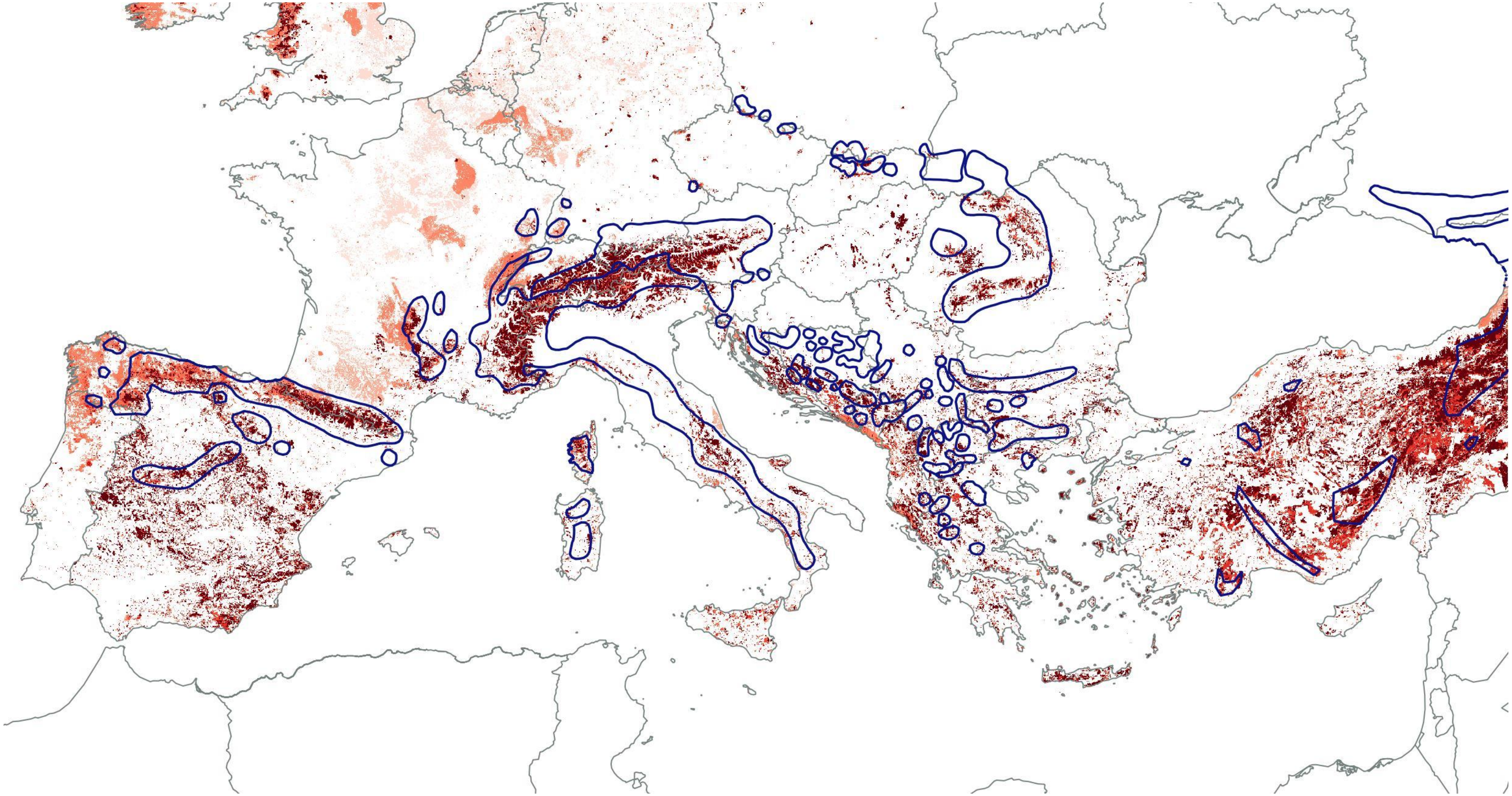

Figure S27. Current observed (blue polygons; according to BirdLife International shapefile) and predicted habitat suitability according to the Boosted Regression Trees model for alpine accentor. The modelled range is quite underestimated. Several occupied areas do not include suitable patches.

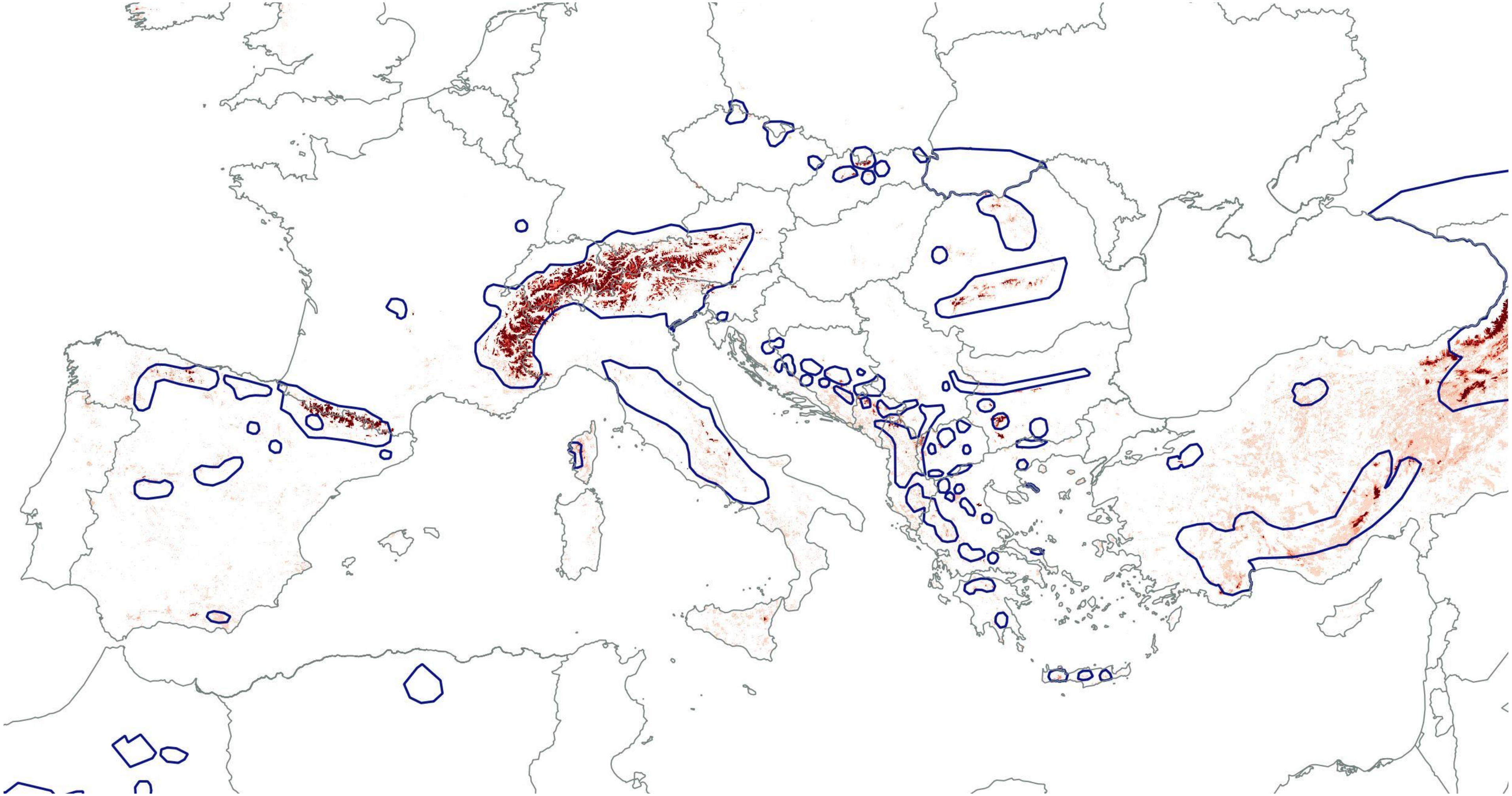

Figure S28. Current observed (blue polygons; according to BirdLife International shapefile) and predicted habitat suitability according to the Boosted Regression Trees model for white-winged snowfinch. The modelled range is slightly underestimated, even if suitable patches occur in nearly all the confirmed occurrence areas.

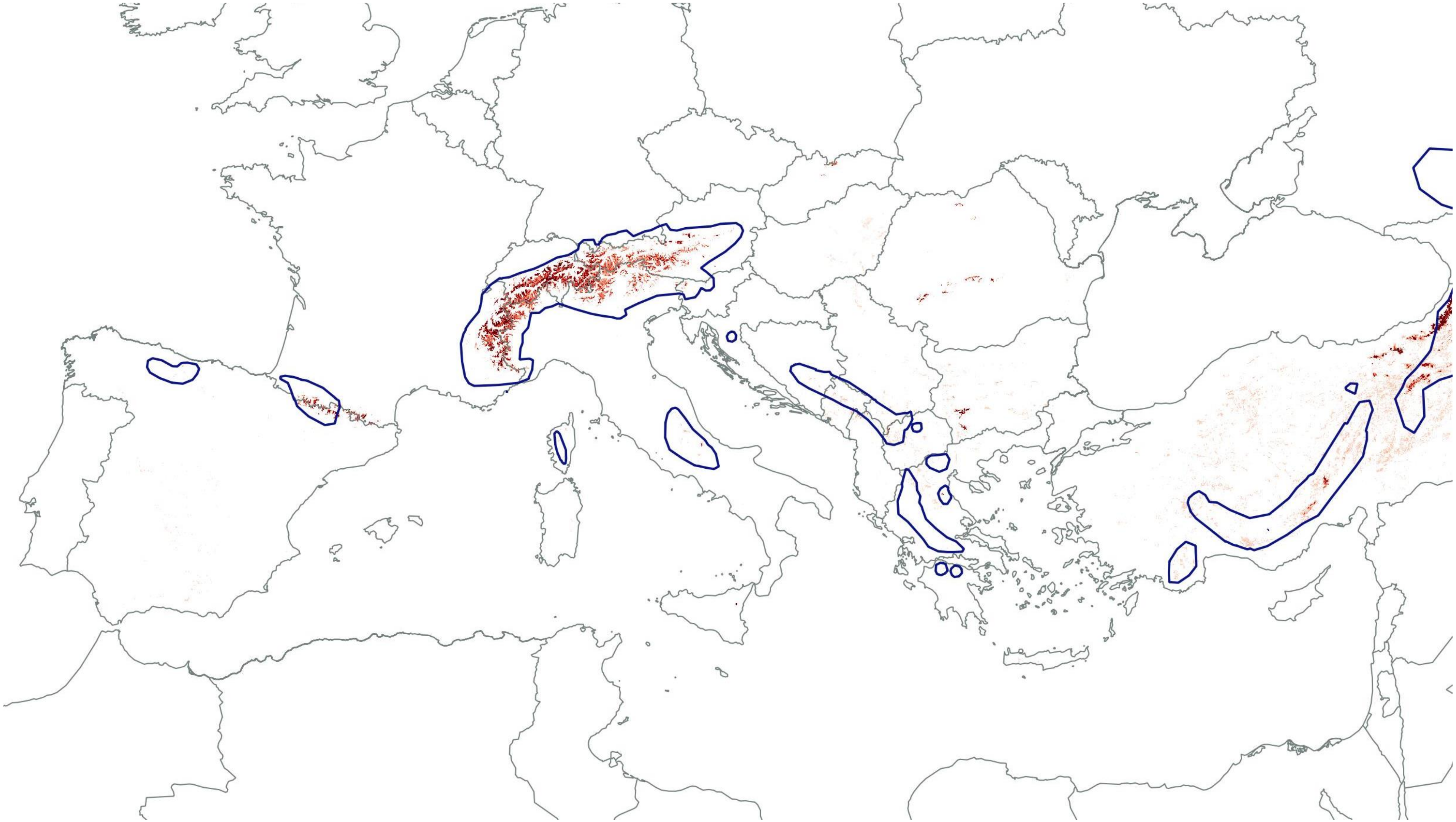

Random Forest models

Figure S29. Current observed (blue polygons; according to BirdLife International shapefile) and predicted habitat suitability according to the Random Forest model for rock ptarmigan. The modelled suitability outside the Alps is overestimated (likely also because of clamping).

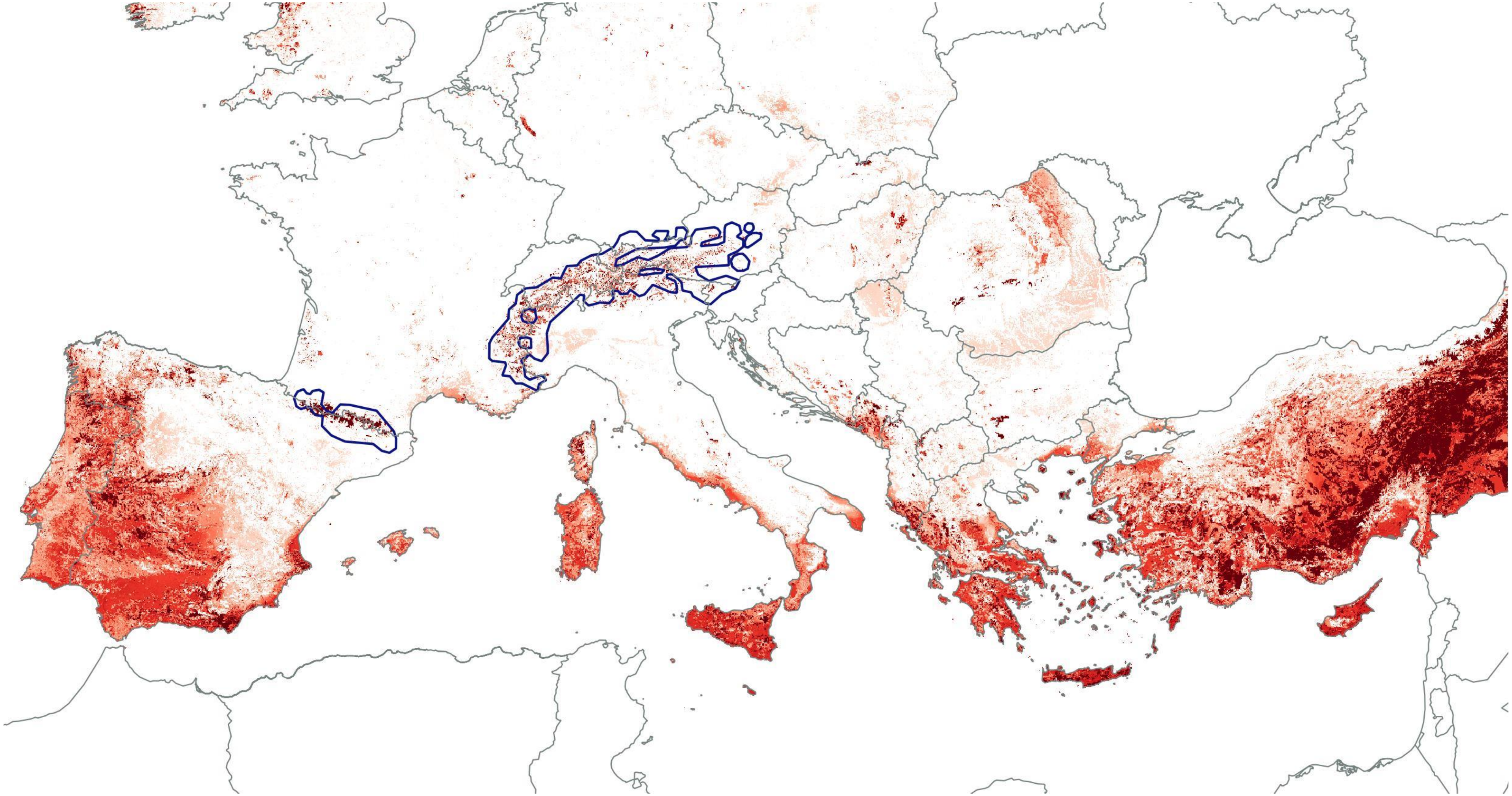

Figure S30. Current observed (blue polygons; according to BirdLife International shapefile) and predicted habitat suitability according to the Random Forest model for water pipit. The modelled range outside the Alps is dramatically overestimated, especially along coasts.

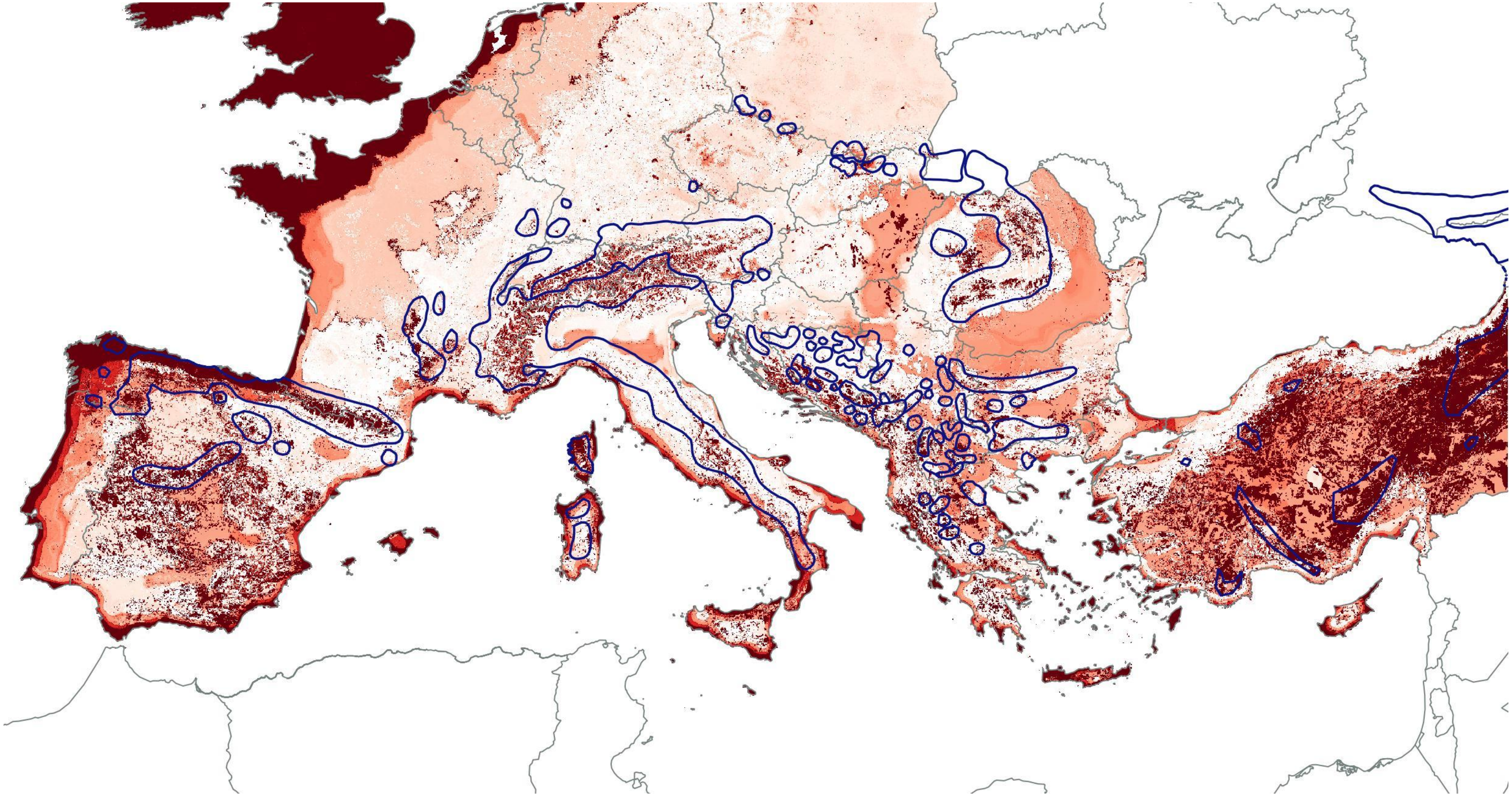

Figure S31. Current observed (blue polygons; according to BirdLife International shapefile) and predicted habitat suitability according to the Random Forest model for alpine accentor. The distribution of suitable areas is likely correct for the Alps but rather meaningless outside that area.

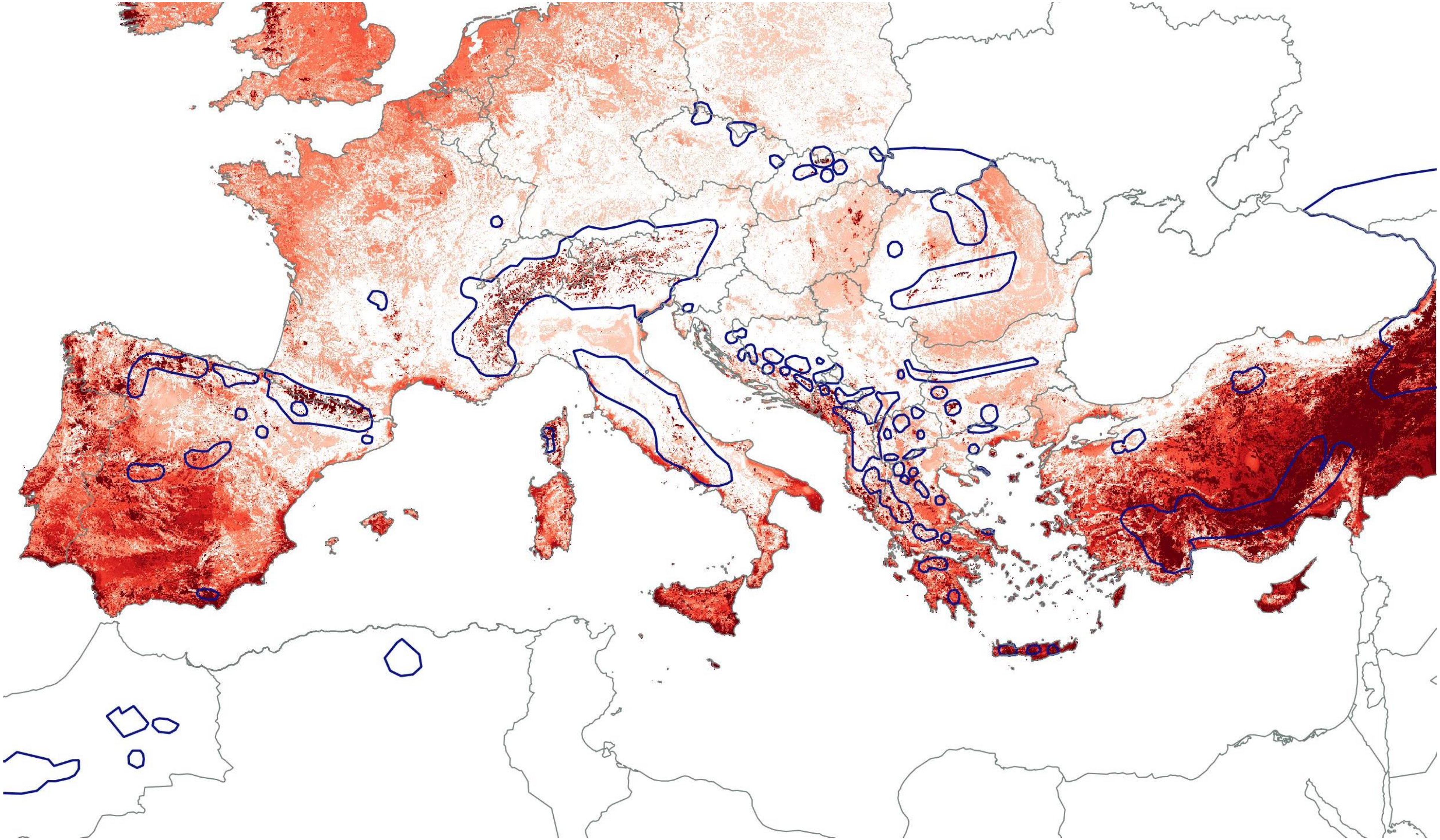

Figure S32. Current observed (blue polygons; according to BirdLife International shapefile) and predicted habitat suitability according to the Random Forest model for white-winged snowfinch. The model works well within the Alps and the Pyrenees, but predictions outside those areas are quite rather meaningless.

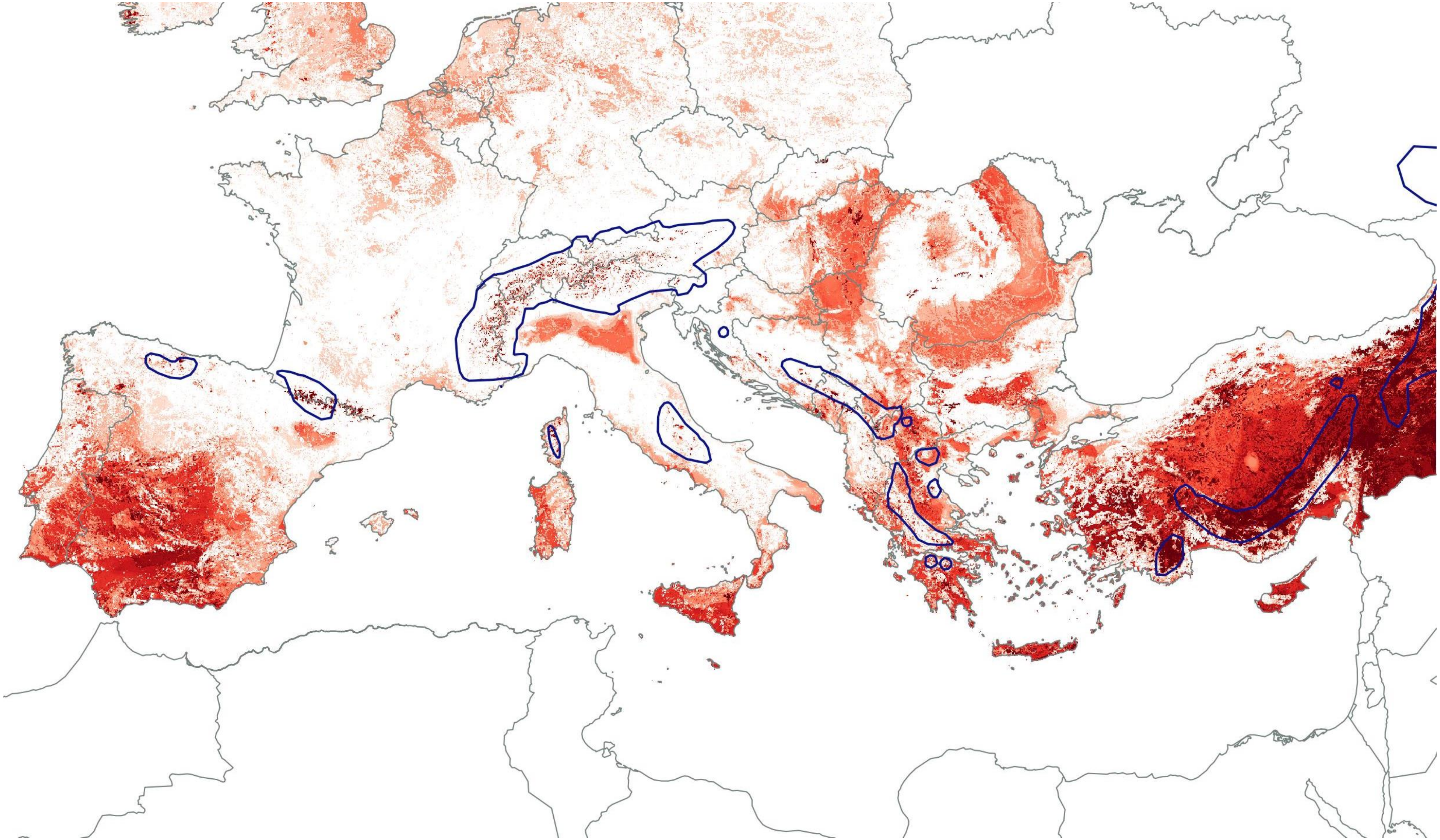

Figure S33. Distribution of type 2 refugia suitable for at least three species and comparison with type 1 refugia suitable for at least three species.

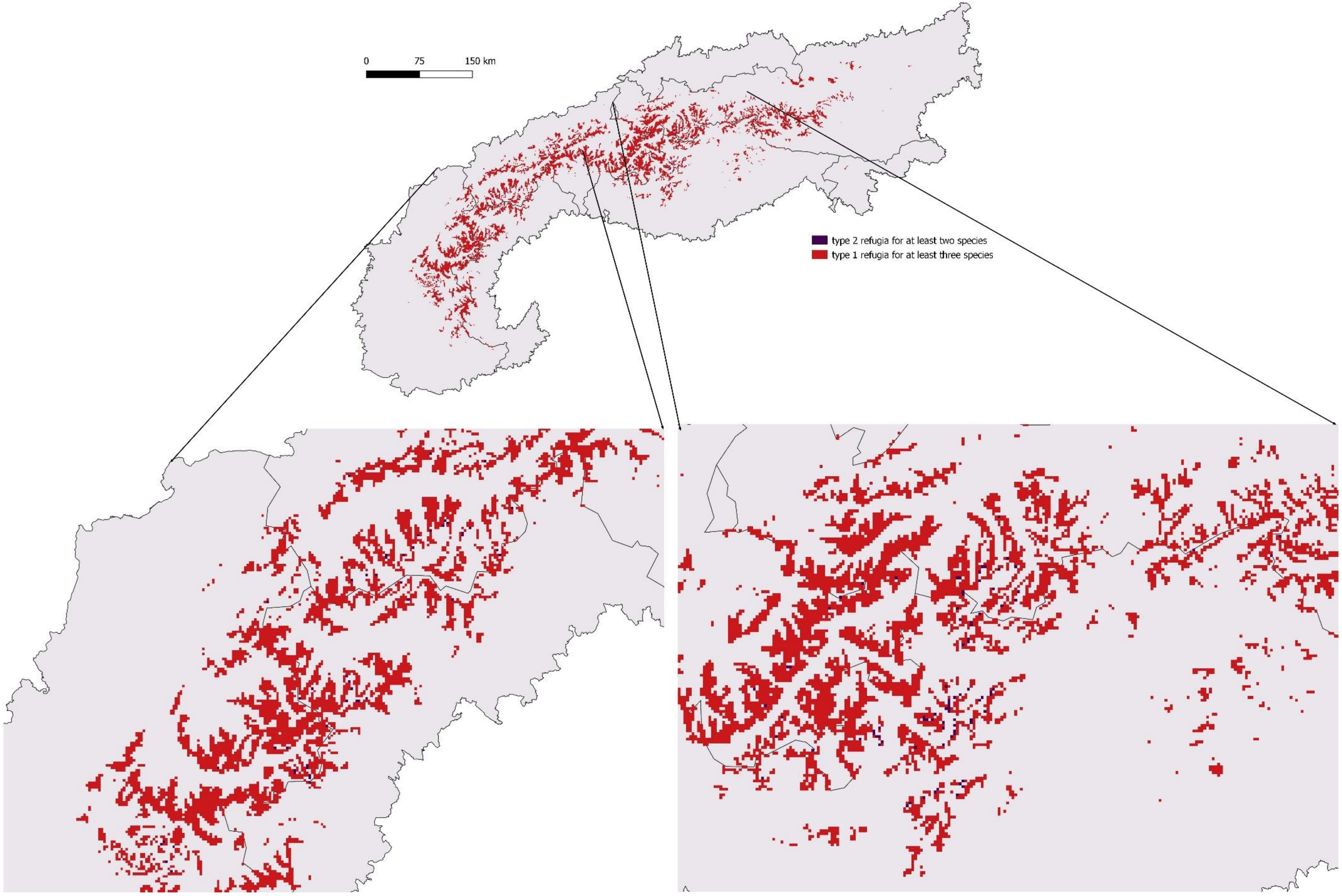

Supplement: Supplementary file 1 — Supplementary Material [file GCB-28-4276-s001.zip › gcb16187-sup-0001-AppendixS1-2.pdf]
