# Supplementary material for: Identifying climate refugia for high‐elevation Alpine birds under current climate warming predictions
Source: Glob Chang Biol. 2022 Apr 20;28(14):4276–91. doi: 10.1111/gcb.16187 (PMC9546033; doi:10.1111/gcb.16187)
Supplement: Supplementary file 1 — Supplementary Material [file GCB-28-4276-s001.zip › gcb16187-sup-0002-Appendix3.pdf]

Appendix S3

Predicted distributions of target species under different future conditions.

Maps showing current and alternative future distribution of environmentally suitable sites for each target species (as suitable areas are considered all the cells with environmental suitability higher than the 10th percentile for the relative MaxEnt model). The darker the blue colour, the higher the environmental suitability.

Species order: rock ptarmigan, water pipit, alpine accentor, white-winged snowfinch.

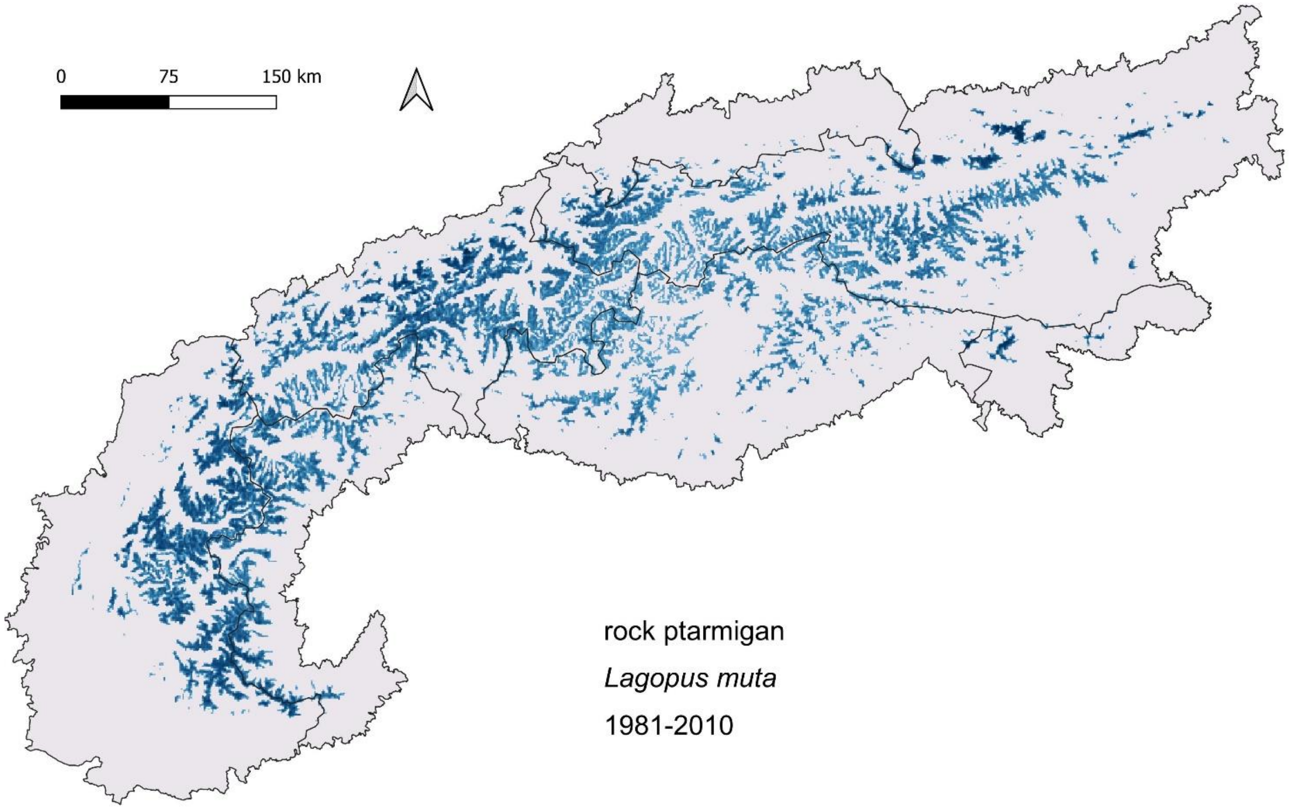
0
75
150 km
rock ptarmigan
Lagopus muta
1981-2010

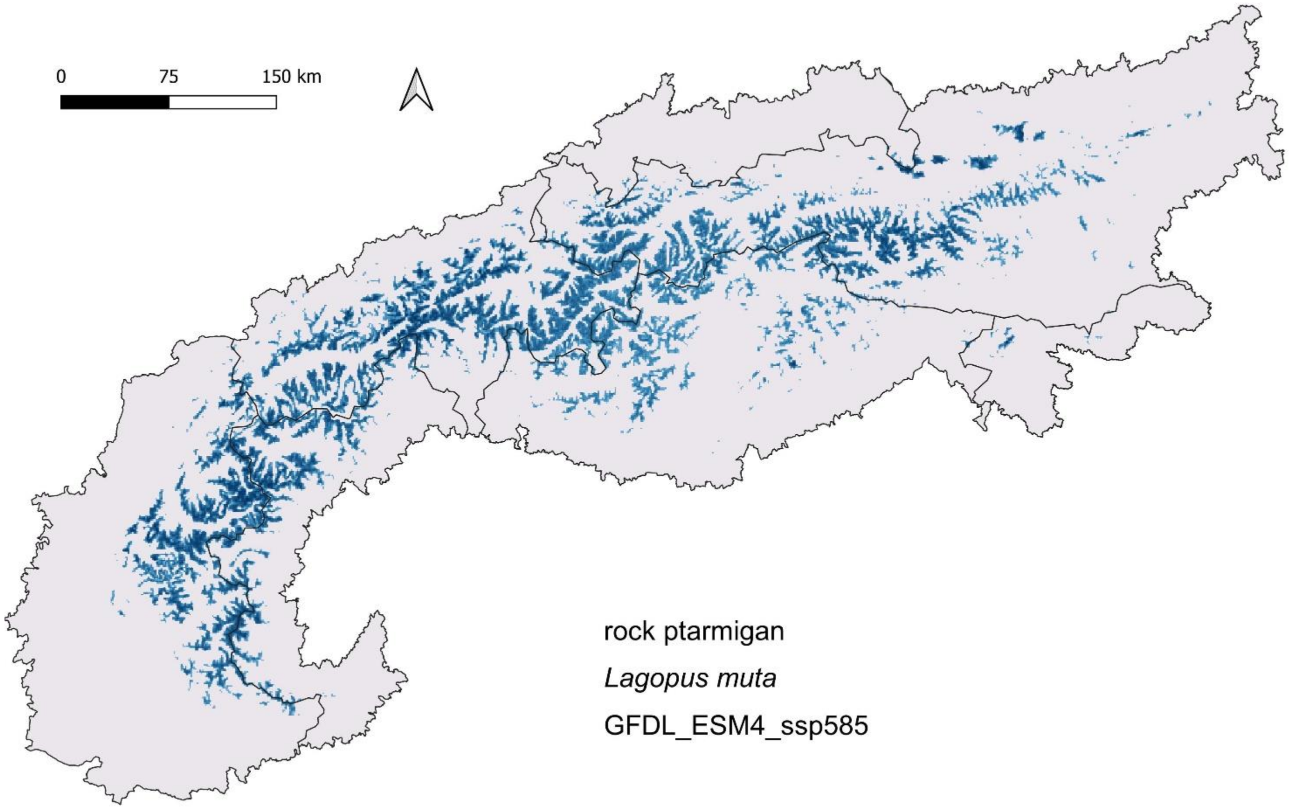
0
75
150 km
rock ptarmigan
Lagopus muta
GFDL_ESM4_ssp585

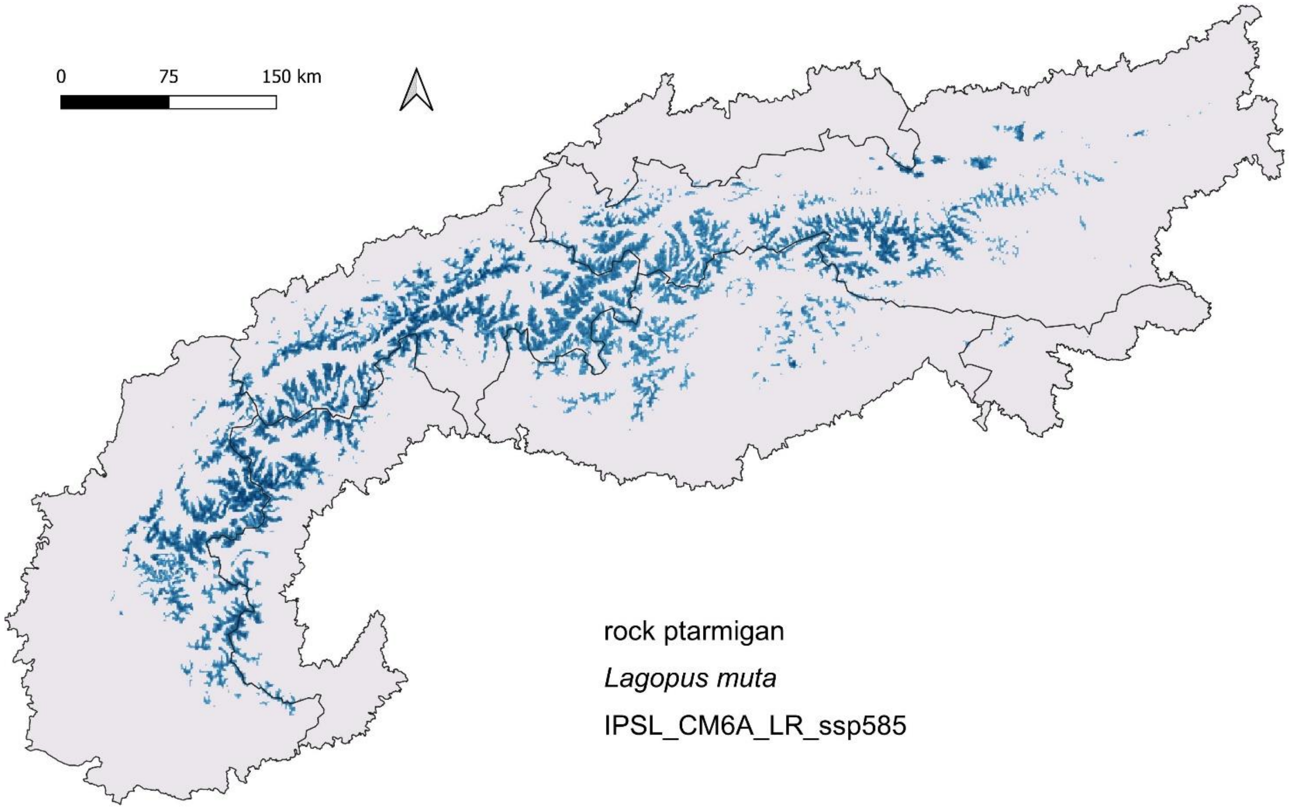
0
75
150 km
rock ptarmigan
Lagopus muta
IPSL_CM6A_LR_ssp585

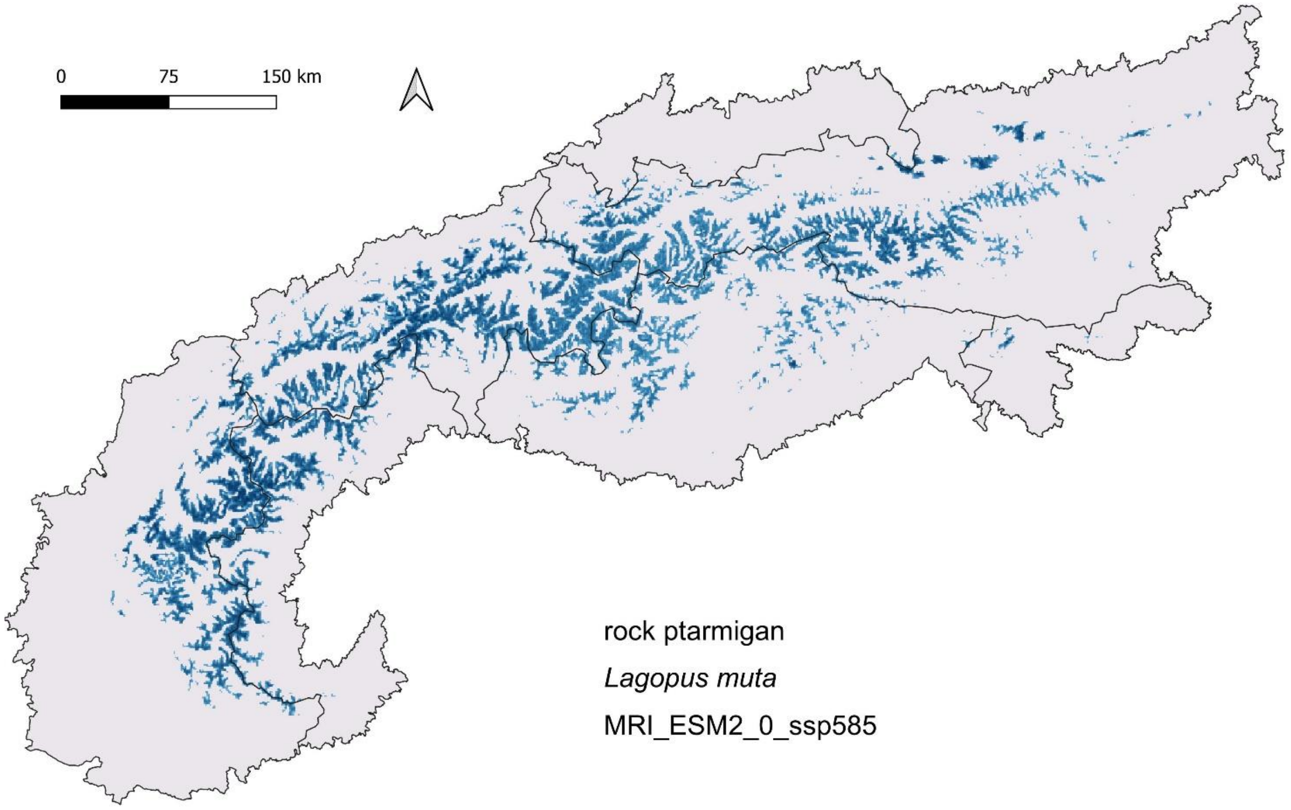
0
75
150 km
rock ptarmigan
Lagopus muta
MRI_ESM2_0_ssp585

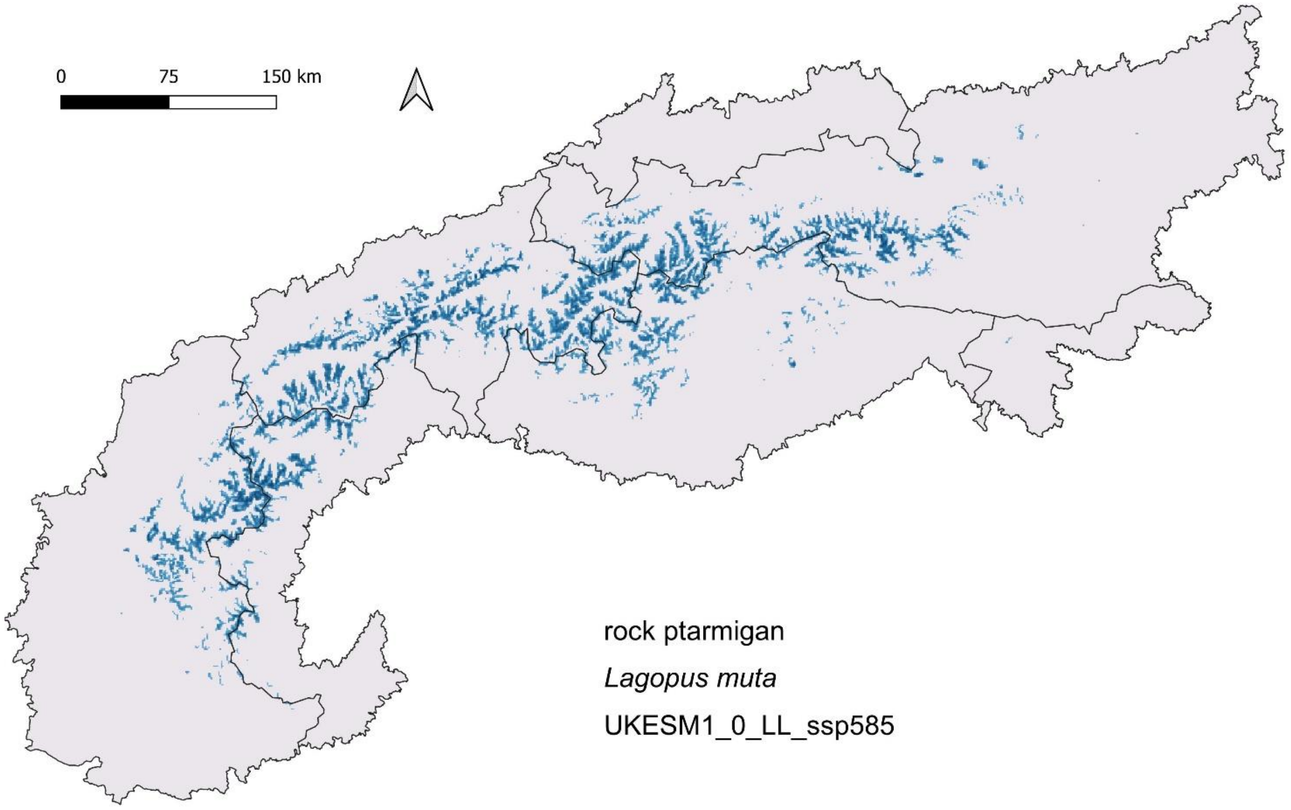

rock ptarmigan

Lagopus muta

UKESM1_0_LL_ssp585

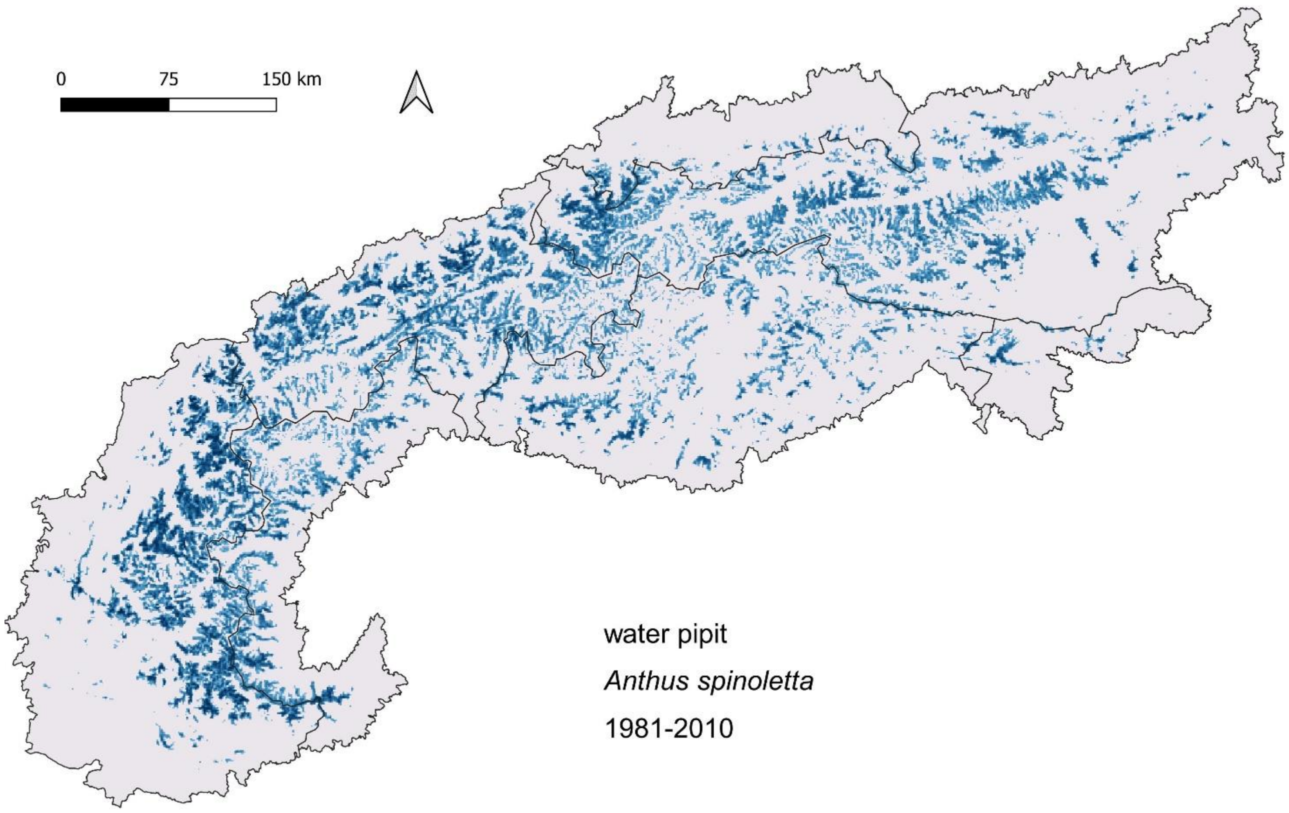
0
75
150 km
water pipit
Anthus spinoletta
1981-2010

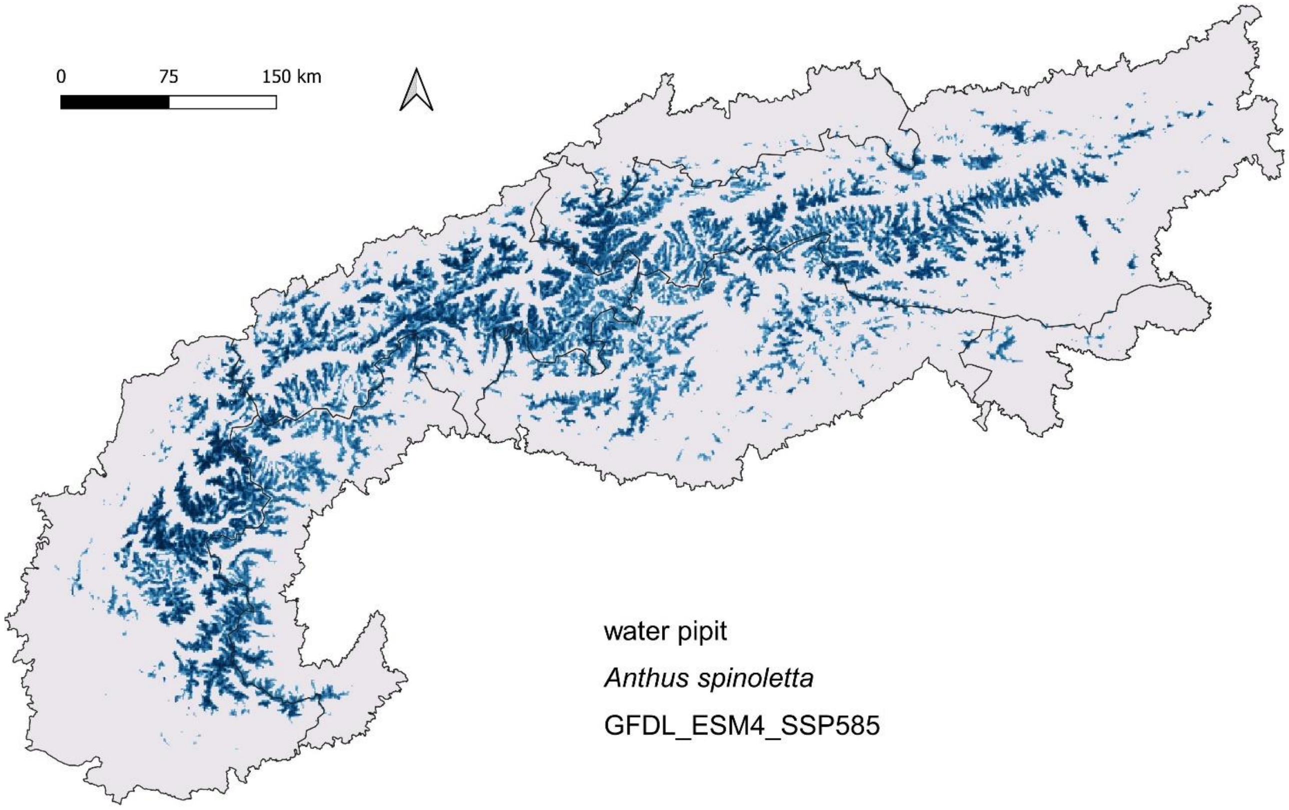
0
75
150 km
water pipit
Anthus spinoletta
GFDL_ESM4_SSP585

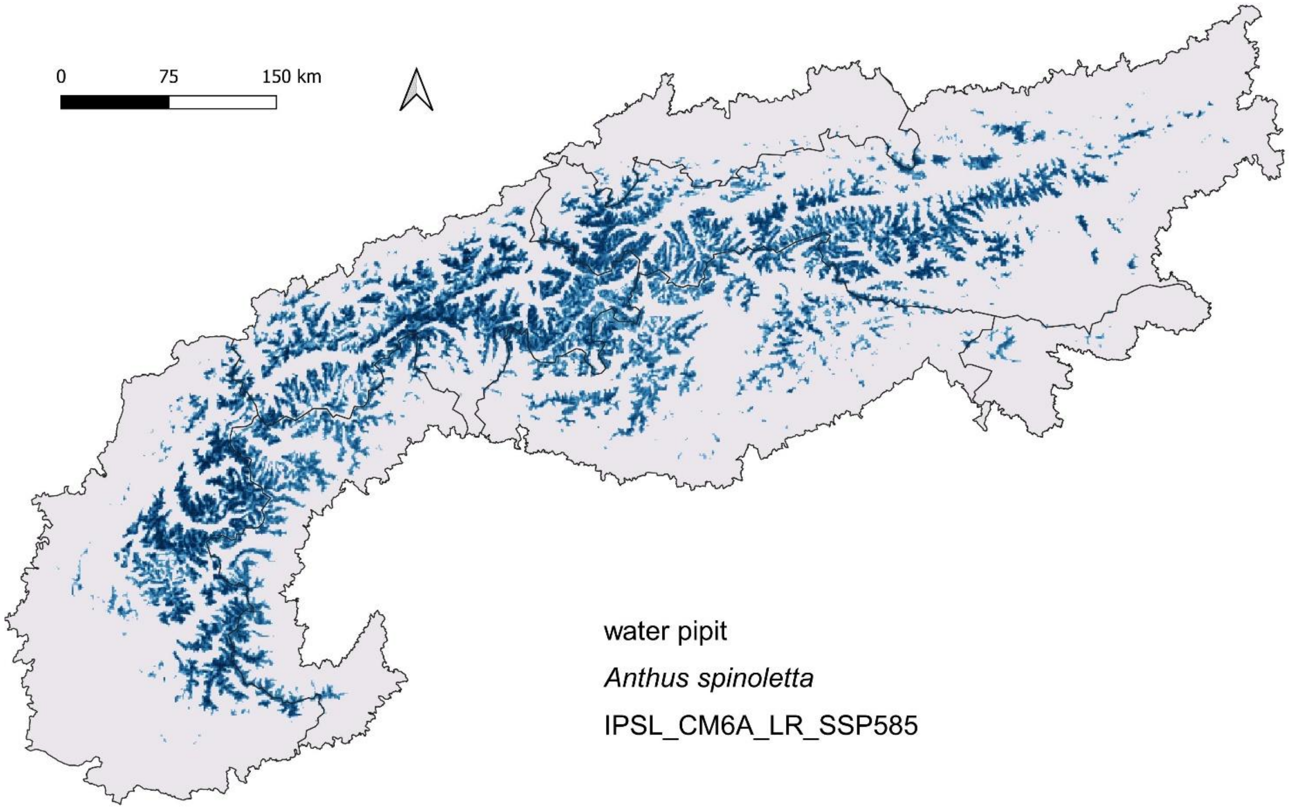
0
75
150 km
water pipit
Anthus spinoletta
IPSL_CM6A_LR_SSP585

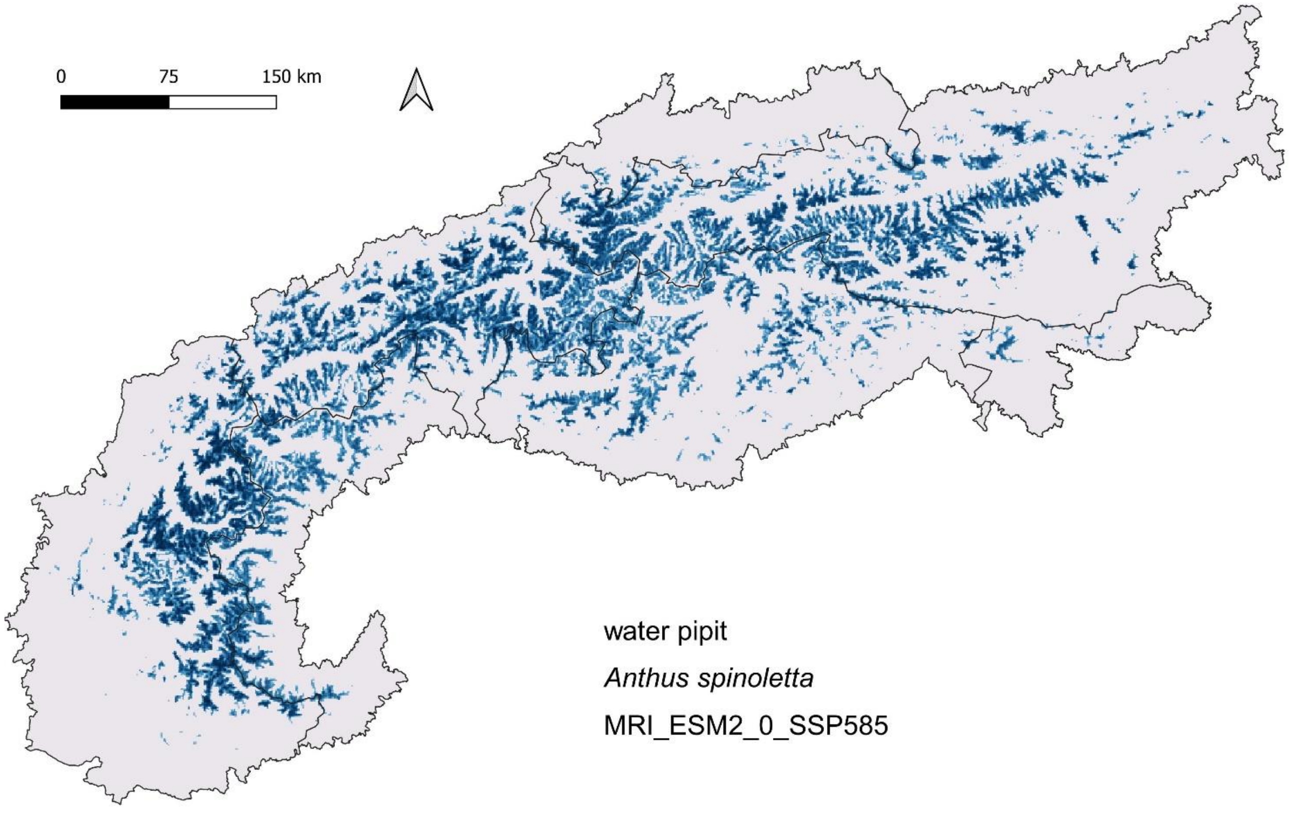
0
75
150 km
water pipit
Anthus spinoletta
MRI_ESM2_0_SSP585

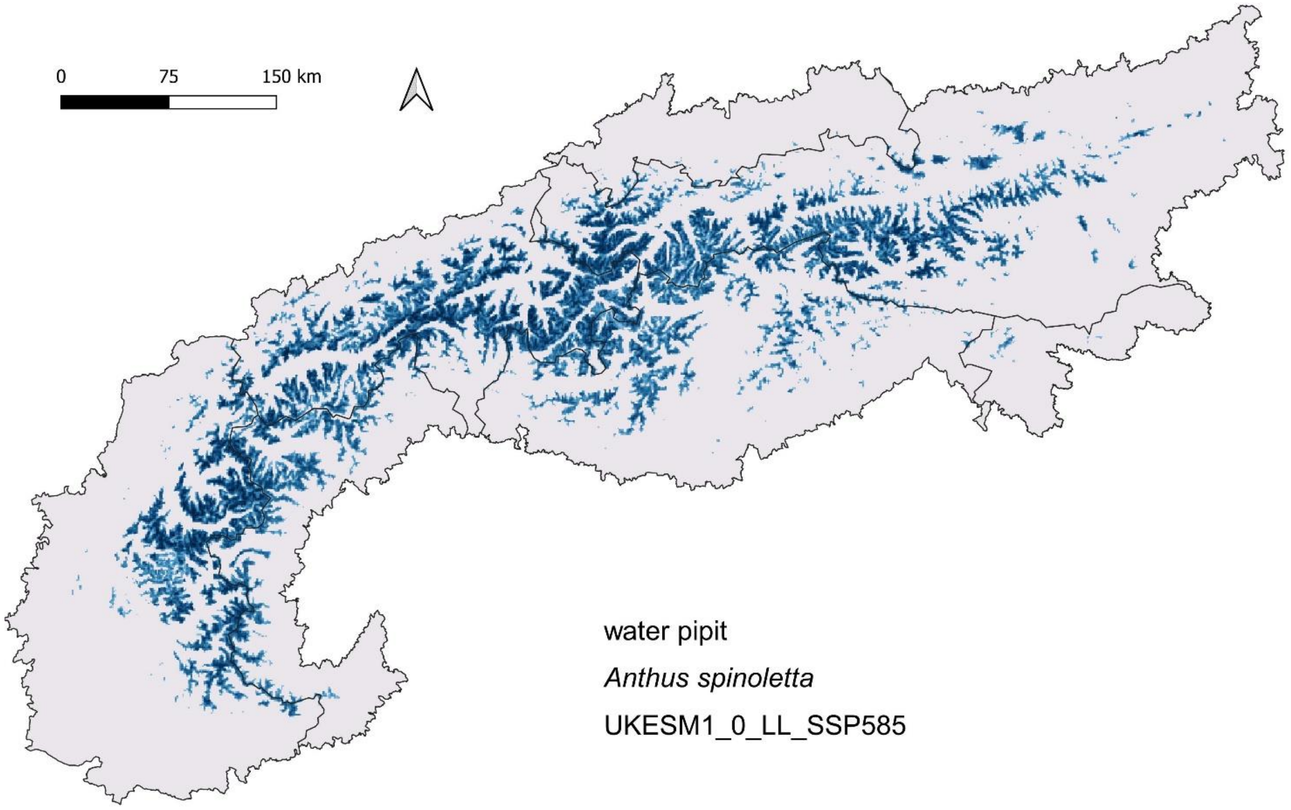

water pipit

Anthus spinoletta

UKESM1_0_LL_SSP585

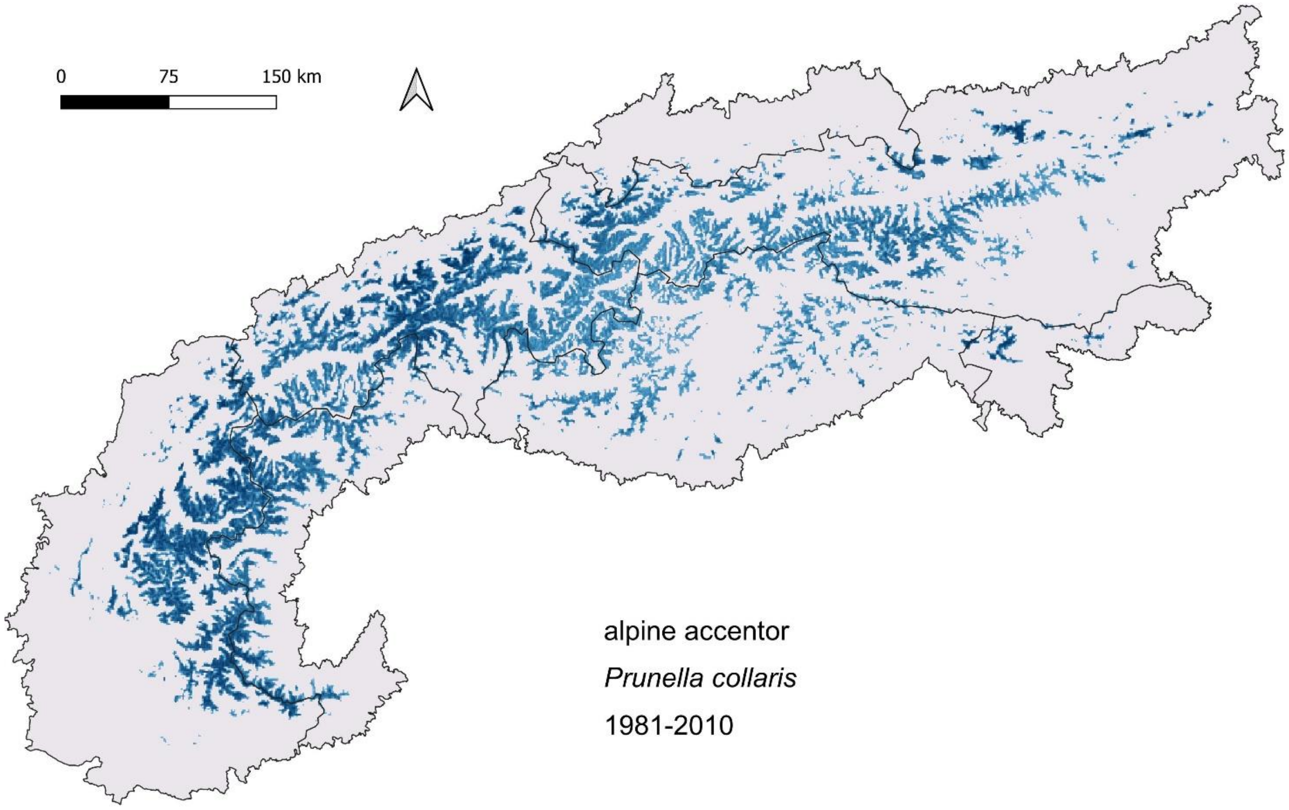
0
75
150 km
alpine accentor
Prunella collaris
1981-2010

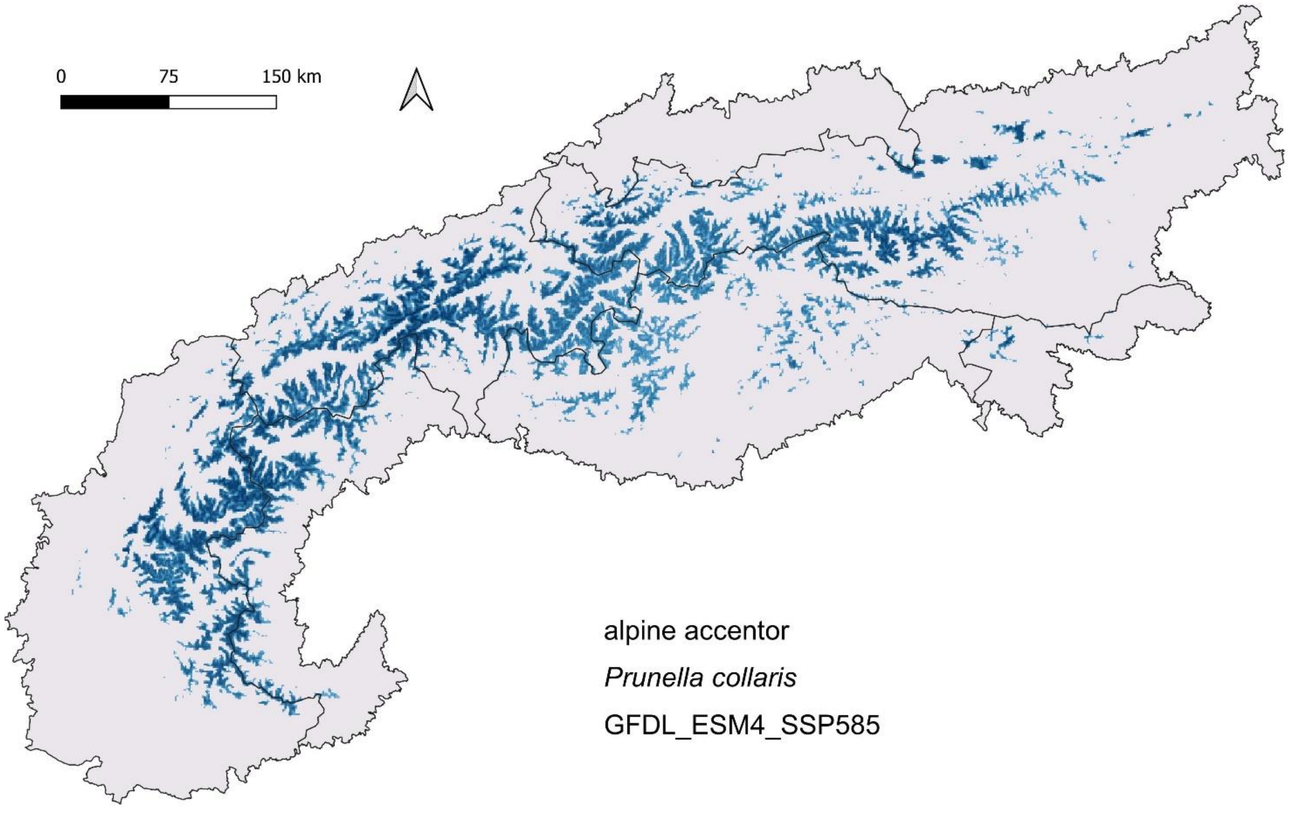
0
75
150 km
alpine accentor
Prunella collaris
GFDL_ESM4_SSP585

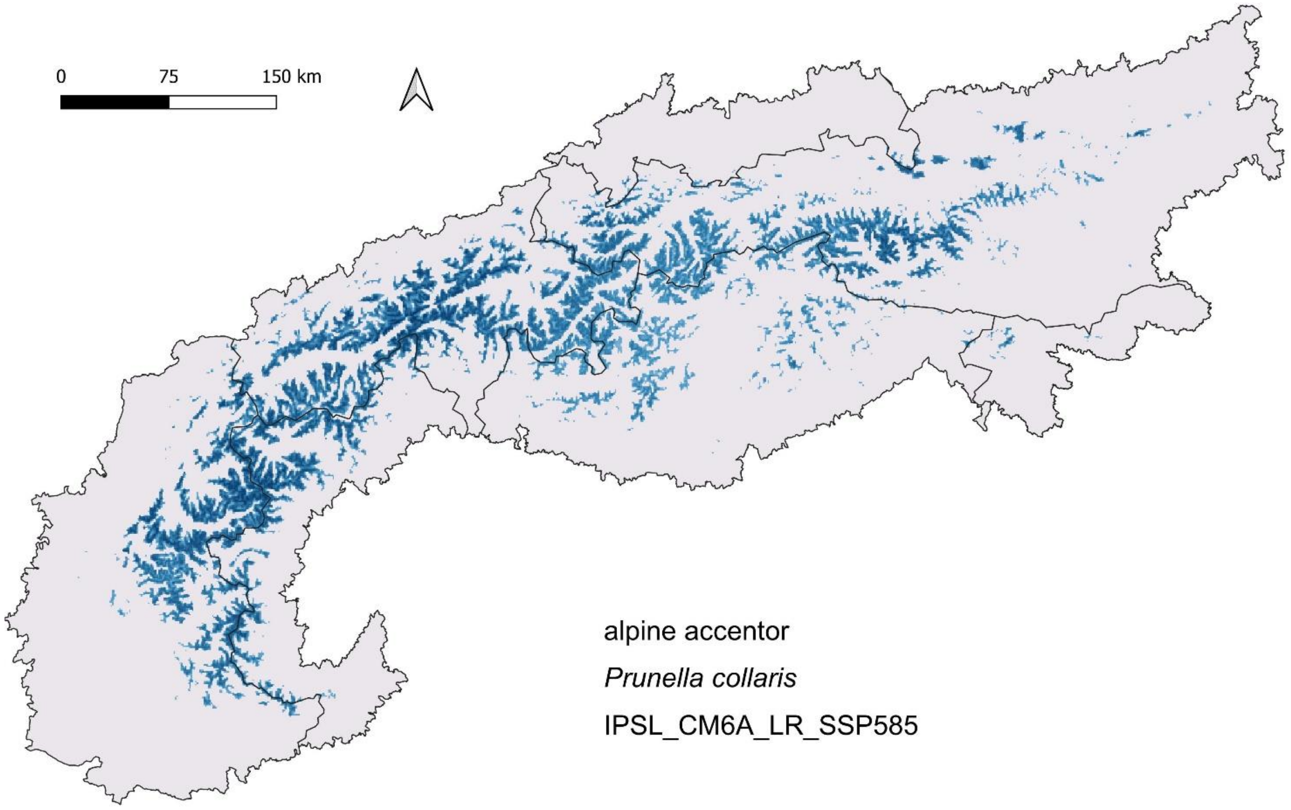
0
75
150 km
alpine accentor
Prunella collaris
IPSL_CM6A_LR_SSP585

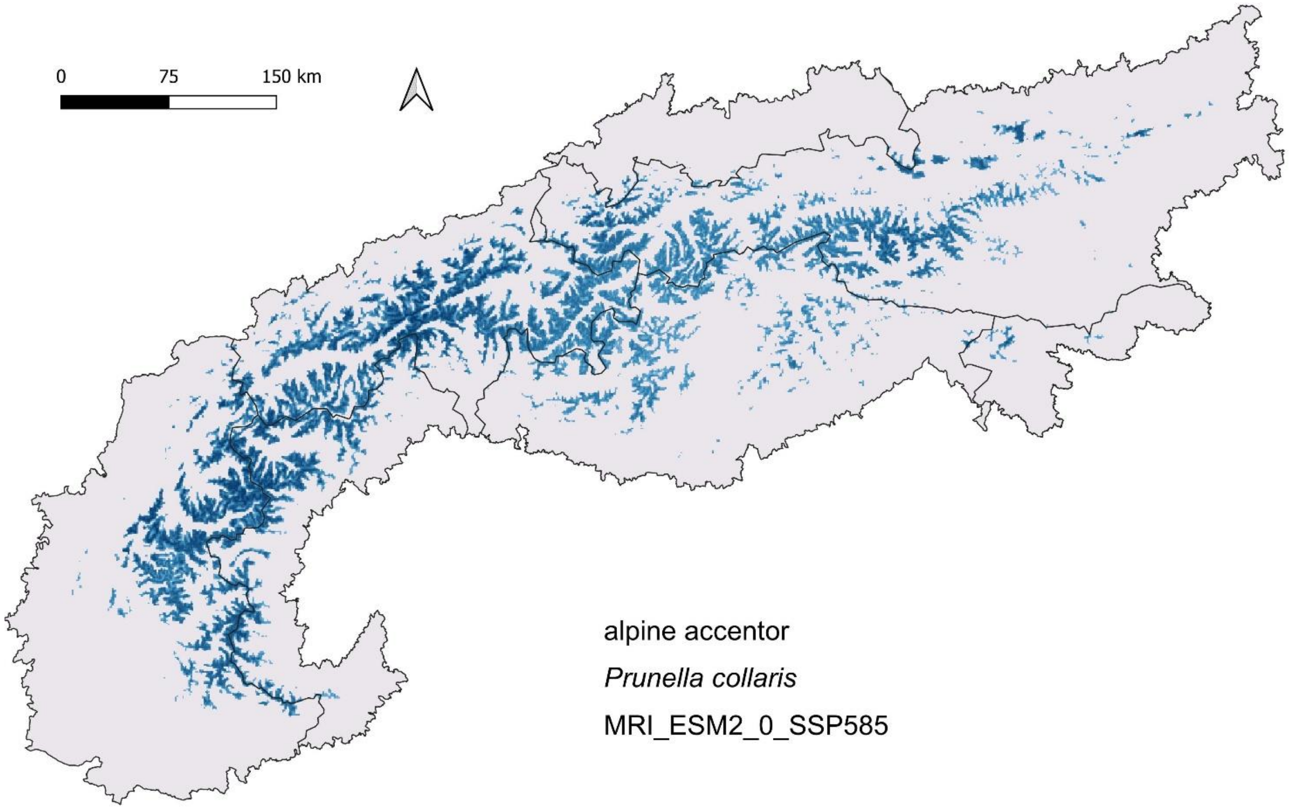
0
75
150 km
alpine accentor
Prunella collaris
MRI_ESM2_0_SSP585

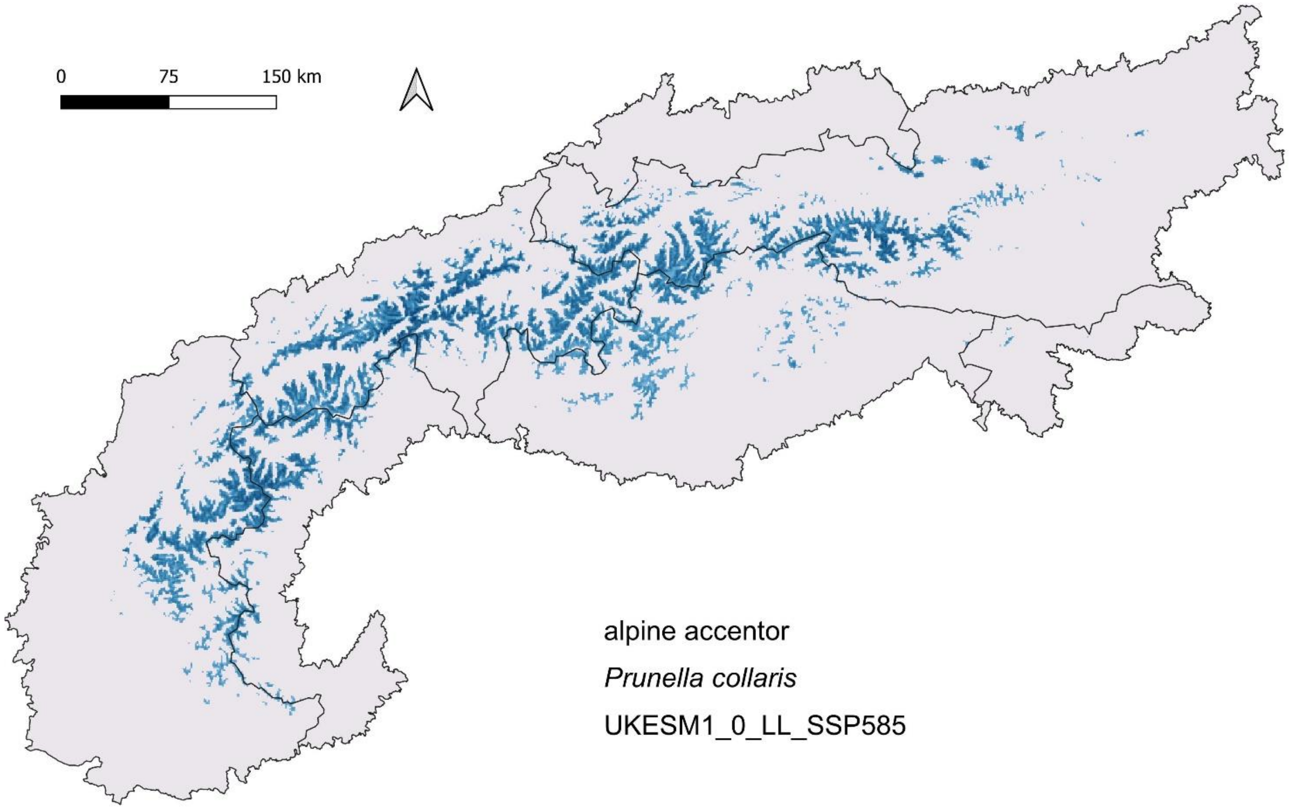

alpine accentor

Prunella collaris

UKESM1_0_LL_SSP585

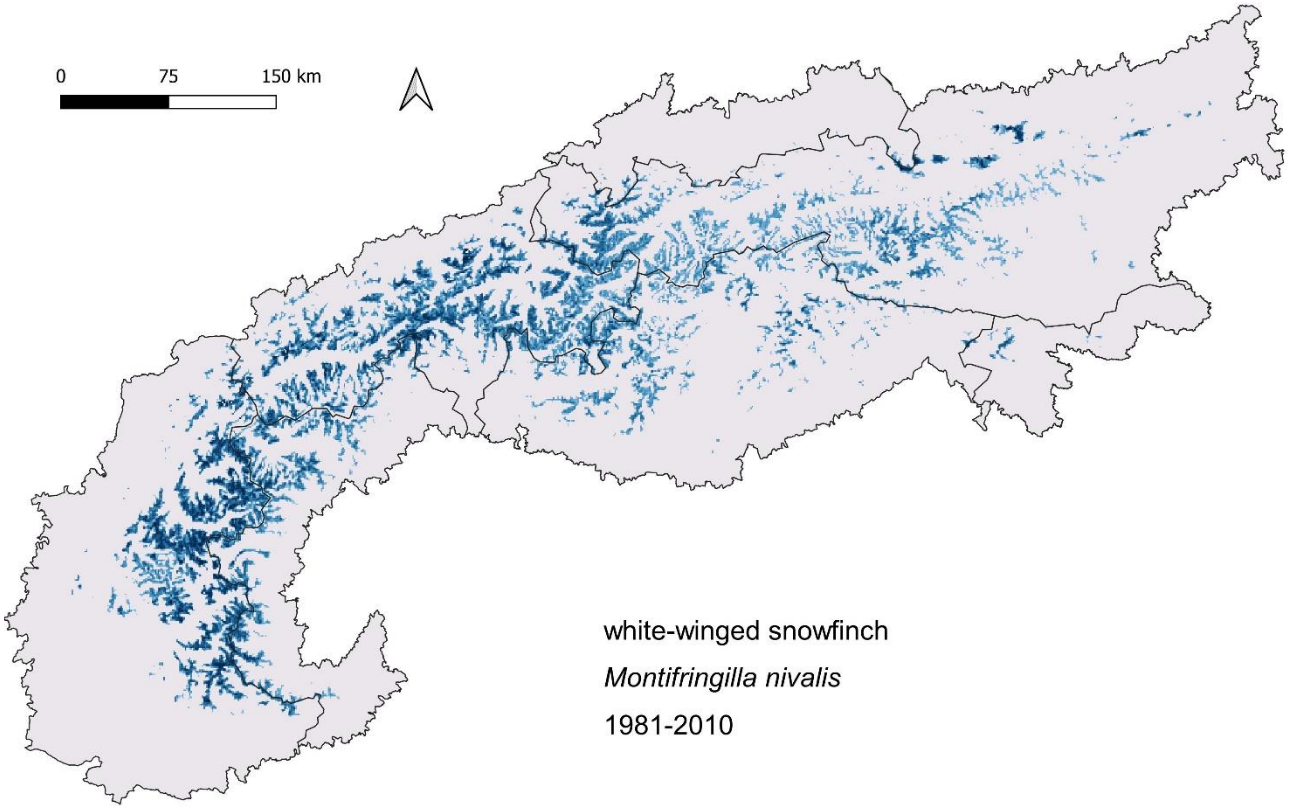
0
75
150 km
white-winged snowfinch
Montifringilla nivalis
1981-2010

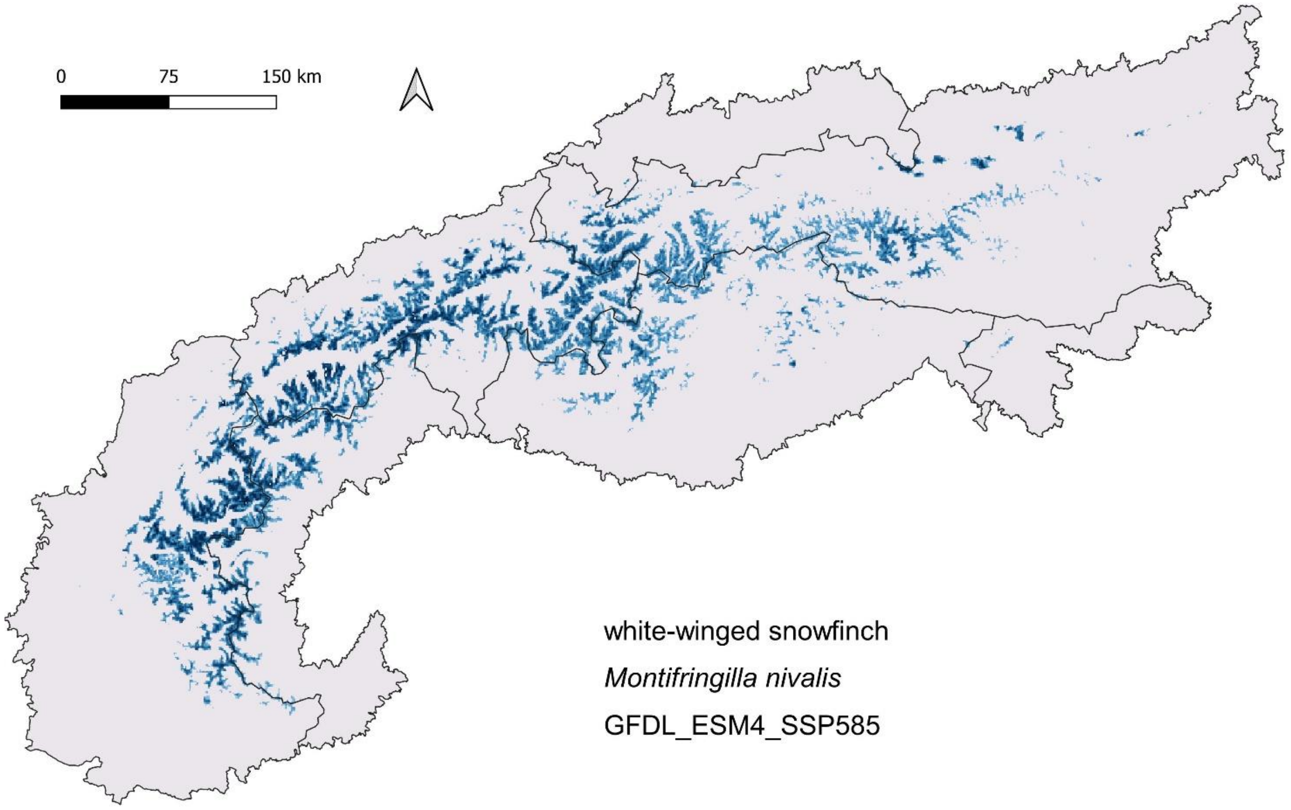
0
75
150 km
white-winged snowfinch
Montifringilla nivalis
GFDL_ESM4_SSP585

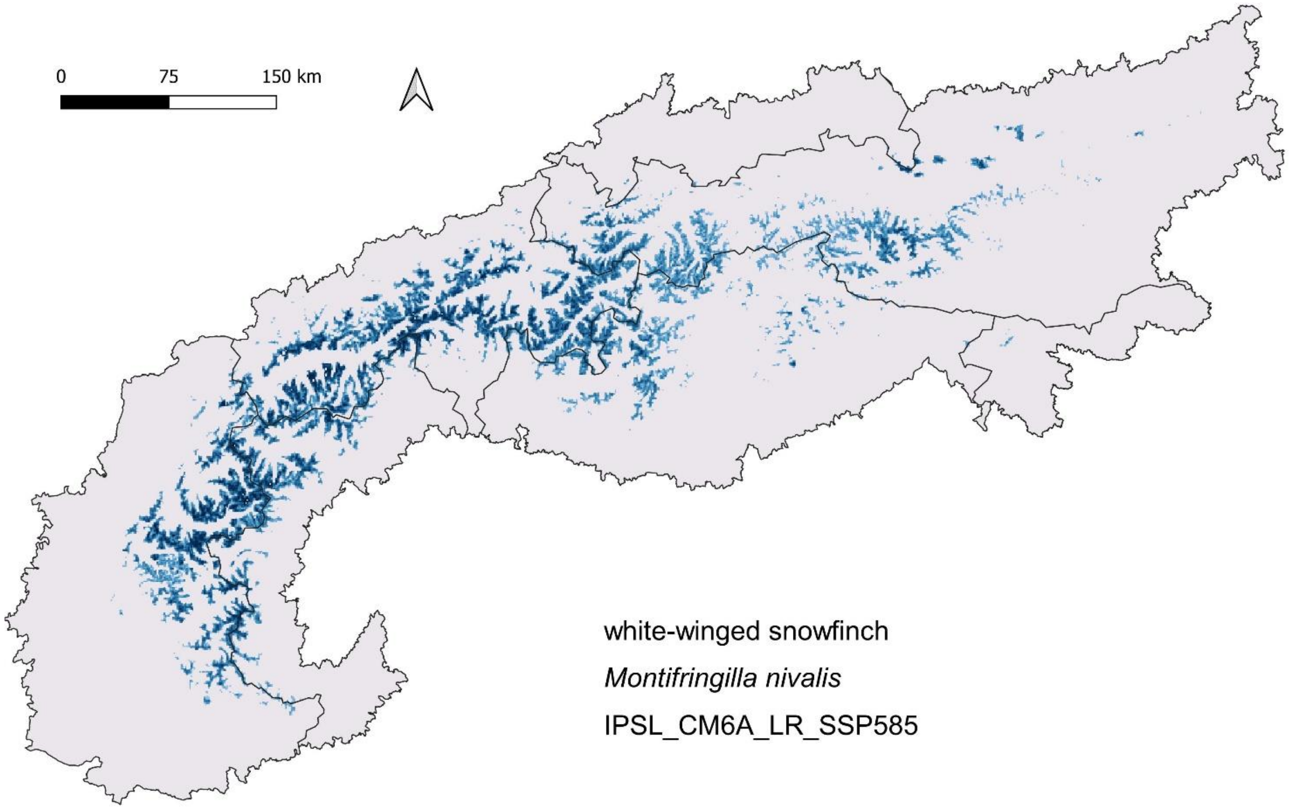
0
75
150 km
white-winged snowfinch
Montifringilla nivalis
IPSL_CM6A_LR_SSP585

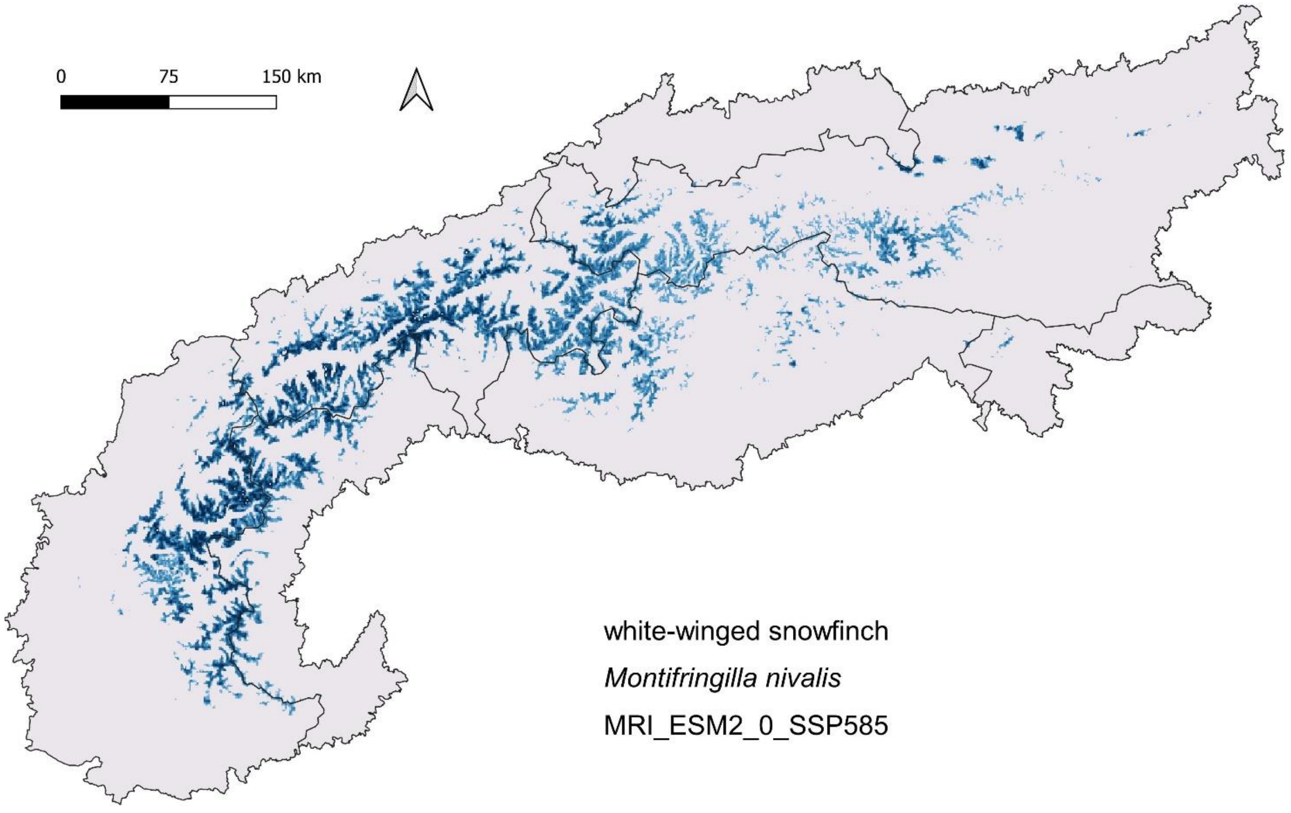
0
75
150 km
white-winged snowfinch
Montifringilla nivalis
MRI_ESM2_0_SSP585

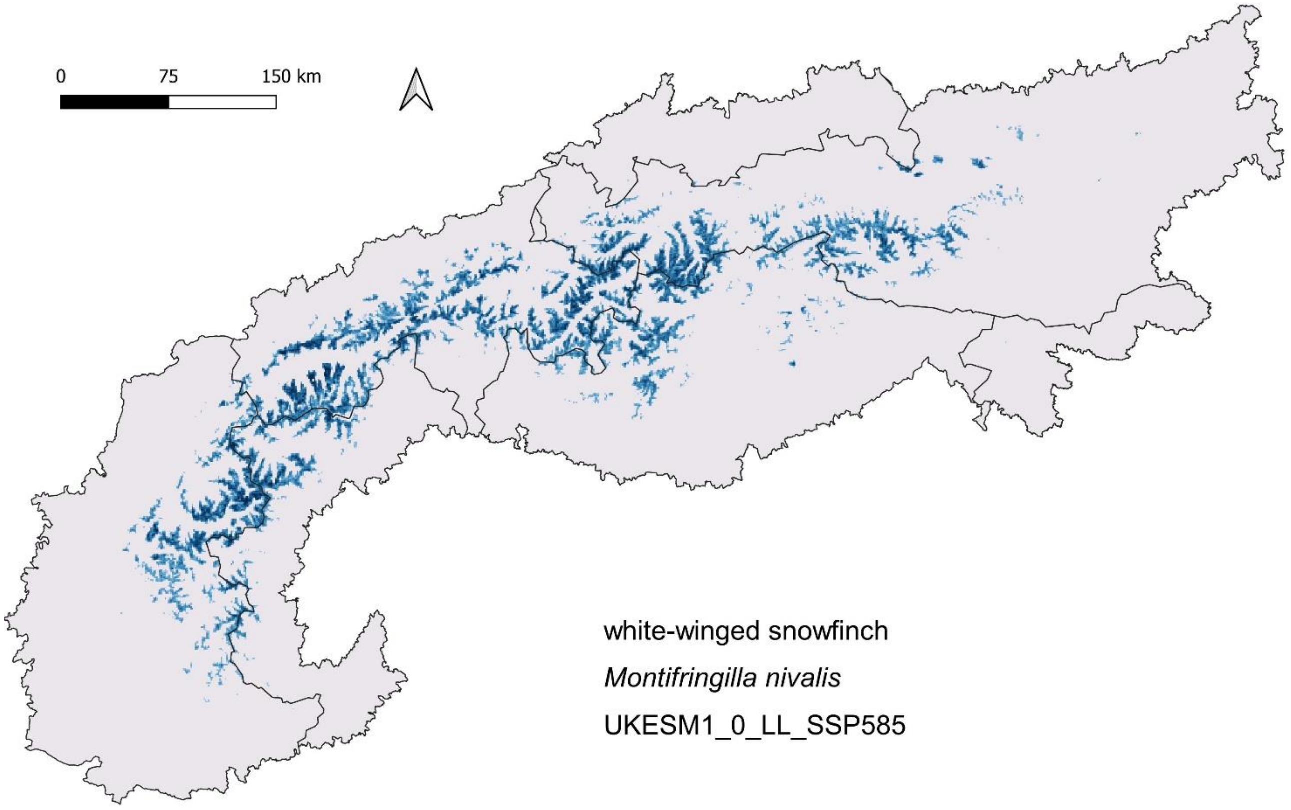

white-winged snowfinch

Montifringilla nivalis

UKESM1_0_LL_SSP585
